# Origin and Early Evolution of Hydrocharitaceae and the Ancestral Role of *Stratiotes*

**DOI:** 10.3390/plants13071008

**Published:** 2024-03-31

**Authors:** Silvia Ulrich, Manuel Vieira, Mario Coiro, Johannes M. Bouchal, Christian Geier, Bonnie F. Jacobs, Ellen D. Currano, Olaf K. Lenz, Volker Wilde, Reinhard Zetter, Friðgeir Grímsson

**Affiliations:** 1 Department of Botany and Biodiversity Research, University of Vienna, 1030 Vienna, Austria; silvia.ulrich@univie.ac.at (S.U.); johannes.martin.bouchal@univie.ac.at (J.M.B.); christian.geier@univie.ac.at (C.G.); reinhard.zetter@univie.ac.at (R.Z.); 2 Department of Historical Archaeology, Austrian Archaeological Institute (OeAI), Austrian Academy of Sciences (OeAW), 1010 Vienna, Austria; 3 Department of Earth Sciences, GeoBioTec, NOVA School of Science and Technology, Campus de Caparica, 2829-516 Caparica, Portugal; manuel.vieira@akerbp.com; 4 Department of Palaeontology, University of Vienna, 1090 Vienna, Austria; 5 Roy M. Huffington Department of Earth Sciences, Southern Methodist University, Dallas, TX 75275, USA; bjacobs@mail.smu.edu; 6 Departments of Botany and Geology & Geophysics, University of Wyoming, Laramie, WY 82071, USA; ecurrano@uwyo.edu; 7 Institute of Applied Geosciences, Technical University Darmstadt, 64287 Darmstadt, Germany; lenz@geo.tu-darmstadt.de; 8 Section Palaeobotany, Division Palaeontology and Historical Geology, Senckenberg Research Institute and Natural History Museum Frankfurt, 60325 Frankfurt am Main, Germany; volker.wilde@senckenberg.de

**Keywords:** aquatic plant, Eocene, Kenya, Messel, paleoecology, plant dispersal route, pollen morphology, tropical forest

## Abstract

The combined morphological features of *Stratiotes* (Hydrocharitaceae) pollen, observed with light and electron microscopy, make it unique among all angiosperm pollen types and easy to identify. Unfortunately, the plant is (and most likely was) insect-pollinated and produces relatively few pollen grains per flower, contributing to its apparent absence in the paleopalynological record. Here, we present fossil *Stratiotes* pollen from the Eocene of Germany (Europe) and Kenya (Africa), representing the first reliable pre-Pleistocene pollen records of this genus worldwide and the only fossils of this family discovered so far in Africa. The fossil *Stratiotes* pollen grains are described and compared to pollen from a single modern species, *Stratiotes aloides* L. The paleophytogeographic significance and paleoecological aspects of these findings are discussed in relation to the Hydrocharitaceae fossil records and molecular phylogeny, as well as the present-day distribution patterns of its modern genera.

## 1. Introduction

The origin and evolution of extant angiosperms is an ever-changing saga that has, since the birth of paleobotanical sciences, been studied using the morphology and anatomy of both living and fossil plants in combination with their current and past distribution patterns (e.g., [1,2]). In the last two decades, molecular data have become an additional source of information, not only for establishing genetic and phylogenetic relationships among angiosperms (e.g., [3,4,5]) but also for pinpointing the origin of particular lineages in both time and space. It is now the foundation for divergence time estimates and explicit biogeographic analyses (e.g., [6], and references therein). The Hydrocharitaceae, a fully aquatic family comprising 14 genera and c. 136 species (Supplementary Material S1, Table S1), is one of those families that has recently been studied using various modern approaches in attempts to clarify phylogenetic relationships among the genera and species, their time and place of origin, and subsequent divergence and dispersal patterns (e.g., [7,8,9,10]). Chen et al. [9] (p. 8) concluded that the Hydrocharitaceae had an “Oriental” (i.e., S./S.E. Asian-Chinese) origin and that the ancestor of *Stratiotes*, representing the earliest diverged lineage of the family, dispersed into Europe during the late Cretaceous and Paleocene. They suggested the genus *Stratiotes* “had diversified widely in this region adapting to wet swamps in the Late Cretaceous” [9] (p. 8), and although this “seems to be inconsistent” with the fossil record of the clade and its relatives, this discrepancy was explained by a preservation “bias in paleobotany.” Indeed, the family’s fossil record is meager and composed only of leaf, seed, and pollen specimens affiliated with certainty to *Stratiotes* L. (earliest Eocene to Holocene), *Ottelia* Pers. (middle Eocene to early Oligocene), *Hydrocharis* L. (middle Eocene to Pleistocene), *Enhalus* Rich./*Thalassia* Banks & Sol, ex K.D.Koenig (middle Eocene), *Najas* L. (late Eocene to Pleistocene), *Hydrilla* Rich. (late Eocene), and *Vallisneria* P. Micheli ex L. (late Eocene to Miocene) (Supplementary Material S1, Table S2). There is currently no fossil evidence of Hydrocharitaceae older than the earliest Eocene, and there is not a single reliable fossil record representing this family from South or Southeast Asia or China, the supposed area of origin ([9] “Oriental area”; Supplementary Material S1, Table S2).

*Stratiotes* is a monotypic genus today. The single living species, *Stratiotes aloides* L., is distributed throughout Europe and western Central Asia (see Figure 8 in [11]). The plant is a peculiar dioecious perennial, which is submerged in freshwater over winter and rises to the surface to flower in spring. The roots of *S. aloides* are simple, up to 180 cm long, and loosely attached to the submerged substrate. The linear, sessile, and rigid leaves are up to 160 cm long, spirotristichous, in a rosette, and with spinous-serrate margins. The male inflorescences are 3–6-flowered, but the females bear mostly single flowers. Male flowers have 5–17 stamens surrounded by 20–30 nectaries; female flowers have six pistils surrounded by 20–30 nectaries [11,12]. *Stratiotes aloides* is entomophilous and mainly pollinated by Diptera flies [13]. The insects are attracted to the flowers by their large white petals, osmophores emitting a smell similar to that of rotten meat, and nectaries [12,14,15]. The pollen grains of *S. aloides* are isodiametric and spheroidal in shape. They are inaperturate, 42–87 µm in diameter, and characterized by a reticulate sculpture with echinate suprasculpture (having twisted and furrowed echini), and numerous sculpture elements (nanoclavate and nanogemmate) within the lumina (Supplementary Material S1, Table S3). The sculpture, when observed with scanning electron microscopy (SEM), makes pollen of *S. aloides* unique within the Hydrocharitaceae (e.g., [16,17]), and in combination with pollen shape, size, and aperture configuration, it can be differentiated from any other angiosperm pollen. The fruit of *S. aloides* is a berry-like capsule containing up to 24 seeds [11]. Seeds are well represented in the fossil record, extending from the beginning of the Eocene to the present time across Europe and into Western Siberia (Supplementary Material S1, Table S2; part of the “West Palearctic” area of [9]). The seeds are borne in fruits that are forced below the water by developing leaves, then sink to the bottom in a gelatinous mass [11], and consequently are more likely than other plant parts to become fossils. Interestingly, *S. aloides* often reproduces vegetatively via stolons or turions [18], but only leaves (or parts) and seeds have been recognized or reported as fossils.

Here we report on the first proof of pre-Pleistocene fossil *Stratiotes* pollen from the early Eocene of Messel, Germany (Figure 1) (i.e., [9] “West Palearctic” area), and the “earliest” late Eocene of Kenya (Figure 2) (i.e., [9] “Afrotropical” area). The German and Kenyan pollen types are compared to those produced by modern *Stratiotes aloides*. The new finds are discussed within the framework of previous fossil occurrences and related (paleo)environmental data, which are compared with extant *Stratiotes,* ecology, and climate preferences. A dated phylogeny and biogeographic analysis based on the fossil record of Hydrocharitaceae are presented and discussed with regard to the origins and dispersal routes of different Hydrocharitaceae genera.

## 2. Results

### 2.1. Systematic Description

Order: ALISMATALES R.Br ex Bercht & J.Presl

Family: HYDROCHARITACEAE Juss.

Genus: *Stratiotes* L.

Species: *Stratiotes* sp., Messel morphotype (MT), pollen close to *Stratiotes aloides* (Figure 3; Supplementary Material S1, Table S3).

“*Description*”: Pollen, monad, P/E ratio isodiametric, outline circular to elliptic; diameter 21–25 μm in LM, 18–23 μm in SEM (Figure 3A–C); inaperturate; exine ca. 1.8 μm thick (excluding echini) in LM; semitectate; sculpture echinate in LM, reticulate with echinate suprasculpture in SEM, and nanogemmate, nanoareolate to nanorugulate inside lumina (SEM; Figure 3D–G); echini 3–3.5 μm in height, 10–12 per 100 µm^2^ pollen surface, straight to twisted, sometimes fused (Figure 3F,G), with furrows extending from base towards apex (SEM); exine ca. 1.8 µm thick (LM), composed of three layers (transmission electron microscopy [TEM]; Figure 3H–J); thin compact-discontinuous endexine; thick compact-continuous footlayer; alveolate-granular infratectum with varying granulae clusters; discontinuous tectum (semitectate); and supratectal elements echini (TEM; Figure 3H–J).

“*Remarks*”: The fossil *Stratiotes* pollen grain (Messel MT) from the early Eocene of Messel, Germany, shares diagnostic characteristics with fossil Stratiotes pollen from the earliest late Eocene of Kenya (Dodori MT; Figure 4 and Figure 5), as well as extant pollen of *S. aloides* in LM, SEM, and TEM (Figure 6; Supplementary Material S1, Table S3; Supplementary Material S3, Figures S1–S4). Some morphological (SEM) and ultrastructural (TEM) differences between the Messel MT and extant *Stratiotes* pollen can be observed. First, the Messel MT pollen is eroded; it is infolded and has lost much of the sexine/tectum layer of the pollen wall. Many of the echini, which are usually abundant and seen as supratectal elements on the muri of the reticulum, are missing. Still, remnants of the muri can be seen in Figure 3D,G (arrows). Despite the poor preservation state, the pollen grain can be affiliated with the genus *Stratiotes* based on, among others, the morphology of the echini, showing the characteristic furrows that run from the base of the echinus towards the apex and the ultrastructure of the pollen wall observed with TEM. The main difference between extant *Stratiotes* pollen and the Messel MT is in size (Supplementary Material S1, Table S3), with the fossil being much smaller (which could be caused by infolding or missing parts in the fossil pollen). Also, the ultrastructure of the Messel MT pollen shows that the infratectum is denser in the fossil compared to that observed in extant pollen, and the footlayer is continuous-compact in the Messel MT versus discontinuous in extant *Stratiotes* pollen (compare Figure 3J with Figure 6J).

Hydrocharitaceae leaves have been reported from Messel [22]. Surprisingly, the characteristic seeds of *Stratiotes* have not been recorded among the rich record of fruits and seeds [23]. Also, in a study on dispersed pollen from Messel [24], Thiele-Pfeiffer described a new species, *Punctilongisulcites microechinatus* Thiele-Pfeiffer, which was assigned to Hydrocharitaceae. The author discussed *Stratiotes* as one of the few potential source genera. However, she was not able to recognize the characteristic reticulate pattern between the spines and left the generic assignment open. Bouchal et al. [25] have now shown that the *Punctilongisulcites microechinatus* pollen type of Thiele-Pfeiffer [24] is sulcate and affiliated with Arecaceae.

Species: *Stratiotes* sp., Dodori MT, pollen close to *Stratiotes aloides* (Figure 4 and Figure 5; Supplementary Material S1, Table S3).

“*Description*”: Pollen, monad, P/E ratio isodiametric, shape spheroidal, circular in polar and equatorial views; diameter 48–60 μm in LM, 50–56 μm in SEM (Figure 4A–C and Figure 5A,B); inaperturate; exine 1.7–2.9 μm thick (excluding echini) in LM; semitectate; sculpture reticulate and echinate in LM, reticulate with echinate suprasculpture in SEM, and nanogemmate to microgemmate inside lumina (Figure 4D–E and Figure 5C–E); muri forming ± pentagonal pattern, echini at intersection of muri; muri rounded, sometimes discontinuous (Figure 4D and Figure 5E), 1.4–5.5 μm long, 0.6–1.2 μm wide (SEM); lumina 3.6–9.8 μm in diameter (SEM); echini 4.5–5.5 μm in height, 3–10 per 100 µm^2^ pollen surface, straight or twisted (Figure 4F and Figure 5C,D), with three furrows extending from the base towards apex (SEM); exine 1.7–2.9 µm thick, composed of three layers (TEM; Figure 4H–J and Figure 5F,G); compact-discontinuous endexine; continuous footlayer; alveolate to granular infratectum with varying granulae clusters; discontinuous tectum (semitectate); and supratectal elements echini (TEM).

“*Remarks*”: The fossil *Stratiotes* pollen (Dodori MT) from the earliest late Eocene of Kenya, Africa, shares diagnostic characteristics with extant pollen of *S. aloides* in LM, SEM, and TEM (Figure S4; Supplementary Material S1, Table S3; Supplementary Material S3, Figure S1–S4). Some morphological (SEM) and ultrastructural (TEM) differences between the fossil and extant pollen can be observed. The sculpture elements of *S. aloides* pollen are shorter and narrower but more frequent than those of the Dodori MT (SEM). The muri are usually longer and broader in the Dodori MT, and the lumina are generally larger (SEM). The echini are taller in the Dodori MT and fewer in number per 100 μm^2^ pollen surface. Also, sculpture elements inside lumina are nanogemmate to microgemmate, but nanoclavate to nanogemmate in pollen of *S. aloides* (SEM) (Supplementary Material S1, Table S3). The ultrastructure of the Dodori MT pollen wall is only slightly different from that of extant *S. aloides*, mainly in terms of thickness. The infratectum of the Dodori MT is more compact than that of the *S. aloides*, the supratectal elements (echini) in the fossil are also larger than in the modern pollen, and the footlayer is continuous-compact in the Dodori MT versus discontinuous in extant *Stratiotes* pollen (compare Figure 4J with Figure 6J).

We are not aware of any previous Hydrocharitaceae (*Stratiotes*) fossil records from this locality or of any other Cenozoic plant-bearing sediments from Africa.

### 2.2. Phylogeny of Hydrocharitaceae

Our IQTREE and MrBayes analysis of the molecular data from Bernardini and Lucchese [10] retrieved a strongly supported topology coherent with this previous study (Supplementary Material S4). All genera are retrieved as monophyletic with good support. Two major clades are retrieved with strong support: a clade including *Lagarosiphon* Harv. as sister to *Elodea* Michx sensu lato and *Ottelia* plus *Blyxa* Noronha ex Thouars (clade B) (100/100/1 Bootstrap/aLRT/Posterior Probability), and a clade including *Limnobium* Rich. plus *Hydrocaris* as sister to a clade of *Najas* plus a clade of the seagrasses (*Halophila* Thouars, *Enhalus*, and *Thalassia*) plus a clade of *Hydrilla* as sister to *Nechamandra* Planch. plus *Vallisneria* (clade A) (82.5/83/1). The placement of *Stratiotes* as sister to the rest of the Hydrocharitaceae was supported, albeit weakly, in IQTREE (75.3/80), but not in the Bayesian analysis.

### 2.3. Dated Phylogeny

Our dated phylogeny inferred the origin of crown-group Hydrocharitaceae between the late Cretaceous and the Paleogene (67–56 Ma) (Figure 7). The divergence of *Stratiotes* from the rest of the family is estimated to have happened between 64 and 50 Ma. Clade A appears to be slightly younger than clade B, the former having originated between 55 and 45 Ma and the latter having originated between 61 and 47 Ma. The genus *Hydrilla* diverged rather early from the *Nechamandra*-*Vallisneria* clade (42–31 Ma). Among the crown groups of the extant genera, the oldest is *Najas*, which originated between 28 and 14 Ma. Of similar age are the *Ottelia-Blyxa* clade (33–14 Ma), the *Nechamandra*-*Vallisneria* clade (27–13 Ma), the Seagrass clade (27–11 Ma), and the genus *Lagarosiphon* (32–10 Ma). The genus *Elodea sensu lato* originated between 22 and 8 Ma, and the genus *Vallisneria* between 17 and 8 Ma. Younger are the crown groups of the genus *Ottelia* (15–5 Ma), the genus *Blyxa* (16–4 Ma), and the genus *Hydrocharis sensu lato* (13–5 Ma).

### 2.4. Biogeographic Analysis

The results of our biogeographical analysis show that most of the ancestral nodes of Hydrocharitaceae were distributed in Europe (Figure 8). The crown node of clade A is inferred to have had a wider distribution including Europe, Africa, and South America, with extirpation leading to an African origin of *Lagarosiphon*, a South American origin of *Elodea sensu lato*, and a European origin of the stem of the *Ottelia*-*Blyxa* clade. This latter group has a much more complex later history, indicating a widespread distribution of *Blyxa* and an Australian–Southeastern origin of *Ottelia.* Representatives of clade B were distributed in Europe for most of their evolutionary history, with the exception of the seagrasses, which appear to have obtained a widespread distribution early in their history. The current rather widespread distribution of *Hydrocharis* and *Najas* appears to have been the result of recent dispersals outside Europe (i.e., through North America or through Southeast Asia), while *Vallisneria* shows an ancestral distribution in Southeast Asia with later dispersal to the Americas.

### 2.5. Climate Preferences Hydrocharitaceae

To estimate the paleoclimatic preferences of *Stratiotes* and other Hyrdocharitaceae, we compiled geographic occurrences and climatic preferences (in total 37.498 data sets, Supplementary Material S1, Table S5) of 107 species belonging to 13 out of the 14 extant Hyrdocharitaceae genera listed in POWO [26]. We used GBIF and prevailing climate across species distribution areas ([27], Supplementary Material S2). For practical reasons, the climatic aspects of Hydrocharitaceae are here discussed by genera and clades following the phylogenetic framework of Chen et al. [9]. Since Hydrocharitaceae grow submerged in waterbodies, precipitation is not a restricting factor. Therefore, we omitted the second letter of the Köppen climate types for this family.

*Stratiotes* is the first diverging genus in the family’s phylogenetic tree. It is monotypic and presently restricted to western Eurasia, where it thrives mainly under temperate and snow climates, with warm (Cfb, Dfb) or cool to short summers (Dfc) (Figure 9). In the Po valley, Italy, the northern coast of the Black Sea, and north of the Caspian Sea, between Dnepr and Wolga, this species extends into humid temperate and snow climates with hot summers (Cfa, Dfa). Within Hydrocharitaceae, *Stratiotes* is today the only genus confined to temperate climates.

*Lagorosiphon* and *Appertiella* C.D.K.Cook et L.Triest belong to the first diverging group in Clade B and show a Madagascan and African distribution. Here, *Lagorosiphon* occupies fully tropical climates (Aw), a variety of hot and cold arid steppe and desert climates (B-climates), and temperate climates with hot and warm summers (C-climates) (Figure 9). No occurrence data was available for *Appertiella*. The second group in Clade B includes *Egeria* Planch. and *Elodea* [9]. *Egeria* is restricted to South America and thrives under tropical (mainly Aw; extending into Af, Am) and arid desert and steppe climates with hot summers (mainly BSh; extending into BWh) but is also found in temperate climates with hot to warm summers (mainly Cfa; extending into Cfb, Cwa, Cwb). *Elodea* is native to North and South America, but absent in Central America. In the Americas, this genus shows the widest climatic range, present in fully tropical (Af, Am, Aw) and in hot and arid desert and steppe climates (B-climates). It is mainly present in temperate and snow climates where summers are either hot or warm (various C- and D-climates). Some species [*Elodea bifoliata* H.St.John, *E. canadensis* Michx., *E. nutalli* (Planch.) H.St.John] extends this range into snow climates with cold and short summers (Dfc, Dsc) (Figure 9). The last group in Clade B consists of *Blyxa* and *Ottelia*. The geographic distribution of both genera includes Africa, India, East Asia, and most of Australasia, with only *Ottelia* extending into South America. *Blyxa* is present in tropical (Af, Am, Aw) and temperate climates with hot to warm summers (Cfa, Cfb, Cwa, Cwb). Only some species extend into arid [BSh, BWh; *Blyxa auberti* Rich., *B. echinosperma* (C.B.Clarke) Hook.f., *B. octandra* (Roxb.) Planch. ex Thwaites] and snow climates [Dwa, Dwb; *B. auberti, B. japonica* (Miq.) Maxim, ex Asch. et Gürke]. *Ottelia* grows in tropical (mainly Aw, extending into Af, Am), arid climates (mainly BSh; extending into BSk, BWh, BWk) as well as temperate climates with hot or warm summers (mainly Cfa, Cfb, Cwa, Cwb; extending into Csa, Csb). Only *Ottelia alismoides* (L.) Pers. occurs in snow climates with hot or warm summers (Dfa, Dfb) (Figure 9).

The first diverging group in Clade A comprises *Hydrocharis* (incl. *Limnobium*), which occurs on all continents except Antarctica. Its climatic preferences range from fully tropical (mainly Aw, Am; extending into Af) to temperate and snow climates with hot to warm summers (mainly C-climates, extending into D-climates) and occasionally into snow climates with cold and short summers (Dfc, Dwc). Only *Hydrocharis morus-ranae* L. is widespread in the Dfc climate of the Eurasian Taiga (Figure 9). The second diverging group in Clade A includes all marine members of Hydrocharitaceae (*Thalassia*, *Halophila*, and *Enhalus*). All taxa in this group are mainly found between the Tropic of Cancer and the Tropic of Capricorn. There, they prevail in fully tropical (Af, Aw, Am), arid (mainly BSh, BWh; extending into BSk, BWk), and temperate climates (mainly Cfa, Csa). *Najas* forms the third group diverging in Clade A, showing a nearly global distribution with a wide climatic tolerance (various A-, B-, and C-climates). Some species [*Najas flexis* (Willd.) Rostk. Et W.LE.Schmidt, *N. gracillima* (A.Braun ex Engelm.) Magnus., *N. graminea* Delile, *N. guadalupensis* (Spreng.) Magnus, *N. marina* L., and *N. tenuissima* (A.Braun ex Magnus) Magnus] thrive well under snow climates with hot to cool short summers (mainly Dfa, Dfb, Dfc; extending into Dsc, Dwa, Dwb, Dwc) (Figure 9). The fourth and last group in Clade A comprises *Vallisneria*, *Nechamandra*, and *Hydrilla*. *Hydrilla verticillata* (L.f.) Royle, the single species of this genus, is native to Eurasia, Australasia, and tropical Africa. Its wide climatic amplitude includes fully tropical (Af, Am, Aw), arid desert and steppe (mainly BSh, BSk; extending into BWk), and temperate climates with hot to warm summers (mainly Cfa, Cfb, Cwa). In Central and East Asia, this species thrives under snow climates with hot to cool short summers (mainly Dfb). *Nechamandra* is restricted to Southeast Asia and tropical (Aw) and temperate climates with hot summers (Cfa, Cwa), occasionally extending into hot desert climates (BWh). The 16 *Vallisneria* species are widely distributed on most continents but rare in South America (only found in Colombia). Some species’ climatic preferences (*Vallisneria annua* S.W.L.Jacobs et K.A.Frank, *V. caulescense* F.M.Bailey et F.Muell., *V. erecta* S.W.L.Jacobs, *V. rubra*, (Rendle) Les et S.W.L.Jacobs, *V. triptera* S.W.L.Jacobs et K.A.Frank) include tropical (mainly Aw; extending into Af, Am) and arid desert and steppe climates (mainly BSh, BWh; extending into BSk, BWk). Other members of this genus [*V. denseserrulata* (Makino) Makino, *V. natans* (Lour.) H.Hara, and *V. spinulosa* S.Z.Yan] thrive predominantly in temperate and snow climates with hot to warm summers (mainly Cfa, Cfb, Dfa, Dfb). *Vallisneria americana* Michx., *V. nana* R.Br., and *V. spiralis* L. are more generalistic in their climatic preference (occurring under various A-, B-, C, and D-climates) (Figure 9).

Based on the climatic preference of Hydrocharitaceae, it becomes apparent that most species prefer tropical to warm temperate climates (Figure 9). The outlier of this pattern is the monotypic *Stratiotes*, which is restricted to warm temperate and snow climates of western Eurasia.

## 3. Discussion

### 3.1. The Paleophytogeographic History of Stratiotes

**Figure 10:**
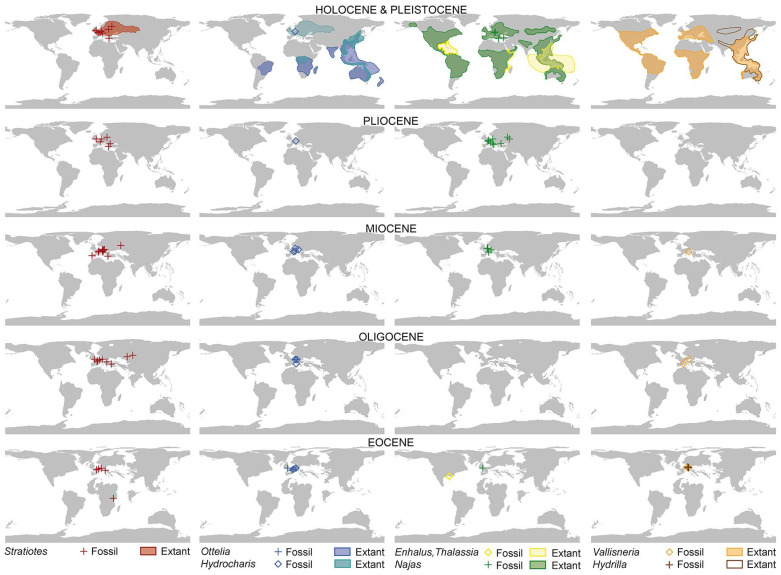
Distribution of Hydrocharitaceae in time and space. Extant distribution of genera is based on a cleaned georeferenced GBIF dataset (Supplementary Material S1, Table S5) and plotted on the Pleistocene map. Fossil records and ages of paleofloras are based on [22,38,39,40,41,42,43,44,45,46,47,48,49,50,51,52,53,54,55,56,57,58,59,60,61,62,63,64,65,66,67,68,69,70,71,72,73,74,75,76,77,78,79,80,81,82,83,84,85,86,87,88,89,90,91,92,93,94,95,96,97,98,99,100,101,102,103,104,105,106,107,108,109,110,111,112,113,114,115,116,117,118,119,120,121,122,123,124,125,126,127,128,129,130,131,132,133,134,135,136,137,138,139,140,141,142,143,144,145,146,147,148,149,150,151,152,153,154,155] (for details see Supplementary Material S1, Tables S2 and S6). Palaeomaps modified after [156], epoch maps are based on GeTech palaeo-plate rotational models for the middle of each epoch’s first stage. For simplicity, all fossil occurrences of a genus within an epoch, regardless of their particular stratigraphic range, were plotted on the epoch’s middle first-stage map.

**Figure 1:**
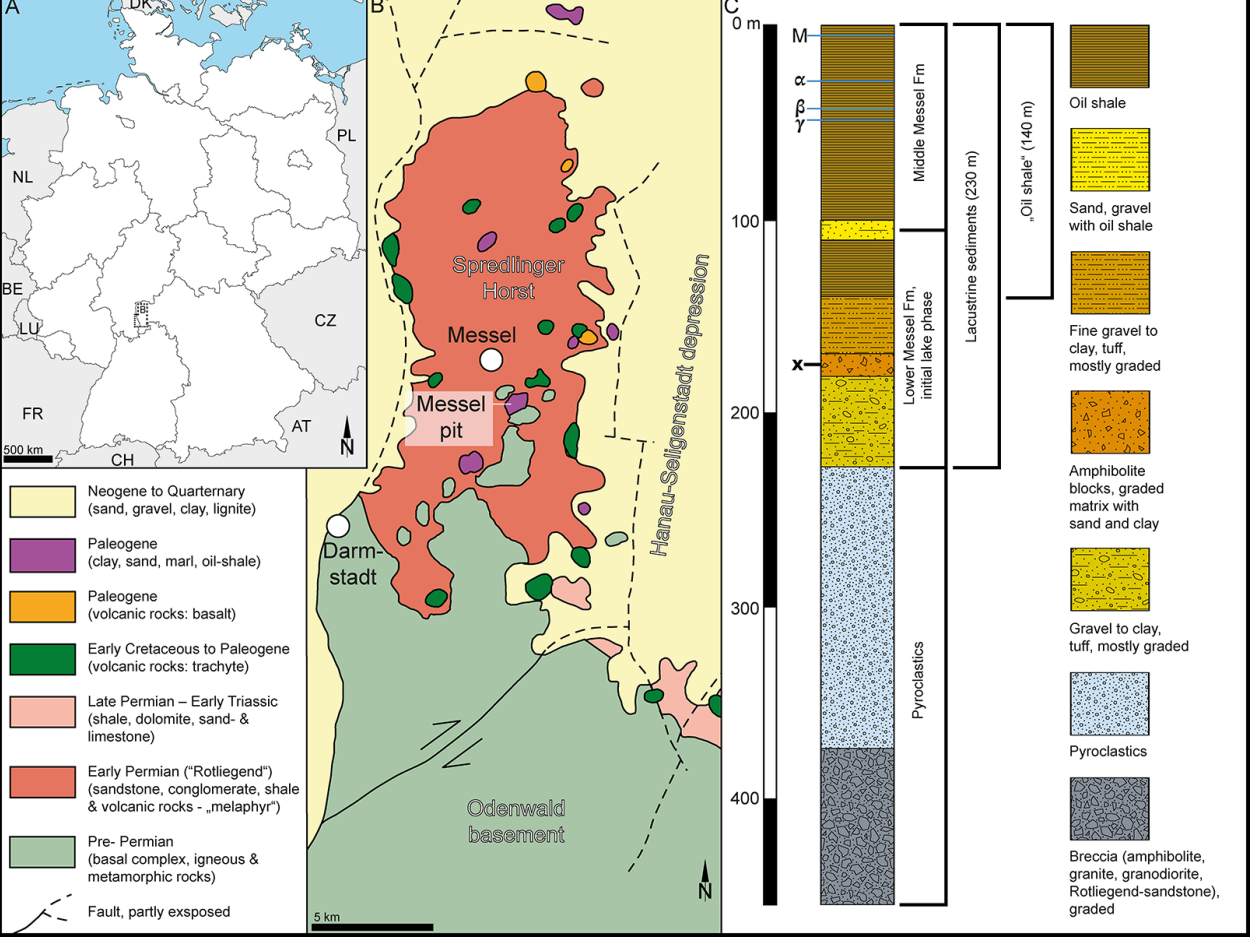
Geography and geology of the sample site at Messel, Germany, Europe. (**A**). Schematic map showing the geographic position of Messel pit, Germany, Europe. (**B**). Simplified geological map of the area surrounding the Messel pit (modified after [19]). (**C**). Compiled stratigraphic profile (modified after [20]). Stratigraphic level of sample comprising the fossil *Stratiotes* is marked with an x.

The oldest *Stratiotes* record reported so far is from the earliest Eocene of the UK (~56 Ma; Supplementary Material S1, Tables S2 and S6) (Figure 10). The timing and provenance of the known fossil record suggest a West European origin for *Stratiotes*, followed by an eastward dispersal across Europe into northwestern Asia during the Eocene. The genus had a European west–east transcontinental distribution by the latest Eocene and extended into European Russia and Western Siberia from the Oligocene onwards. Apparently, the taxon then thrived in various parts of Europe and Western Siberia until the Pliocene/Pleistocene (Supplementary Material S1, Table S2). Recent phylogenetic studies ([9] Figure 1; [10] Figure 1) place *Stratiotes* as the earliest diverging lineage and sister to the remaining Hydrocharitaceae. If the family originated in South/Southeast Asia as inferred by Chen et al. [9], it would have needed to radiate quickly into Europe where it thrived for the next 50+ Ma, while going extinct in its area of origin. The early Eocene fossil records and phylogenetic position of *Stratiotes* would point towards Europe as the focal point of origin for the genus/family. This is consistent with our new finds, one of which represents an Eocene lineage of *Stratiotes* in sub-Saharan Africa, an area that today hosts the second diverging lineage of the family, *Lagarosiphon*. The fossil record of *Stratiotes* has until now been restricted to Europe and Western Siberia (Figure 10) and composed mostly of entire seeds or single valves of germinated seeds. The lack of *Stratiotes* in the fossil plant record of Africa is not surprising. *Stratiotes* fossils have until now been confined to Cenozoic fruit/seed records (Supplementary Material S1, Table S2) and such assemblages are rare in Africa (summarized in [28], Table 5.1 and Figure 5.1). Four fruit/seed floras from the Paleocene to Eocene have been reported in Egypt, along with a single Eocene fruit/seed flora in Sierra Leone [28]. None of the reports on these five floras documents the presence of *Stratiotes*. The notable absence of dispersed *Stratiotes* pollen in the pre-Pleistocene global paleopalynological records to date can be attributed to the entomophilous nature of the plants [13]. *Stratiotes* produce only a small amount of pollen (even none at all) per fertile stamen [15], and extant *Stratiotes* also reproduce vegetatively [29]. Also, because of the structure and function of the pollen wall (see before), the grains disintegrate easily. Furthermore, the majority of paleopalynological studies rely only on LM, which is not sufficient to determine the generic affinity of this pollen type and can easily lead to misidentification as echinate and the much more common Arecaceae pollen. Eocene palynofloras from Europe, for example, are known for their rich and diverse palm pollen components (e.g., [30]).

**Figure 2:**
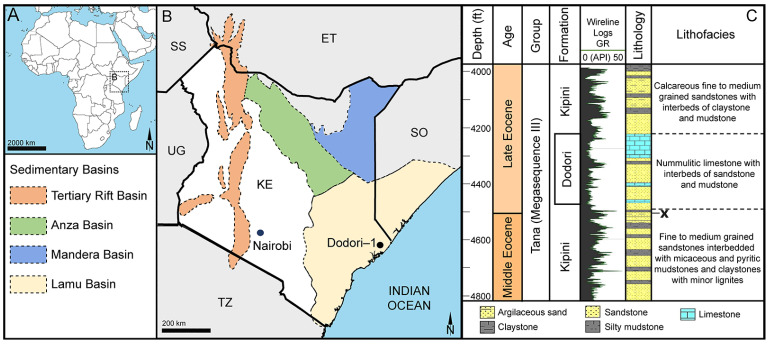
Geography and geology of the sample site at Dodori, Kenya, Africa. (**A,B**). Schematic maps showing the geographic position of the Dodori-1 well and major geological formations. (**C**). Compiled stratigraphic profile (modified after [21]). Stratigraphic level of sample comprising the fossil *Stratiotes* is marked with an x.

**Figure 3:**
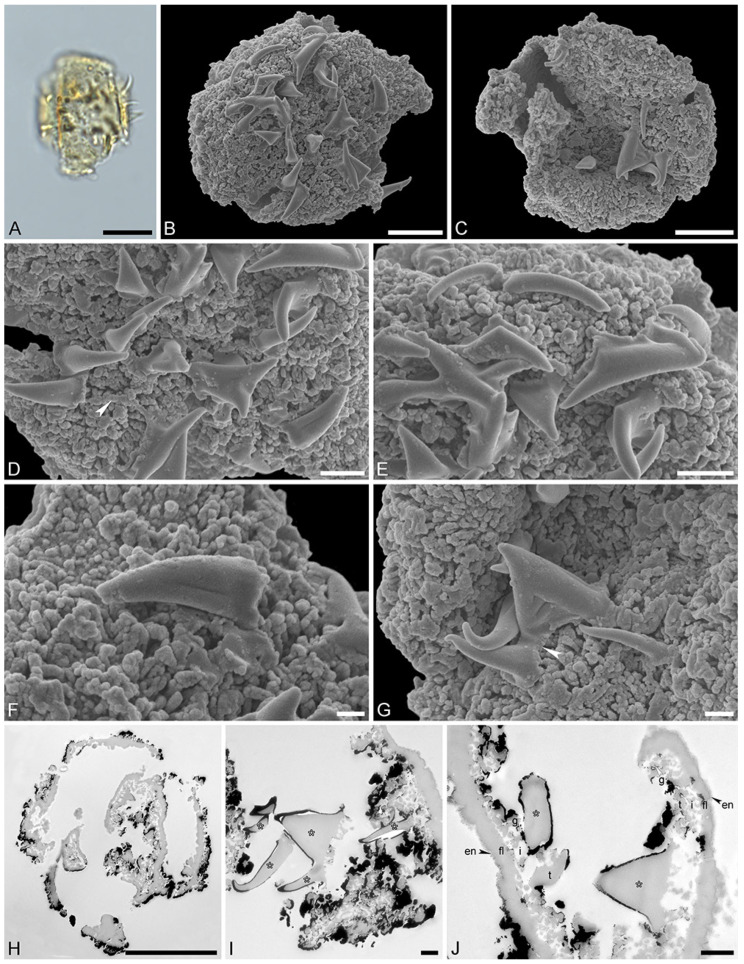
Fossil *Stratiotes* pollen from Messel, early Eocene, Germany, Europe. LM (**A**), SEM (**B**–**G**), and TEM (**H**–**J**) micrographs. (**D**,**E**) Close-up showing reticulum, columellae supporting the eroded murus (white arrowhead). (**F**,**G**) Close-up showing echini, murus between echini (white arrowhead). (**H**) Cross-section of pollen grain, unstained, TEM. (**I**,**J**) Detail showing cross-sections of pollen wall with thin compact-continuous endexine (en, black arrowheads), thick, compact-continuous footlayer (fl), alveolate-granular infratectum (i), semitectum (t), supratectal elements (echini, asterisk), and smaller sculpture elements such as granules (g, white arrowhead); note the electron-dense gold layer, unstained, TEM. Scale bars—10 μm (**A**–**C**,**H**), 2 μm (**D**,**E**), 1 µm (**F**,**G**,**I**,**J**).

**Figure 4:**
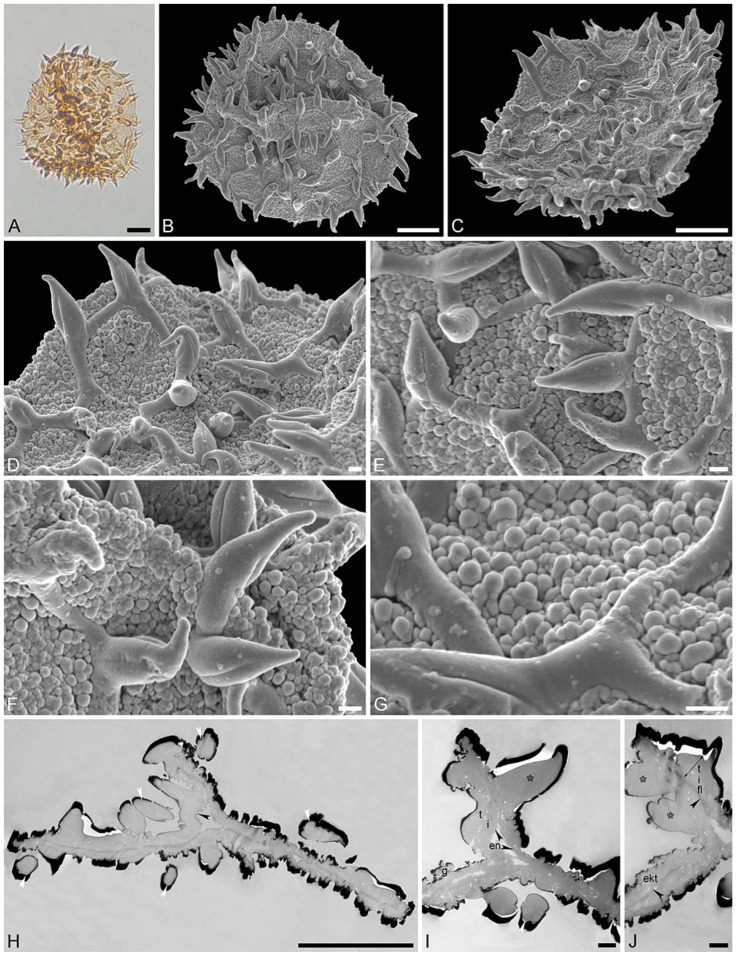
Fossil *Stratiotes* pollen from Dodori, earliest late Eocene, southeast Kenya, Africa. LM (**A**), SEM (**B**–**G**), and TEM (**H**–**J**) micrographs. (**D**) Close-up showing reticulum. (**E**) Close-up showing reticulum and muri of varying length. (**F**) Close-up showing twisted and furrowed echinus. (**G**) Close-up showing gemmate sculpture. (**H**) Cross-section of pollen grain, endexine pinpointed with black arrowhead, cross-sections through echini pinpointed by white arrowheads, potassium permanganate, TEM. (**I**) Detail showing cross-section of pollen wall with continuous-compact endexine (en, black arrowheads), granular infratectum (i), semitectum (t), and supratectal elements (echini, asterisk), cross-section through echini pinpointed by white arrowhead, unstained, TEM. (**J**) Detail showing cross-section of pollen wall with endexine (black arrowheads) and ektexine (ekt) with semitectum (t) alveolate-infratectum (i), thin, continuous-compact footlayer (fl), and supratectal elements (echini, asterisk), potassium permanganate, TEM. Scale bars—10 μm (**A**–**C**,**H**), 1 μm (**D**–**G**,**I**,**J**).

**Figure 5:**
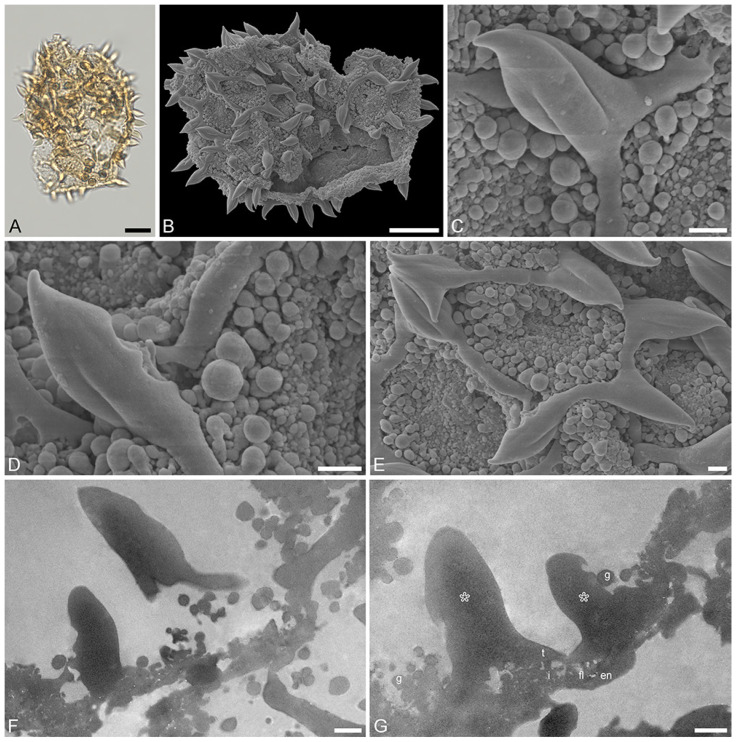
Fossil *Stratiotes* pollen from Dodori, earliest late Eocene, southeast Kenya, Africa. LM (**A**), SEM (**B**–**E**), and TEM (**F**,**G**) micrographs. (**C**,**D**) Close-up showing twisted and furrowed echinus. (**E**) Close-up showing one brochus of reticulum and muri of varying length. (**F**,**G**) Cross-section of pollen wall with compact, more or less continuous endexine (en), thin, compact-continuous footlayer (fl) alveolate-granular infratectum (i), semitectum (t), supratectal elements (echini, asterisk), and smaller sculpture elements such as granules (g), unstained, TEM. Scale bars—10 μm (**A**–**C**), 1 μm (**D**–**G**).

**Figure 6:**
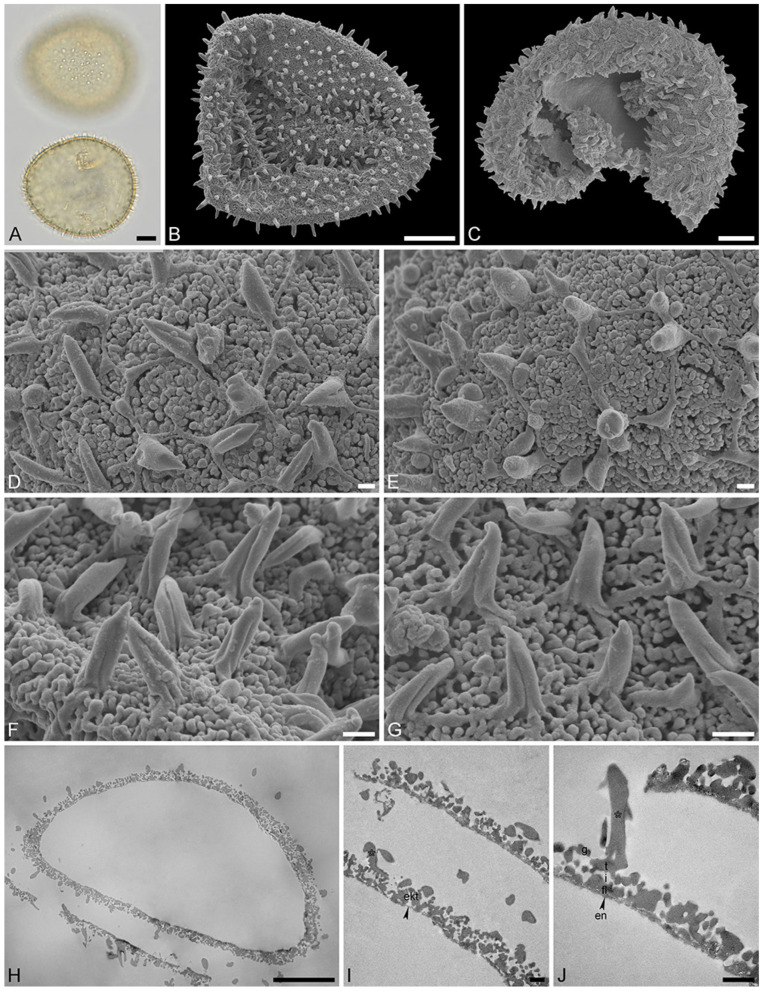
Recent *Stratiotes aloides* L. pollen from herbaria specimens (**A**,**B**,**F**,**G**) from WU0152795; (**C**–**E**,**H**–**J**) from WU0152793). (**A**) LM micrographs in upper focus plane and optical cross-section. (**B**,**C**) SEM overviews. (**D**–**G**) Close-ups of pollen surface showing the reticulum, sculpture elements inside lumina, and twisted, three-furrowed echini atop the muri. (**H**) Cross-section of a pollen grain in TEM, unstained. (**I**,**J**) Detail showing cross-section of pollen walls with thin compact-continuous endexine (en, black arrowhead), compact-discontinuous footlayer (fl), alveolate-granular infratectum (i), semitectum (t), supratectal elements (echini, asterisk), and smaller sculpture elements such as granules (g), unstained, TEM. Scale bars—10 μm (**A**–**C**,**H**), 1 μm (**D**–**G**,**I**,**J**).

**Figure 7:**
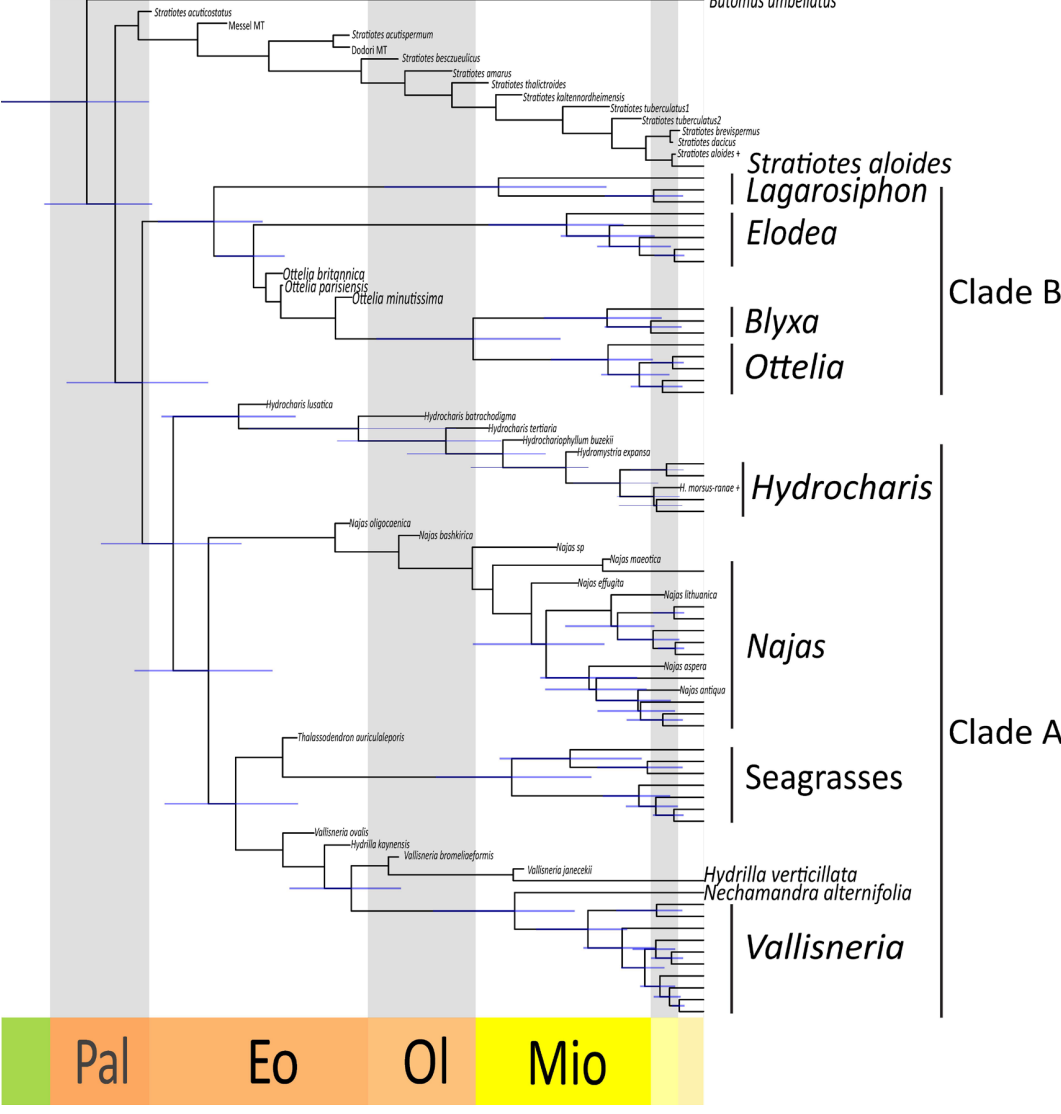
Dated phylogeny of the Hydrocharitaceae. Maximum Clade Credibility tree obtained after removing the fossil taxa from the posterior trees. In addition, 95% Highest Posterior Density intervals are shown at nodes (blue lines). Late Cretaceous (green), Paleocene (Pal), Eocence (Eo), Oligocene (Ol), Miocene (Mio), Pliocene (light yellow), Pleistocene (beige).

**Figure 8:**
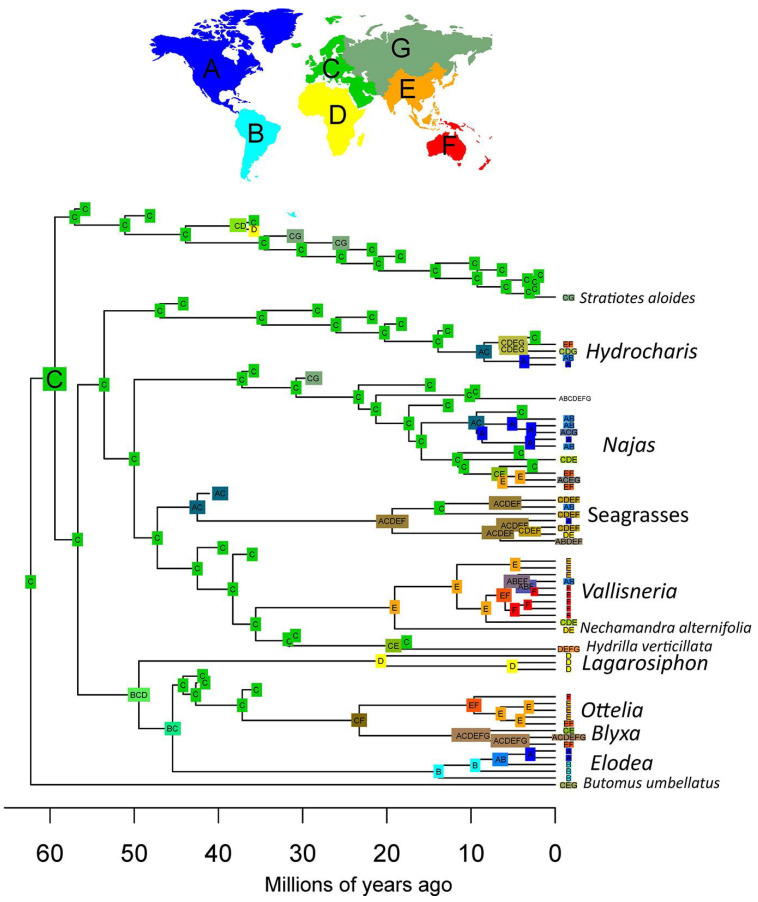
Biogeographical reconstruction of the Hydrocharitaceae. Reconstruction produced using the Dispersal–Extinction–Cladogenesis (DEC) model, scoring absences in fossil tips as uncertain. The reconstruction shows a European origin of most extant clades.

**Figure 9:**
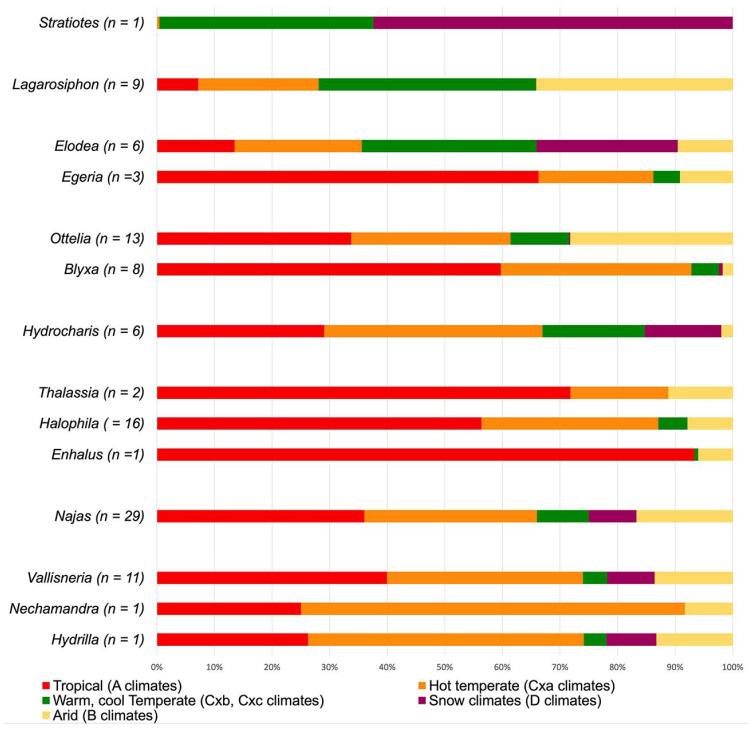
Main climatic profiles of extant Hydrocharitaceae. Climatic parameters are defined in Supplementary Material S2. Graphs/bar charts including all Köppen climate types are available in Supplementary Material S2. *n* = number of investigated species.

Based on the current fossil record (Figure 10), including our new finds, the sole surviving *Stratiotes* species is a relict of the first phase of Hydrocharitaceae radiation involving intercontinental dispersal towards the Americas, Asia, and Africa from Europe. The inferred late Cretaceous and Paleocene East Asian (Oriental) origin for Hydrocharitaceae/*Stratiotes* of Chen et al. [9] is not justified by the “*fact*” that “*the genetic diversity centre of the family is in topical Asia*” [9] (p. 7), but biased by the according modern-day underrepresentation of the earliest diverging non-Asian lineages. In addition, Chen et al. [9] relied on two (outdated) methods for biogeographic analysis that ignored the phylogenetic distances along their tree. Most of the tips are scored as combined areas (coded as polymorphisms; [9] Figure 2), including the “Oriental area” I, hence, the “Oriental” origin of the family, although only four species scattered across the tree and with different biogeographic affinities (South American, Australian, and sub-Saharan African sisters) have been coded as exclusively E. In the case of *Stratiotes (aloides)*, the “Oriental” area was erroneously included: the species stretches from Europe into Siberia (cf. [11] Figure 8), i.e., covers the “West…” and “East Palearctic” area. Thus, their analysis set-up was strongly biased towards an “Oriental” origin. Repeated intercontinental dispersal is likely in the case of aquatic plants such as the Hydrocharitaceae that are dispersed mainly by birds [31], and in the case of *Stratiotes*, through interconnected bodies of water via turions that serve as vegetative dispersal units [18]. In addition, the extant distribution of many plant lineages only reflects the latest Cenozoic dispersal and East Asian refugia (e.g., [9,32]).

Based on the available fossil record (Figure 10), niche preferences, and paleogeography, we suggest that *Stratiotes* originated in the British Islands region in the late Paleocene/earliest Eocene and then dispersed eastwards across Europe and southwards into Africa throughout the Eocene. With tropical and subtropical climate equivalents and corresponding vegetation extending from the equator to mid and high latitudes in Europe, temperature would not have been a barrier to a southward dispersal of *Stratiotes* from Europe into Africa. Such a ‘northern route’ from Eurasia into Africa during the Eocene was recently proposed for the Picrodendraceae, believed to have dispersed into Africa from the Americas via Europe [33], and the Loranthaceae, believed to have entered Africa from Asia around the same time [34,35]. Paleocene to Eocene palynological studies from northern Africa also indicate the presence of typically Northern Hemisphere taxa, including a group of triporate forms likely related to Betulaceae, Hamamelidaceae, and Juglandaceae, as well as fruits of Fagaceae from Egypt (e.g., [28]). Eocene high sea levels resulted in the widespread availability of lacustrine, lagoonal, and estuarine environments between Europe and Africa during the latest Paleocene until at least the middle-to-late Eocene [36]. These environments would have facilitated southward migration by *Stratiotes*, even though North Africa was covered by shallow seas with some scattered islands at that time, thus requiring some long-distance dispersal. Interestingly, the vertebrate paleontological record also documents several groups that migrated into Africa from Eurasia (primarily Europe) during the Paleogene, and as Africa was depauperate in vertebrate diversity following the breakup of Gondwana in the late Cretaceous, northern immigrants became established, evolving into several characteristic endemic clades [37]. Why and how *Stratiotes* was extirpated in Africa can only be hypothesized. On the basis of its co-occurrence with pollen of the mangrove palm, *Nypa* (F. Grímsson, pers. obs.), a relatively expanded shallow coastal zone and large interior basins were present in northern tropical Africa during the latest Paleocene through the middle Eocene, and a major global decline in mangrove communities in the later Eocene, and at the Eocene–Oligocene transition [36], it seems reasonable to hypothesize the loss of *Stratiotes* from Africa at the same time. Based on the fossil record, the phylogeny, and the current distribution of modern genera, it is possible that African *Stratiotes* evolved into *Lagarosiphon* (?*Appertiella*), the first diverging genus of sister clade B. Both *Lagarosiphon* and *Appertiella* are currently endemic to Africa and/or Madagascar. This scenario would imply that the *Stratiotes* pollen is primitive within the family.

### 3.2. Ecological and Climatic Preferences of Extant and Fossil Stratiotes

Today, *Stratiotes* grows in freshwater lakes, sheltered bays, backwaters of rivers, ponds, ditches, and other water bodies that are 2–5 m deep with a stable water level, and is never found in temporary nor greatly fluctuating waters, or in shallow or swiftly flowing water bodies. The plant prefers nutrient-rich muddy substrates and usually occurs in calcareous mesotrophic water [11]. During the latest Paleocene to Eocene, at the time when *Stratiotes* originated and diverged, all of Europe (excluding the Scandinavian craton and Western Russia) was composed of numerous archipelagoes extending from the British Islands to what today is Asia Minor and Central Asia (e.g., [157,158] and references therein). Numerous embayments and brackish and freshwater lakes occurred across Europe during that time (e.g., [159]), as was true of tropical Africa as well [36], providing havens for various aquatic plants and aquatic plant communities (e.g., [160]). In Eocene Germany, in the region of Messel, several paleolakes of volcanic origin (maar lakes) are known [161,162]. Regardless of the origin of the lake, being volcanic like the Messel maar or otherwise, they sustained complex ecosystems ranging from deep to shallow waters and from coastline to adjacent terrestrial surroundings (e.g., [163]), providing also ideal aquatic habitats for *Stratiotes*.

Interestingly, some of the Eocene and Oligocene *Stratiotes* fossil seeds originate from sediments that accumulated in brackish or shallow marine environments (e.g., [11,160]). The sedimentary context of the fossil African *Stratiotes*, the Dodori MT from the earliest late Eocene, fits within this scenario. The fossil pollen originated from the Kipini Fm (Figure 2) and was sampled from a sedimentary sequence dominated by sandstones interbedded with various mudstones, claystones, and lignite layers interpreted to have accumulated within a delta-front environment [164]. Further evidence of brackish or estuarine water comes from the co-occurrence of *Nypa* (“mangrove”) palm pollen in the same sample. We can hypothesize that the African *Stratiotes* was part of the lowland wetland/aquatic vegetation in East Africa during the Eocene, growing in lagoonal or estuarine bodies close to the coastline associated with paleo-river(s) running eastwards into the ocean.

As mentioned previously, *Stratiotes’* current distribution covers Europe and parts of western Central Asia (see [11] Figure 8). In its main distribution area, Western and Central Europe, *Stratiotes* is thriving under a fully humid warm temperate climate with warm summers (Köppen–Geiger Cfb-climate; cf. [165]) (Supplementary Material S2). At its margins, in parts of Scandinavia, Eastern Europe, and Central Asia, *Stratiotes* is growing under a fully humid snow climate with warm and/or cool summers and cold winters (Dfb-, Dfc-climates). Taking into account that the European fossil record of *Stratiotes* extends approximately 56 million years back in time (Supplementary Material S1, Table S2) and that climate in Europe was (much) warmer during most of the Cenozoic than at present (e.g., [166,167]), one can conclude that the current distinctly temperate climate preference of the sole surviving species, *S. aloides*, is not representative of its generic lineage. Previous studies record between 8 and 16 different *Stratiotes* species throughout the Cenozoic of Eurasia (e.g., [11,50,81,121,137,160]). During most of the Eocene, when *Stratiotes* dispersed eastwards across Europe and was represented there by up to five different taxa (e.g., [11,137,160]), the region was in a hot and humid “paratropical” climate comparable to that of the present-day moist tropics to hot subtropics [166,167]. In Europe, this paleoclimatic regime sustained a unique thermophilic flora, the so-called Paratropical Rainforest (e.g., [160]), that disappeared from Europe at the end of the Eocene. The potential modern analogs of many Paratropical Rainforest plant taxa (e.g., [160]) are currently thriving under various equatorial climates (Af-, Am-, Aw-climates) or fully humid warm temperate climates with hot summers (essentially a subtropical Cfa-climate). This means that during its initial European and out-of-Europe radiation, *Stratiotes* was accustomed to a climate considerably warmer (and more humid) than at present. The climate in Messel, Germany, c. 48 Ma was much warmer than present and located approximately 6° farther south than today, at a paleolatitude of about 45° N (see [161] Figure 2.2). Paleoclimatic estimations based on fossil plant remains from Messel suggest a mean annual temperature ranging from 16.8 to 23.9 °C, with temperatures during summer months reaching 24.7–27.9 °C. The temperature of the coldest month is believed to have exceeded 10 °C, supporting a frost-free climate [168]. Oxygen isotope measurements based on vertebrate fossils from Messel indicate similar paleotemperatures around 18 ± 2.5 °C [169]. The climate in Kenya 38–37 Ma was undoubtedly warmer than present and located approximately 10° farther south than today, at a paleolatitude of about 8° S. A recent reassessment of sea surface temperatures (SSTs) determined from coupled clumped isotope (Δ47) and Mg/Ca measurements on the carbonate from early Eocene foraminifera in cores from off the coast of Tanzania indicate tropical SSTs were between 30 °C and 36 °C throughout the Eocene [170]. Middle Eocene clumped isotope (Δ47)-derived values (at paleolatitude ~19° S) are near the higher end of that range. Although we do not precisely know the land-based equivalent of sea surface temperatures, it was indeed hot. By comparison, the modern mean annual temperature in Lamu, Kenya, is approximately 27 °C. Global climate models produce an enhanced hydrological cycle at low latitudes (e.g., [171]), but this may not be important regarding *Stratiotes*, an aquatic plant.

### 3.3. Paleogene Origin and Dispersal of Hydrocharitaceae

Although our dated phylogeny does not retrieve considerably different ages from Chen et al. [9], our biogeographical analysis shows that inferring the ancestral age of Hydrocharitaceae based solely on the distribution of extant taxa is potentially misleading. Indeed, our results support a much more important role for Europe in the distribution of the family. Although this could be partially driven by the excellent European record for the family, which in turn could be driven by collection bias, our analysis did not retrieve major conflicting signals that could have informed the distribution of fossil taxa, whose absence in the other biogeographical areas was left as uncertain. With the exception of the seagrasses, whose distribution is probably not limited by biogeographical barriers, widespread clades such as *Najas* and *Hydrocharis* seem to have dispersed out of Europe after the mid-Miocene, while more endemic genera such as *Vallisneria, Lagarosiphon,* and *Elodea sensu lato* obtained their current distribution (or close to their current distribution) earlier than the Miocene. This difference in biogeographical history, taken together with the results of our climate signatures, opens the possibility that at least some of the genera of the Hydrocharitaceae were limited in their distribution not by biogeographical barriers, but rather by the availability of appropriate climates. The mid-Miocene transition pushed some of the genera outside of the boreotropics and into the tropical climates of Southeast Asia, Africa, and the Americas, while eradicating them from Europe. On the other hand, the more cold-tolerant forms of *Stratiotes* persisted at high latitudes, leading to the current distribution of *Stratiotes aloides.*

## 4. Material and Methods

### 4.1. Origin of Samples

#### 4.1.1. Messel, Germany, Europe

The fossil *Stratiotes* pollen from Messel, Germany (Figure 1), was extracted from a clayey siltstone sample, originally positioned 176 m below the surface (sample nr. 54 in [172]), from core Messel 2001, Messel, Germany. The Messel 2001 core was drilled in 2001 during drilling to a depth of 433 m in the ± central part of the Messel pit to confirm the assumed volcanic origin of the Messel paleolake [20].

#### 4.1.2. Dodori, Kenya, Africa

The fossil *Stratiotes* pollen from Kenya, Africa (Figure 2), was recovered from a silty mudstone sample, originally positioned 4503 ft (1373 m) below the surface, from core Dodori-1, Lamu Basin, Kenya, Africa. The Dodori-1 core is named after a settlement in Kenya’s Lamu County that is situated 13 miles (21 km) inland from the coast. The Dodori-1 core was produced in 1964 during drilling to a depth of c. 14,140 feet (4340 m) in order to investigate potential hydrocarbon reservoirs in Upper Cretaceous to Cenozoic sedimentary rocks at the coast of the ancient Lamu Embayment, southeast Kenya [164].

### 4.2. Preparation and Study of Samples

The sedimentary rock samples were processed, and fossil pollen was extracted according to the method explained in Grímsson et al. [173]. Pollen from extant *Stratiotes aloides* was sampled from herbarium specimens WU0152793 [174], WU0152794 [174], and WU0152795 [174], housed in the University of Vienna Herbarium (WU; Supplementary Material S1, Table S3). The fossil and extant pollen were investigated both by light microscopy (LM) and SEM using the single-grain method as described by Zetter [175] and Halbritter et al. [176] (pp. 121–123), as well as with transmission electron microscopy (TEM) following Ulrich and Grímsson [177]. The SEM stubs and TEM sections with the fossil and extant pollen produced under this study are stored in the collection of the Department of Botany and Biodiversity Research, University of Vienna, Austria. The pollen terminology follows Punt et al. [178] (LM) and Halbritter et al. [176] (LM, SEM, and TEM). For practical reasons, the fossil pollen types are classified as morphotypes (MTs) named after the place/well where they were found.

During pollen preparation for LM, SEM, and TEM, it became clear that fossil and extant *Stratiotes* pollen grains are incredibly fragile and break and disintegrate easily. Sculpture elements (echini and muri) also fall off easily. This instability is due to the open structure, loosely attached segments comprising the infratectum, and the discontinuity of the footlayer and tectum. The pollen of *Stratiotes* is also inaperturate, and the pollen wall can rupture anywhere. Already during acetolysis, the pollen grains started to break and it can be assumed that this is part of the reason for the absence of *Stratiotes* pollen in the geological record.

### 4.3. Geographic and Geological Background

#### 4.3.1. Messel, Germany, Europe

The Messel pit (Figure 1A,B) is world-famous for the numerous exceptional fossils that have been discovered there since 1875 [179] and include priceless (in a scientific context) plant, invertebrate, and vertebrate remains (e.g., [180,181,182]). The sediments of the Messel pit accumulated during the early and middle Eocene in a crater/maar structure caused by phreatomagmatic activity dated as c. 47.8 Ma [183], c. 48.2 Ma [184], or 48.06 Ma [185]. The crater filling has been divided into four main units based on the Messel 2001 core and previous onsite research [20,182]. At the base, at a depth of 433–228 m, are massive pyroclastics and a diatreme breccia bearing no formal name (Figure 1C). The lake sediments (rocks) overlying the pyroclastics have been divided into the Lower, Middle, and Upper Messel Formation (Fm). The Lower Messel Fm occurs at 228–94 m depth in the core and comprises breccia, sand- to claystone, as well as lapilli tuffs (Figure 1C). The Middle Messel Fm occurs at 94–0 m depth in the core and consists of finely laminated dark olive-grey to dark brownish-grey clay- and siltstones (oil shale) intercalated with few sandy-gravelly layers and occasional layers of siderite (Figure 1C). The Upper Messel Fm, which has been mined away, was allegedly up to 40 m thick and composed of finely laminated blackish claystone intercalated with lignite seams and clayey sands (e.g., [20,182]). The sedimentary sample with the fossil *Stratiotes* pollen presented herein originates from the Lower Messel Fm, assigned to the Ypresian (early Eocene), and is between 48.27 and 48.05 Ma [184].

#### 4.3.2. Dodori, Kenya, Africa

The Lamu Basin covers a large area of southeastern Kenya and southwestern Somalia with an aerial extent of 132,720 km^2^ (Figure 2A) [186]. It is the largest basin in Kenya, composed of sedimentary rocks of Permian to Cenozoic age, including continental rift basin sandstones, fluvio-deltaic sandstones, marine shales, and carbonates. The Cretaceous and Cenozoic sediments have been divided into three megasequences deposited under alternating periods of transgression and regression, thus incorporating unconformities of regional and local significance [164]. Megasequence II comprises the rocks deposited between the late Jurassic and the late Paleocene, also defined as the Sabaki Group. Megasequence III constitutes the Tana Group, comprising, among others, the Kipini Fm and the Dodori limestone (Figure 2B), which are Paleogene deposits accumulated during three pulses of sea-level rise and a single regressive phase. Megasequence IV (Coastal Group) is defined by an unconformity that separates the underlying late Oligocene strata of the Tana Group from the youngest sediments filling the basin [164]. The Kipini Fm comprises several sandstones interbedded with claystones and mudstones, and the Dodori limestone is an expression of the shelf carbonate deposition that occurred in the area until the late Eocene [164]. The studied sample originates from a greyish shale of the Kipini Fm, positioned just below the Dodori limestone (at 4503 ft (1373 m) below the surface; Figure 2B), and is of earliest late Eocene (earliest Priabonian) age (~37.71 Ma; following [187]). The consistent occurrence of the large foraminifera, *Nummulites fabianii* Prever (from 4410 ft (1344 m) upwards), as well as various dinoflagellate cysts (including *Cordosphaeridium fibrospinosum* R.J.Davey & G.L.Williams) characteristic of the late Eocene, support the Priabonian age of the sedimentary matrix containing the fossil *Stratiotes* pollen. Generally, middle-to-late Eocene sediments in this region consist of interbedded nummulitic sands, poorly sorted and fine to very coarse-grained calcareous sandstones, nummulitic and micritic limestones, and dark olive greenish grey shales and grey-green silty mudstones. The sample containing the fossil *Stratiotes* pollen originates from the top of a sedimentary rock unit interpreted to have been deposited within a delta-front environment (Figure 2B) [164].

### 4.4. Dated Phylogeny

We inferred a dated phylogeny of the Hydrocharitaceae using the molecular matrix of Bernardini and Lucchese [10]. This included one nuclear locus (*ITS*) and five plastid loci (matK, rbcl, rpoB, rpoC, and trnK) for 50 species of Hydrocharitaceae, including the genera *Blyxa* (3/14 species sampled), *Elodea* (5/9, including 1 species formerly in *Agapanthe* Planch. and 2 species formerly in *Egeria*), *Enhalus* (1/1), *Halophila* (4/17), *Hydrilla* (1/1), *Hydrocharis* (4/5, including 2 species formerly in *Limnobium*), *Lagarosiphon* (3/9), *Najas* (10/39), *Nechamandra* (1/1), *Ottelia* (5/23), *Stratiotes* (1/1), *Thalassia* (1/2), and *Vallisneria* (10/16, including 1 species formerly in *Maidenia* Rendle). This dataset covers all the genera of the Hydrocharitaceae, with the exclusion of the monotypic *Appertiella* C.D.K.Cook & L.Triest, for which no molecular data are available.

After trimming one unidentified species and using *Butomus umbellatus* L. as the only outgroup, we used the resulting alignment in a tip-dating analysis under the Fossilized Birth-Death prior as employed in the software MrBayes v. 3.2.7 [188] as implemented in the CIPRES science gateway [189]. Fossil taxa of Hydrocharitaceae were added and constrained to their more likely position (see Supplementary Material S1, Table S4). The prior on the age of the root was set between 113 Ma and 56 Ma, corresponding to the oldest unequivocal fossil evidence of the monocots [82] and the oldest age of the older fossil, respectively. We employed two unlinked clock models, one for the plastid markers and one for the ITS, using an uncorrelated log-normal clock. Tip ages were implemented as uniform distributions to improve accuracy [190]. The proportion of extant sampling was set to 0.37. The MCMC was run for 50,000,000 generations, sampling every 5000 generations. Convergence was assessed using the software Tracer [191], checking for ESS of more than 200. A Maximum Clade Credibility (MCC) tree was generated discarding the first 10% of the posterior trees as burn-in. The tree was then plotted using the R package MCMCtreeR.

### 4.5. Biogeographical Analysis

A biogeographical analysis was conducted using the DEC model implemented in the R package BioGeoBEARS [192] using the MCC tree from the Bayesian analysis. We conducted the analyses using the biogeographical areas used by Chen et al. [9]. Area occupancy of the different species was scored based on the distribution retrieved from the Plants of the World Online database [26]. For the area occupancy of the fossils, we used the option “useAmbiguities = TRUE” in BioGeoBEARS, to allow the areas where the fossil was not sampled from to be scored as unknown, to reflect the uncertainty over the true absence of fossil taxa across their potential distribution.

### 4.6. Climate Data Harvesting and Analysis

We used Köppen profiles (e.g., [193,194,195,196,197]) to summarize the climatic niches occupied by extant Hydrocharitaceae species/genera and to hypothesize about their climatic niche evolution (Supplementary Material S2). A Köppen profile reflects the proportional Köppen–Geiger climate (cf. [165,198]) zone coverage of modern plant species based on their gridded distribution data from GBIF.org (Supplementary Material S1, Table S5). Unfortunately, no distribution data were available on GBIF.org for the genus *Appertiella* and some species of *Blyxa*, *Ottelia*, *Hydrocharis*, *Hydromystria* G.Mey., *Halophila*, *Thalassia*, *Najas*, and *Vallisneria*. Modern species distributions were checked for outliers (e.g., neophyte distribution) using published chorological data (e.g., [26]). Additionally, multiple occurrences with identical coordinates were merged (labeled ‘unique localities’ in the diagrams (Supplementary Material S2). The revised georeferenced occurrence data were then plotted onto 1 km^2^ grid Köppen–Geiger maps (1979–2013 data; [27]) to establish Köppen profiles for all modern species/genera. The georeferenced data and the Köppen–Geiger maps with 1 km^2^ resolution were processed using the ‘Sample Raster Values’ Toolbox in Qgis v.3.16.4-Hannover. The Köppen–Geiger climates occupied by extant Hydrocharitaceae species/genera are shown as maps generated in Qgis and as frequency (proportional distribution) diagrams (Supplementary Material S2). To simplify interpretations, the Köppen profiles of Hydrocharitaceae genera are summarized into five climatic niches. Additionally, precipitation (second letter in Köppen climate types) was excluded since all members of Hydrocharitaceae are aquatic and, therefore, not susceptible to rainfall seasonality/humidity availability.

## 5. Conclusions and Future Considerations

The oldest *Stratiotes* fossils are seeds from the early Eocene of England (London Clay), and the oldest unequivocal pollen is from the early Eocene of Germany (Messel) and the late Eocene of equatorial Kenya (Dodori, Africa). The Eocene is known as the global warm house [199], meaning the oldest known *Stratiotes* fossils were preserved under tropical or paratropical conditions. Therefore, it can be assumed that the ancestral climatic signal for *Stratiotes*, and Hydrocharitaceae in general, was tropical to hot/warm temperate. We assume that the African lineages of *Stratiotes* went extinct while the European lineages diversified and adapted to the changing climate during the Oligocene and Neogene, surviving the transition from a hot/warmhouse to a coolhouse climate. The ice ages probably presented a final bottleneck in reducing the lineage’s diversity to the remaining monotypic *Stratiotes aloides*.

The fossil *Stratiotes* pollen described herein is unique, and the only reliable pre-Holocene pollen record of this genus so far. The *Stratiotes* pollen from the Messel pit is the first unequivocal fossil record representing this genus from this locality. This finding underlines the potentiality of re-investigating European paleopalynofloras using combined LM and SEM, which have until now mostly been investigated using conventional LM.

Despite the size of the African continent, it has revealed few Cretaceous to Cenozoic macro- and mesofloras [28]. This places African palynofloras on a pedestal and makes them the most important paleobotanical source for resolving paleophytogeographic distribution patterns and interpreting past vegetation and climate changes in this part of the globe. The unique fossil *Stratiotes* pollen from Kenya (Africa) presented herein again demonstrates the usefulness of the single-grain method for paleopalynology (e.g., [200,201,202]). Future work on Africa’s palynofloras must include combined LM and SEM studies. The African paleopalynoflora is a closed treasure chest, and the single-grain method is the key to opening it. Routinely applying this method when analyzing Cretaceous and Cenozoic palynofloras from this part of the globe will certainly cast new light on the origin and evolution of Africa’s forests and vegetation units.

## Data Availability

All data supporting the results are either provided within the manuscript or in the Supplementary Materials.

## Supplementary Materials

The following are available online at https://www.mdpi.com/article/10.3390/plants13071008/s1. Supplementary Material S1. This Excel file comprises Tables S1–S6 (and references therein, Table S7) referred to in the main text [11,12,22,24,25,38,39,40,41,42,43,44,45,46,47,48,49,50,51,52,53,54,55,56,57,58,59,60,61,62,63,64,65,66,67,68,69,70,71,72,73,74,75,76,77,78,79,80,81,82,83,84,85,86,87,88,89,90,91,92,93,94,95,96,97,98,99,100,101,102,103,104,105,106,107,108,110,111,112,113,114,115,116,117,118,119,120,121,122,123,124,125,126,127,128,129,130,131,132,133,134,135,136,137,138,139,140,141,142,143,144,145,146,147,148,149,150,151,152,153,154,155,160,180,203,204,205,206,207,208,209,210,211,212,213,214,215,216,217]. Supplementary Material S2. This PDF comprises distribution and climate data for Hydrocharitaceae in a phylogenetic context [9,27,165,198,218]. Supplementary Material S3. This PDF comprises additional LM, SEM, and TEM micrographs of extant *Stratiotes aloides* pollen. Supplementary Material S4. This PDF comprises Bayesian and IQTREE produced for this study.
