# Supplementary material for: Origin and Early Evolution of Hydrocharitaceae and the Ancestral Role of Stratiotes
Source: Plants (Basel). 2024 Mar 31;13(7):1008. doi: 10.3390/plants13071008 (PMC11013807; doi:10.3390/plants13071008)
Supplement: Supplementary file 1 [file plants-13-01008-s001.zip › Supplementary_material_02_new.pdf]

Supplementary material 02
Distribution and climate of Hydrocharitaceae

1. Köppen-Geiger climate symbols and defining criteria (Peel et al. 2007, Kottek et al. 2006, Rubel et al. 2017, Cui et al. 2021)

1st	2nd	3rd	Description and criteria
A			**Equatorial**/tropical—MTCM ≥ 18 °C
	f		Rainforest, fully humid—P_{dry} ≥ 60 mm
	m		Monsoonal—not *Af* & P_{dry} ≥ 100 − MAP/25
	s		Savannah with dry summer—P_{sdry} < 60 mm
	w		Savannah with dry winter—P_{wdry} < 60 mm
B			**Arid**—MAP < 10 × $P_{threshold}$
	W		Desert—MAP < 5 × $P_{threshold}$
	S		Steppe—MAP ≥ 5 × $P_{threshold}$
		h	Hot arid—MAT ≥ 18 °C
		k	Cold arid—MAT < 18 °C
C			**Warm temperate** (subtropical to temperate)—T_{hot} > 10 °C & 0 °C < MTCM < 18 °C
D			**Snow** (cold-temperate to boreal)—T_{hot} > 10 °C & MTCM ≤ 0 °C
	s		Summer dry—P_{sdry} < 40 mm & P_{sdry} < P_{wwet}/3
	w		Winter dry—P_{wdry} < P_{swet}/10
	f		Fully humid, without a dry season, i.e. not *s* or *w*
		a	Hot summer—T_{hot} ≥ 22 °C
		b	Warm summer—not *a* & T_{mon10} ≥ 4
		c	Cool/cold and short summer—not *a/b* & 1 ≤ T_{mon10} < 4
		d	Extremely continental: cold/very short or no summer & very cold winter—not *a/b* & MTCM < −38 °C
E			**Polar—**T_{hot} < 10 °C
	T		Tundra—(T_{hot} > 0)
	F		Ice, permanent frost (‘frost desert’)—(T_{hot} ≤ 0)

MAP = mean annual precipitation, MAT = mean annual temperature, T_{hot} = mean temperature of the hottest month, T_{cold} = mean temperature of the coldest month, T_{mon10} = number of months where the temperature is above 10 °C, P_{dry} = precipitation of the driest month, P_{sdry} = precipitation of the driest month in summer, P_{wdry} = precipitation of the driest month in winter, P_{swet} = precipitation of the wettest month in summer, P_{wwet} = precipitation of the wettest month in winter, $P_{threshold}$ = varies according to the following rules: if 70% of MAP occurs in winter then $P_{threshold}$ = 2 x MAT, if 70% of MAP occurs in summer then $P_{threshold}$ = 2 x MAT + 28, otherwise $P_{threshold}$ = 2 x MAT + 14. Summer (winter) is defined as the warmer (cooler) six months period of October to March and April to September.

2. Köppen-Geiger climate map colour coding

Af	Am	As	Aw	BSh	BSk	BWh	BWk	Cfa	Cfb	Cfc
Csa	Csb	Csc	Cwa	Cwb	Cwc	Dfa	Dfb	Dfc	Dfd	Dsa
Dsb	Dsc	Dsd	Dwa	Dwb	Dwc	Dwd	EF	ET		

3. Phylogenetic relationships within Hydrocharitaceae from Chen et al. (2012, fig. 1)

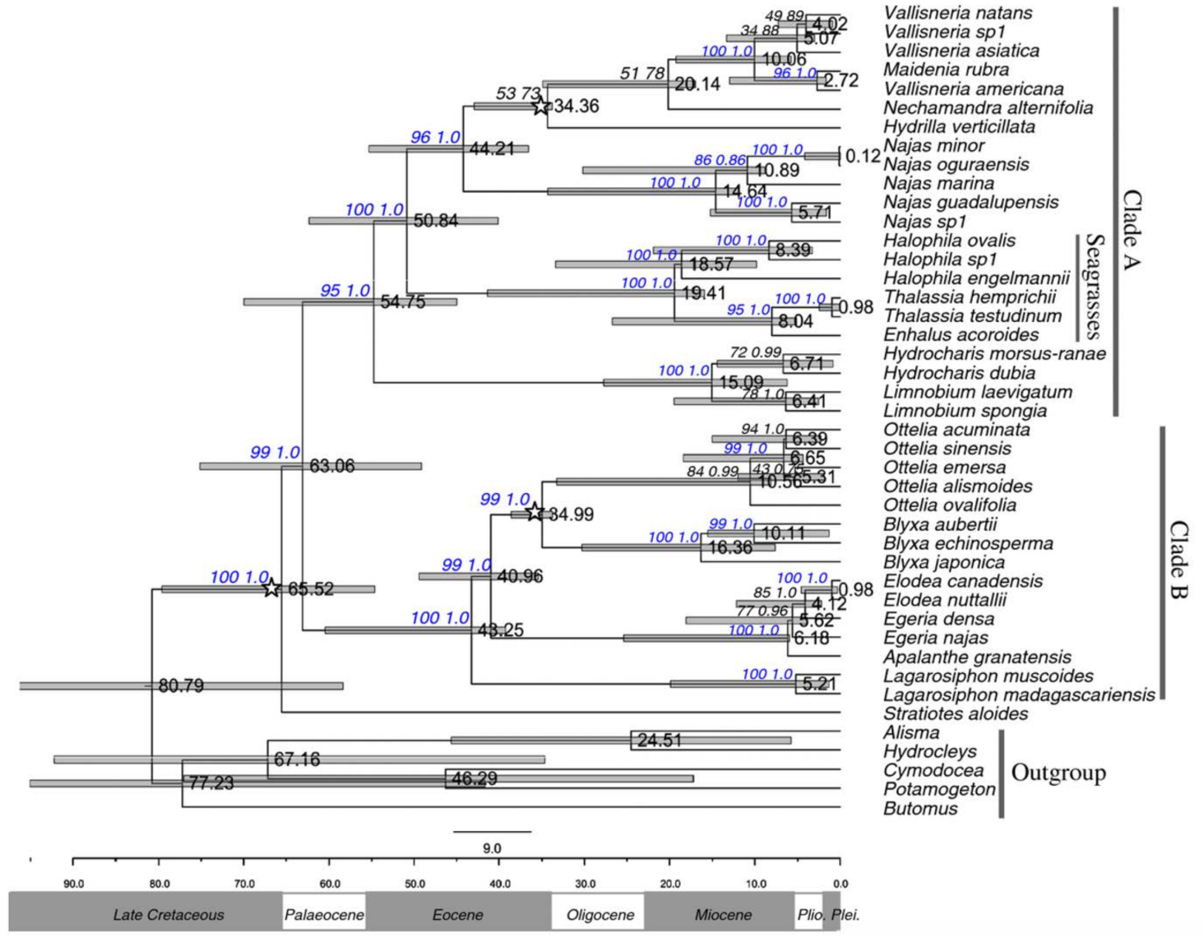

4. 1. Summarized Hydrocharitaceae genera Köppen profiles (incl. all Köppen climate types)

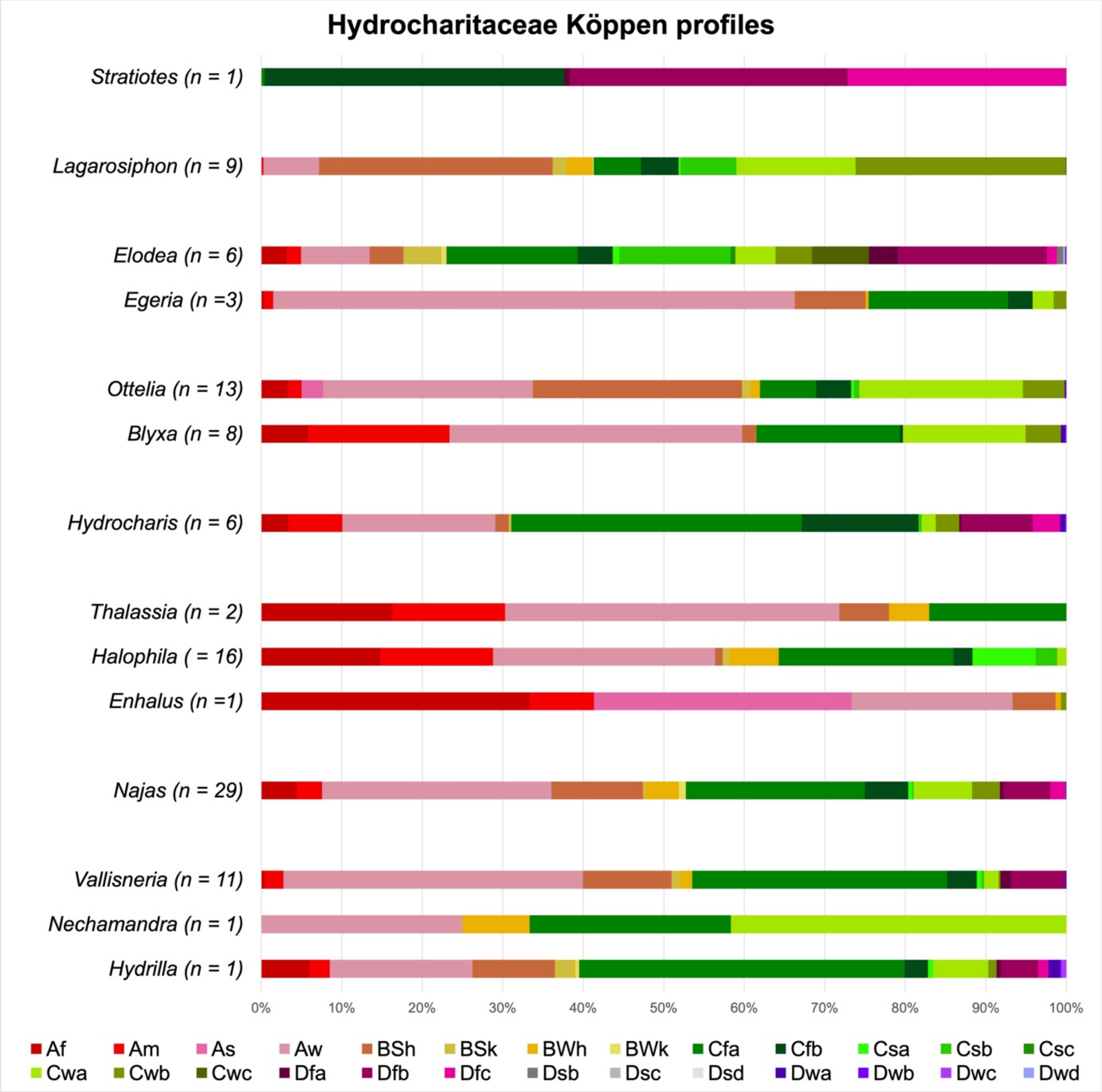

4. 2. Summarized Hydrocharitaceae genera Köppen profiles (incl. the four most commn Köppen climate types)

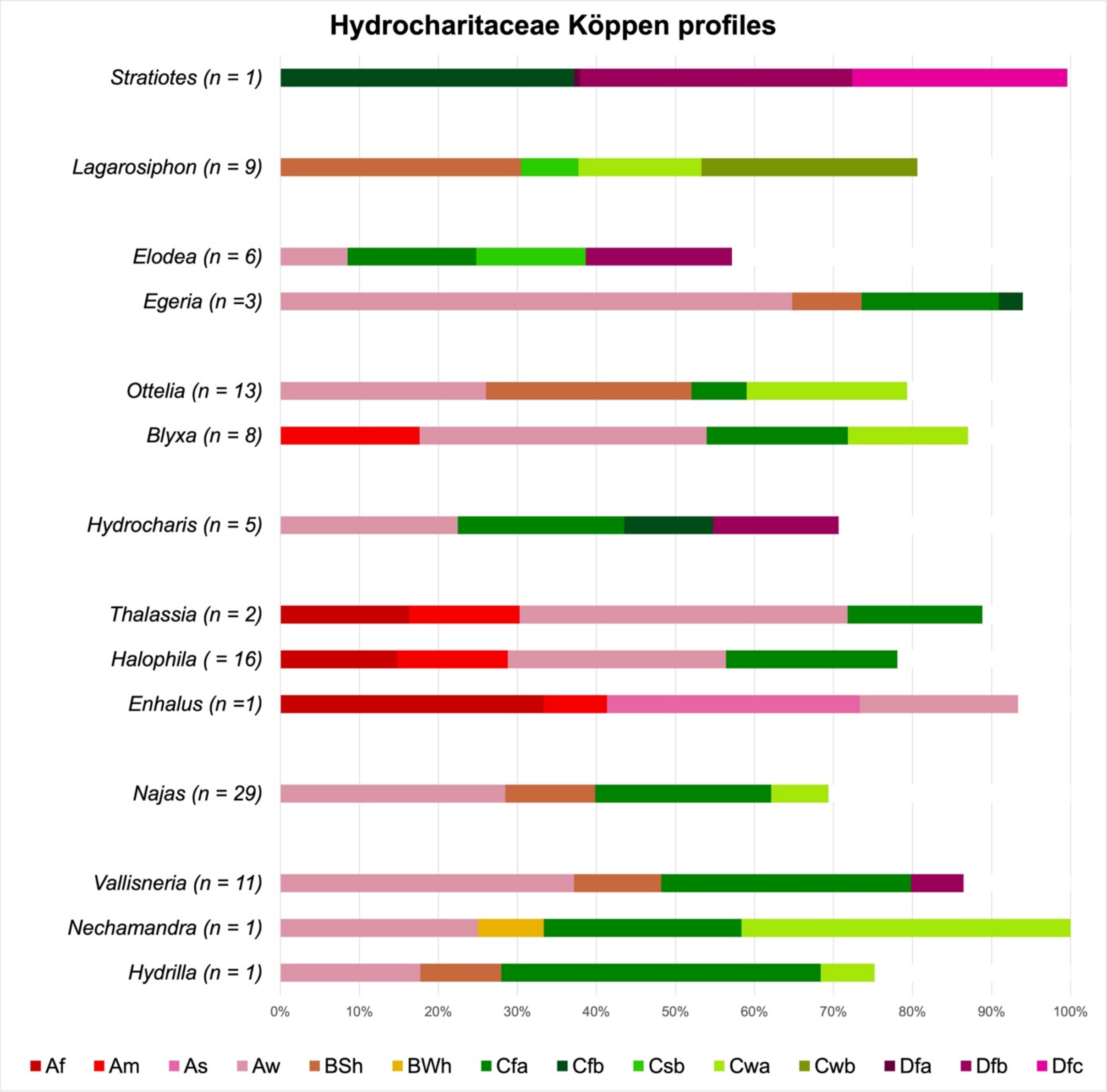

5. Hydrocharitaceae species Köppen Profiles

5.1. First diverging genus within Hydrocharitaceae cf. Chen et. al (2012)

5.1.1. Genus *Stratiotes* L., 1753.

5.1.1.1. Köppen profile, distribution and climate map– GBIF all occurrences of *Stratiotes aloides* L. 1753; all localities (n = 28431), unique localities (n = 15341), herbarium specimens excluding duplicate occurrences (n = 820), other observations (n = 14537).

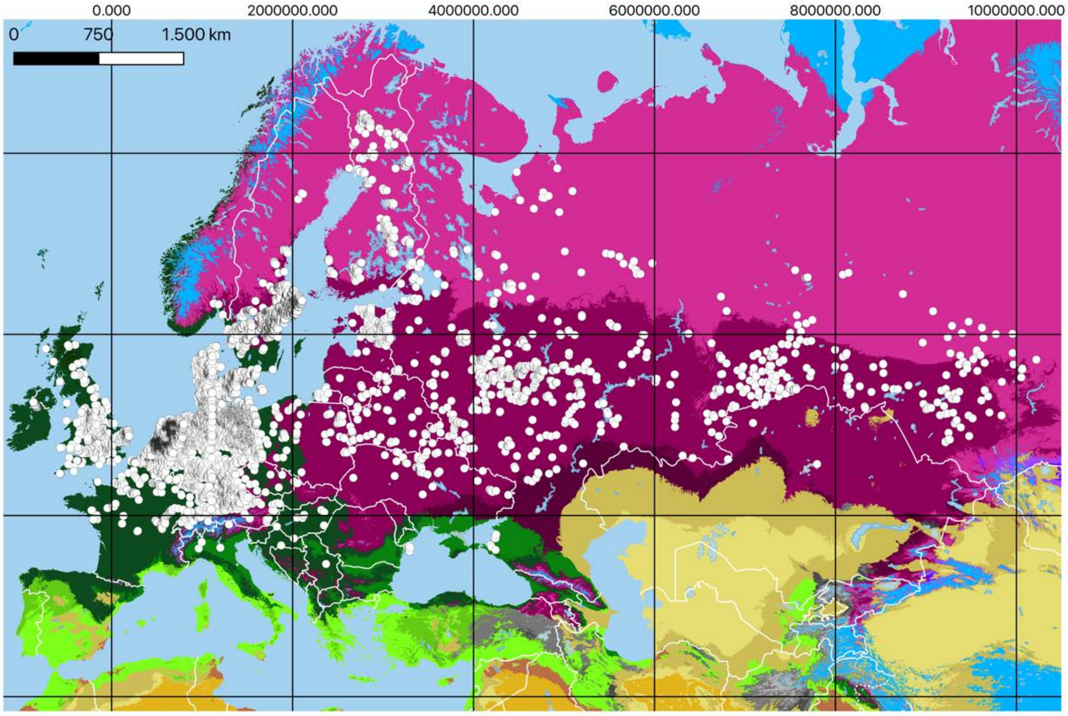

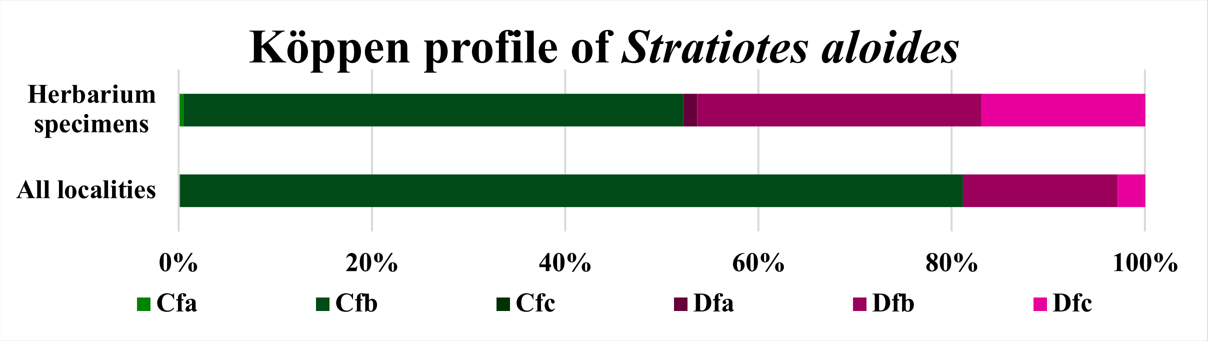

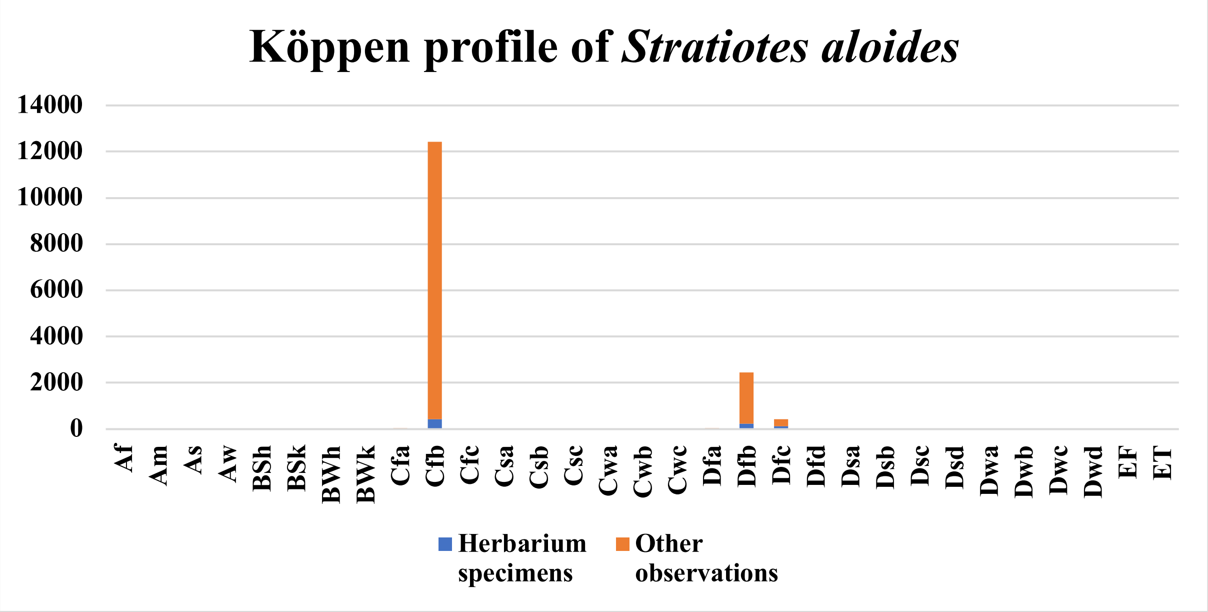

5.2 Clade B

5.2.1. First diverging genera within Clade B of Hydrocharitaceae cf. Chen et. al (2012), comprising *Lagarosiphon* Harv., 1841 and *Appertiella* C.D.K.Cook & Triest, 1982 (GBIF database did not contain data sets of *Appertiella* including geographic occurrence data).

5.2.1.1. Genus *Lagarosiphon* Harv., 1841

Distribution and Köppen profiles for *Lagarosiphon* incl. *L. cordofanus*, *L. hydrilloides*, *L. ilicifolius*, *L. madagascarensis*, *L. major*, *L. muscoides*, *L. rubellus*, *L. steudneri*, and *L. verticillifolius,* based on herbarium specimens (n = 445).

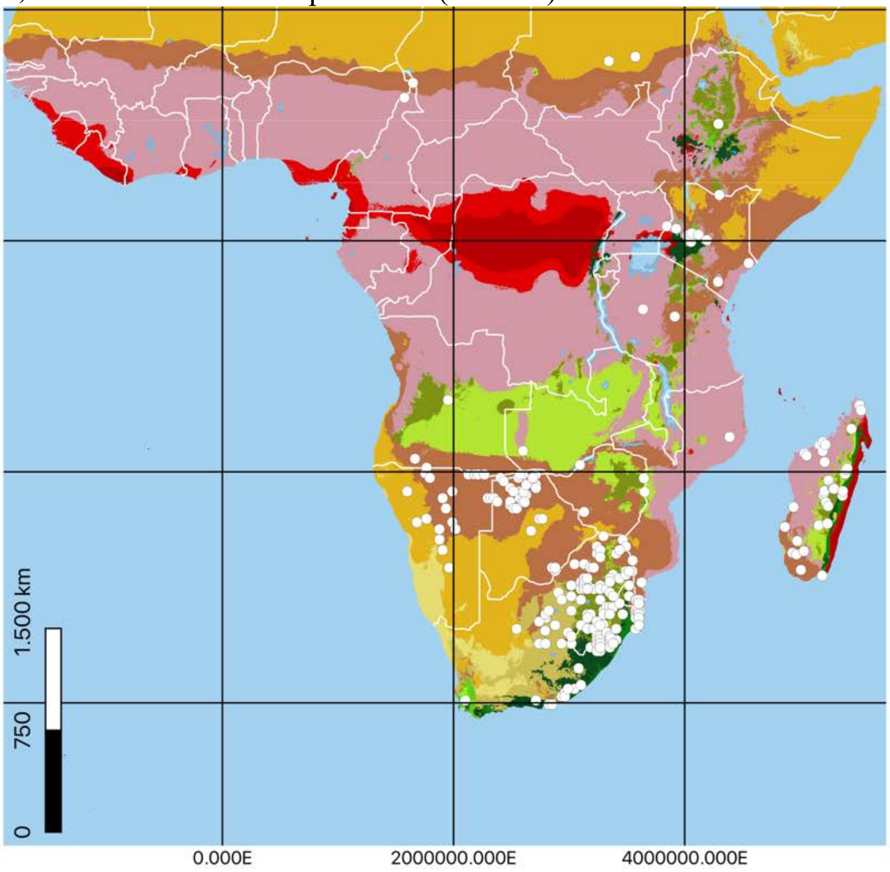

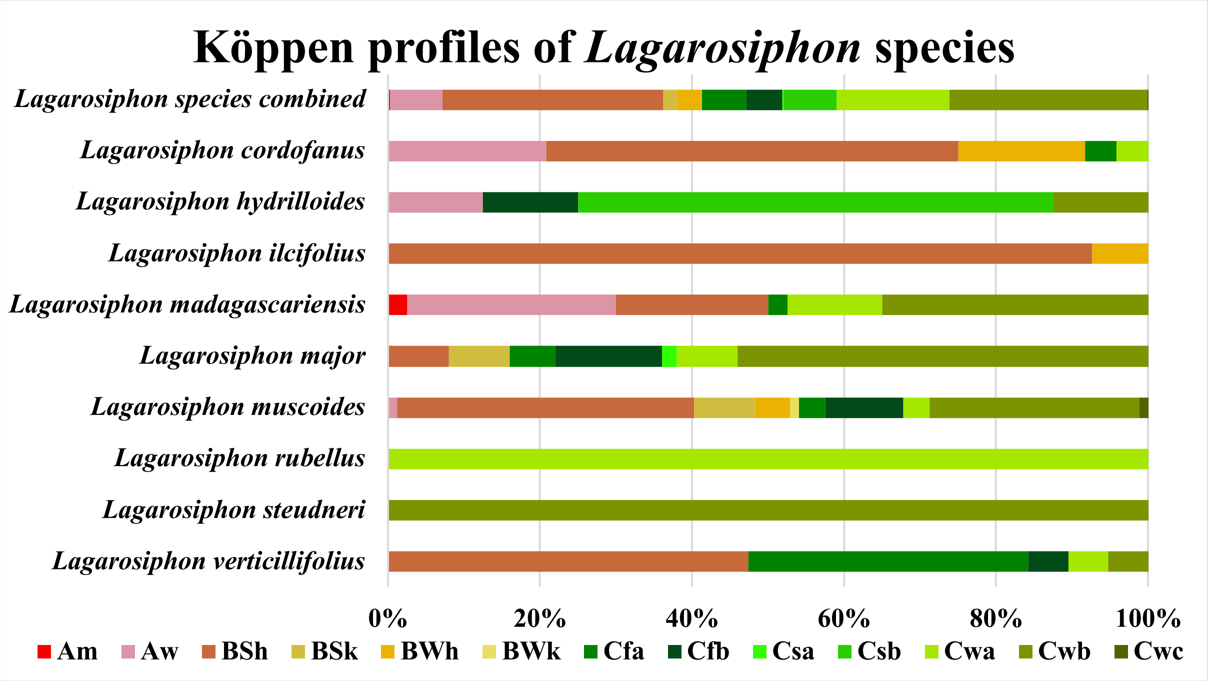

5.2.1.1.1. Köppen profile, distribution and climate map– GBIF occurrences of *Lagarosiphon cordofanus* Casp. 1858; herbarium specimens excluding duplicate occurrences (n = 24).

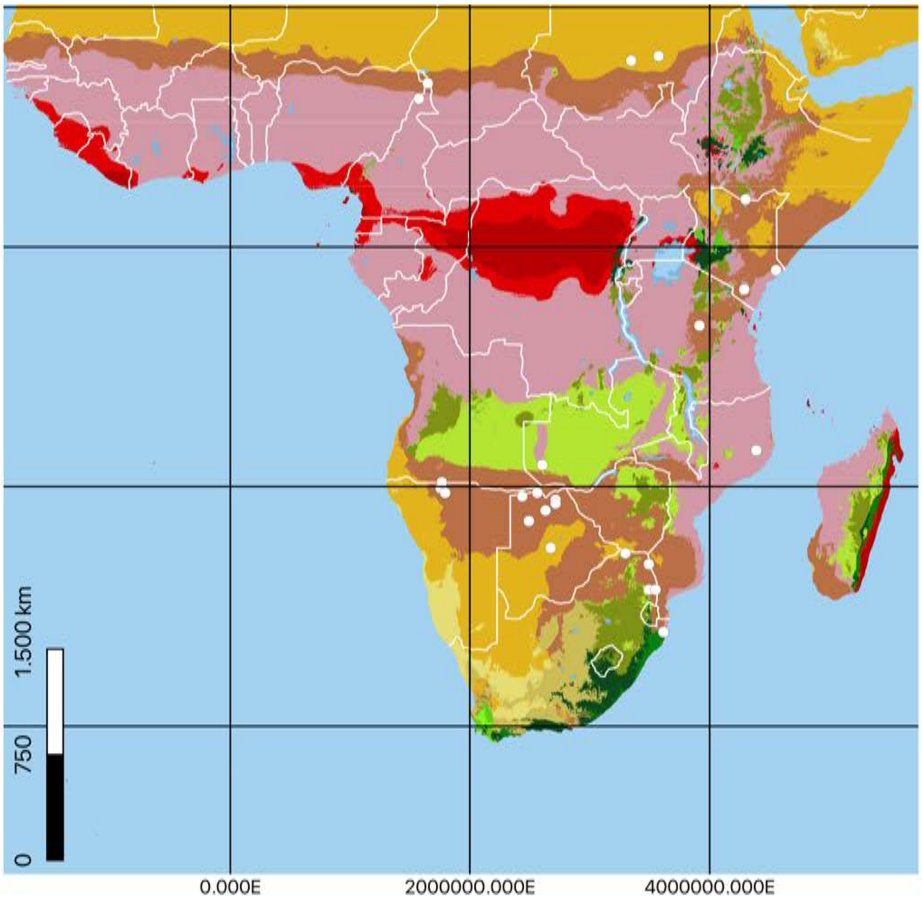

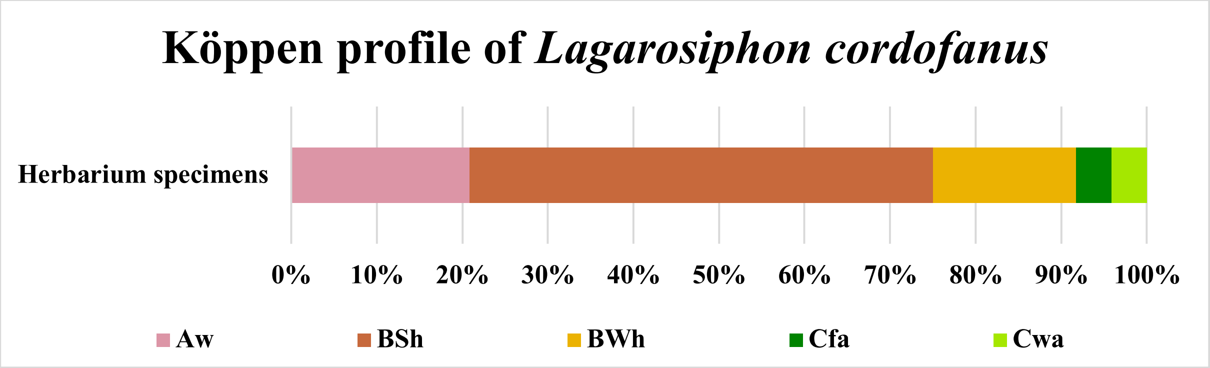

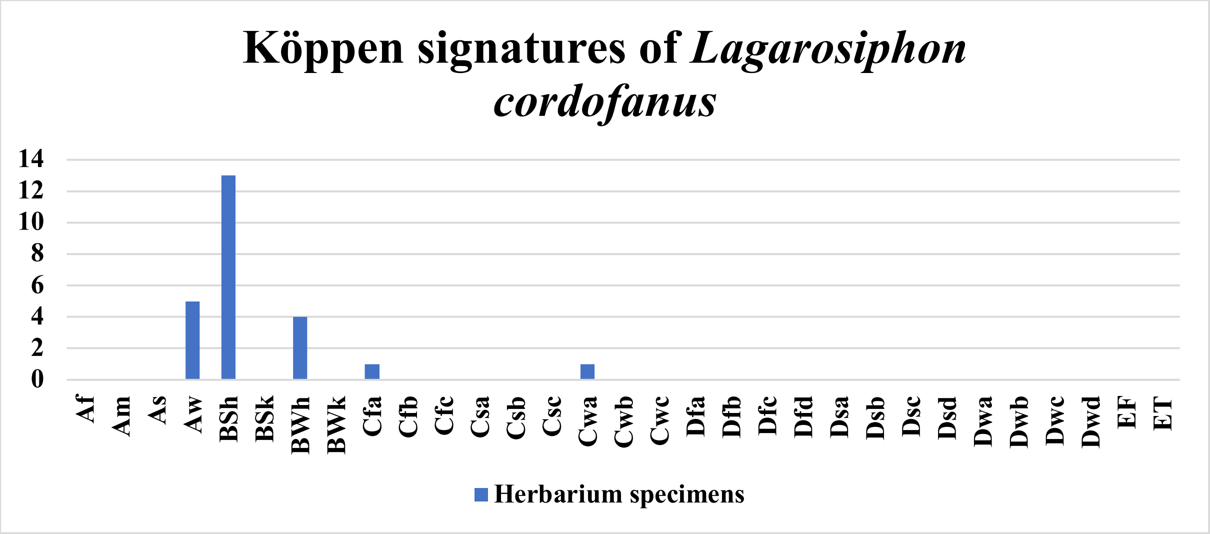

Köppen signatures of *Lagarosiphon cordofanus*
14
12
10
8
6
4
2
0
Af
Am
As
Aw
BSh
BSk
BWh
BWk
Cfa
Cfb
Cfc
Csa
Csb
Csc
Cwa
Cwb
Cwc
Dfa
Dfb
Dfc
Dfd
Dsa
Dsb
Dsc
Dsd
Dwa
Dwb
Dwc
Dwd
EF
ET
Herbarium specimens

5.2.1.1.2. Köppen profile, distribution and climate map– GBIF occurrences of *Lagarosiphon hydrilloides* Rendle 1895; herbarium specimens excluding duplicate occurrences (n = 8).

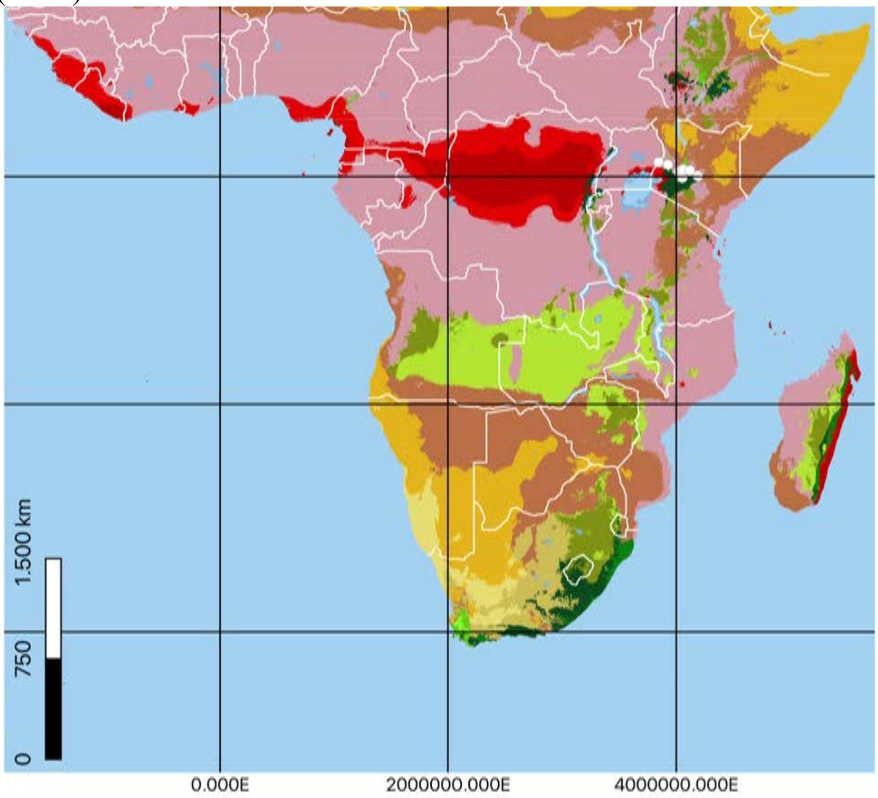

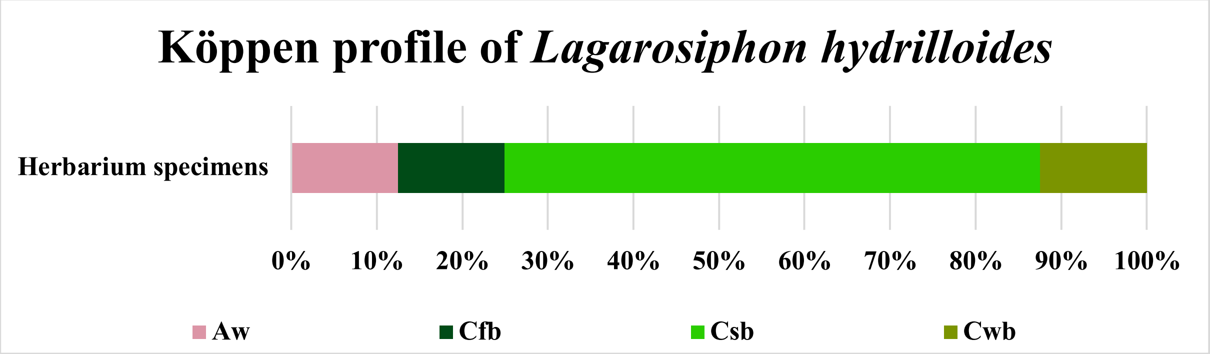

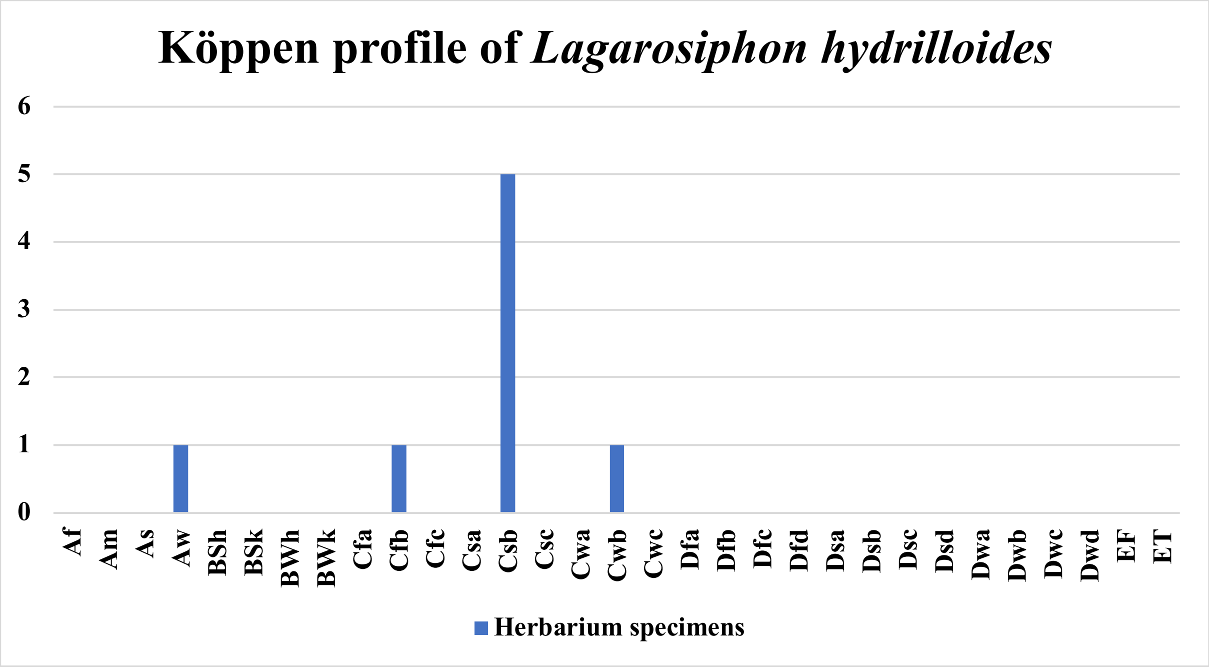
Köppen profile of *Lagarosiphon hydrilloides*
6
5
4
3
2
1
0
Af
Am
As
Aw
BSh
BSk
BWh
BWk
Cfa
Cfb
Cfc
Csa
Csb
Csc
Cwa
Cwb
Cwc
Dfa
Dfb
Dfc
Dfd
Dsa
Dsb
Dsc
Dsd
Dwa
Dwb
Dwc
Dwd
EF
ET
Herbarium specimens

5.2.1.1.3. Köppen profile, distribution and climate map–GBIF occurrences of *Lagarosiphon ilicifolius* Oberm. 1964; herbarium specimens excluding duplicate occurrences (n = 27).

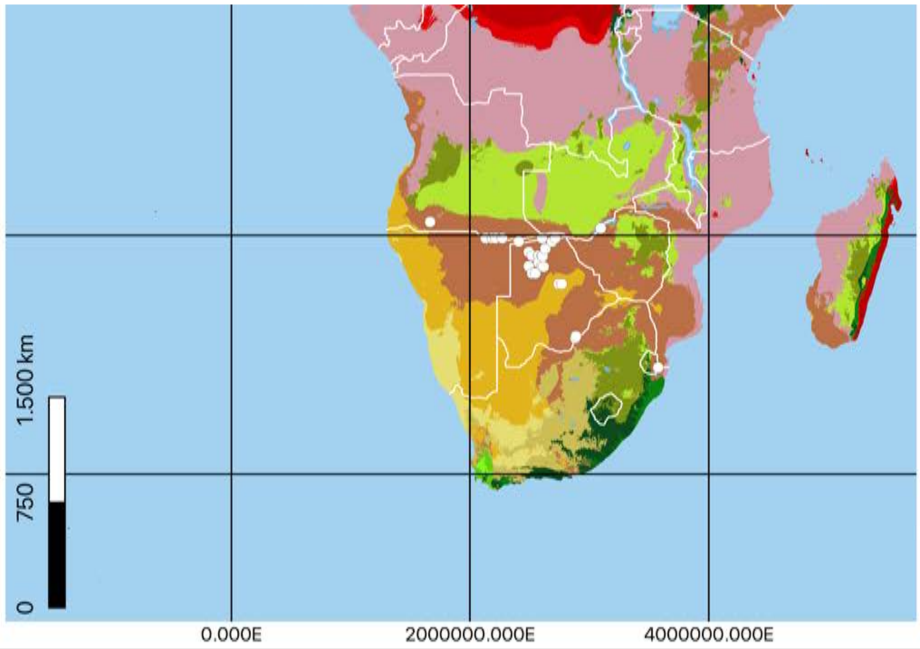

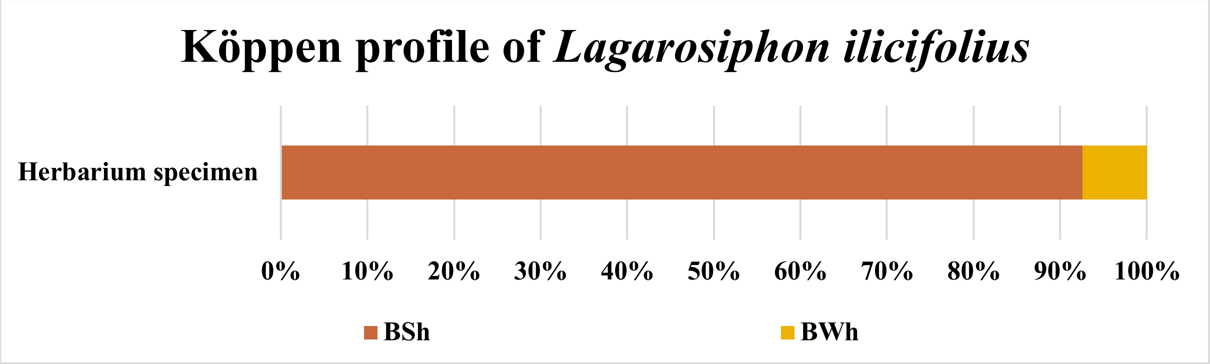

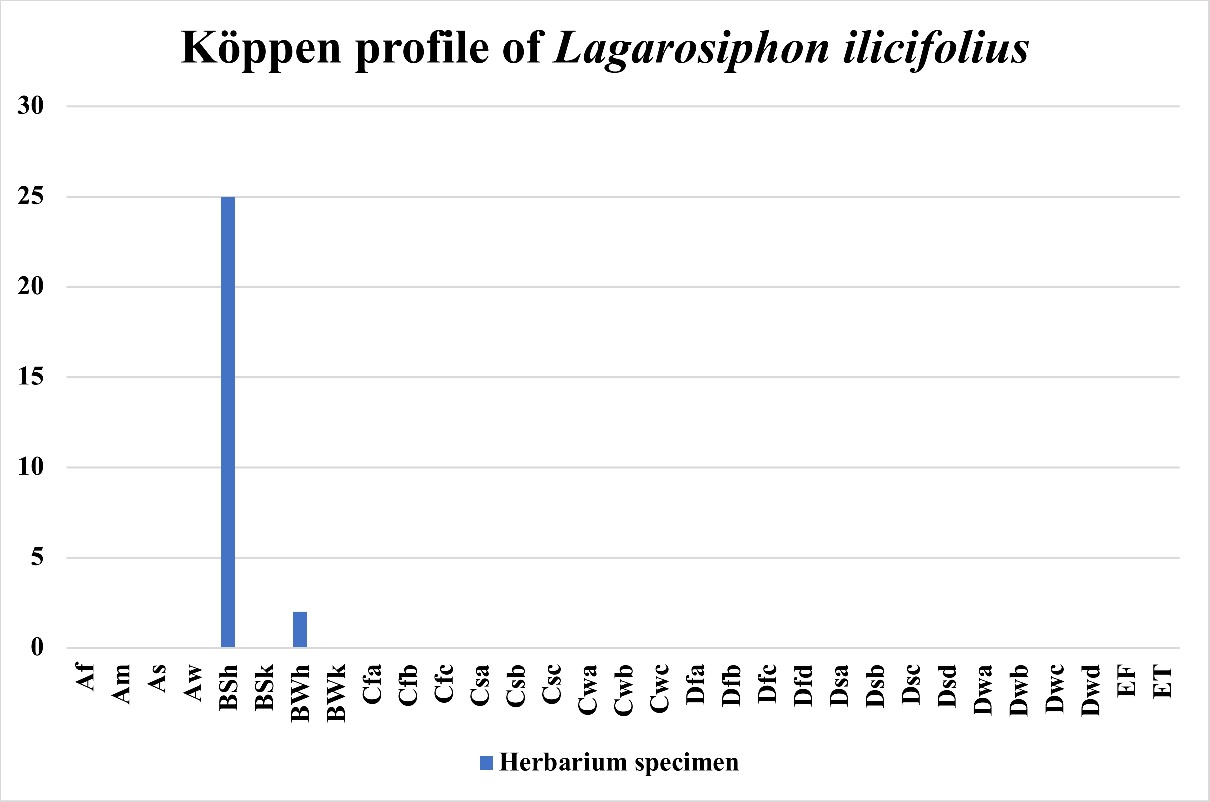
Köppen profile of *Lagarosiphon ilicifolius*
30
25
20
15
10
5
0
Af
Am
As
Aw
BSh
BSk
BWh
BWk
Cfa
Cfb
Cfc
Csa
Csb
Csc
Cwa
Cwb
Cwc
Dfa
Dfb
Dfc
Dfd
Dsa
Dsb
Dsc
Dsd
Dwa
Dwb
Dwc
Dwd
EF
ET
Herbarium specimen

5.2.1.1.3. Köppen profile, distribution and climate map–GBIF occurrences of *Lagarosiphon madagascariensis* Harv. 1841; herbarium specimens excluding duplicate occurrences (n = 40).

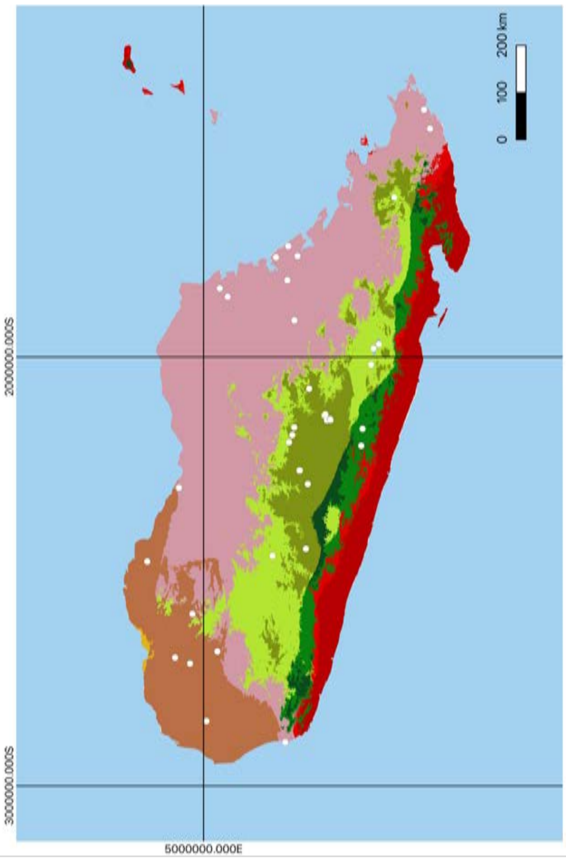

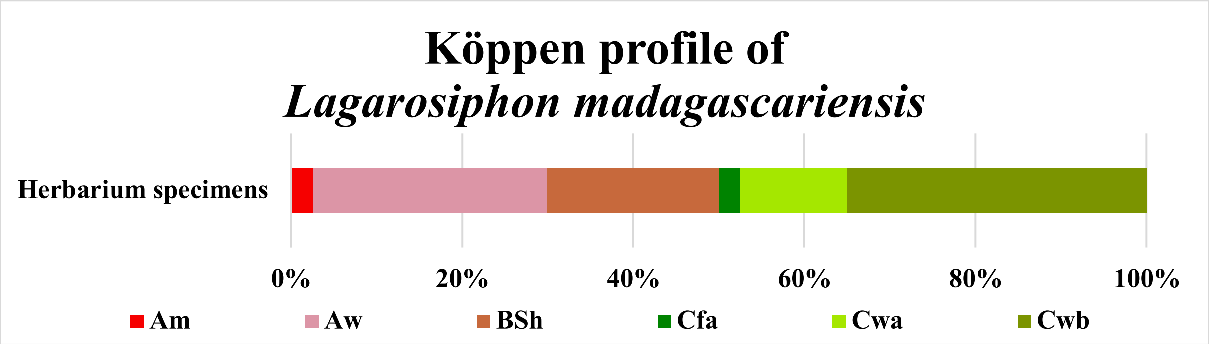

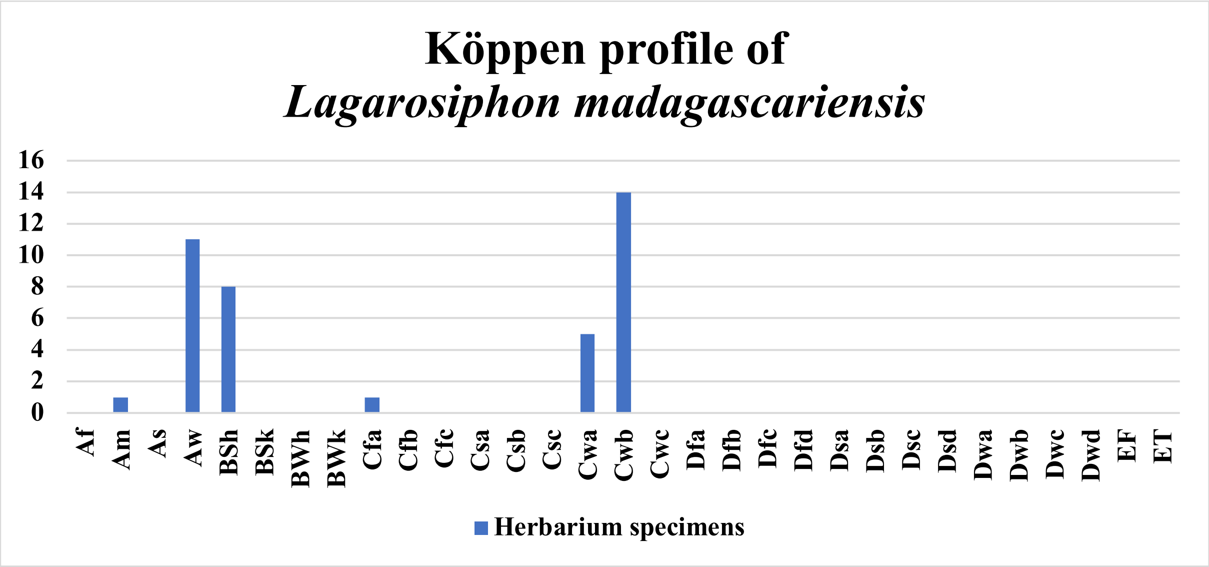

5.2.1.1.4. Köppen profile, distribution and climate map–GBIF occurrences of *Lagarosiphon major* (Ridl. 1886) Moss 1928; herbarium specimens excluding duplicate occurrences (n = 50).

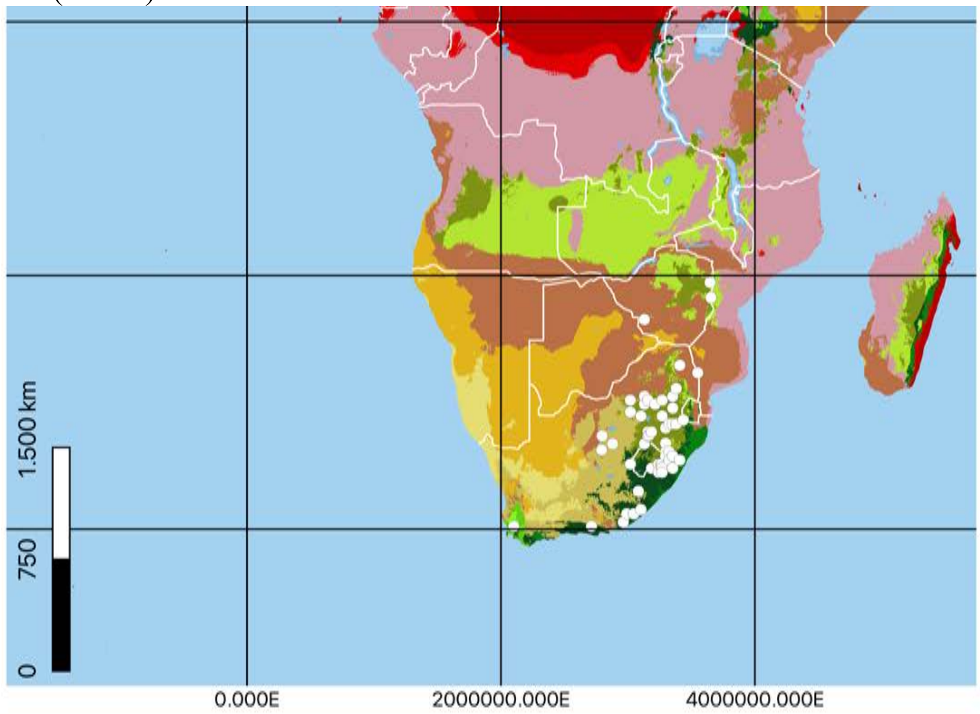

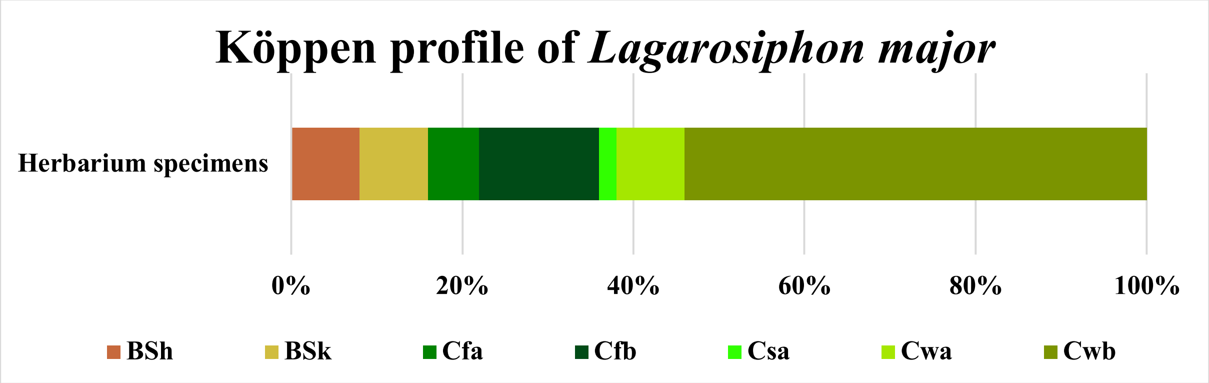

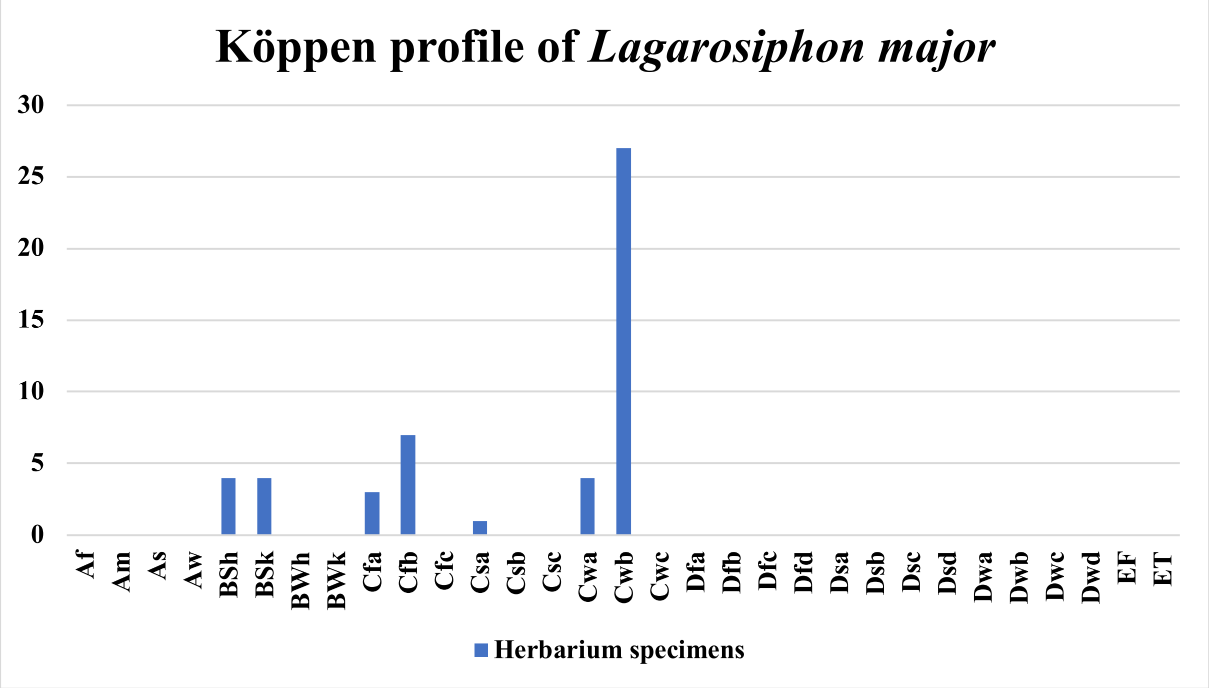

5.2.1.1.5. Köppen profile, distribution and climate map– GBIF occurrences of *Lagarosiphon muscoides* Harv. 1841; herbarium specimens excluding duplicate occurrences (n = 87).

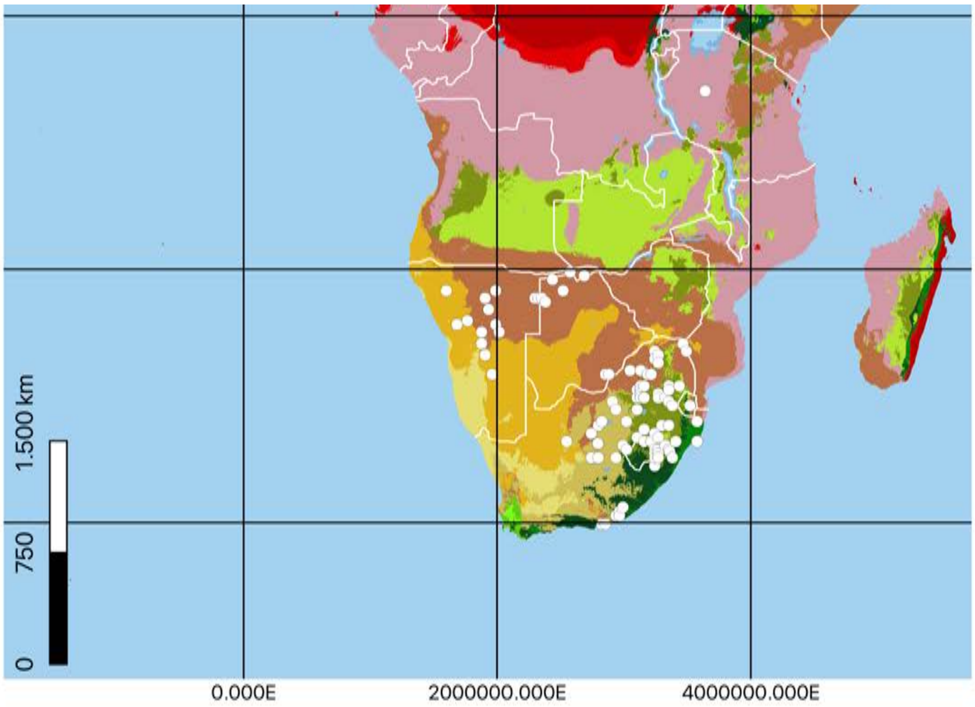

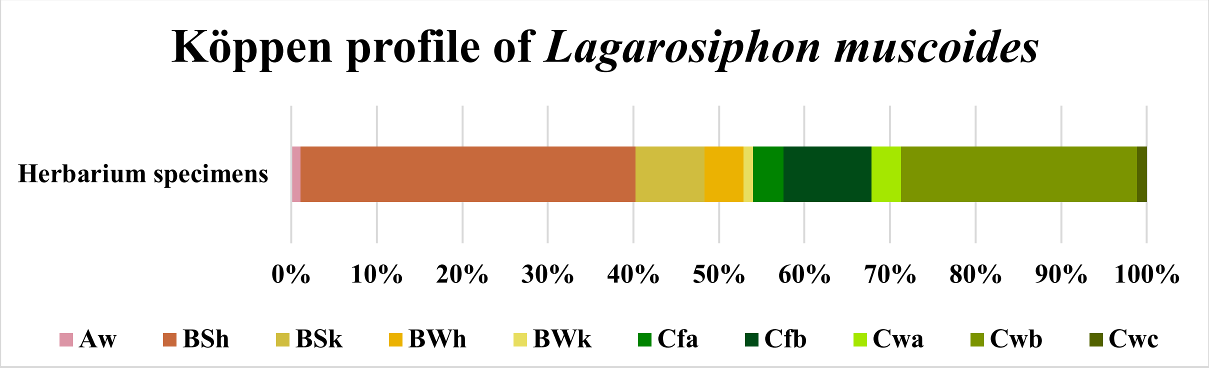

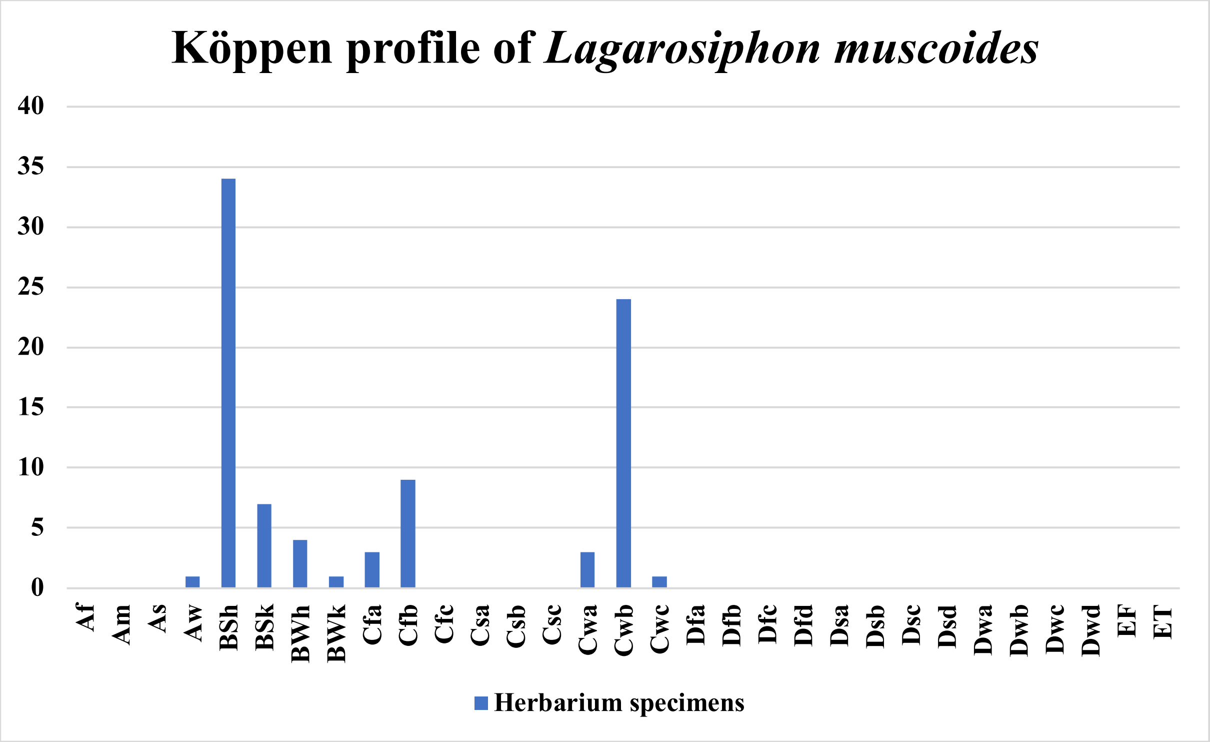

Köppen profile of *Lagarosiphon muscoides*
40
35
30
25
20
15
10
5
0
Af
Am
As
Aw
BSh
BSk
BWh
BWk
Cfa
Cfb
Cfc
Csa
Csb
Csc
Cwa
Cwb
Cwc
Dfa
Dfb
Dfc
Dfd
Dsa
Dsb
Dsc
Dsd
Dwa
Dwb
Dwc
Dwd
EF
ET
Herbarium specimens

5.2.1.1.6. Köppen profile, distribution and climate map–GBIF occurrences of *Lagarosiphon rubellus* Ridl. 1886; herbarium specimens excluding duplicate occurrences (n = 1).

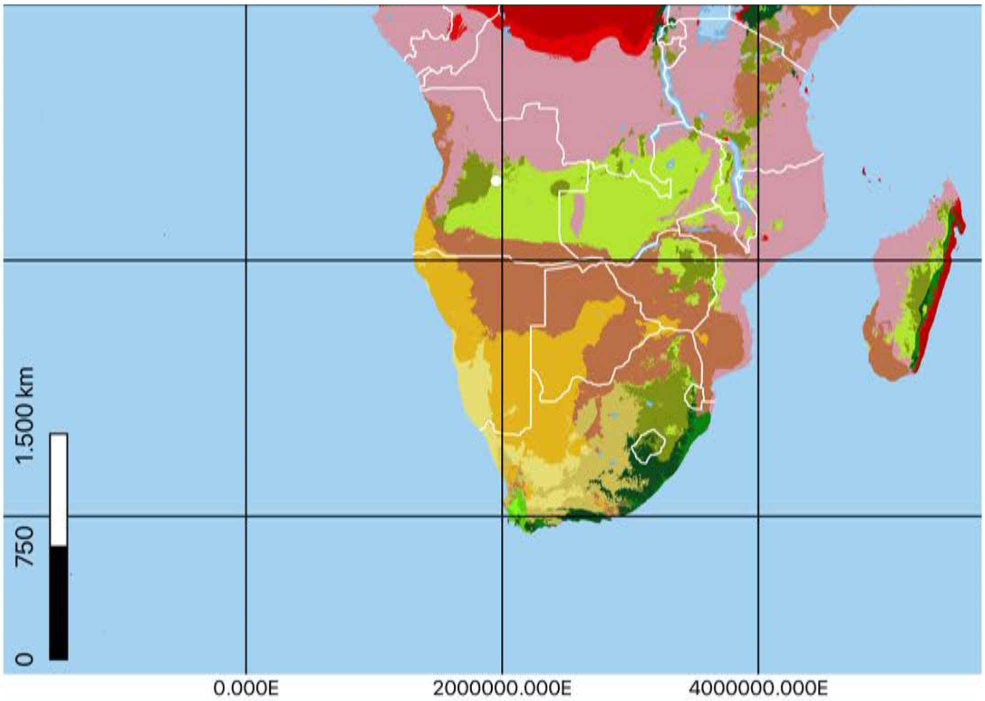

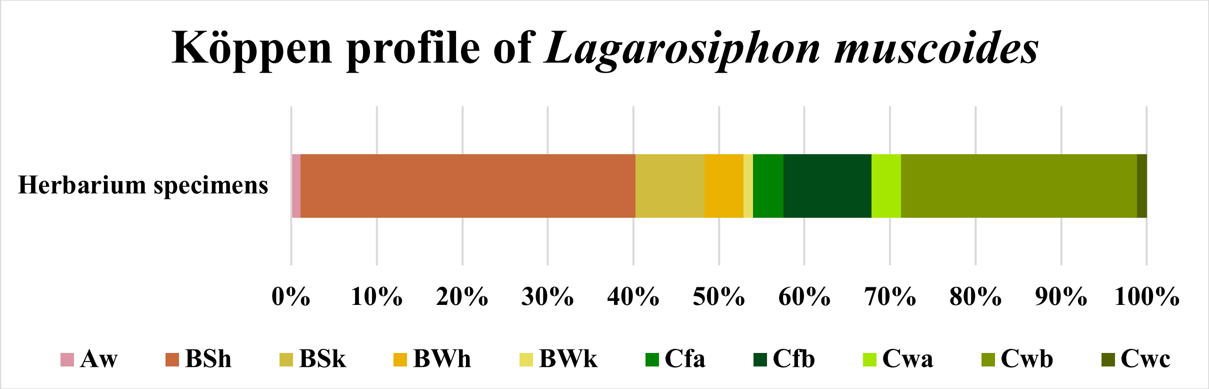

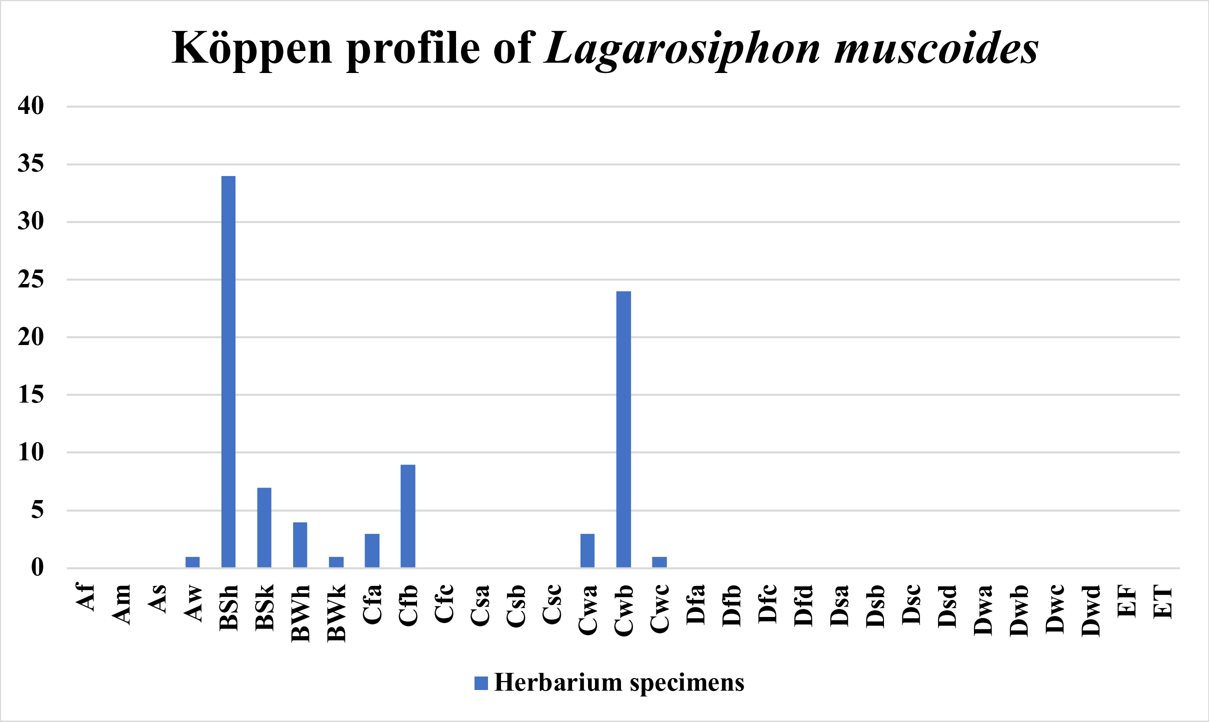

Köppen profile of *Lagarosiphon muscoides*
40
35
30
25
20
15
10
5
0
Af
Am
As
Aw
BSh
BSk
BWh
BWk
Cfa
Cfb
Cfc
Csa
Csb
Csc
Cwa
Cwb
Cwc
Dfa
Dfb
Dfc
Dfd
Dsa
Dsb
Dsc
Dsd
Dwa
Dwb
Dwc
Dwd
EF
ET
Herbarium specimens

5.2.1.1.6. Köppen profile, distribution and climate map– GBIF occurrences of *Lagarosiphon steudneri* Casp. 1858; herbarium specimens excluding duplicate occurrences (n = 1).

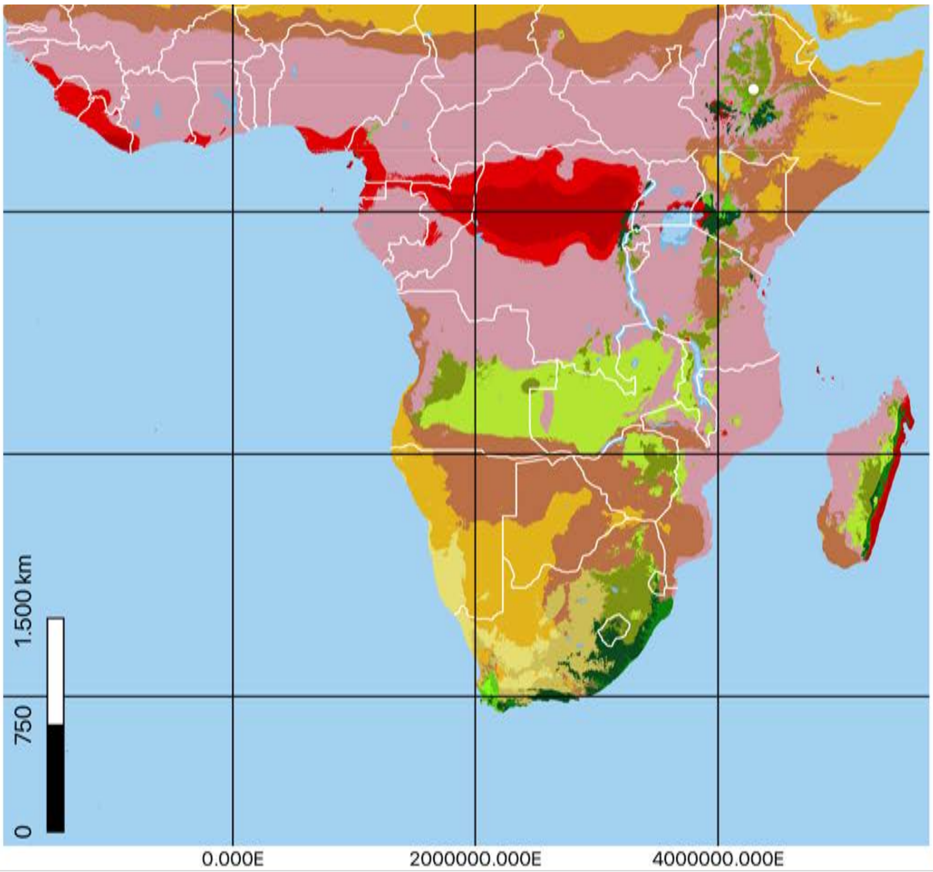

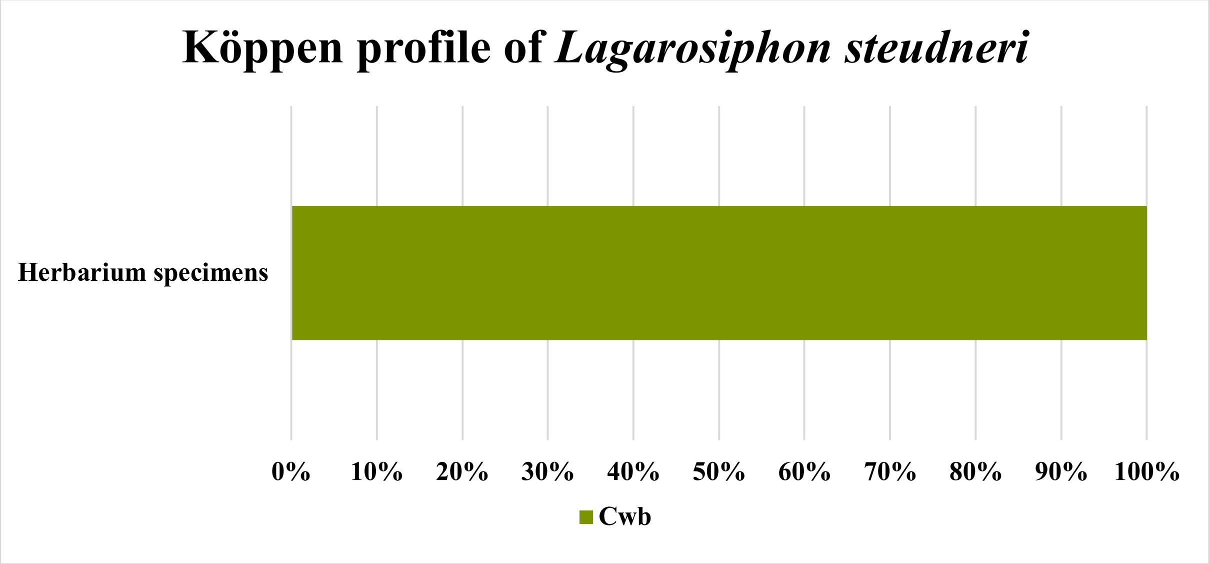

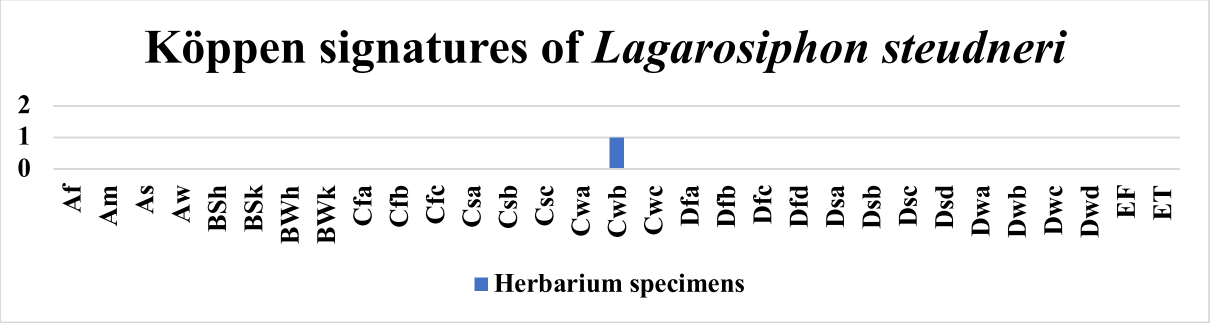

5.2.1.1.6. Köppen profile, distribution and climate map– GBIF occurrences of *Lagarosiphon verticillifolius* Oberm. 1964; herbarium specimens excluding duplicate occurrences (n = 19).

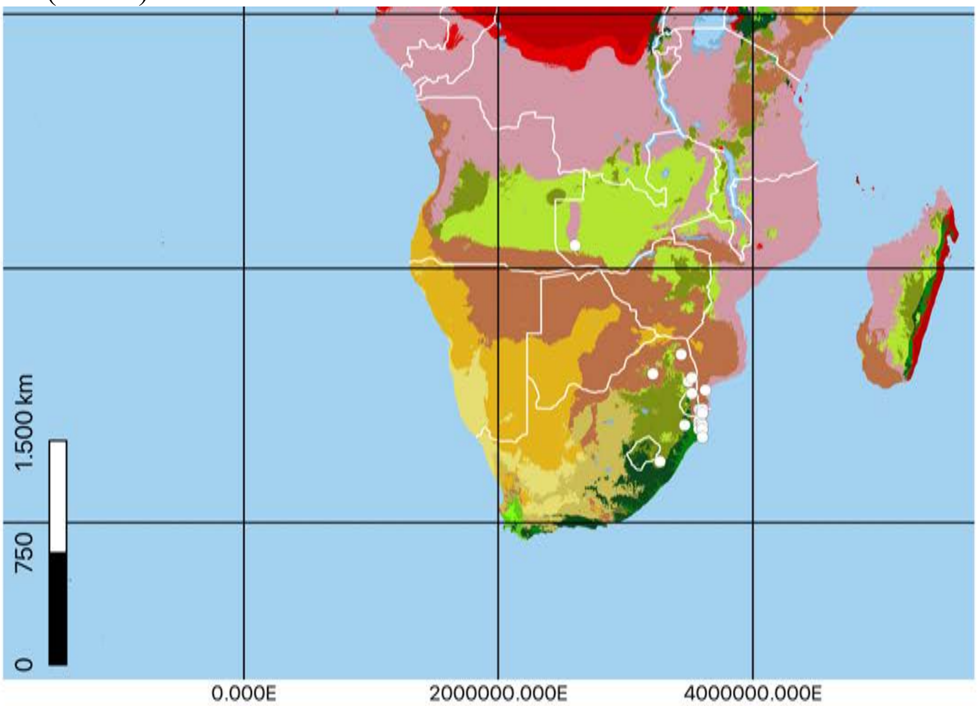

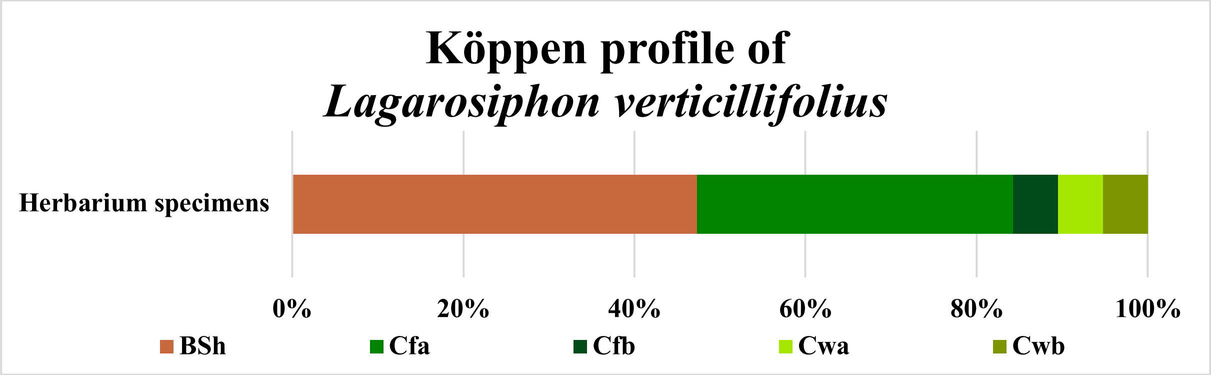

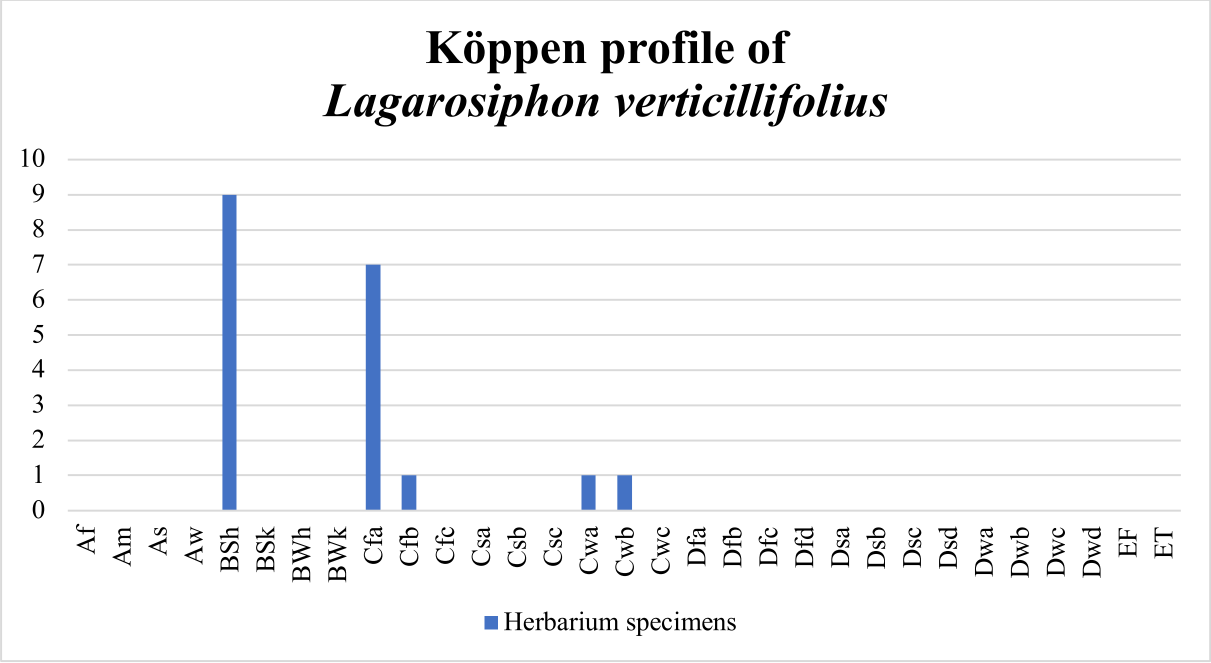

5.2.2. Second diverging genera within Clade B of Hydrocharitaceae cf. Chen et. al (2012), comprising *Egeria* Planch. 1849 and *Elodea* Michx. 1803 (native distribution)

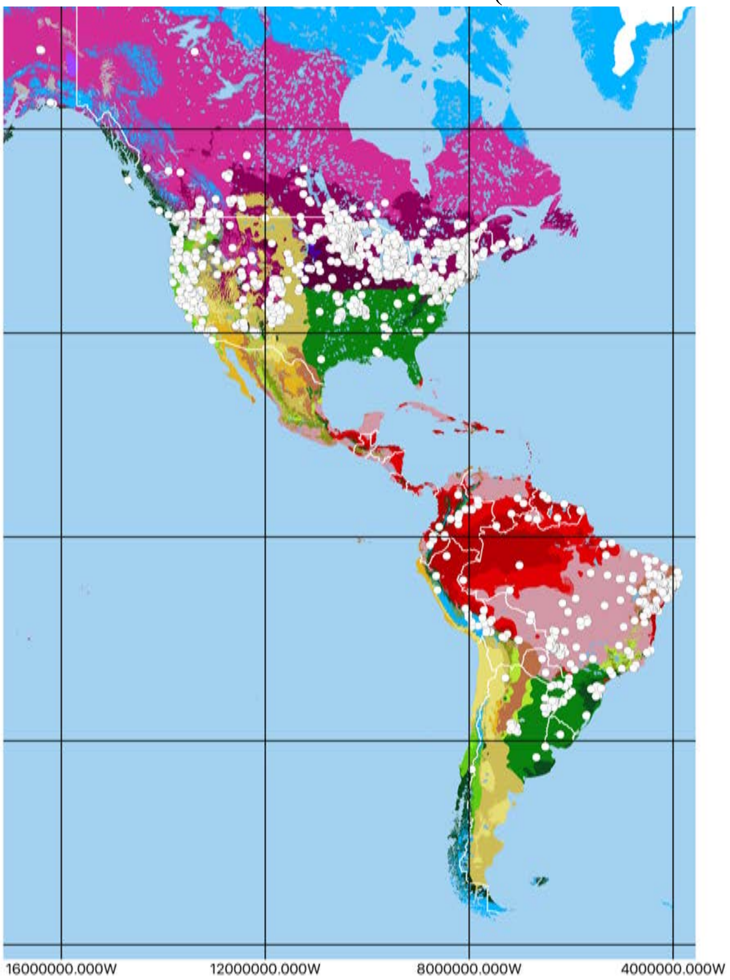

Egeria and *Elodea* preserved specimens, native and neophyte distribution.

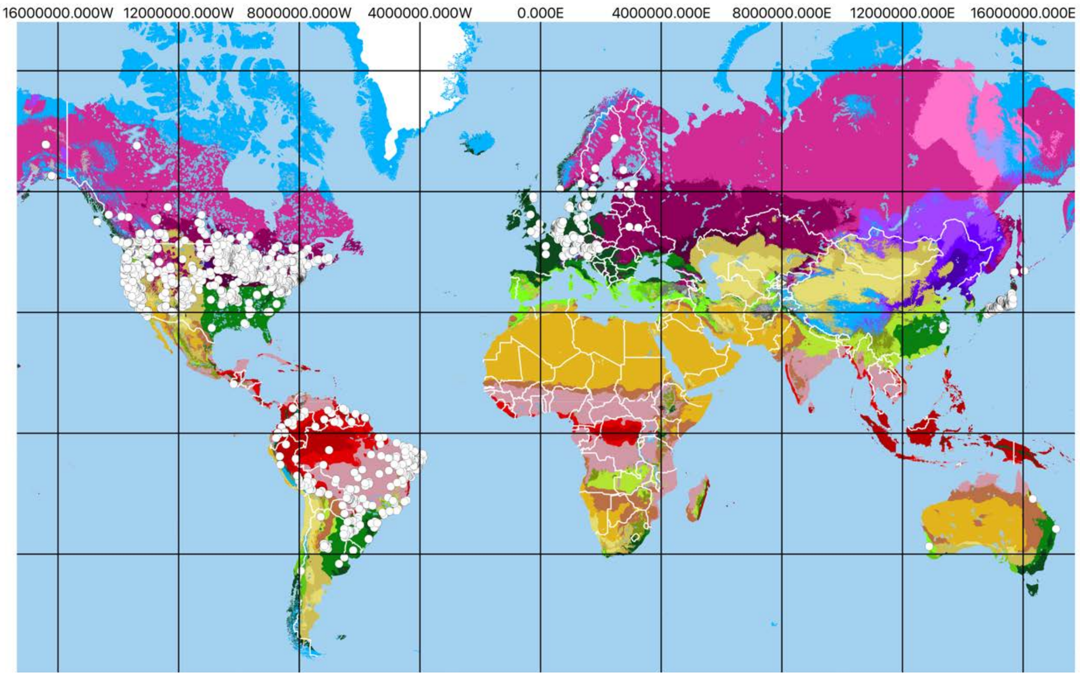

5.2.2.1 Genus *Egeria* Planch. 1849.
Distribution and climate map and Köppen profiles for *Egeria* incl. *Egeria densa*, *E. heterostemon*, and *E. najas,* based on herbarium specimens excluding duplicate occurrences (n = 241).

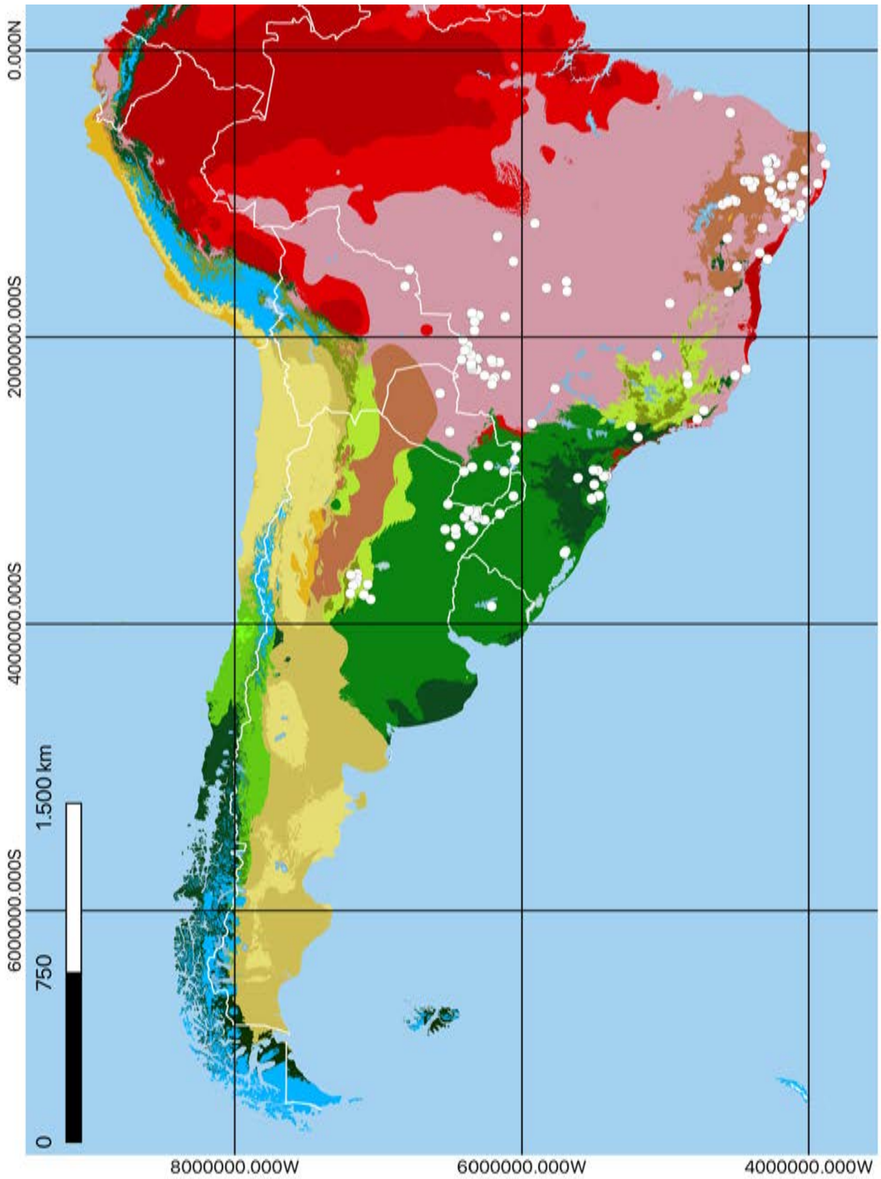

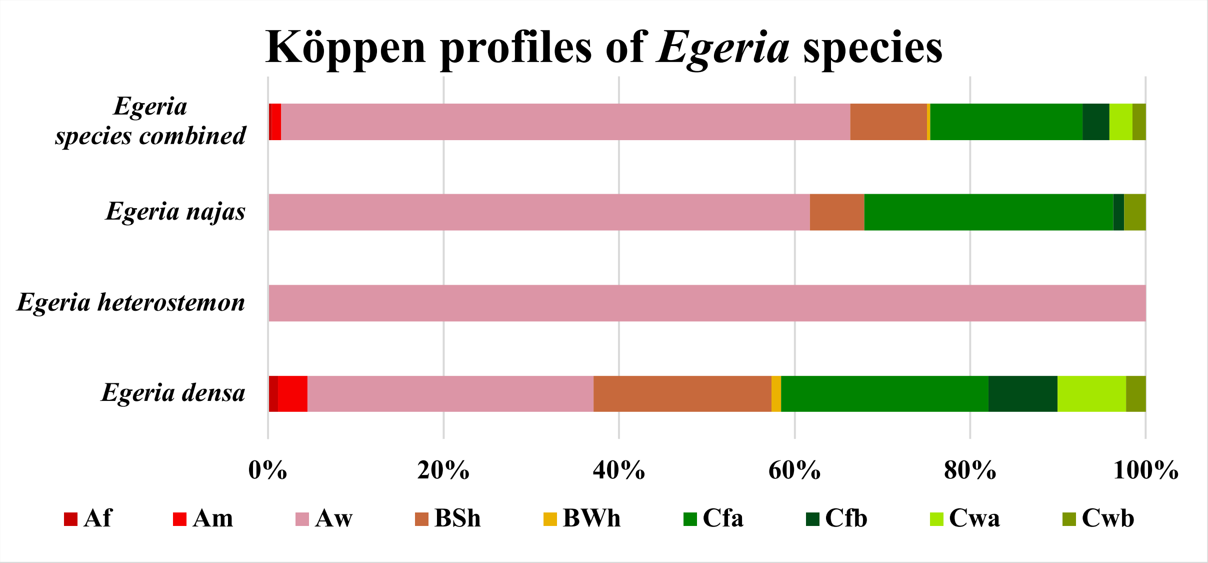

5.2.2.1.1. Köppen profile, distribution and climate map– GBIF occurrences of *Egeria densa* Planch. 1949; herbarium specimens excluding duplicate occurrences (n = 89).

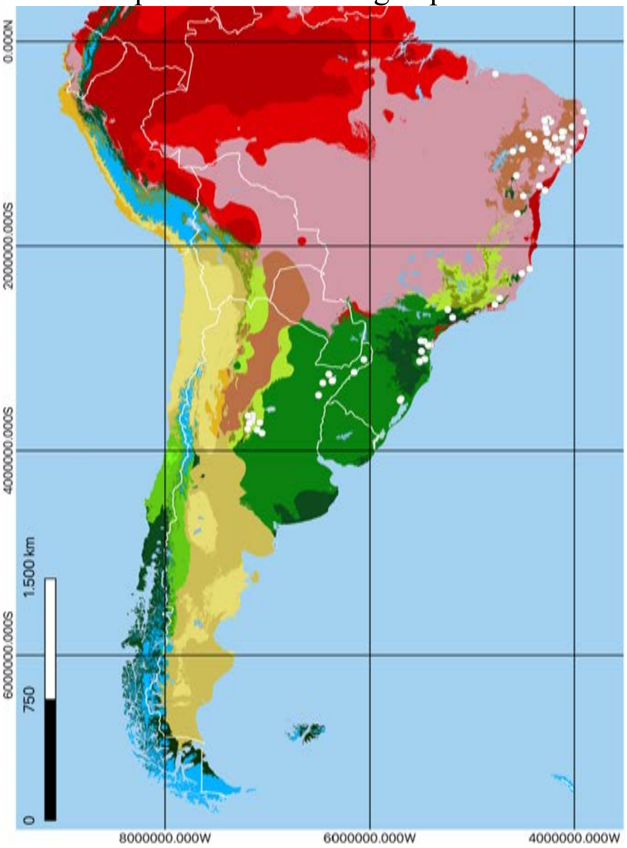

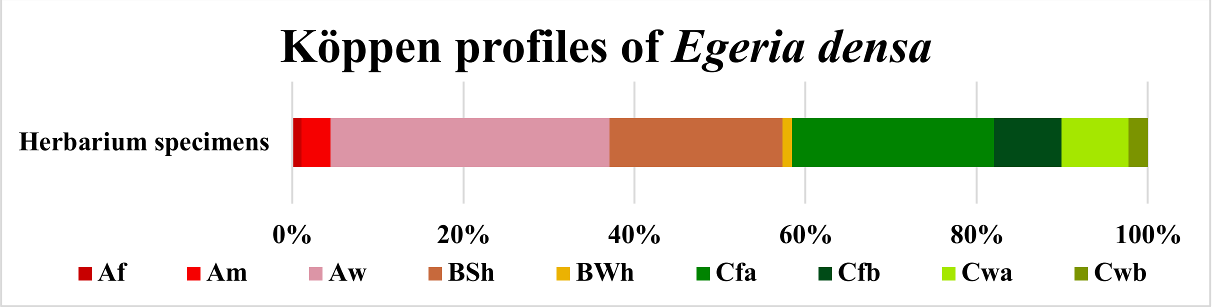

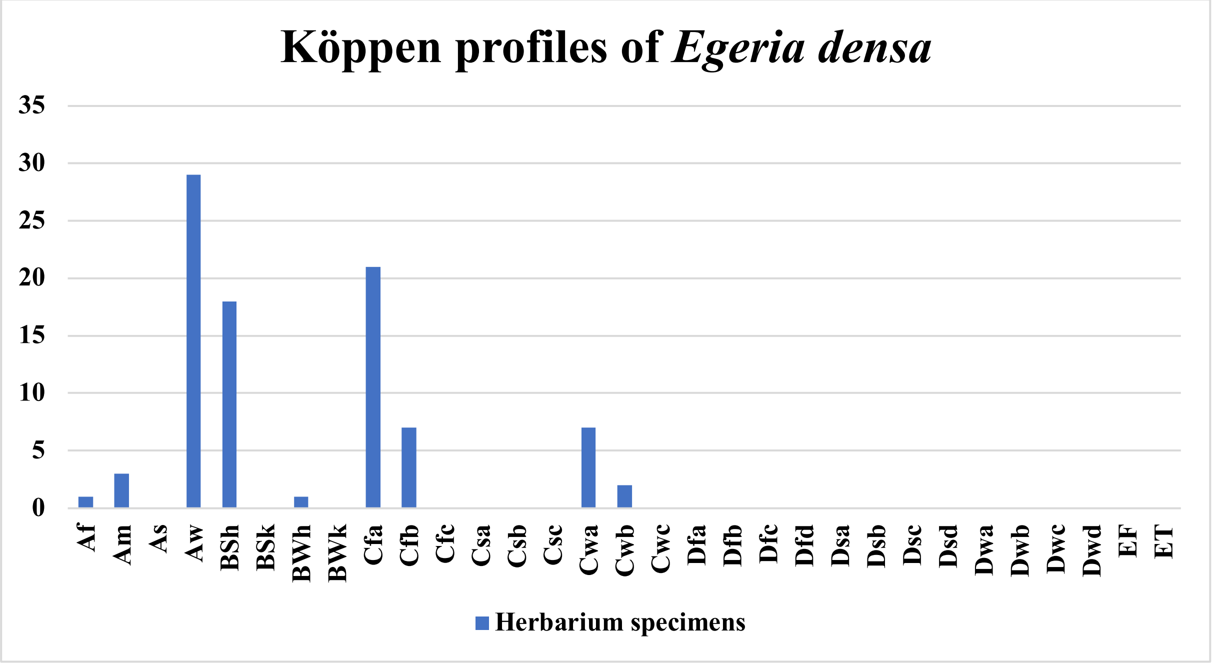

5.2.2.1.2. Köppen profile, distribution and climate map– GBIF occurrences of *Egeria heterostemon* S.Koehler & C.P.Bove 2001; herbarium specimens excluding duplicate occurrences (n = 2).

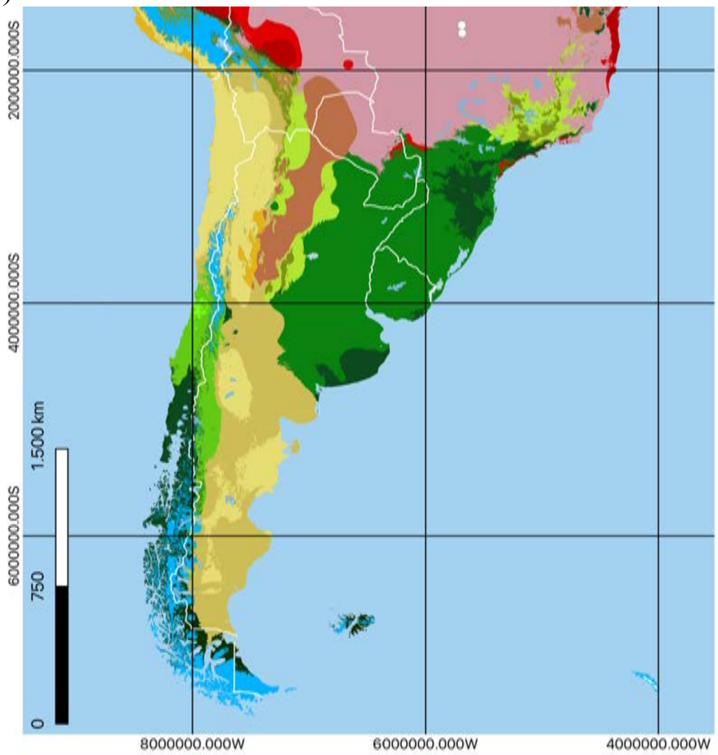

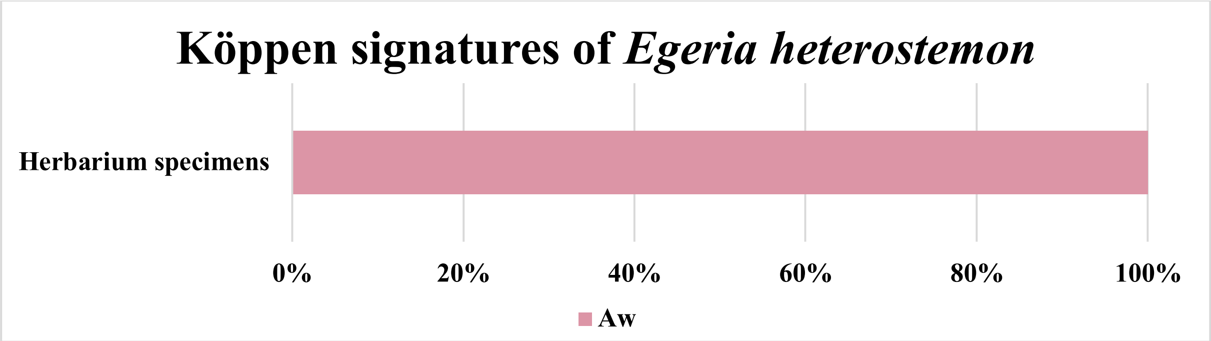

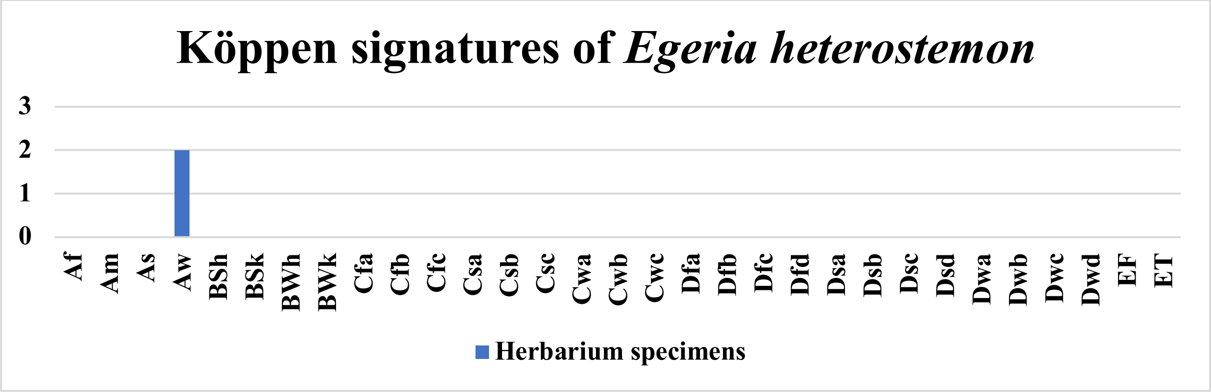

5.2.2.1.3. Köppen profile, distribution and climate map– GBIF occurrences of *Egeria najas* Planch. 1849; herbarium specimens excluding duplicate occurrences (n = 81).

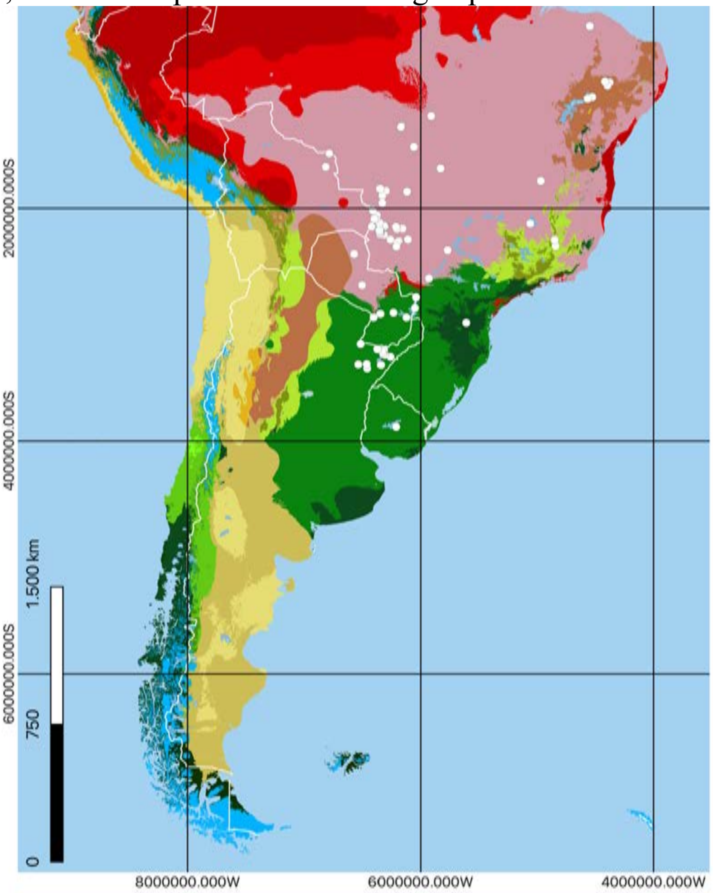

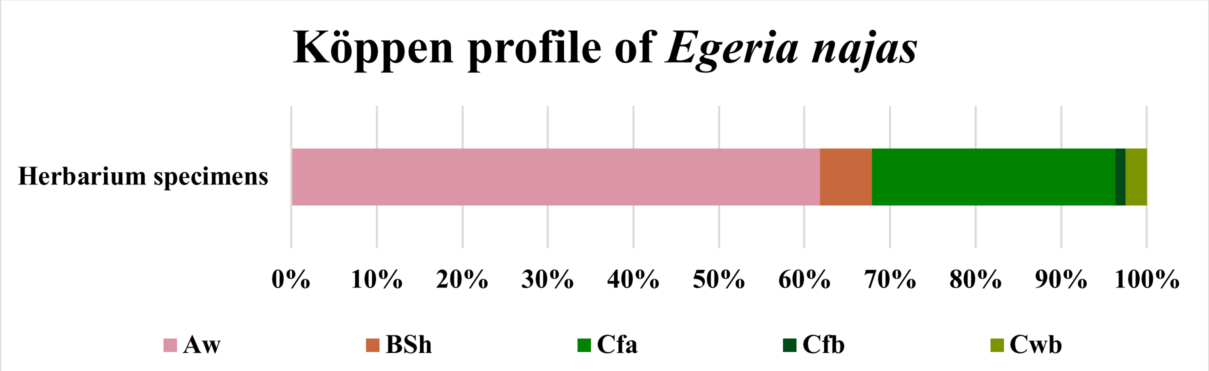

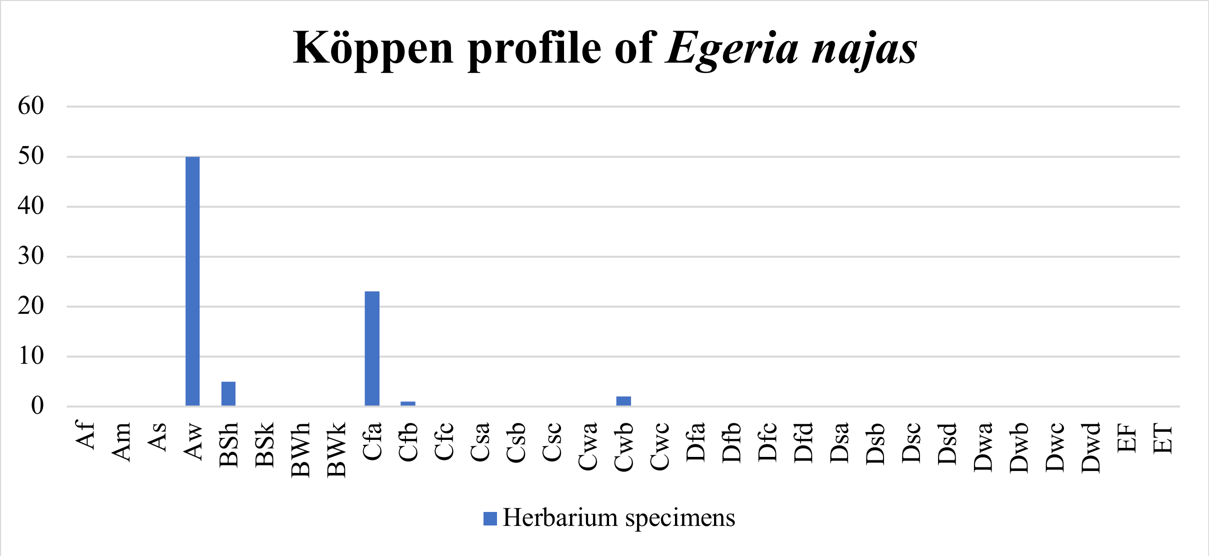

5.2.2.2. Genus *Elodea* Michx. 1803.

Distribution and Köppen profiles of genus *Elodea* incl. *E. bifoliata, E. callitrichoides, E. canadensis, E. granatensis, E. nuttalli,* and *E. potamogeton,* based on herbarium specimens (n = 2458).

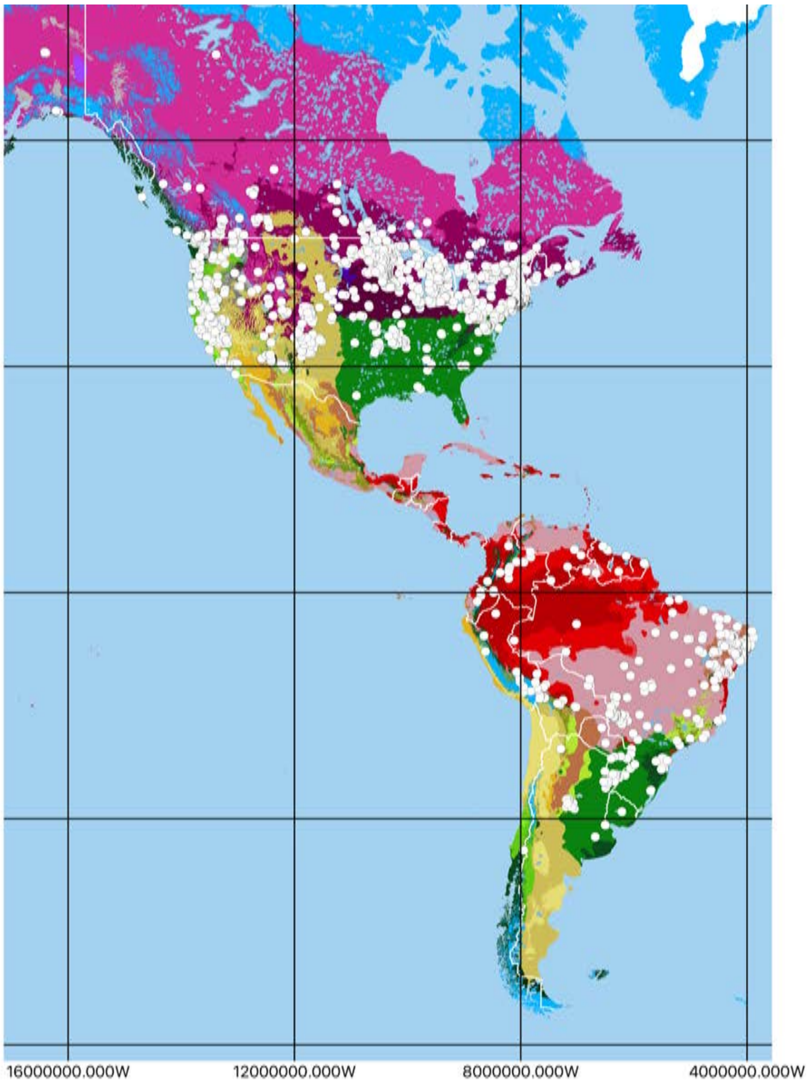

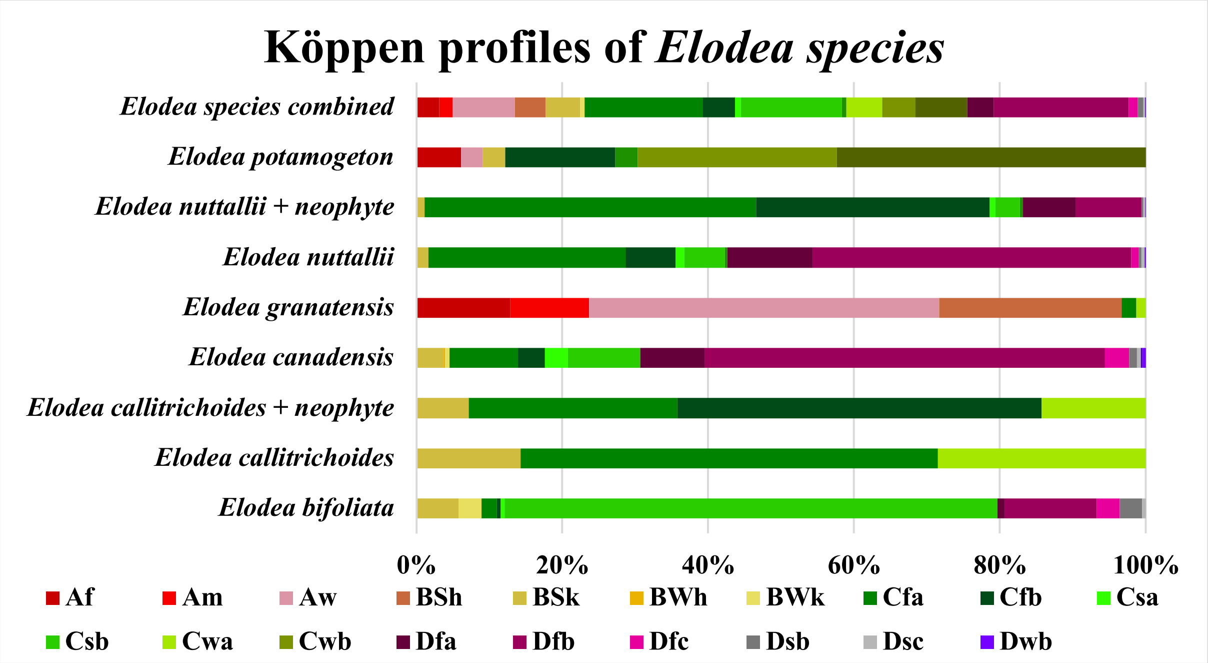

5.2.2.2.1. Köppen profile, distribution and climate map– GBIF occurrences of *Elodea bifoliata* H.St.John 1962; herbarium specimens excluding duplicate occurrences (n = 191).

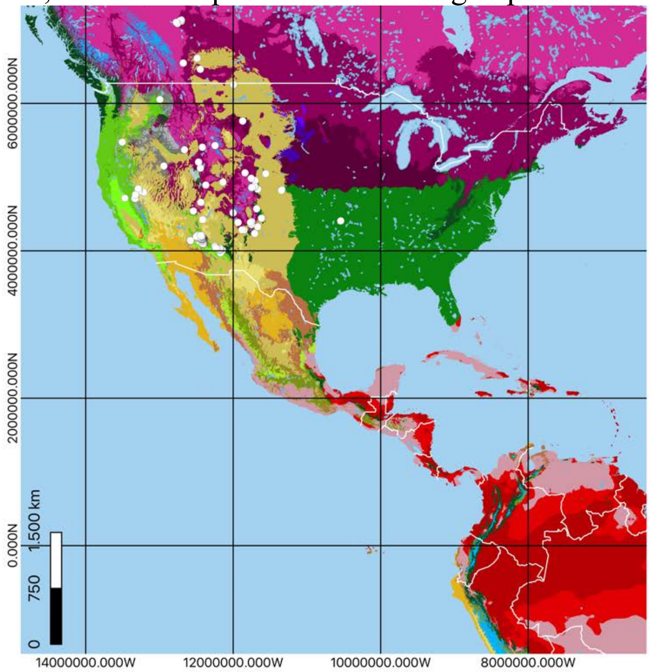

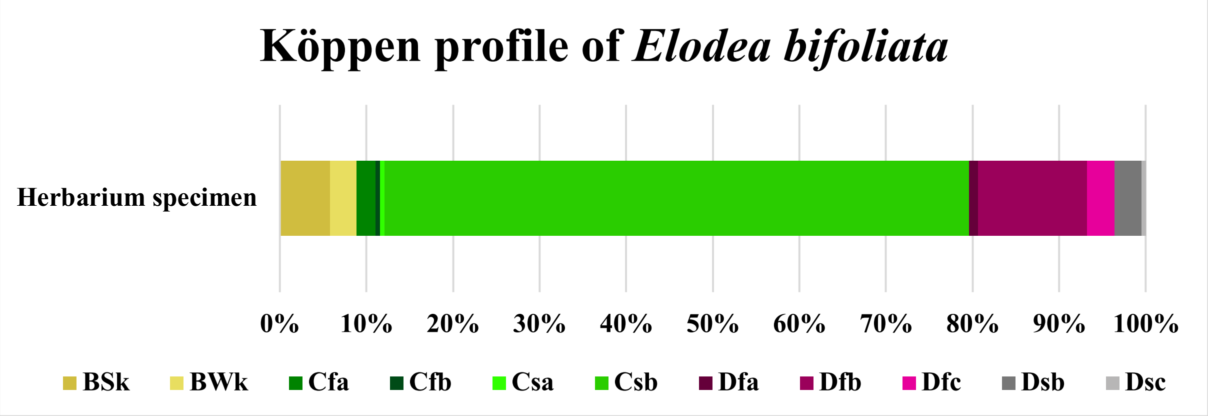

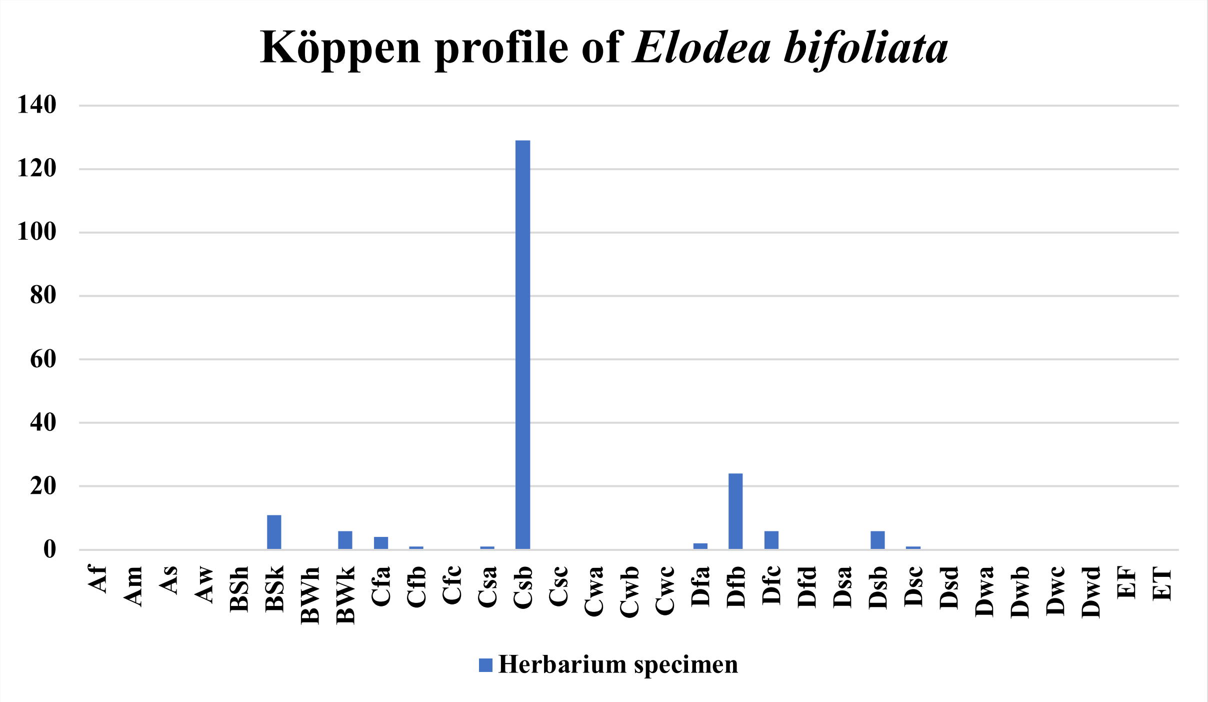

5.2.2.2.2. Köppen profile, distribution and climate map– GBIF occurrences of *Elodea callitrichoides* Casp. 1857, natural and neophyte distribution; herbarium specimen (n = 14).

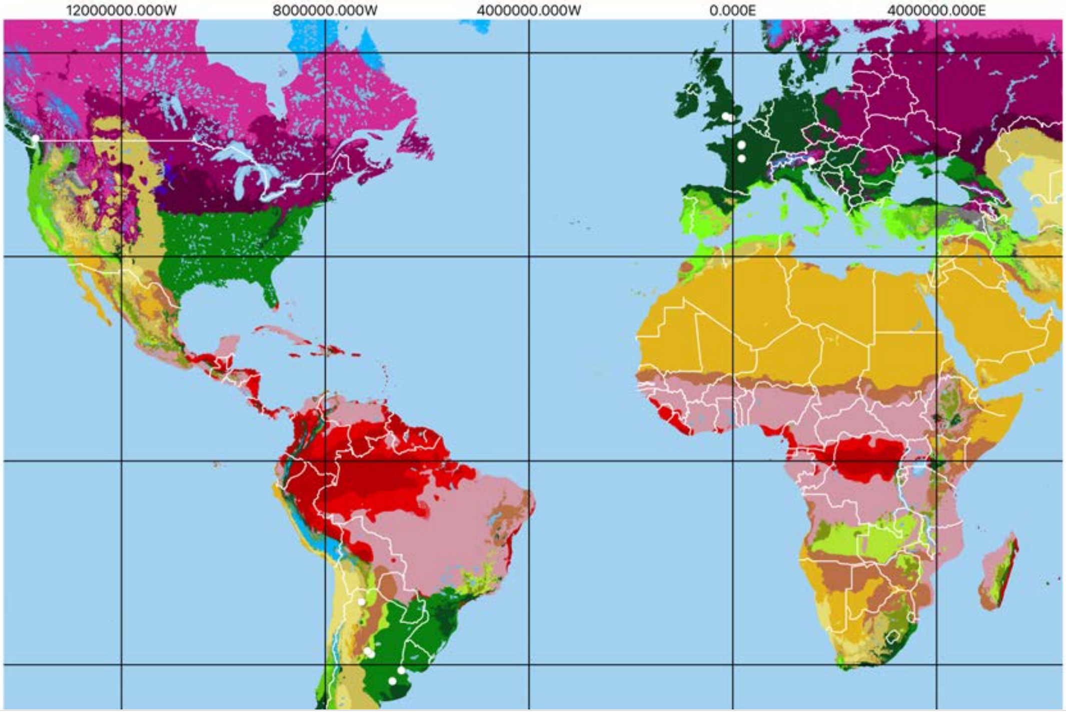

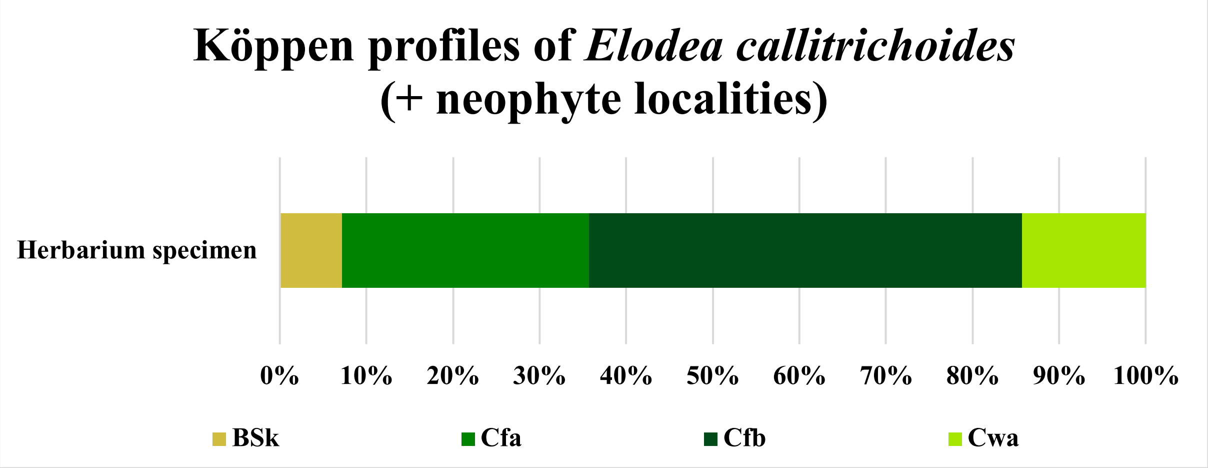

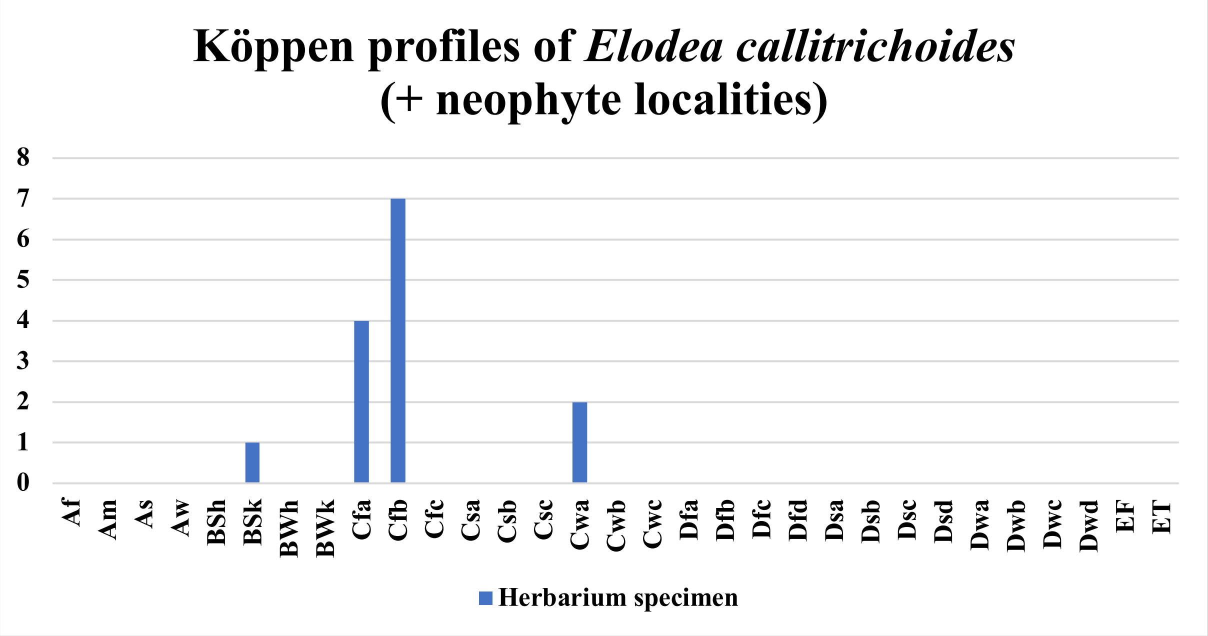

Köppen profile, distribution and climate map – GBIF occurrences of *Elodea callitrichoides*, natural distribution; herbarium specimens excluding duplicate occurrences (n = 7).

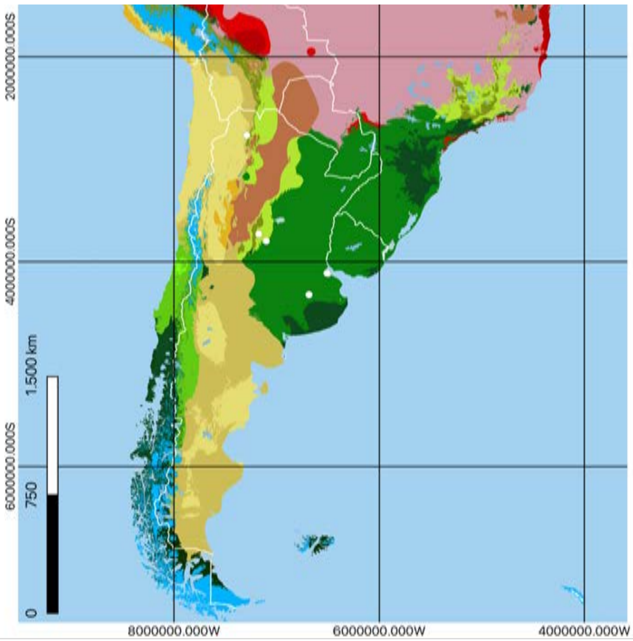

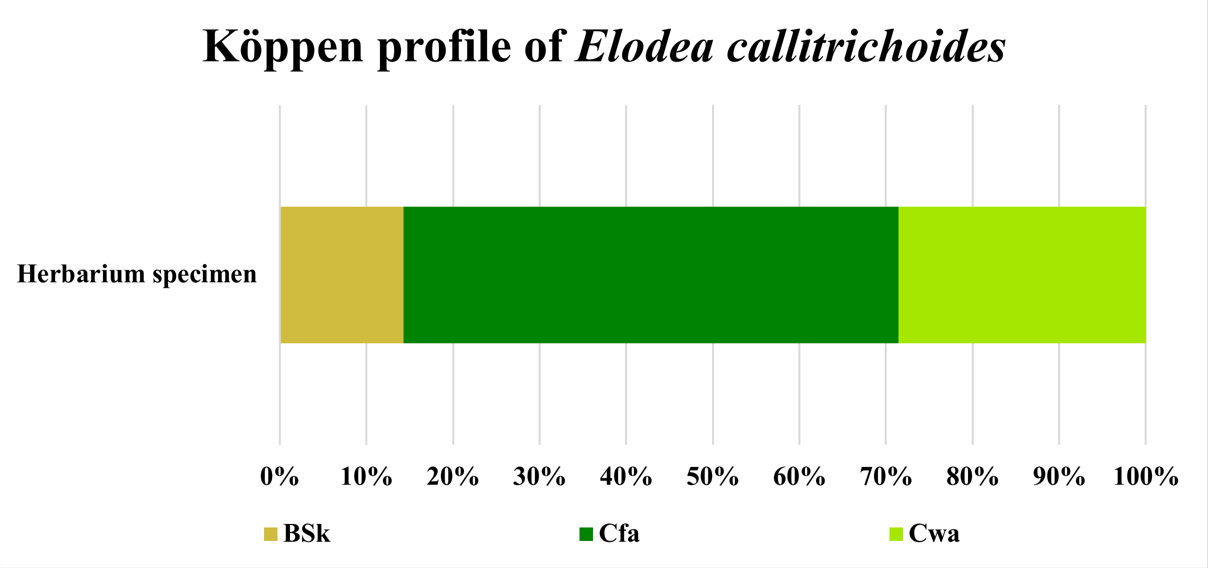

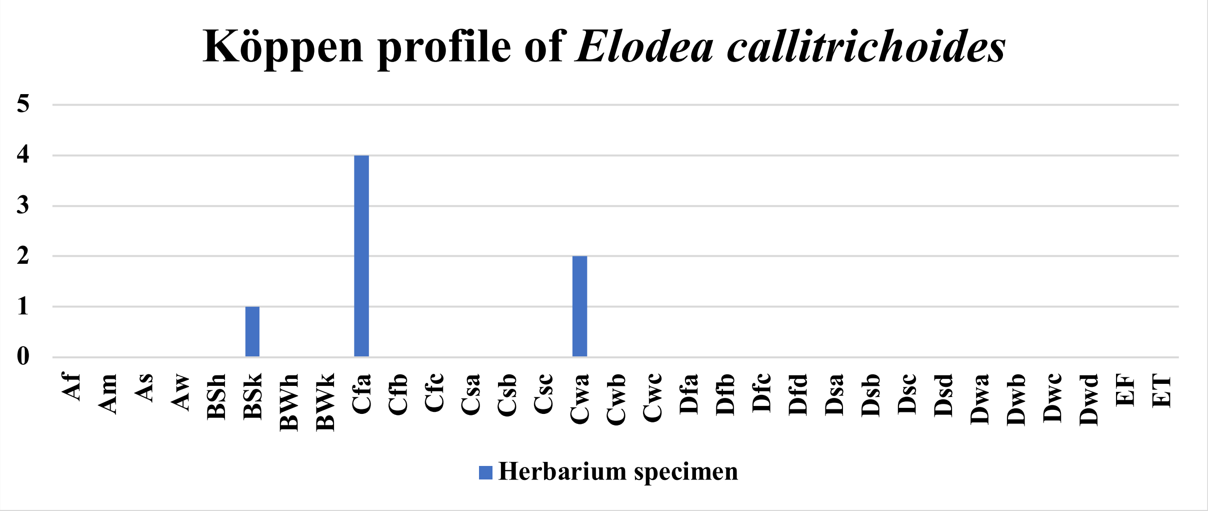

5.2.2.2.3. Köppen profile, distribution and climate map – GBIF occurrences of *Elodea canadensis* Michx. 1803; herbarium specimens excluding duplicate occurrences (n = 1134).

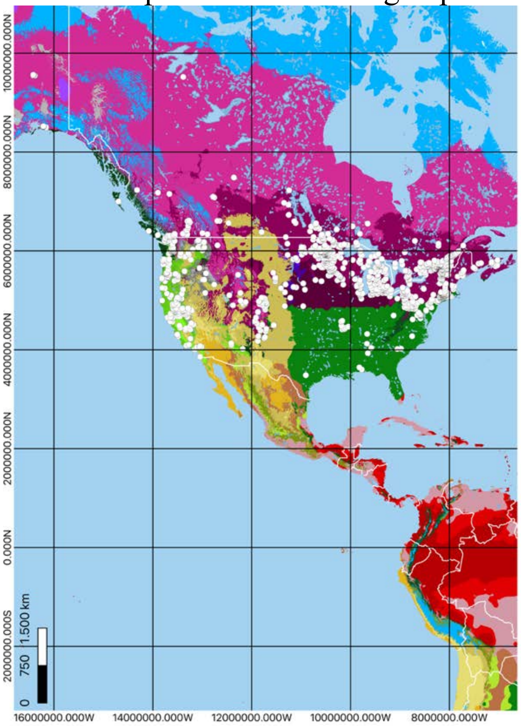

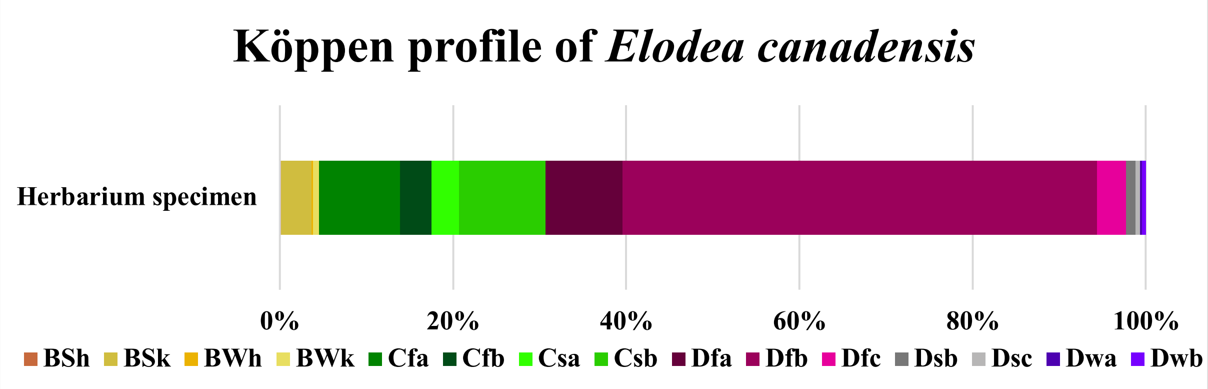

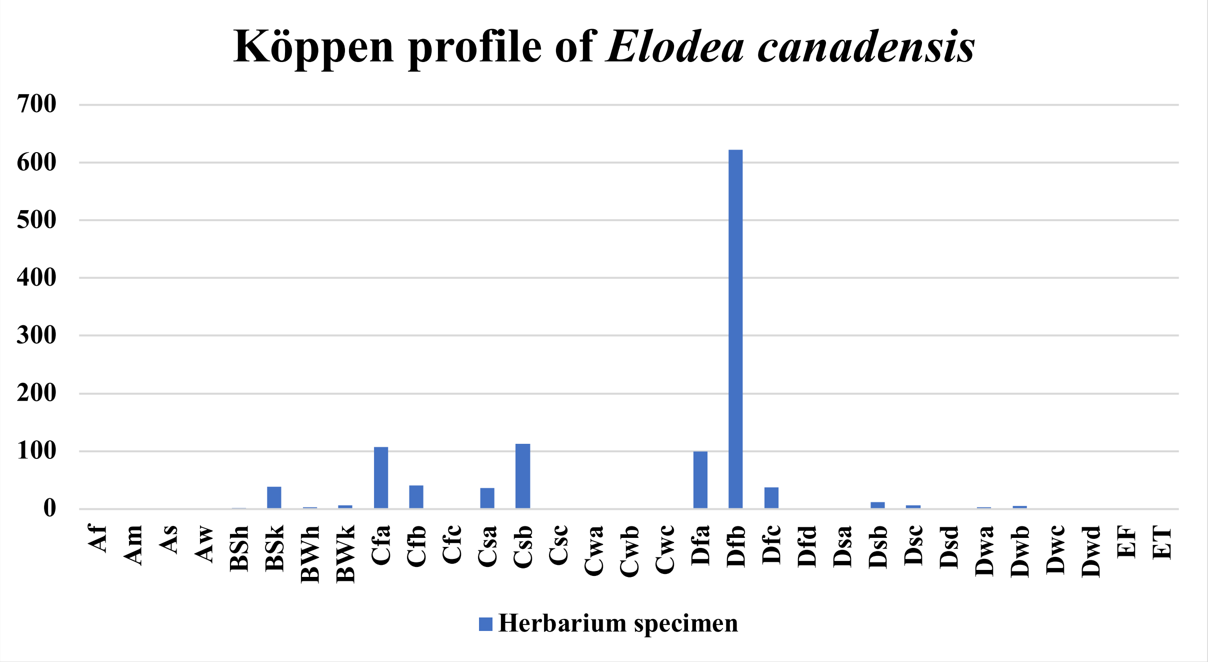

5.2.2.2.4. Köppen profile, distribution and climate map– GBIF occurrences of *Elodea granatensis* H.St.John 1962; herbarium specimens excluding duplicate occurrences (n = 148).

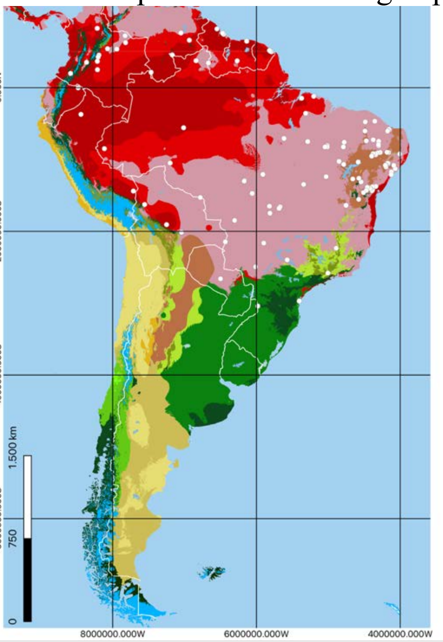

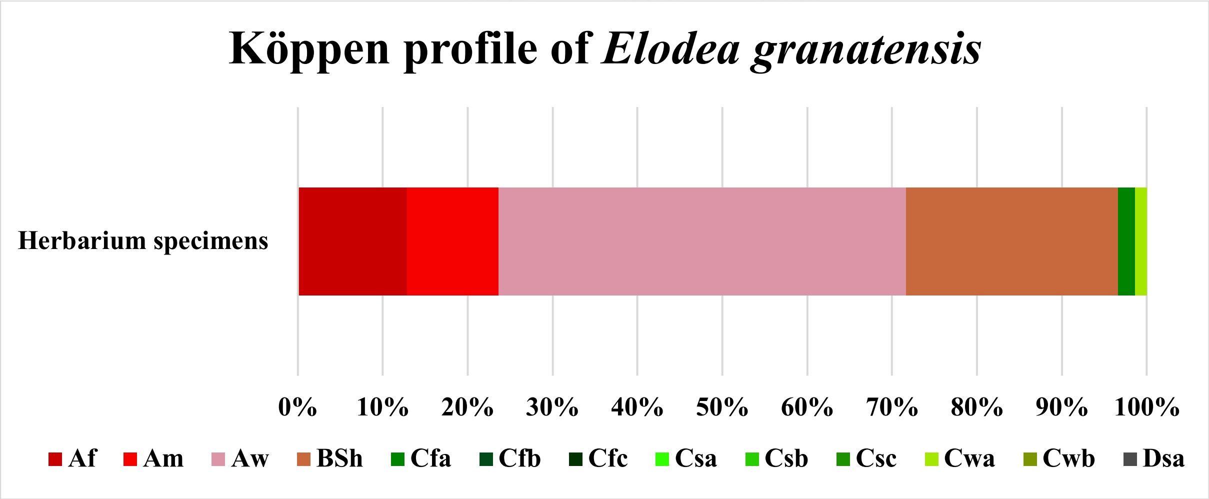

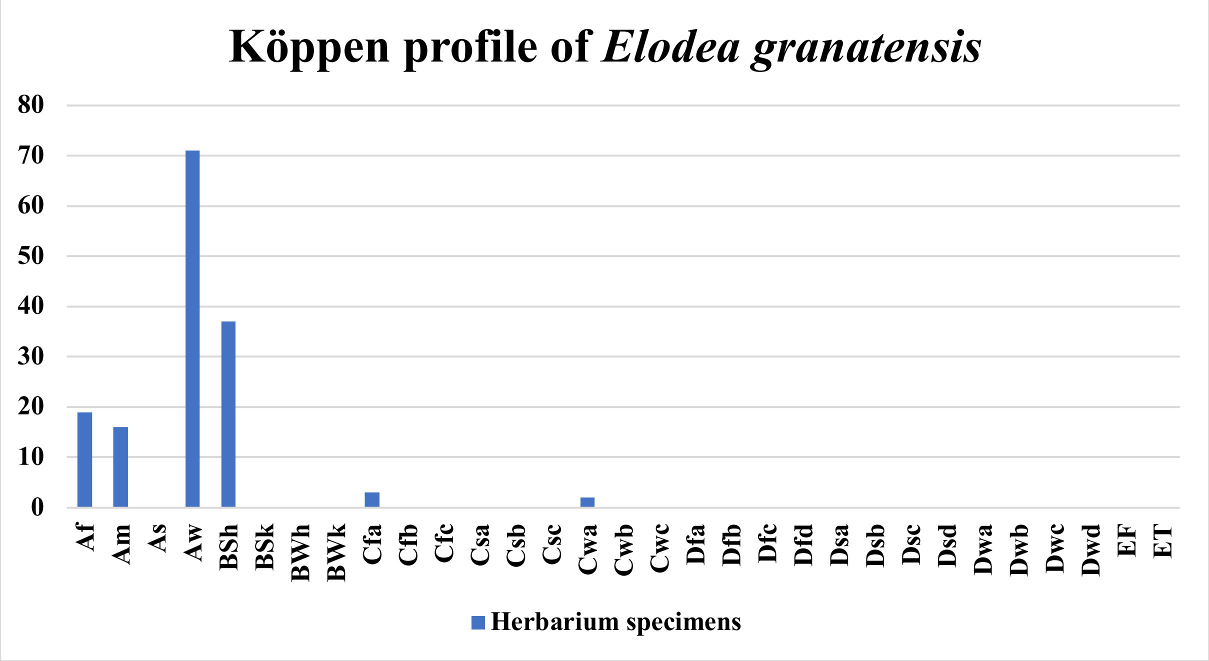

5.2.2.2.5. Köppen profile, distribution and climate map– GBIF occurrences of *Elodea nuttallii* H.St.John 1920; natural and neophyte distribution; herbarium specimen (n = 896).

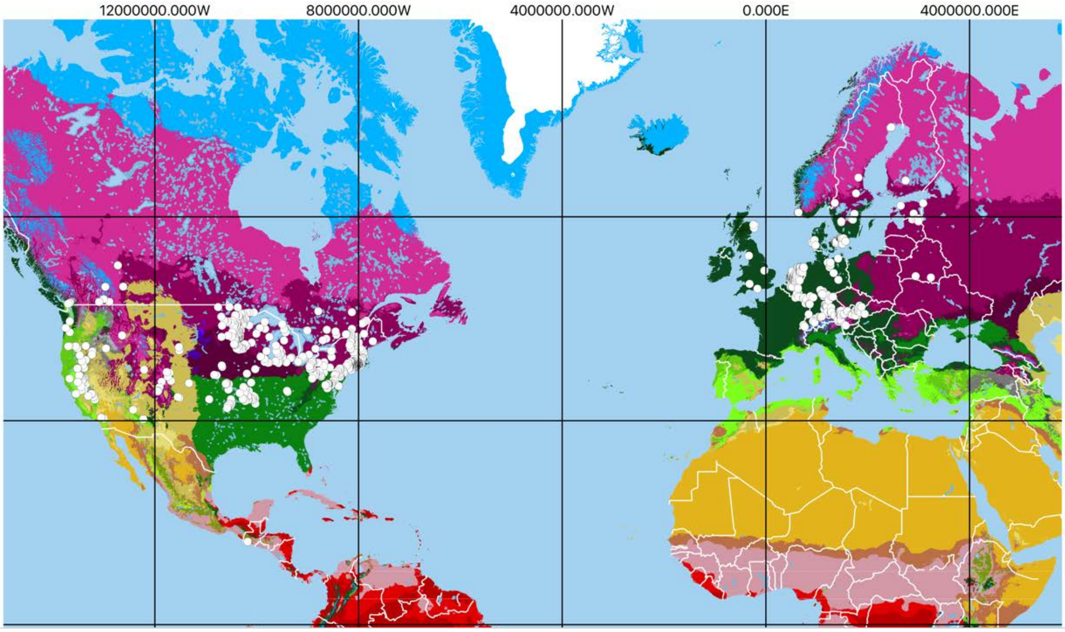

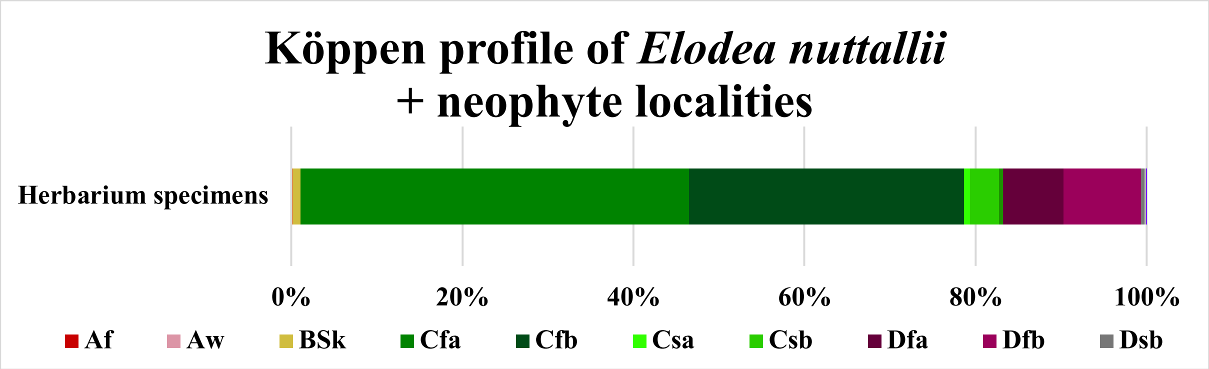

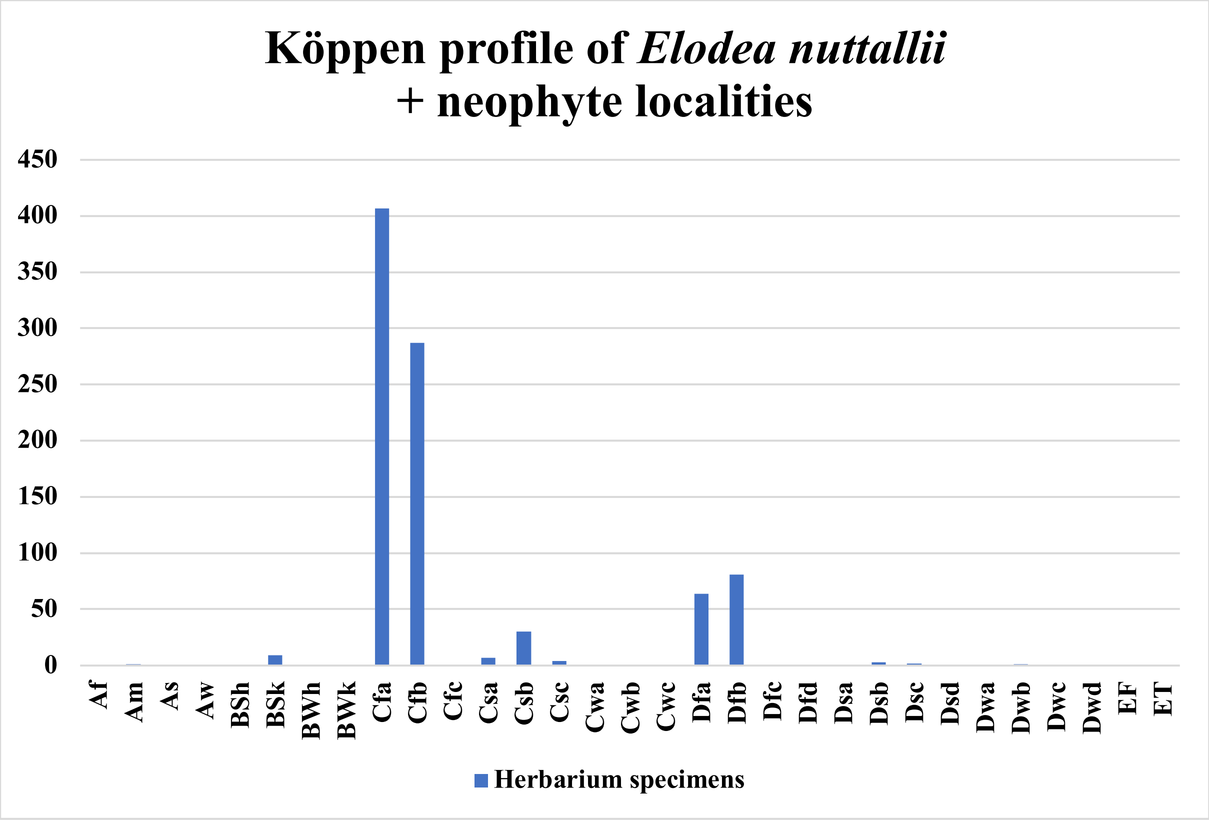

Köppen profile, distribution and climate map– GBIF occurrences of *Elodea nuttallii*; natural distribution; herbarium specimen (n = 488).

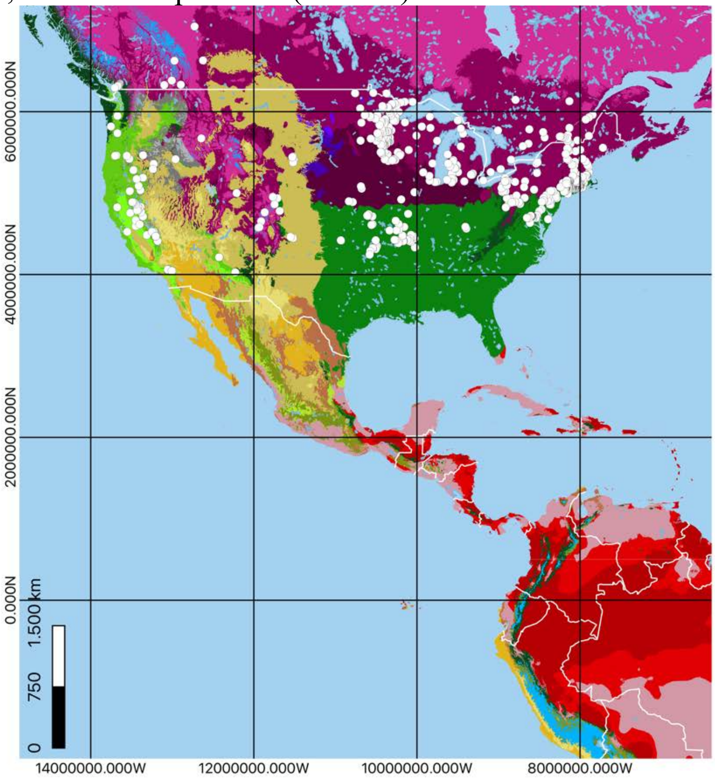

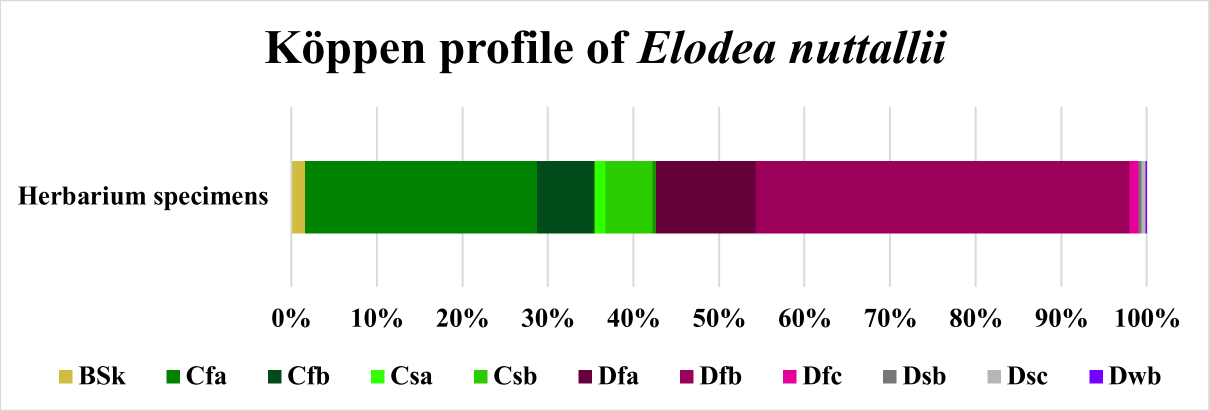

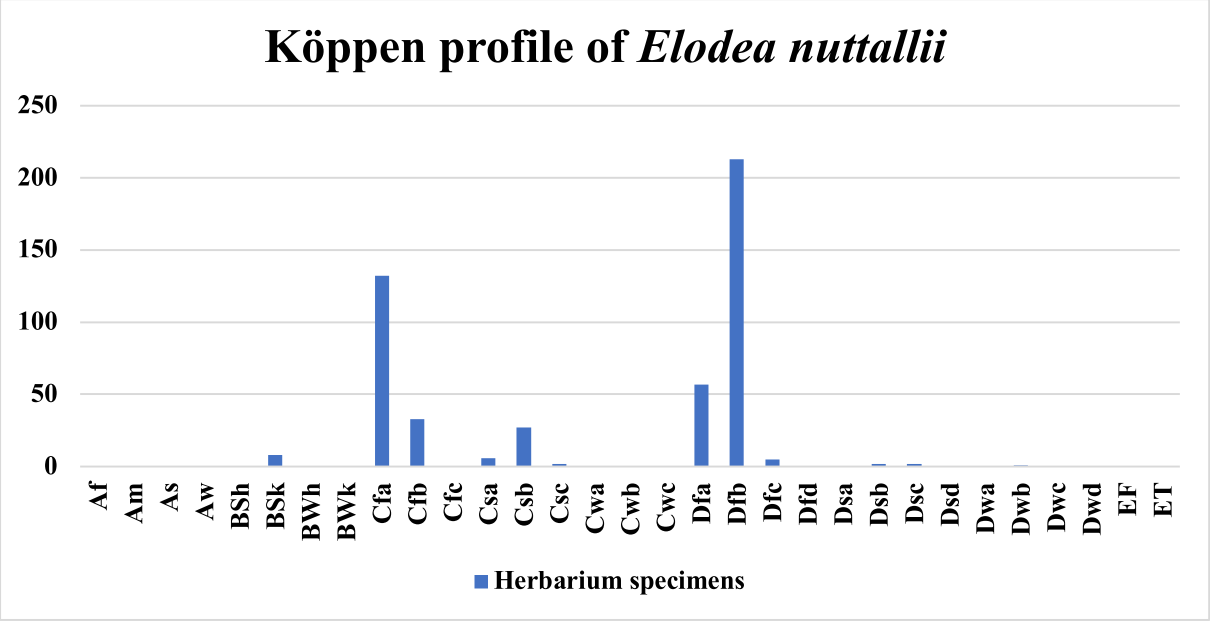

5.2.2.2.6. Köppen profile, distribution and climate map–GBIF occurrences of *Elodea potamogeton* Espinosa 1928; herbarium specimens excluding duplicate occurrences (n = 33).

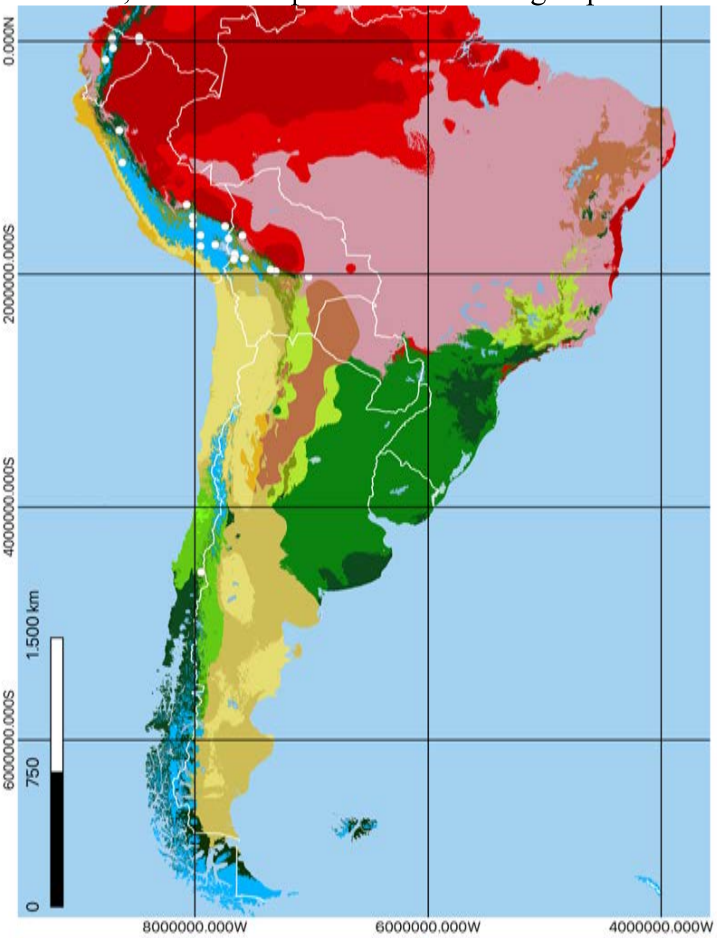

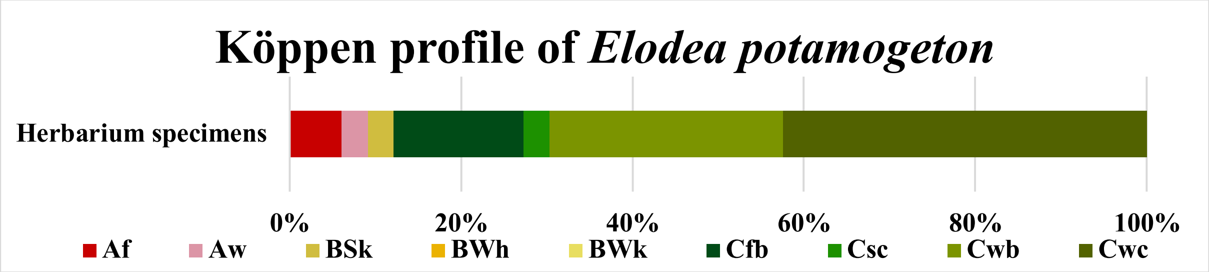

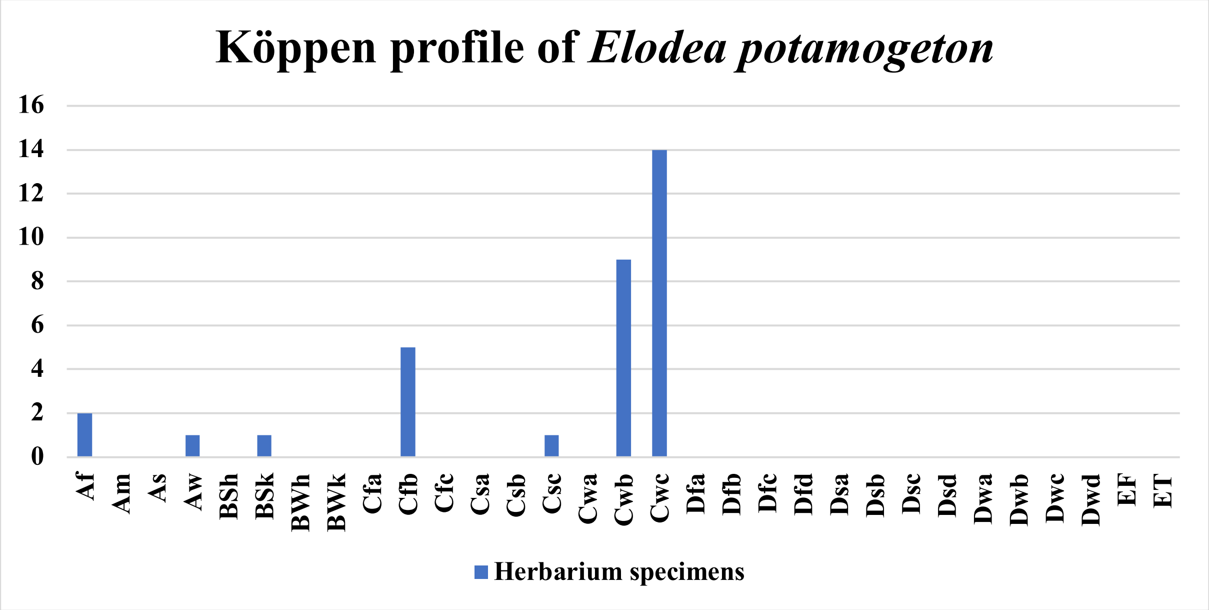

5.2.3. Crown group Clade B of Hydrocharitaceae cf. Chen et. al (2012), comprising *Blyxa* Noronha ex Thouars, 1806 and *Ottelia* Pers., 1805

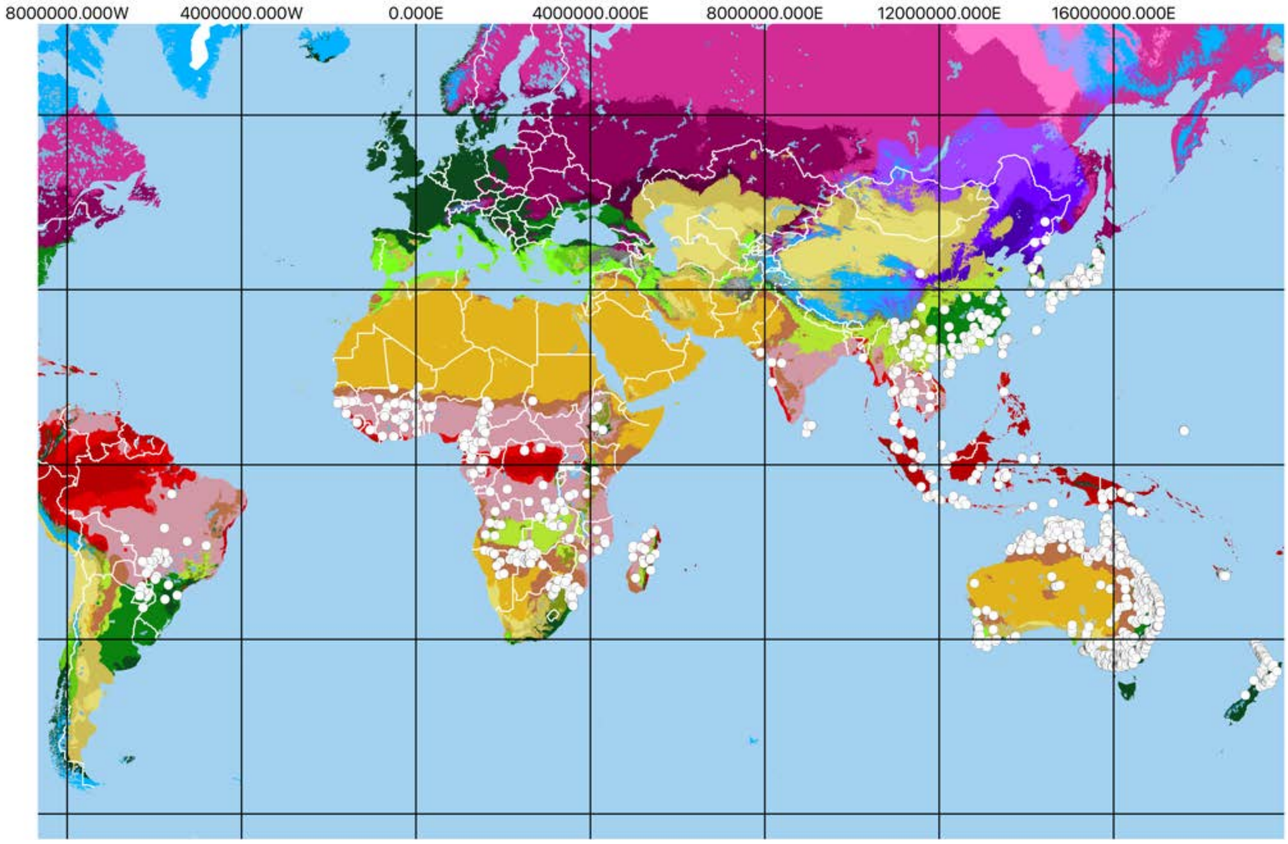

5.2.3.1 Genus *Blyxa* Noronha ex Thouars 1806.

Distribution and Köppen profiles for genus *Blyxa* incl. *B. auberti, B. echinosperma, B. hexandra, B. japonica, B. octandra, B. senegalensis, B. shimadai,* and *B. somai,* based on preserved specimen (n = 926).

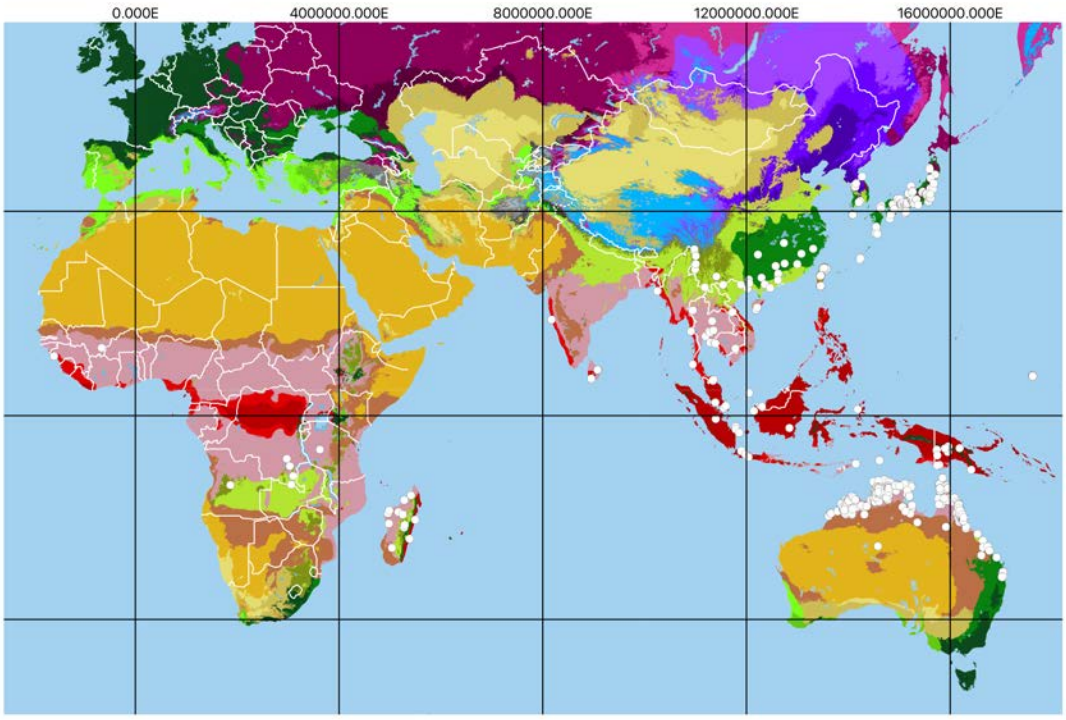

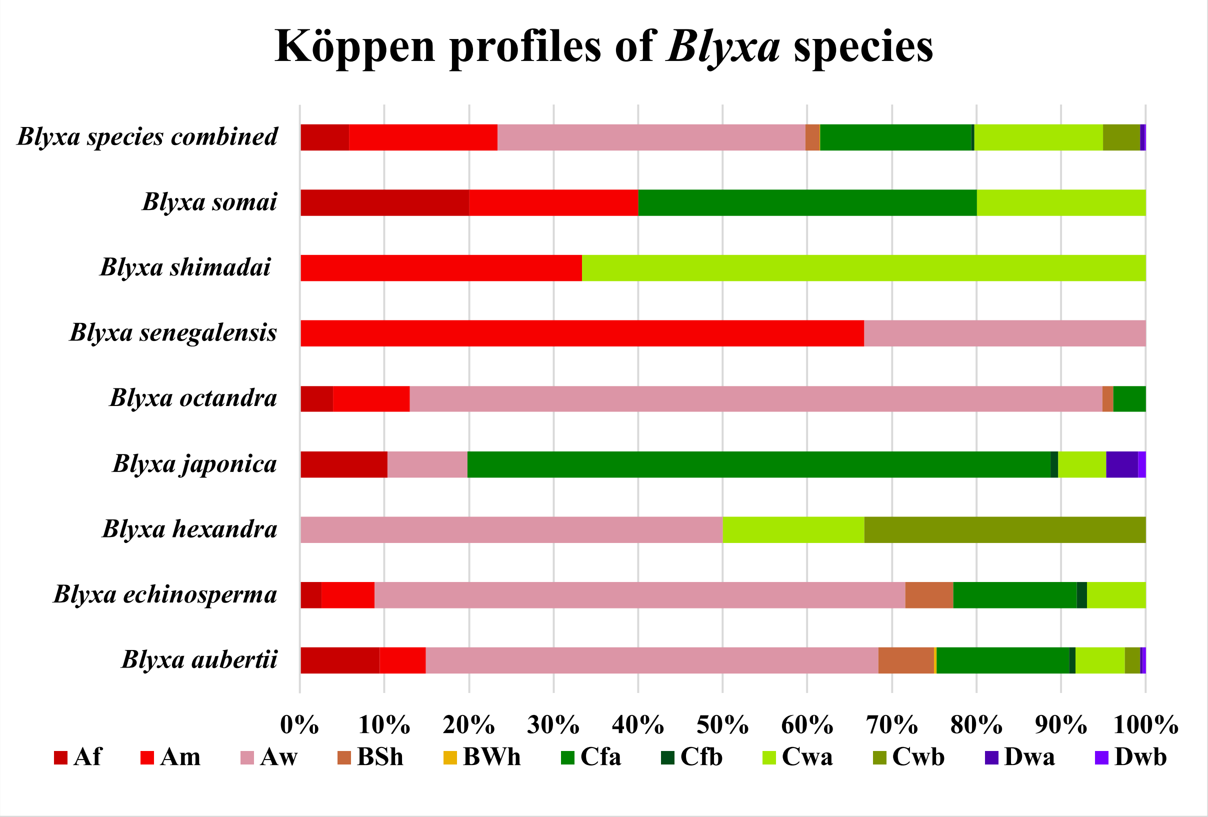

5.2.3.1.1. Köppen profile, distribution and climate map– GBIF occurrences of *Blyxa aubertii* Rich. 1814; herbarium specimens excluding duplicate occurrences (n = 275).

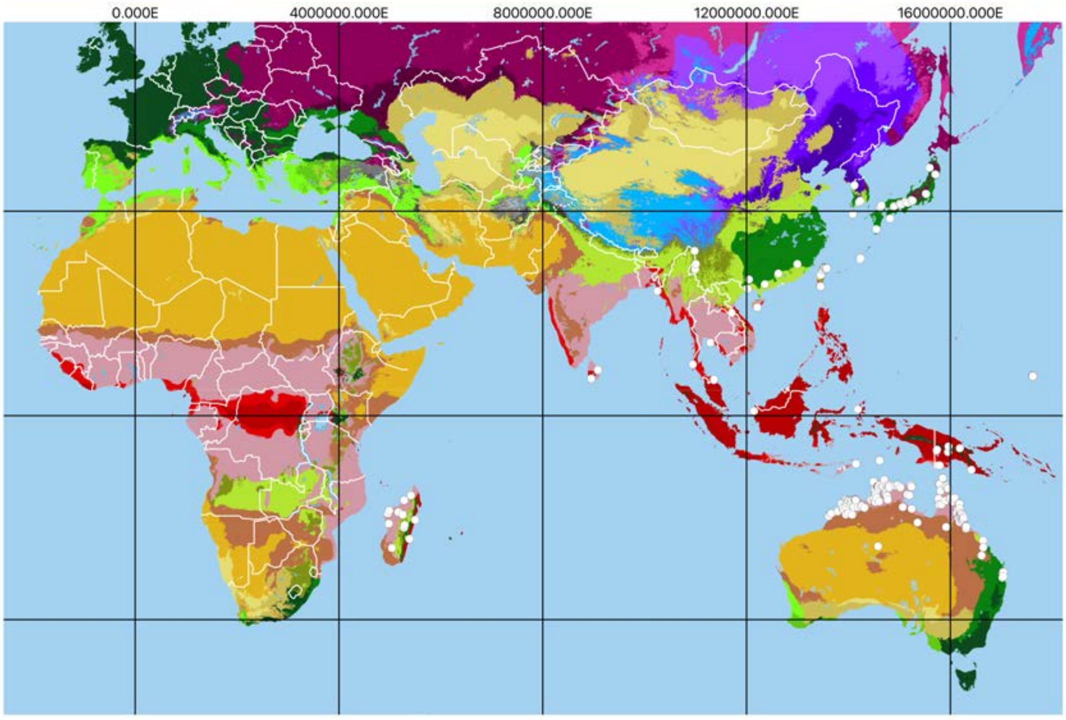

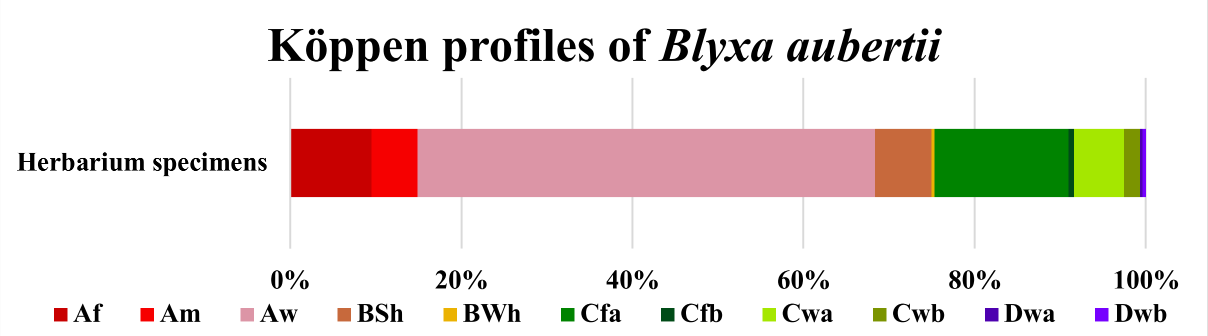

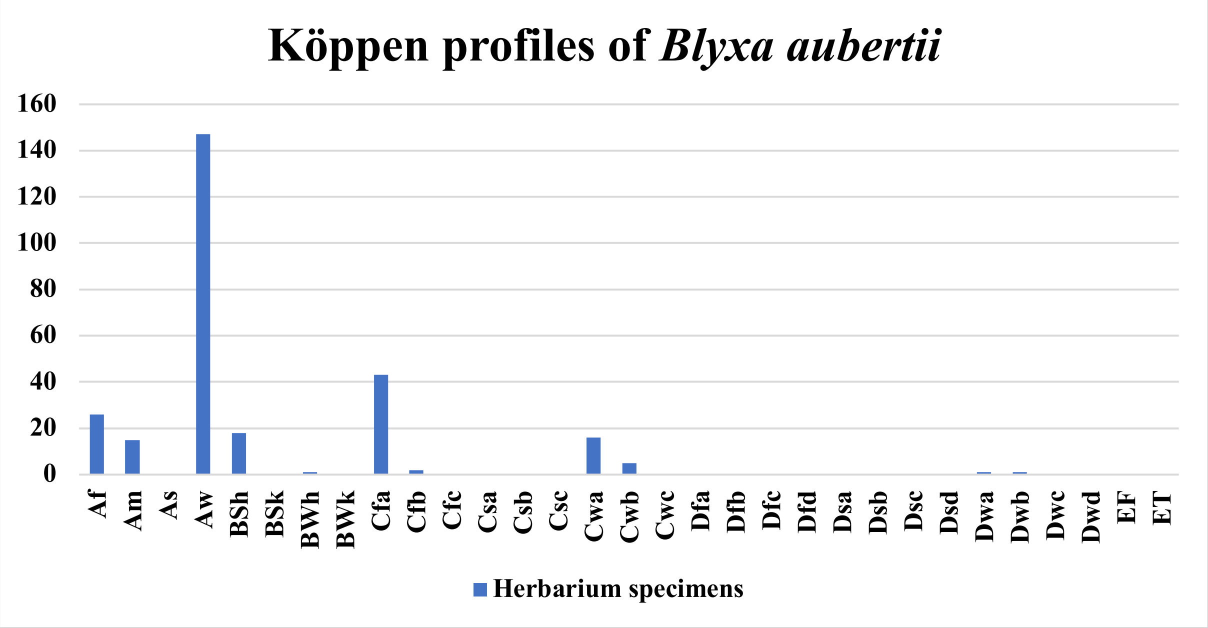

5.2.3.1.2. Köppen profile, distribution and climate map– GBIF occurrences of *Blyxa echinopserma* Hook f. 1888; herbarium specimens excluding duplicate occurrences (n = 158).

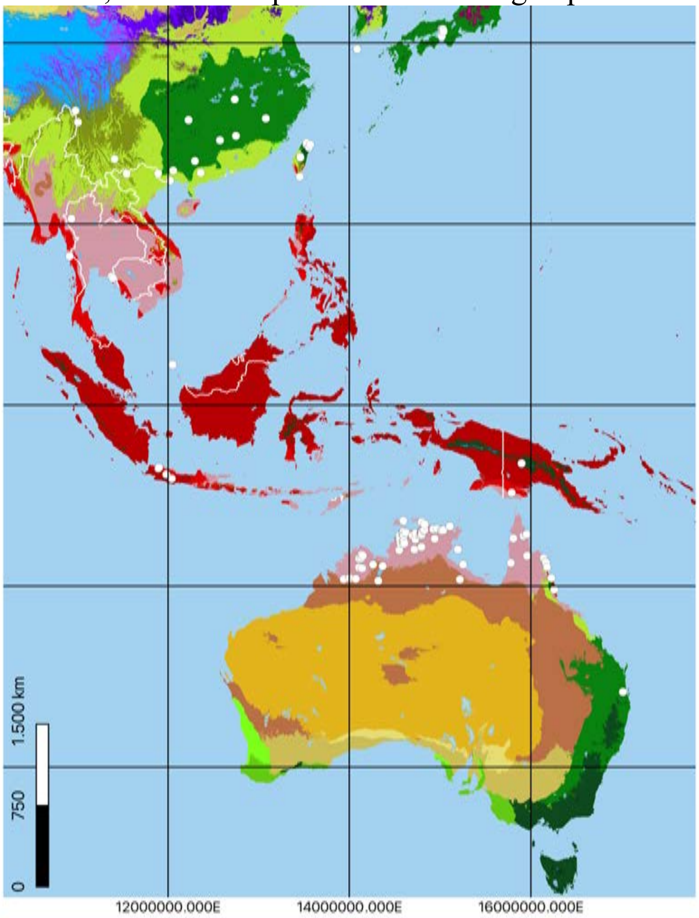

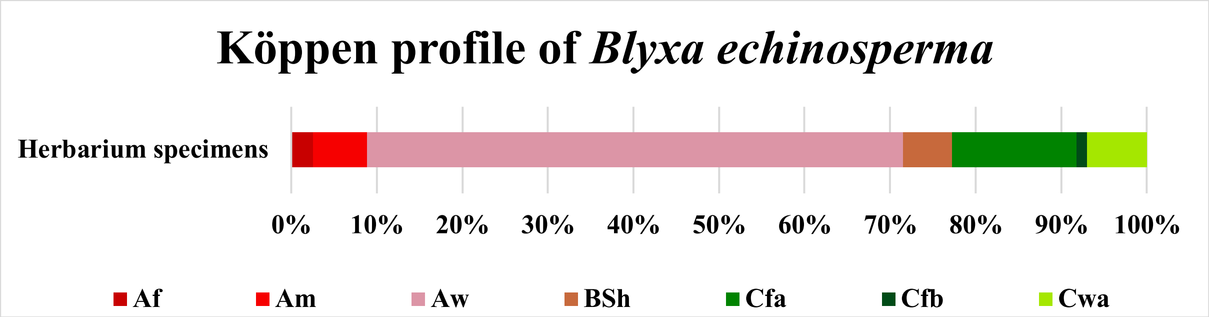

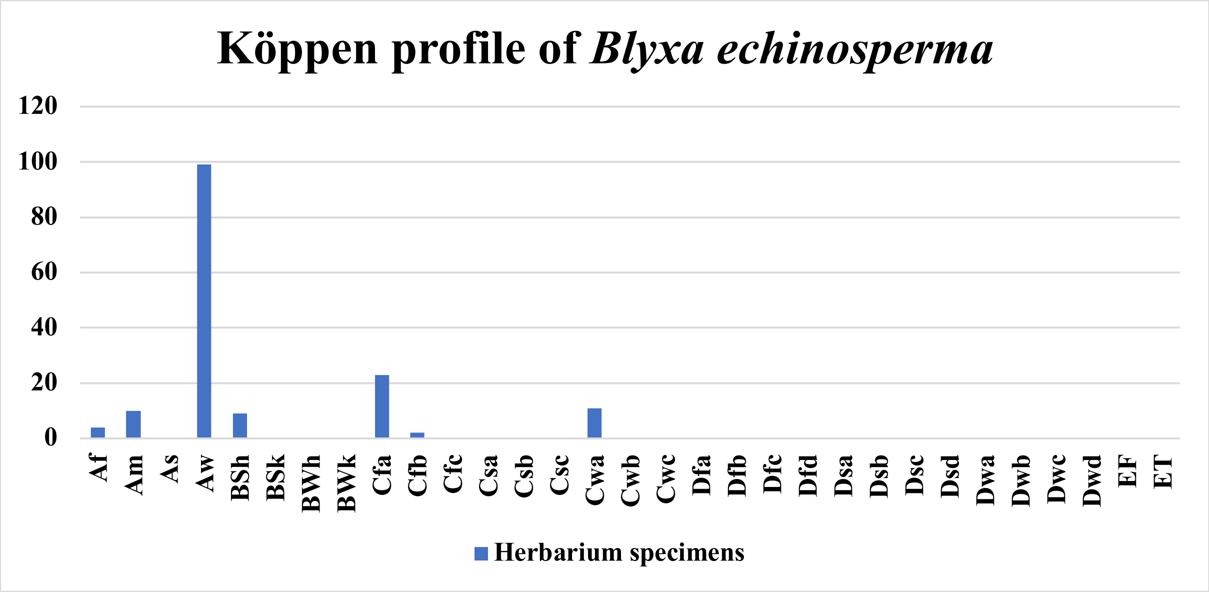

5.2.3.1.3. Köppen profile, distribution and climate map– GBIF occurrences of *Blyxa hexandra* C.D.K.Cook & Luond 1980; herbarium specimens excluding duplicate occurrences (n = 6).

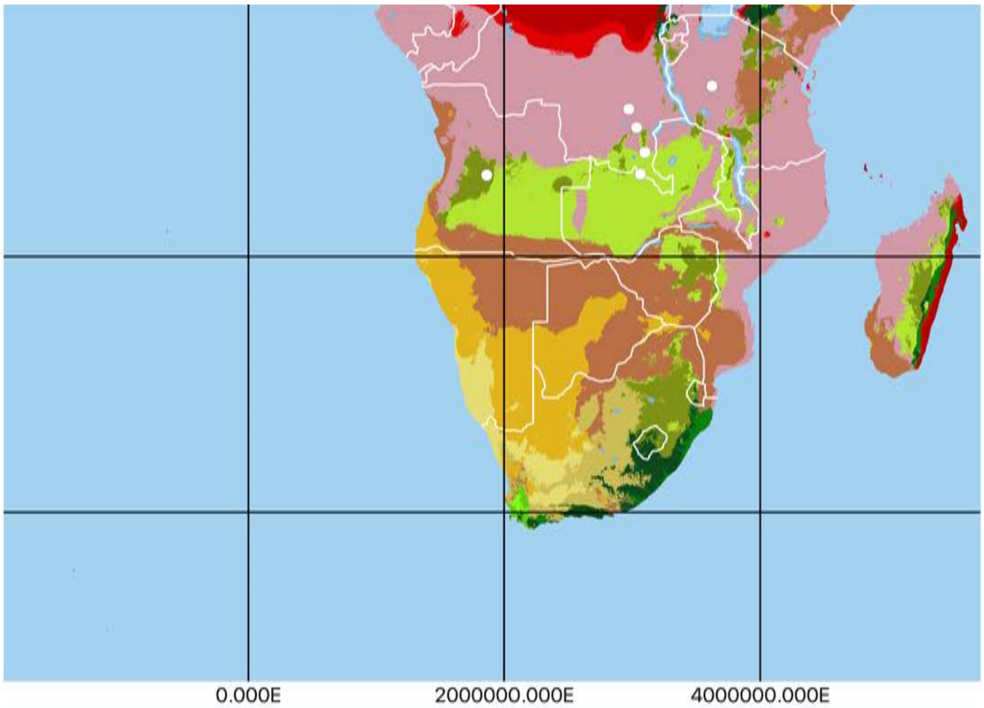

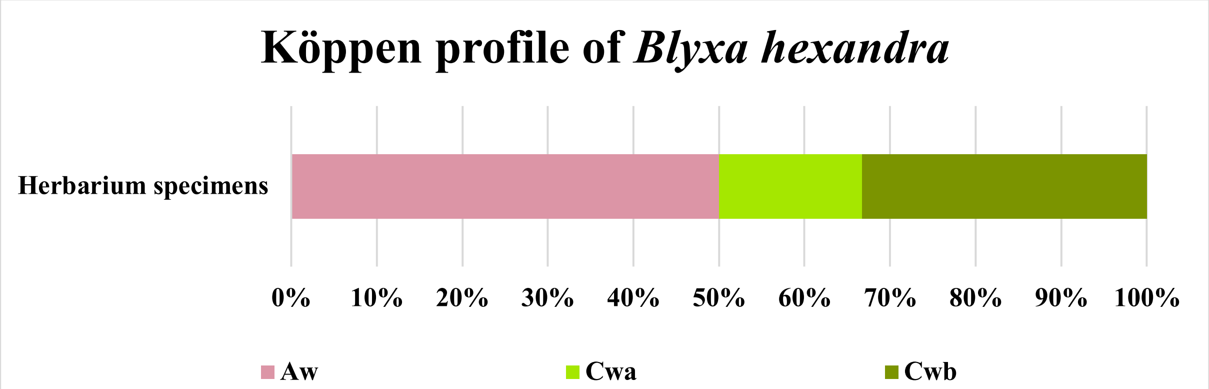

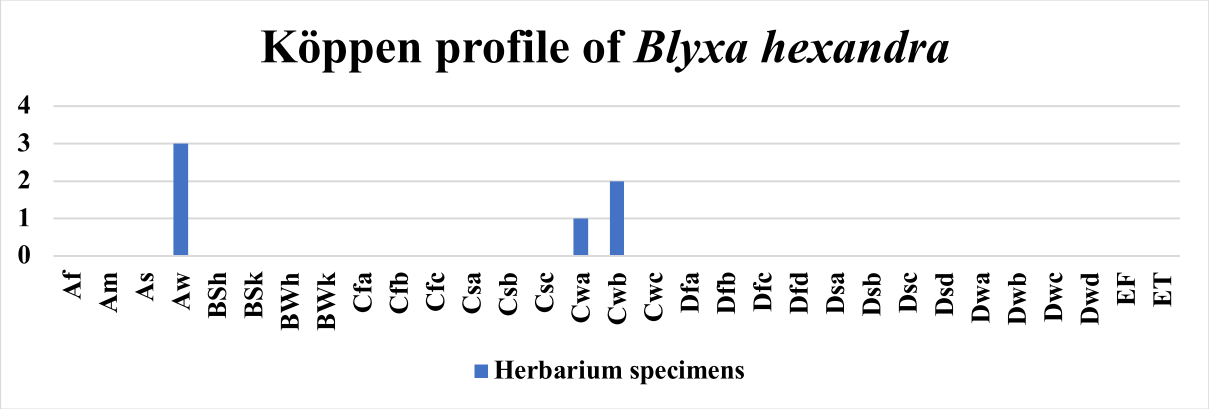

5.2.3.1.4. Köppen profile, distribution and climate map– GBIF occurrences of *Blyxa japonica* (Miq. 1866) Maxim. ex Asch & Gürke 1889; herbarium specimens excluding duplicate occurrences (n = 106).

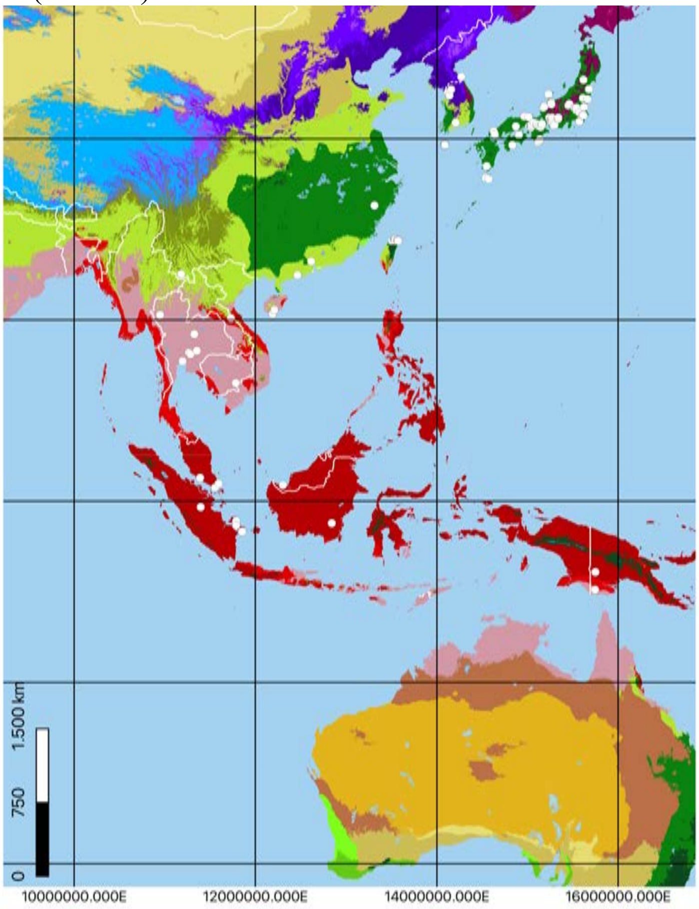

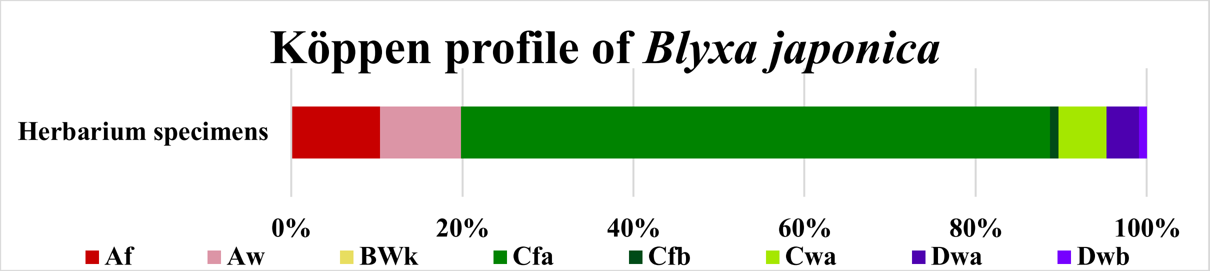

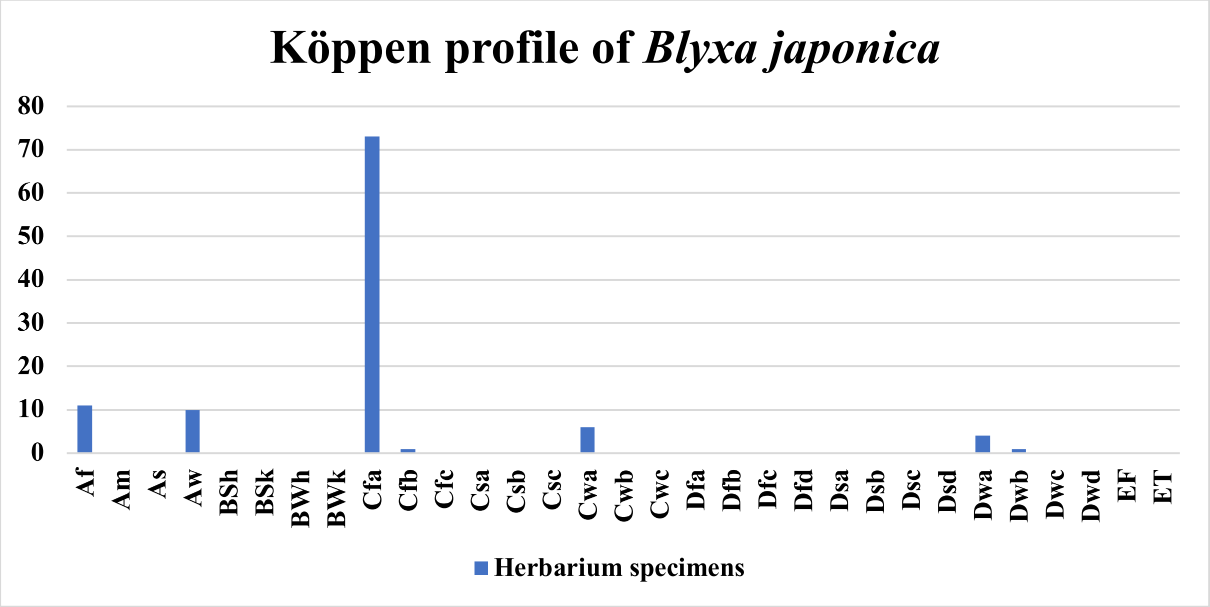

5.2.3.1.5. Köppen profile, distribution and climate map– GBIF occurrences of *Blyxa octandra* (Roxb. 1802) Planch. ex Thwaites 1864; herbarium specimens excluding duplicate occurrences (n = 77).

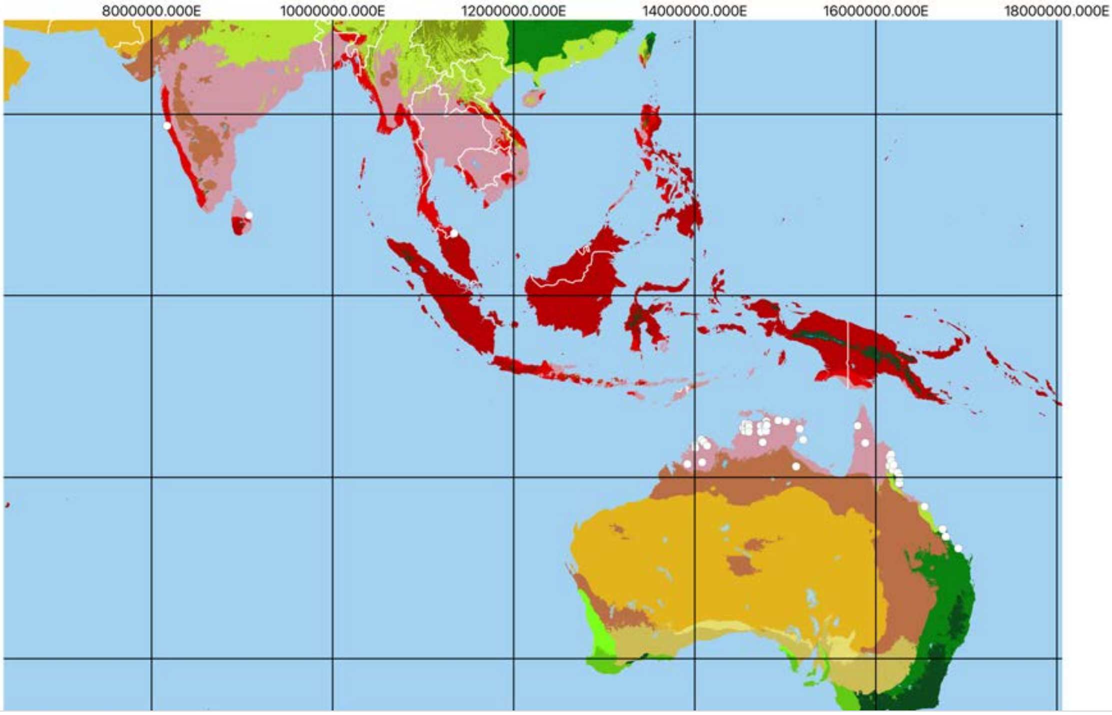

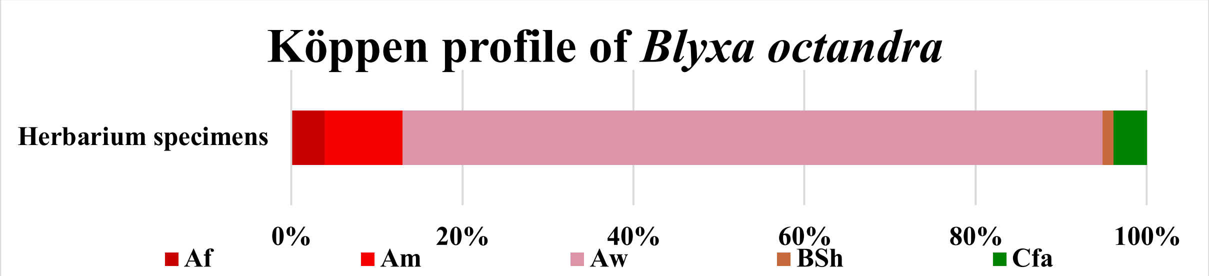

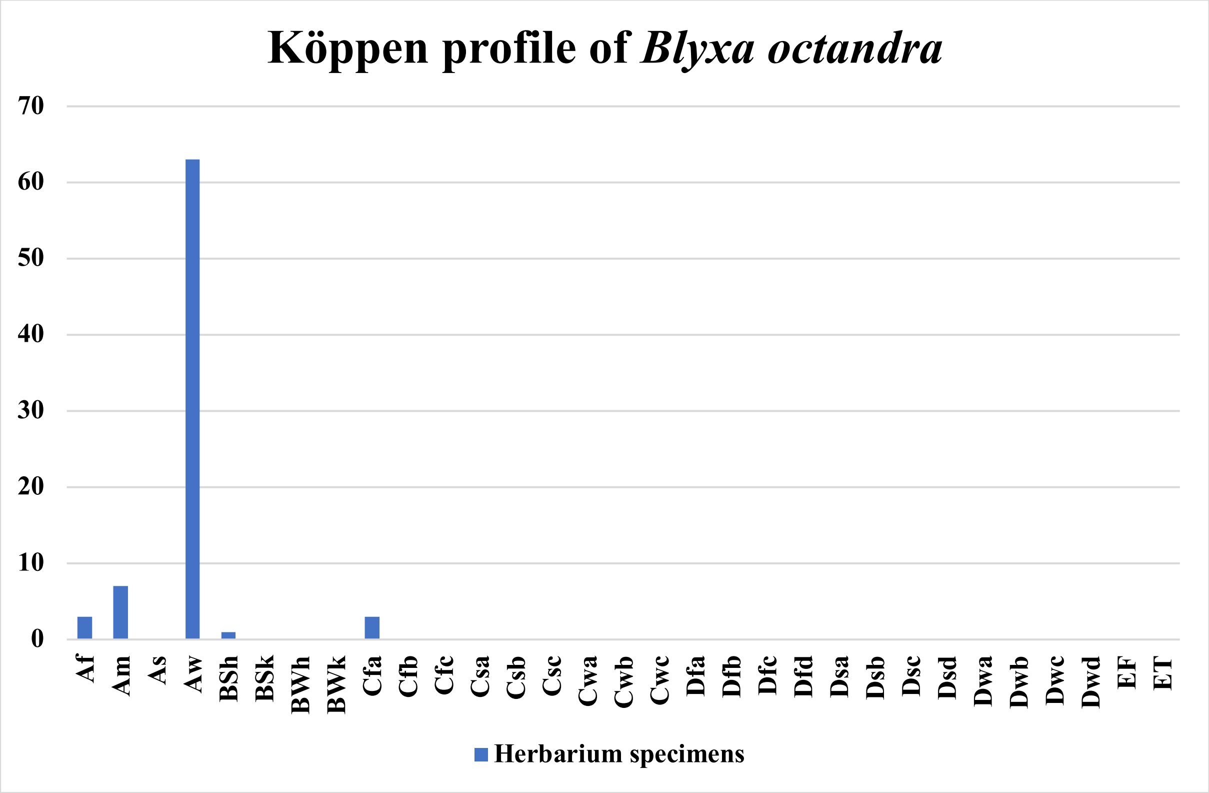

5.2.3.1.6. Köppen profile, distribution and climate map– GBIF occurrences of *Blyxa senegalensis* Dandy 1934; herbarium specimens excluding duplicate occurrences (n = 3).

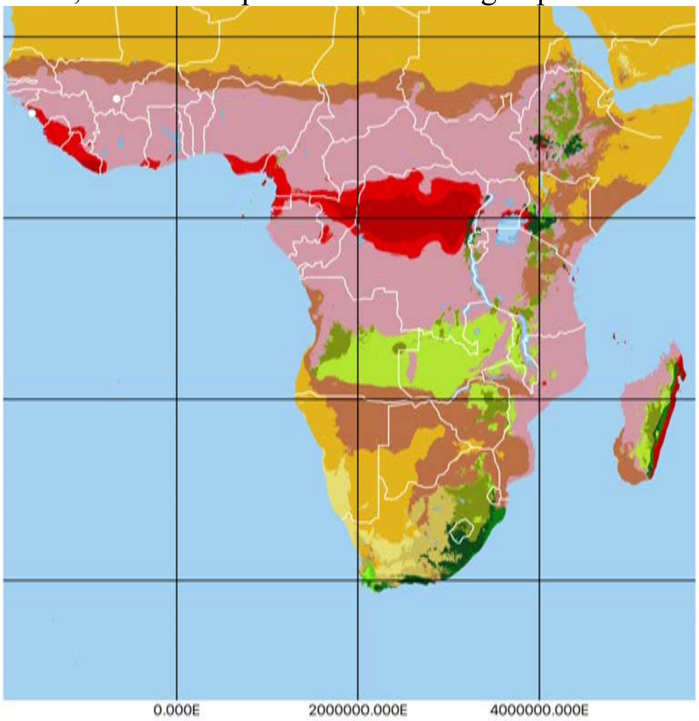

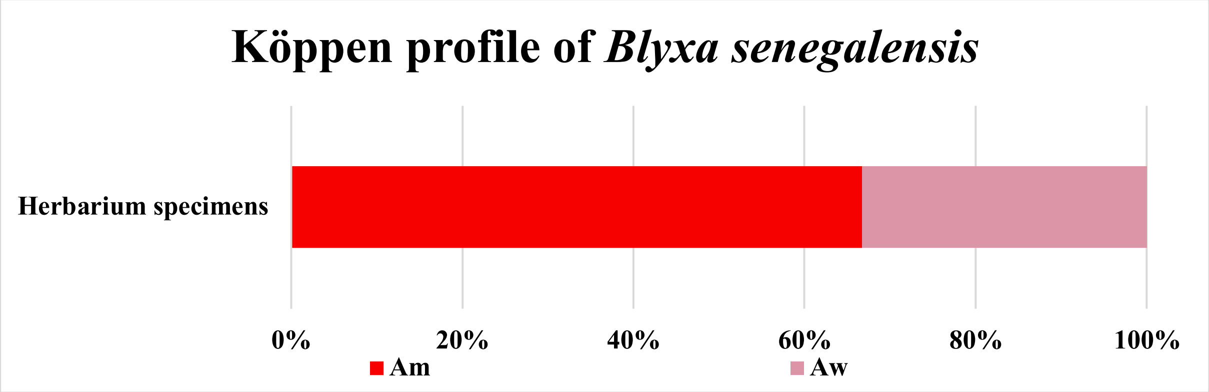

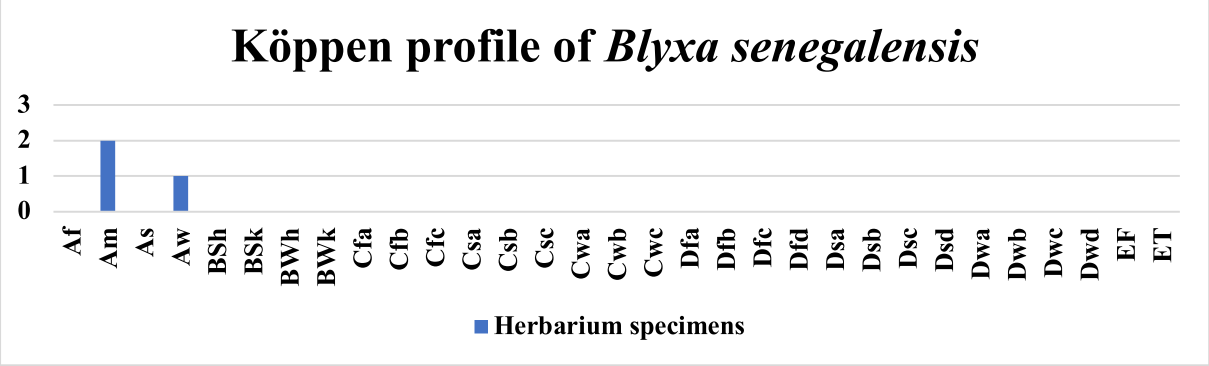

5.2.3.1.7. Köppen profile, distribution and climate map– GBIF occurrences of *Blyxa somai* Hayata 1915; herbarium specimens excluding duplicate occurrences (n = 5).

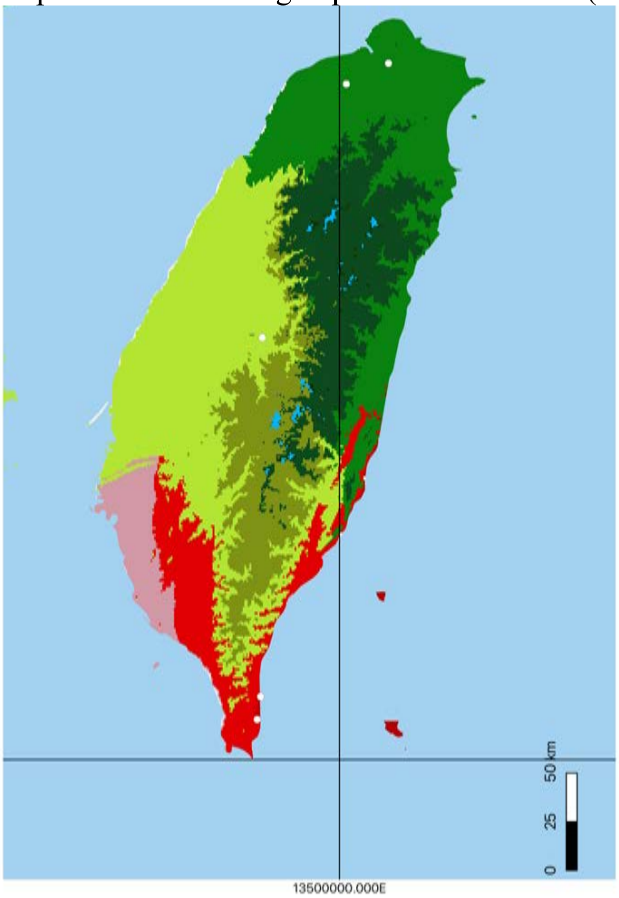

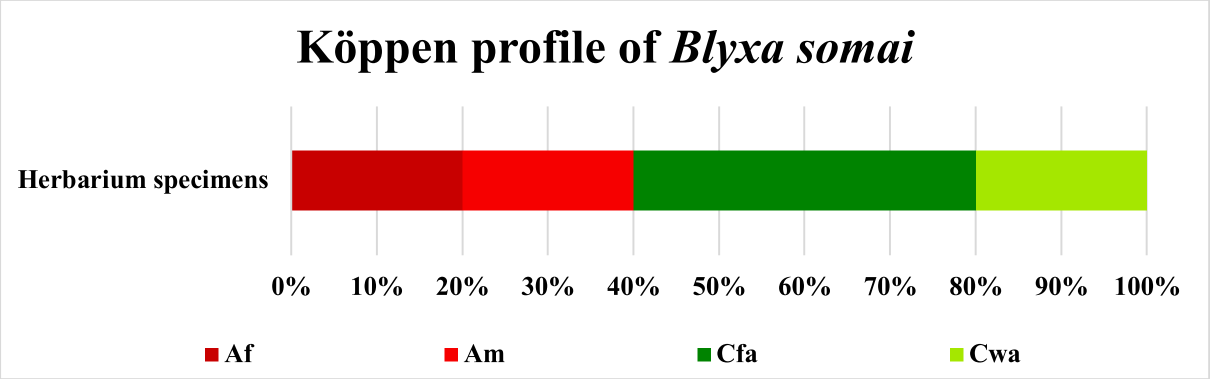

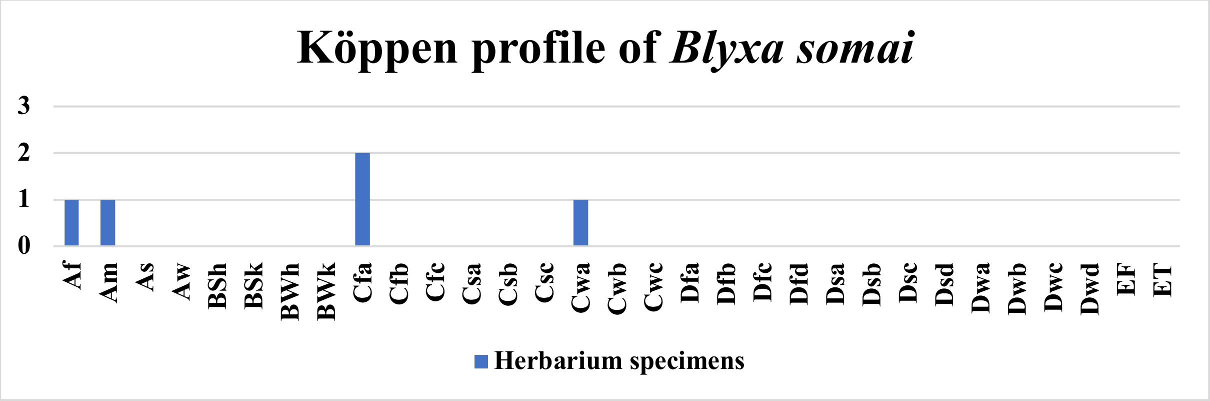

5.2.3.1.8. Köppen profile, distribution and climate map– GBIF occurrences of *Blyxa shimadai* Hayata 1915; herbarium specimens excluding duplicate occurrences (n = 13).

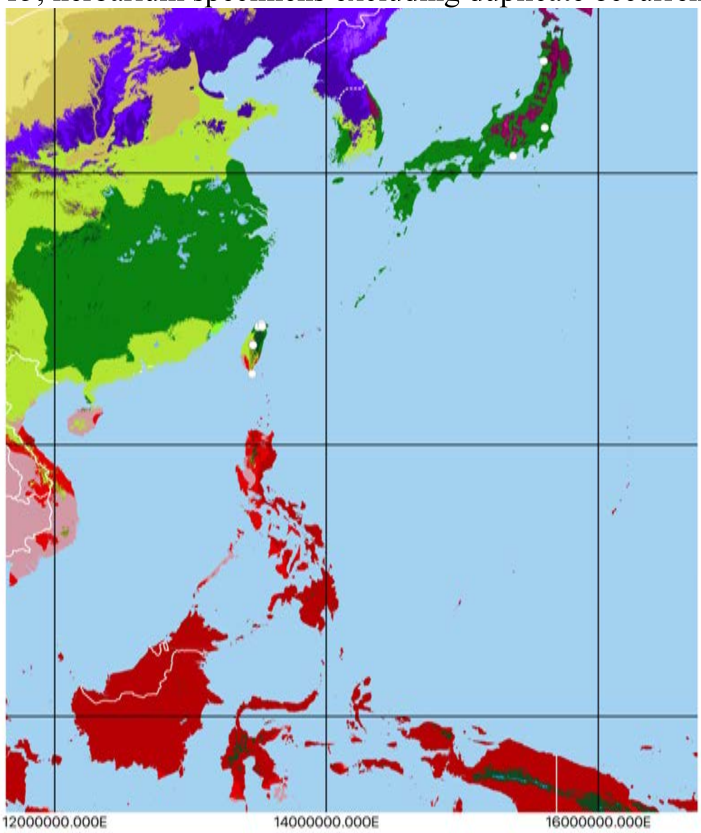

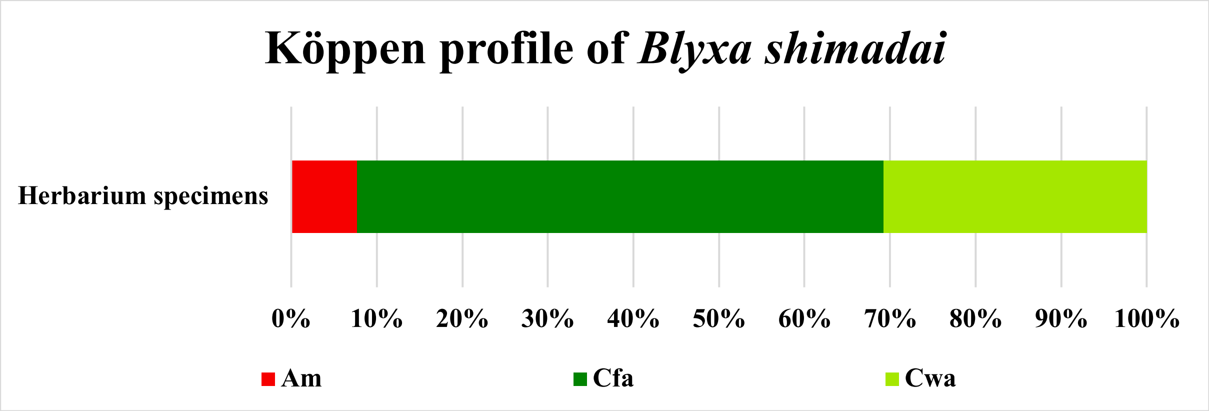

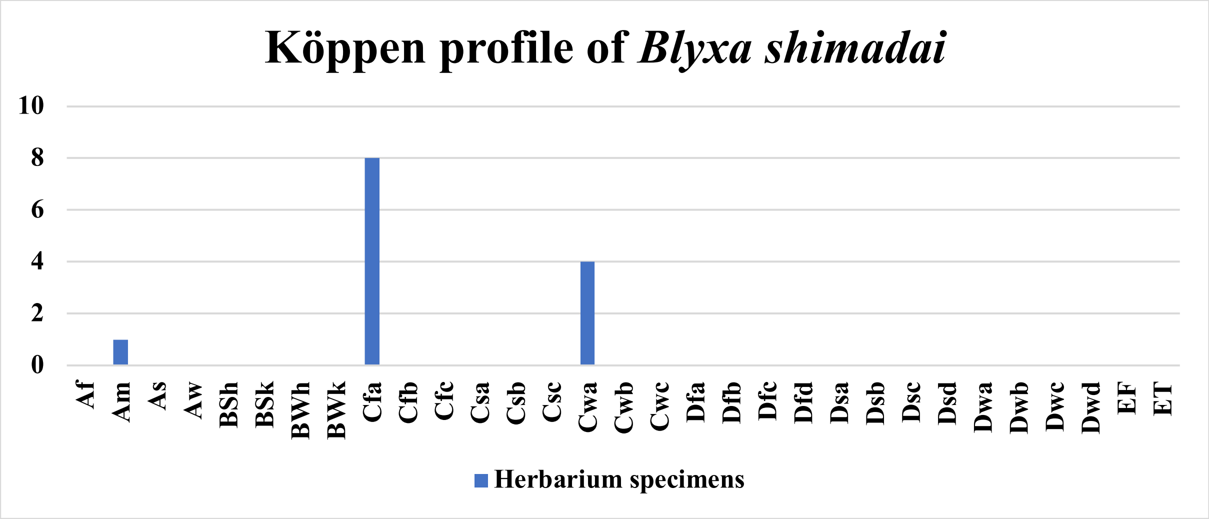

5.2.3.2. Genus *Ottelia* Pers. 1805.

Distribution and Köppen profiles of genus *Ottelia* incl. *Ottelia acuminata*, *O. alismoides*, *O. balanesnsae, O. brasiliensis*, *O. cordata, O. exserta, O. kunenensis, O. mesenterium, O. muricata, O. ovalifolia, O. ulvifolia,* and *O. verdickii,* based on preserved specimen (n = 1670).

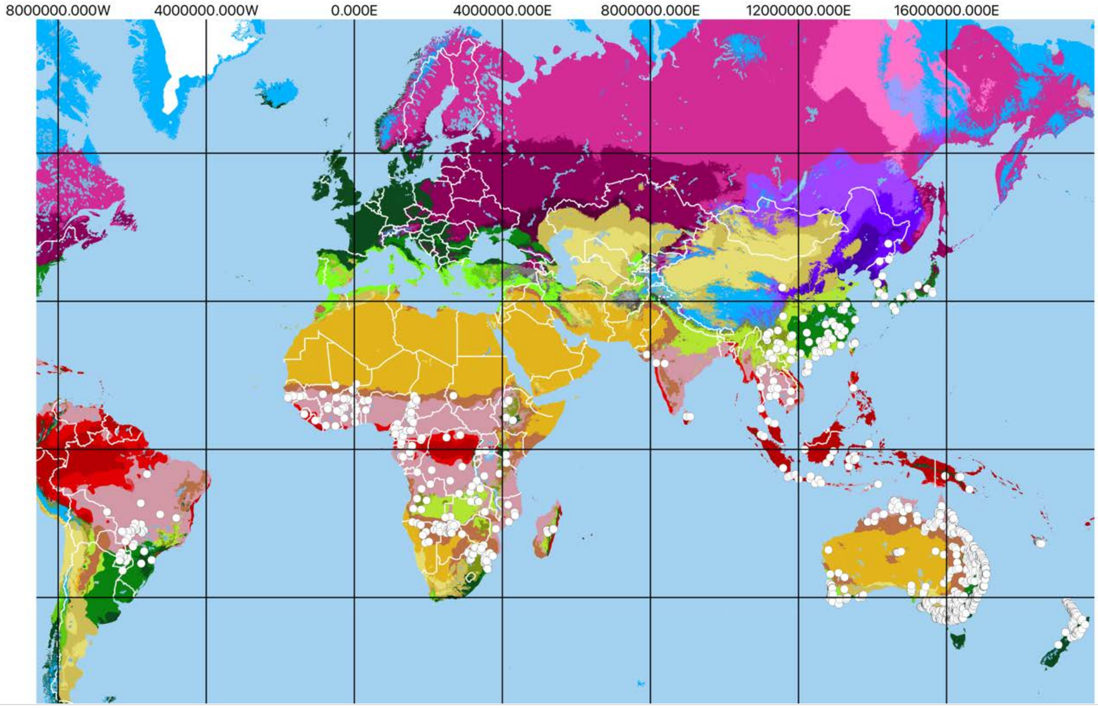

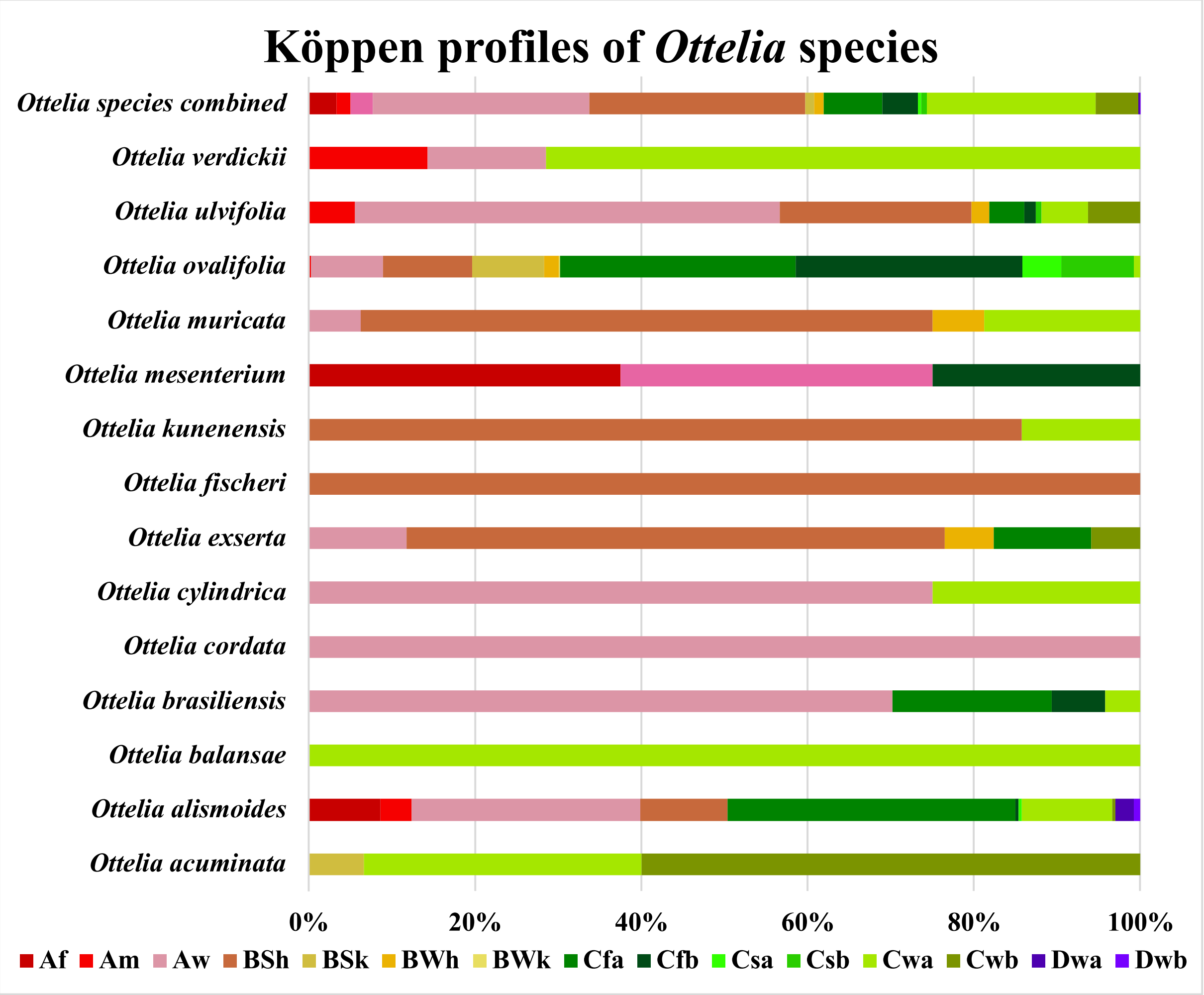

5.2.3.2.1. Köppen profile, distribution and climate map– GBIF occurrences of *Ottelia acuminata* (Gagnep. 1907) Dandy 1934; herbarium specimens excluding duplicate occurrences (n = 15).

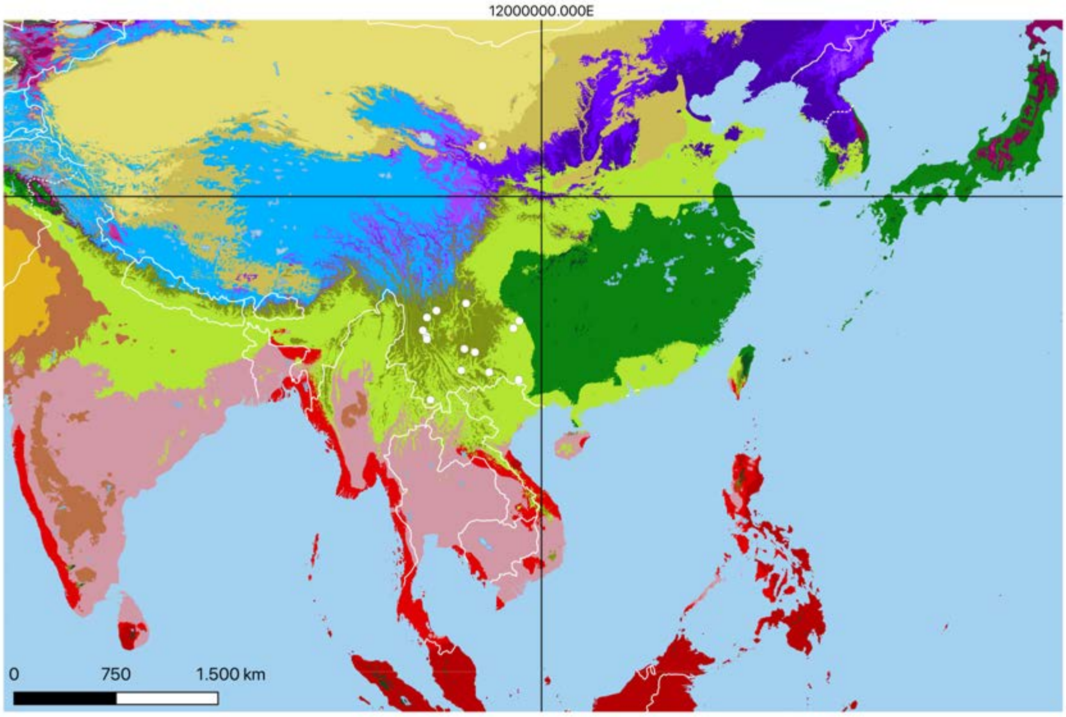

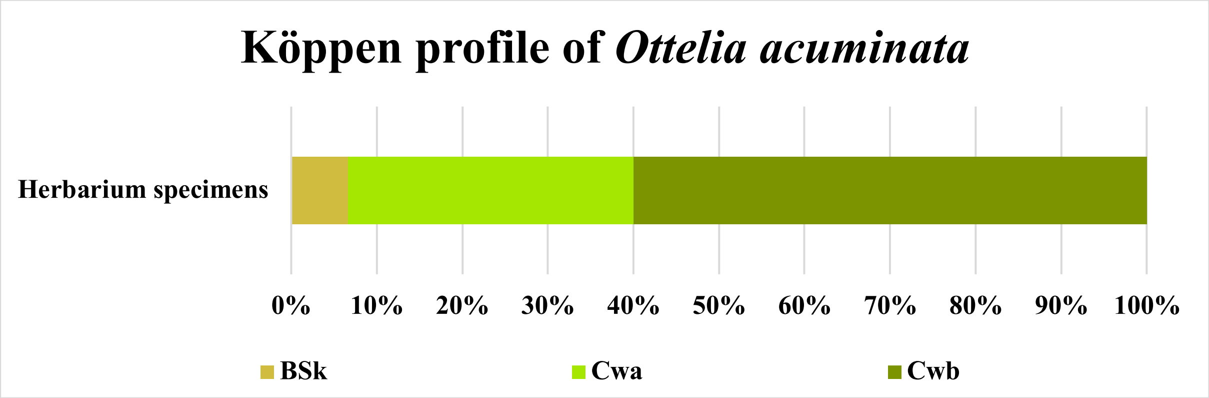

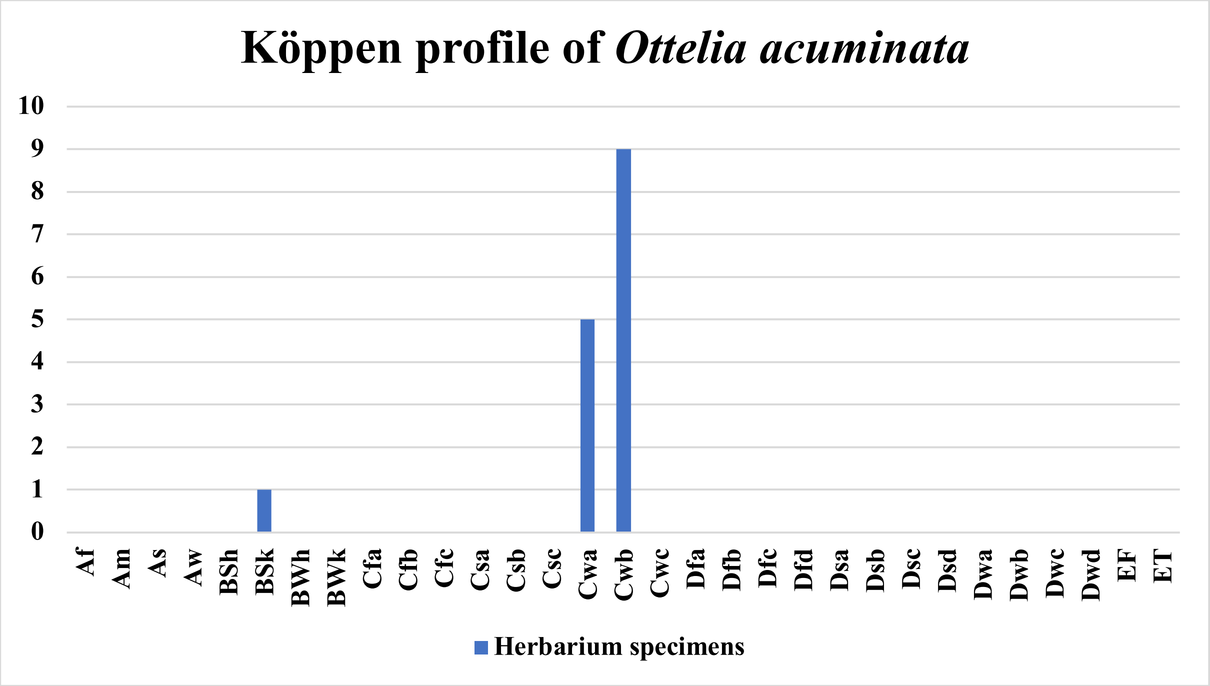
Köppen profile of *Ottelia acuminata*
10
9
8
7
6
5
4
3
2
1
0
Af
Am
As
Aw
BSh
BSk
BWh
BWk
Cfa
Cfb
Cfc
Csa
Csb
Csc
Cwa
Cwb
Cwc
Dfa
Dfb
Dfc
Dfd
Dsa
Dsb
Dsc
Dsd
Dwa
Dwb
Dwc
Dwd
EF
ET
Herbarium specimens

5.2.3.2.2. Köppen profile, distribution and climate map – GBIF occurrences of *Ottelia alismoides* (L. 1753) Pers. 1805; herbarium specimens excluding duplicate occurrences (n = 266).

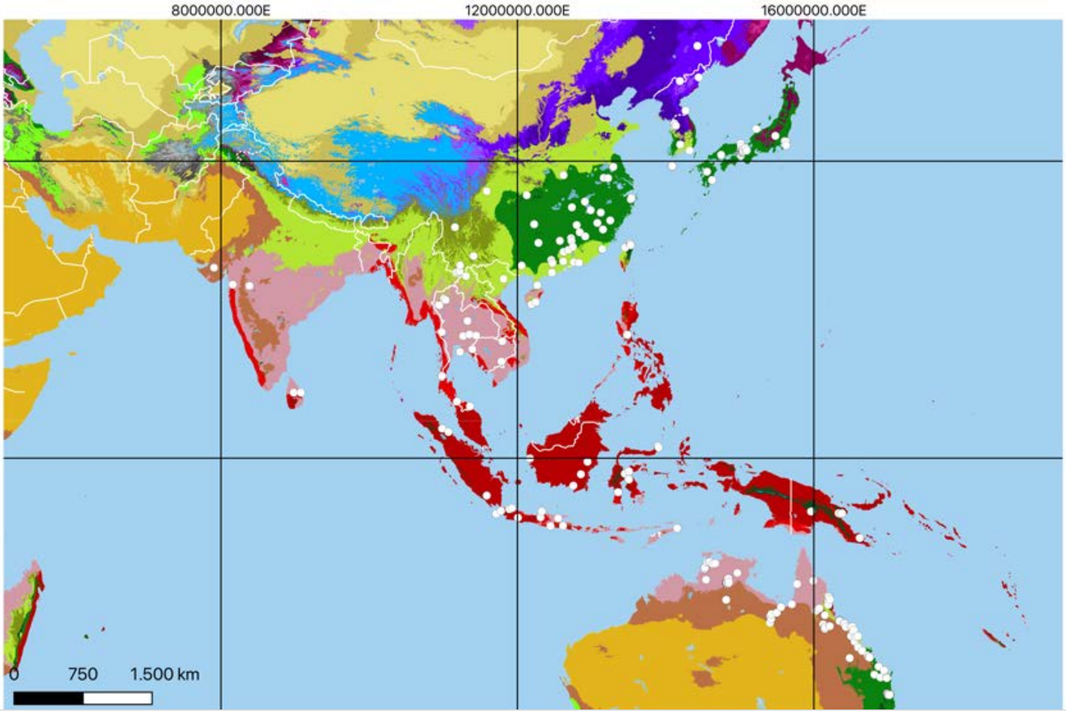

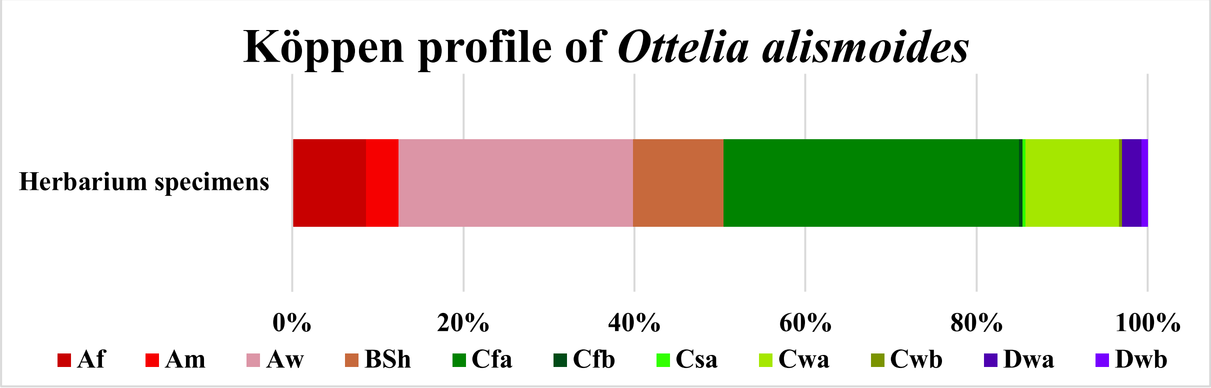

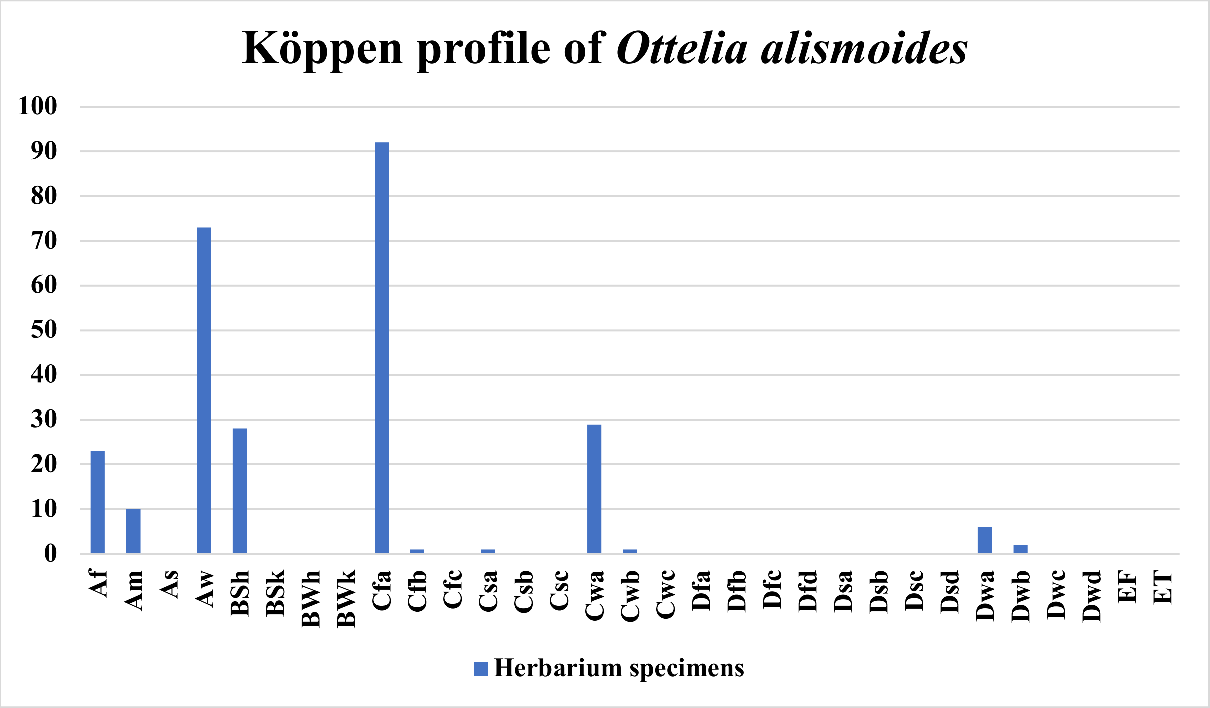
Köppen profile of *Ottelia alismoides*
100
90
80
70
60
50
40
30
20
10
0
Af
Am
As
Aw
BSh
BSk
BWh
BWk
Cfa
Cfb
Cfc
Csa
Csb
Csc
Cwa
Cwb
Cwc
Dfa
Dfb
Dfc
Dfd
Dsa
Dsb
Dsc
Dsd
Dwa
Dwb
Dwc
Dwd
EF
ET
Herbarium specimens

5.2.3.2.3. Köppen profile, distribution and climate map – GBIF occurrences of *Ottelia balansae* (Gagnep. 1907) Dandy 1934; herbarium specimens excluding duplicate occurrences (n = 1).

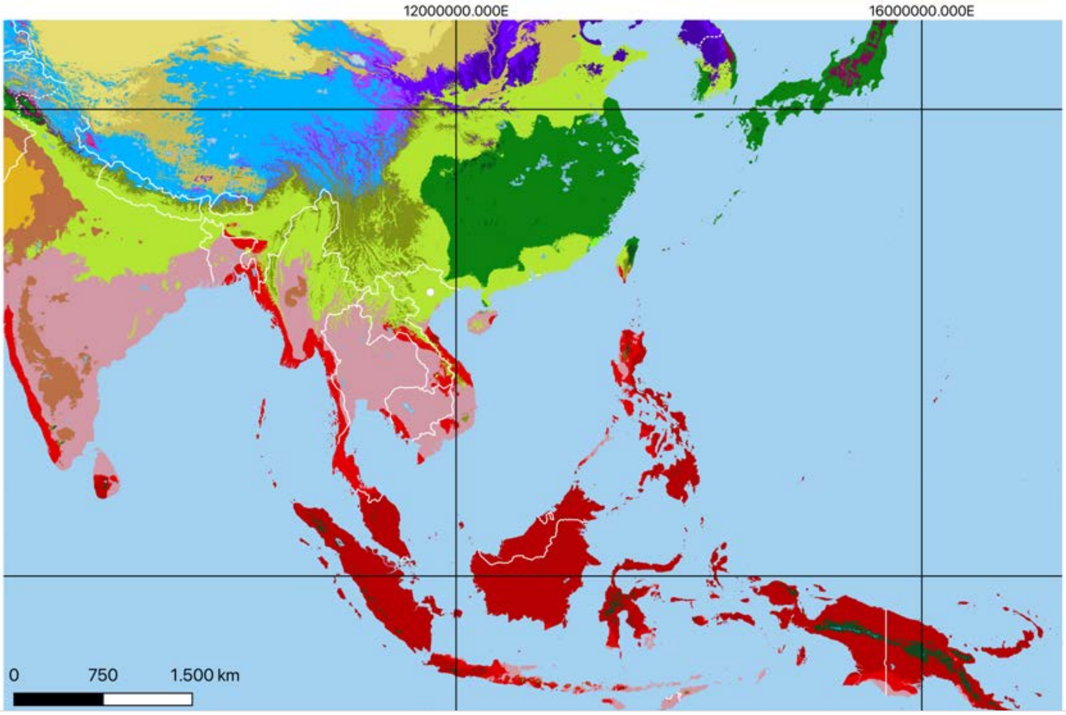

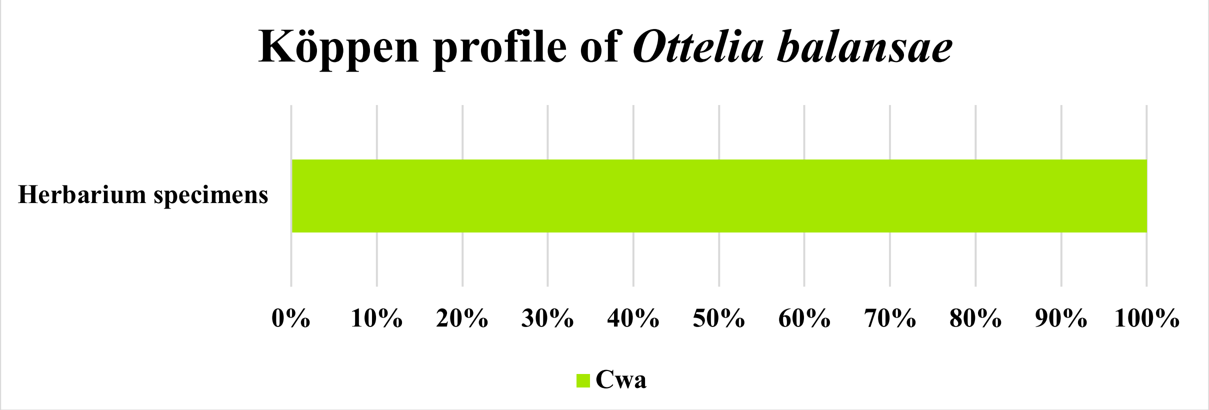

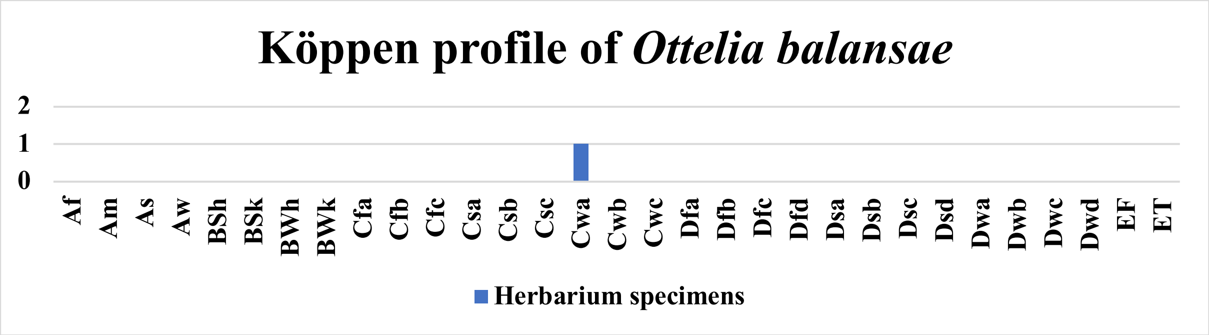

5.2.3.2.4. Köppen profile, distribution and climate map – GBIF occurrences of *Ottelia brasiliensis* Walp. 1852; herbarium specimens excluding duplicate occurrences (n = 47).

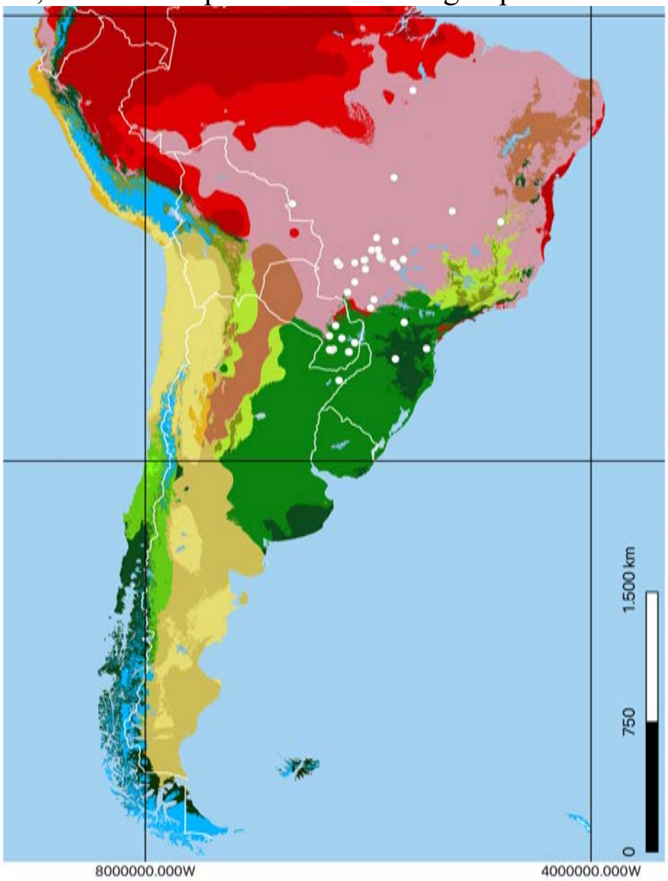

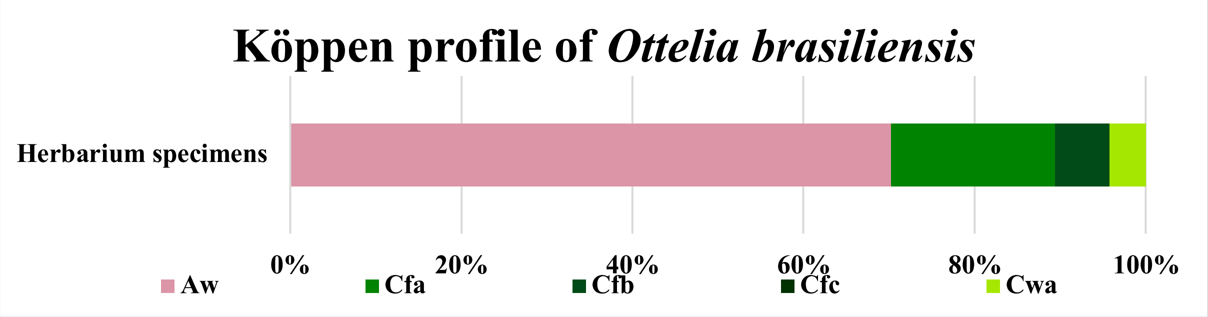

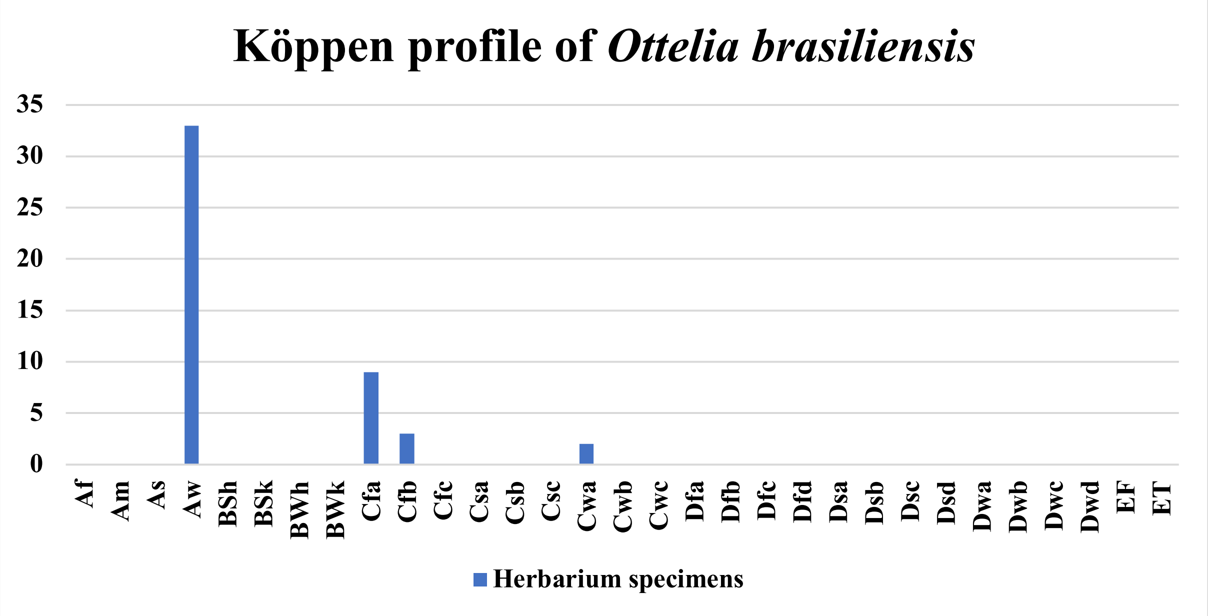

5.2.3.2.5. Köppen profile, distribution and climate map – GBIF occurrences of *Ottelia cordata* (Wall. 1830) Dandy 1934; herbarium specimens excluding duplicate occurrences (n = 1).

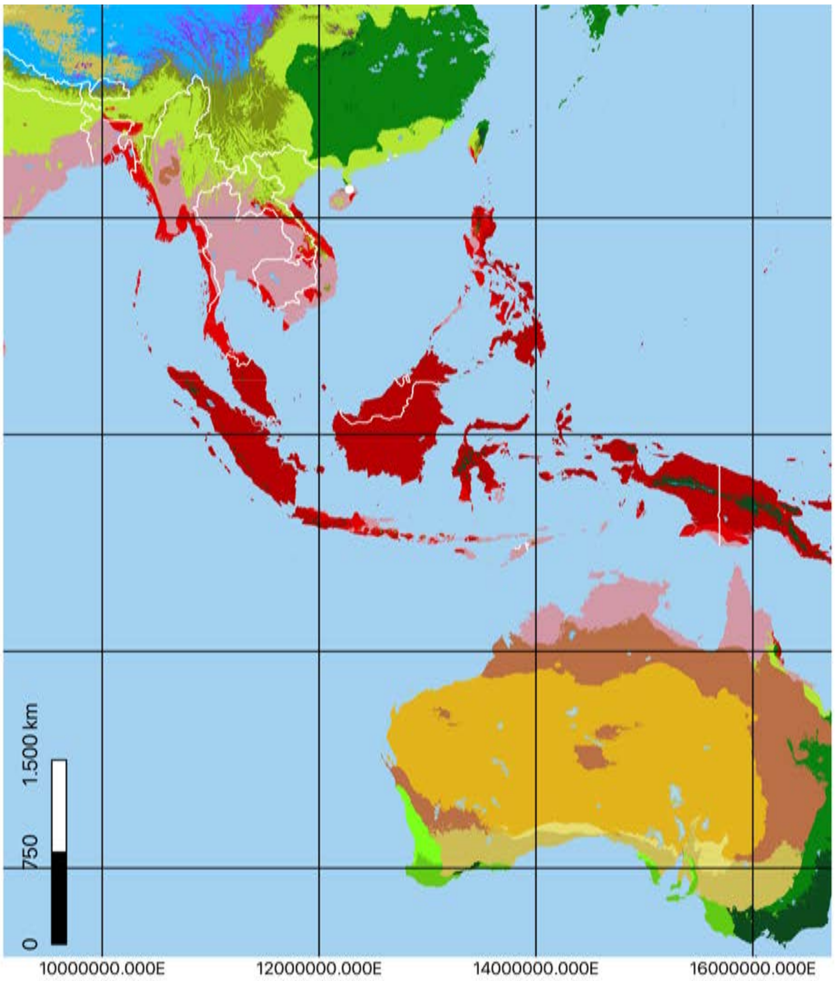

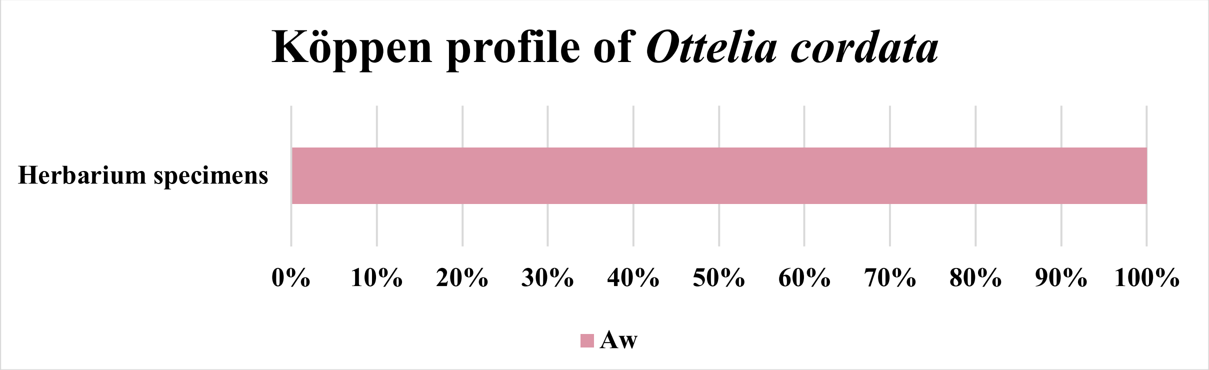

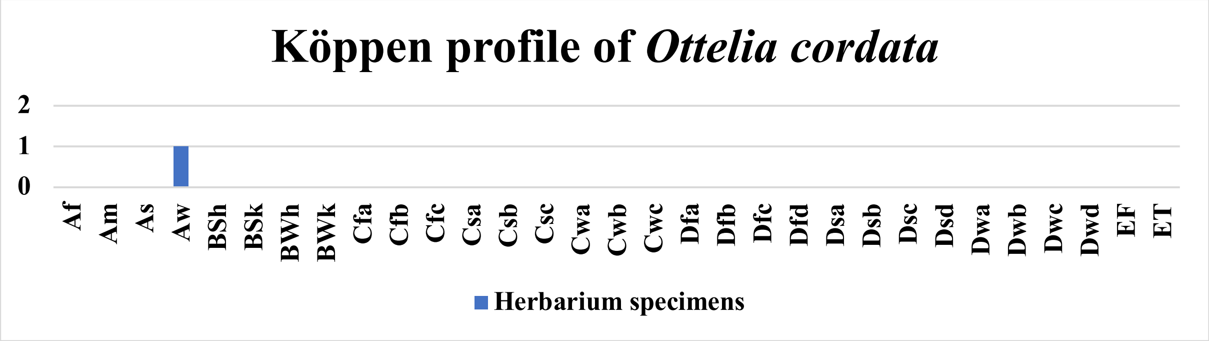

5.2.3.2.6. Köppen profile, distribution and climate map – GBIF occurrences of *Ottelia cylindrica* (T.C.E.Fr.) Dandy 1934; herbarium specimens excluding duplicate occurrences (n = 3).

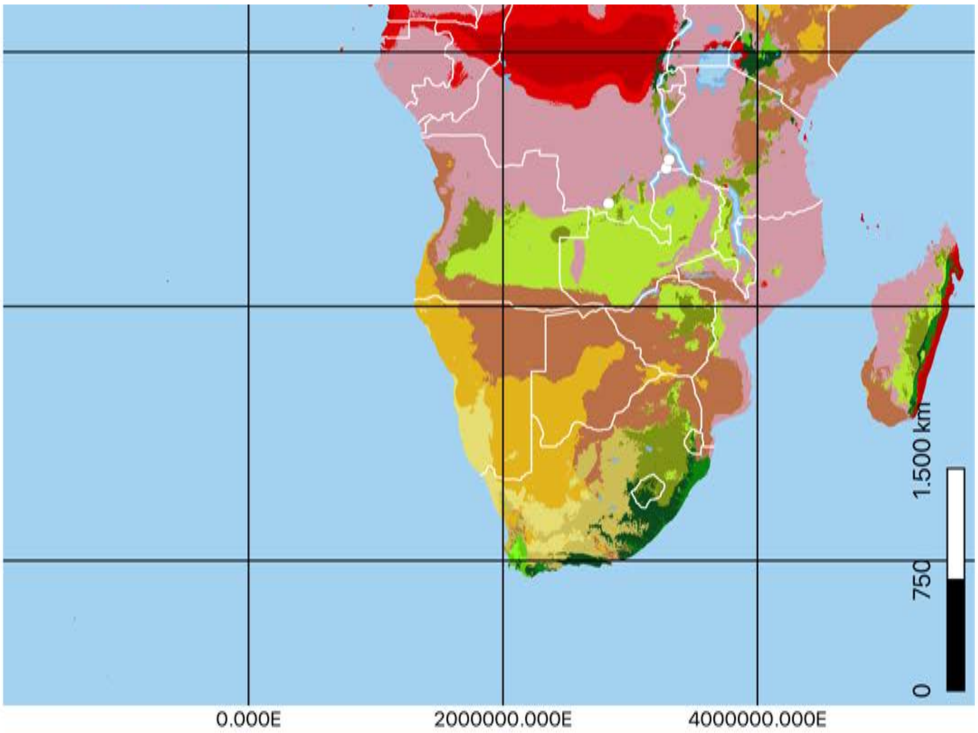

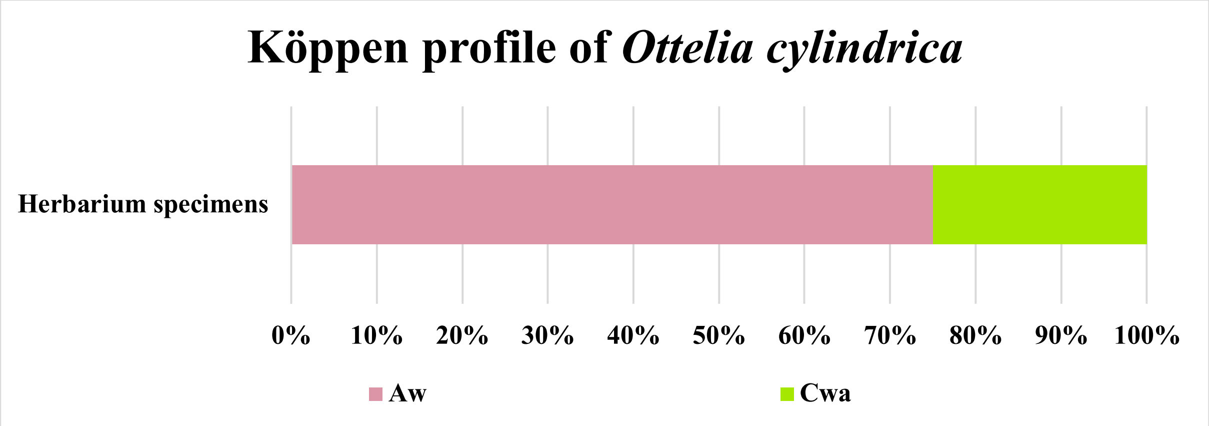

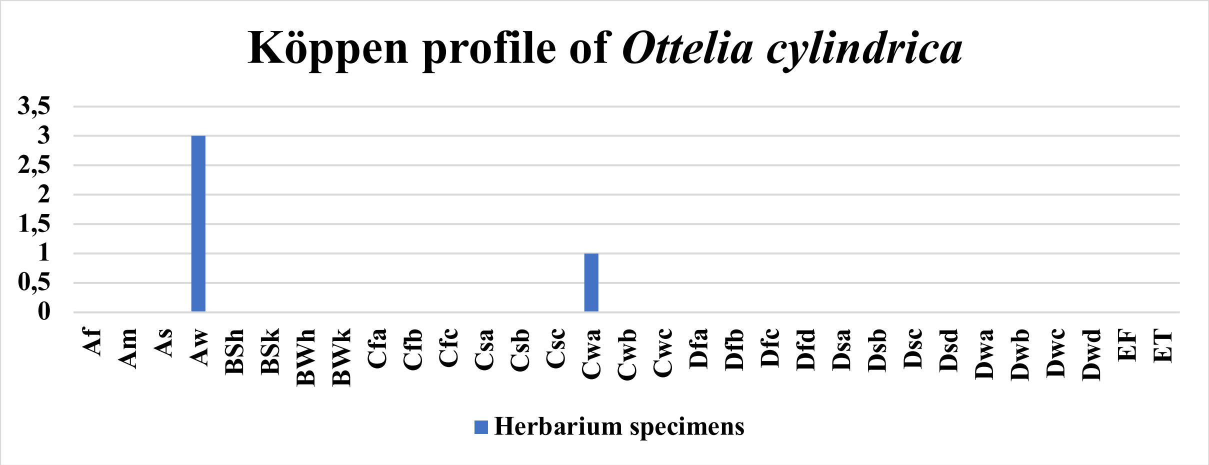

5.2.3.2.7. Köppen profile, distribution and climate map – GBIF occurrences of *Ottelia exserta* (Ridl. 1886) Dandy 1934; herbarium specimens excluding duplicate occurrences (n = 17).

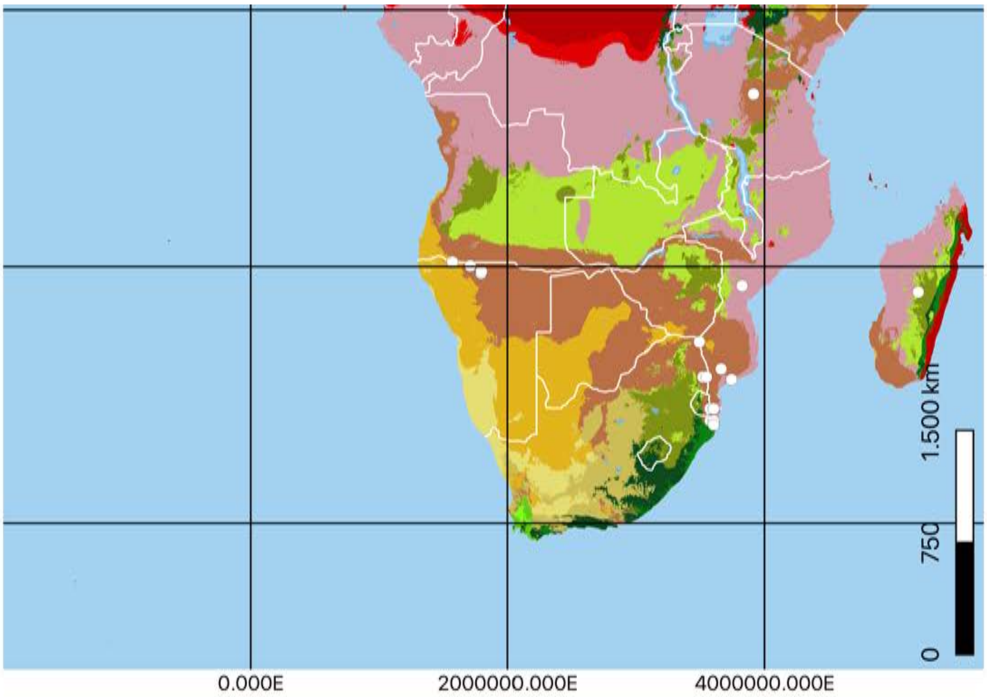

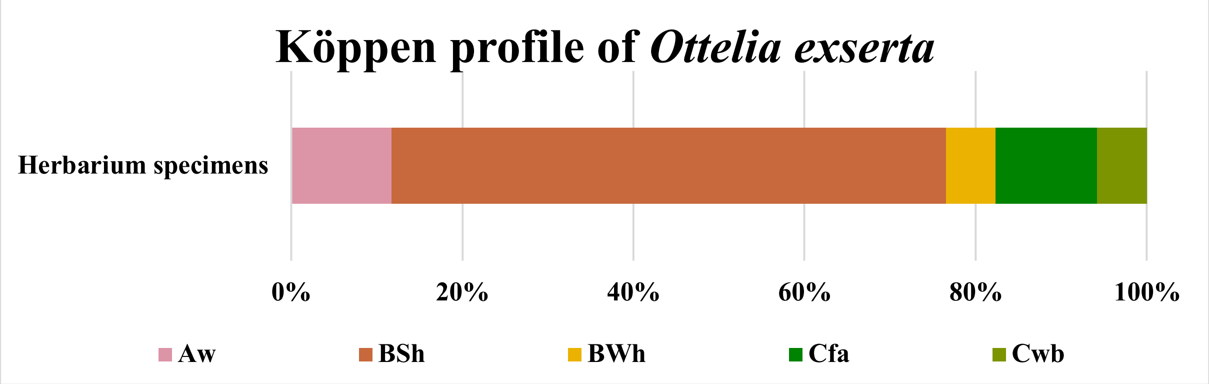

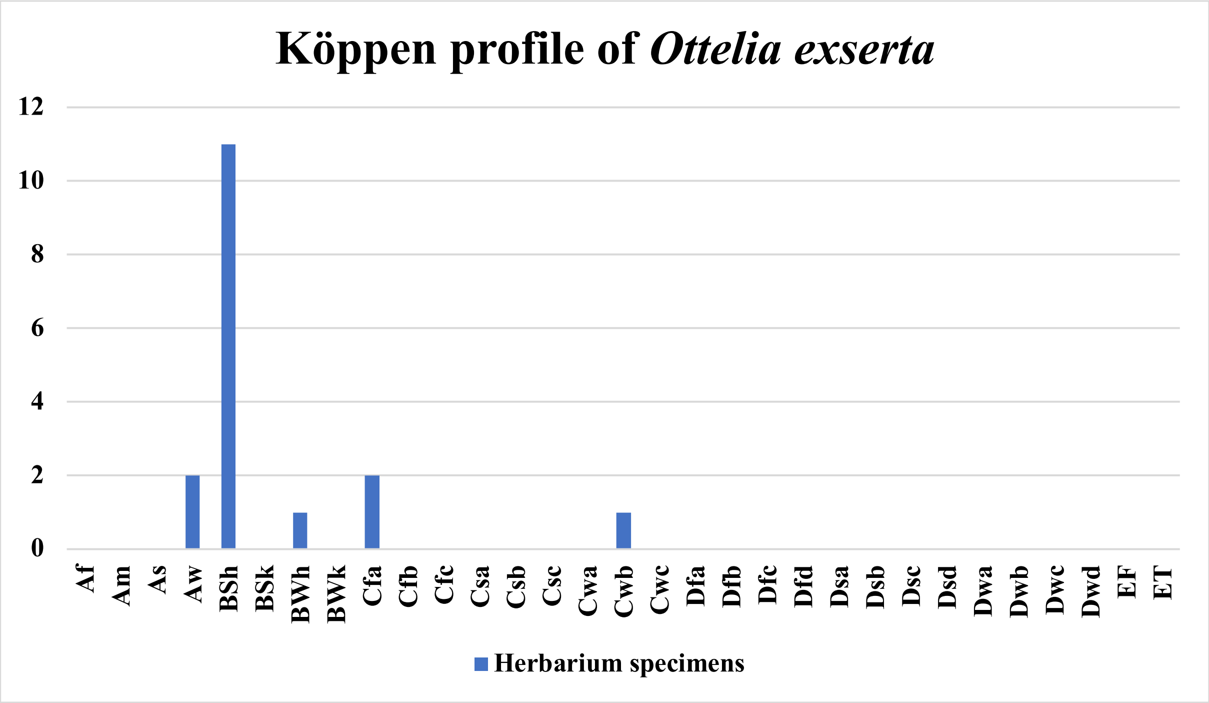

5.2.3.2.8. Köppen profile, distribution and climate map – GBIF occurrences of *Ottelia fischeri* (Gürke 1895) Dandy 1934; herbarium specimens excluding duplicate occurrences (n = 1).

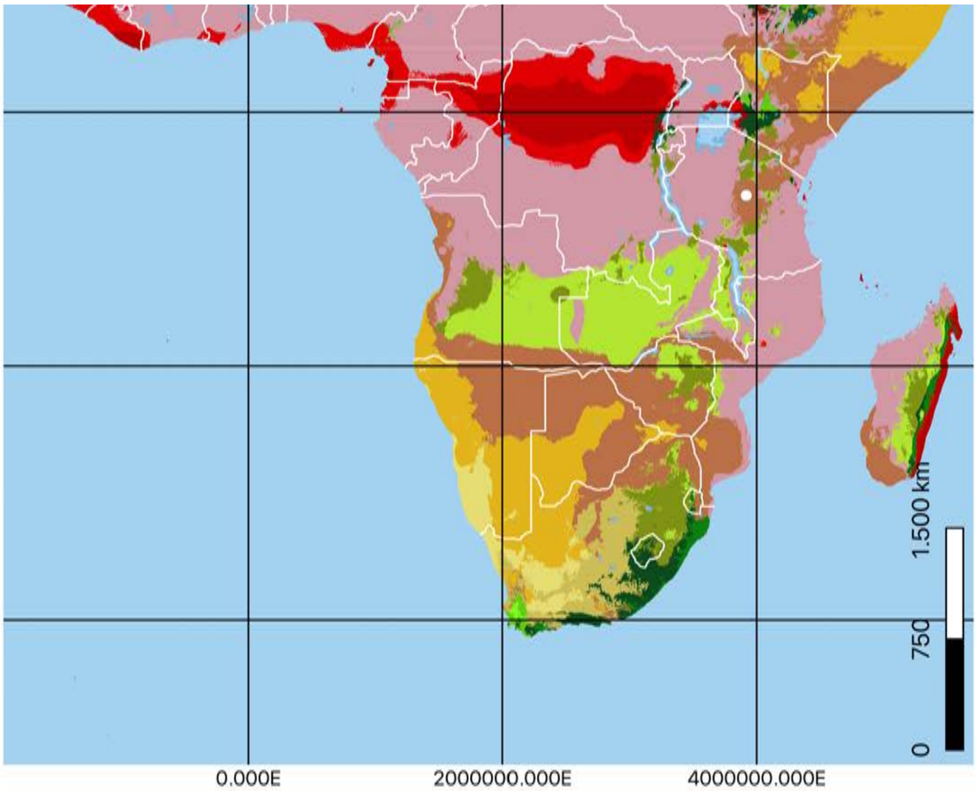

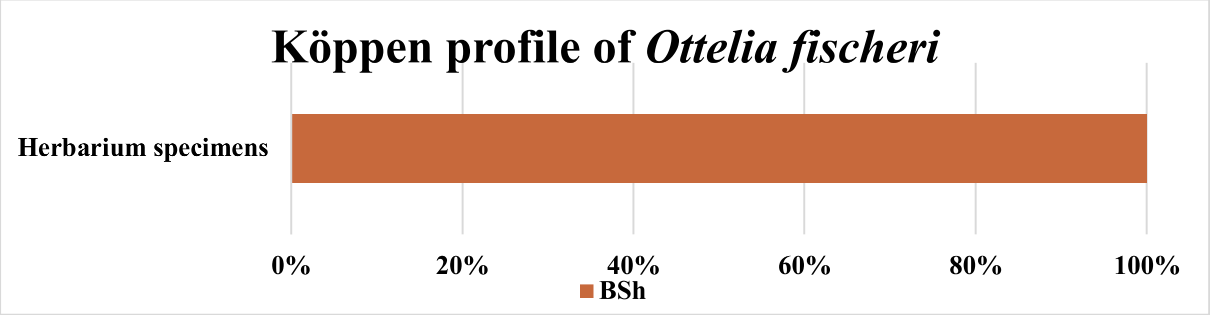

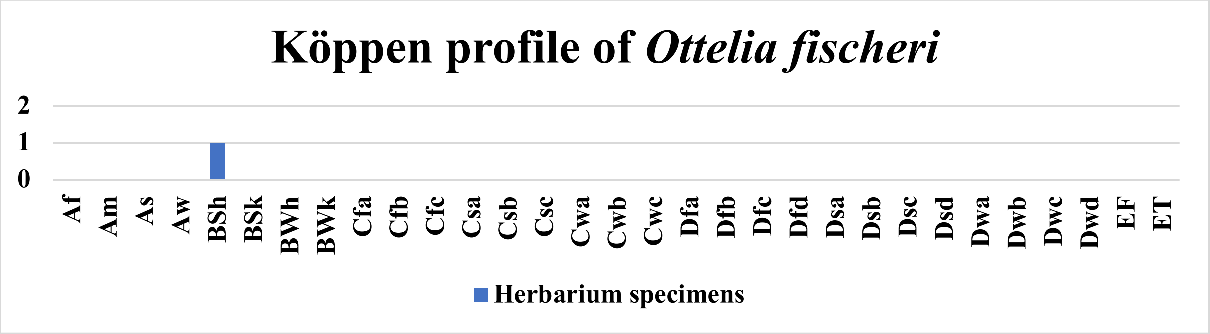

5.2.3.2.9. Köppen profile, distribution and climate map – GBIF occurrences of *Ottelia kunenensis* (Gürke 1895) Dandy 1934; herbarium specimens excluding duplicate occurrences (n = 14).

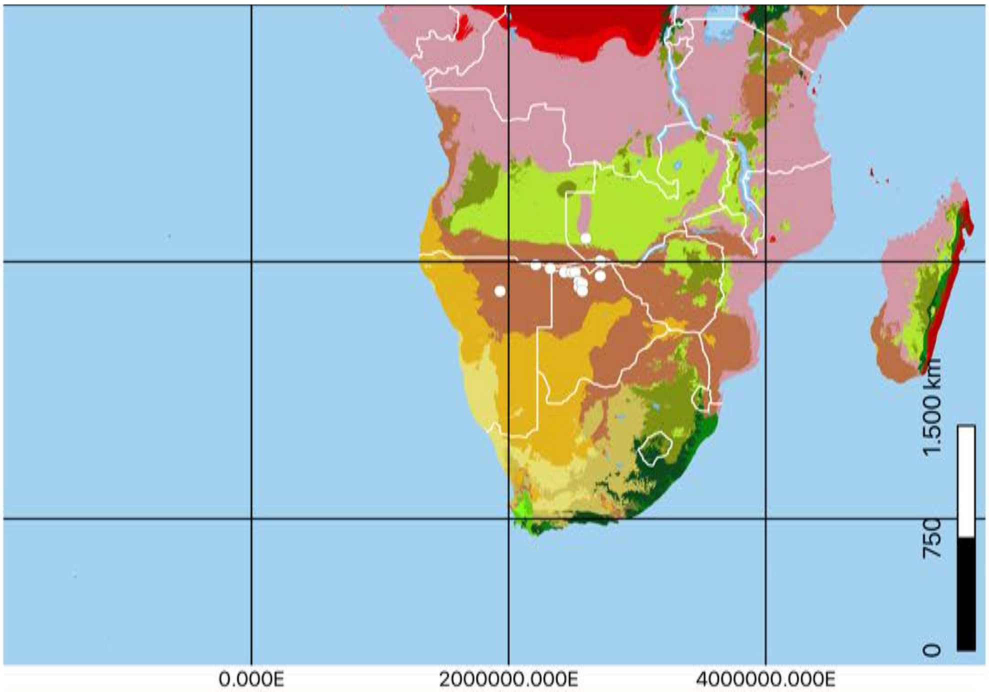

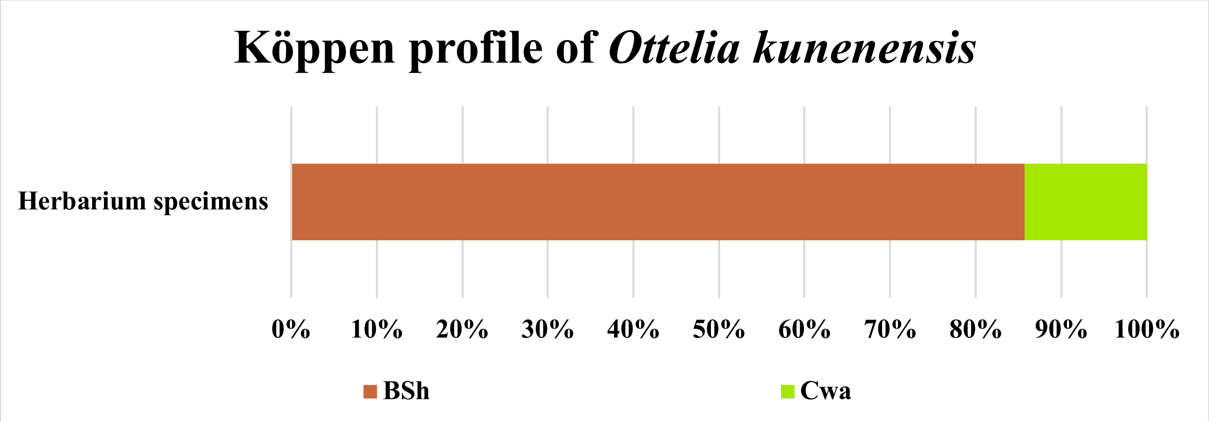

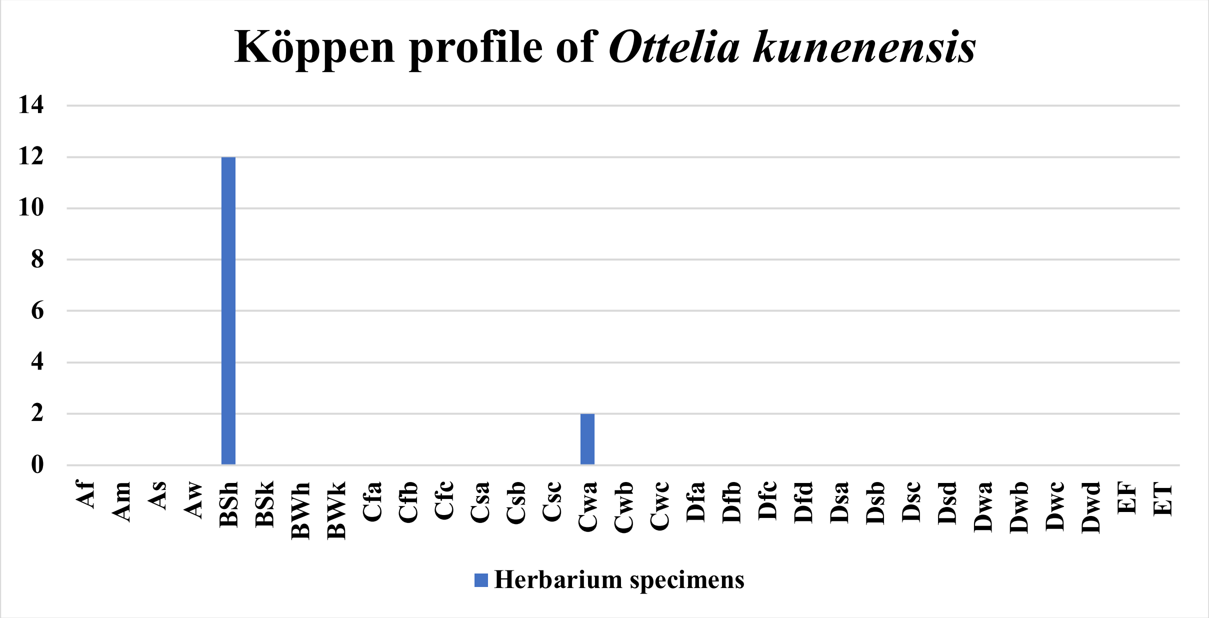

5.2.3.2.10. Köppen profile, distribution and climate map – GBIF occurrences of *Ottelia mesenterium* (Hallier f. 1915) Hartog 1957; herbarium specimens excluding duplicate occurrences (n = 8).

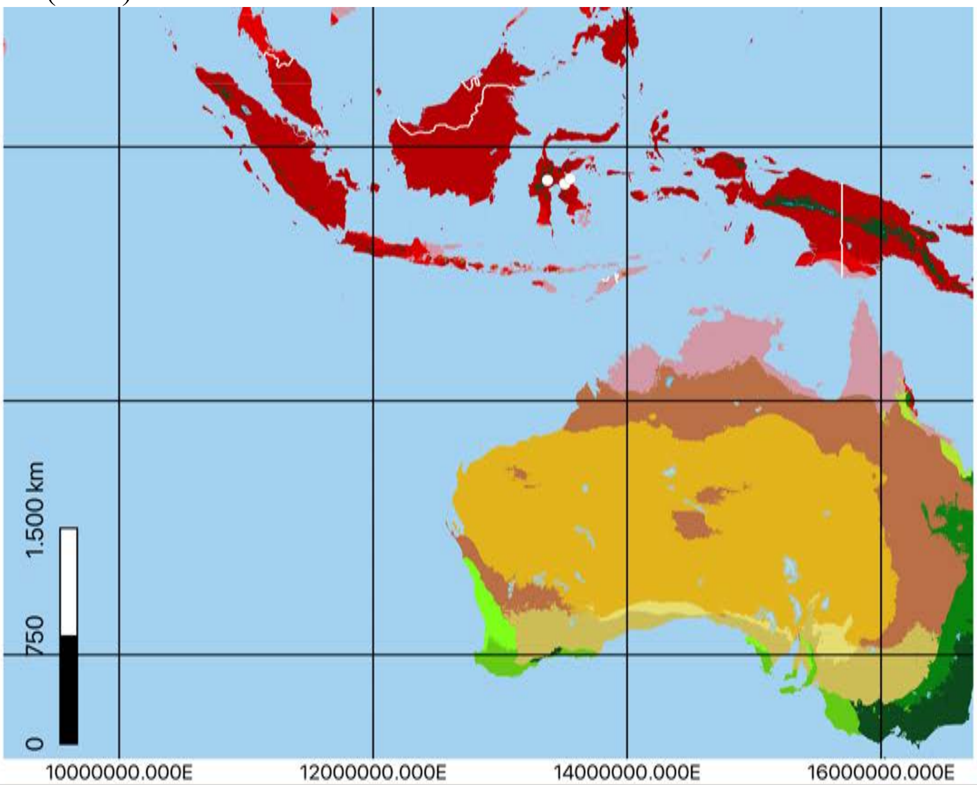

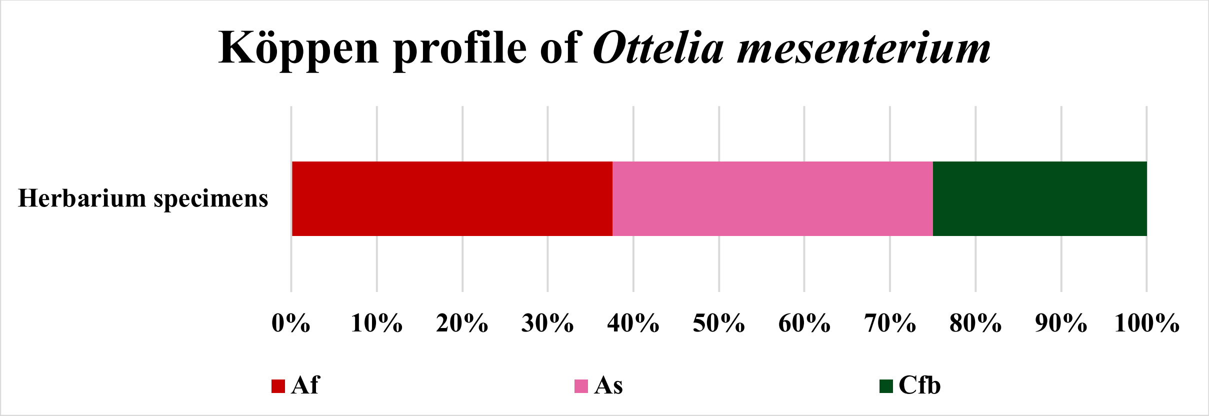

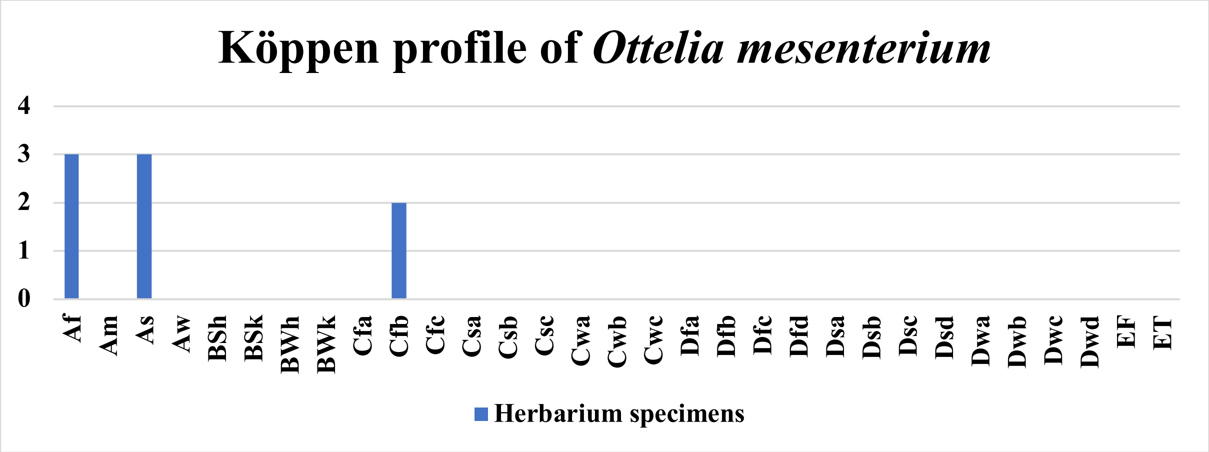

5.2.3.2.11. Köppen profile, distribution and climate map – GBIF occurrences of *Ottelia muricata* (C.H.Wright 1898) Dandy 1934; herbarium specimens excluding duplicate occurrences (n = 16).

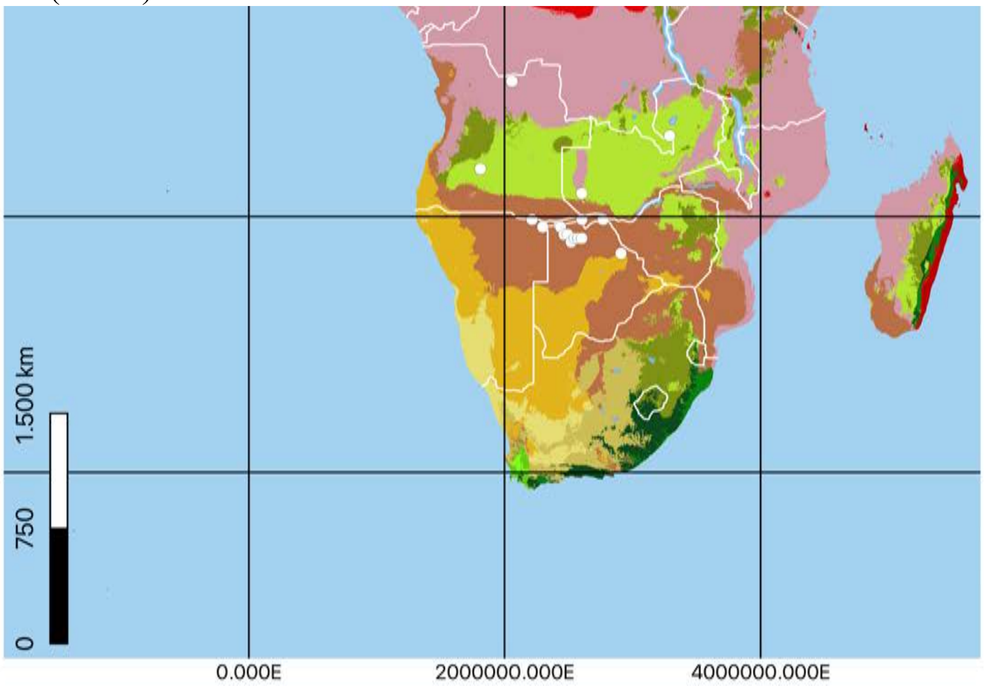

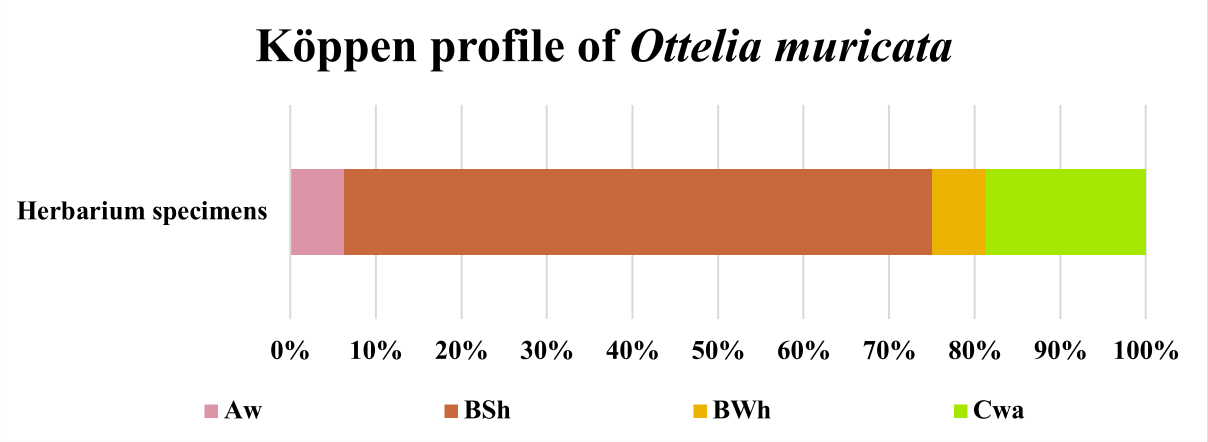

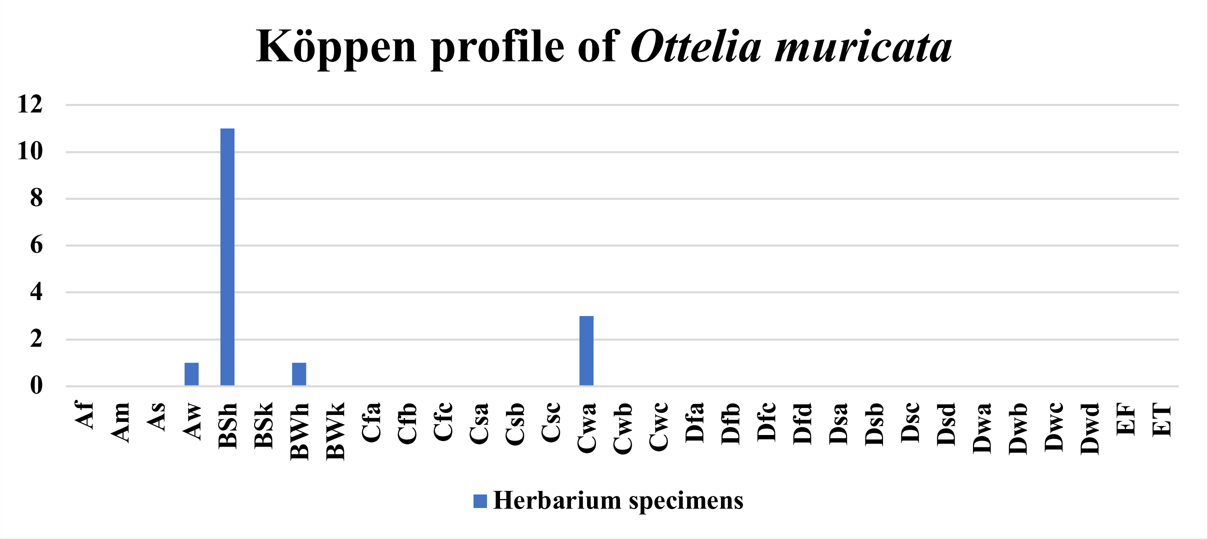

5.2.3.2.12. Köppen profile, distribution and climate map – GBIF occurrences of *Ottelia ovalifolia* (R.Br. 1810) Rich. 1814; herbarium specimens excluding duplicate occurrences (n = 661).

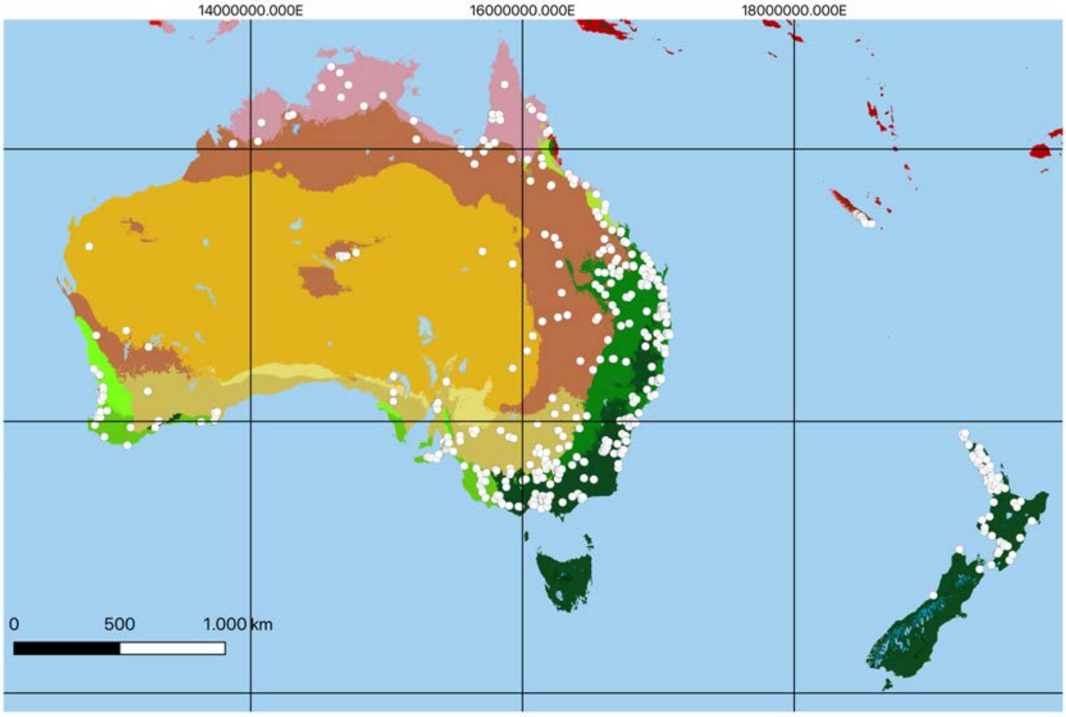

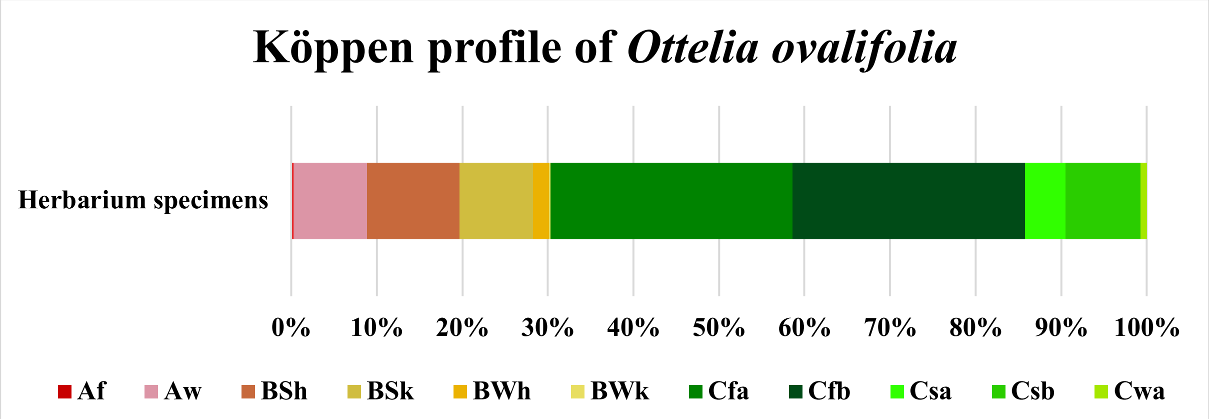

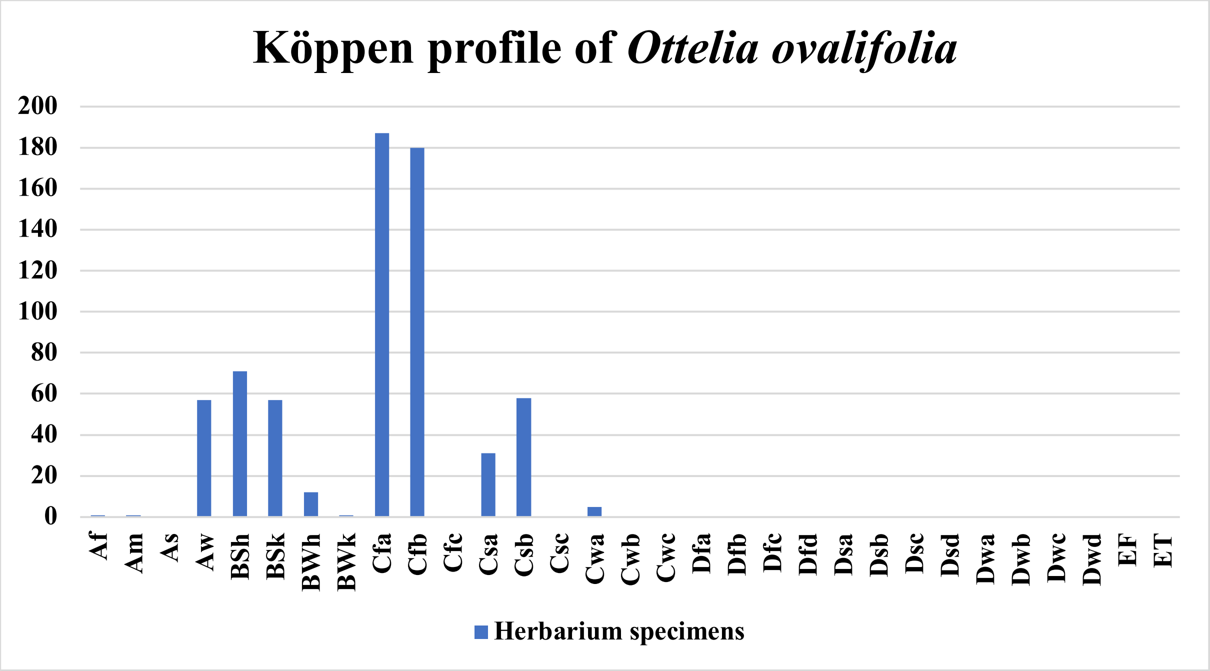

Köppen profile of *Ottelia ovalifolia*
200
180
160
140
120
100
80
60
40
20
0
Af
Am
As
Aw
BSh
BSk
BWh
BWk
Cfa
Cfb
Cfc
Csa
Csb
Csc
Cwa
Cwb
Cwc
Dfa
Dfb
Dfc
Dfd
Dsa
Dsb
Dsc
Dsd
Dwa
Dwb
Dwc
Dwd
EF
ET
Herbarium specimens

5.2.3.2.13. Köppen profile, distribution and climate map – GBIF occurrences of *Ottelia ulvifolia* (Planch. 1849) Walp. 1852; herbarium specimens excluding duplicate occurrences (n = 143).

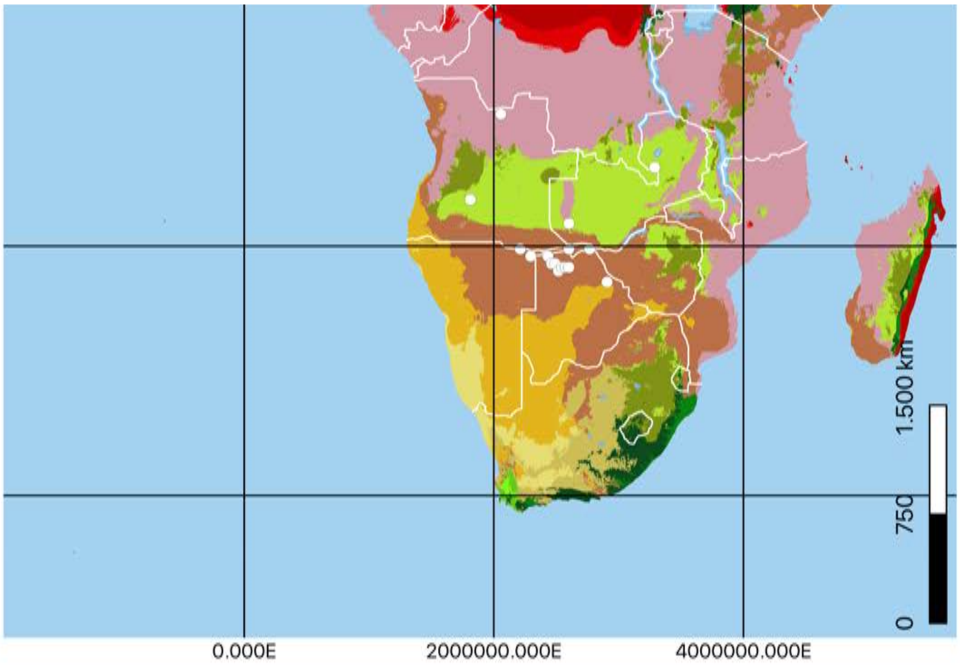

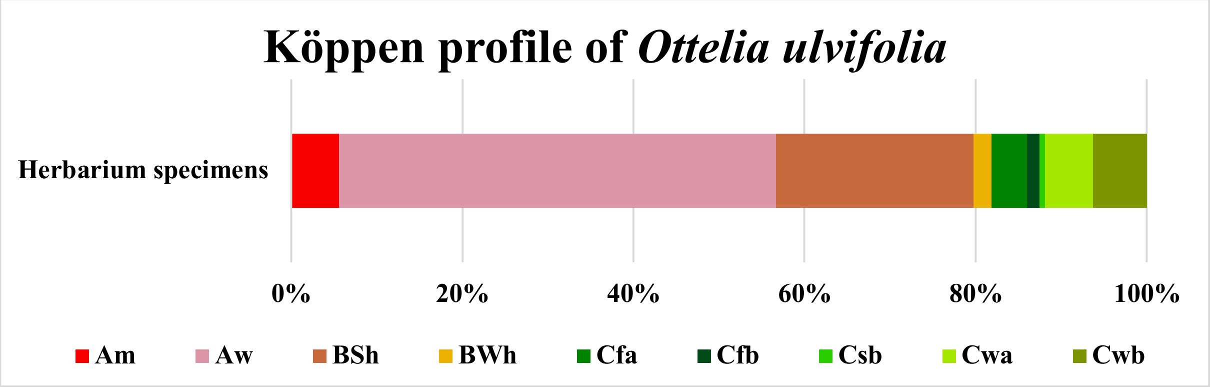

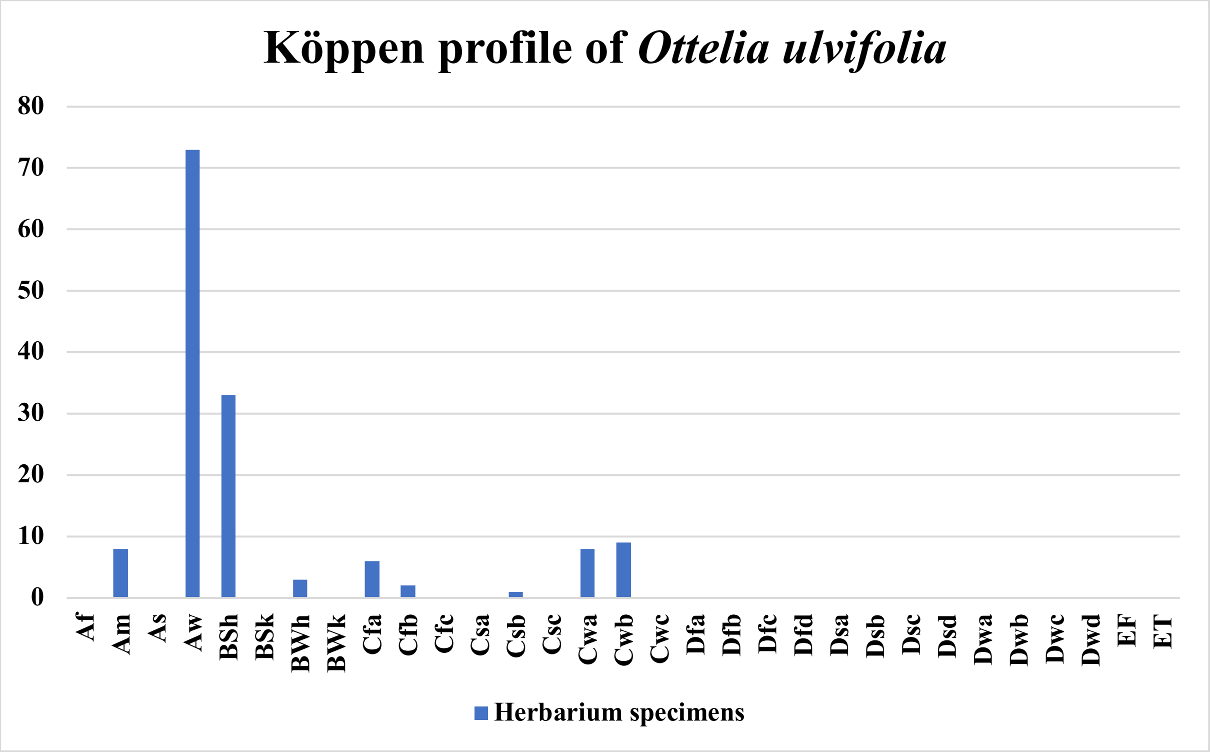

Köppen profile of *Ottelia ulvifolia*
80
70
60
50
40
30
20
10
0
Af
Am
As
Aw
BSh
BSk
BWh
BWk
Cfa
Cfb
Cfc
Csa
Csb
Csc
Cwa
Cwb
Cwc
Dfa
Dfb
Dfc
Dfd
Dsa
Dsb
Dsc
Dsd
Dwa
Dwb
Dwc
Dwd
EF
ET
Herbarium specimens

5.2.3.2.14. Köppen profile, distribution and climate map – GBIF occurrences of *Ottelia verdickii* Gürke ex De Wild., 1903; herbarium specimens excluding duplicate occurrences (n = 7).

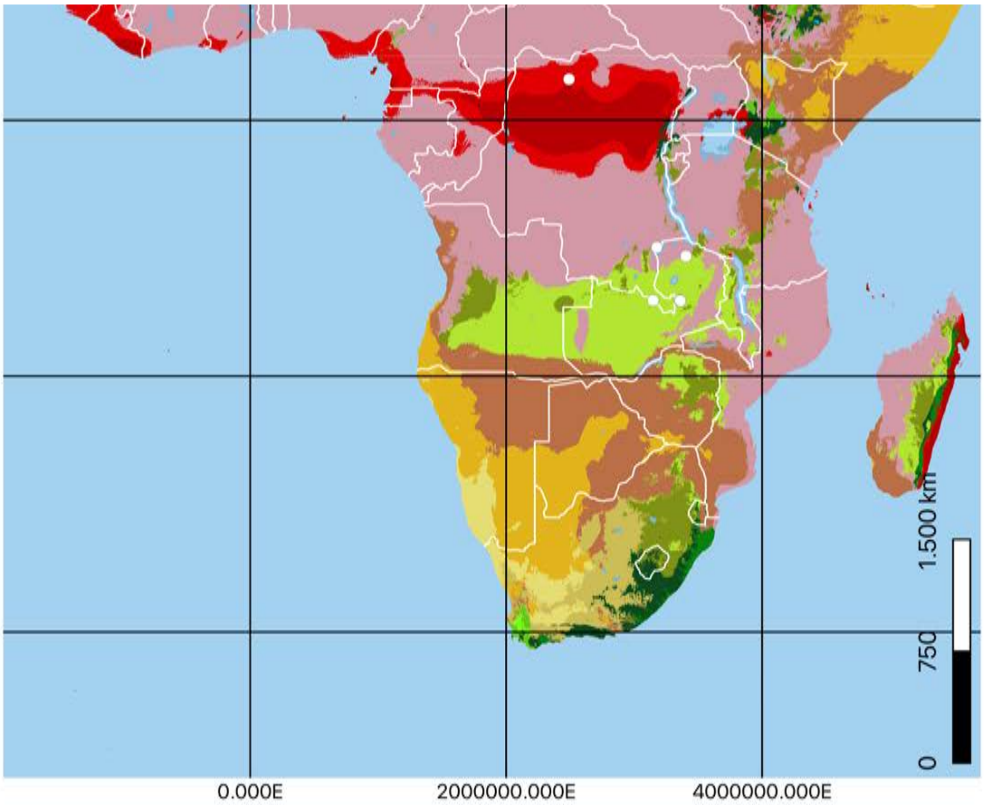

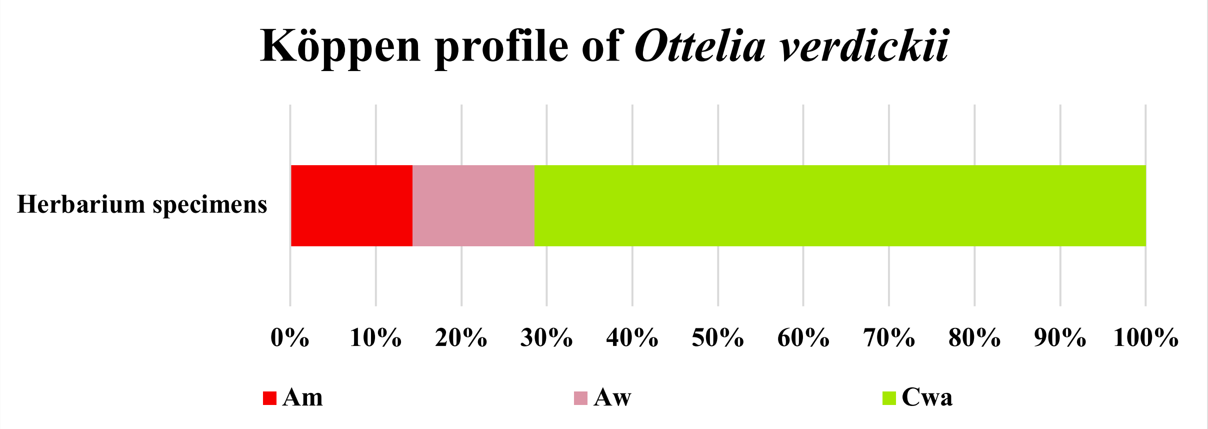

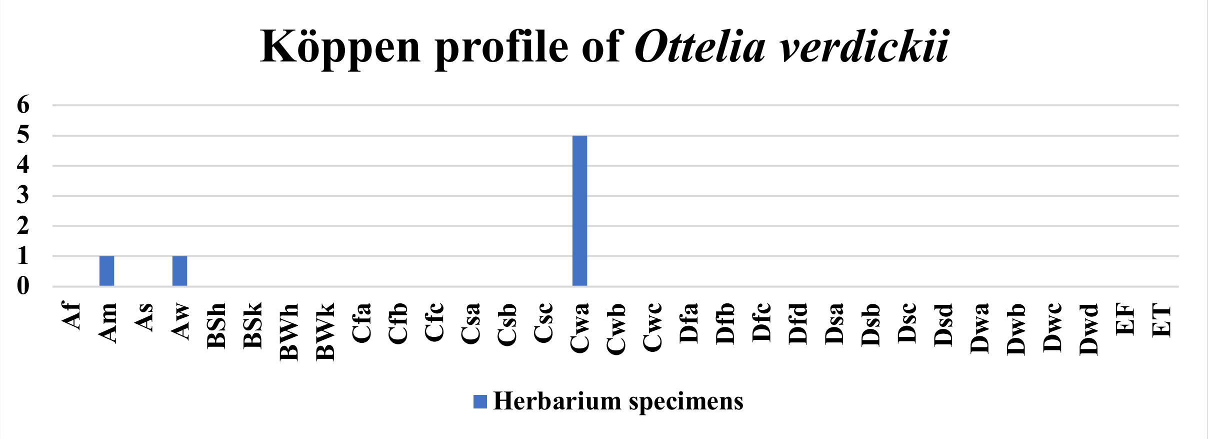

5.3. Clade A

5.3.1. First diverging genus within Clade A of Hydrocharitaceae cf. Chen et. al (2012), comprising *Hydrocharis* L., 1753

5.3.1.1. Genus *Ottelia* Pers. 1805.

Distribution and Köppen profiles of genus *Hydrocharis* incl. *H. chevalieri*, *H. dubia, H. laevigatum*, *H. morsus-ranae,* and *H. spongia* based on preserved specimen (n = 5269).

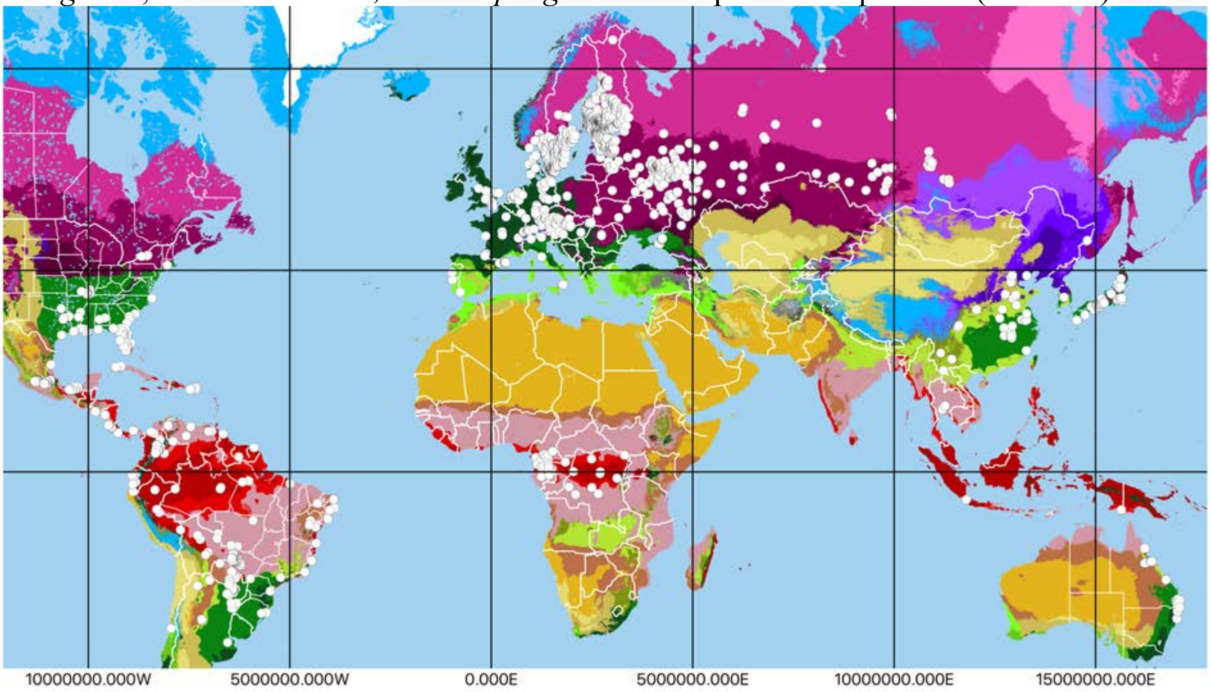

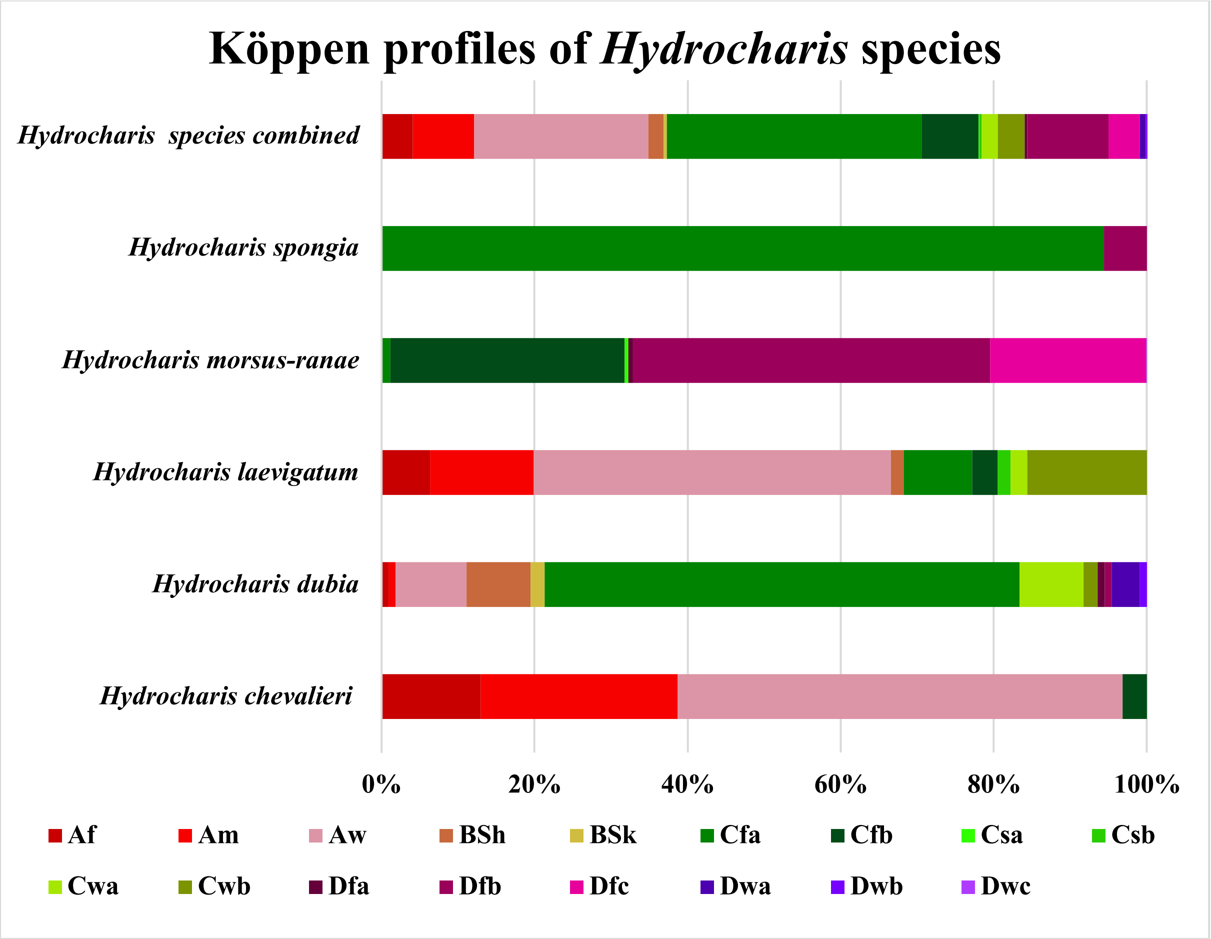

5.3.1.1.1. Köppen profile, distribution and climate map– GBIF occurrences of *Hydrocharis chevalieri* (De Wild. 1922) Dandy 1932; herbarium specimens excluding duplicate occurrences (n = 33).

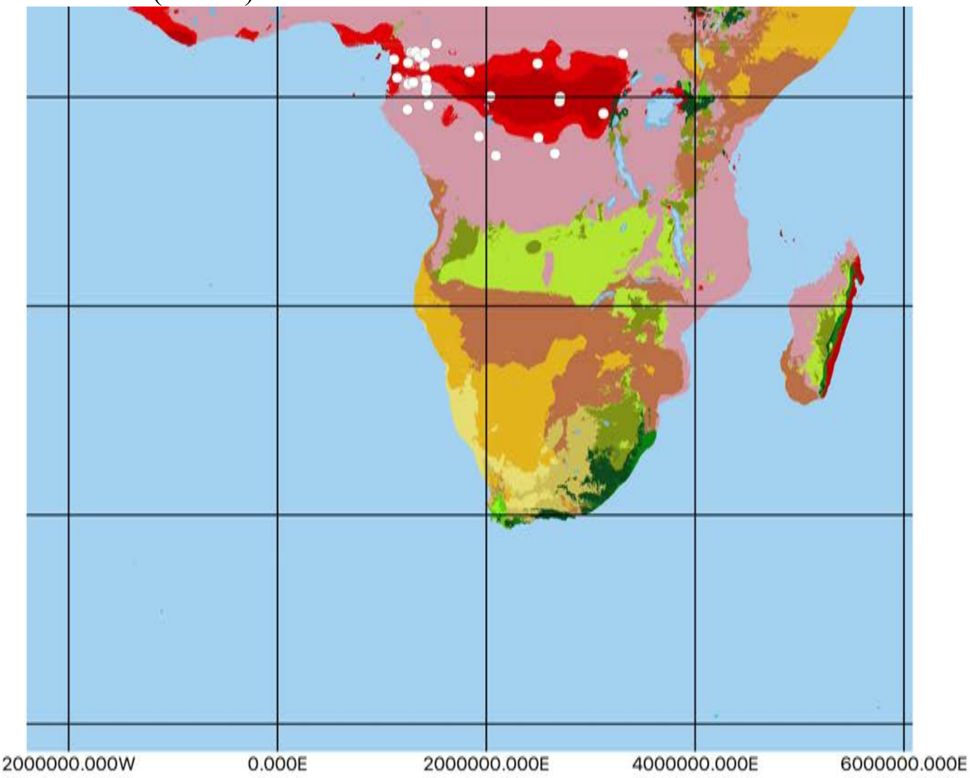

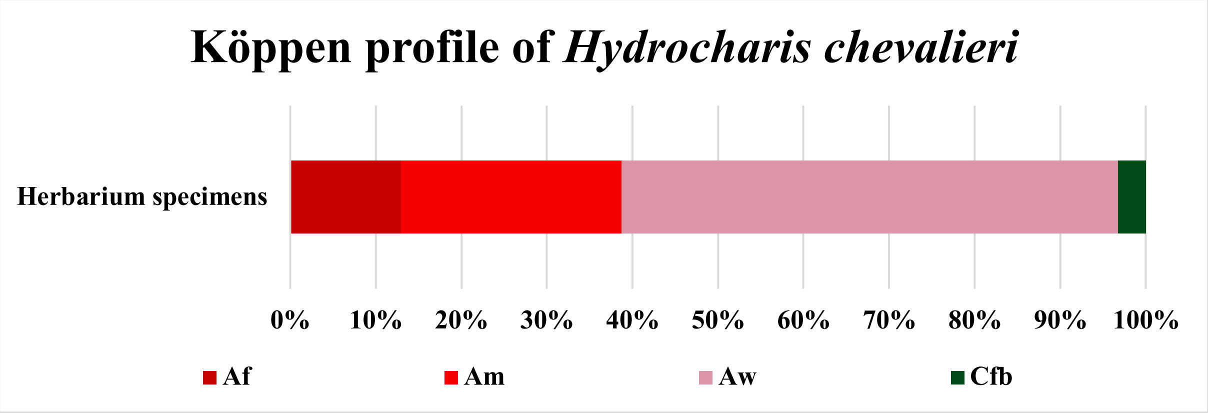

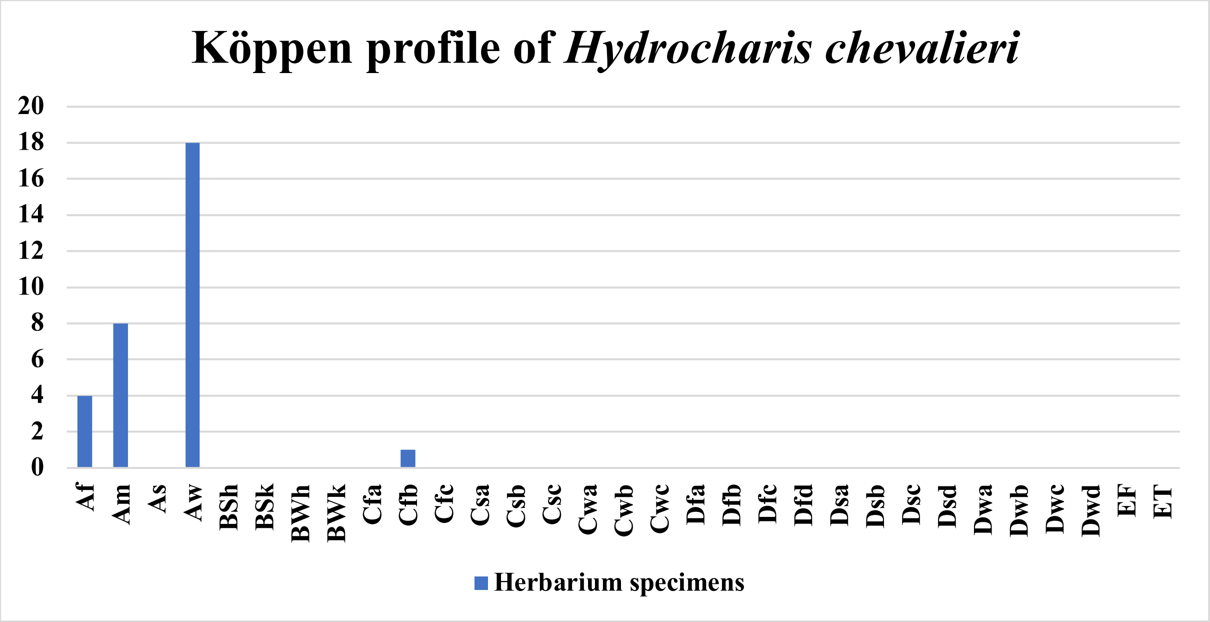
Köppen profile of *Hydrocharis chevalieri*
20
18
16
14
12
10
8
6
4
2
0
Af
Am
As
Aw
BSh
BSk
BWh
BWk
Cfa
Cfb
Cfc
Csa
Csb
Csc
Cwa
Cwb
Cwc
Dfa
Dfb
Dfc
Dfd
Dsa
Dsb
Dsc
Dsd
Dwa
Dwb
Dwc
Dwd
EF
ET
Herbarium specimens

5.3.1.1.2. Köppen profile, distribution and climate map– GBIF occurrences of *Hydrocharis dubia* (Blume 1827) Backer 1925; herbarium specimens excluding duplicate occurrences (n = 108).

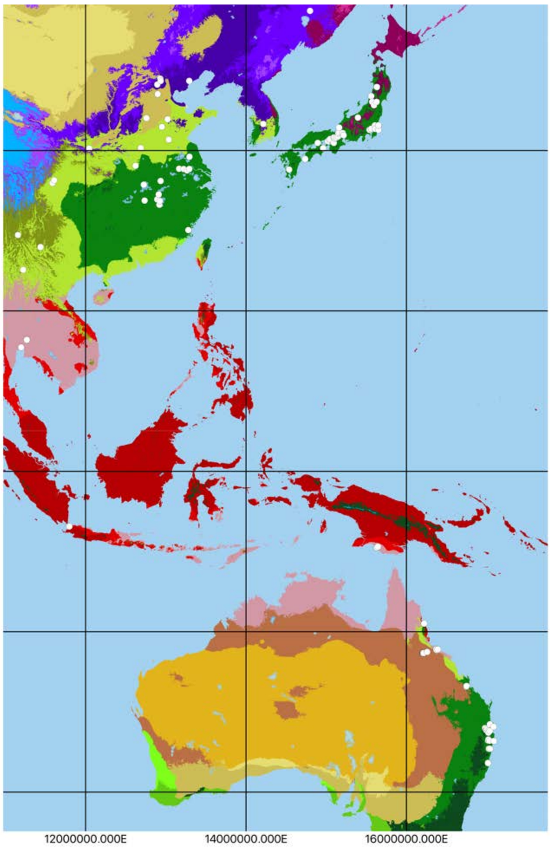

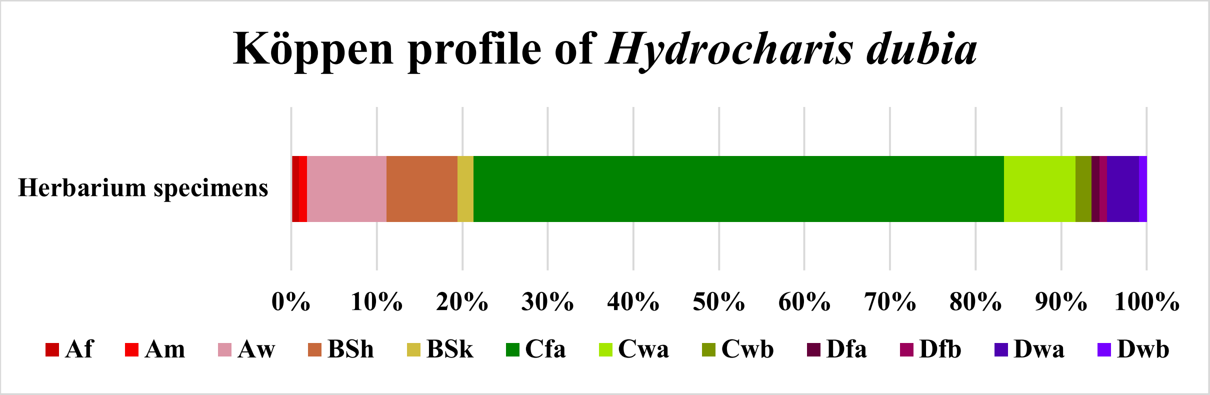

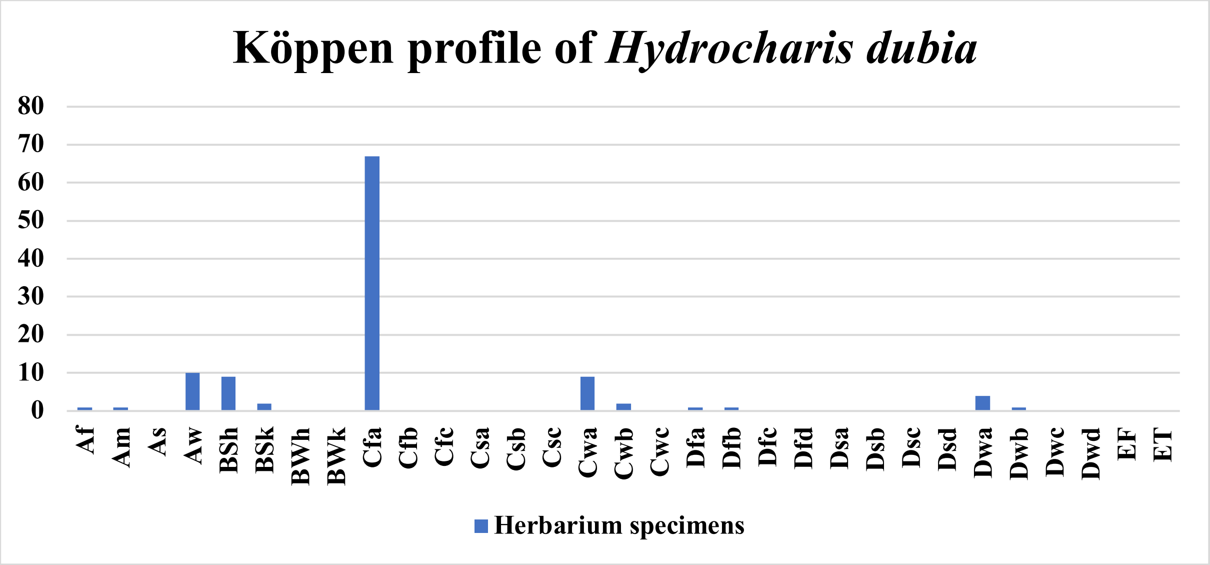
Köppen profile of Hydrocharis dubia
80
70
60
50
40
30
20
10
0
Af
Am
As
Aw
BSh
BSk
BWh
BWk
Cfa
Cfb
Cfc
Csa
Csb
Csc
Cwa
Cwb
Cwc
Dfa
Dfb
Dfc
Dfd
Dsa
Dsb
Dsc
Dsd
Dwa
Dwb
Dwc
Dwd
EF
ET
Herbarium specimens

5.3.1.1.3. Köppen profile, distribution and climate map– GBIF occurrences of *Hydrocharis laevigata* (Humb. & Bonpl. ex Willd. 1810) Byng & Christenh. 2018; herbarium specimens excluding duplicate occurrences (n = 238).

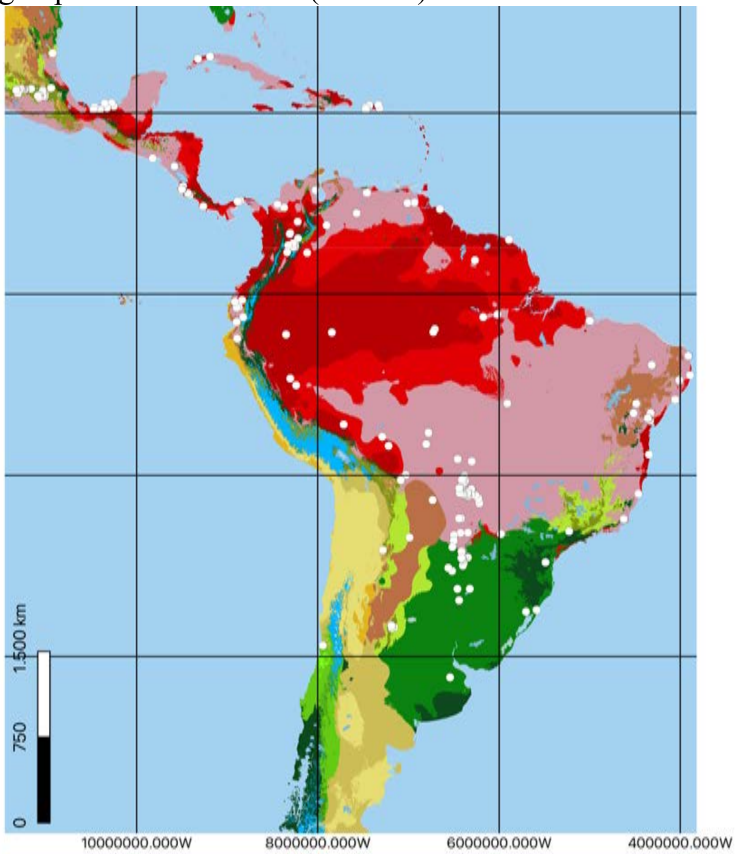

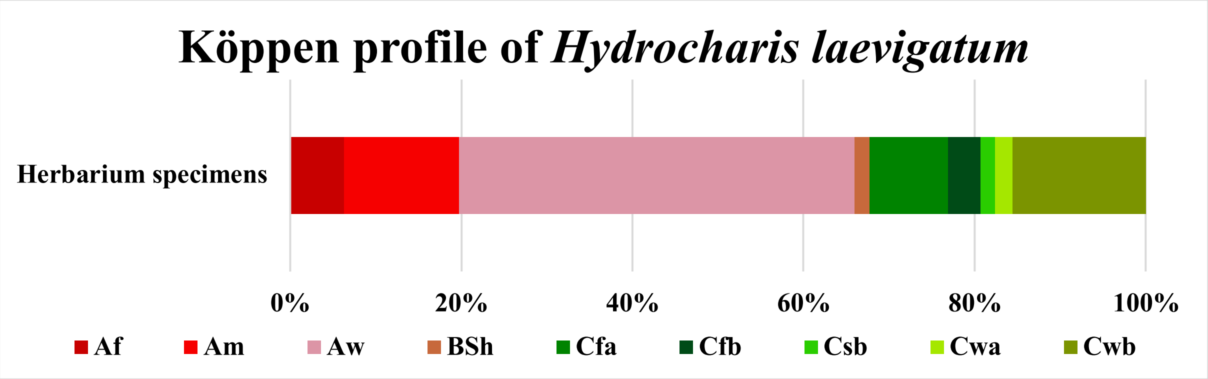

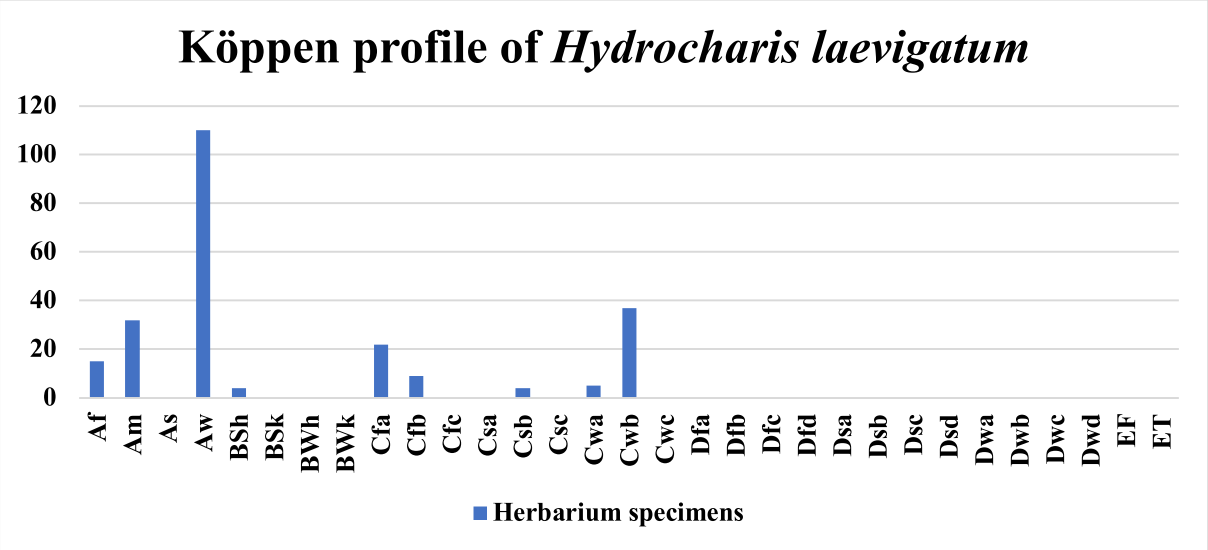

5.3.1.1.4. Köppen profile, distribution and climate map – GBIF occurrences of *Hydrocharis morsus-ranae* L. 1753; herbarium specimens excluding duplicate occurrences (n = 1138).

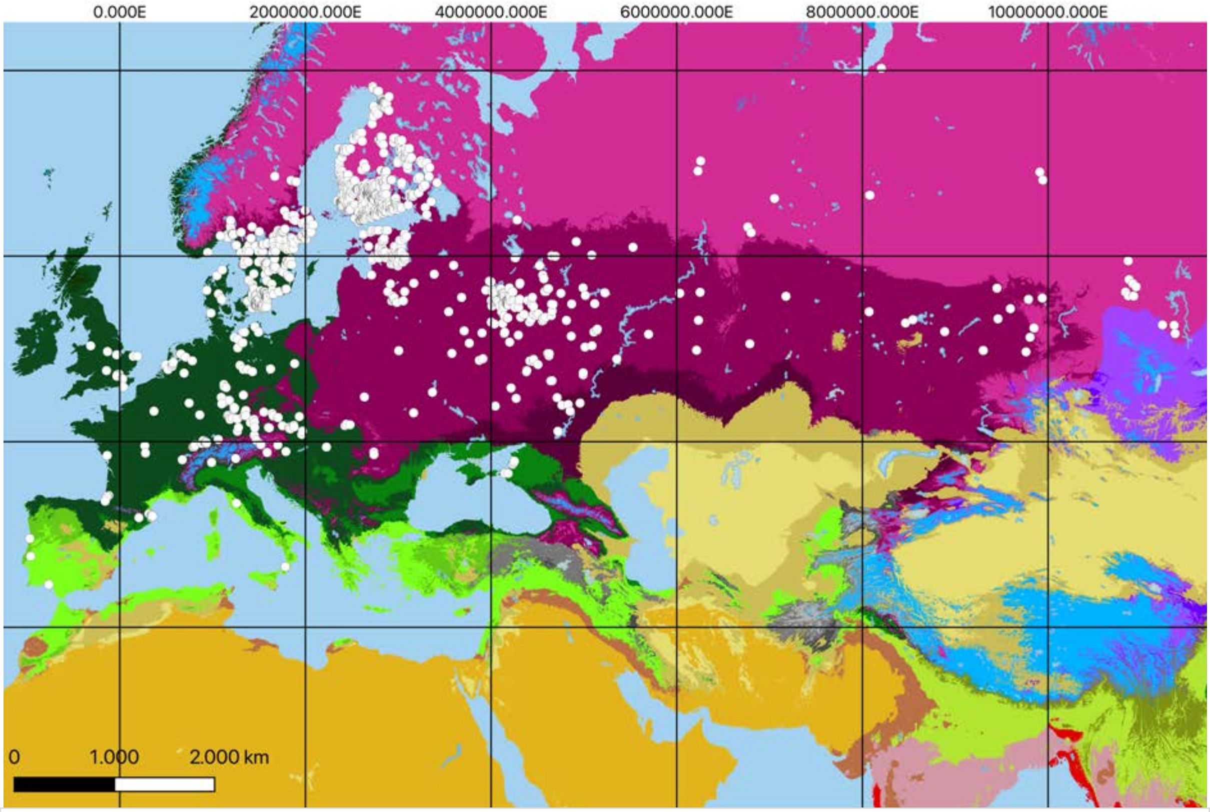

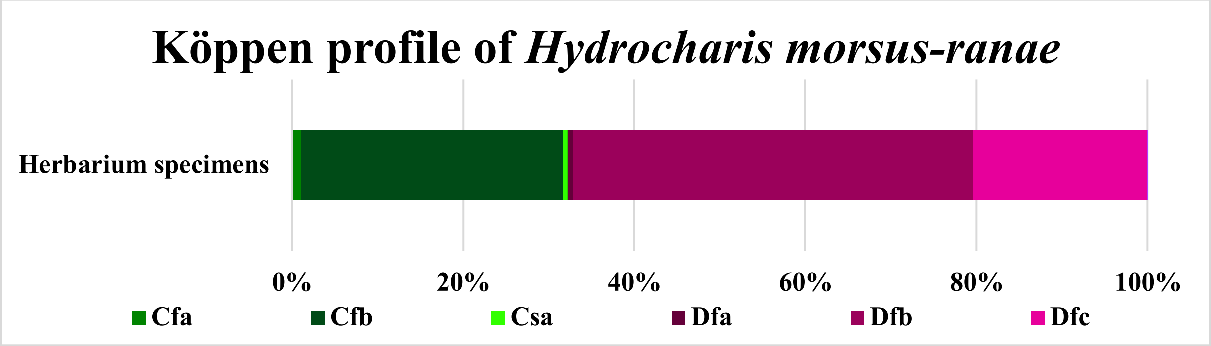

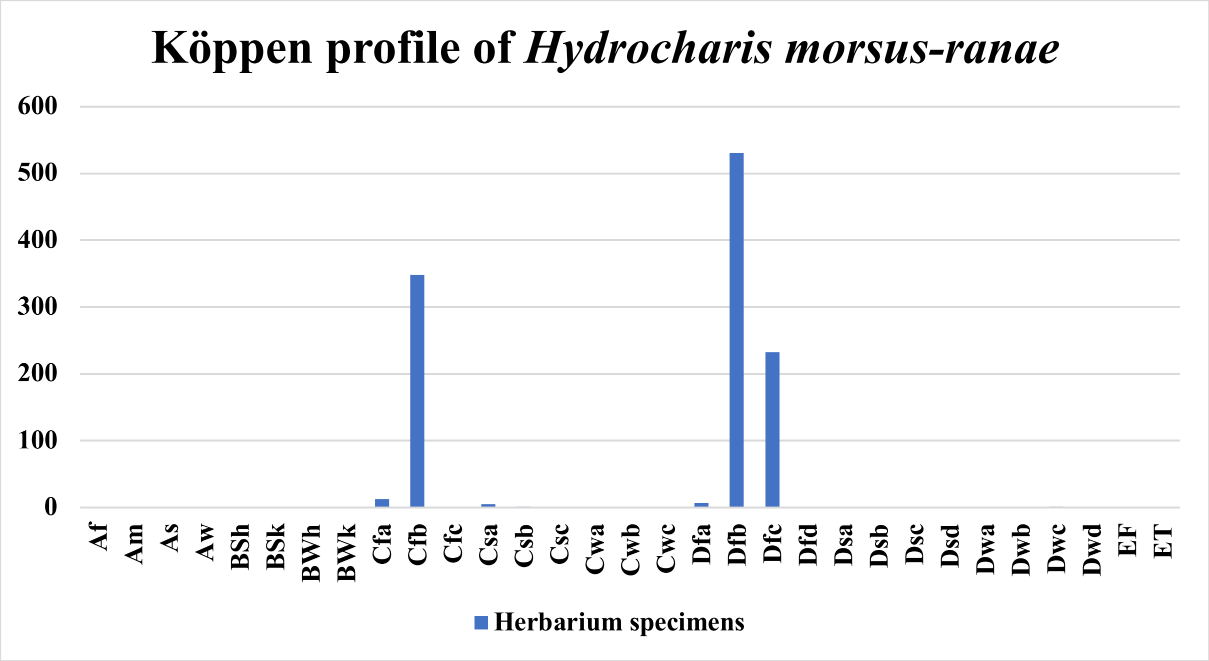
Köppen profile of *Hydrocharis morsus-ranae*
600
500
400
300
200
100
0
Af
Am
As
Aw
BSh
BSk
BWh
BWk
Cfa
Cfb
Cfc
Csa
Csb
Csc
Cwa
Cwb
Cwc
Dfa
Dfb
Dfc
Dfd
Dsa
Dsb
Dsc
Dsd
Dwa
Dwb
Dwc
Dwd
EF
ET
Herbarium specimens

5.3.1.1.5. Köppen profile, distribution and climate map – GBIF occurrences of *Hydrocharis spongia* Bosc, 1807; herbarium specimens excluding duplicate occurrences (n = 71).

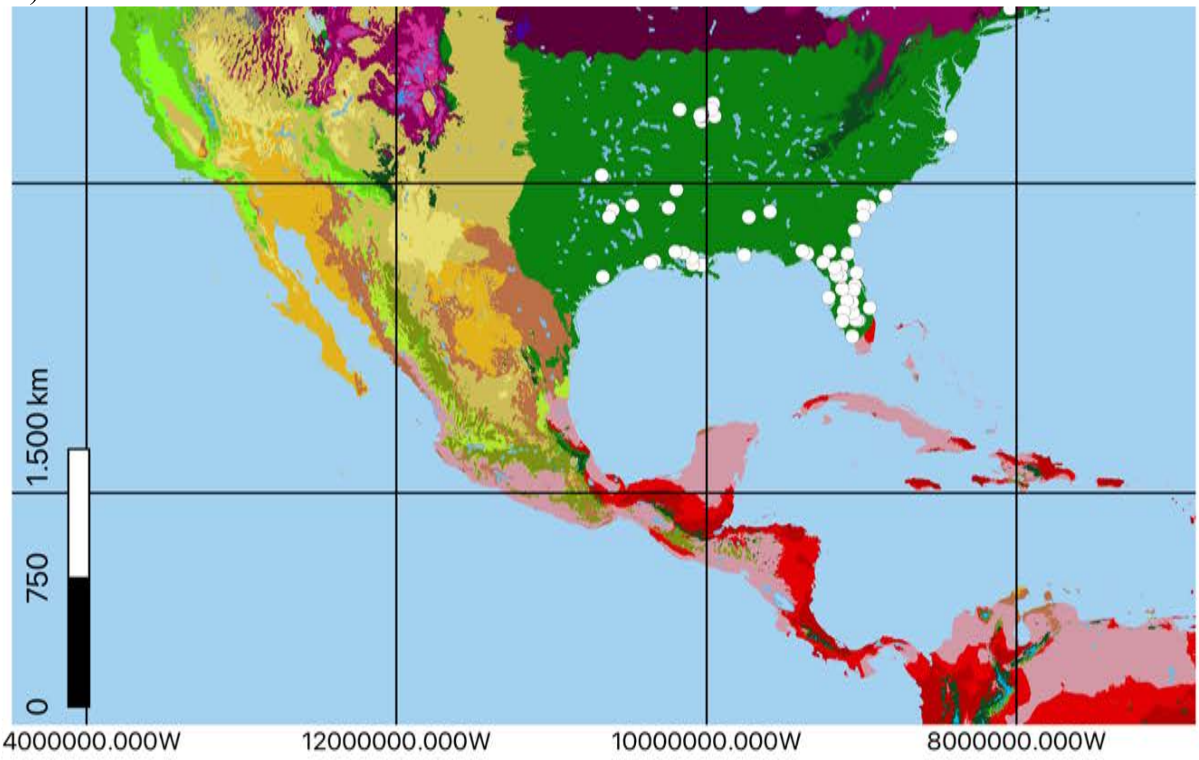

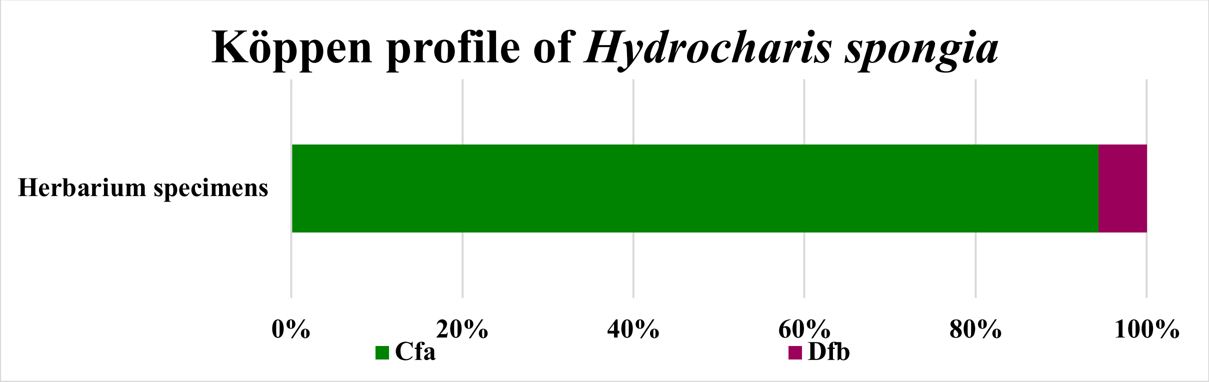

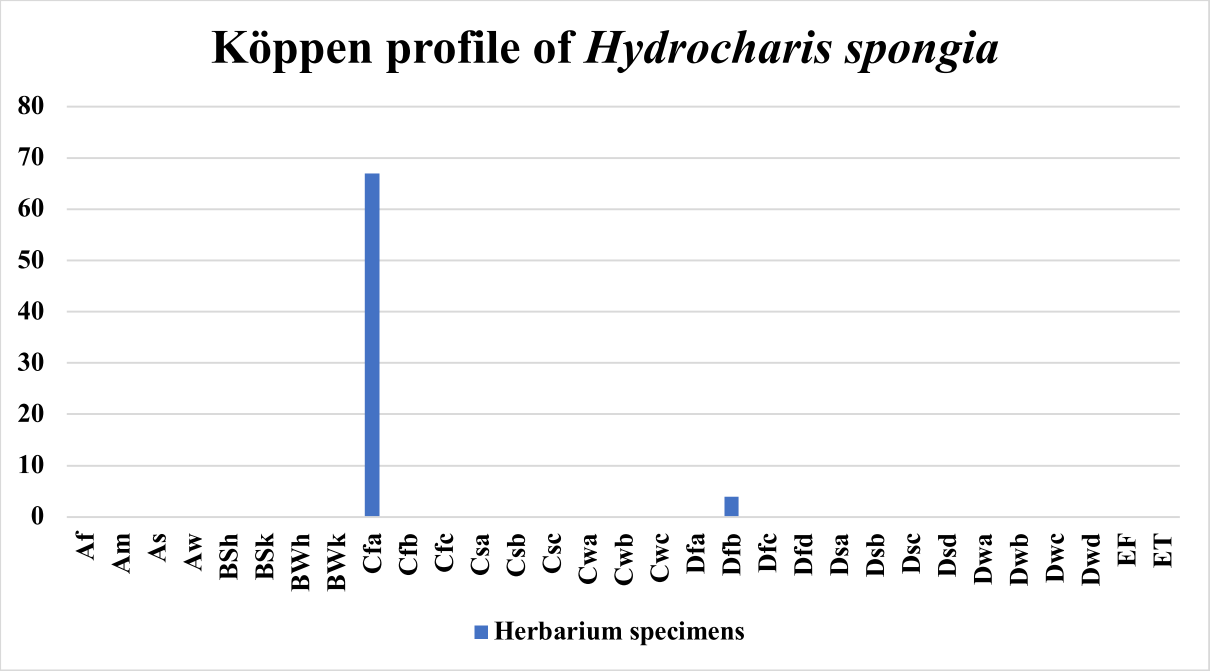
Köppen profile of Hydrocharis spongia
80
70
60
50
40
30
20
10
0
Af
Am
As
Aw
BSh
BSk
BWh
BWk
Cfa
Cfb
Cfc
Csa
Csb
Csc
Cwa
Cwb
Cwc
Dfa
Dfb
Dfc
Dfd
Dsa
Dsb
Dsc
Dsd
Dwa
Dwb
Dwc
Dwd
EF
ET
Herbarium specimens

5.3.2. Second diverging genus within Clade A of Hydrocharitaceae cf. Chen et. al (2012), comprising *Enhalus* Rich. 1811, *Halophia* Thouars 1806, and *Thalassia* Banks ex K.D.Koenig 1805

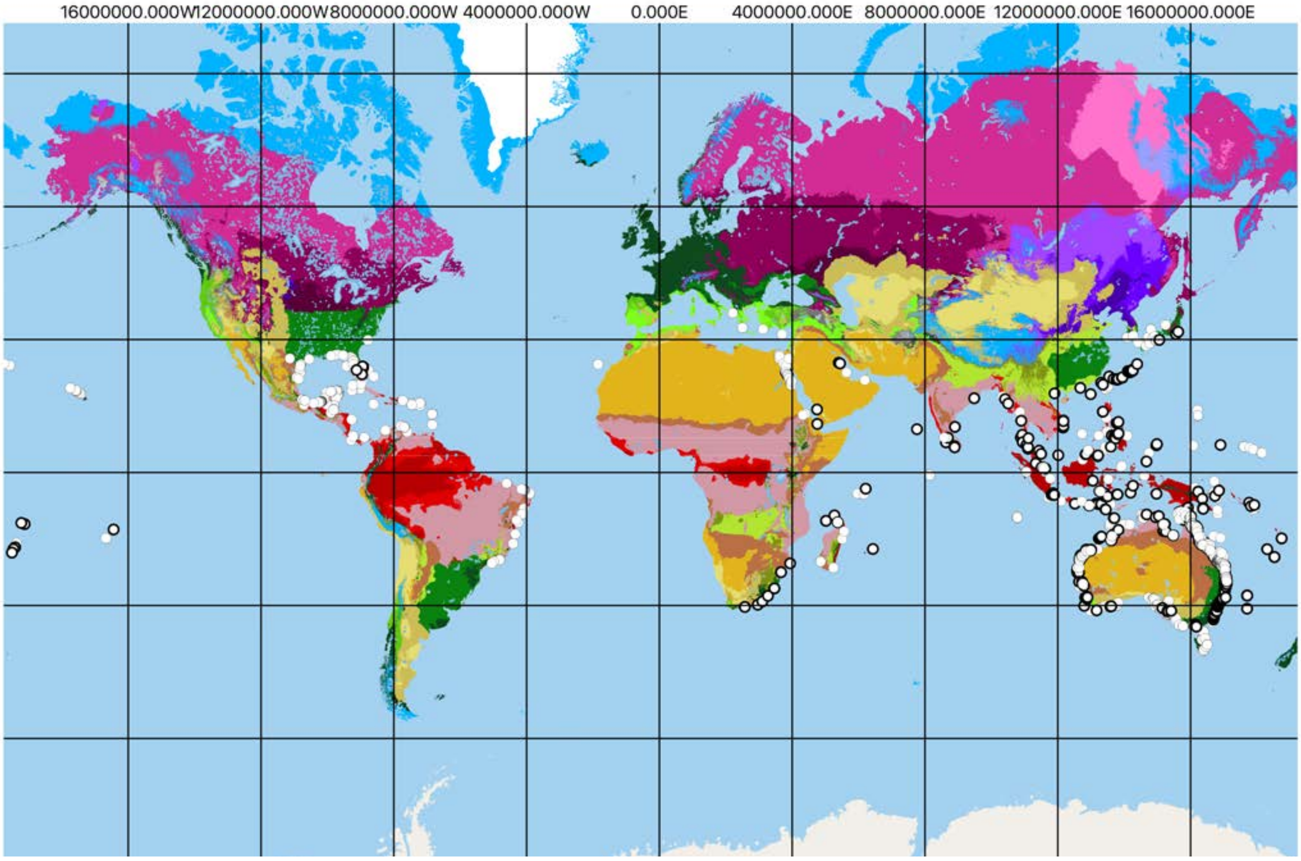

5.3.2.1. Genus *Enhalus* Rich. 1811.

Köppen profile, distribution and climate map – GBIF occurrences of *Enhalus acoroides* (L.f. 1782) Royle 1839; herbarium specimens excluding duplicate occurrences (n = 102).

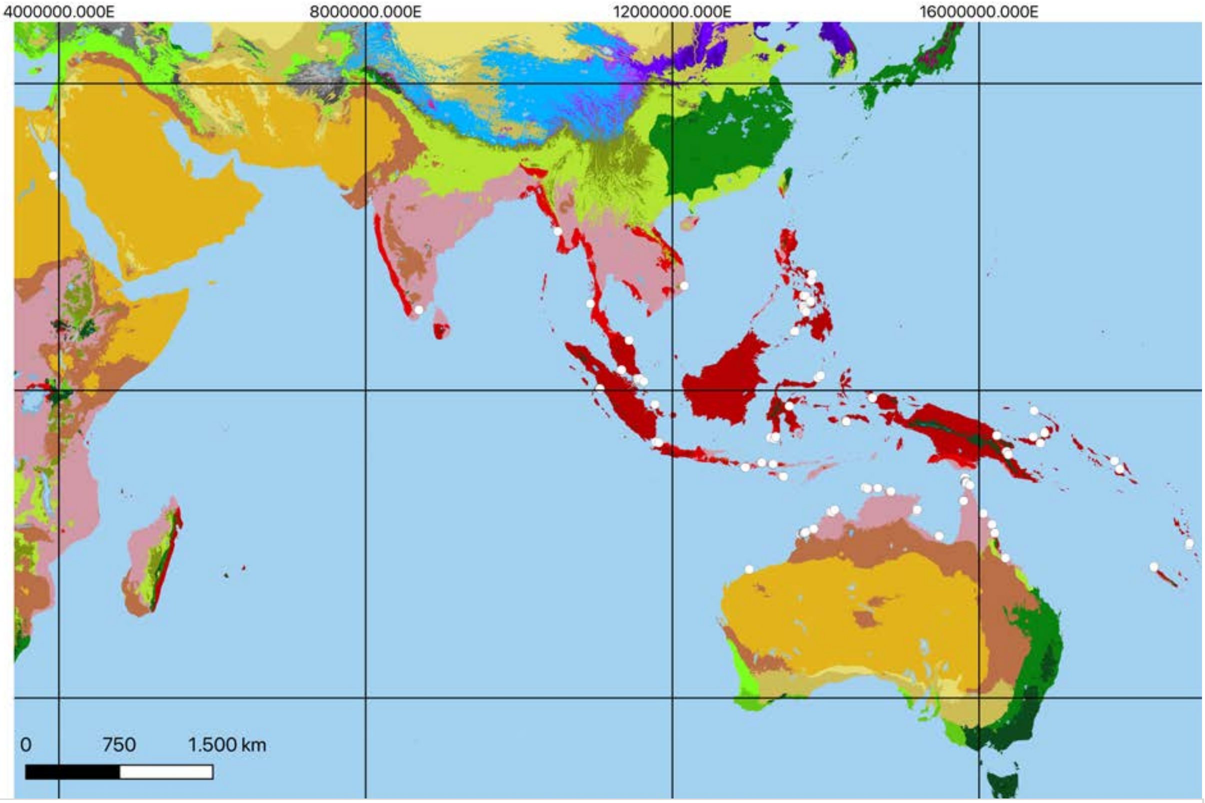

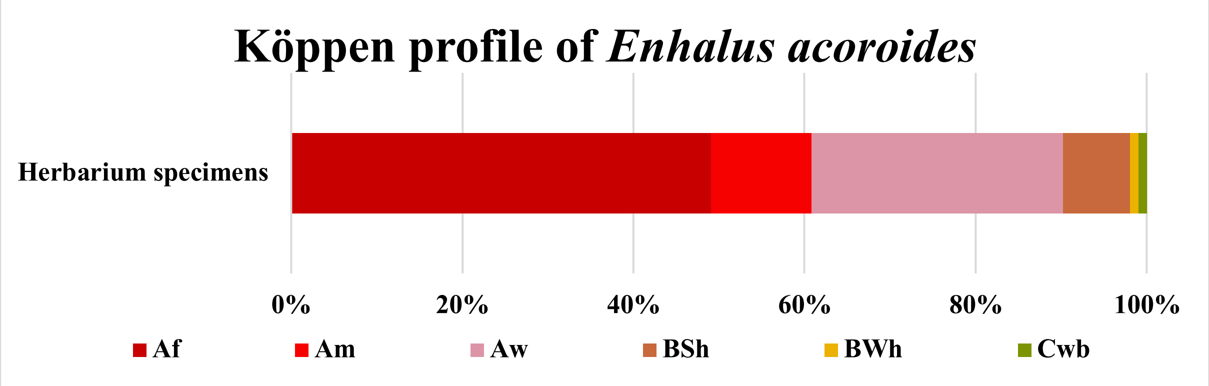

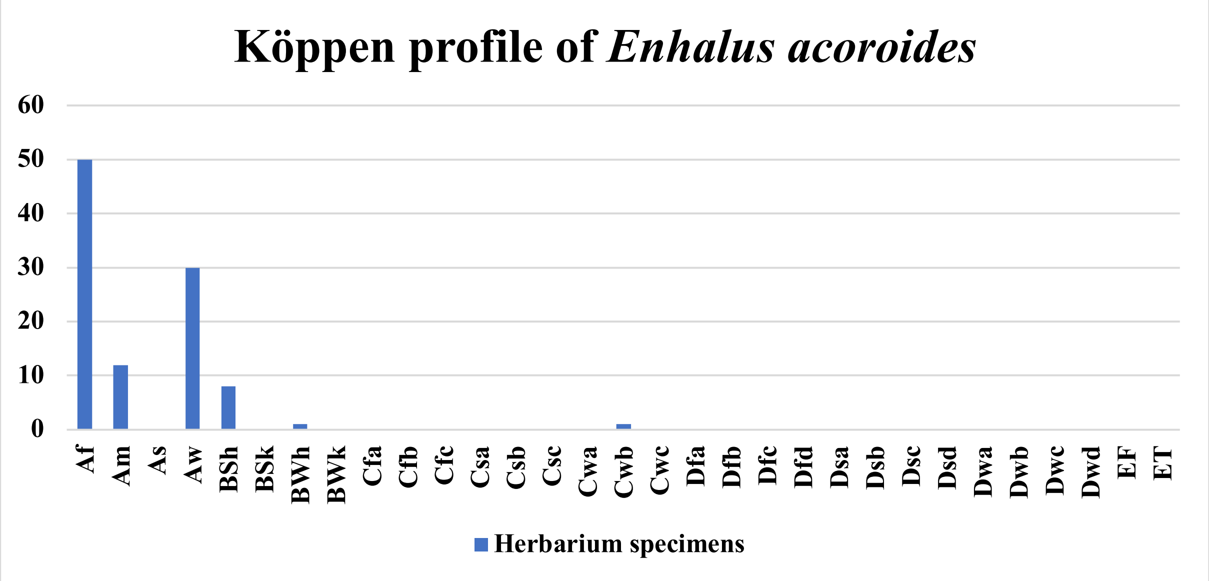

5.3.2.1. Genus *Halophila* Thouars 1806.

Distribution and Köppen profiles of genus *Halophila* incl. *H. australis, H. baillonis, H. beccari, H. capricorni, H. decipiens, H. engelmannii, H. gaudichaudii, H. hawaiiana, H. major, H. minor, H. nipponica, H. ovalis, H. spinulosa, H. stipulacea, H. sulawesii, and H. tricostata* (n = 1767).

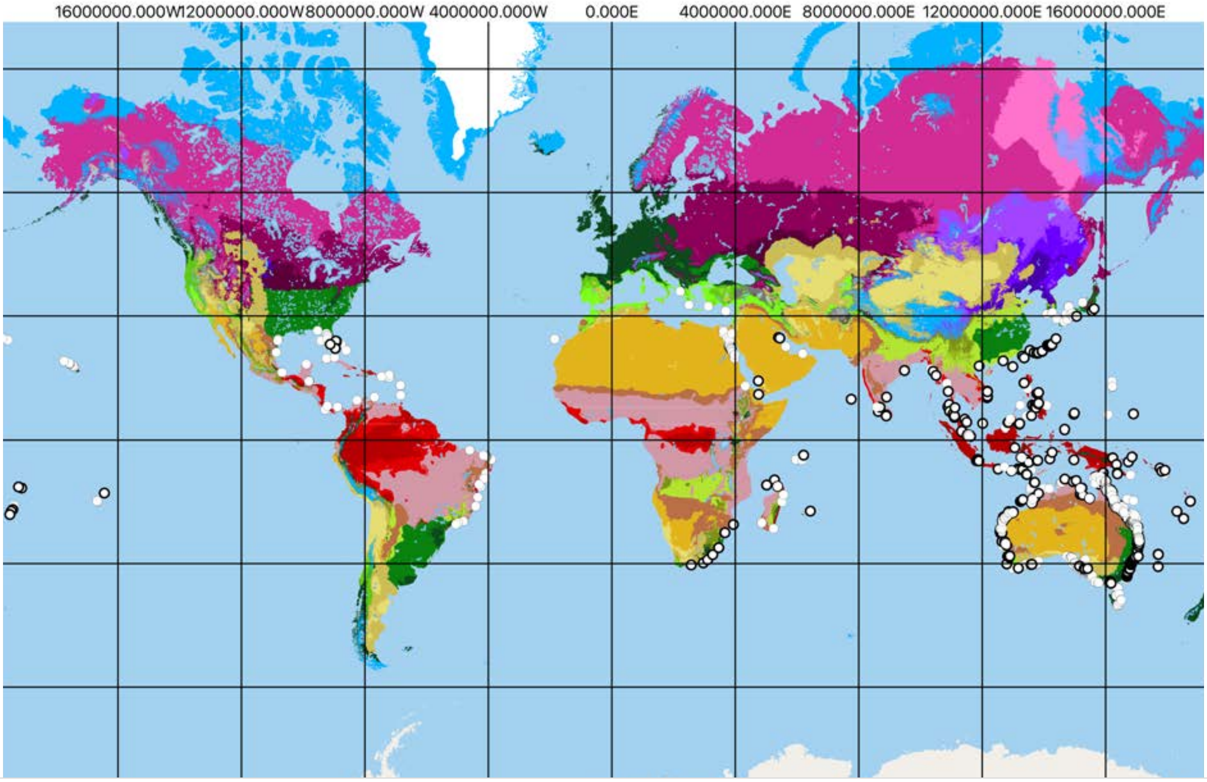

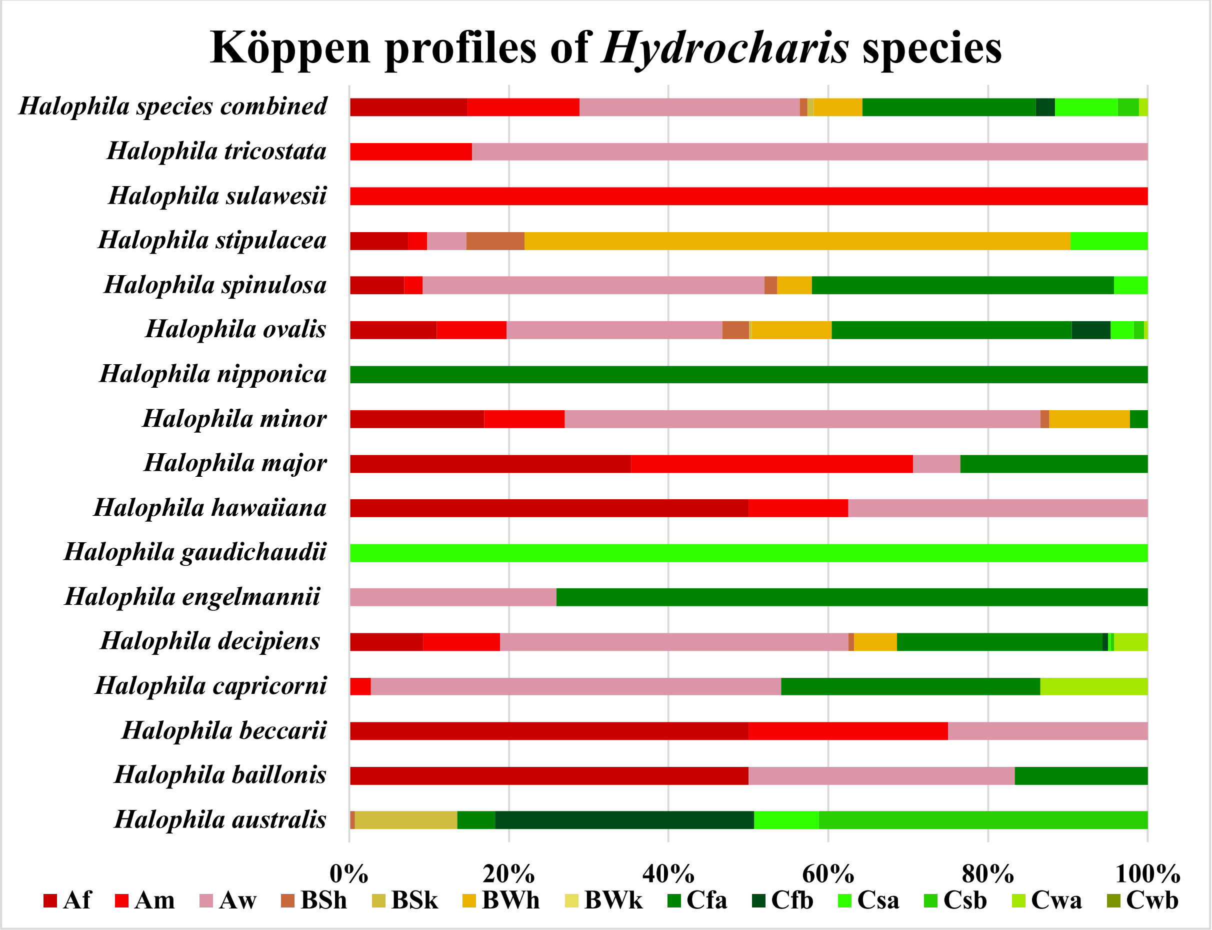

5.3.2.1.1. Köppen profile, distribution and climate map – GBIF occurrences of *Halophila australis* Doty & B.C.Stone 1967; herbarium specimens excluding duplicate occurrences (n = 148).

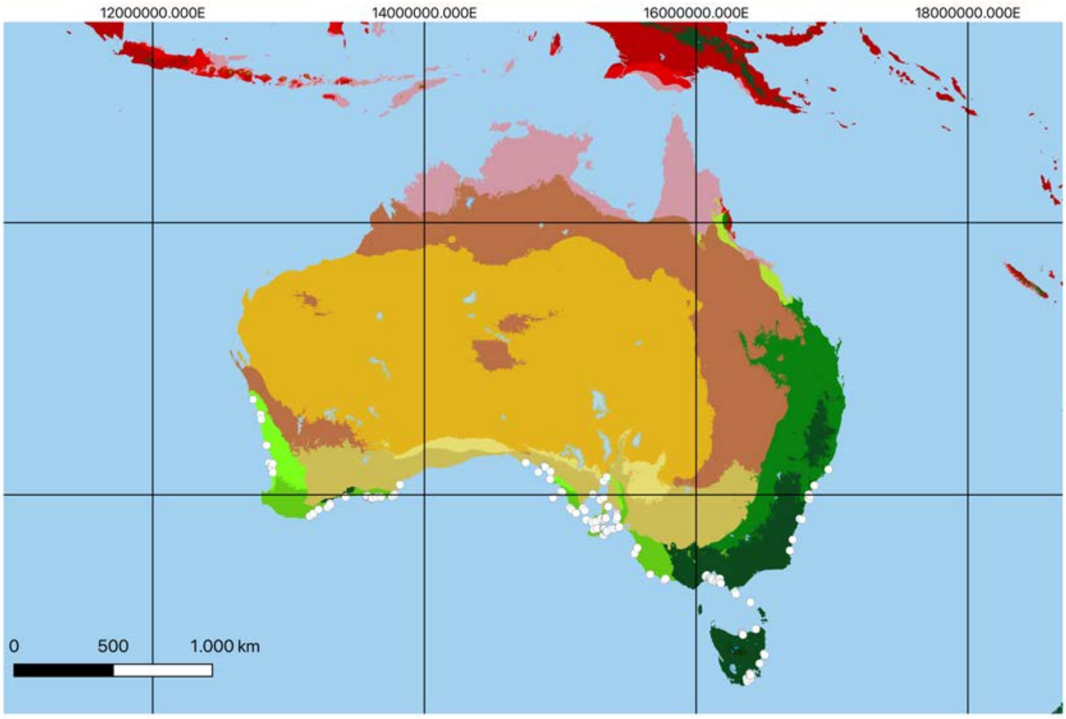

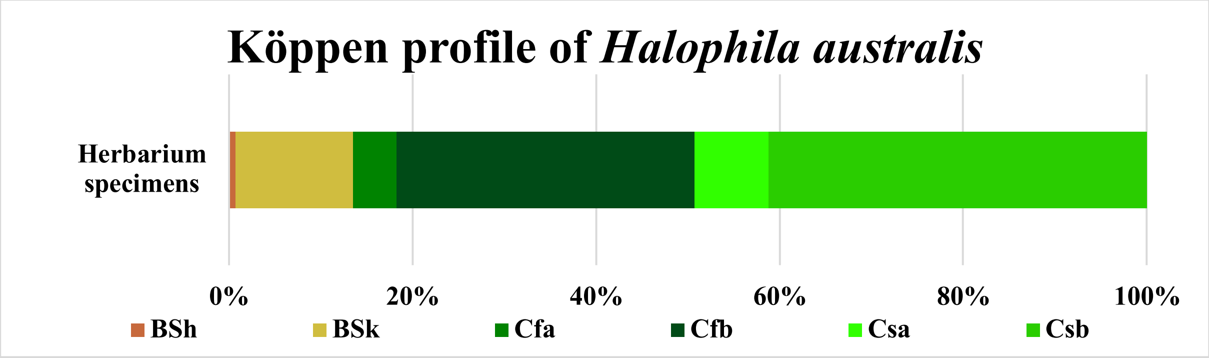

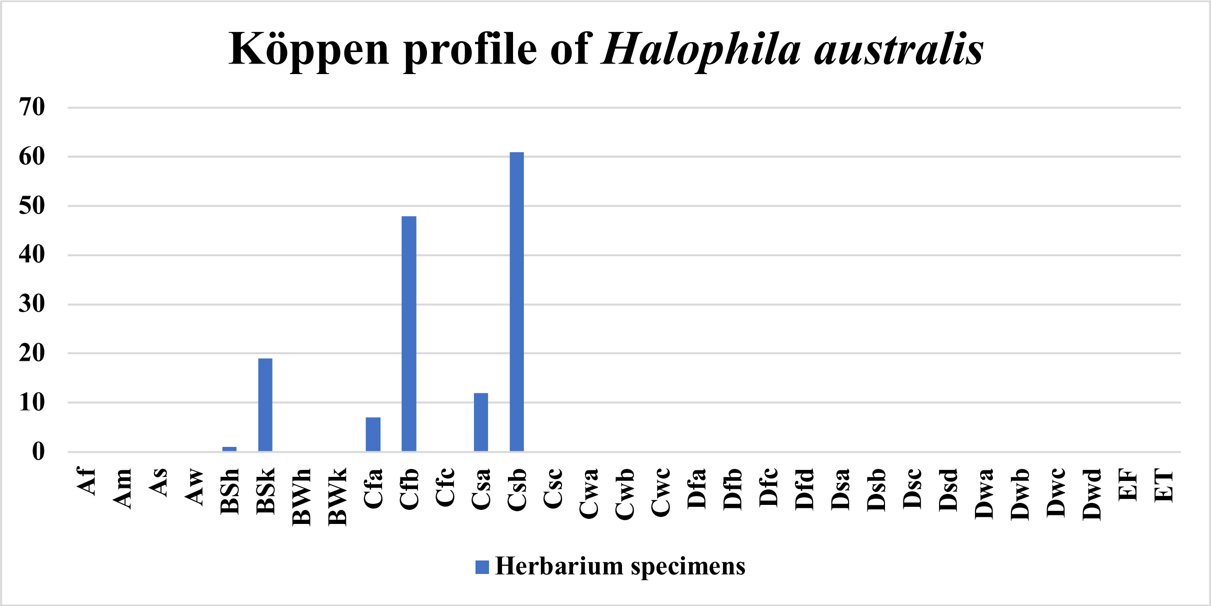
Köppen profile of *Halophila australis*
70
60
50
40
30
20
10
0
Af
Am
As
Aw
BSh
BSk
BWh
BWk
Cfa
Cfb
Cfc
Csa
Csb
Csc
Cwa
Cwb
Cwc
Dfa
Dfb
Dfc
Dfd
Dsa
Dsb
Dsc
Dsd
Dwa
Dwb
Dwc
Dwd
EF
ET
Herbarium specimens

5.3.2.1.2. Köppen profile, distribution and climate map – GBIF occurrences of *Halophila baillonis* Asch. ex Dickie 1874; herbarium specimens excluding duplicate occurrences (n = 6).

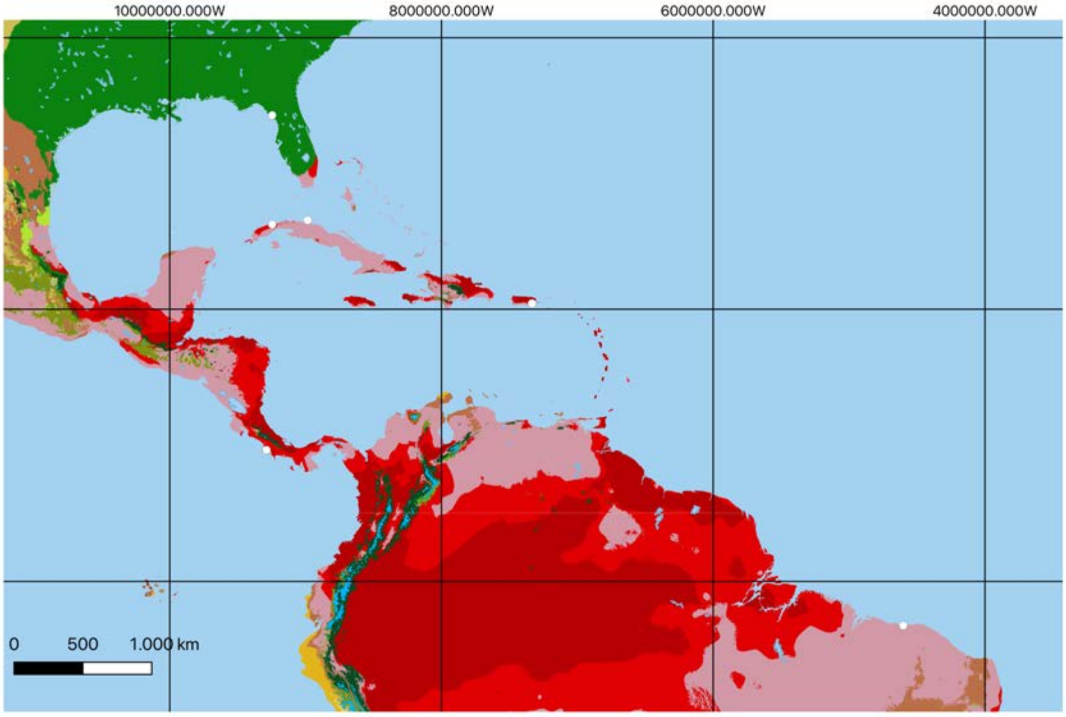

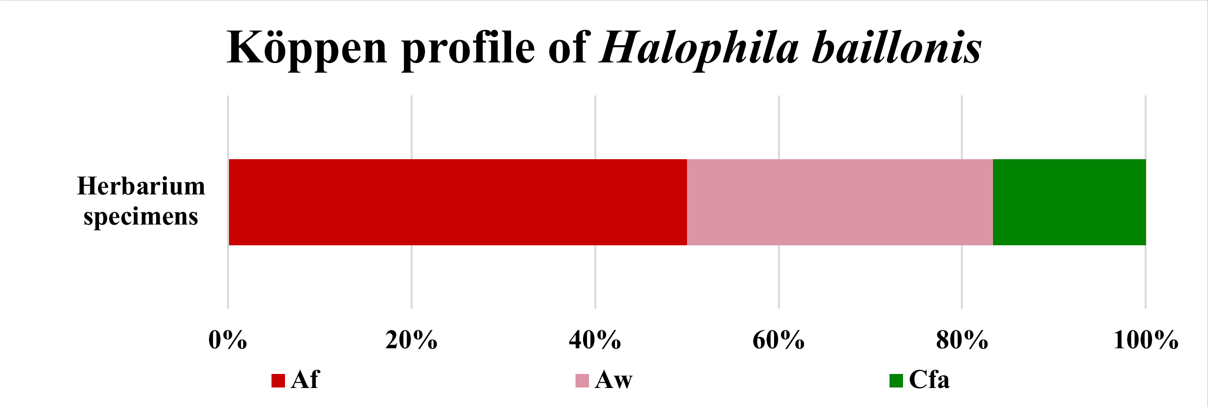

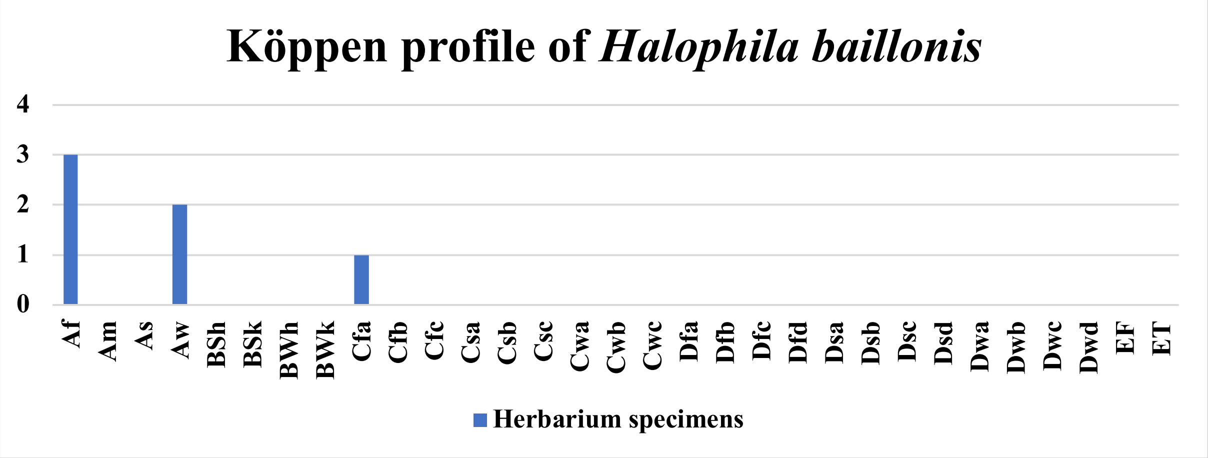

5.3.2.1.3. Köppen profile, distribution and climate map – GBIF occurrences of *Halophila beccarii* Asch. 1874; herbarium specimens excluding duplicate occurrences (n = 8).

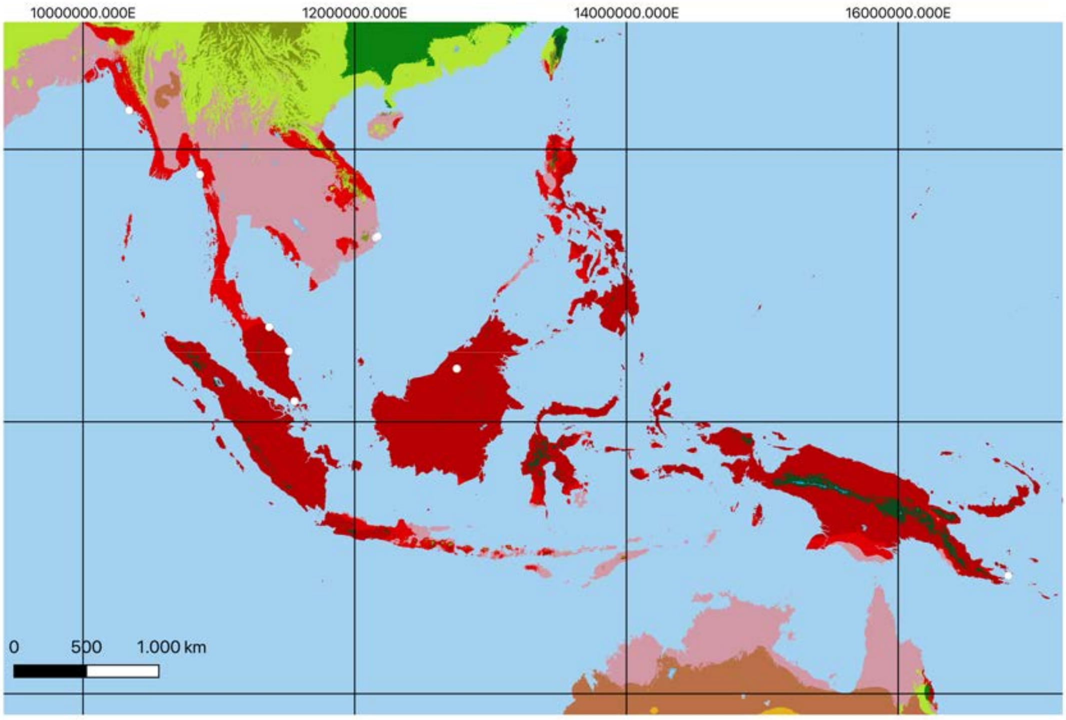

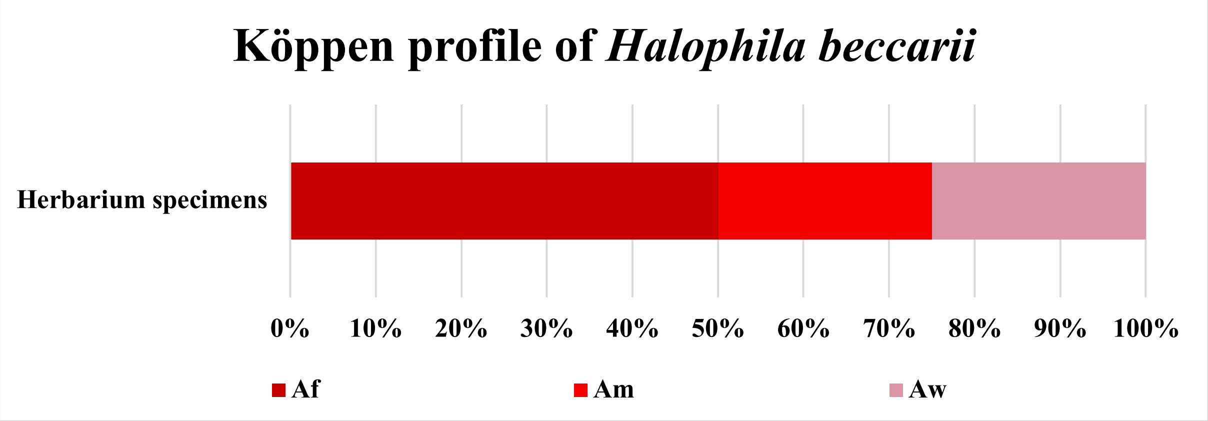

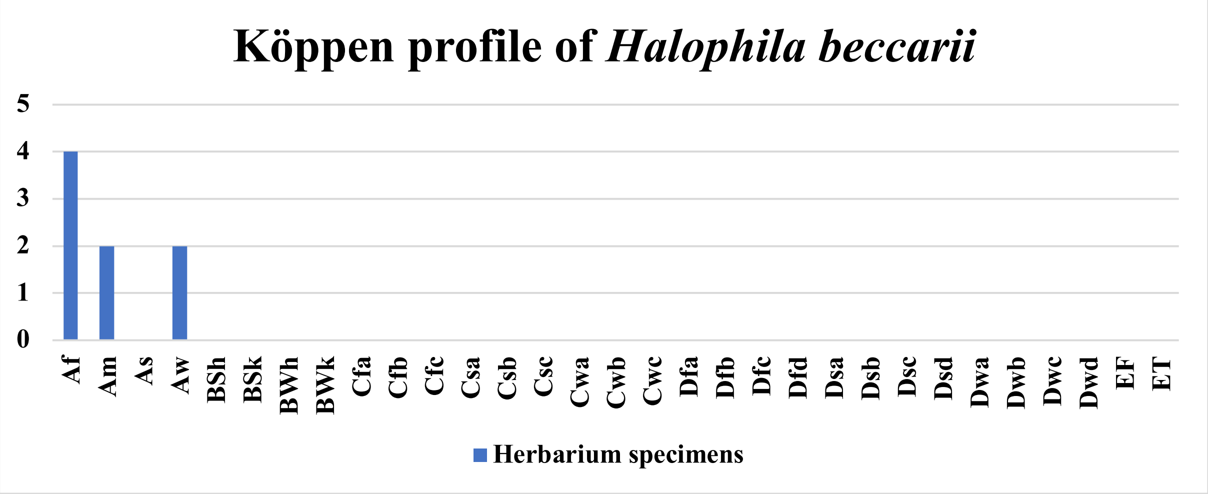

5.3.2.1.4. Köppen profile, distribution and climate map – GBIF occurrences of *Halophila capricorni* Larkum 1995; herbarium specimens excluding duplicate occurrences (n = 37).

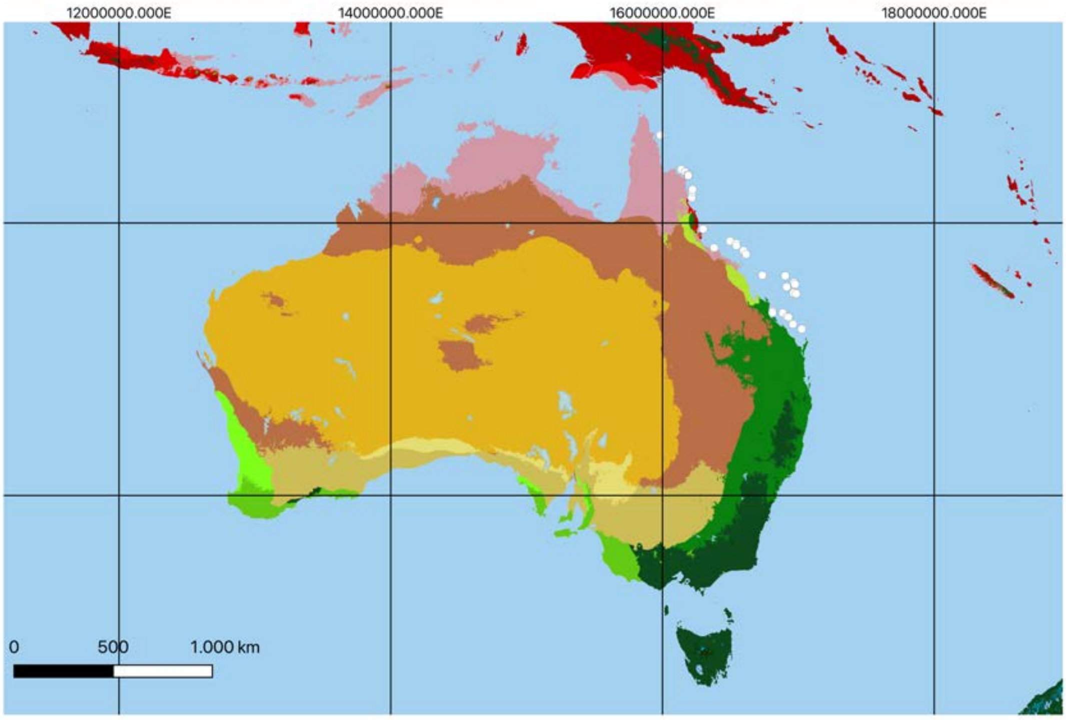

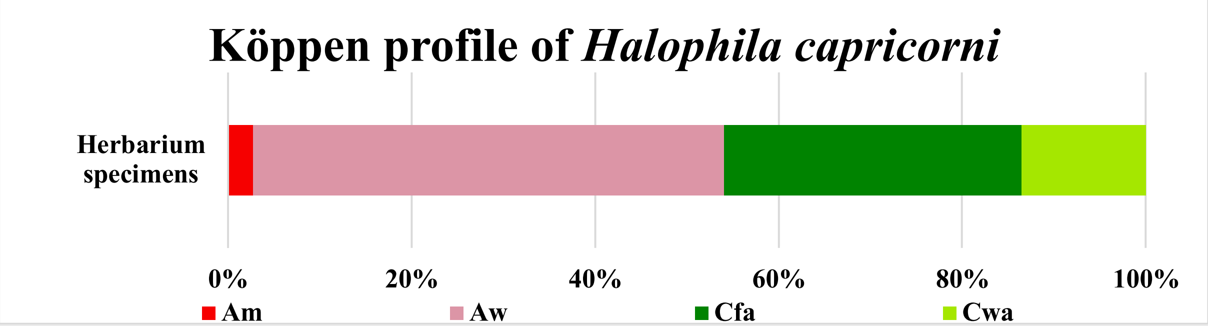

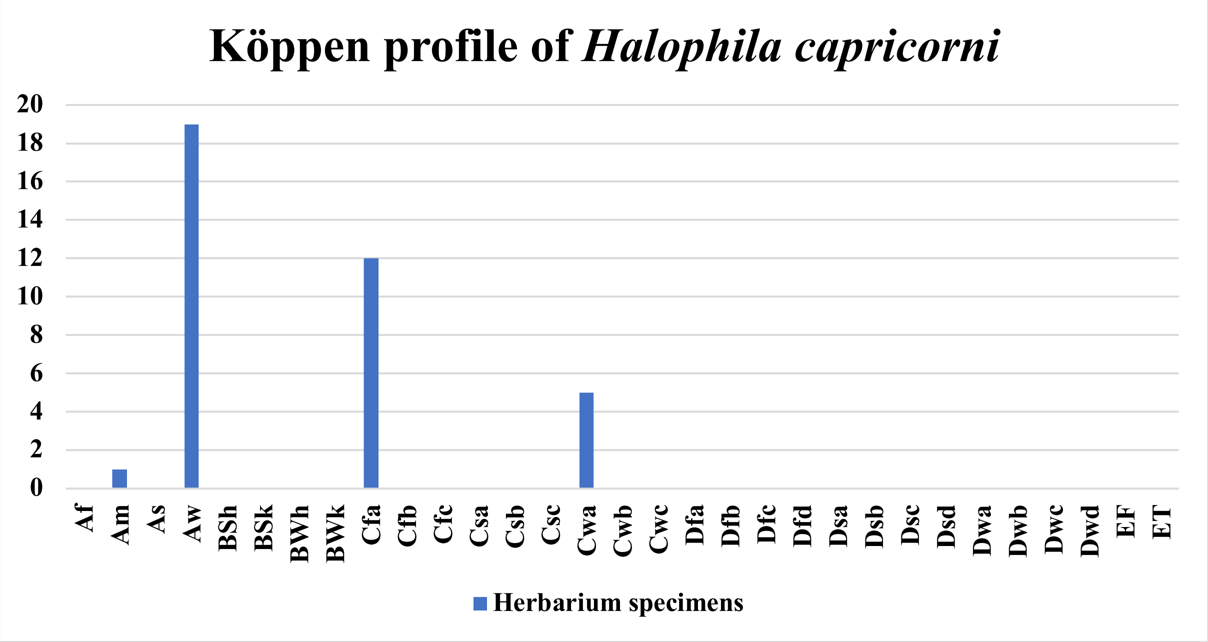

5.3.2.1.5. Köppen profile, distribution and climate map – GBIF occurrences of *Halophila decipiens* Ostenf. 1902; herbarium specimens excluding duplicate occurrences (n = 280).

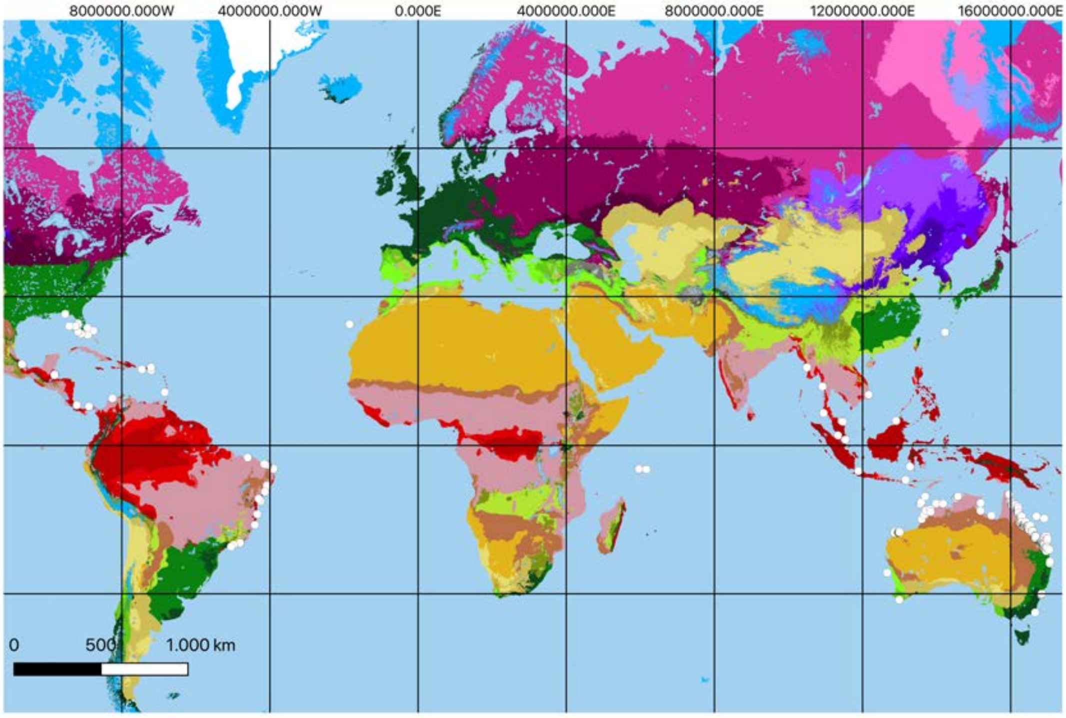

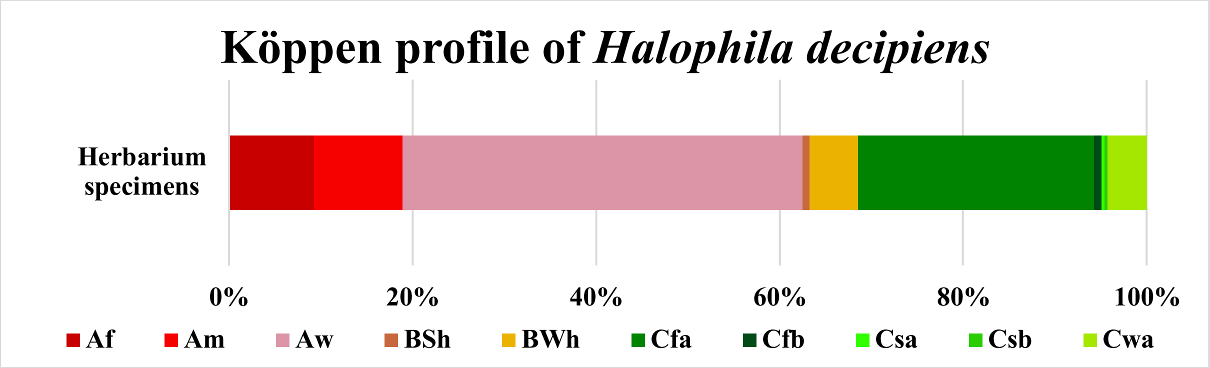

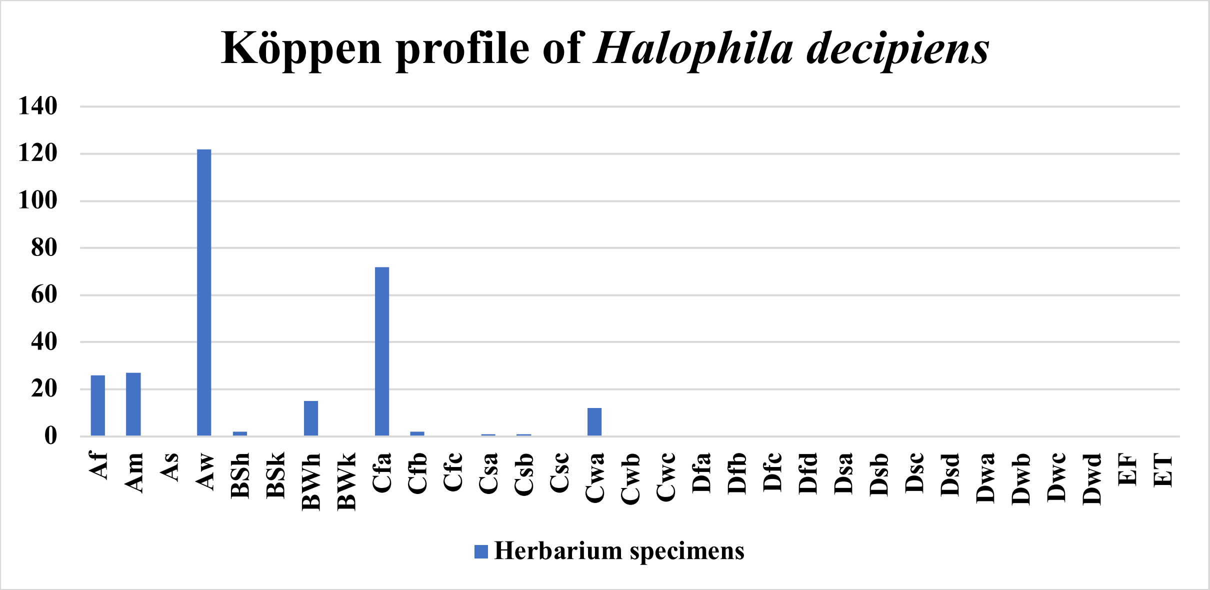

5.3.2.1.6. Köppen profile, distribution and climate map – GBIF occurrences of *Halophila engelmannii* Asch. 1974; herbarium specimens excluding duplicate occurrences (n = 27).

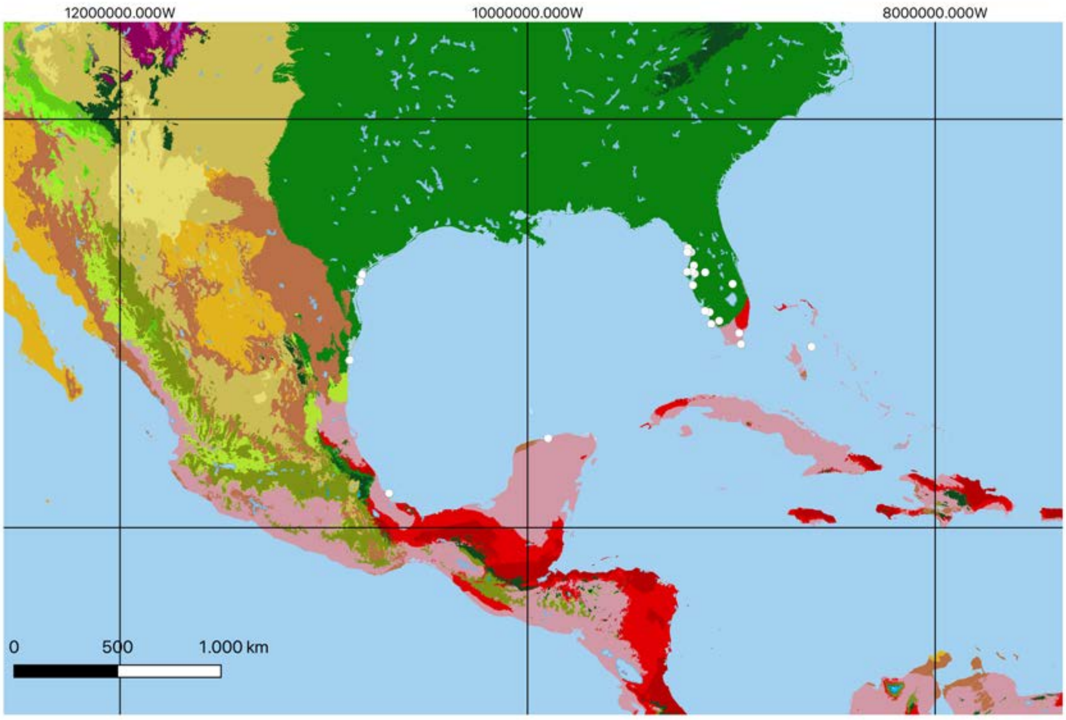

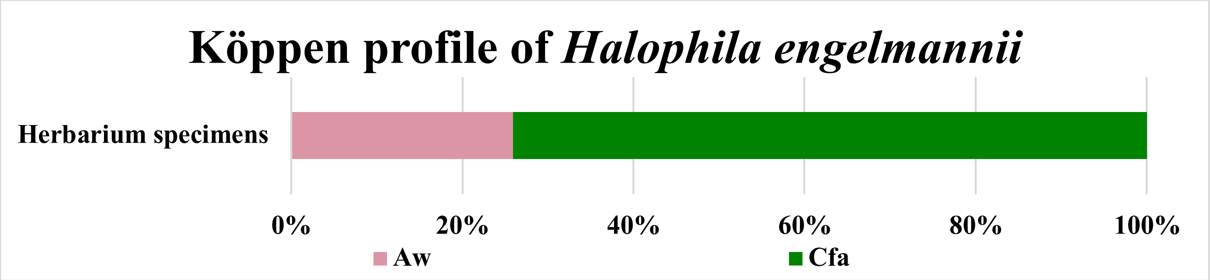

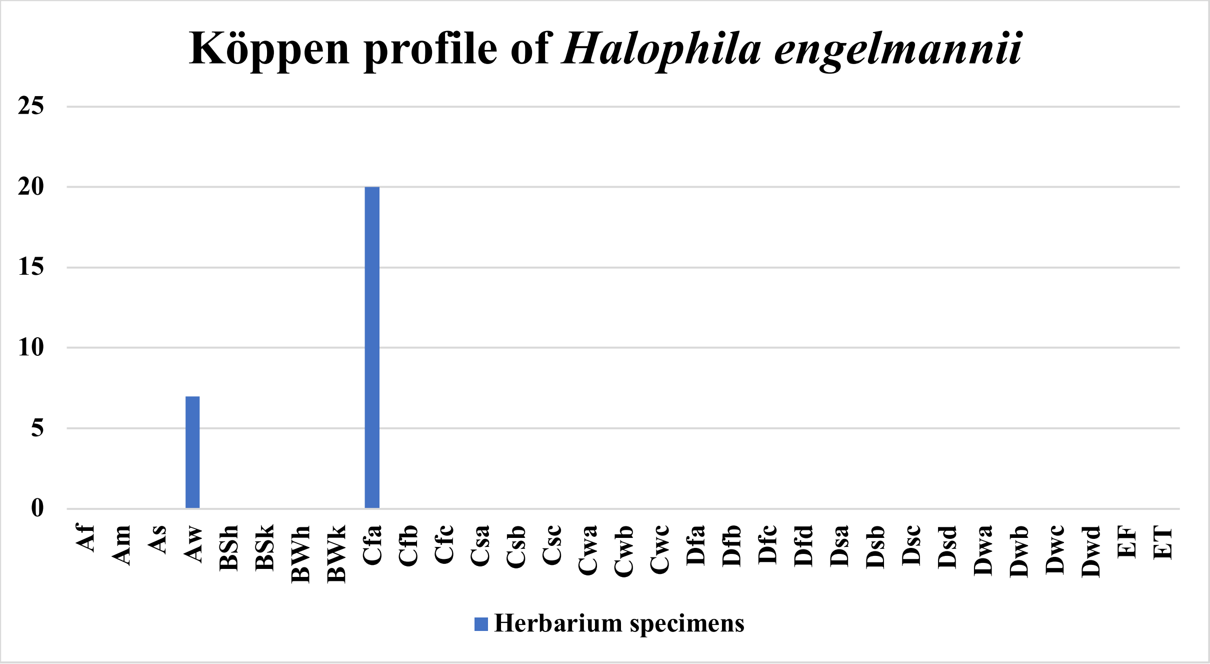

5.3.2.1.7. Köppen profile, distribution and climate map – GBIF occurrences of *Halophila gaudichaudii* J.Kuo 2006; herbarium specimens excluding duplicate occurrences (n = 4).

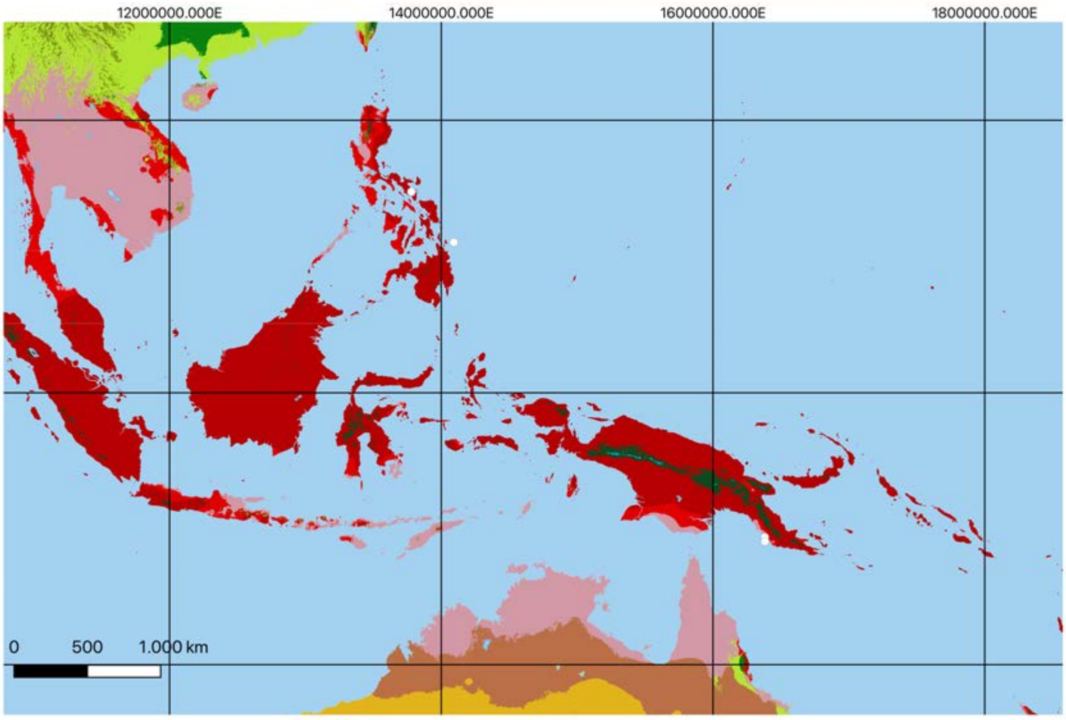

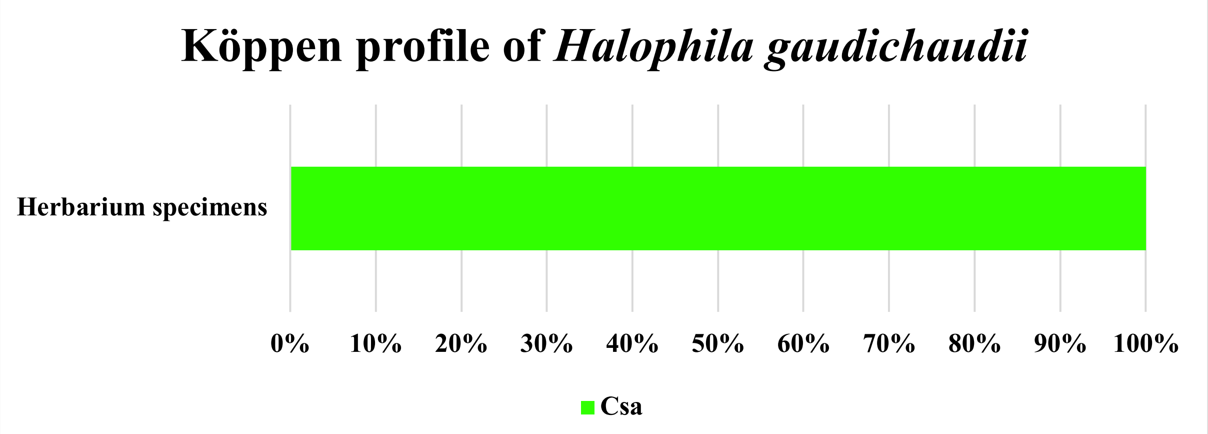

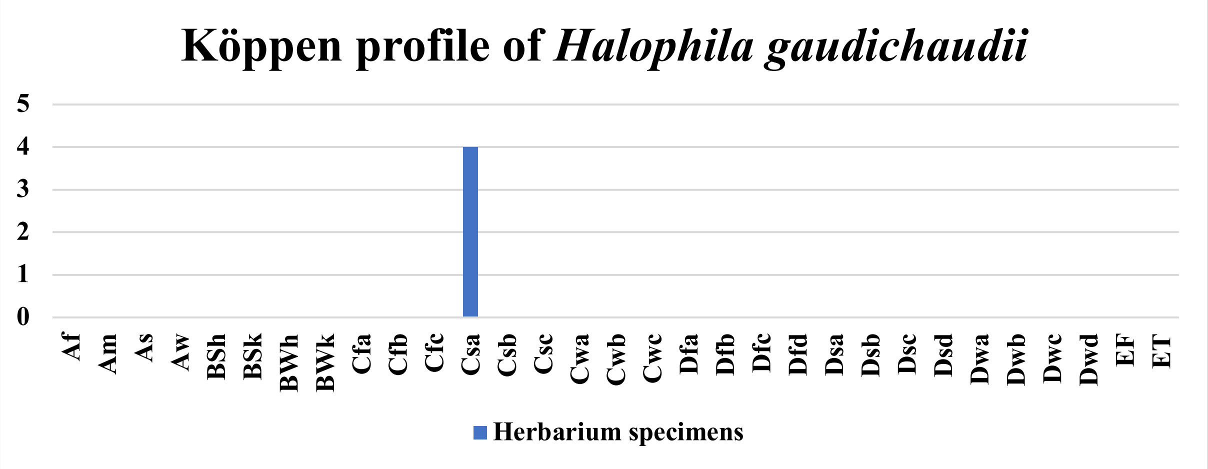

5.3.2.1.8. Köppen profile, distribution and climate map – GBIF occurrences of *Halophila hawaiiana* Kuo 2006; herbarium specimens excluding duplicate occurrences (n = 8).

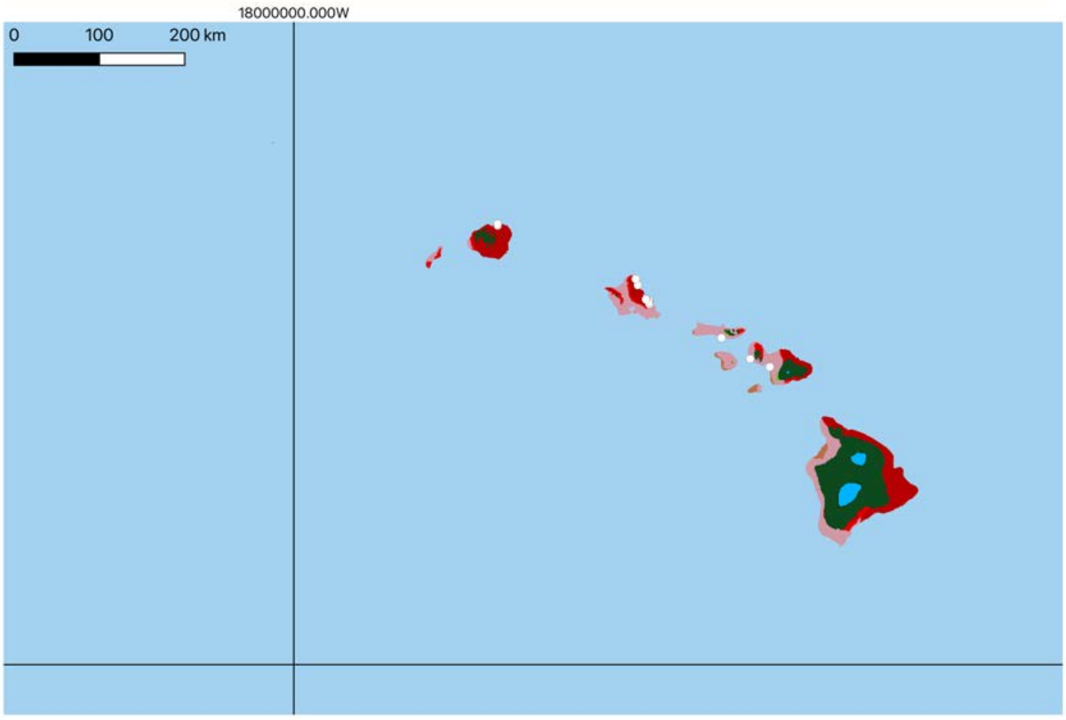

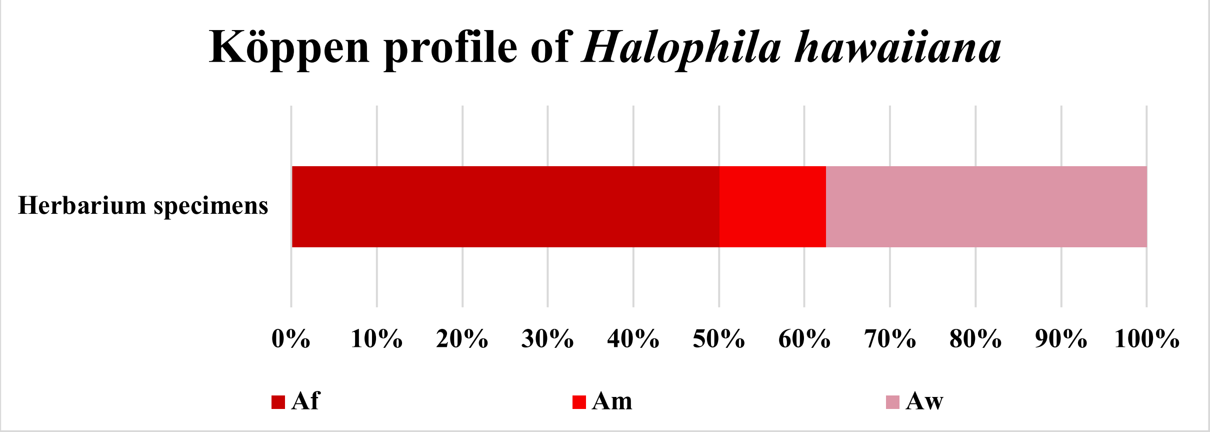

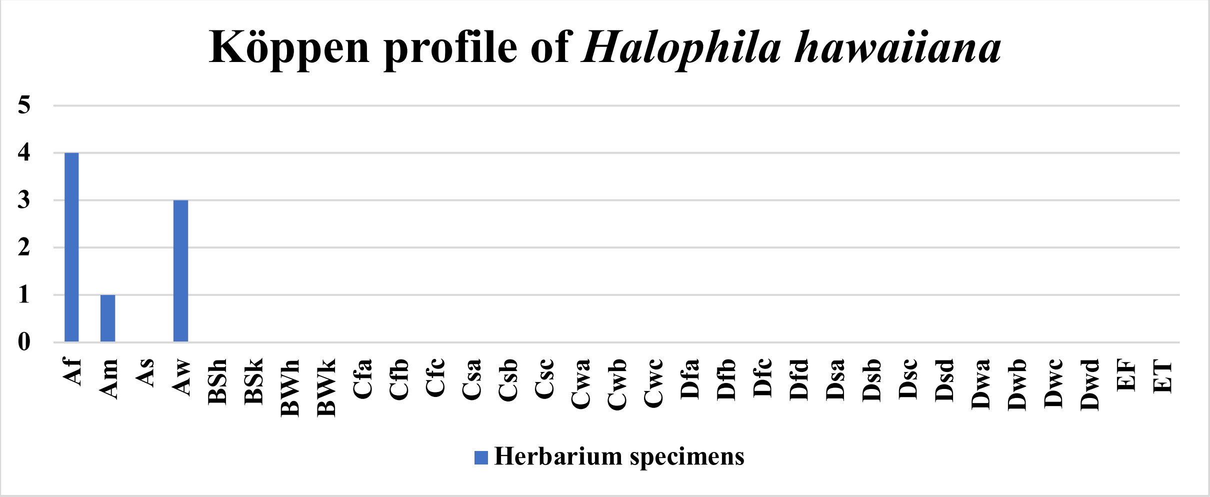

5.3.2.1.9. Köppen profile, distribution and climate map – GBIF occurrences of *Halophila major* Mirq. 1856; herbarium specimens excluding duplicate occurrences (n = 17).

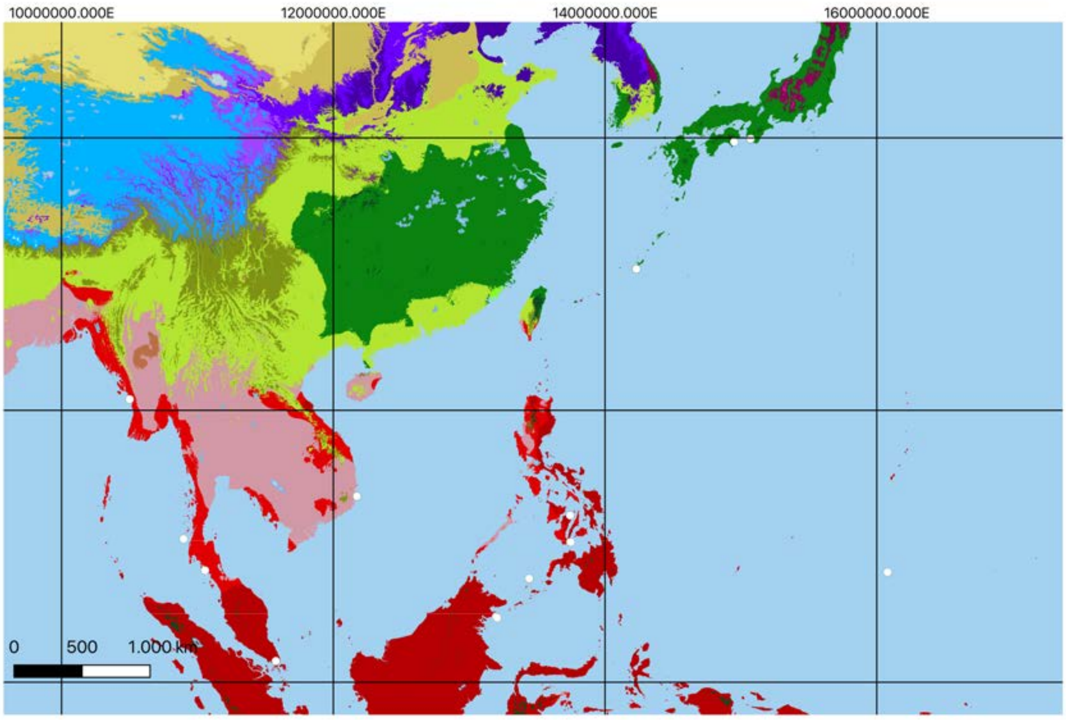

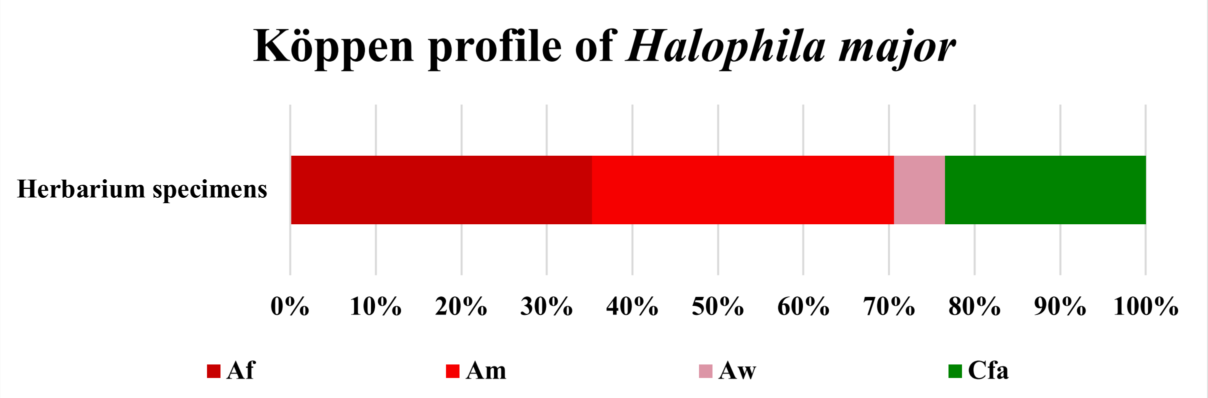

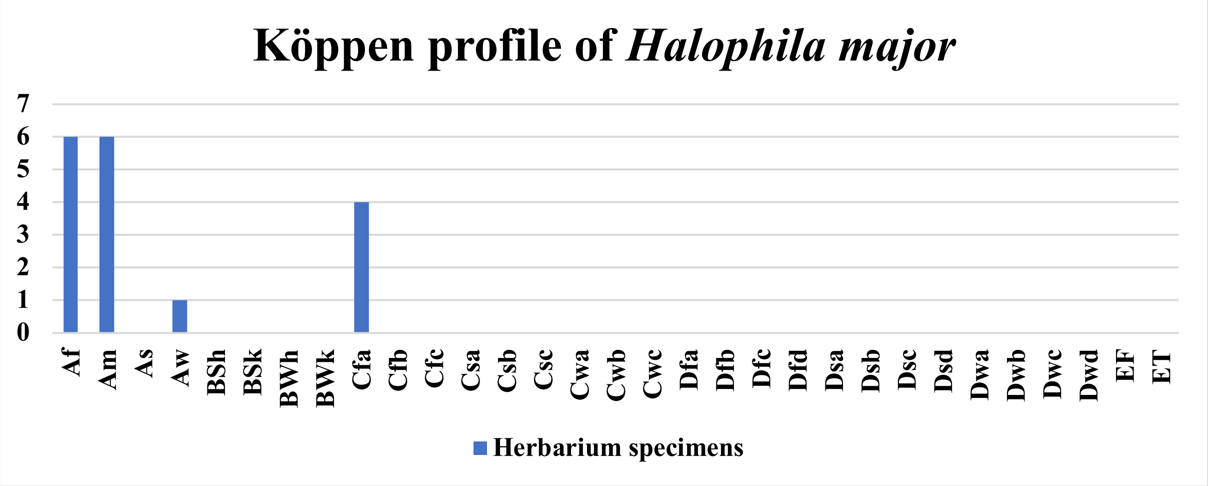

5.3.2.1.10. Köppen profile, distribution and climate map – GBIF occurrences of *Halophila minor* (Zoll.) Hartog 1957; herbarium specimens excluding duplicate occurrences (n = 89).

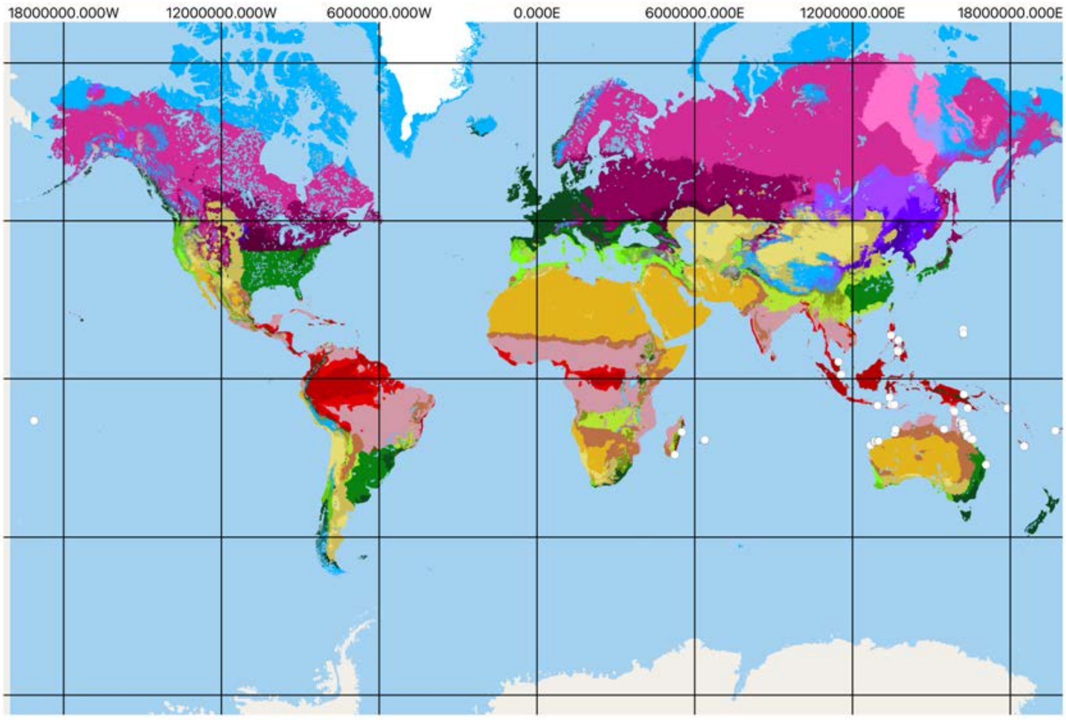

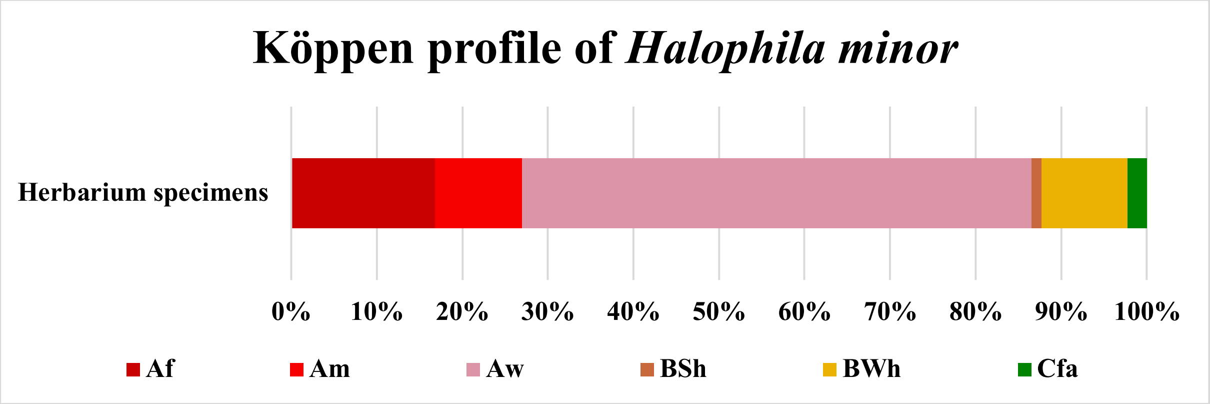

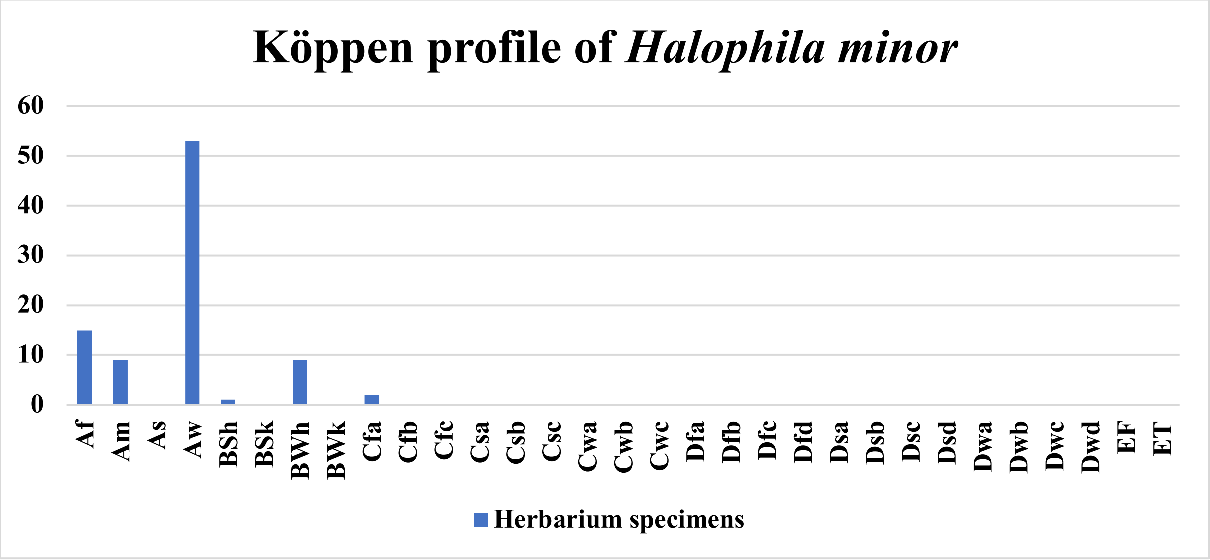

5.3.2.1.11. Köppen profile, distribution and climate map – GBIF occurrences of *Halophila nipponica* J.Kuo 2006; herbarium specimens excluding duplicate occurrences (n = 16).

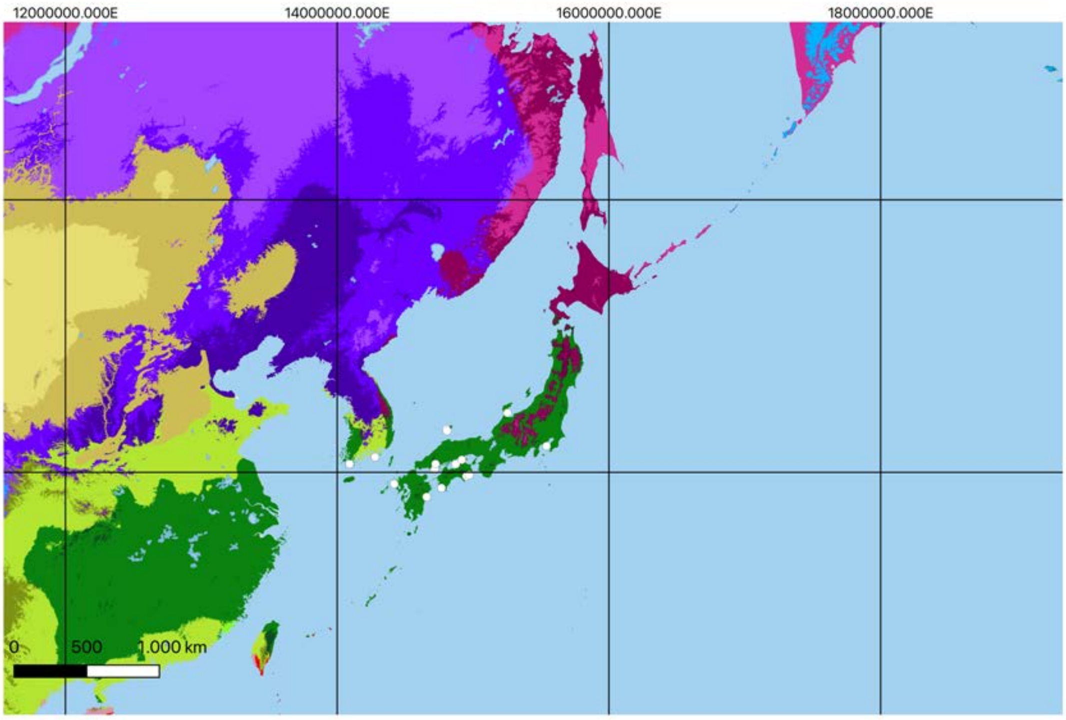

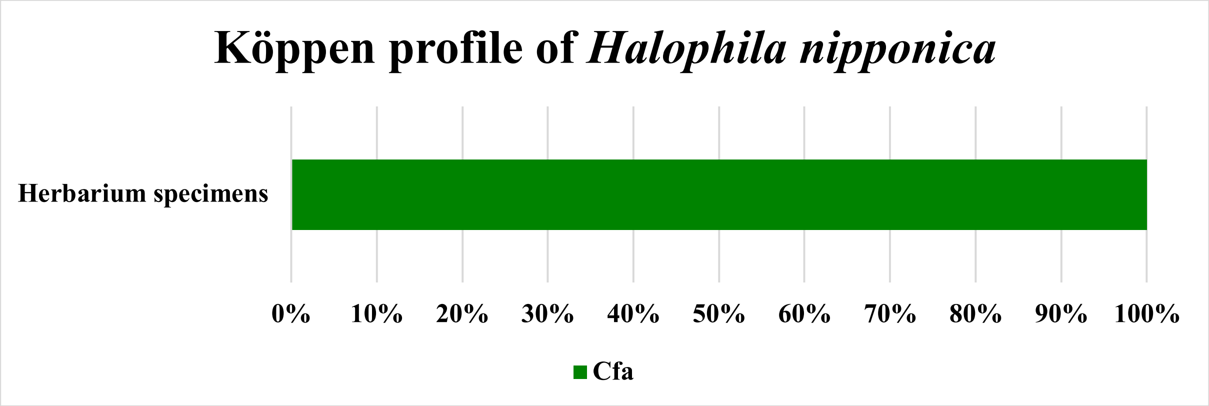

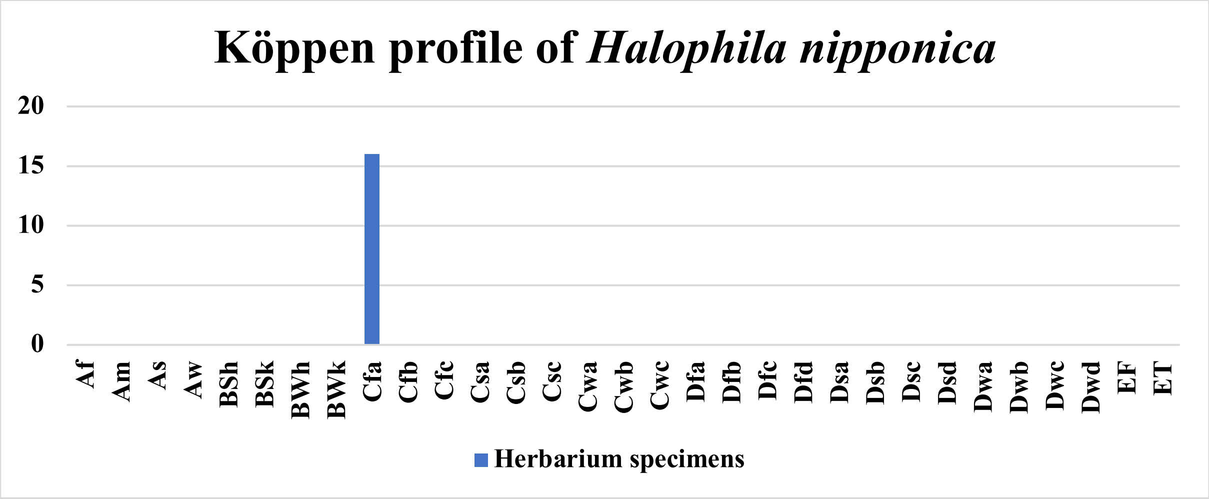

5.3.2.1.11. Köppen profile, distribution and climate map – GBIF occurrences of *Halophila ovalis* (R.Br. 1810) Hook.f. 1859; herbarium specimens excluding duplicate occurrences (n = 619).

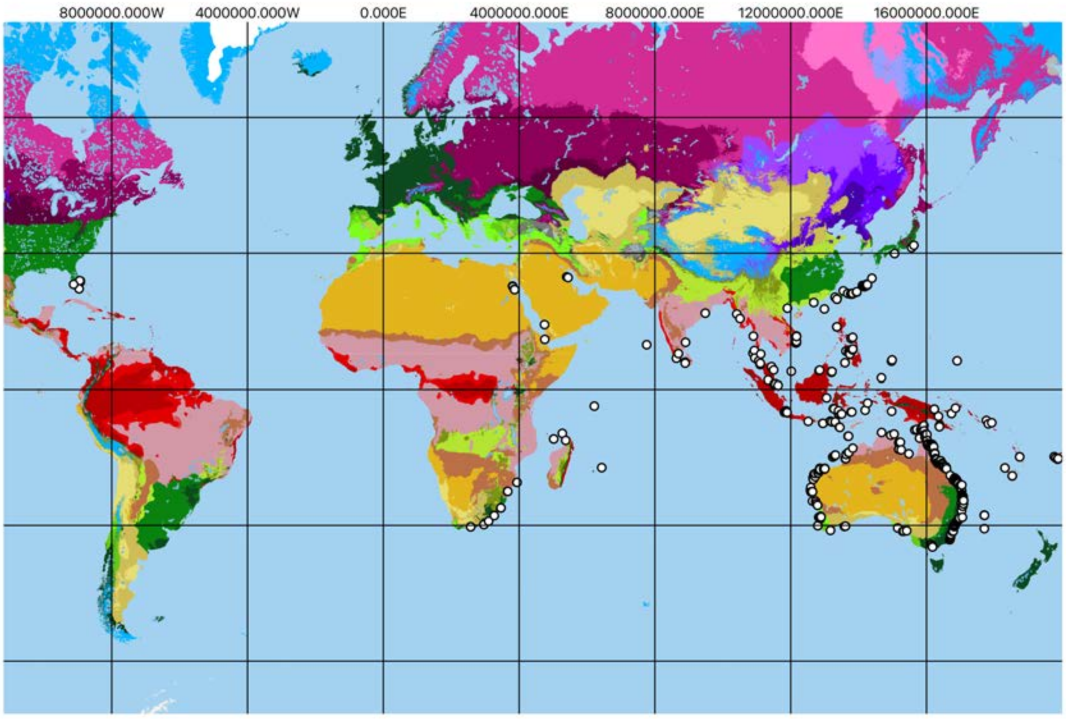

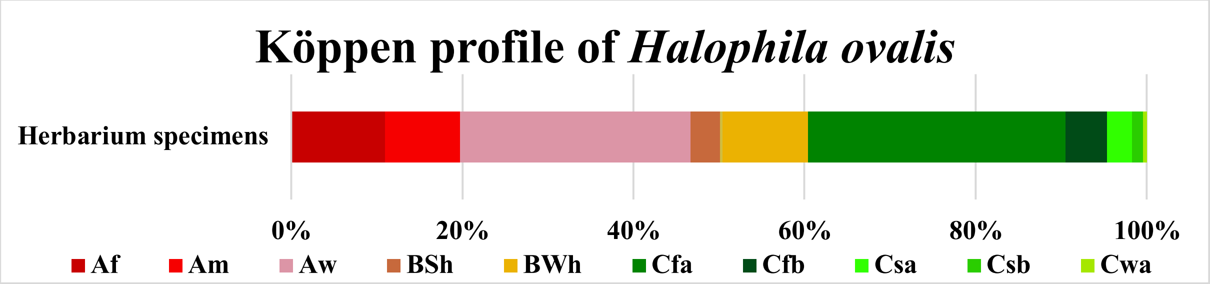

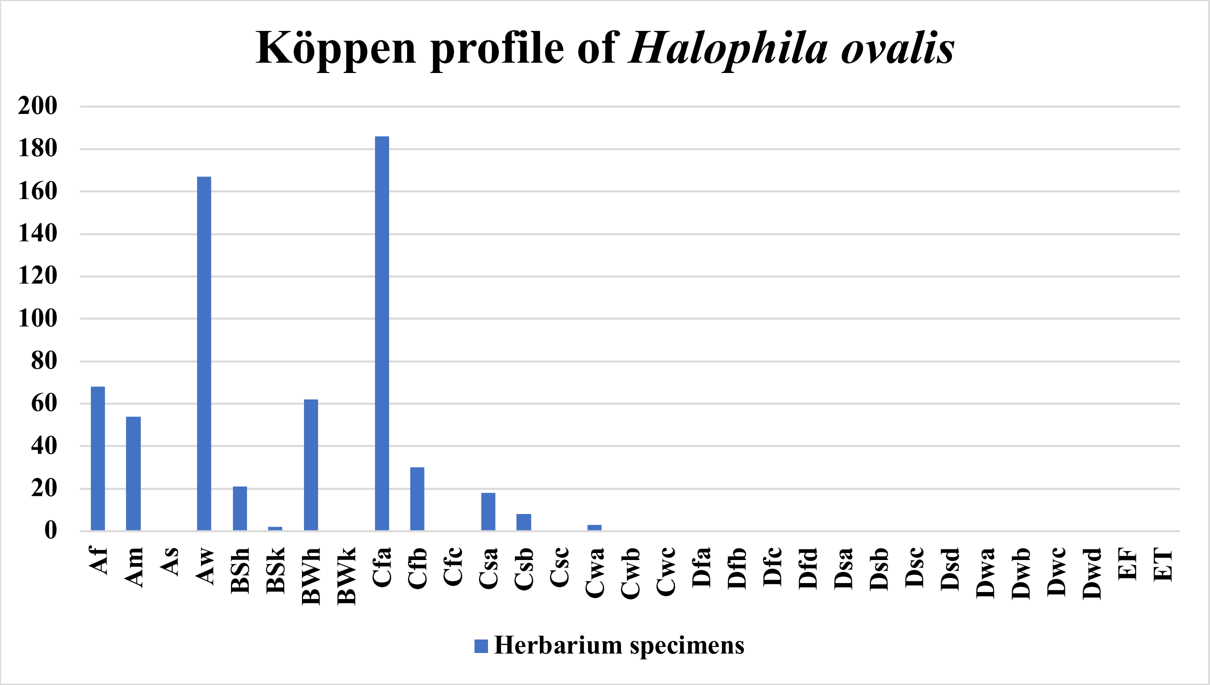

Köppen profile of *Halophila ovalis*
200
180
160
140
120
100
80
60
40
20
0
Af
Am
As
Aw
BSh
BSk
BWh
BWk
Cfa
Cfb
Cfc
Csa
Csb
Csc
Cwa
Cwb
Cwc
Dfa
Dfb
Dfc
Dfd
Dsa
Dsb
Dsc
Dsd
Dwa
Dwb
Dwc
Dwd
EF
ET
Herbarium specimens

5.3.2.1.12. Köppen profile, distribution and climate map – GBIF occurrences of *Halophila spinulosa* (R.Br. 1810) Asch. 1875; herbarium specimens excluding duplicate occurrences (n = 304).

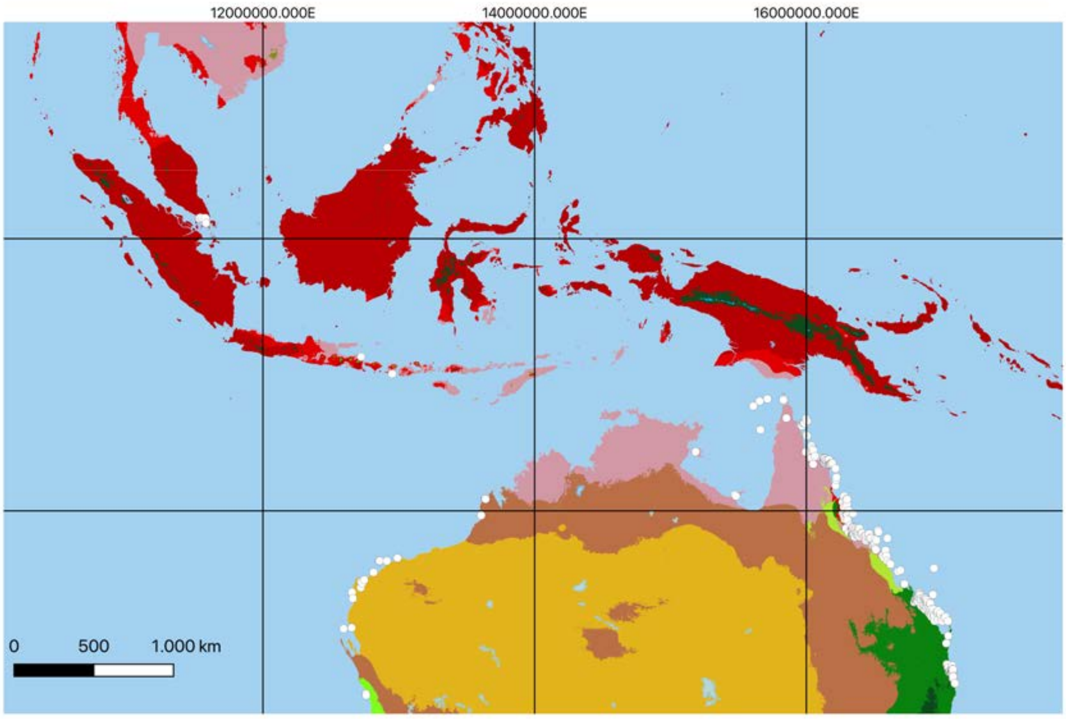

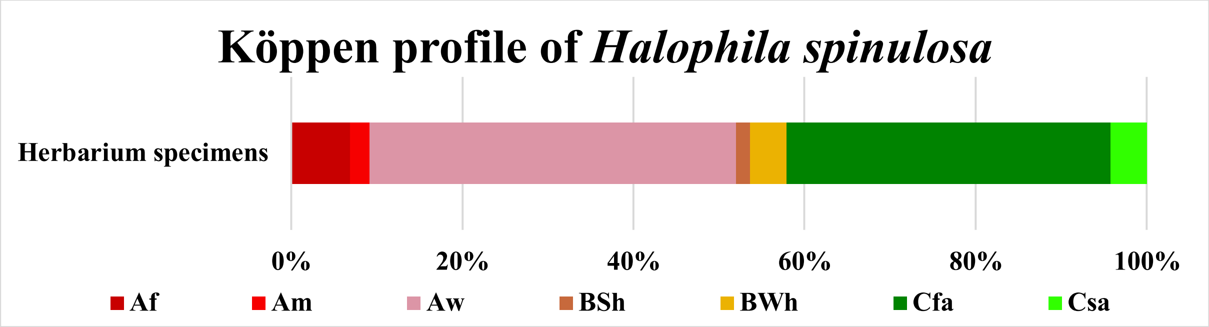

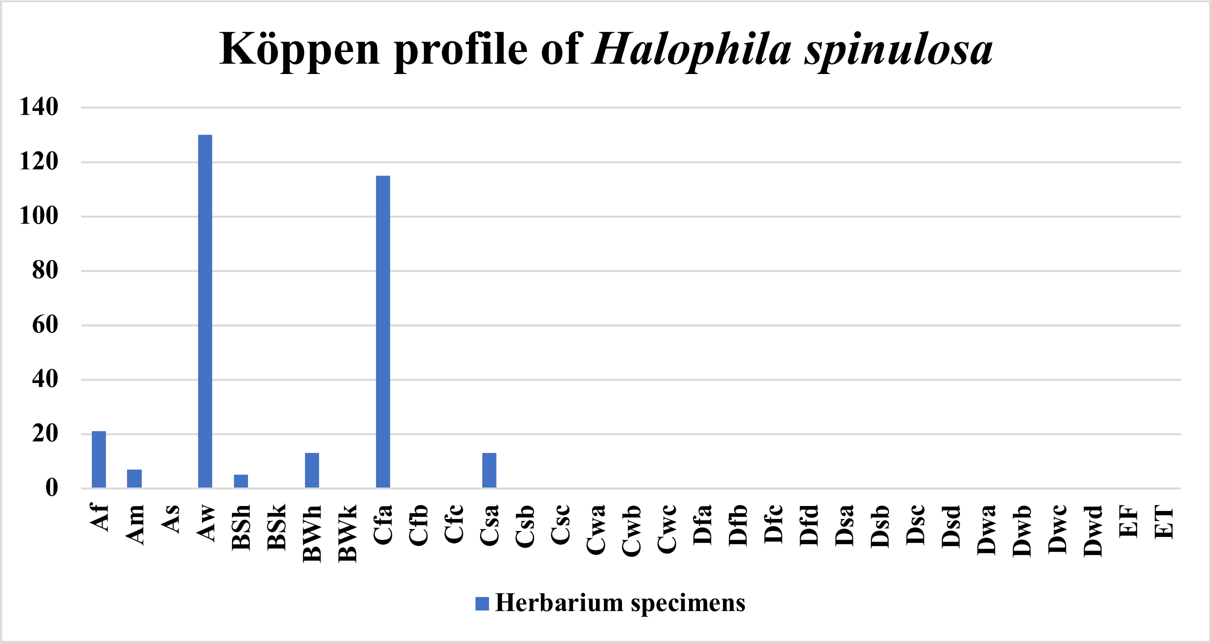

Köppen profile of *Halophila spinulosa*
140
120
100
80
60
40
20
0
Af
Am
As
Aw
BSh
BSk
BWh
BWk
Cfa
Cfb
Cfc
Csa
Csb
Csc
Cwa
Cwb
Cwc
Dfa
Dfb
Dfc
Dfd
Dsa
Dsb
Dsc
Dsd
Dwa
Dwb
Dwc
Dwd
EF
ET
Herbarium specimens

5.3.2.1.13. Köppen profile, distribution and climate map – GBIF occurrences of *Halophila stipulacea* (Forssk.) Asch. 1867; herbarium specimens excluding duplicate occurrences (n = 41).

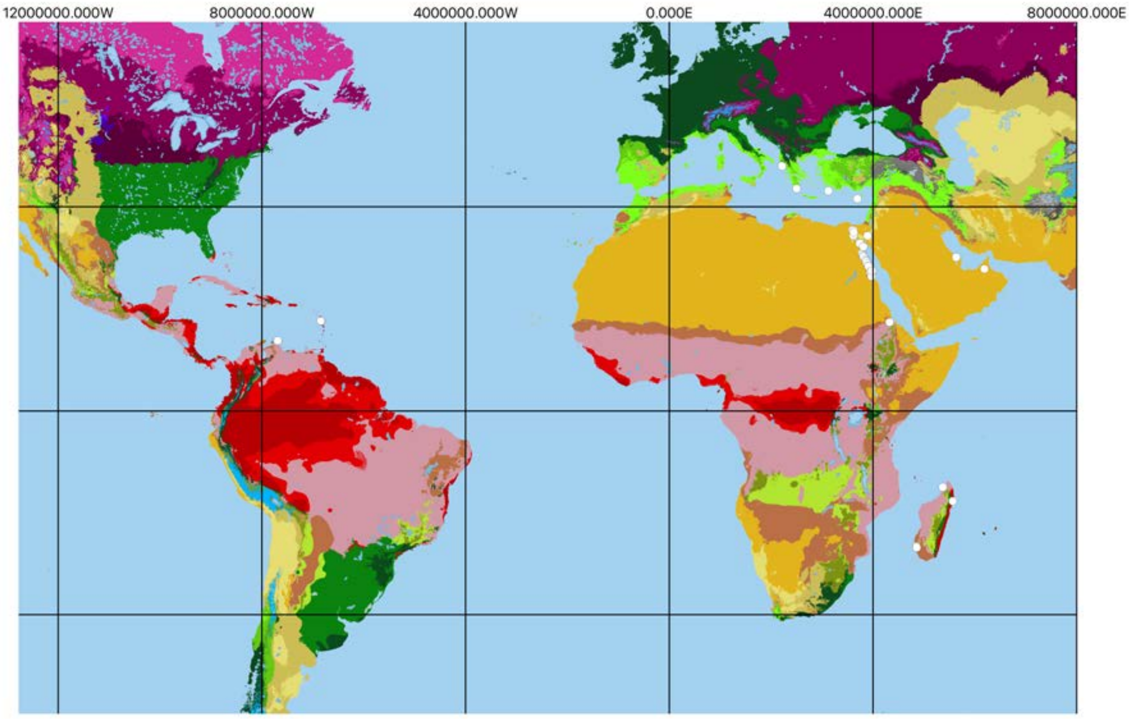

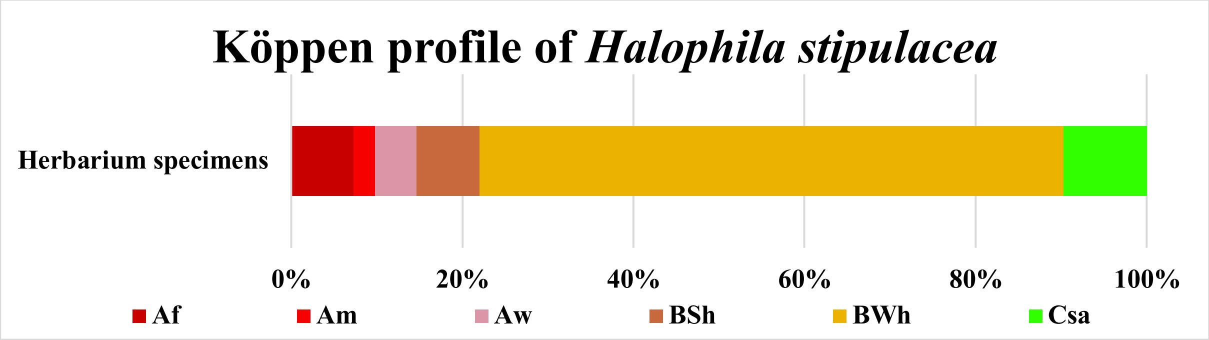

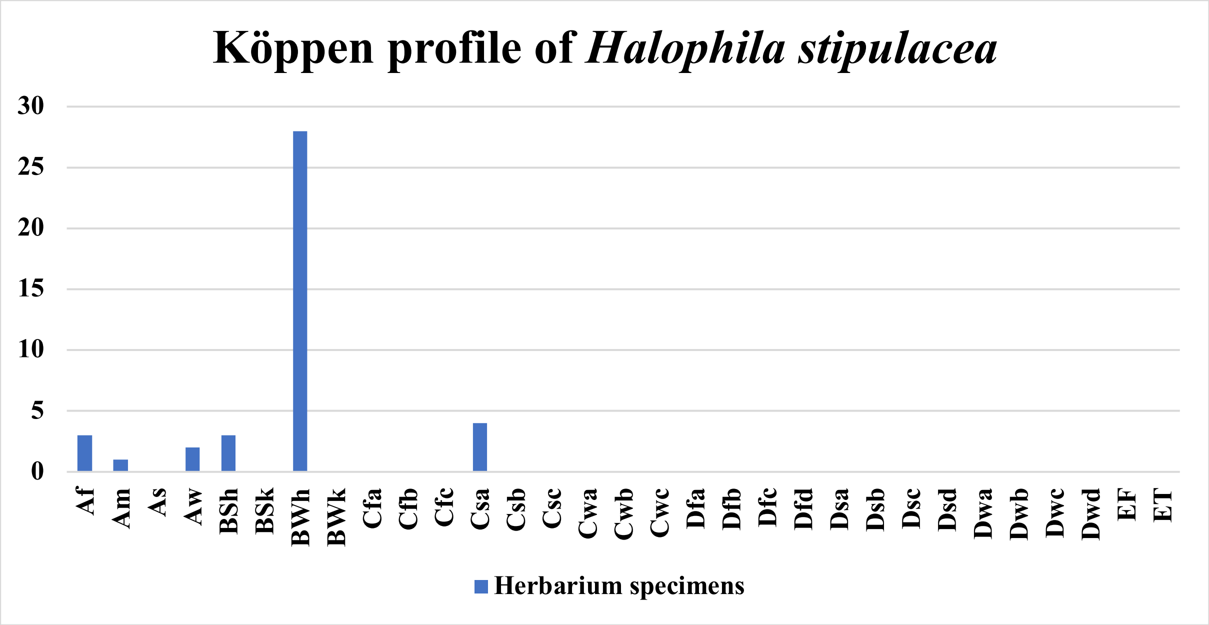

Köppen profile of *Halophila stipulacea*
30
25
20
15
10
5
0
Af
Am
As
Aw
BSh
BSk
BWh
BWk
Cfa
Cfb
Cfc
Csa
Csb
Csc
Cwa
Cwb
Cwc
Dfa
Dfb
Dfc
Dfd
Dsa
Dsb
Dsc
Dsd
Dwa
Dwb
Dwc
Dwd
EF
ET
Herbarium specimens

5.3.2.1.14. Köppen profile, distribution and climate map – GBIF occurrences of *Halophila sulawesii* J.Kuo 2006; herbarium specimens excluding duplicate occurrences (n = 7).

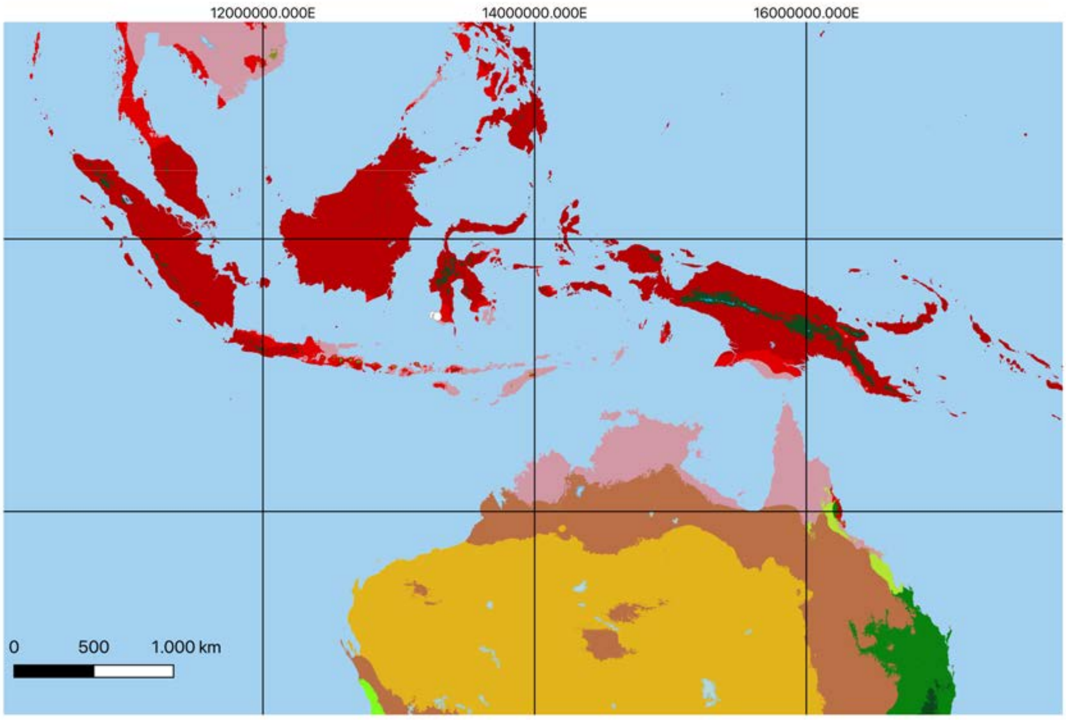

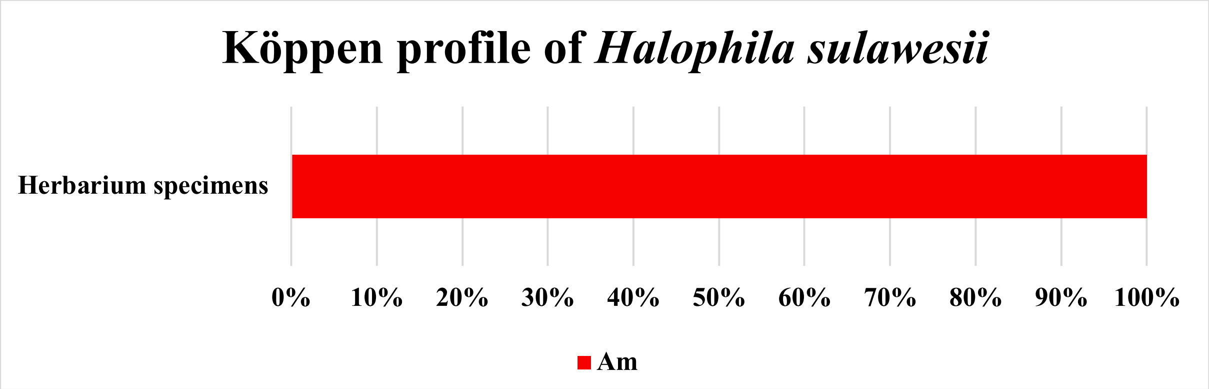

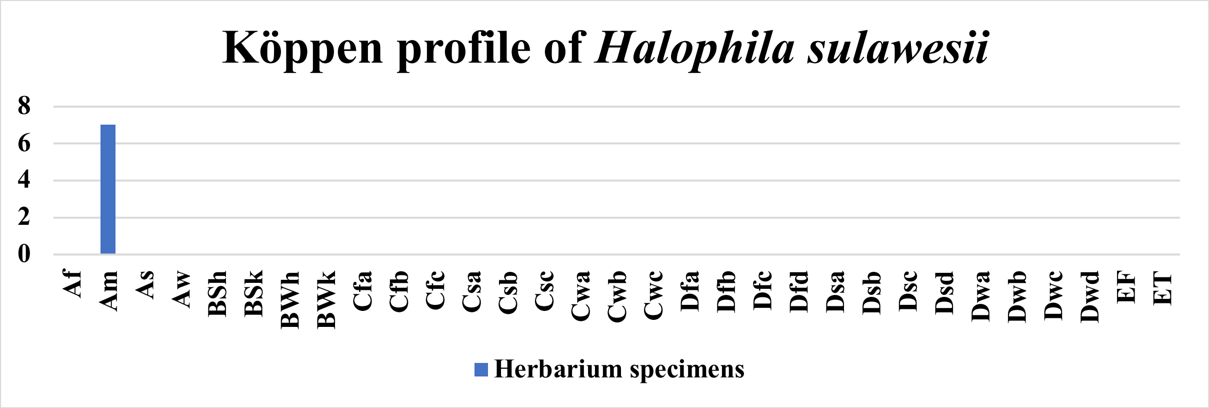

5.3.2.1.15. Köppen profile, distribution and climate map – GBIF occurrences of *Halophila tricostata* M.Greenway 1979; herbarium specimens excluding duplicate occurrences (n = 7).

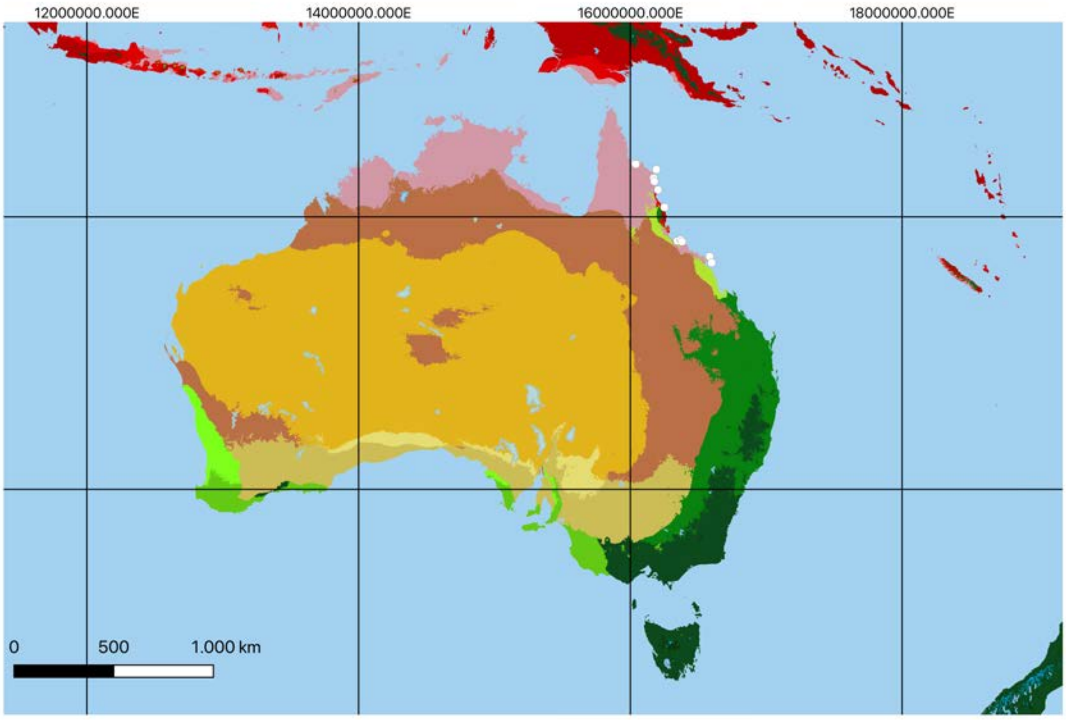

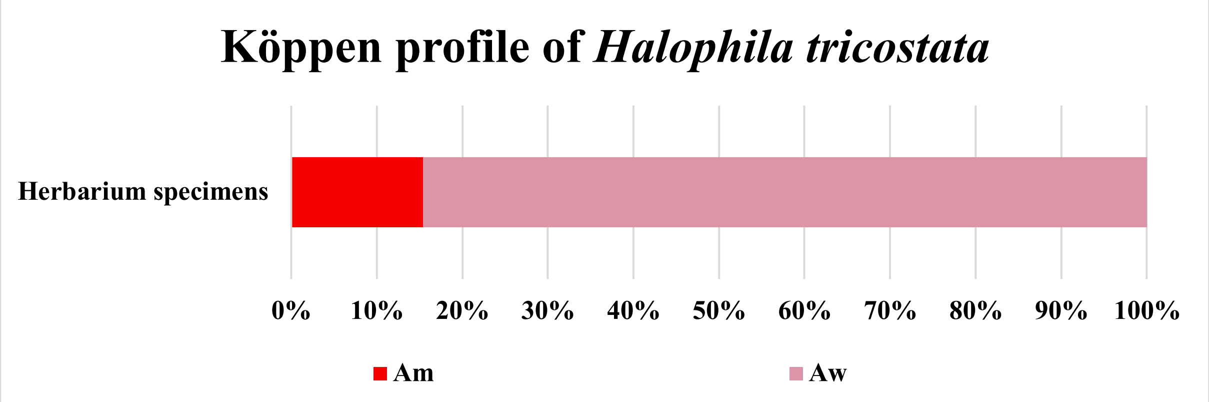

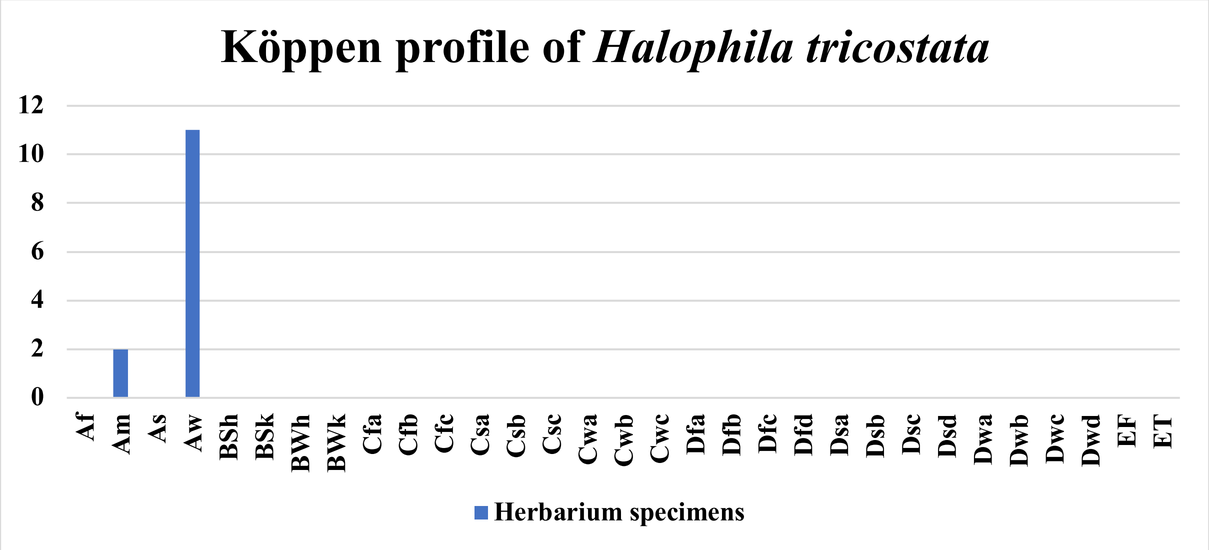

5.3.2.2. Genus *Thalassia* Banks & Sol. ex K.D.Koenig 1805.
Distribution and Köppen profiles of genus *Thalassia* incl. *T. hemprichii* and *T. testudinum,* based on preserved specimen (n = 341).

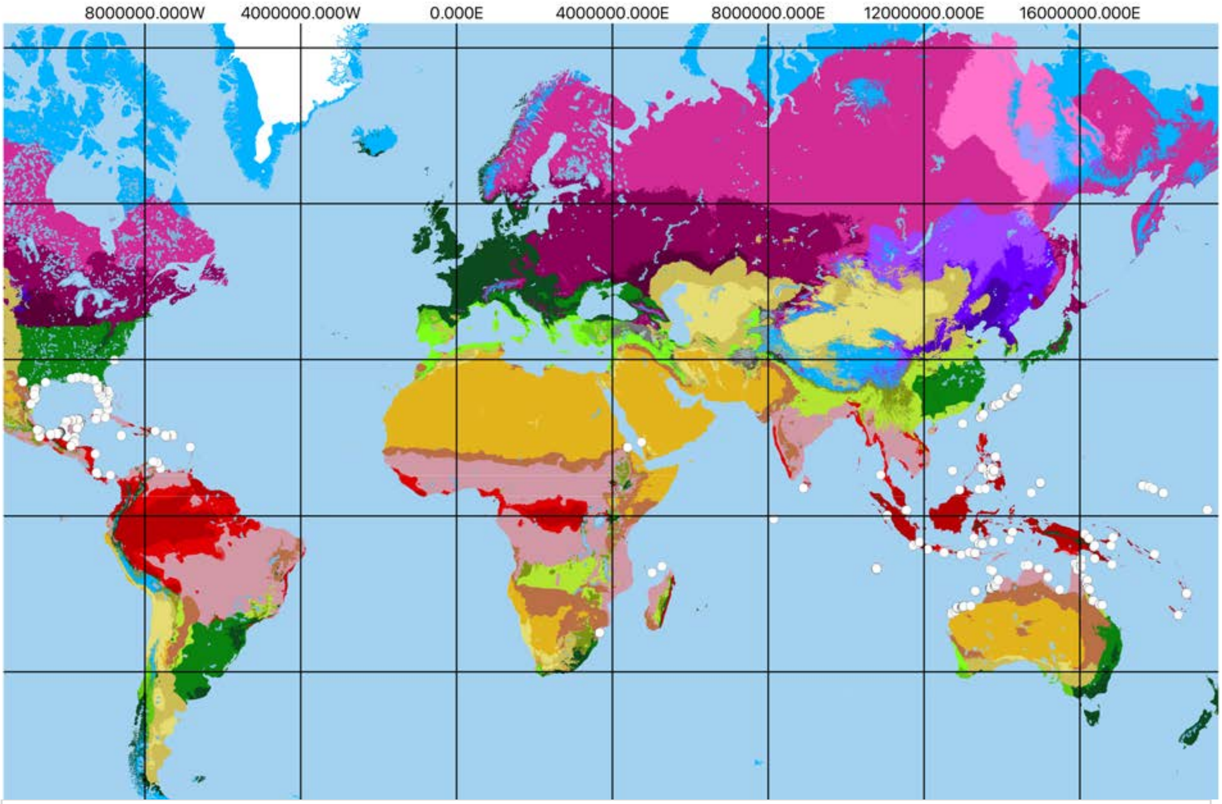

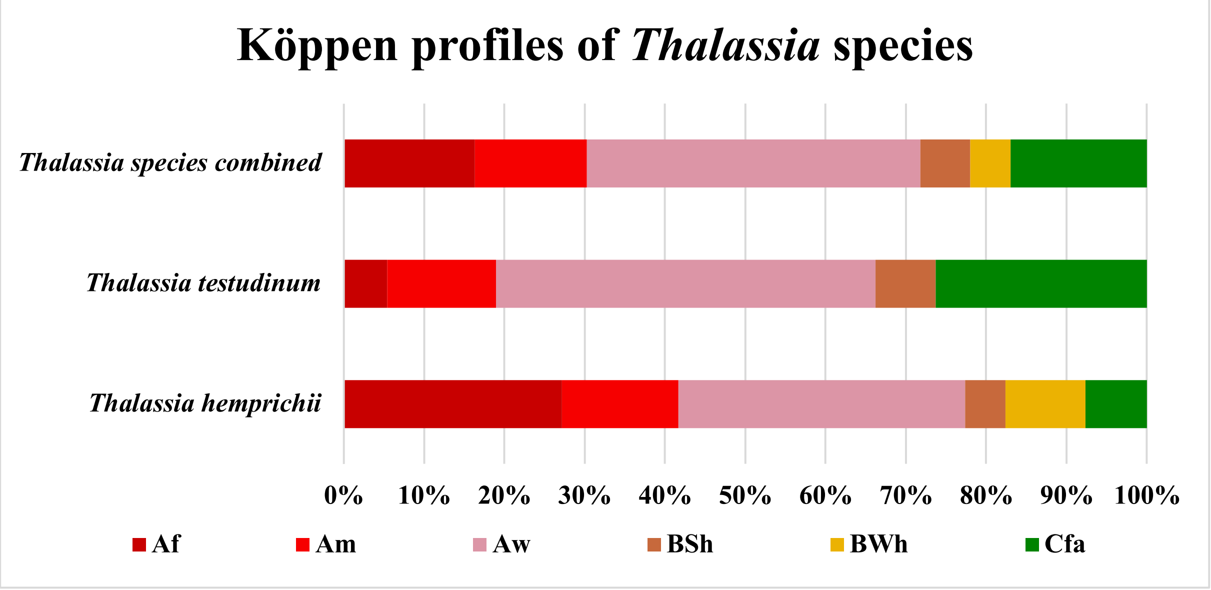

5.3.2.2.1. Köppen profile, distribution and climate map – GBIF occurrences of *Thalassia hemprichii* (Ehrenb. 1832) Asch. 1871; herbarium specimens excluding duplicate occurrences (n = 221).

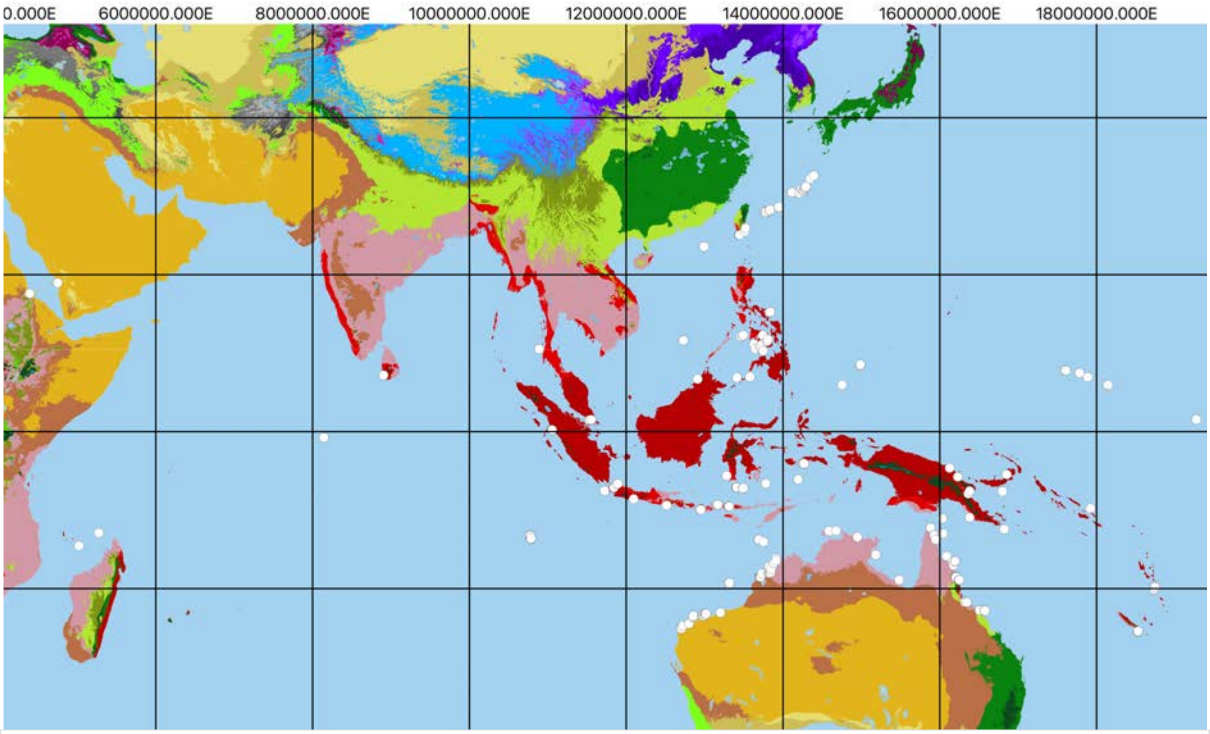

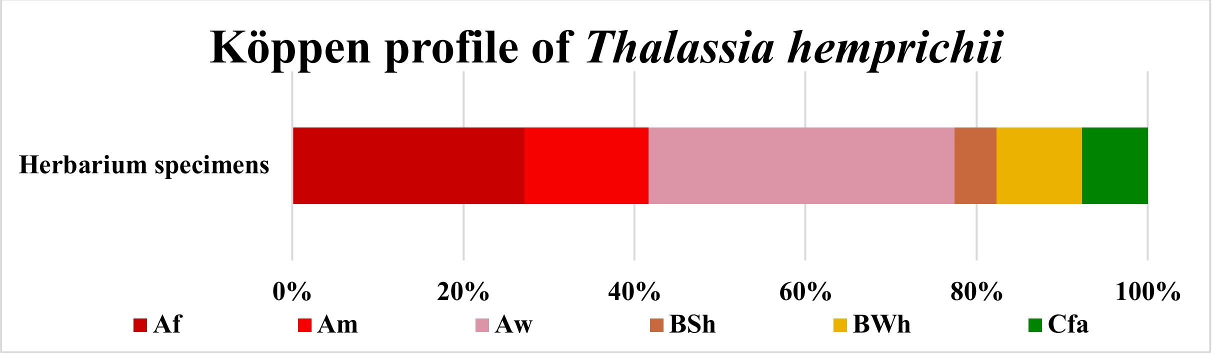

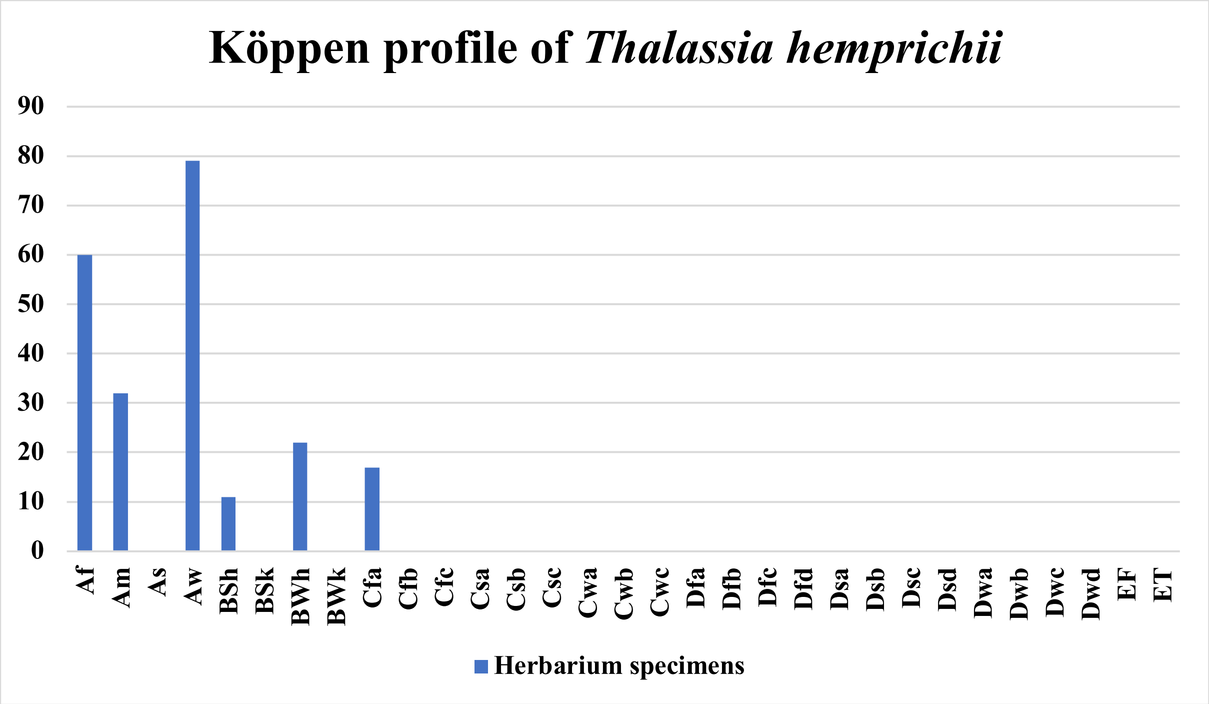

Köppen profile of *Thalassia hemprichii*
90
80
70
60
50
40
30
20
10
0
Af
Am
As
Aw
BSh
BSk
BWh
BWk
Cfa
Cfb
Cfc
Csa
Csb
Csc
Cwa
Cwb
Cwc
Dfa
Dfb
Dfc
Dfd
Dsa
Dsb
Dsc
Dsd
Dwa
Dwb
Dwc
Dwd
EF
ET
Herbarium specimens

5.3.2.2.2. Köppen profile, distribution and climate map – GBIF occurrences of *Thalassia testudinum* K.D.Koenig 1805; herbarium specimens excluding duplicate occurrences (n = 148).

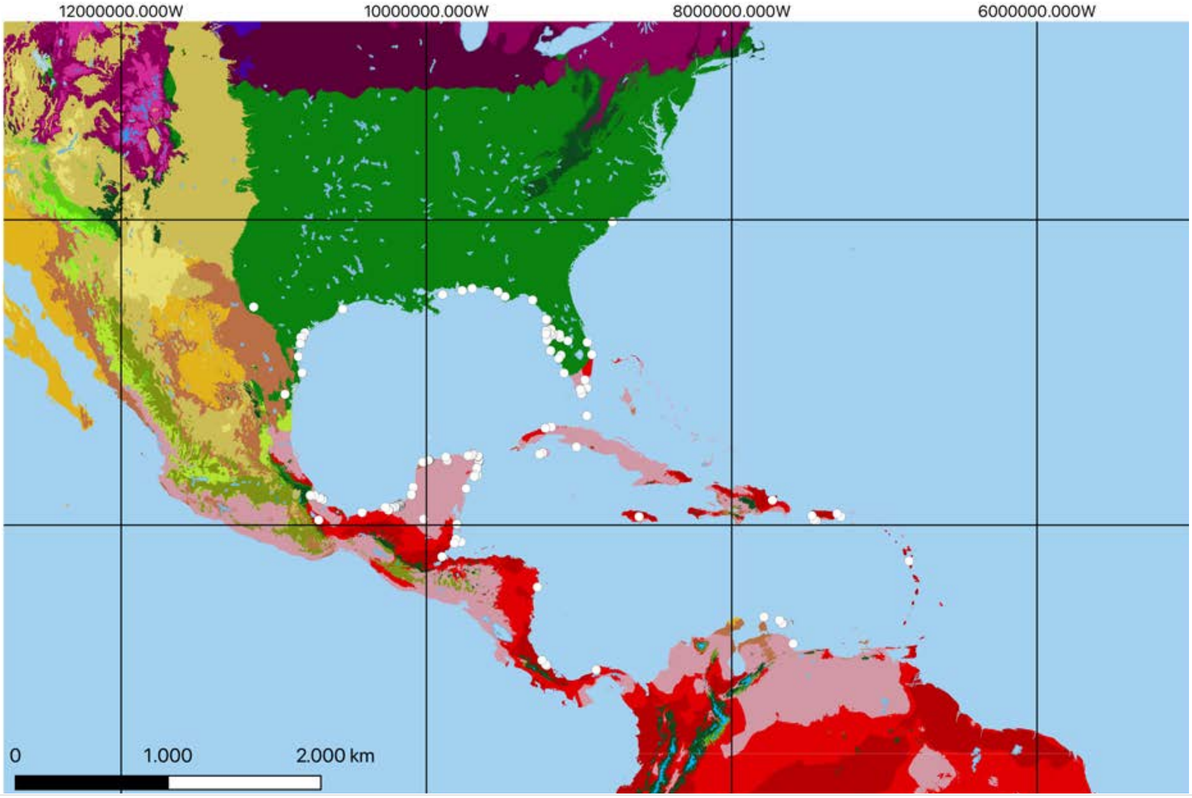

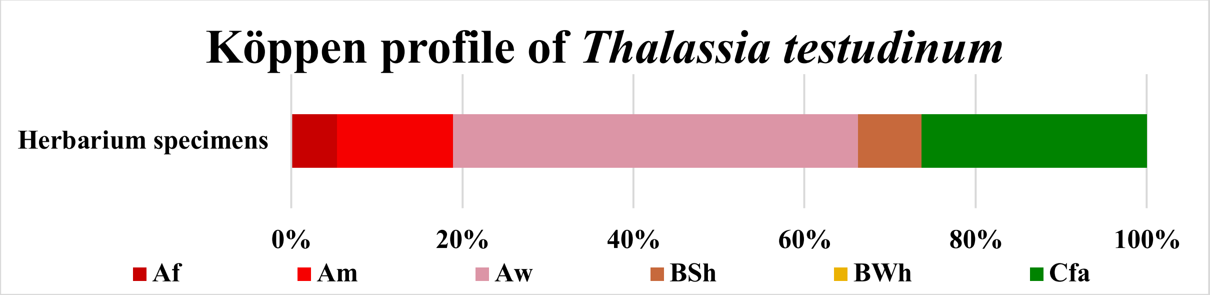

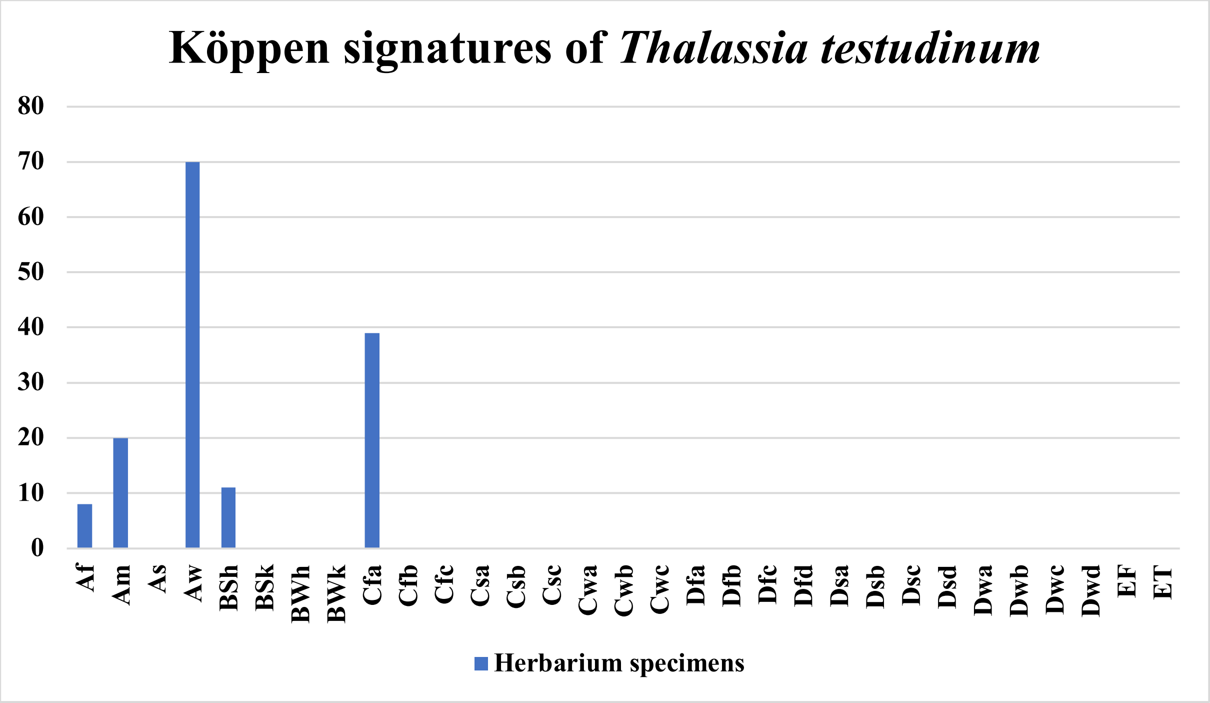
Köppen signatures of *Thalassia testudinum*
80
70
60
50
40
30
20
10
0
Af
Am
As
Aw
BSh
BSk
BWh
BWk
Cfa
Cfb
Cfc
Csa
Csb
Csc
Cwa
Cwb
Cwc
Dfa
Dfb
Dfc
Dfd
Dsa
Dsb
Dsc
Dsd
Dwa
Dwb
Dwc
Dwd
EF
ET
Herbarium specimens

5.3.3. Third diverging genus within Clade A of Hydrocharitaceae cf. Chen et. al (2012), comprising ***Najas* L., 1753**

5.3.3.1. Genus *Najas* L. 1753.

Distribution and Köppen profiles of genus *Najas* L., incl. *N. affinis*, *N. ancistrocarpa*, *N. arguta*, *N. australis*, *N. baldwinii*, *N. browniana*, *N. chinensis*, *N. conferta*, *N. filifolia, N. flexilis*, *N. gracillima*, *N. graminea*, *N. guadalupensis*, *N. hagerupii*, *N. halophila*, *N. horrida*, *N. indica*, *N. kurziana*, *N. madagascariensis*, *N. malesiana*, *N. marina*, *N. oguraensis*, *N. pectinate*, *N. schweinfurthii*, *N. tenuicaulis*, *N. tenuifolia*, *N. tenuissima*, *N. testui*, *N. welwitschia* , *N. wrightiana* (n = 8269).

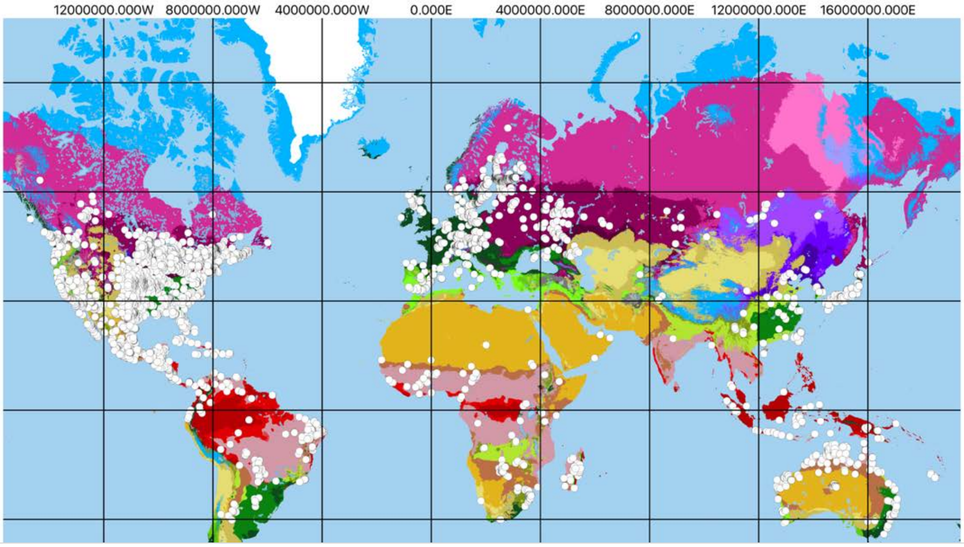

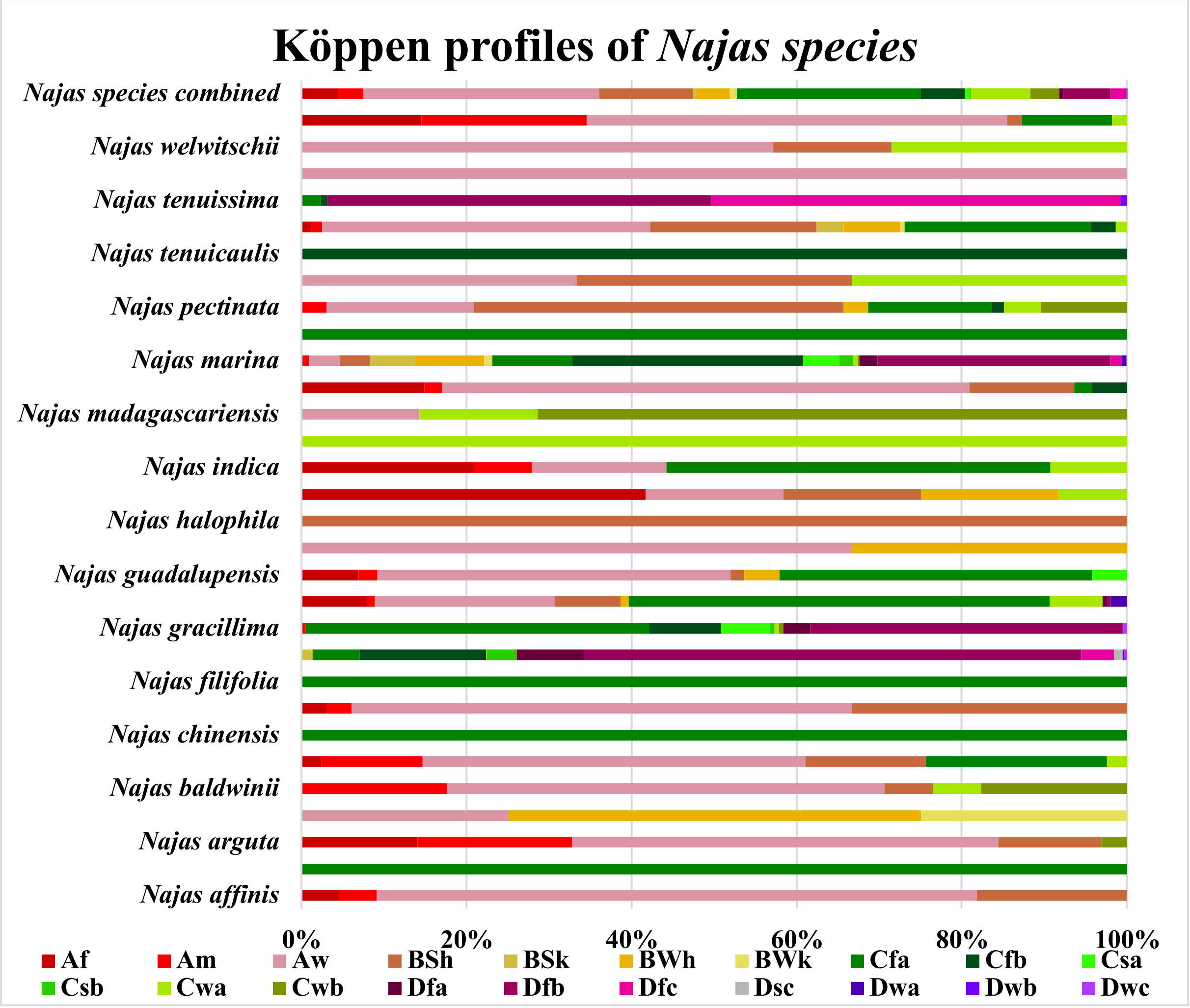

5.3.3.1.1. Köppen profile, distribution and climate map – GBIF occurrences of *Najas affinis* Rendle 1900; herbarium specimens excluding duplicate occurrences (n = 22).

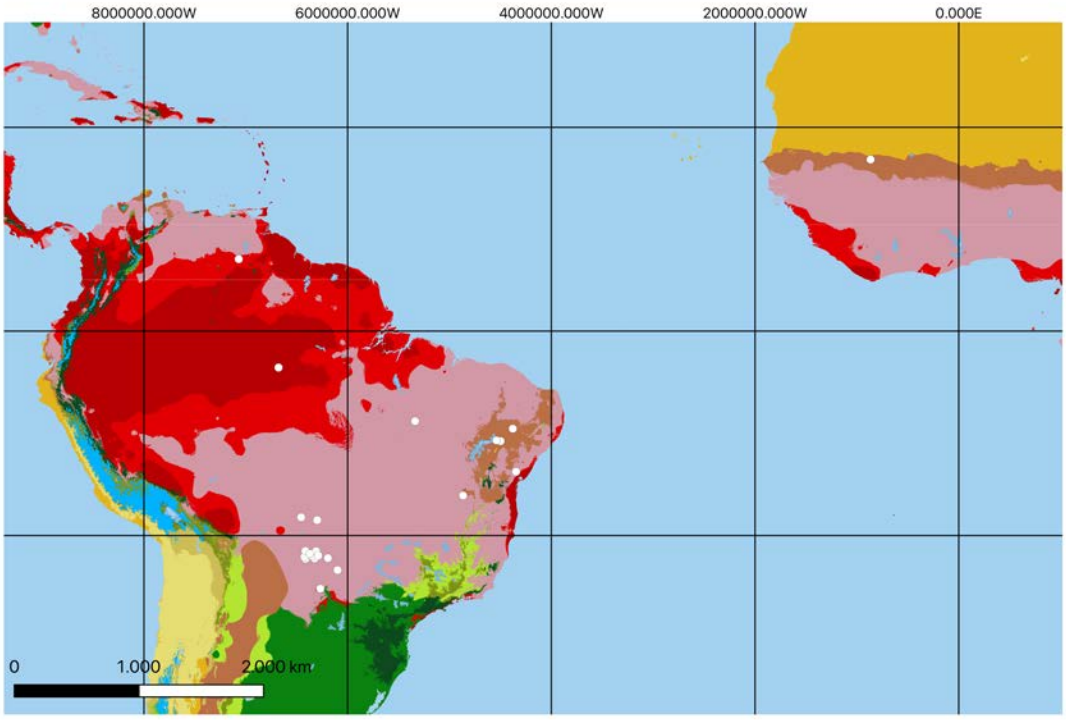

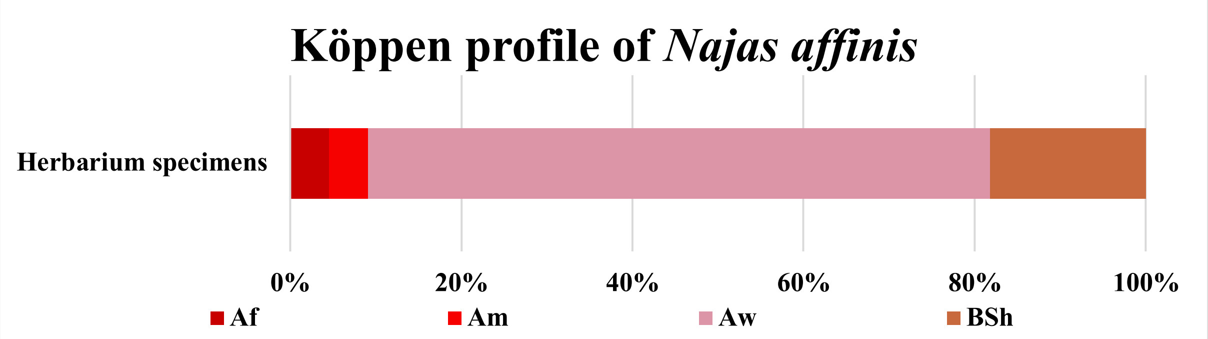

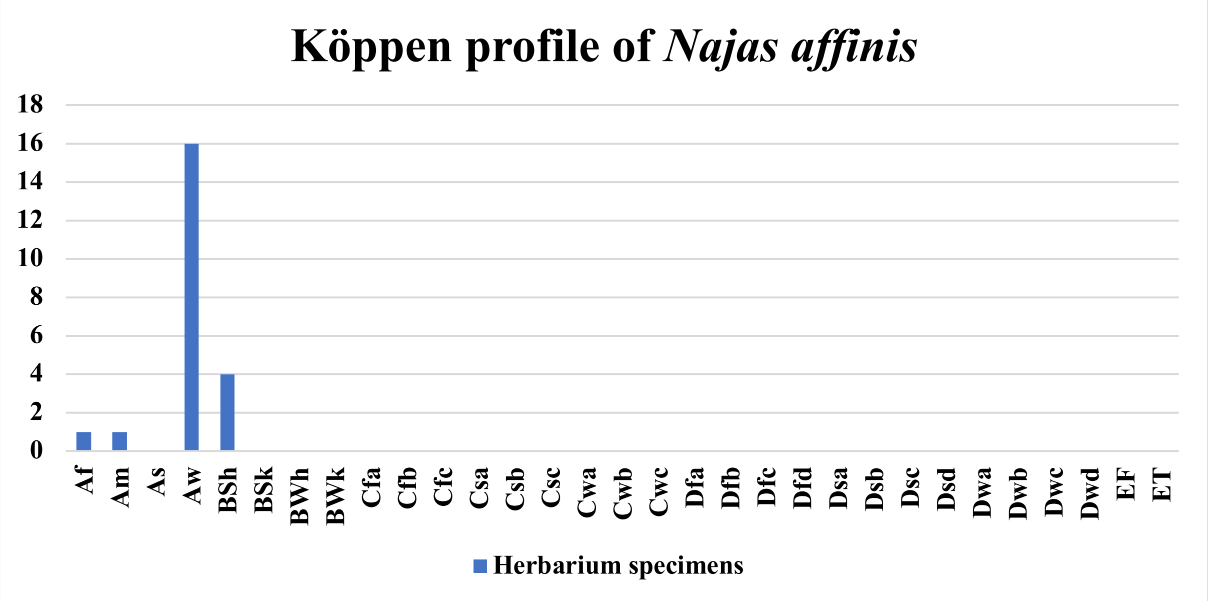

5.3.3.1.2. Köppen profile, distribution and climate map – GBIF occurrences of *Najas ancistrocarpa* A.Braun ex Magnus 1870; herbarium specimens excluding duplicate occurrences (n = 10).

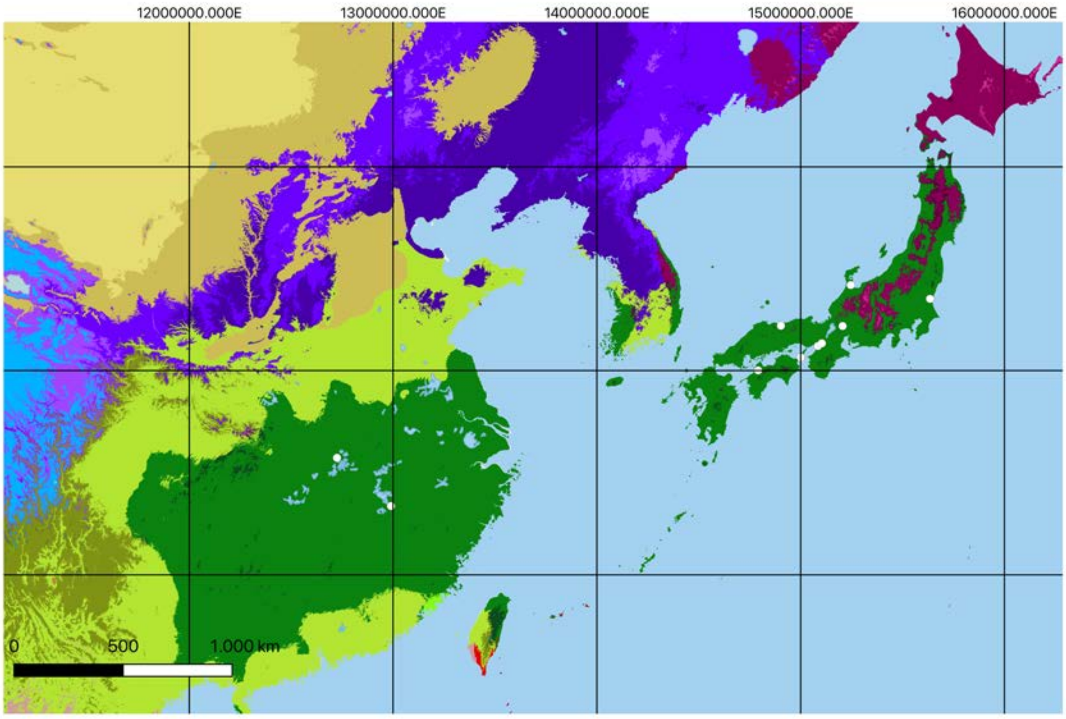

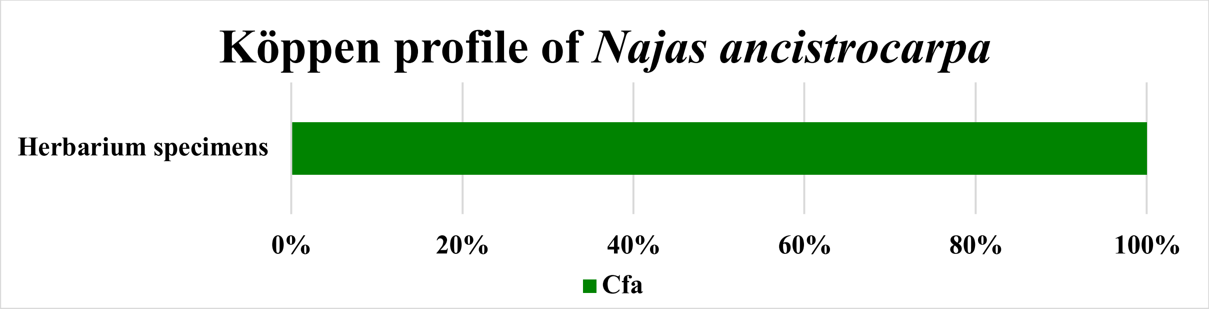

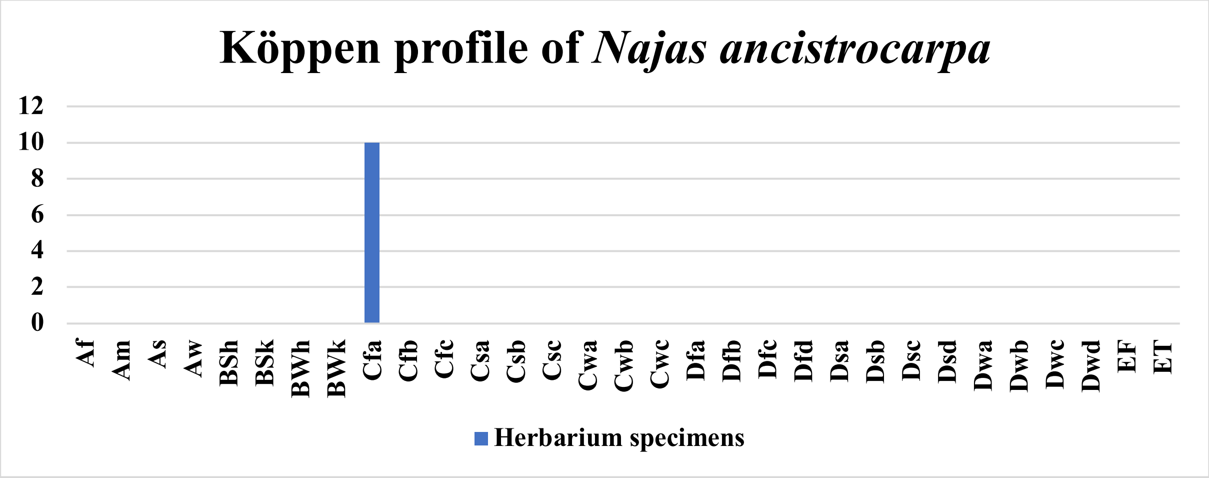

5.3.3.1.3. Köppen profile, distribution and climate map – GBIF occurrences of *Najas arguta* Kunth 1816; herbarium specimens excluding duplicate occurrences (n = 64).

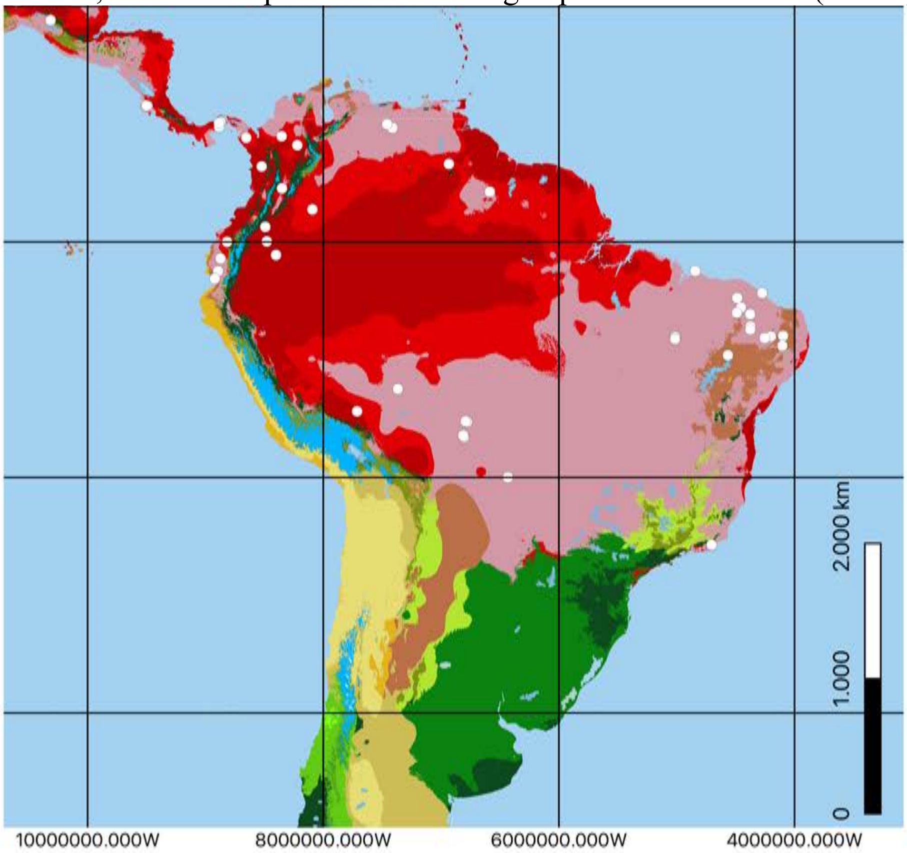

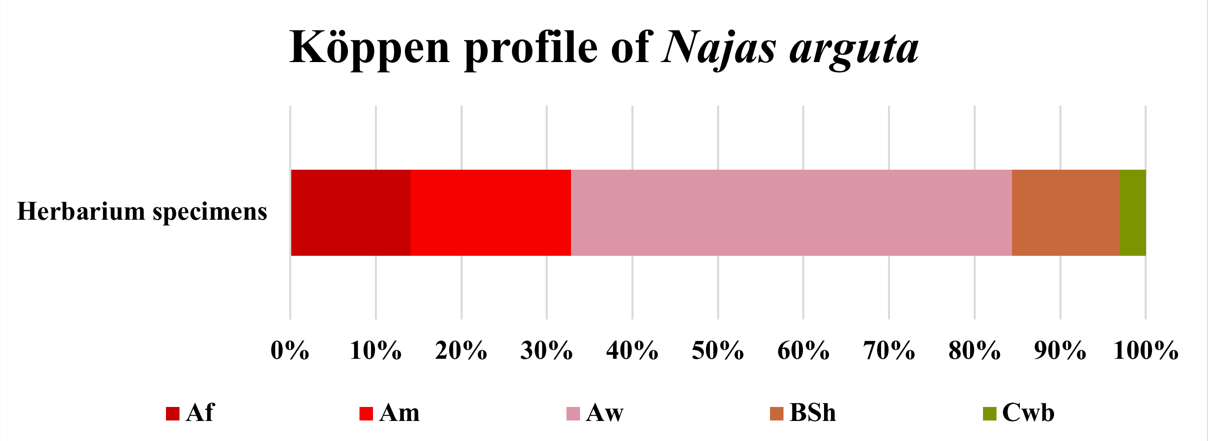

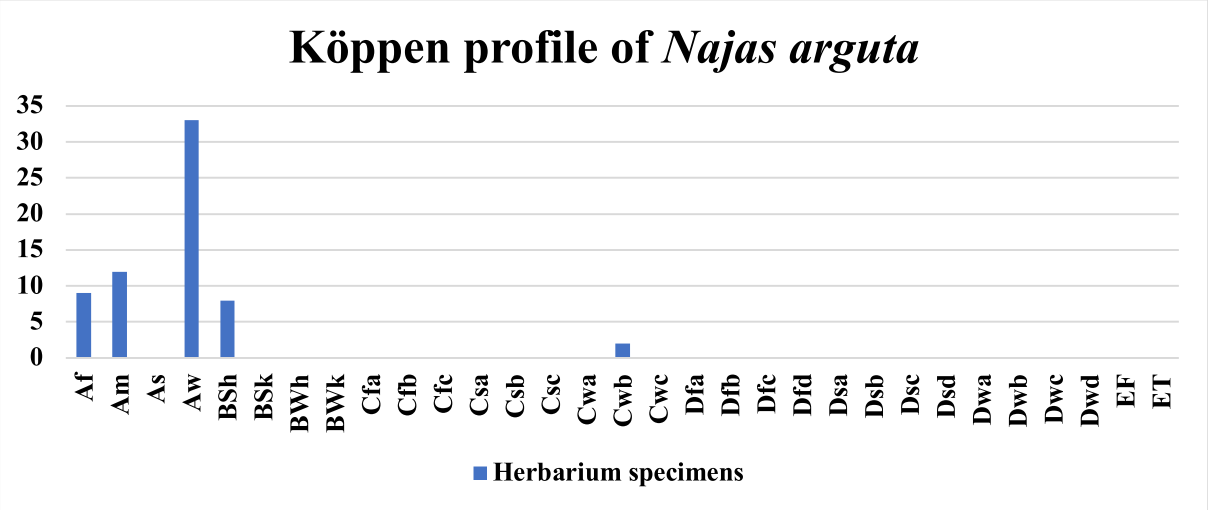

5.3.3.1.4. Köppen profile, distribution and climate map – GBIF occurrences of *Najas australis* Borey ex Cham. 1829; herbarium specimens excluding duplicate occurrences (n = 4).

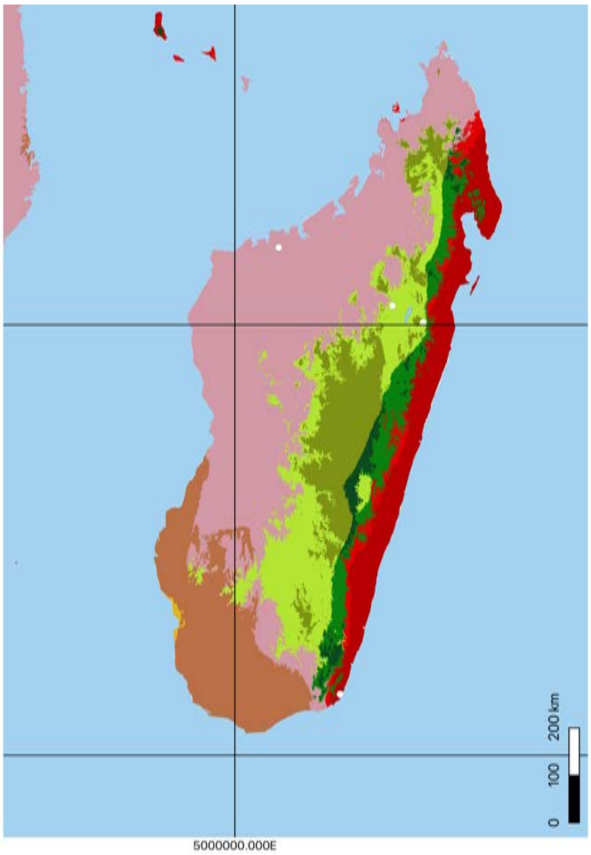

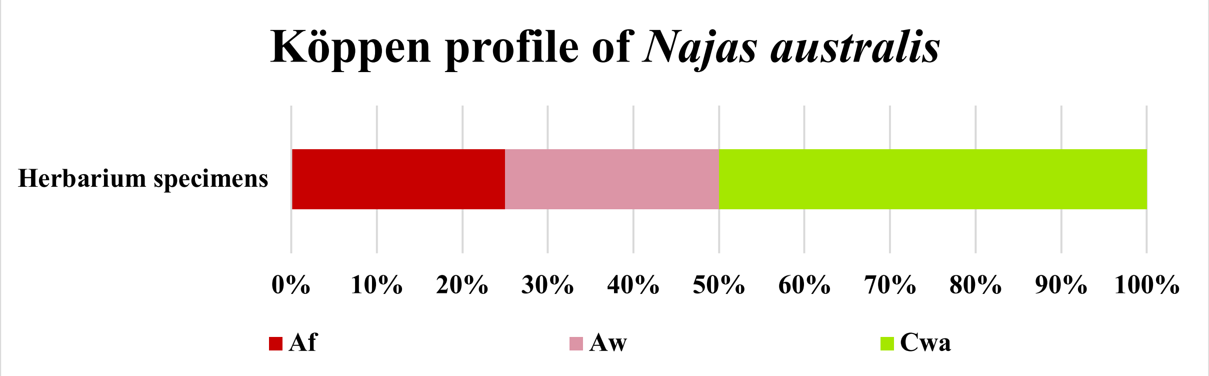

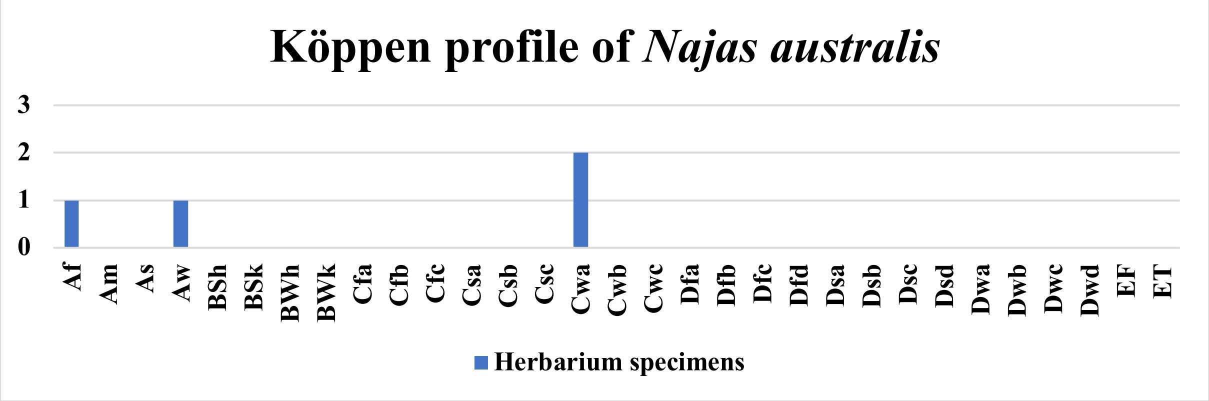

5.3.3.1.5. Köppen profile, distribution and climate map – GBIF occurrences of *Najas baldwinii* Horn 1950; herbarium specimens excluding duplicate occurrences (n = 17).

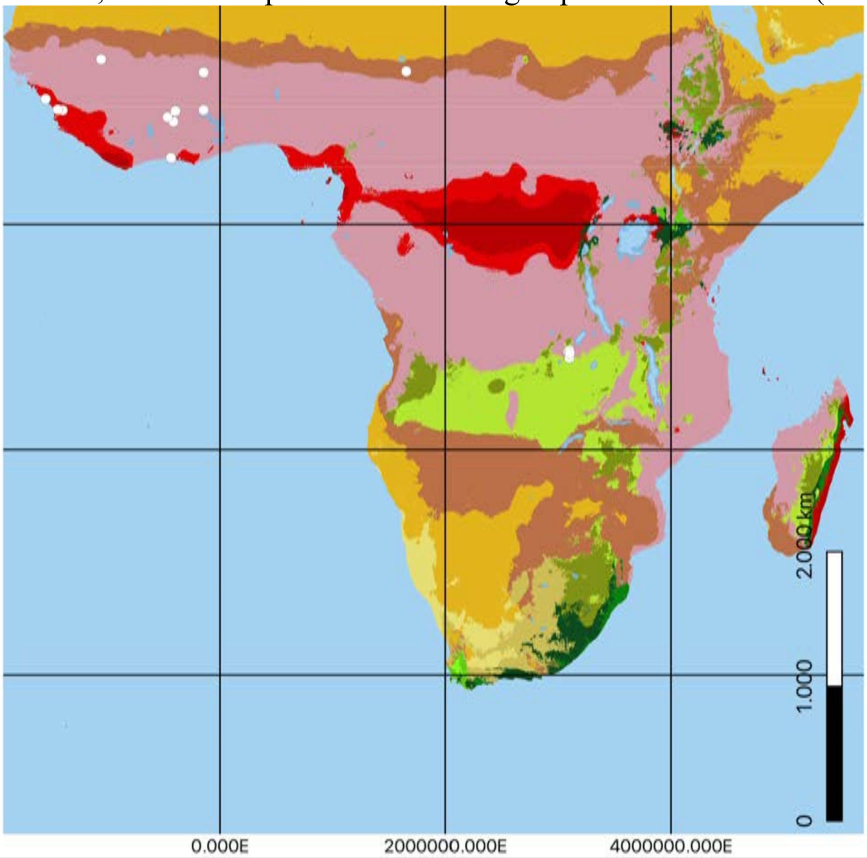

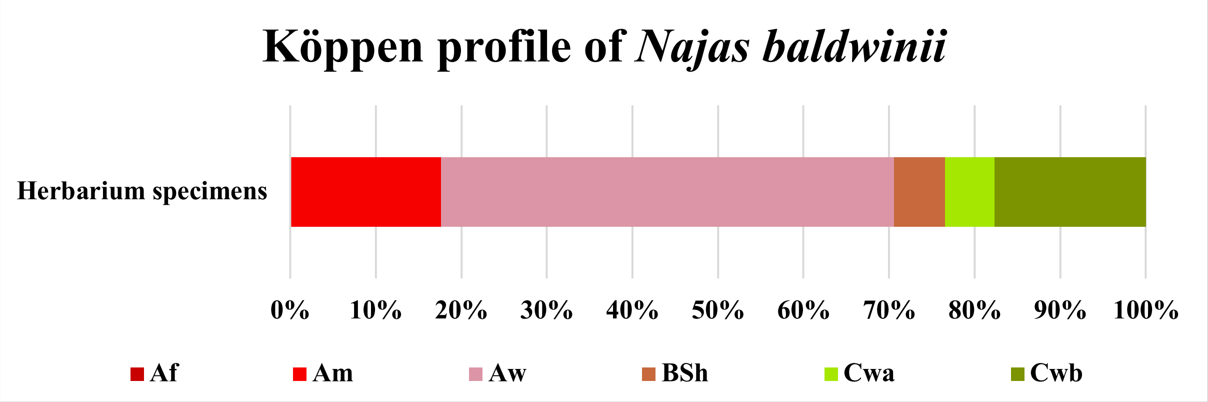

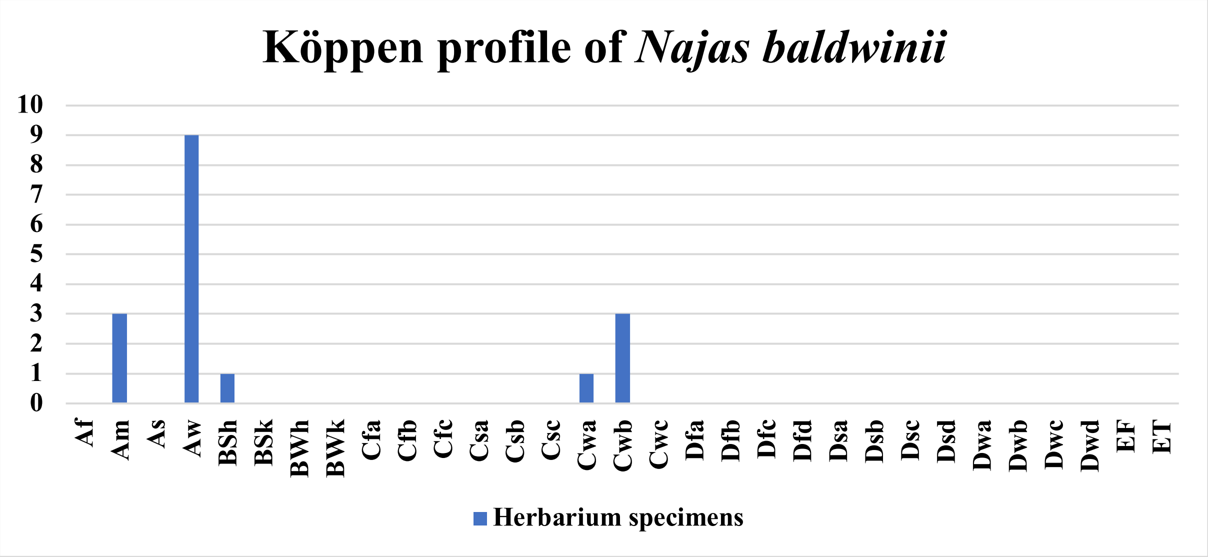

5.3.3.1.6. Köppen profile, distribution and climate map – GBIF occurrences of *Najas browniana* Rendle 1899; herbarium specimens excluding duplicate occurrences (n = 41).

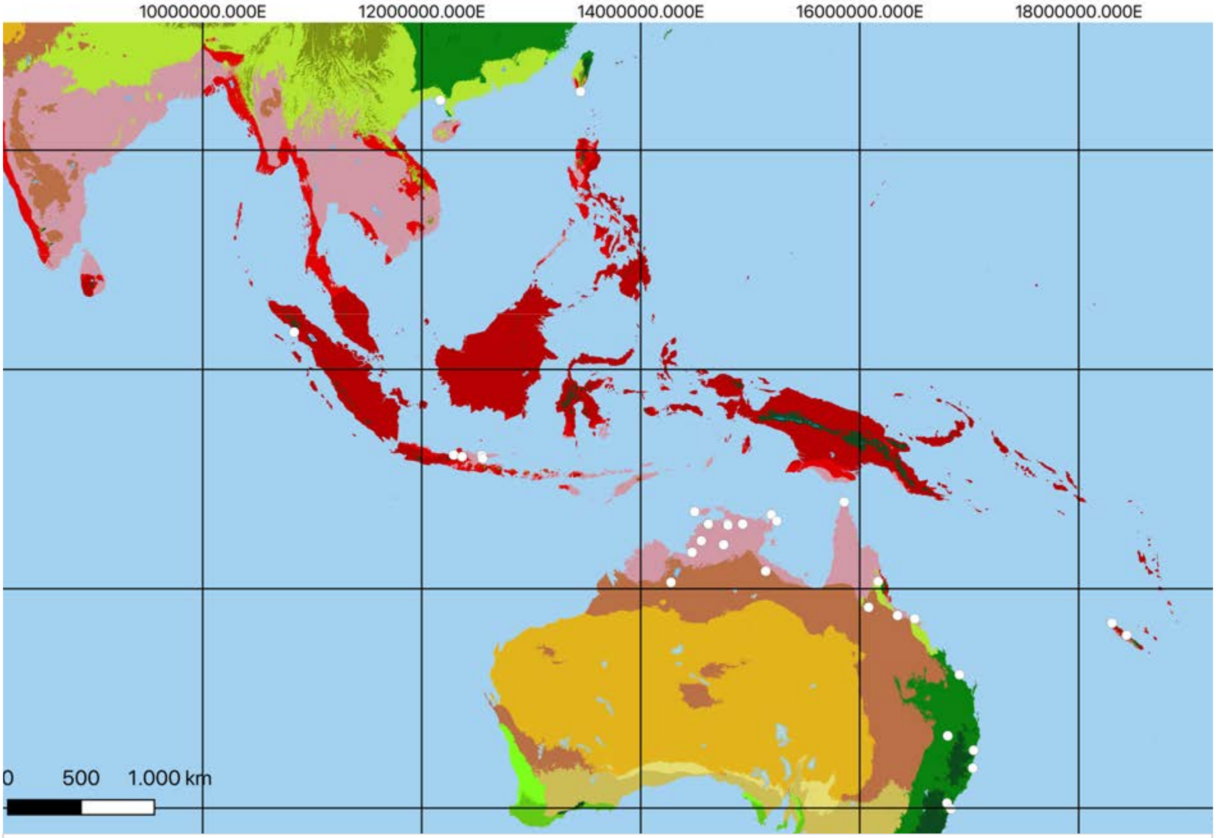

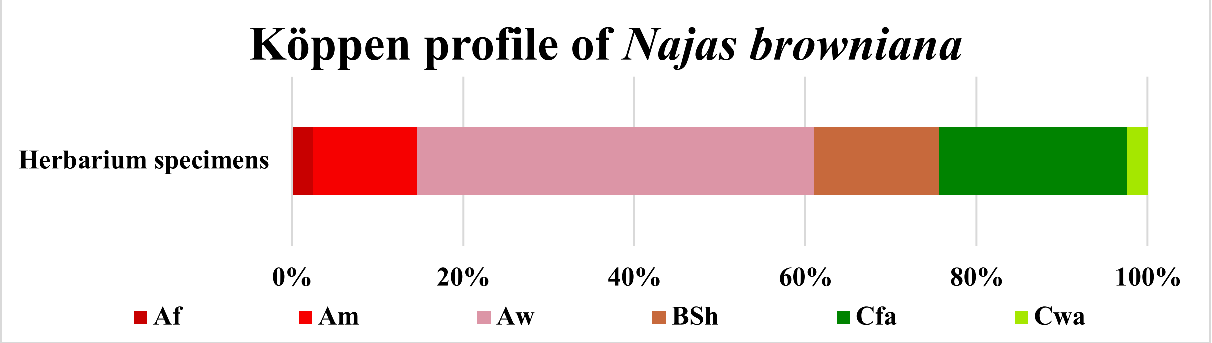

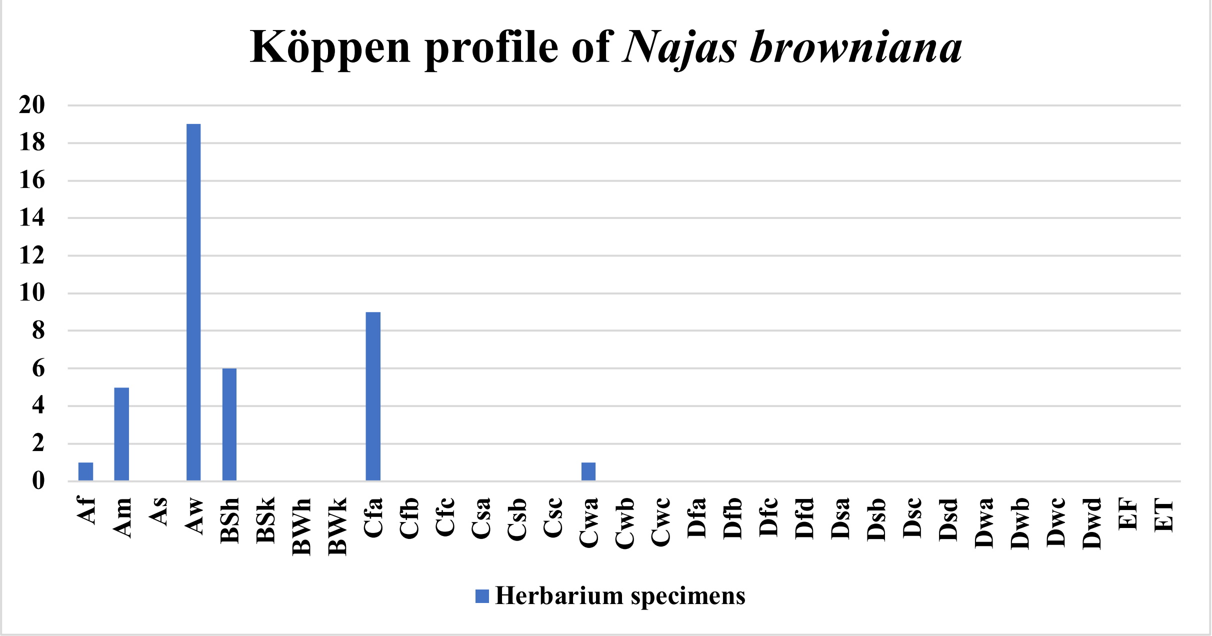

5.3.3.1.7. Köppen profile, distribution and climate map – GBIF occurrences of *Najas chinensis* N.Z.Wang 1985; herbarium specimens excluding duplicate occurrences (n = 6).

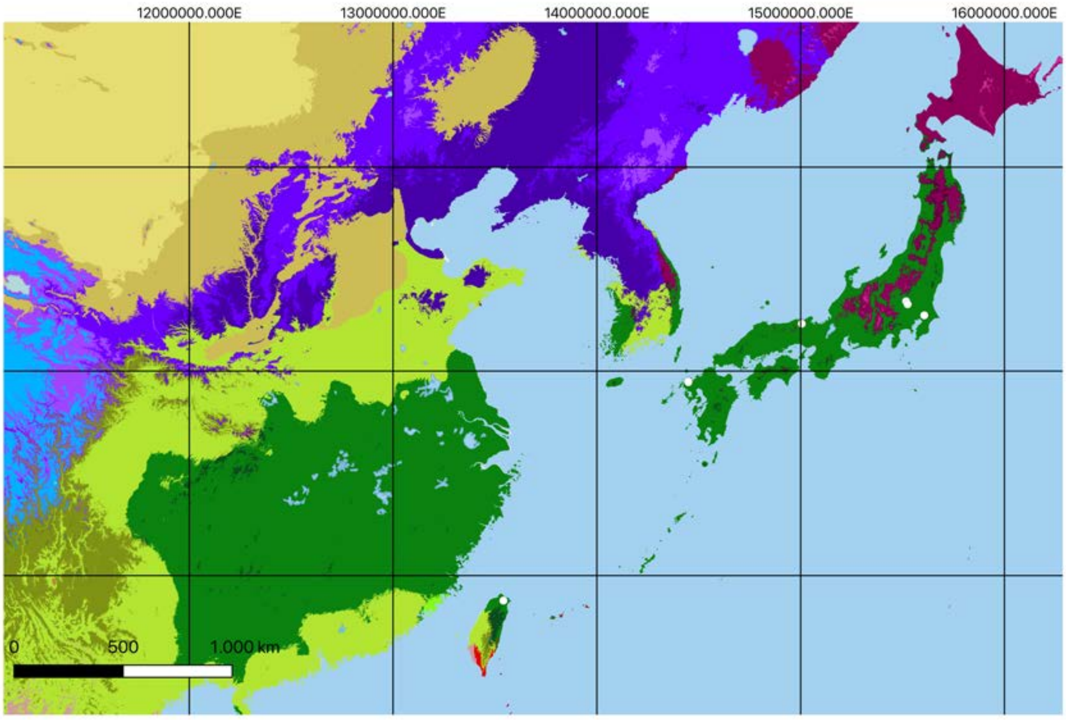

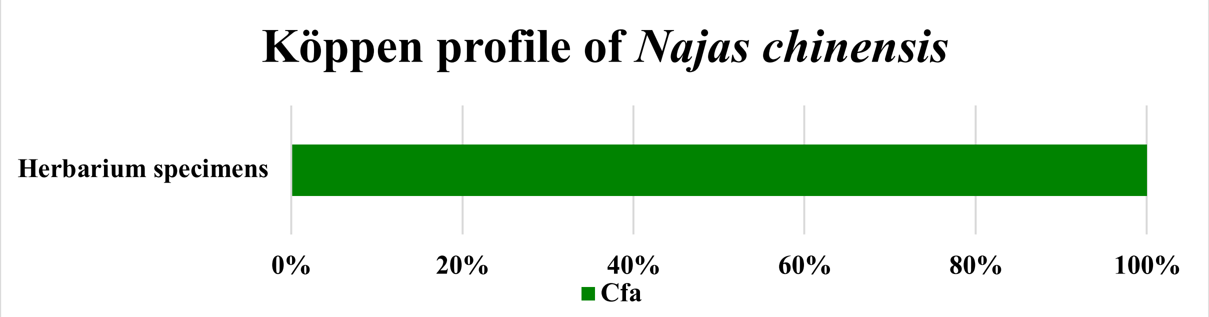

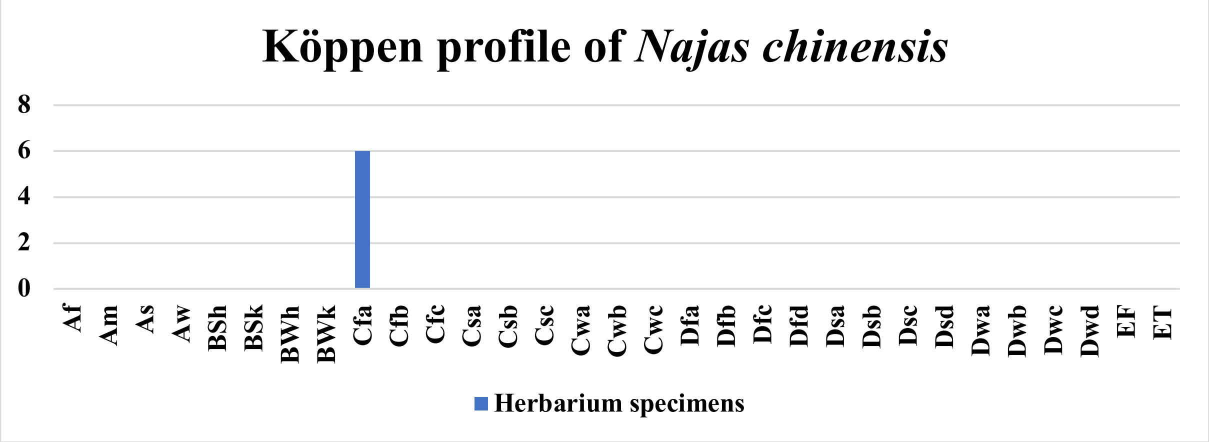

5.3.3.1.8. Köppen profile, distribution and climate map – GBIF occurrences of *Najas conferta* (A.Braun 1864) A.Braun 1868; herbarium specimens excluding duplicate occurrences (n = 33).

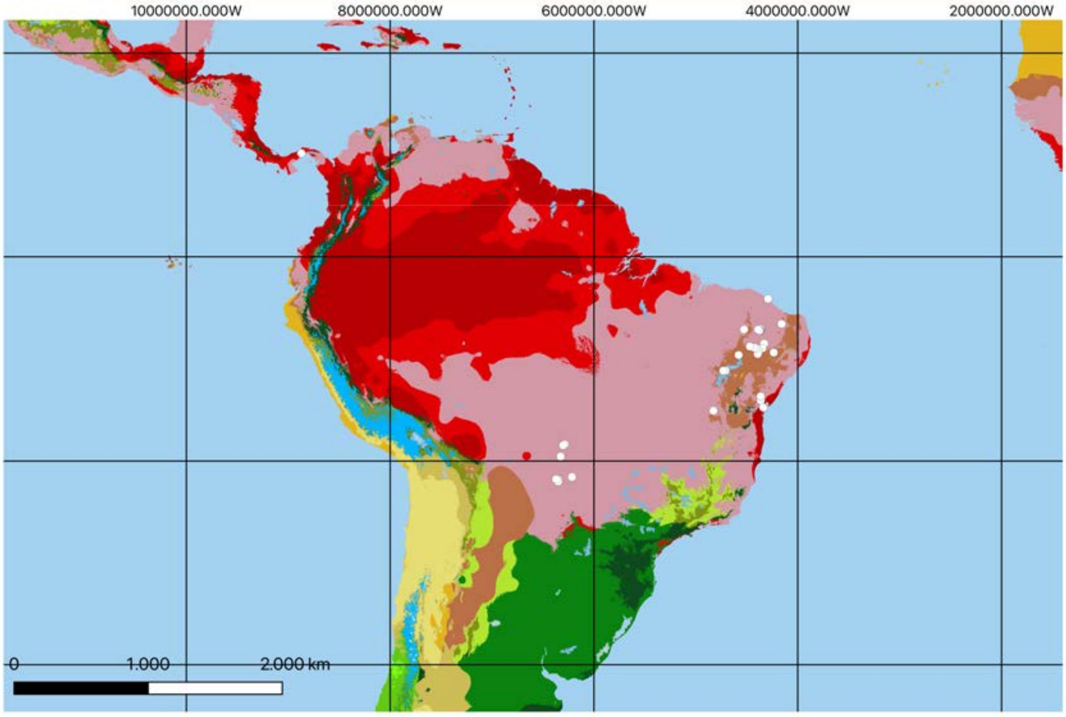

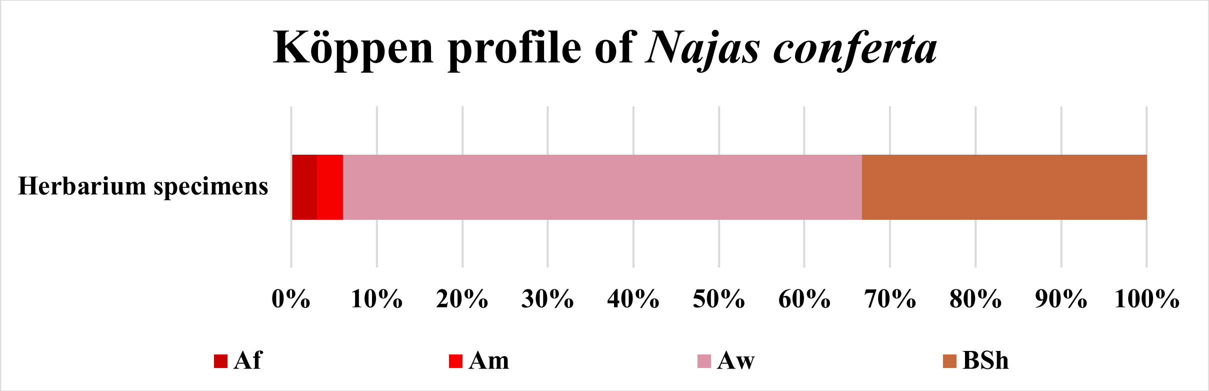

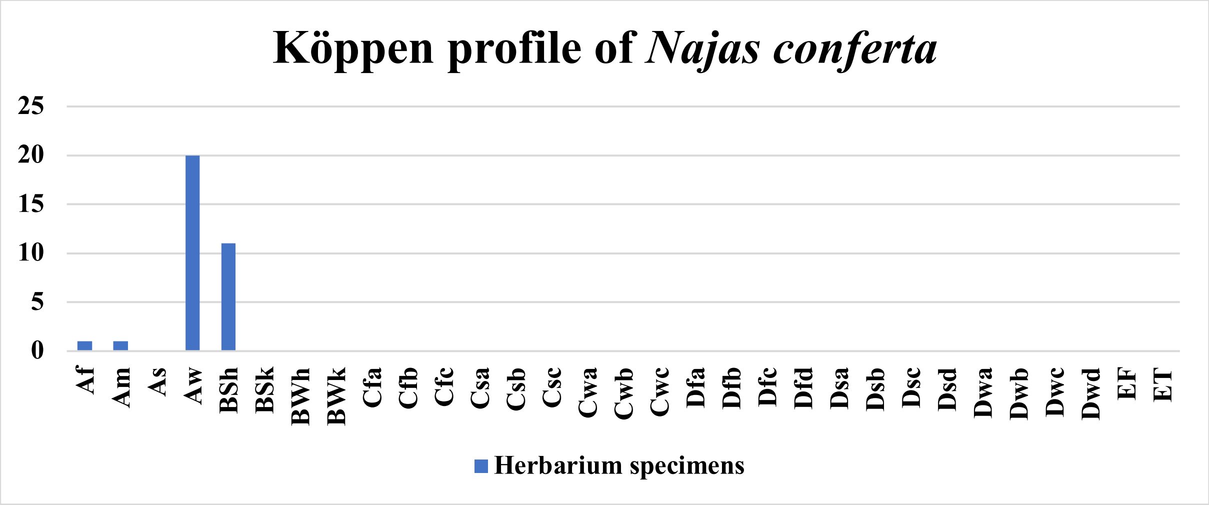

5.3.3.1.9. Köppen profile, distribution and climate map – GBIF occurrences of *Najas filifolia* R.R.Haynes 1985; herbarium specimens excluding duplicate occurrences (n = 6).

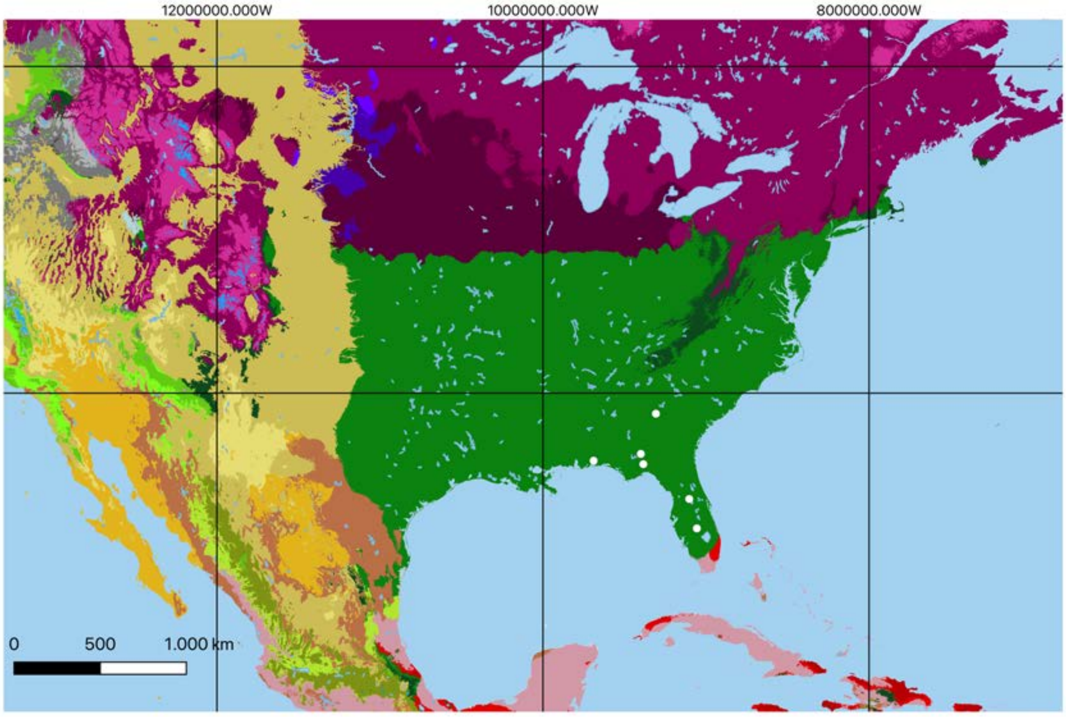

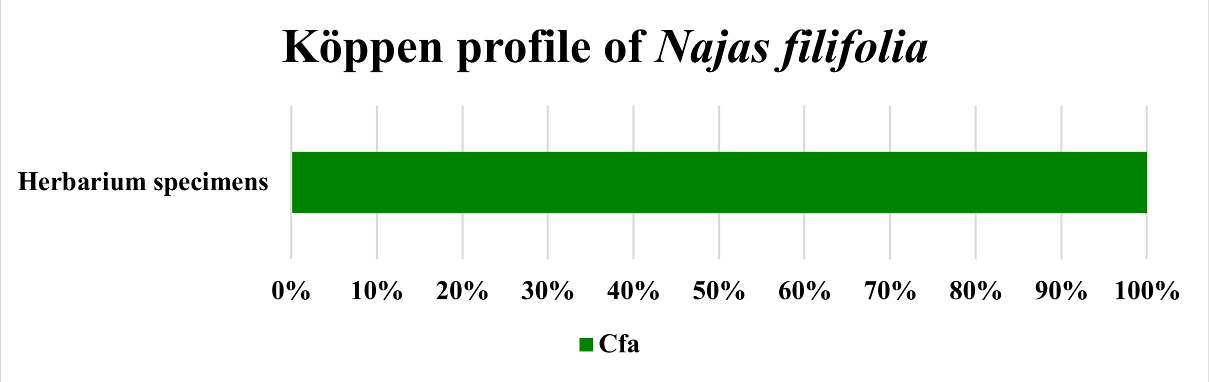

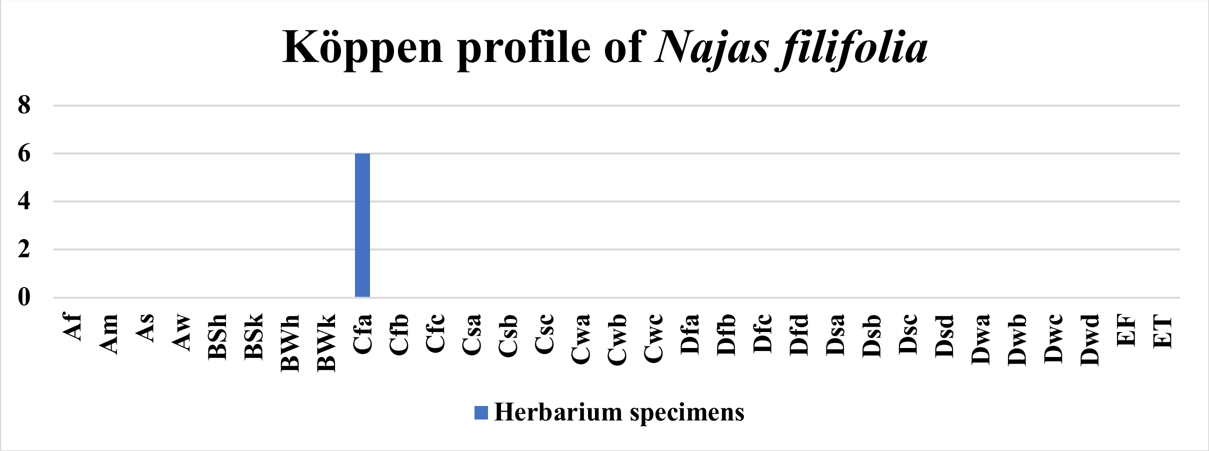

5.3.3.1.10. Köppen profile, distribution and climate map – GBIF occurrences of *Najas flexilis* (Willd. 1801) Rostk. & W.L.E.Schmidt 1924; herbarium specimens excluding duplicate occurrences (n = 1419).

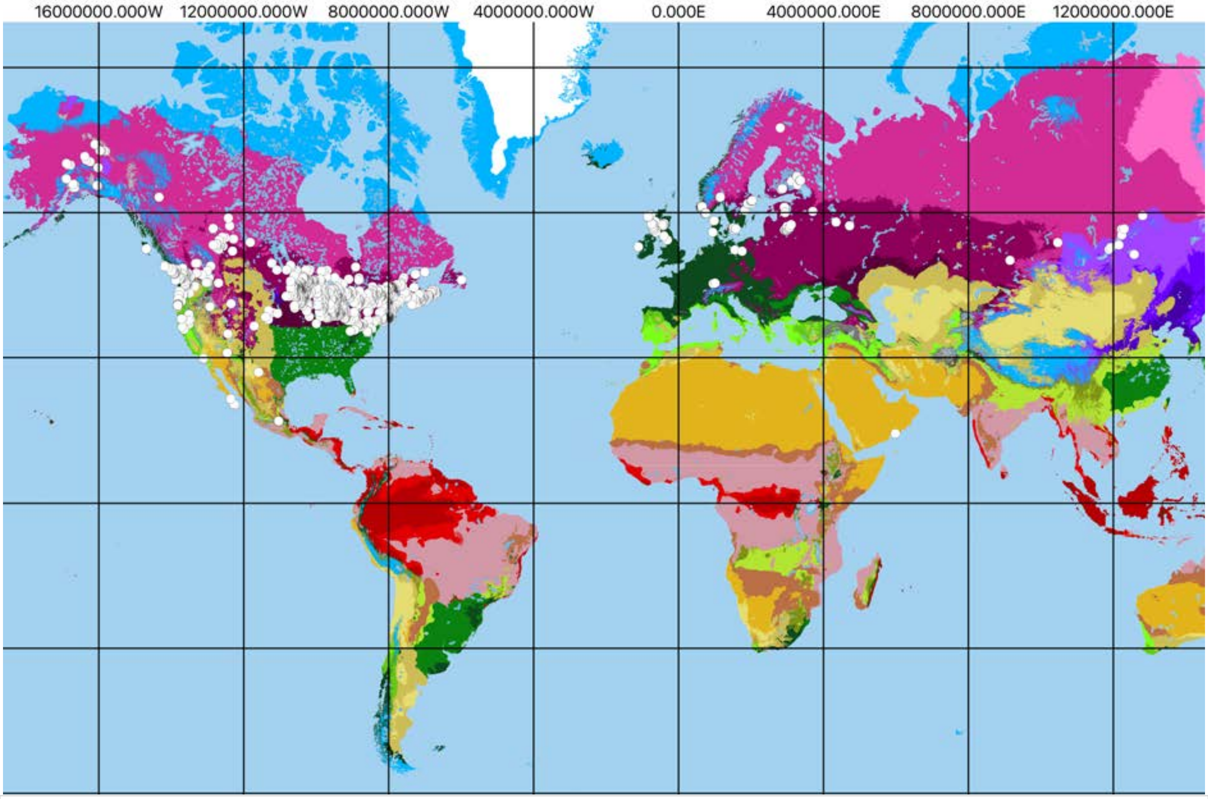

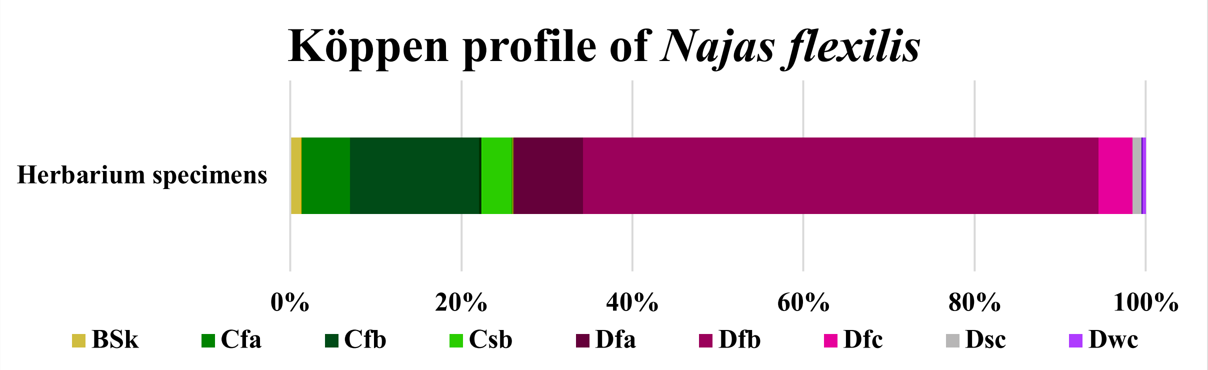

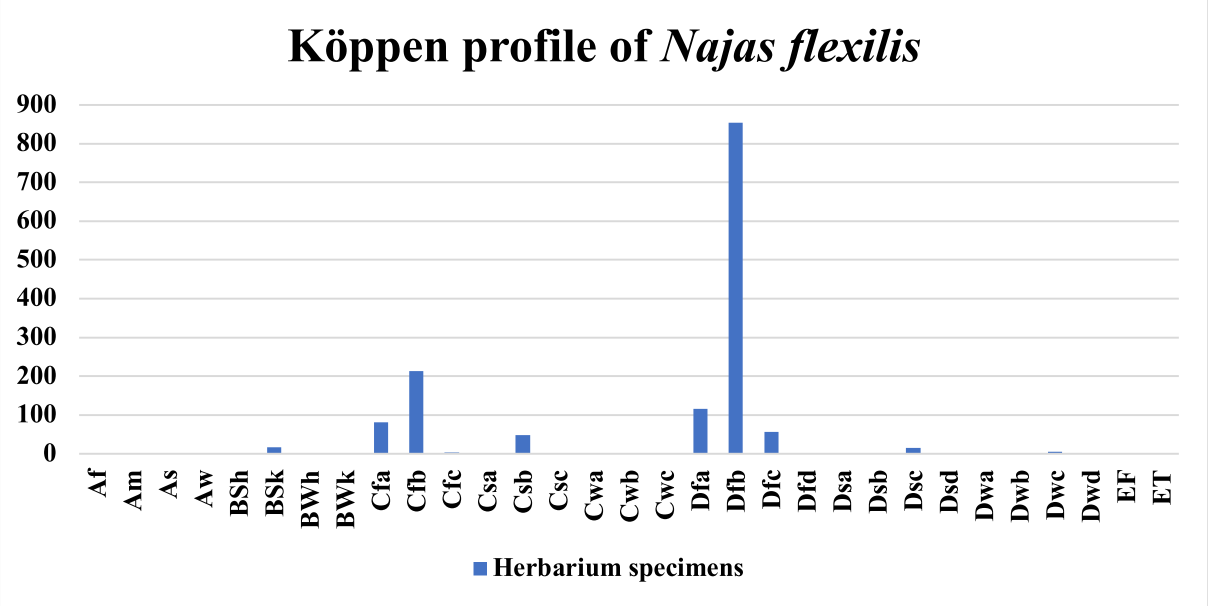

5.3.3.1.11. Köppen profile, distribution and climate map – GBIF occurrences of *Najas gracillima* (A.Braun ex Engelm. 1868) Magnus 1870; herbarium specimens excluding duplicate occurrences (n = 185).

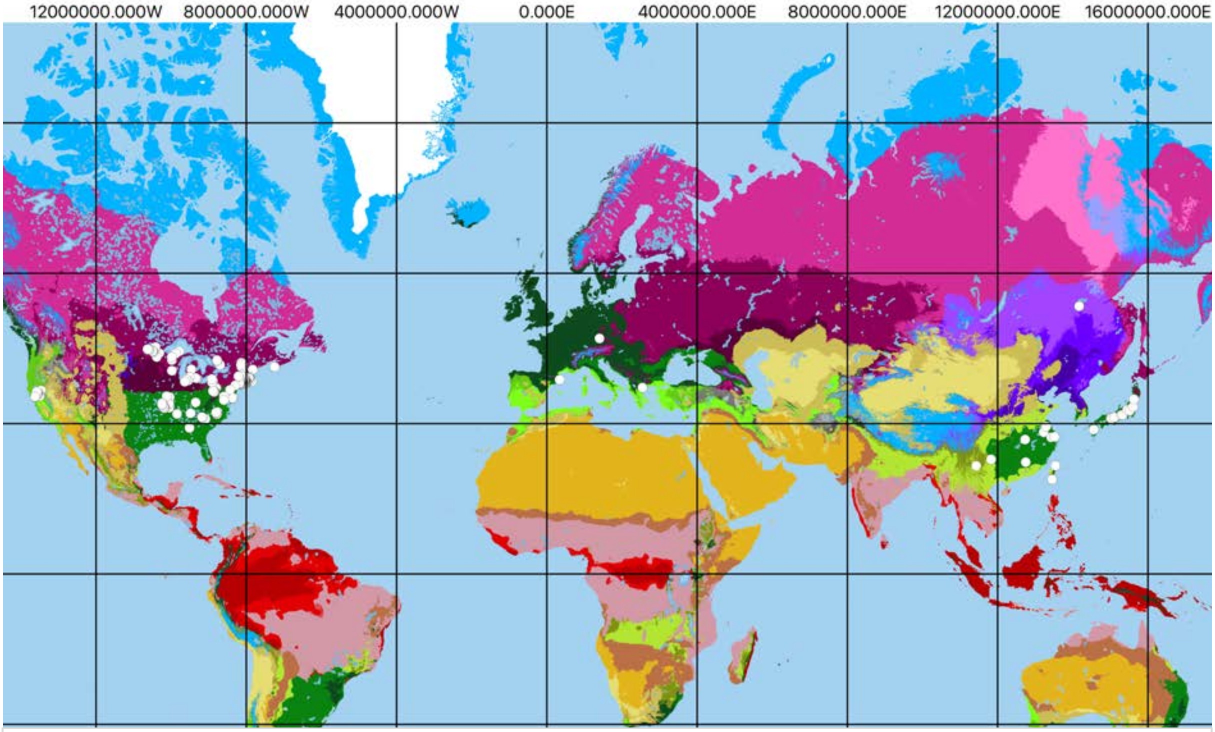

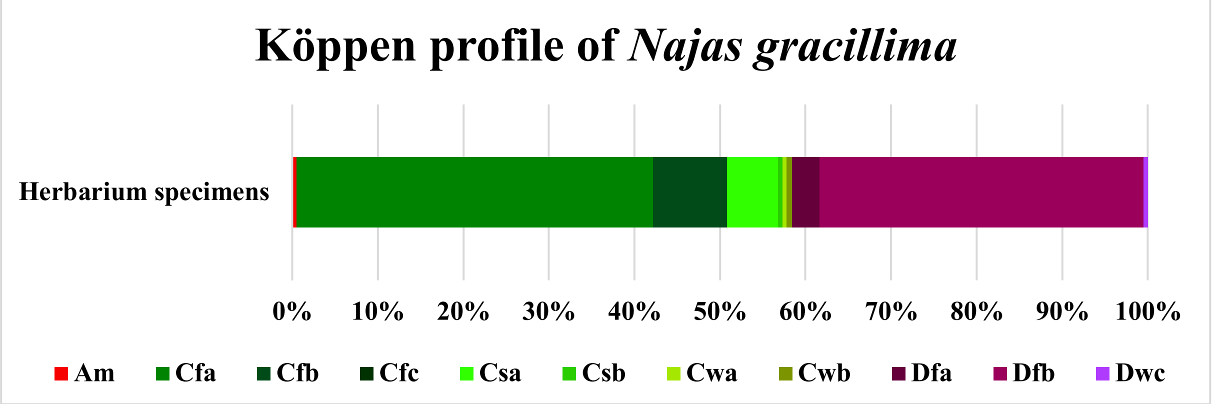

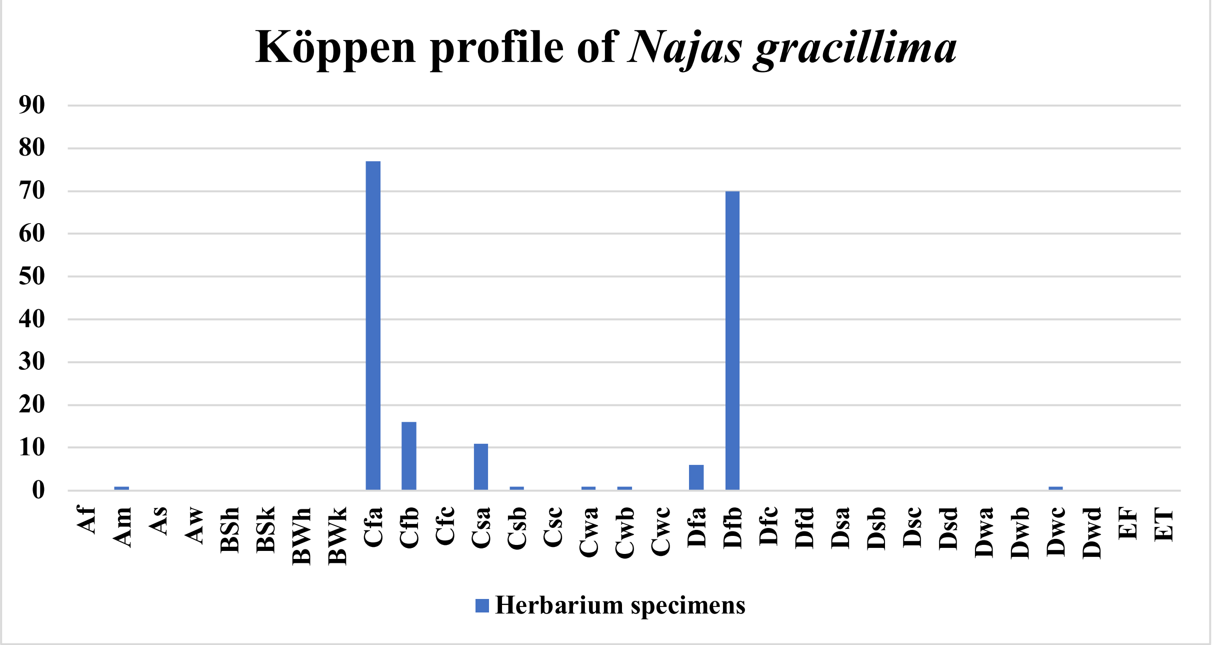

5.3.3.1.12. Köppen profile, distribution and climate map – GBIF occurrences of *Najas graminea* Delile 1814; herbarium specimens excluding duplicate occurrences (n = 202).

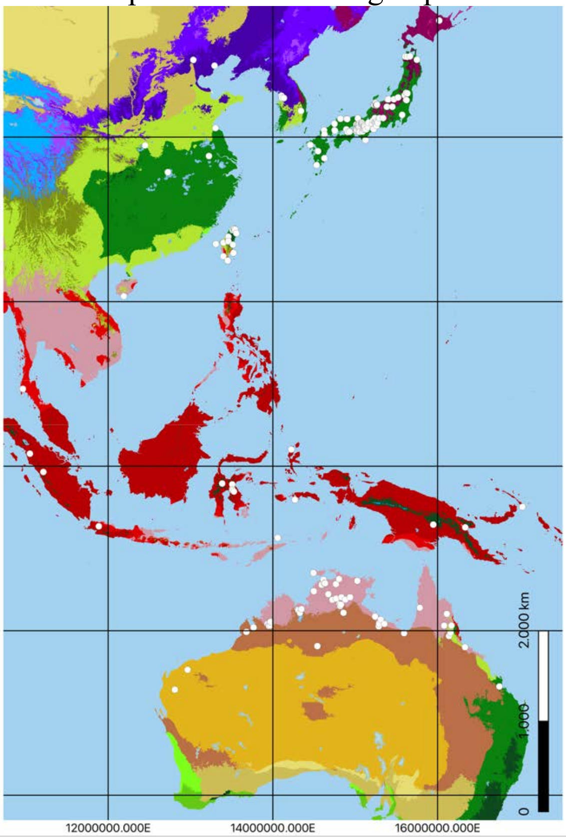

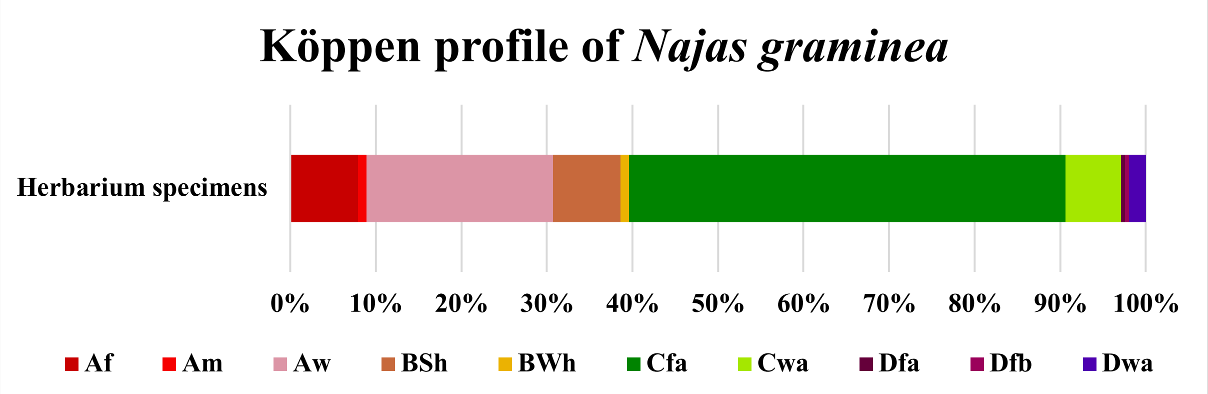

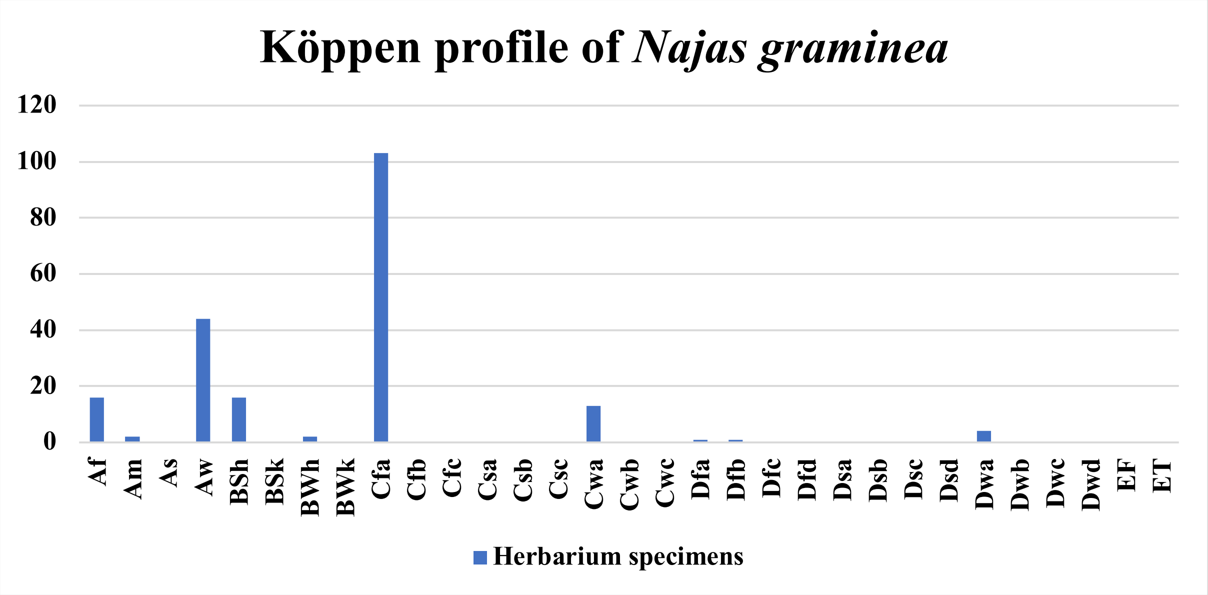

5.3.3.1.13. Köppen profile, distribution and climate map – GBIF occurrences of *Najas guadalupensis* (Spreng. 1824) Magnus 1870; herbarium specimens excluding duplicate occurrences (n = 1387).

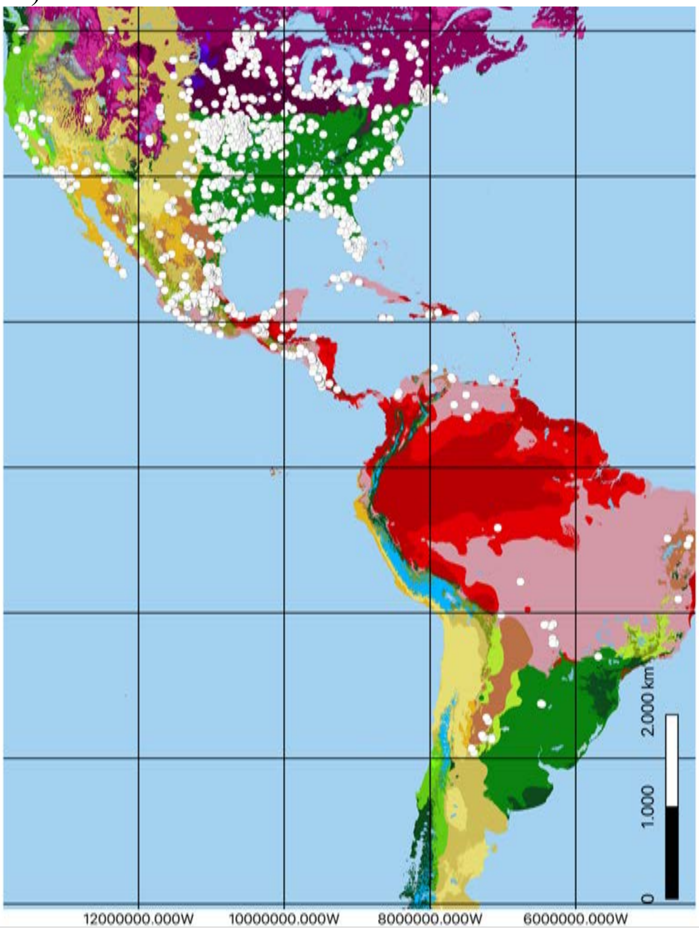

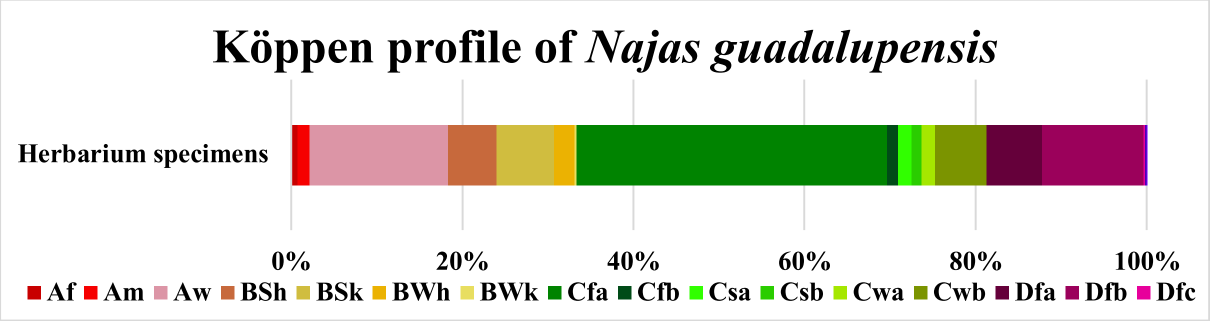

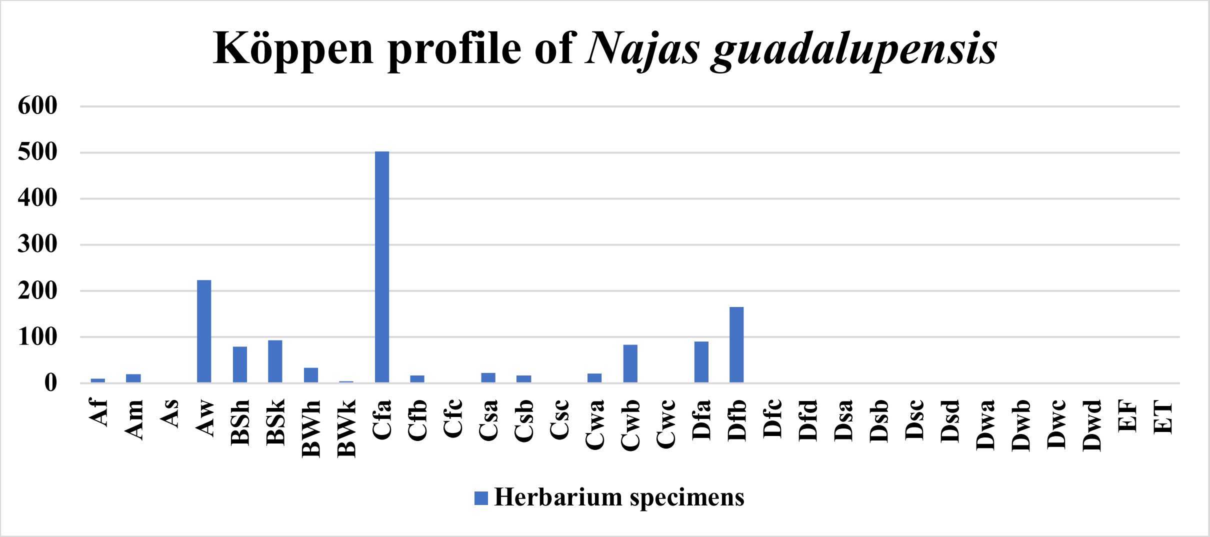

5.3.3.1.14. Köppen profile, distribution and climate map – GBIF occurrences of *Najas hagerupii* Horn 1952; herbarium specimens excluding duplicate occurrences (n = 3).

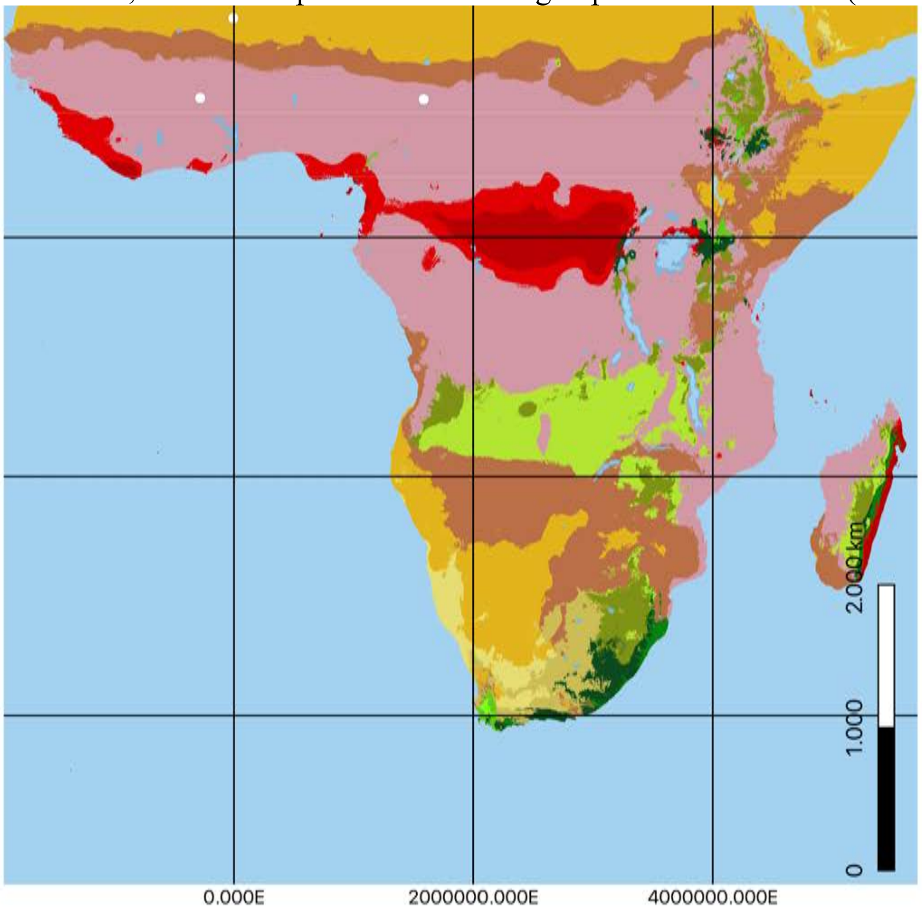

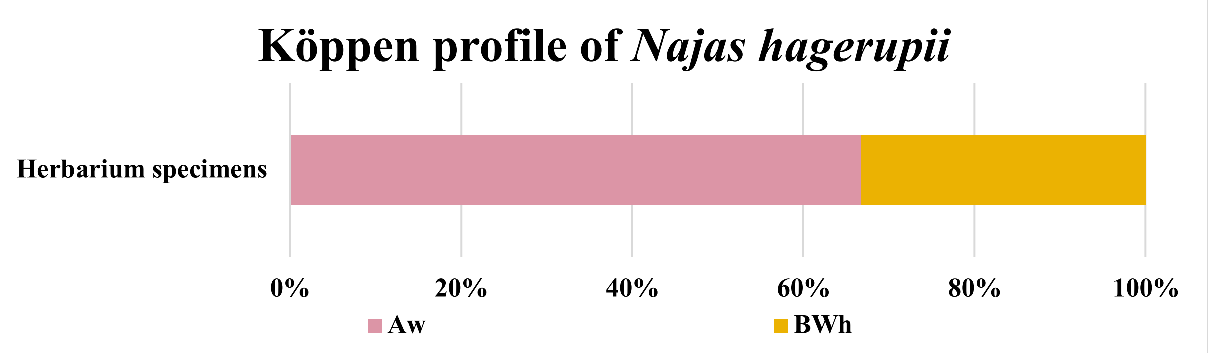

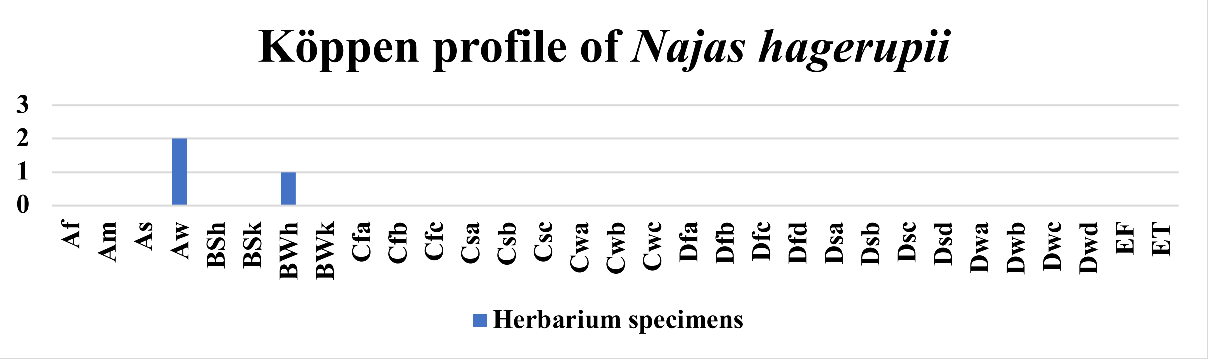

5.3.3.1.15. Köppen profile, distribution and climate map – GBIF occurrences of *Najas halophila* L.Triest 1988 herbarium specimens excluding duplicate occurrences (n = 1).

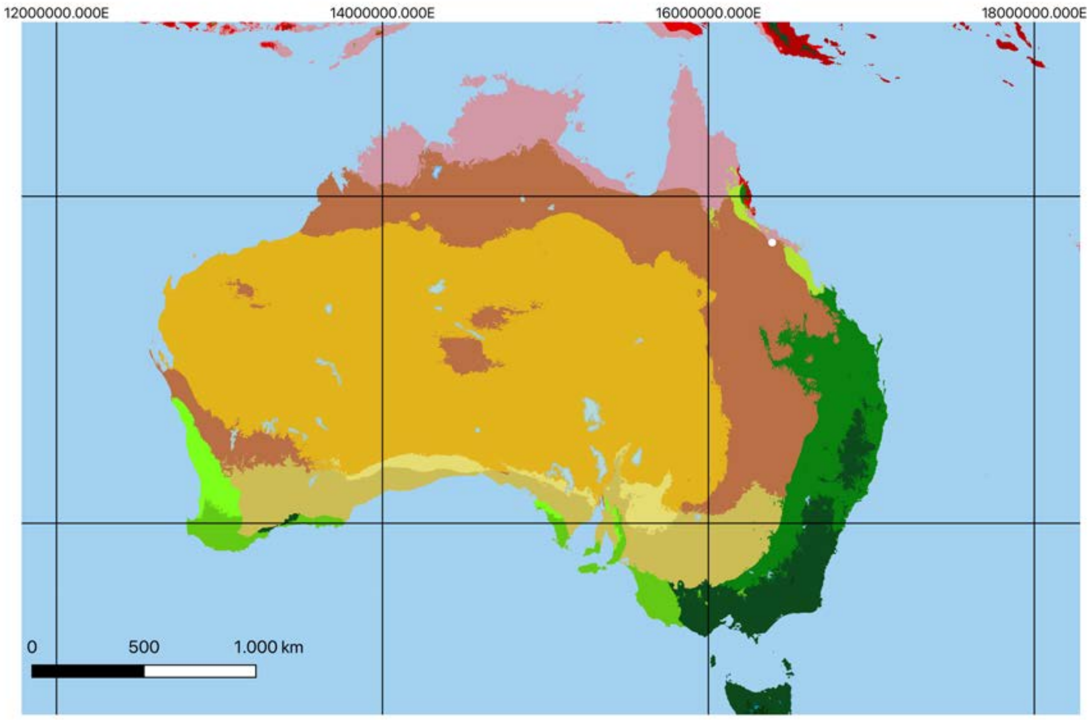

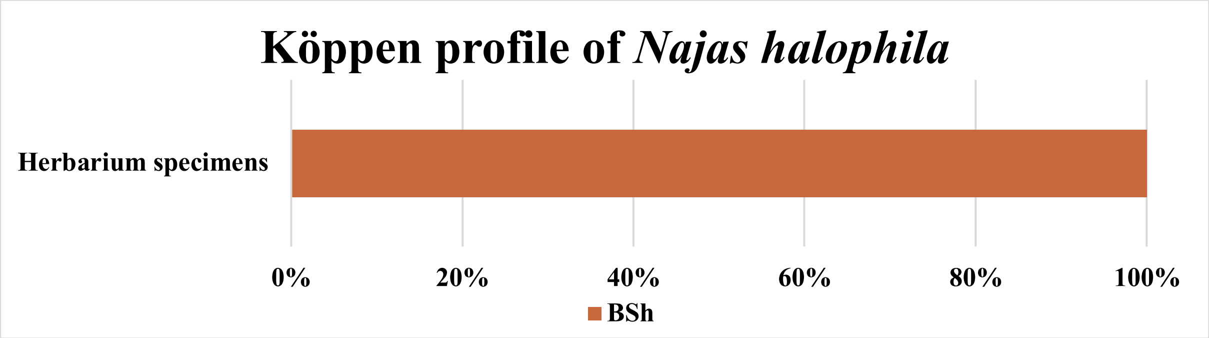

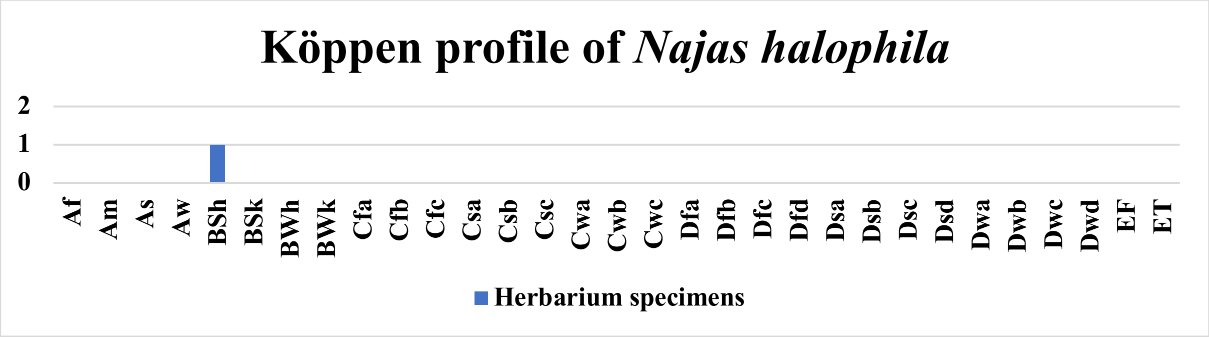

5.3.3.1.16. Köppen profile, distribution and climate map – GBIF occurrences of *Najas horrida* A.Braun 1870 herbarium specimens excluding duplicate occurrences (n = 12).

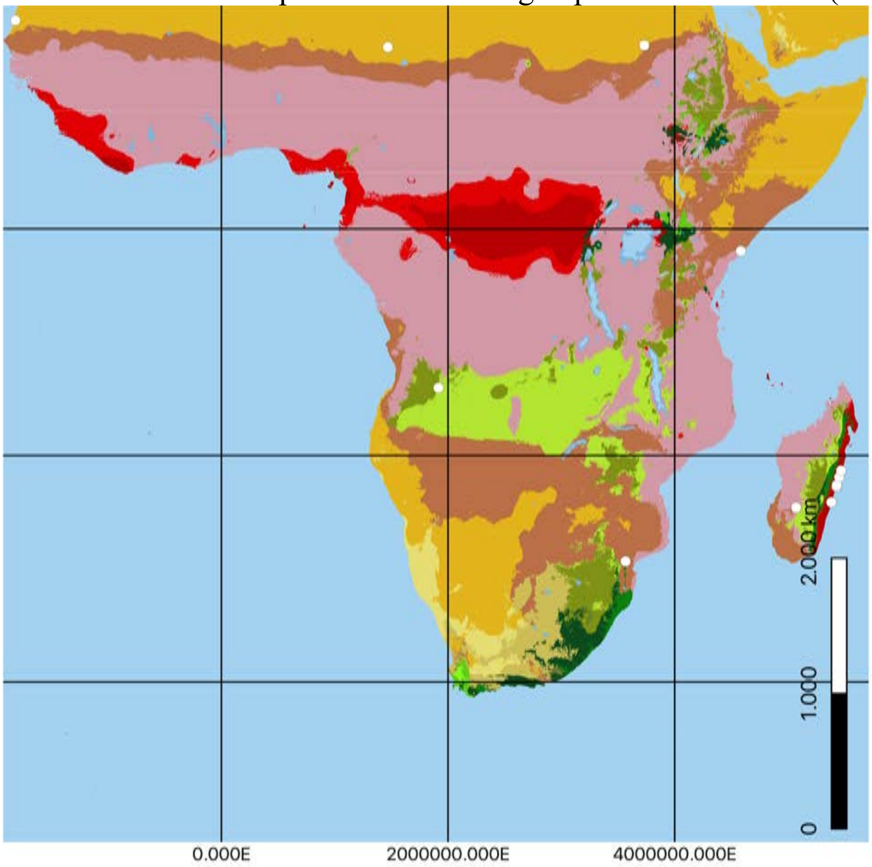

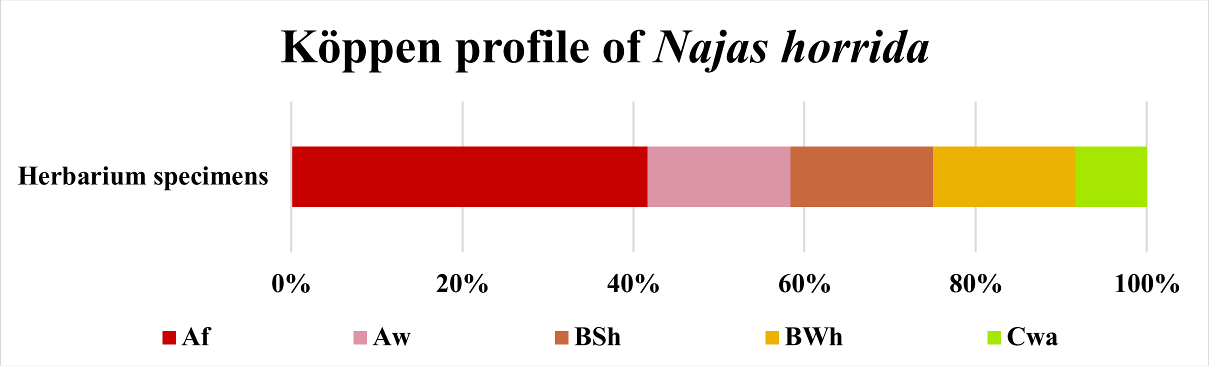

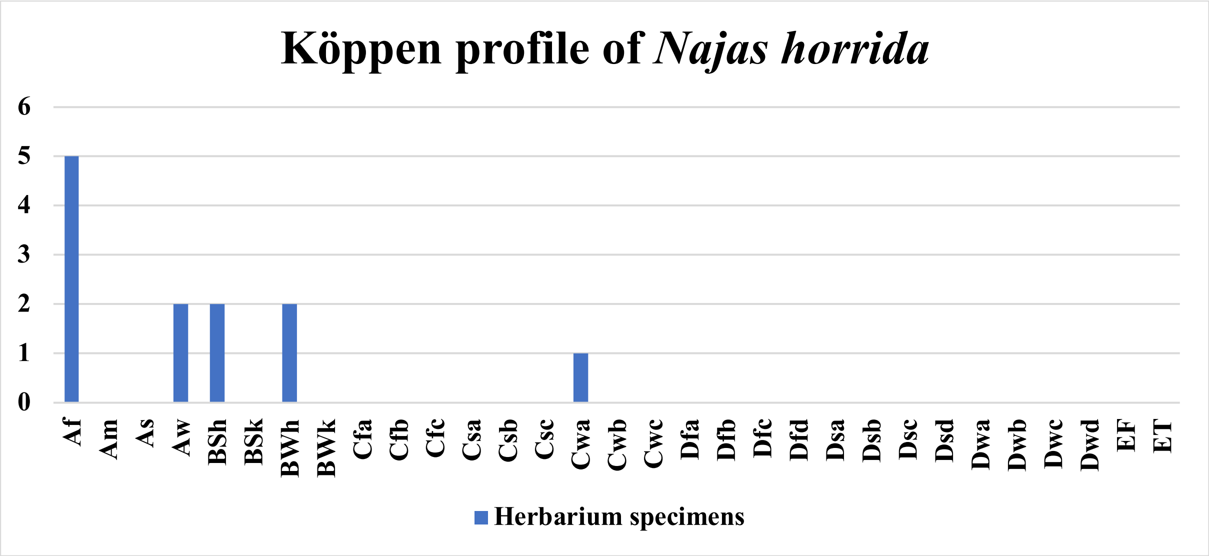

5.3.3.1.17. Köppen profile, distribution and climate map – GBIF occurrences of *Najas indica* (Willd. 1801) Cham. 1829; herbarium specimens excluding duplicate occurrences (n = 43).

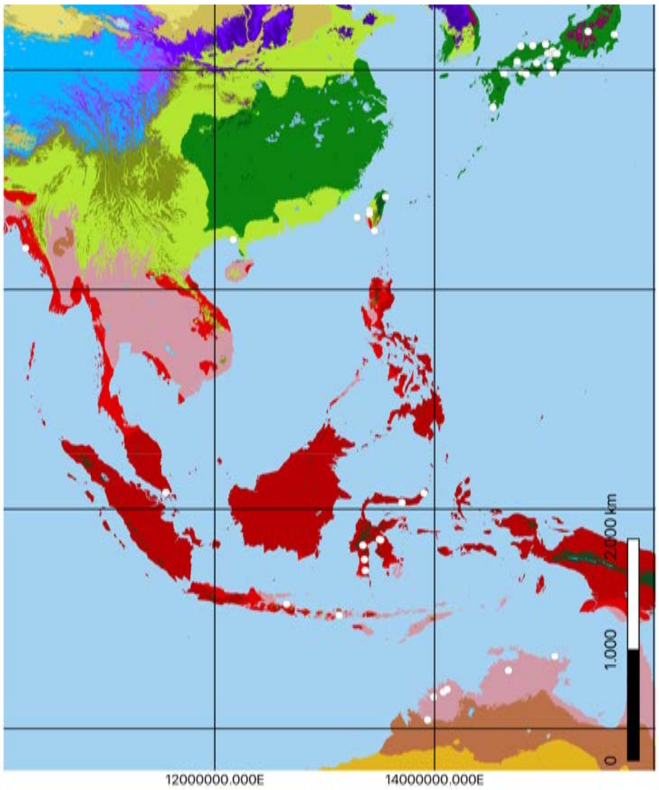

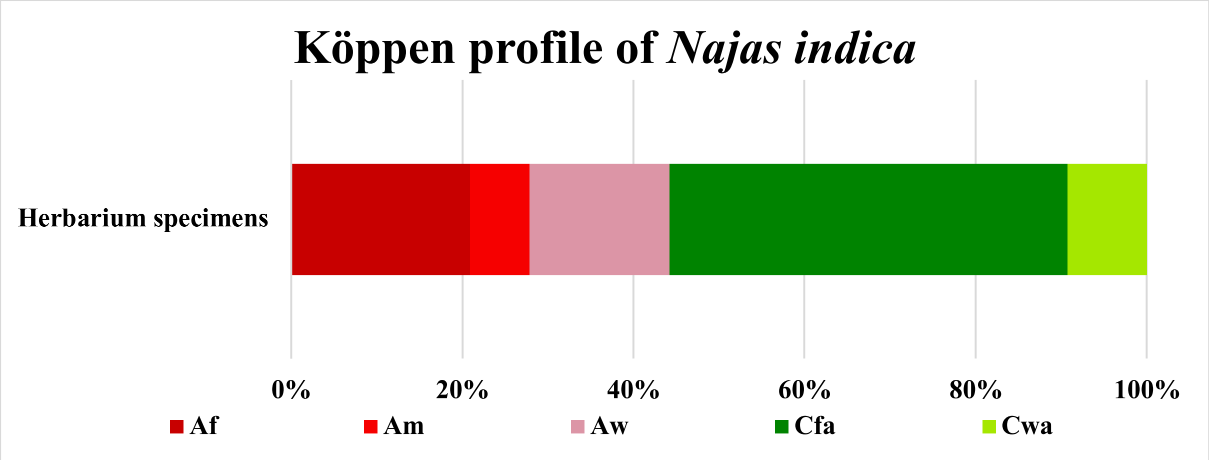

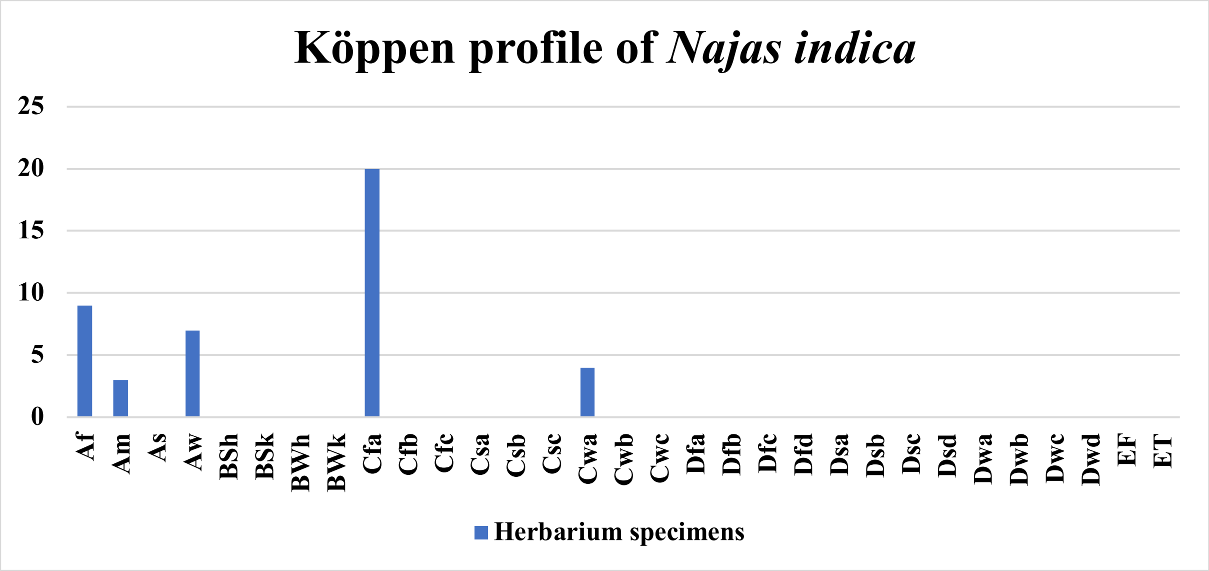
Köppen profile of *Najas indica*
25
20
15
10
5
0
Af
Am
As
Aw
BSh
BSk
BWh
BWk
Cfa
Cfb
Cfc
Csa
Csb
Csc
Cwa
Cwb
Cwc
Dfa
Dfb
Dfc
Dfd
Dsa
Dsb
Dsc
Dsd
Dwa
Dwb
Dwc
Dwd
EF
ET
Herbarium specimens

5.3.3.1.18. Köppen profile, distribution and climate map – GBIF occurrences of *Najas kurziana* Rendle 1899; herbarium specimens excluding duplicate occurrences (n = 1).

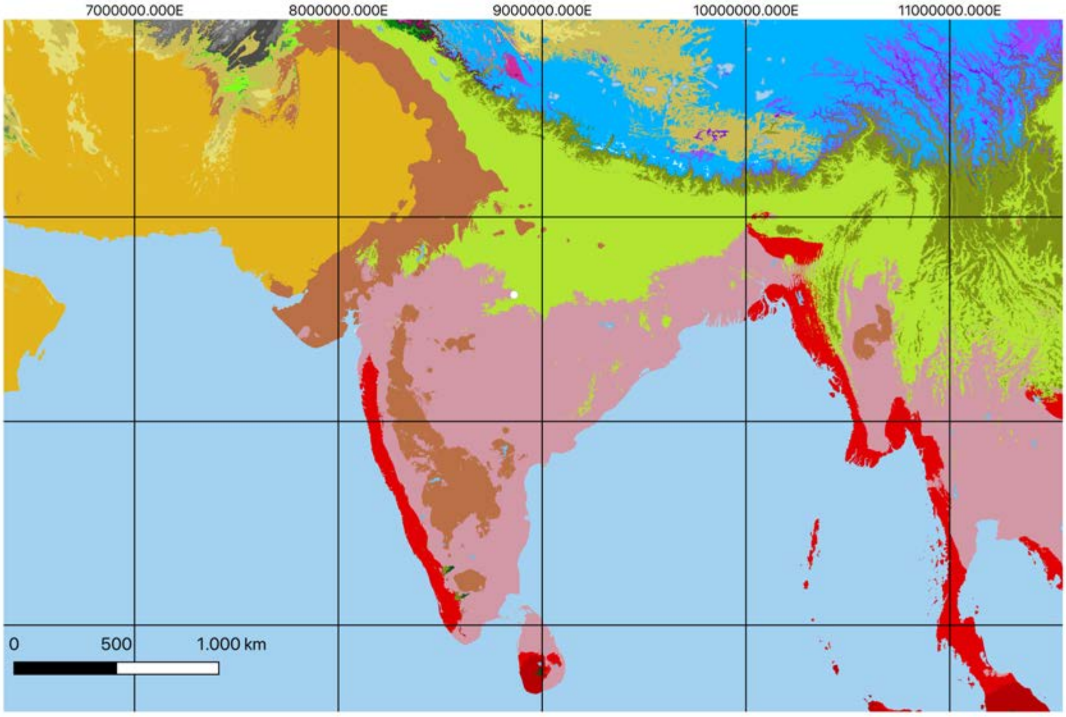

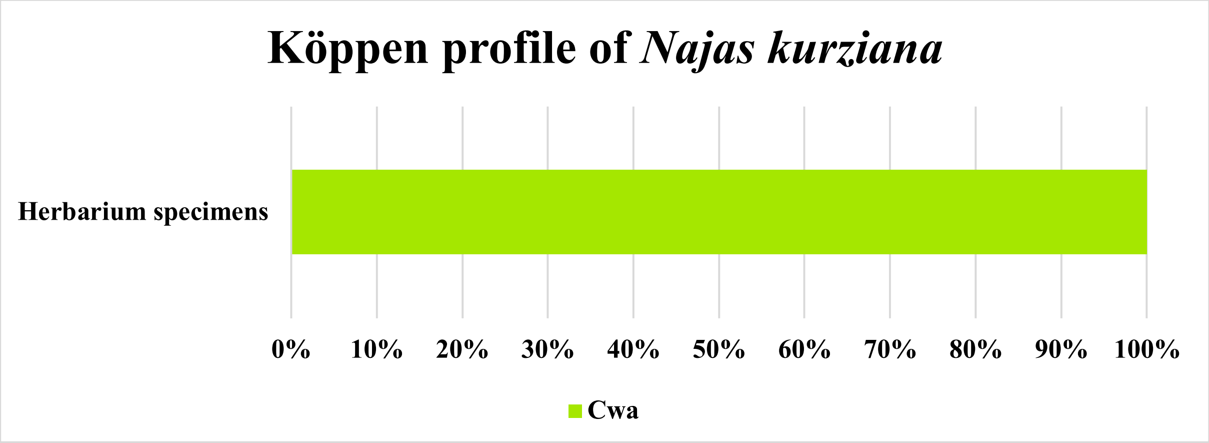

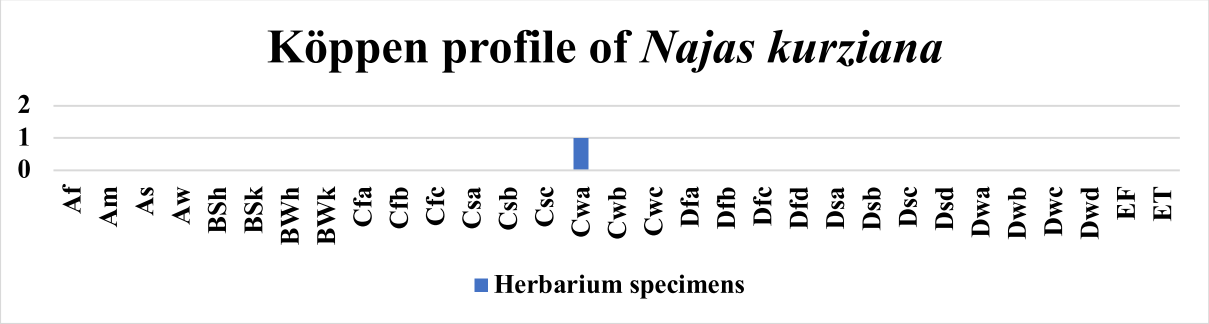

5.3.3.1.19. Köppen profile, distribution and climate map – GBIF occurrences of *Najas madagascariensis* Rendle 1889; herbarium specimens excluding duplicate occurrences (n = 7).

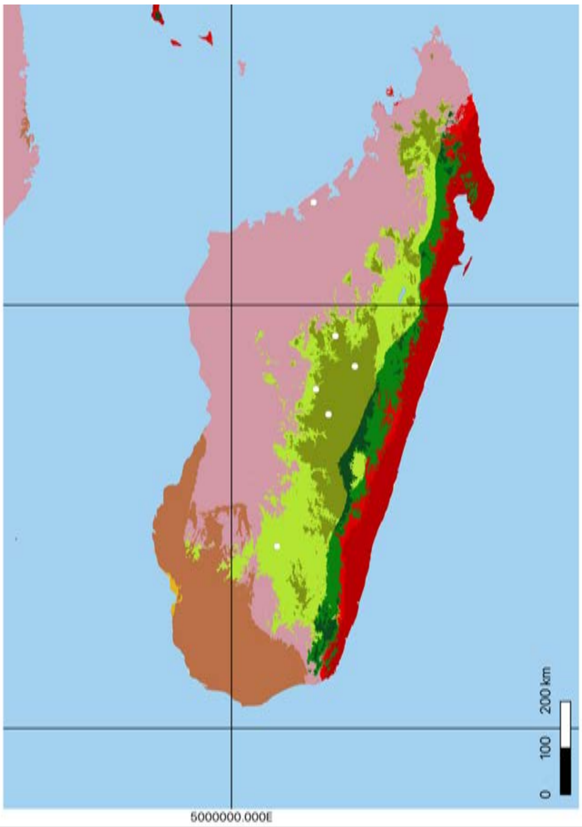

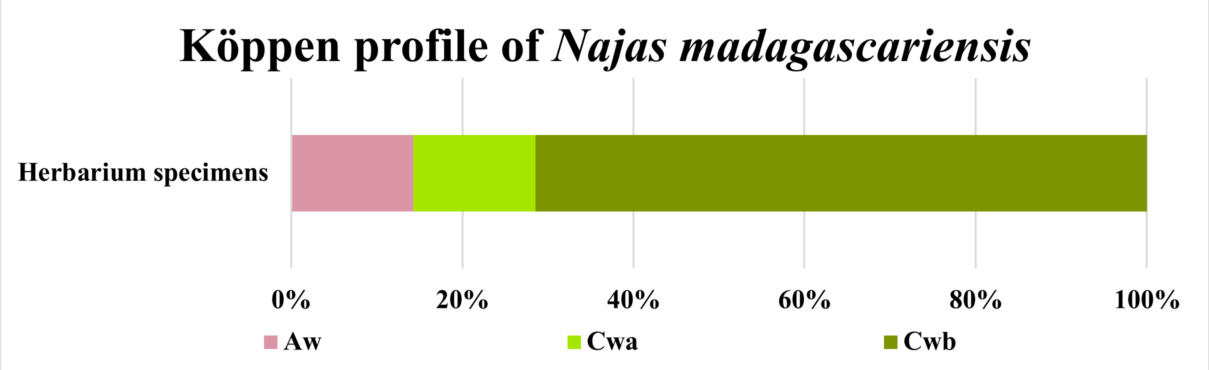

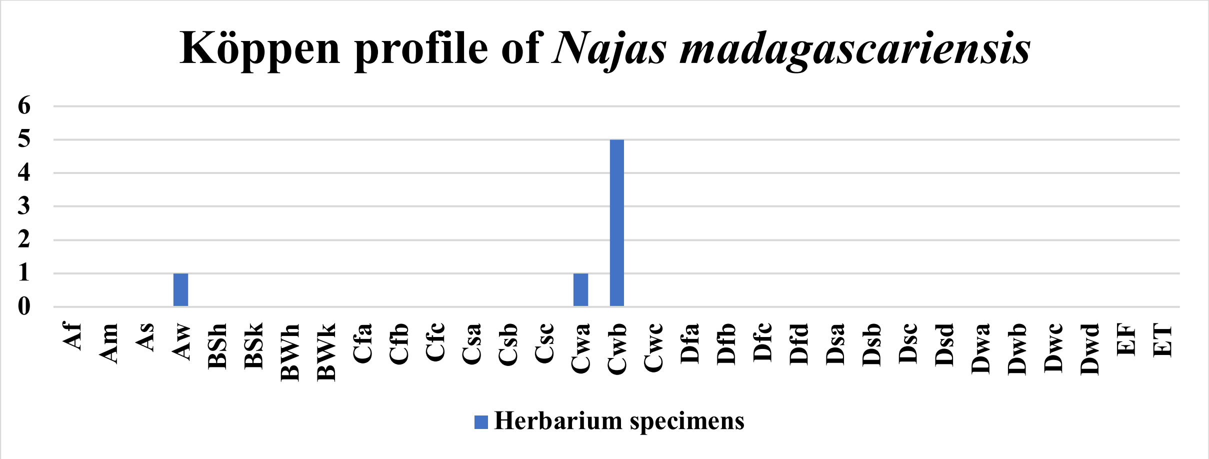

5.3.3.1.20. Köppen profile, distribution and climate map – GBIF occurrences of *Najas malesiana* W.J.de Wilde 1968; herbarium specimens excluding duplicate occurrences (n = 47).

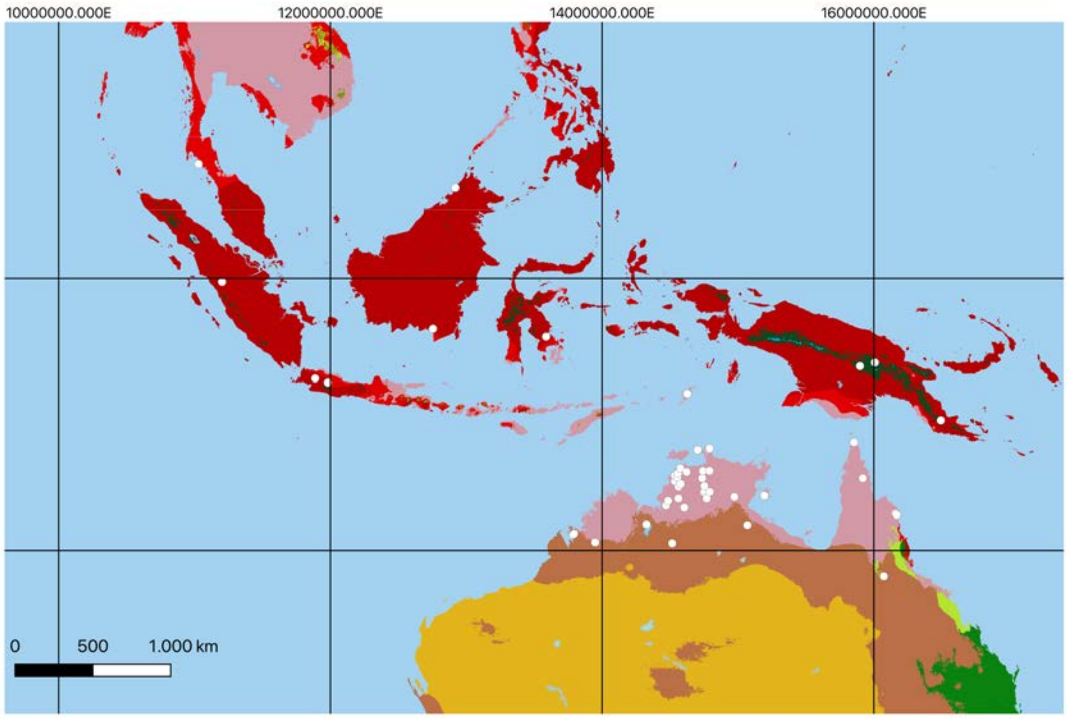

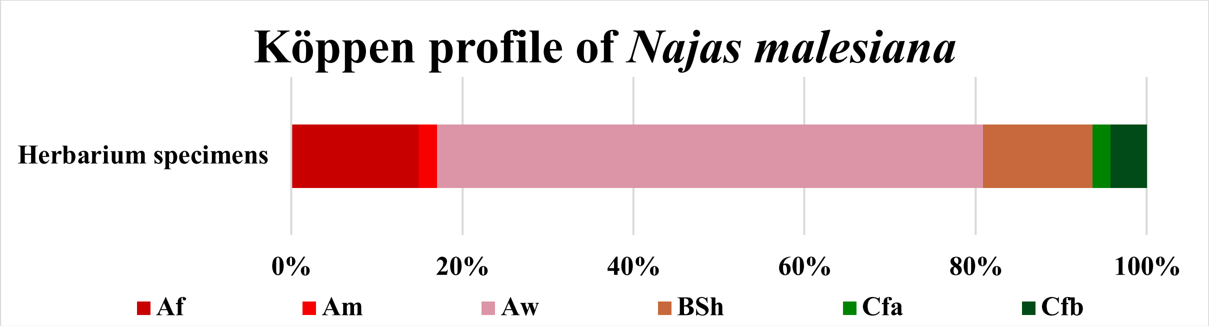

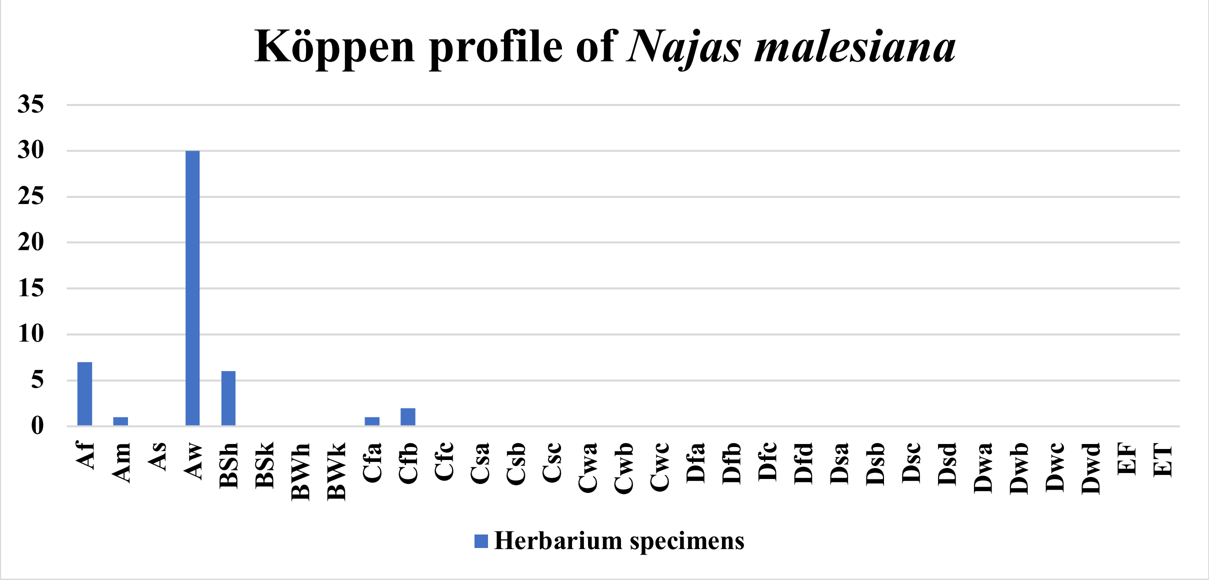

5.3.3.1.21. Köppen profile, distribution and climate map – GBIF occurrences of *Najas marina* L. 1753; herbarium specimens excluding duplicate occurrences (n = 1441).

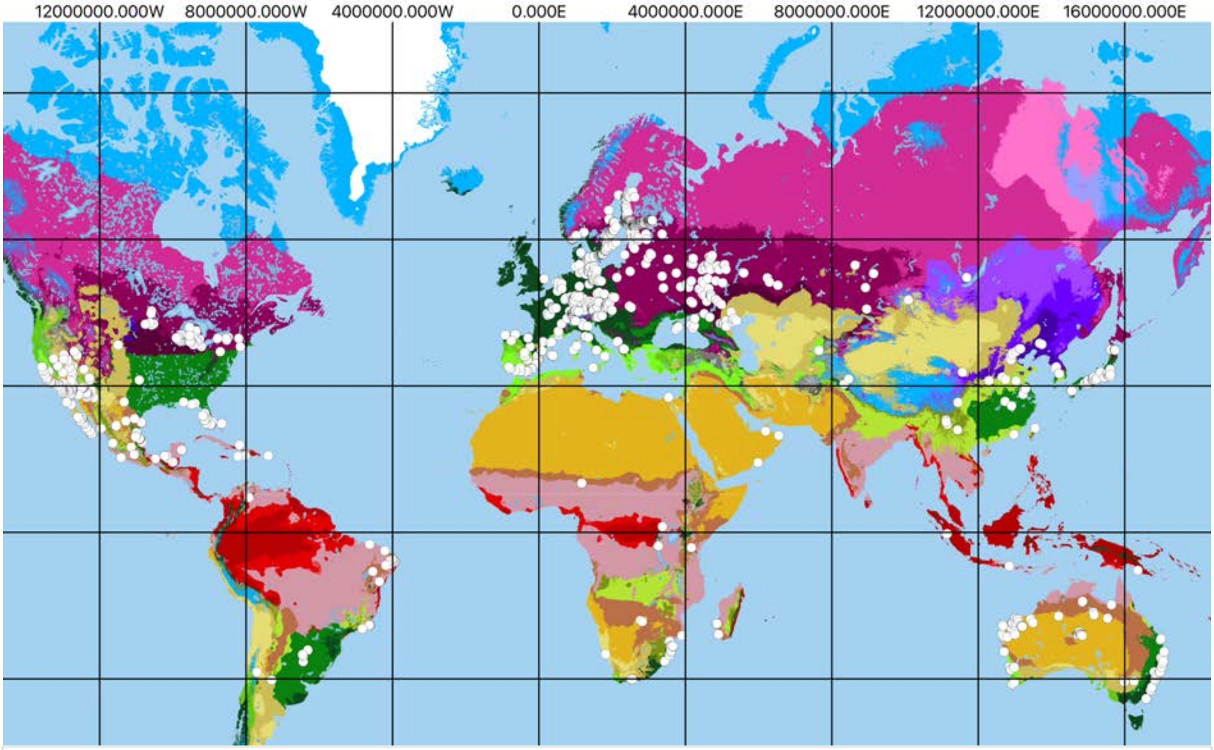

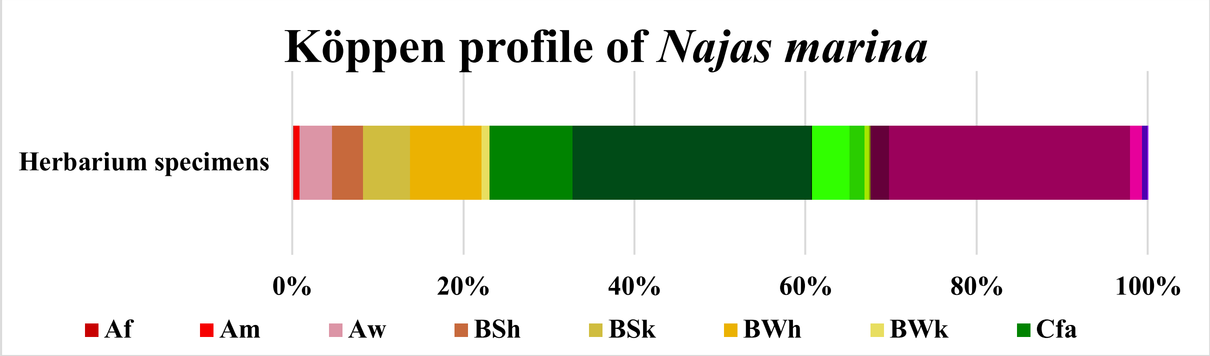

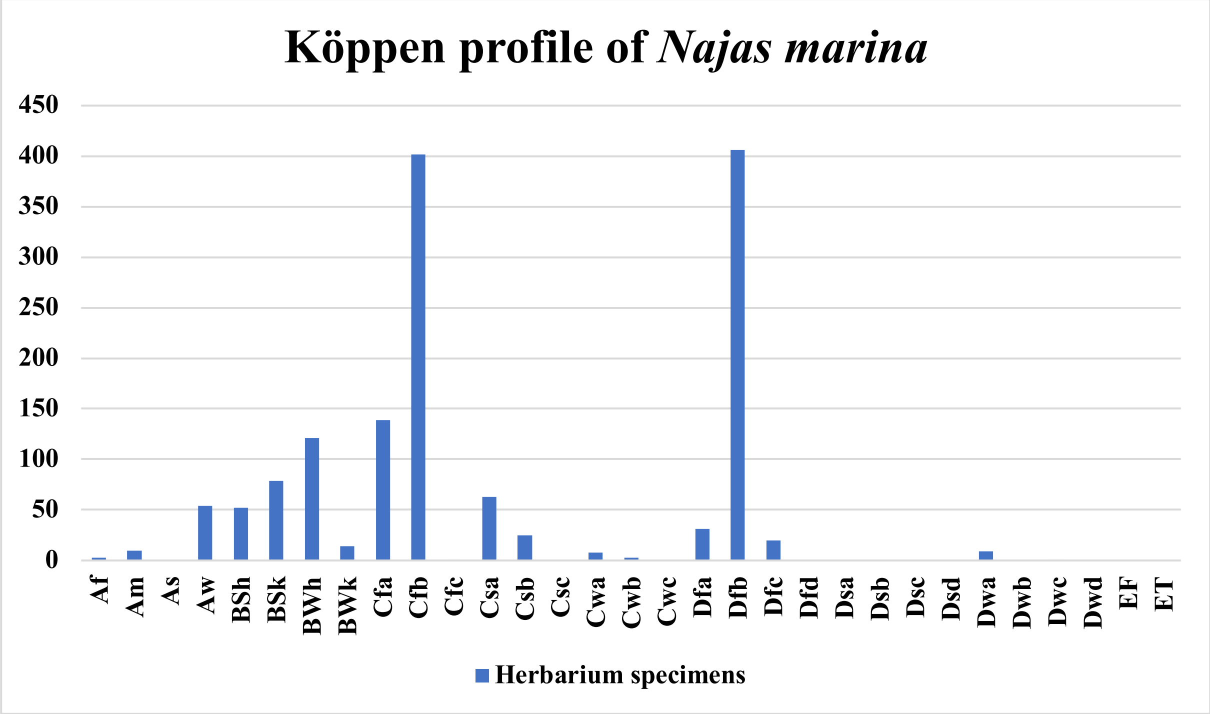

5.3.3.1.22. Köppen profile, distribution and climate map – GBIF occurrences of *Najas oguraensis* Miki 1935; herbarium specimens excluding duplicate occurrences (n = 31).

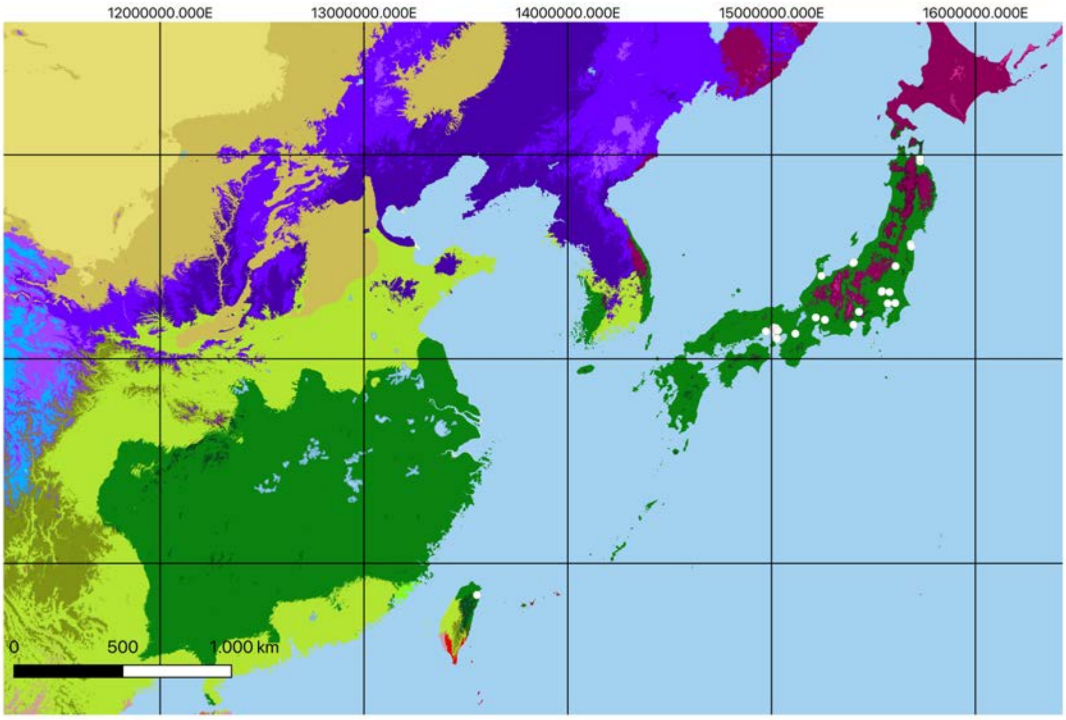

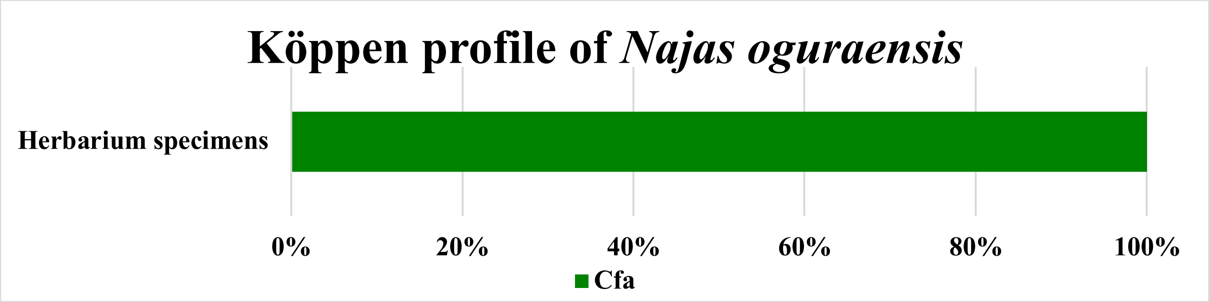

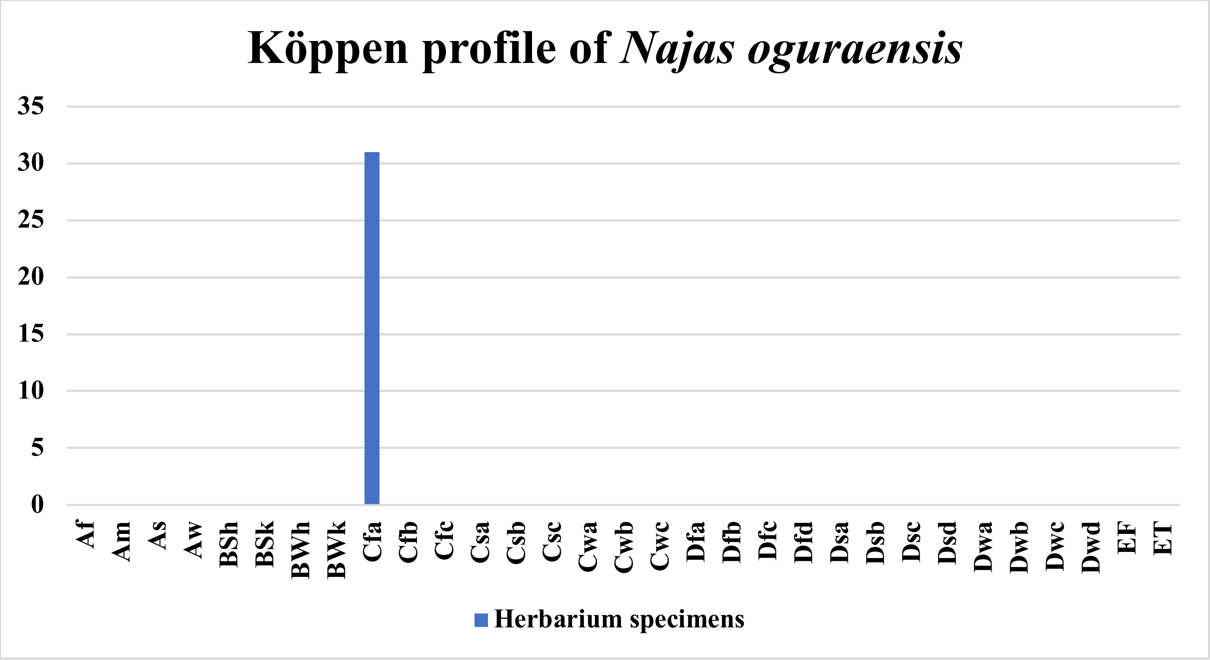

5.3.3.1.23. Köppen profile, distribution and climate map – GBIF occurrences of *Najas pectinate* (Parl. 1860) Magnus 1887; herbarium specimens excluding duplicate occurrences (n = 67).

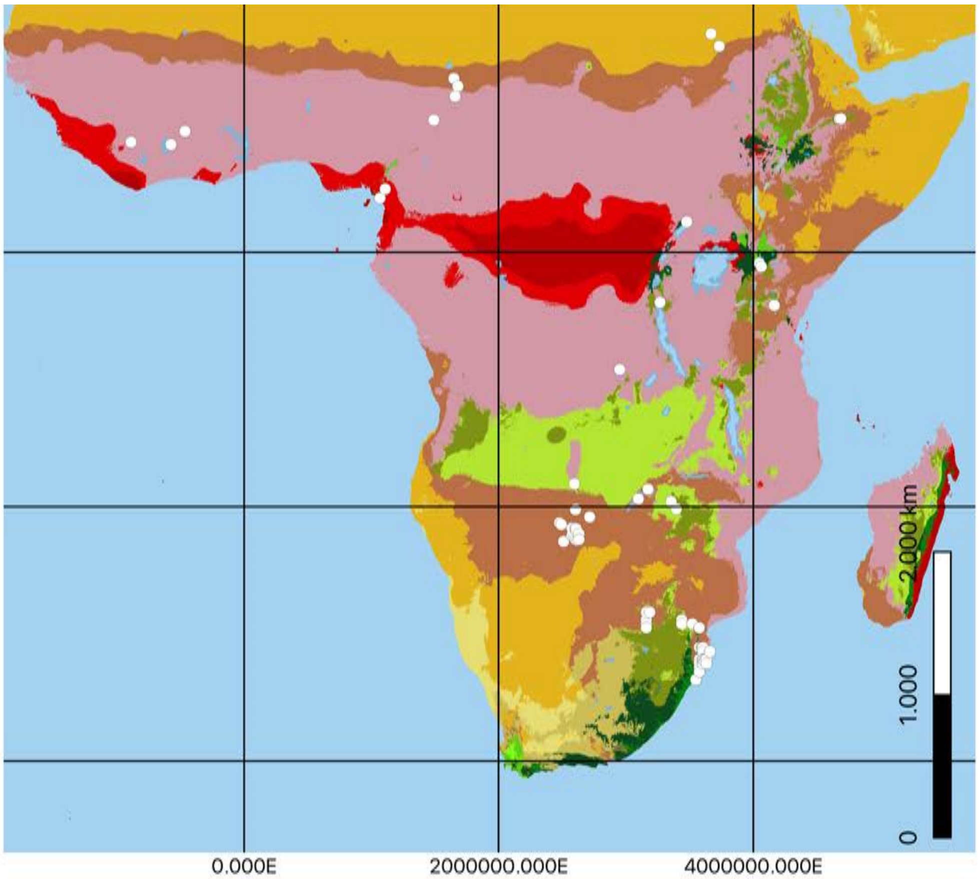

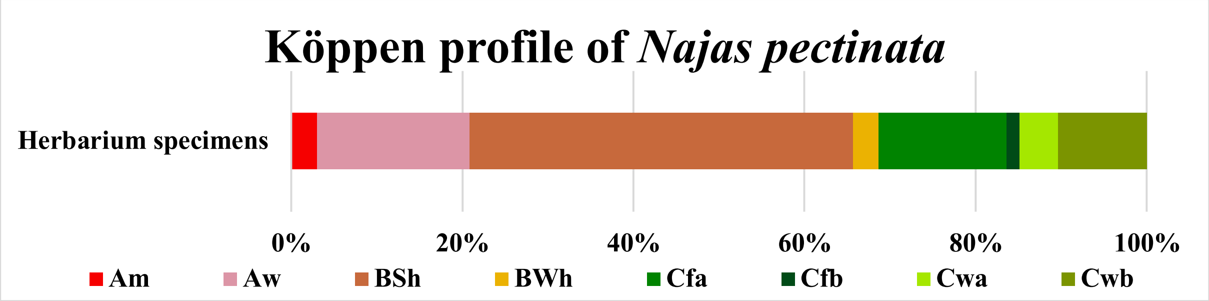

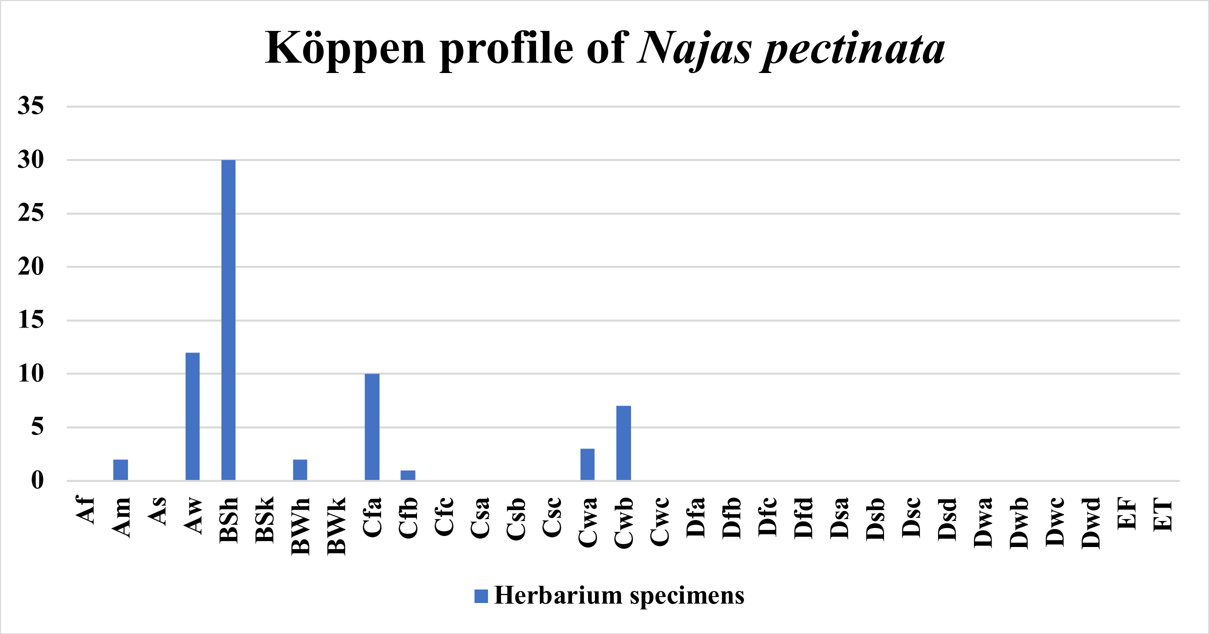
Köppen profile of *Najas pectinata*
35
30
25
20
15
10
5
0
Af
Am
As
Aw
BSh
BSk
BWh
BWk
Cfa
Cfb
Cfc
Csa
Csb
Csc
Cwa
Cwb
Cwc
Dfa
Dfb
Dfc
Dfd
Dsa
Dsb
Dsc
Dsd
Dwa
Dwb
Dwc
Dwd
EF
ET
Herbarium specimens

5.3.3.1.24. Köppen profile, distribution and climate map – GBIF occurrences of *Najas schweinfurthii* Magnus 1894; herbarium specimens excluding duplicate occurrences (n = 3).

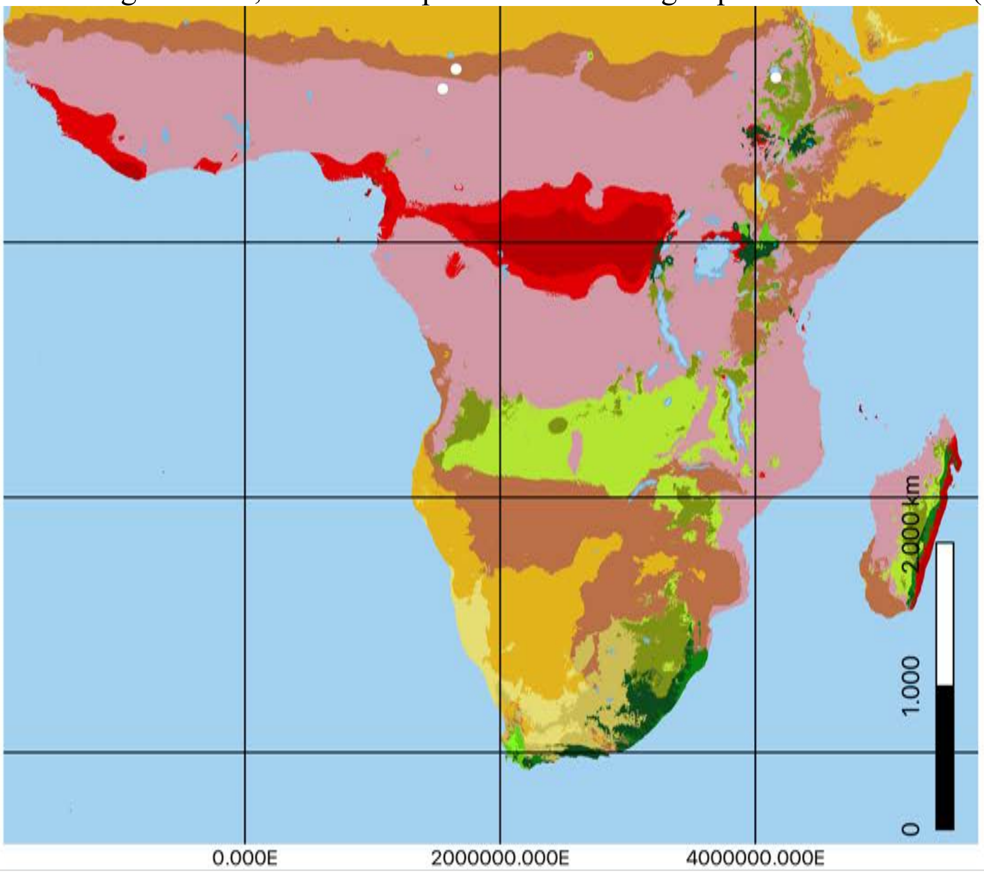

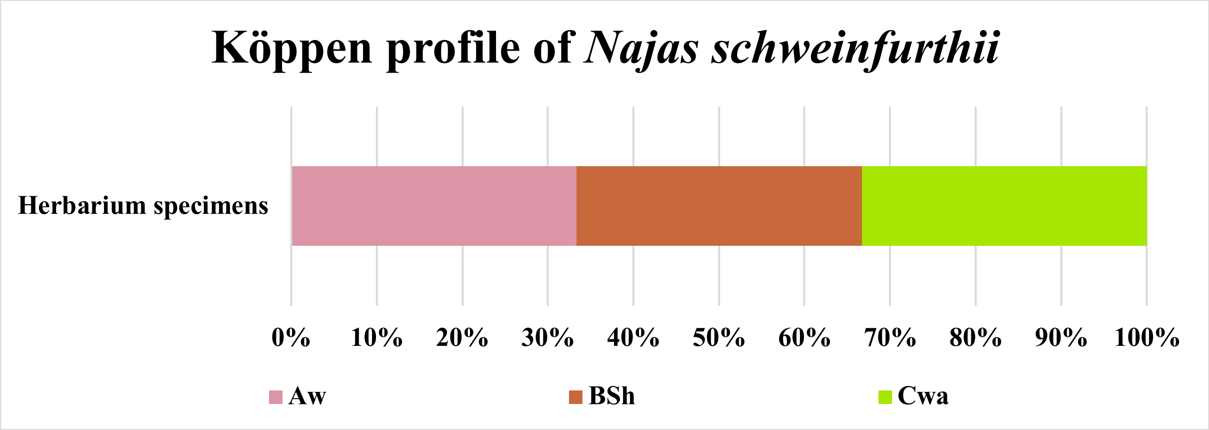

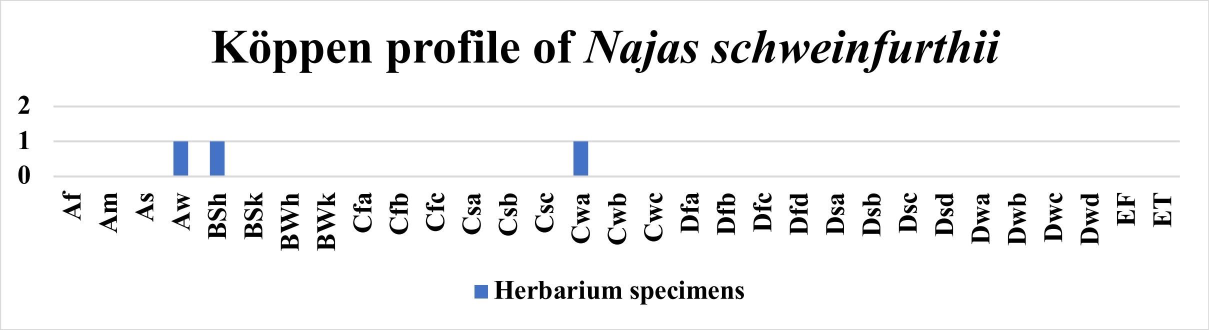

5.3.3.1.25. Köppen profile, distribution and climate map – GBIF occurrences of *Najas tenuicaulis* Miki 1935; herbarium specimens excluding duplicate occurrences (n = 1).

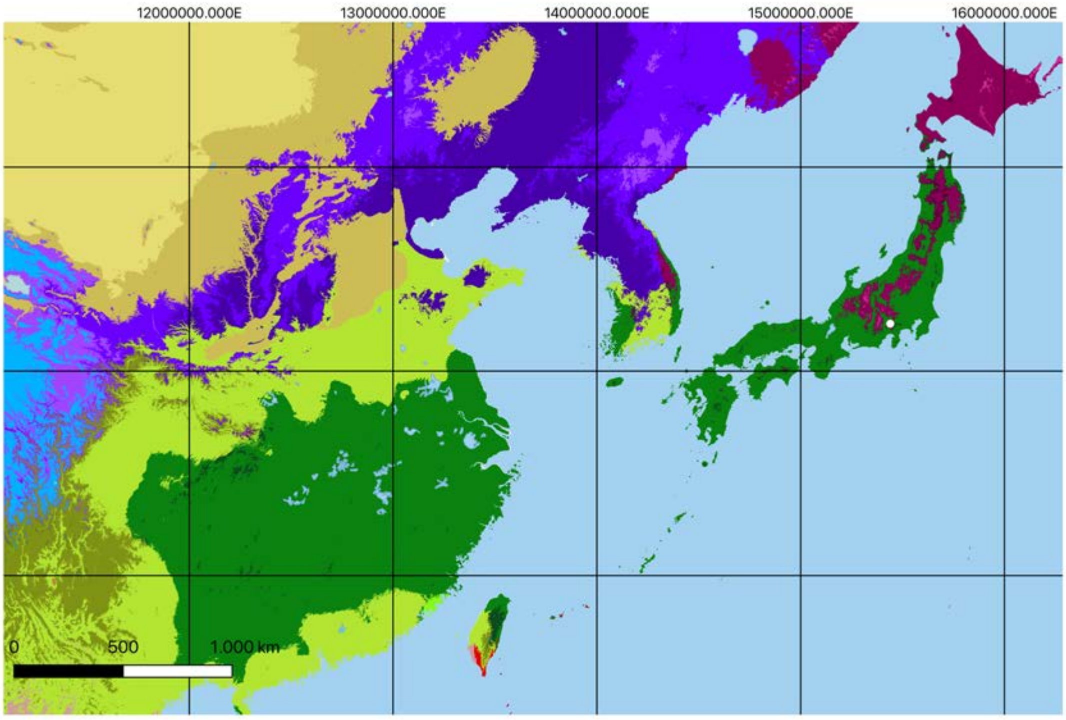

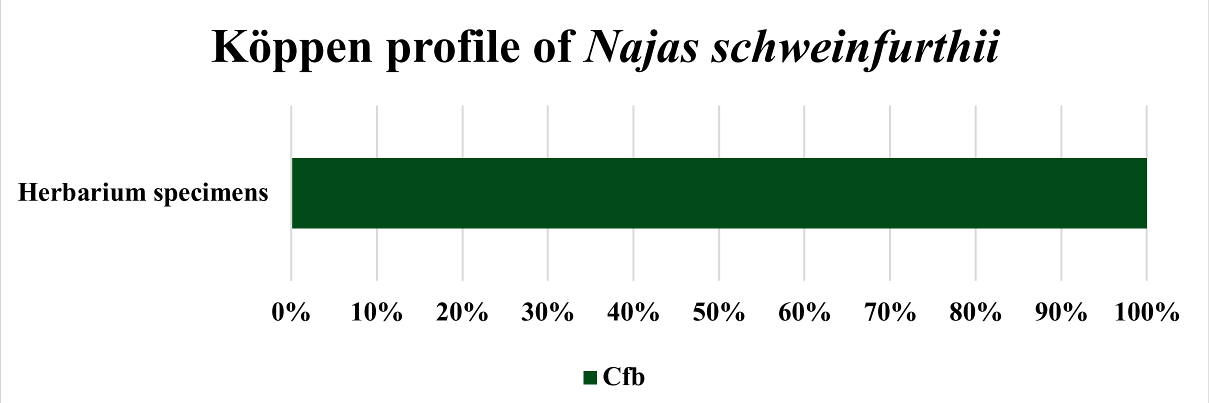

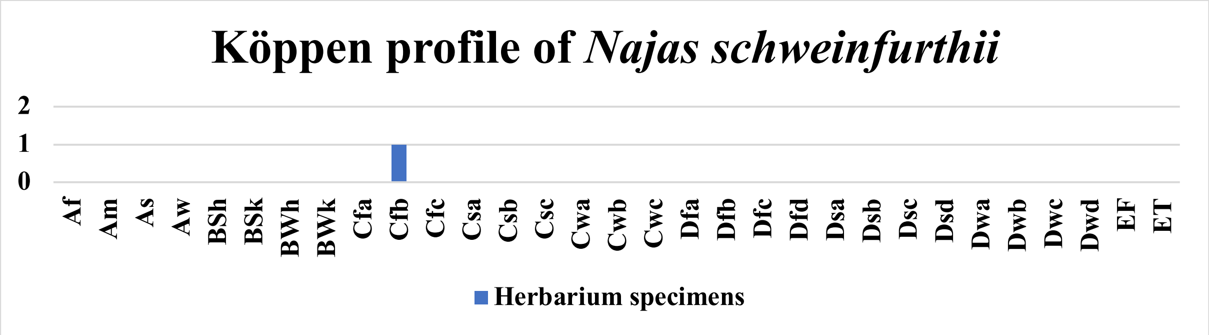

5.3.3.1.26. Köppen profile, distribution and climate map – GBIF occurrences of *Najas tenuifolia* R.Br., 1810; herbarium specimens excluding duplicate occurrences (n = 367).

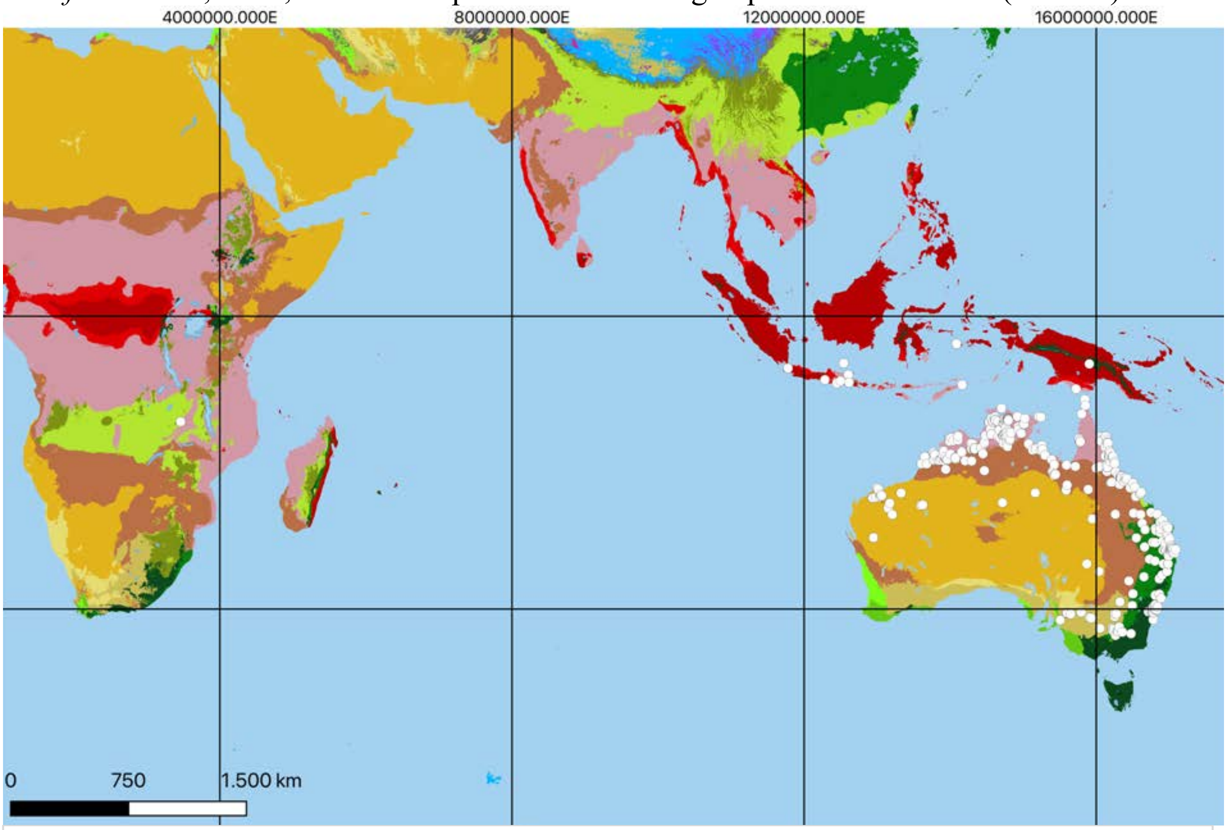

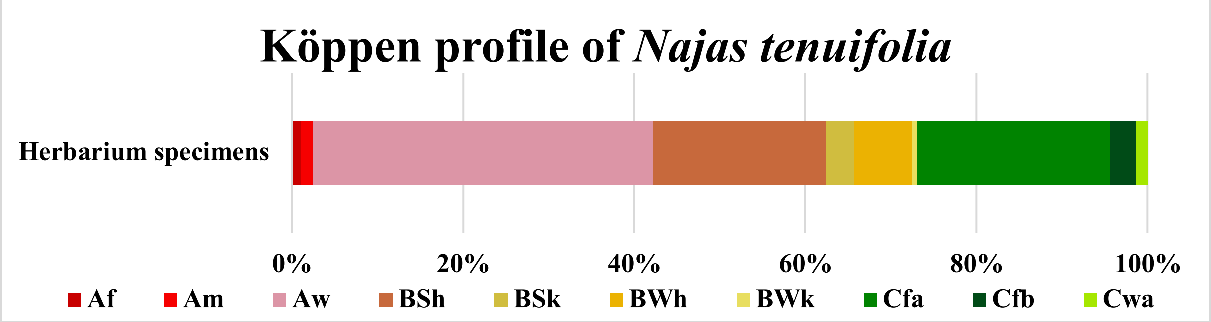

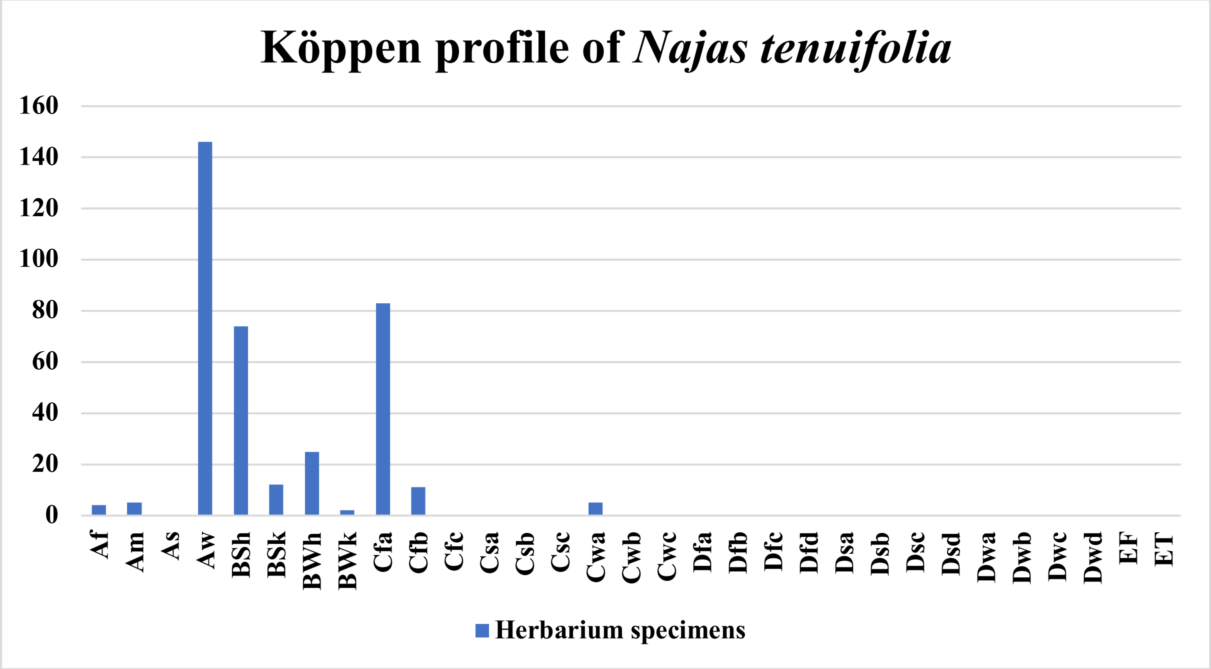

5.3.3.1.27. Köppen profile, distribution and climate map – GBIF occurrences of *Najas tenuissima* (A.Braun ex Magnus 1868) Magnus 1870; herbarium specimens excluding duplicate occurrences (n = 129).

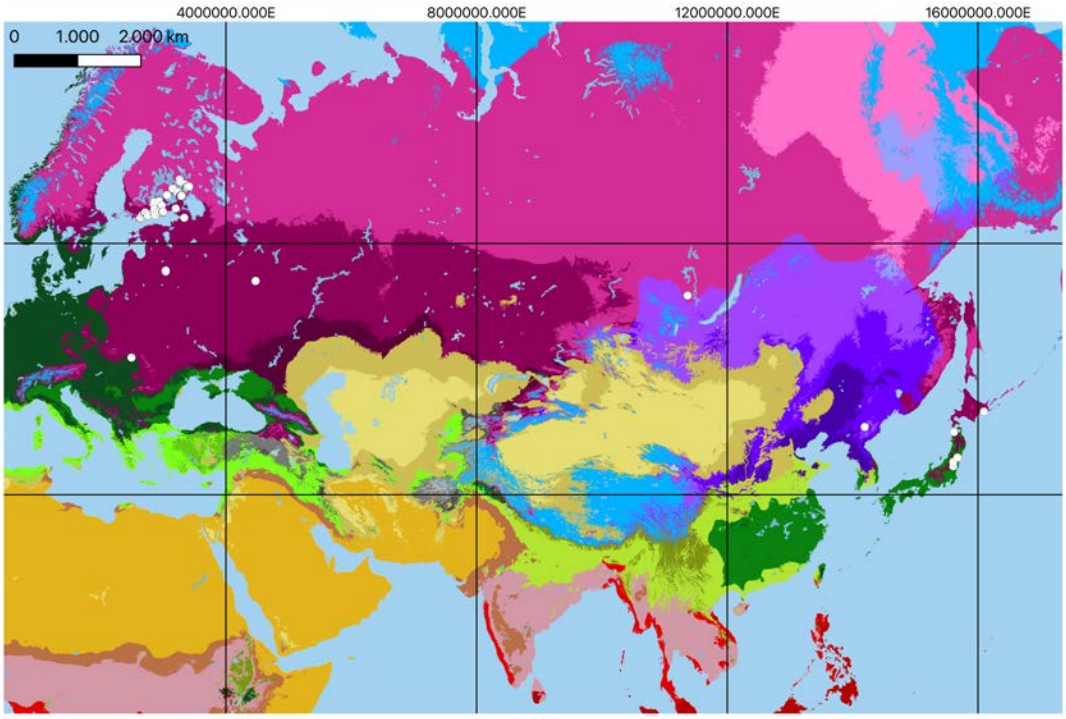

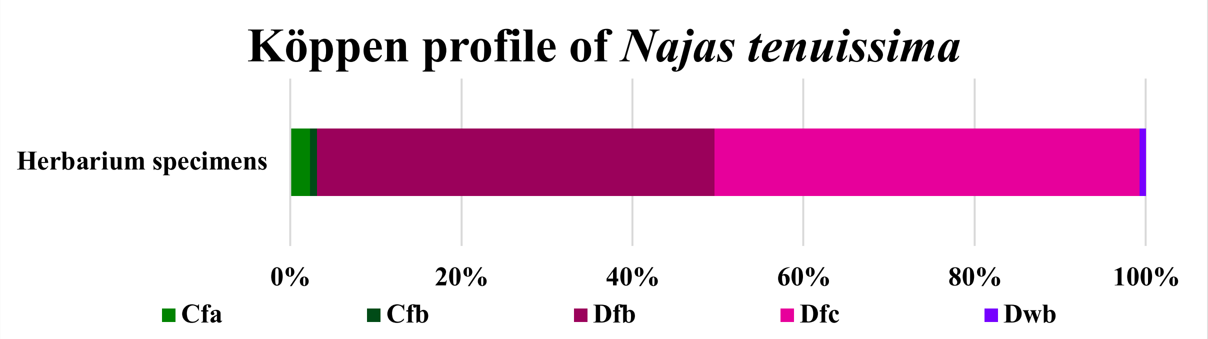

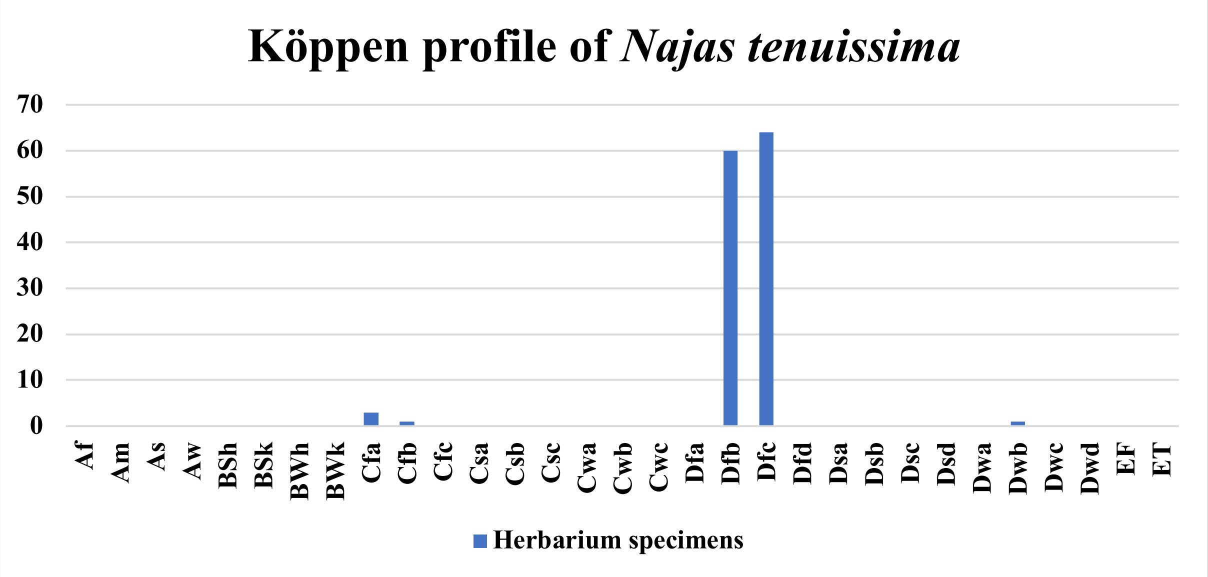

5.3.3.1.28. Köppen profile, distribution and climate map – GBIF occurrences of *Najas testui* Rendle 1937; herbarium specimens excluding duplicate occurrences (n = 4).

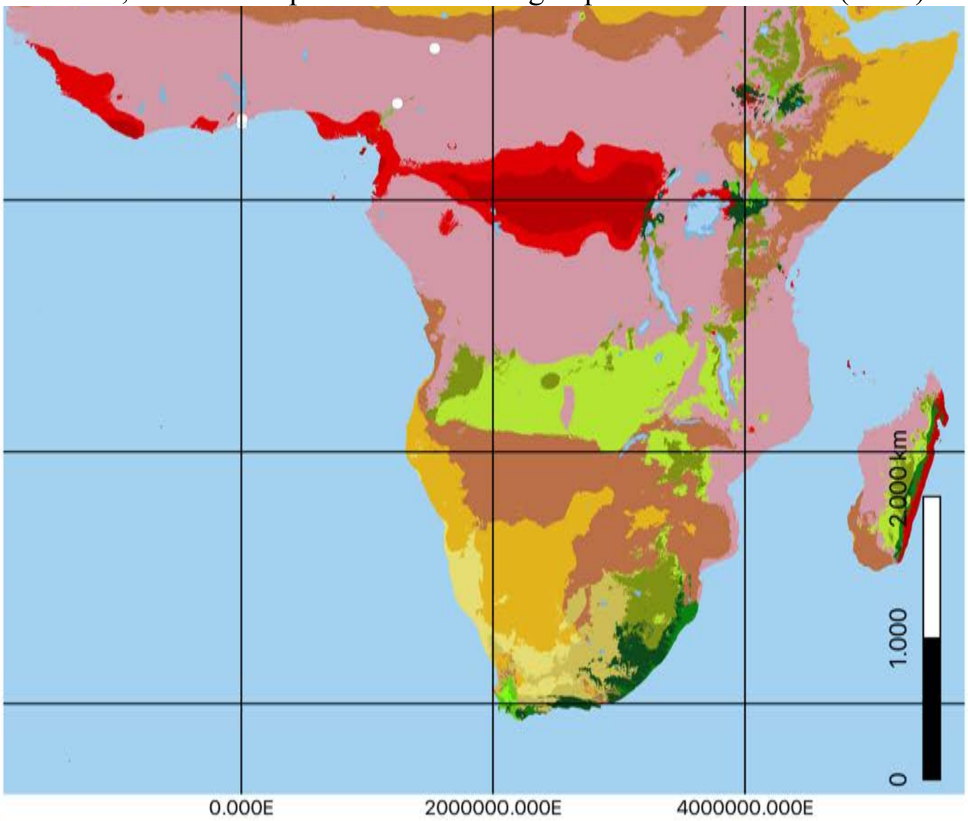

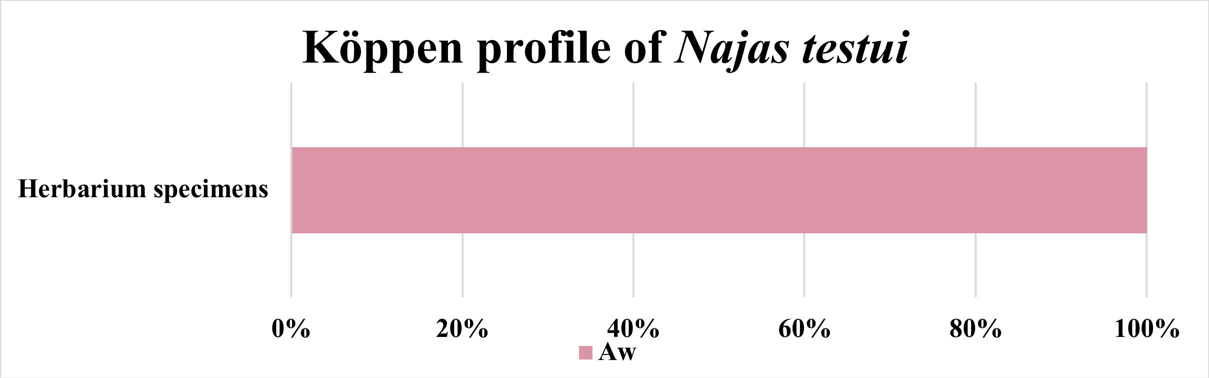

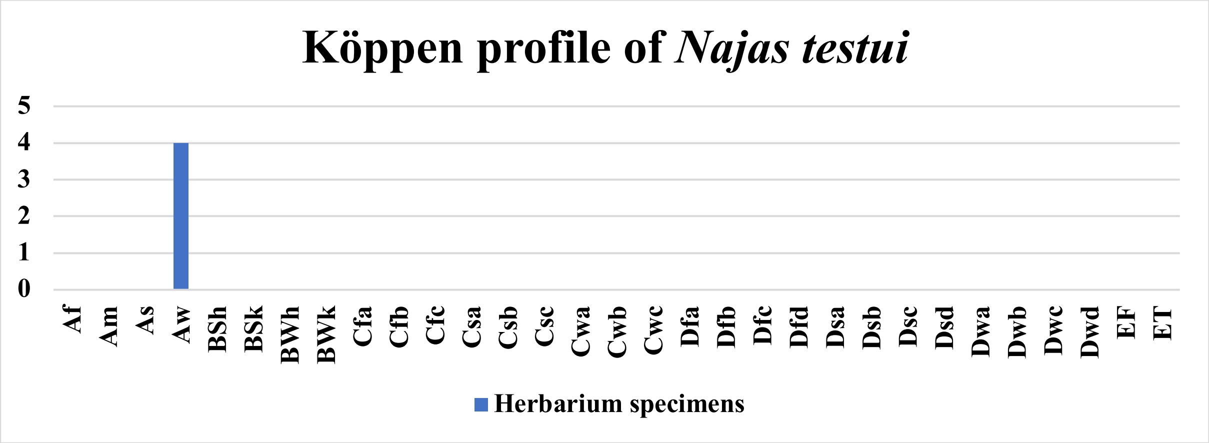

5.3.3.1.29. Köppen profile, distribution and climate map – GBIF occurrences of *Najas welwitschia* Rendle 1899; herbarium specimens excluding duplicate occurrences (n = 7).

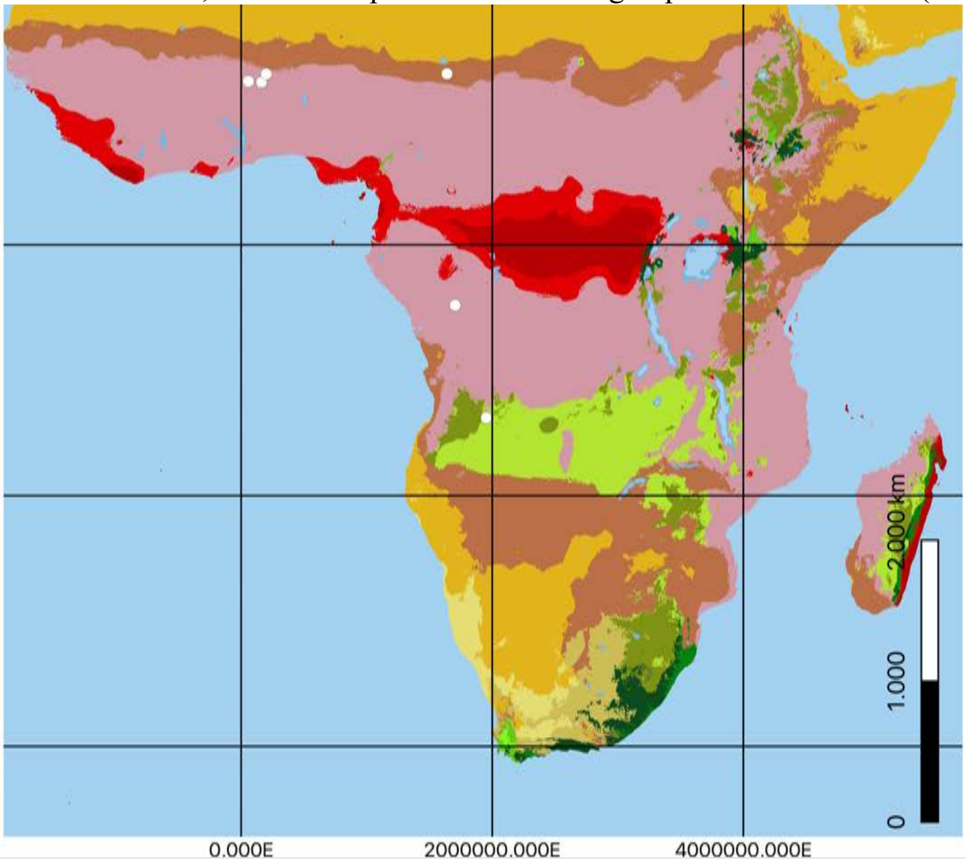

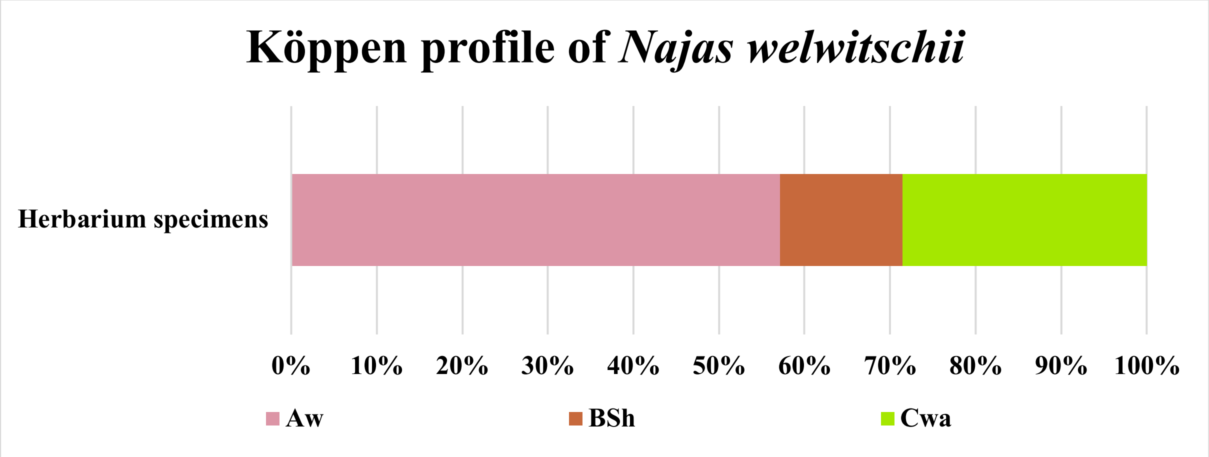

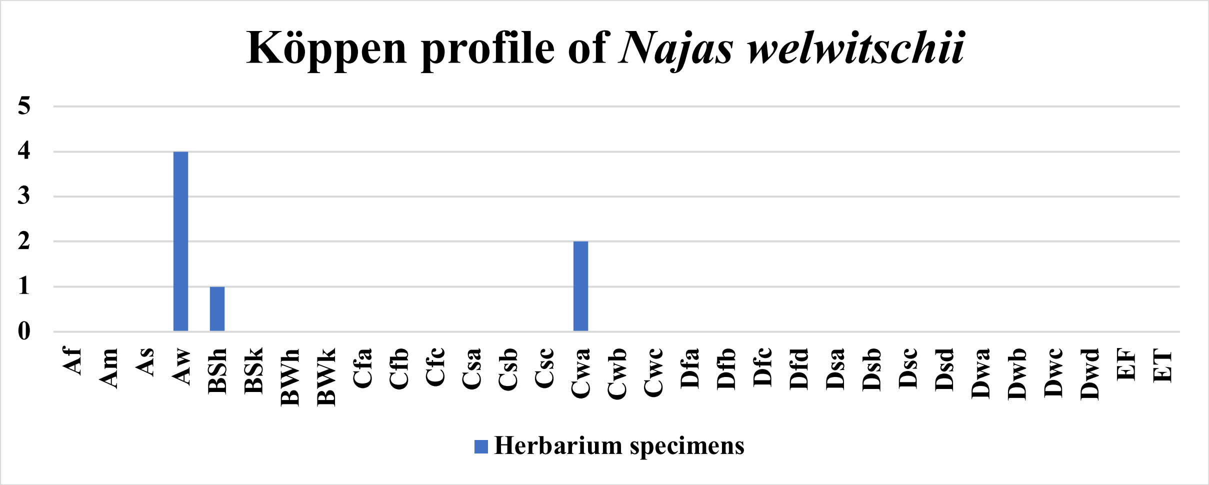

5.3.3.1.30. Köppen profile, distribution and climate map – GBIF occurrences of *Najas wrightiana* A.Braun 1868; herbarium specimens excluding duplicate occurrences (n = 55).

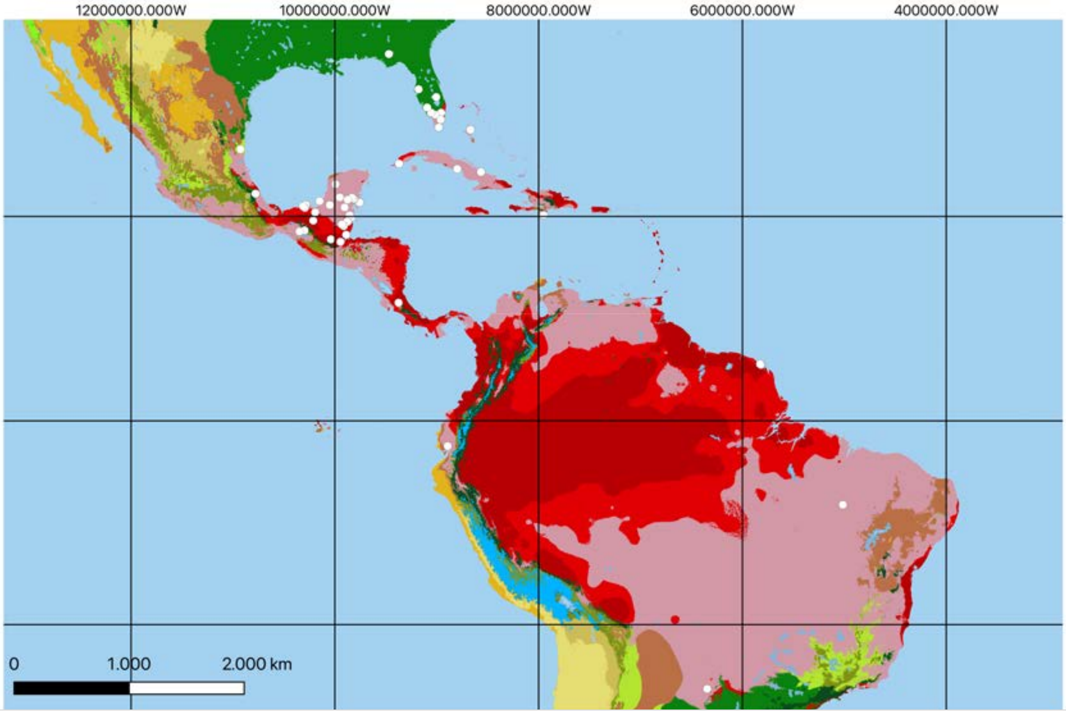

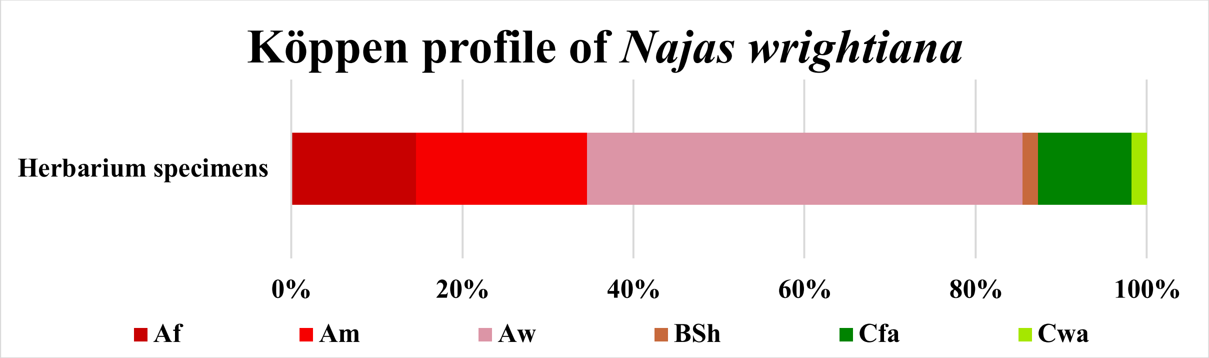

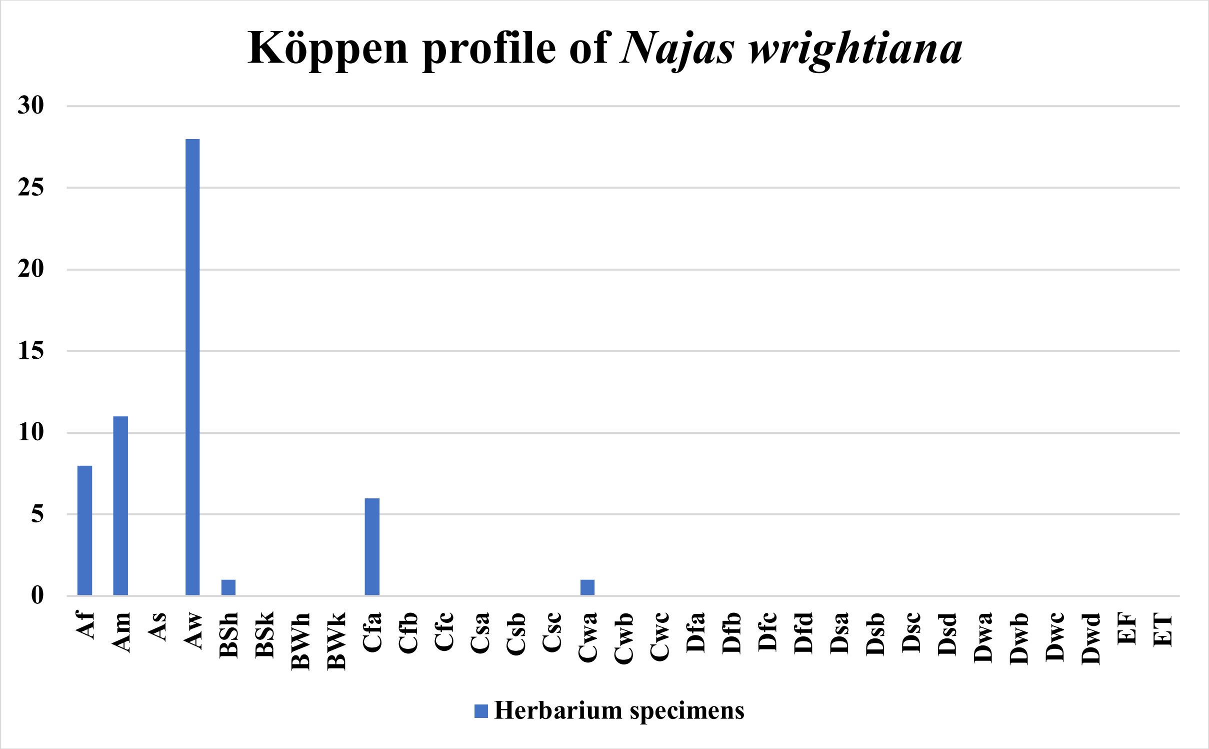

5.3.4. Crown group Clade A of Hydrocharitaceae cf. Chen et. al (2012), comprising *Hydrilla* Rich. 1811, *Nechamandra* Planch. 1849, and *Vallisneria* P.Micheli ex L. 1753

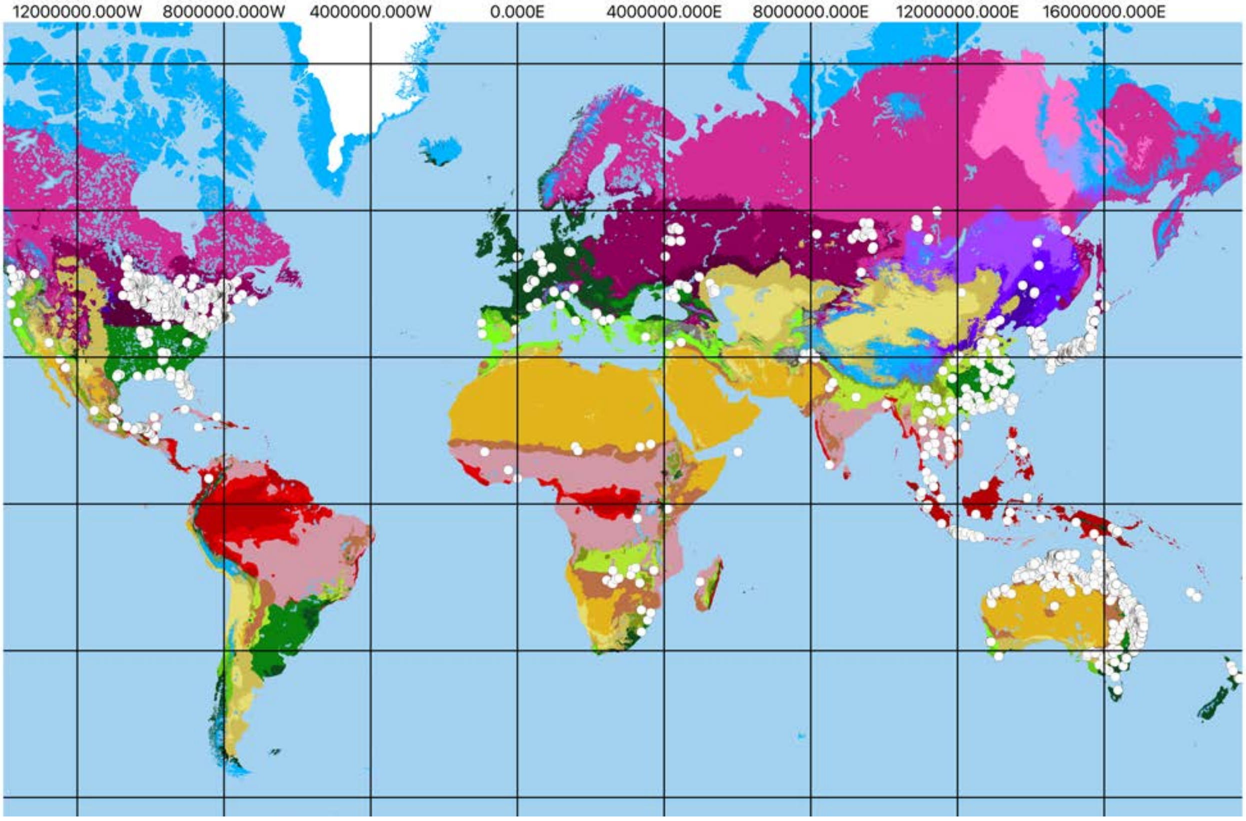

5.3.4.1. Genus *Hydrilla* Rich. 1811

5.3.4.1.1. Köppen profile, distribution and climate map – GBIF occurrences of *Hydrilla verticillate* (L.f 1782) Royle 1839; herbarium specimens excluding duplicate occurrences (n = 762).

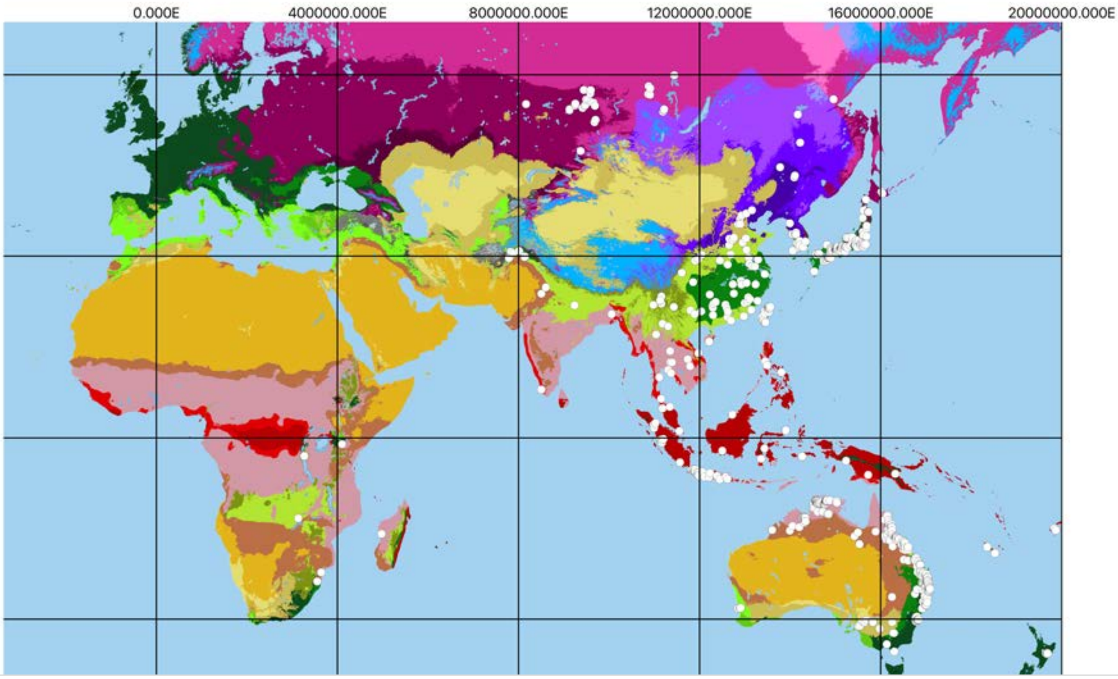

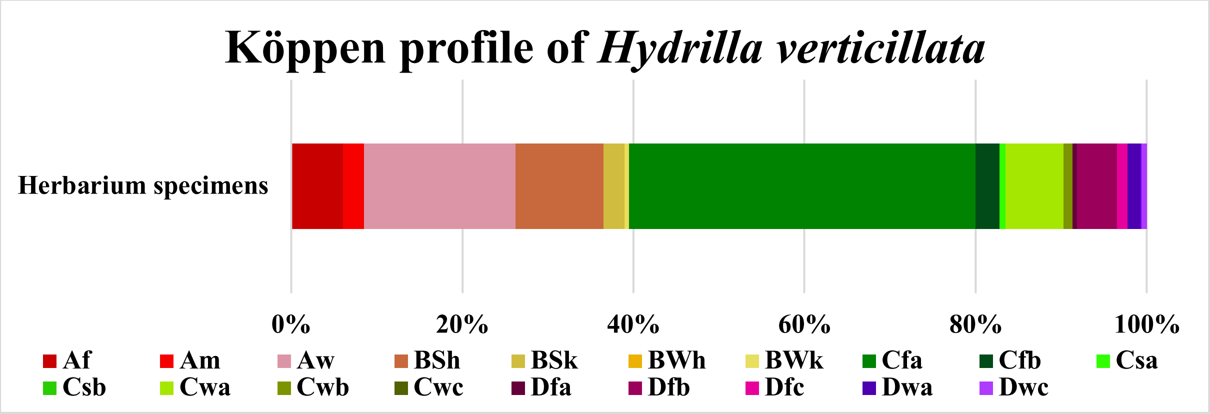

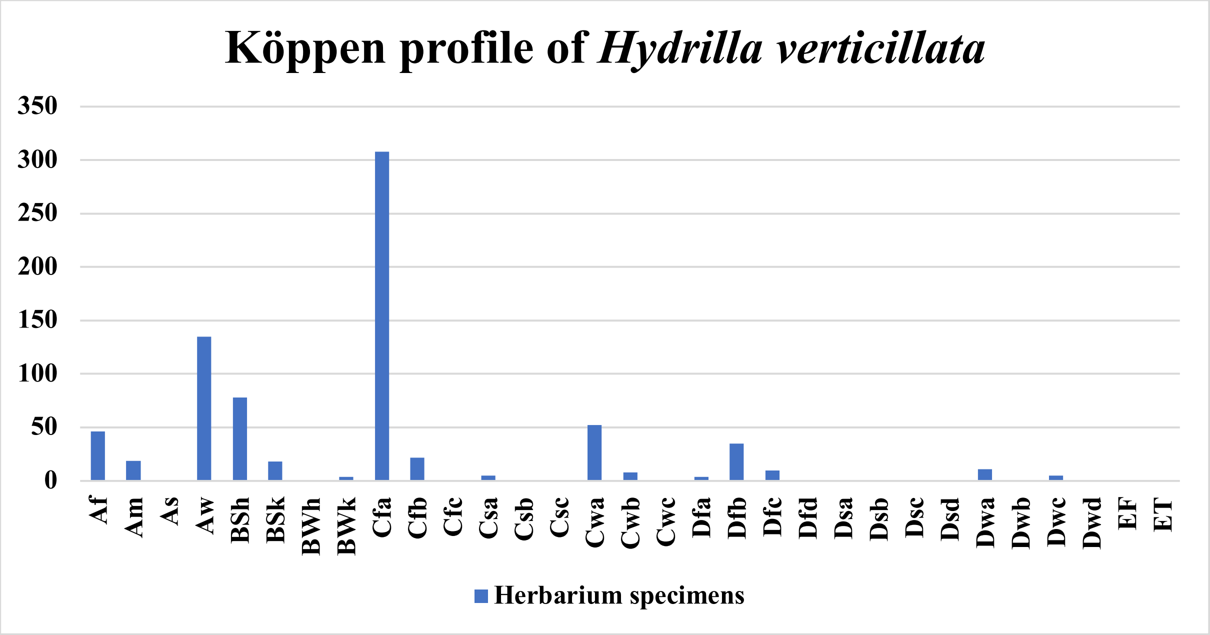

Köppen profile of *Hydrilla verticillata*
350
300
250
200
150
100
50
0
Af
Am
As
Aw
BSh
BSk
BWh
BWk
Cfa
Cfb
Cfc
Csa
Csb
Csc
Cwa
Cwb
Cwc
Dfa
Dfb
Dfc
Dfd
Dsa
Dsb
Dsc
Dsd
Dwa
Dwb
Dwc
Dwd
EF
ET
Herbarium specimens

5.3.4.2. Genus *Nechamandra* Planch. 1849.
5.3.4.2.1. Köppen profile, distribution and climate map – GBIF occurrences of *Nechamandra alternifolia* Thwaites, 1864; herbarium specimens excluding duplicate occurrences (n = 12).

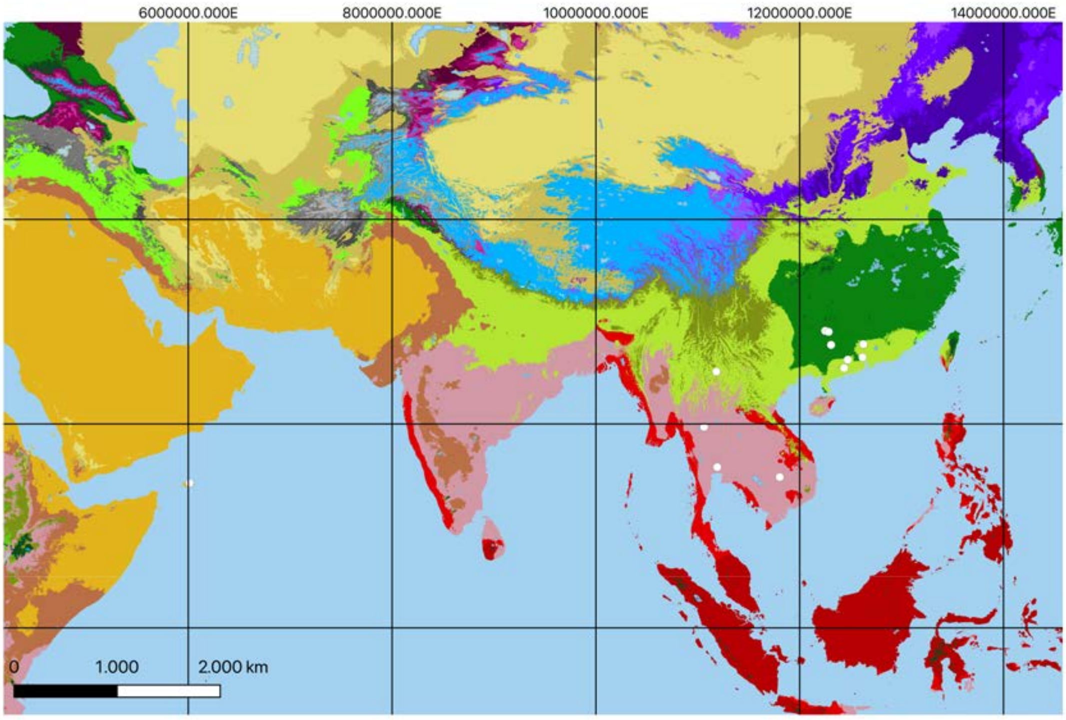

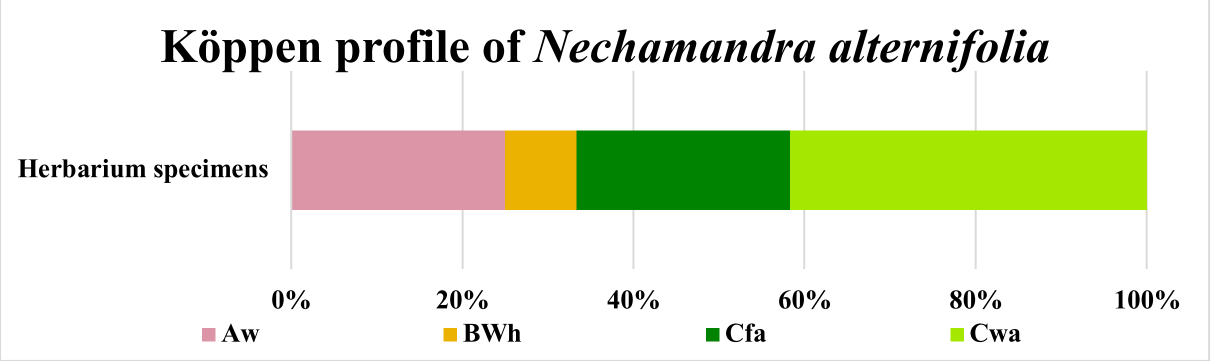

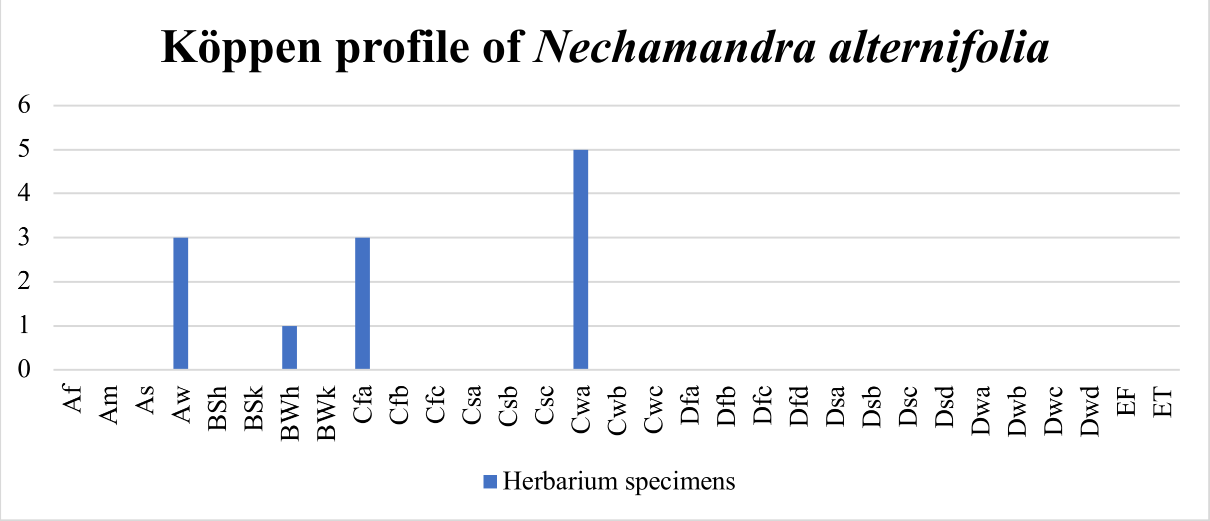

5.3.4.3. Genus *Vallisneria* Micheli ex L. 1753.
Distribution and Köppen profiles of genus *Vallisneria*, incl. *V. americana*, *V. annua*, *V. caulescens*, *V. denseserrulata*, *V. erecta*, *V. nana*, *V. natans*, *V. rubra*, *V. spiralis, V. spinulosa*, and *V. triptera*. (n = 2698).

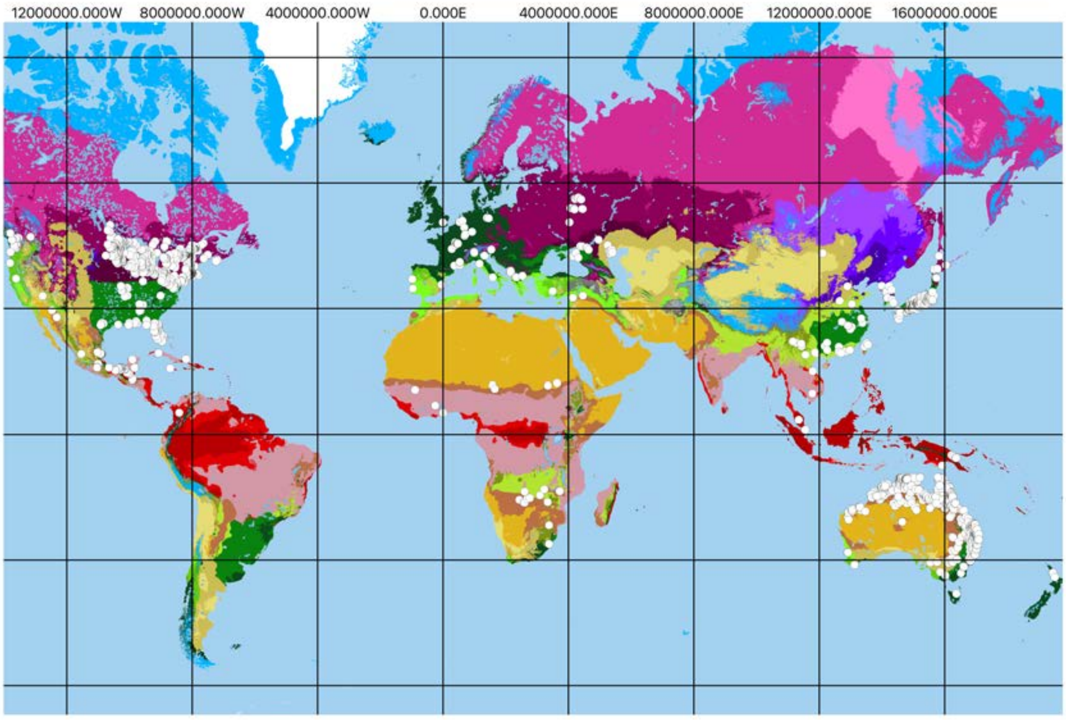

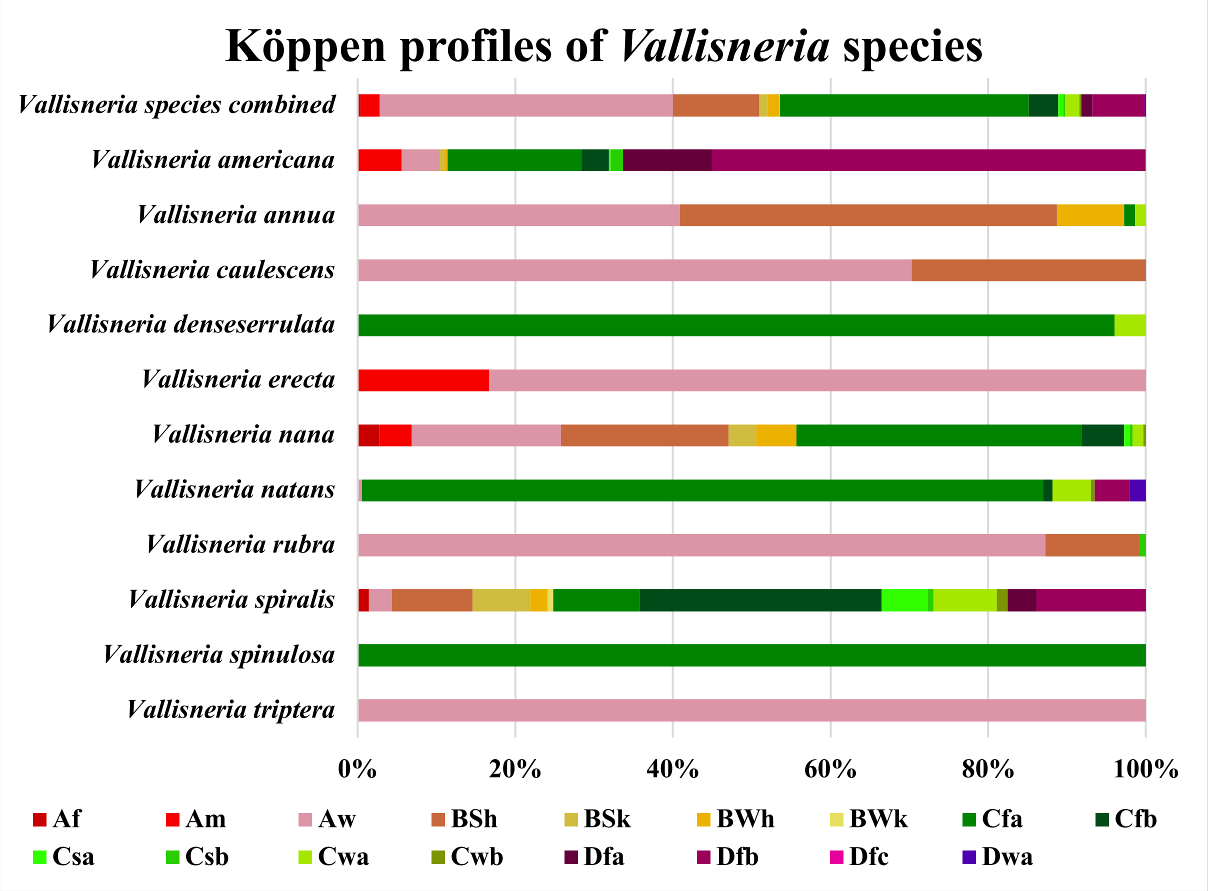

5.3.4.3.1. Köppen profile, distribution and climate map – GBIF occurrences of *Vallisneria americana* Michx. 1803; herbarium specimens excluding duplicate occurrences (n = 719).

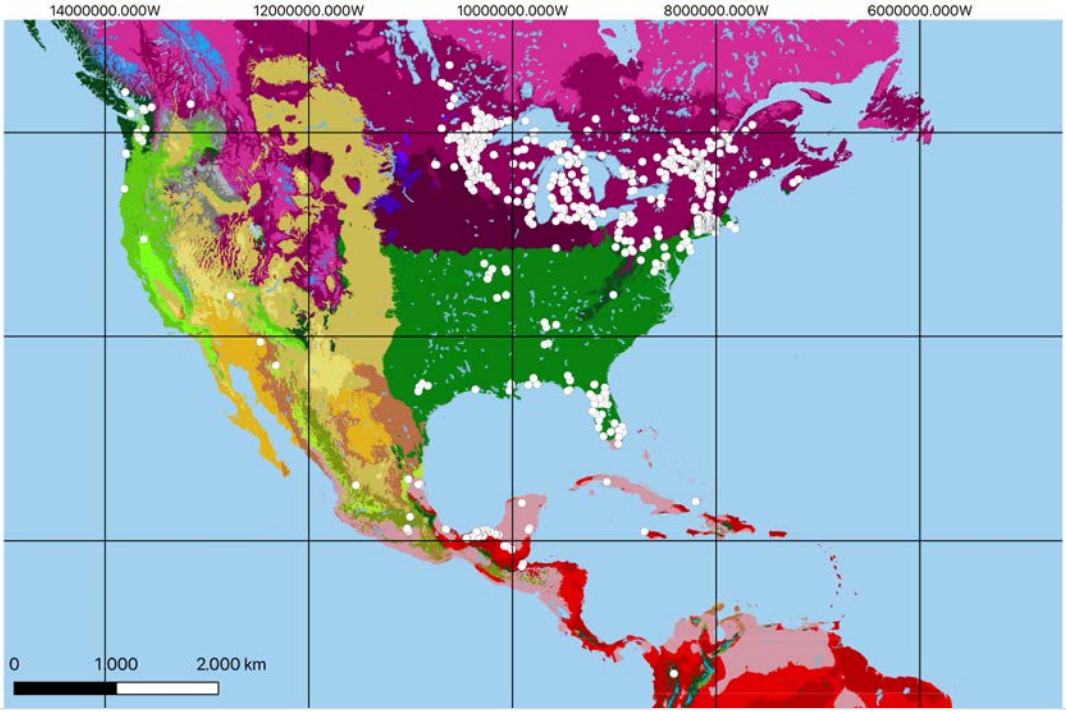

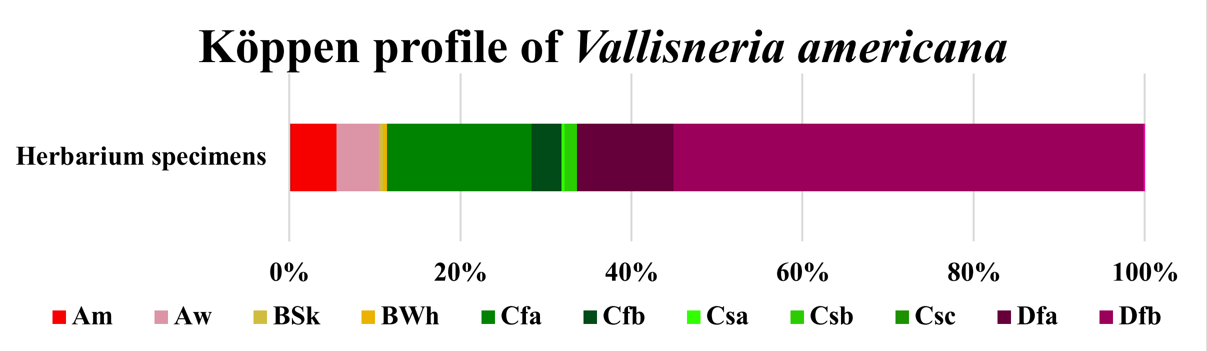

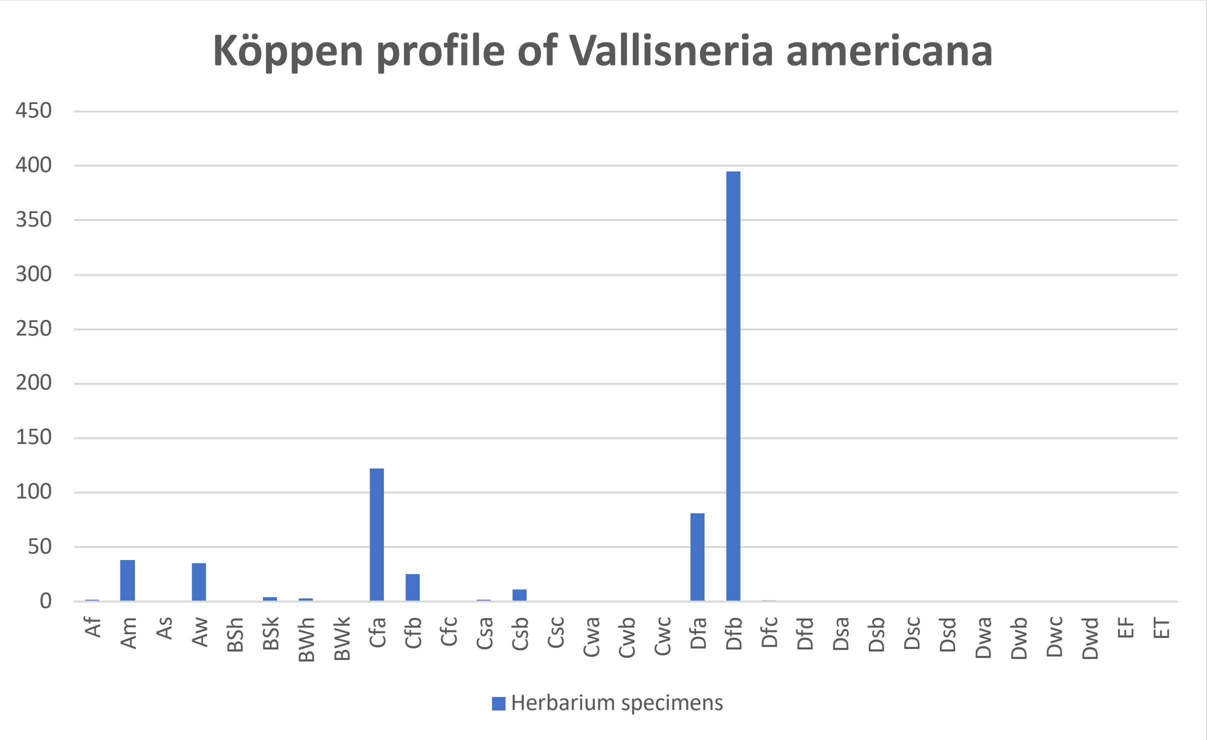

5.3.4.3.2. Köppen profile, distribution and climate map – GBIF occurrences of *Vallisneria annua* S.W.L.Jacobs & K.A.Frank 1997; herbarium specimens excluding duplicate occurrences (n = 142).

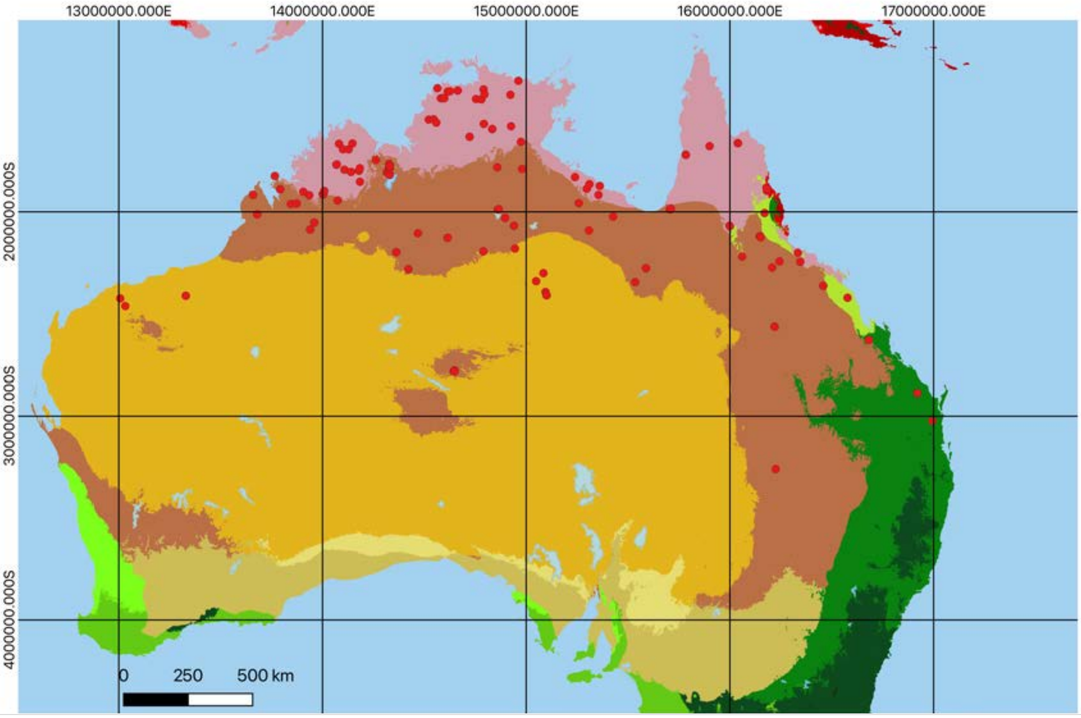

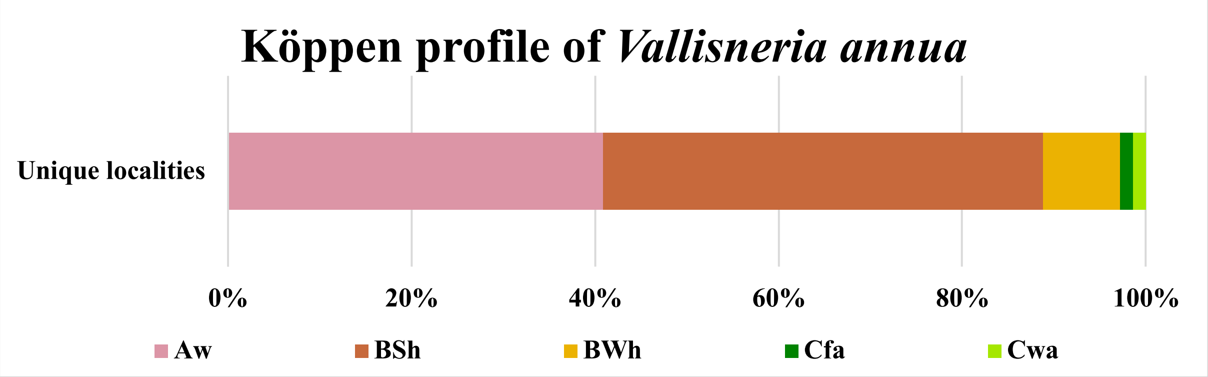

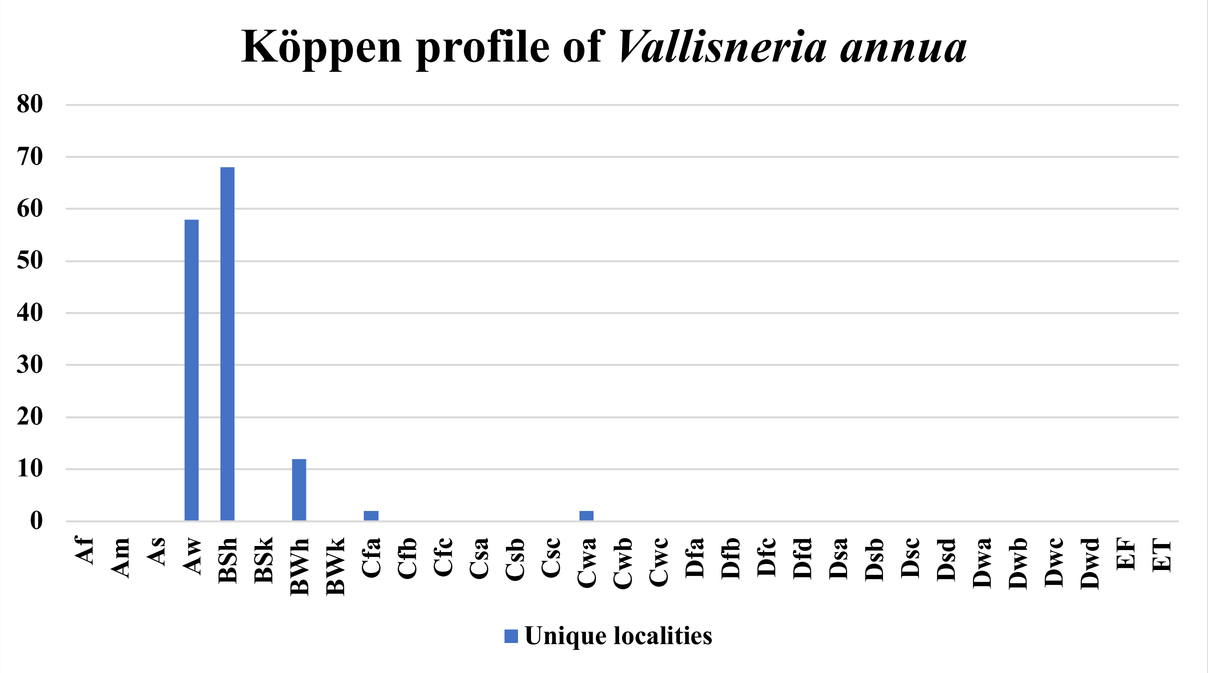

5.3.4.3.3. Köppen profile, distribution and climate map – GBIF occurrences of *Vallisneria caulescens* F.M.Bailey & F.Muell. 1888; herbarium specimens excluding duplicate occurrences (n = 101).

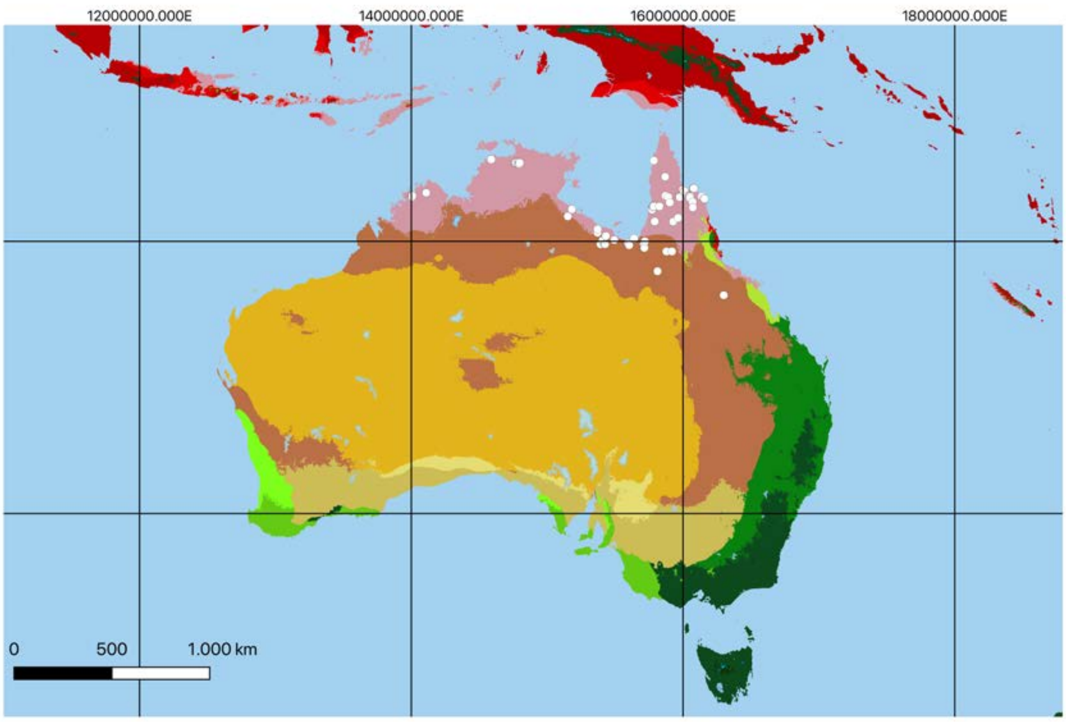

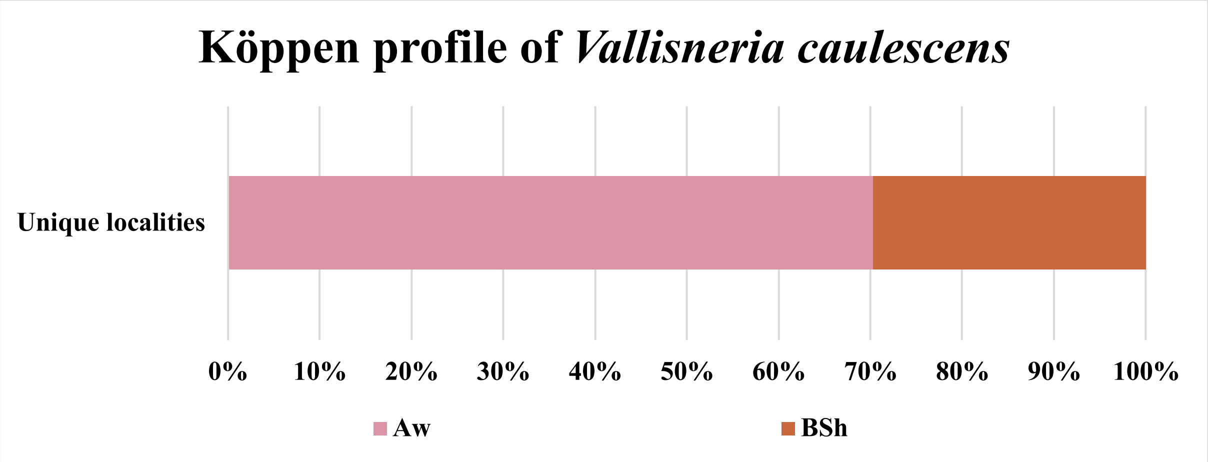

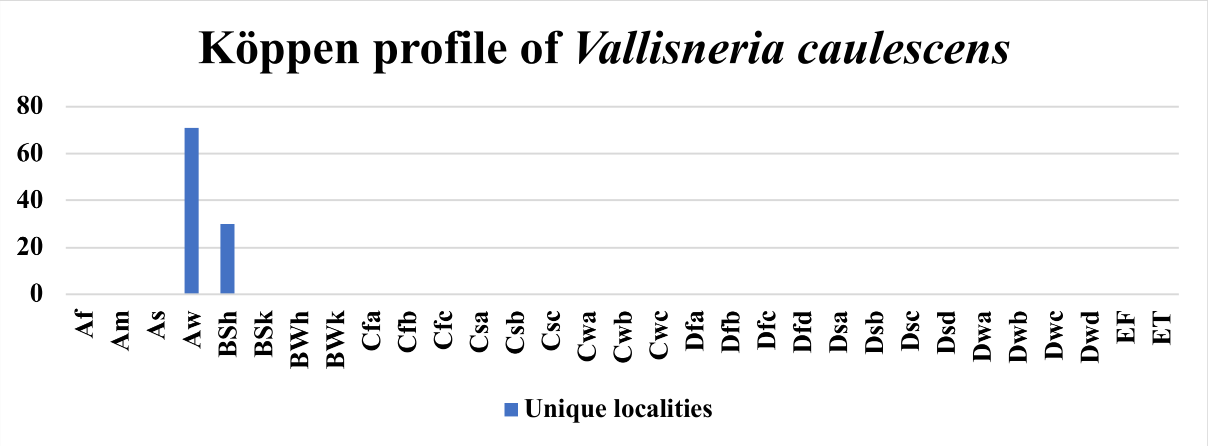

5.3.4.3.4. Köppen profile, distribution and climate map – GBIF occurrences of *Vallisneria denseserrulata* (Makino 1914) Makino 1921; herbarium specimens excluding duplicate occurrences (n = 25).

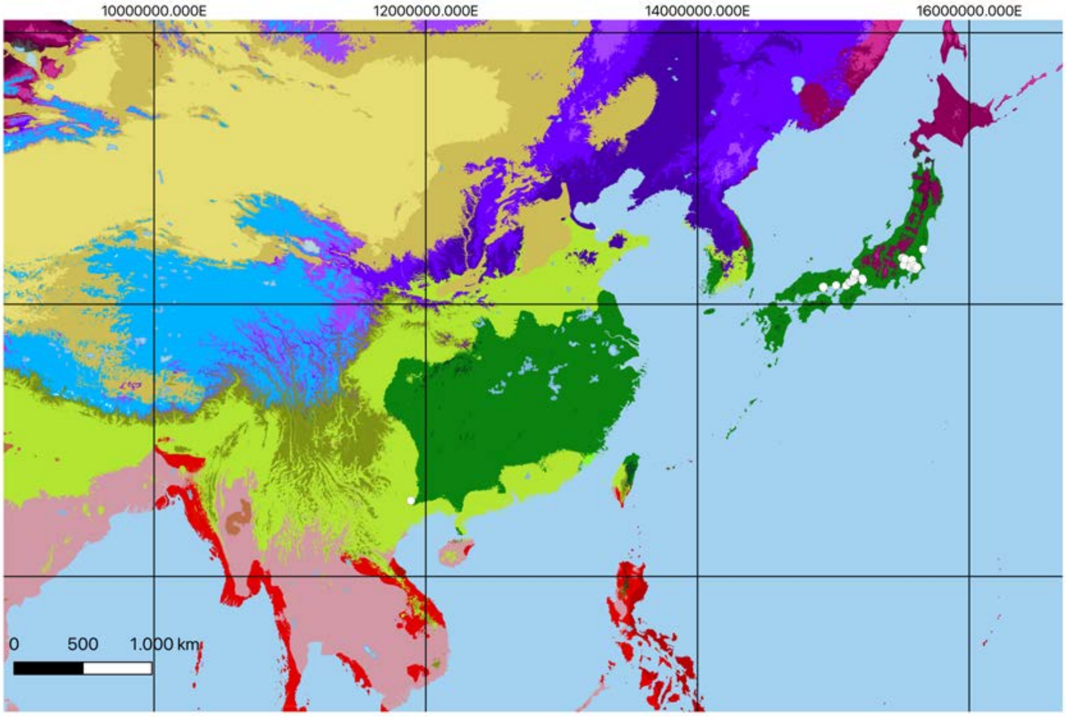

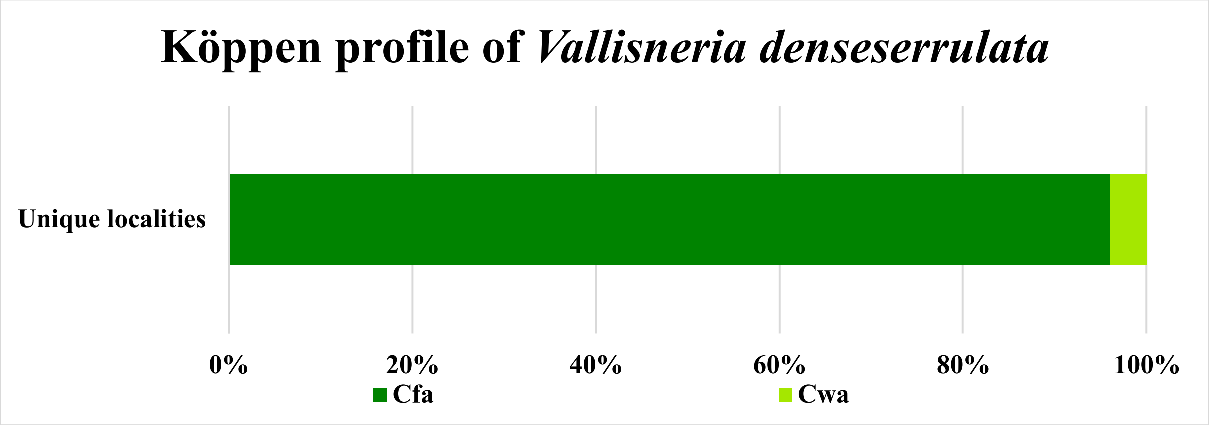

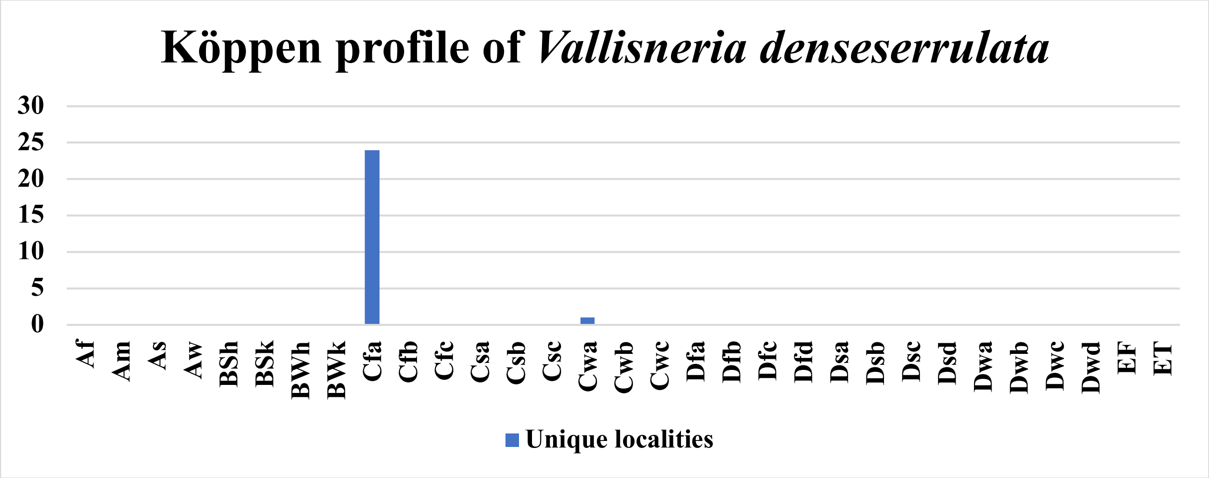

5.3.4.3.5. Köppen profile, distribution and climate map – GBIF occurrences of *Vallisneria erecta* S.W.L.Jacobs 2008; herbarium specimens excluding duplicate occurrences (n = 6).

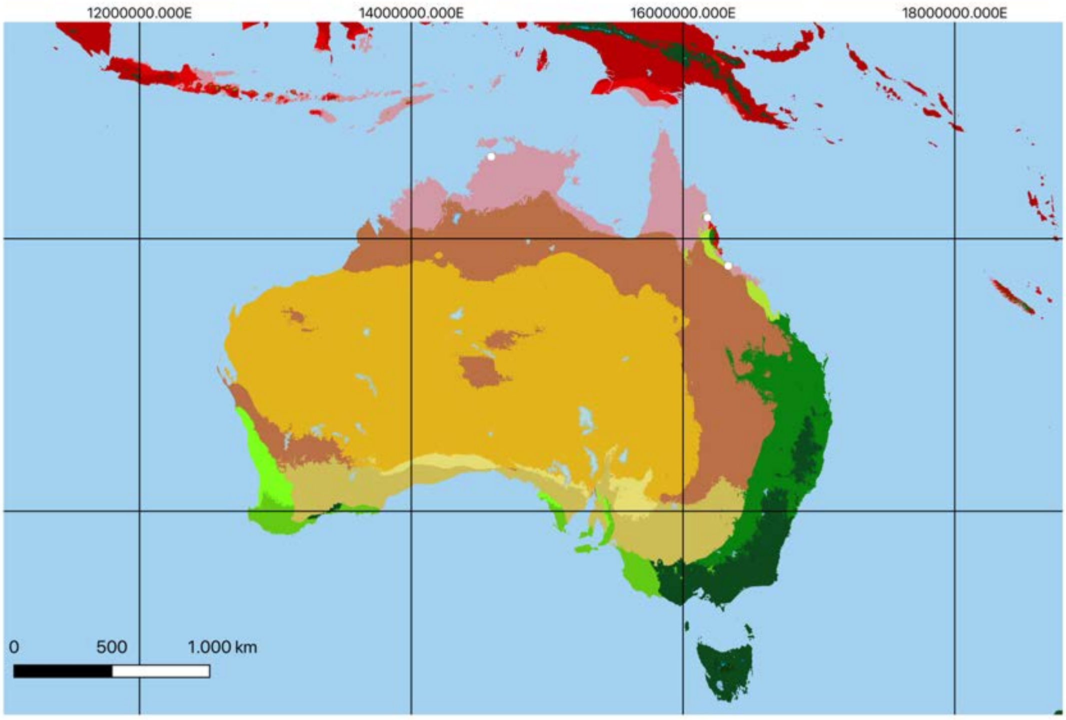

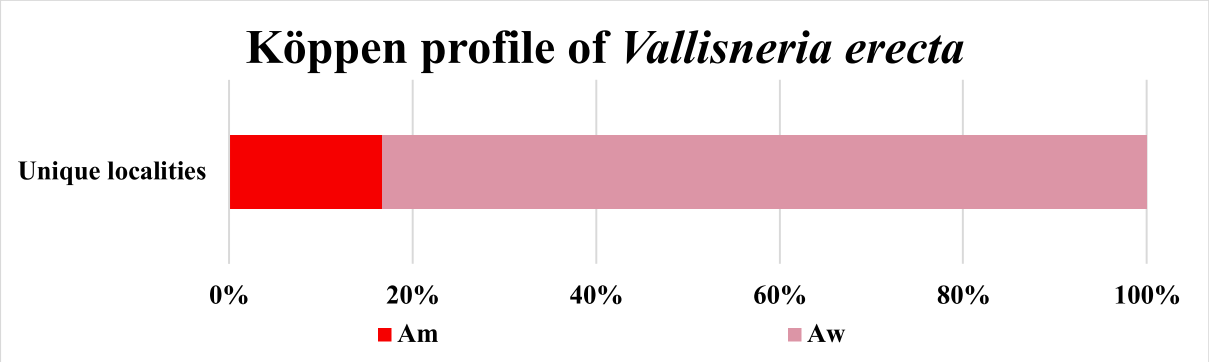

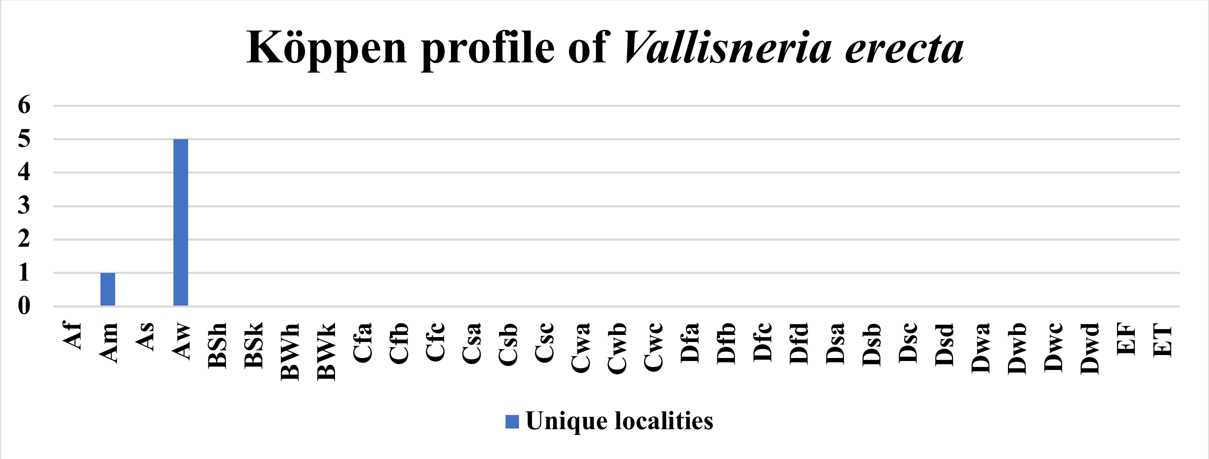

5.3.4.3.6. Köppen profile, distribution and climate map – GBIF occurrences of *Vallisneria nana* R.Br. 1810; herbarium specimens excluding duplicate occurrences (n = 291).

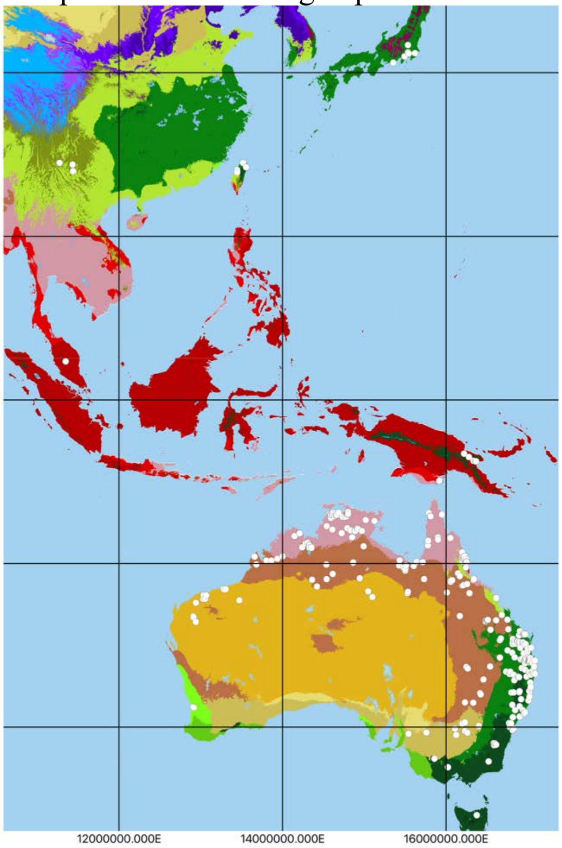

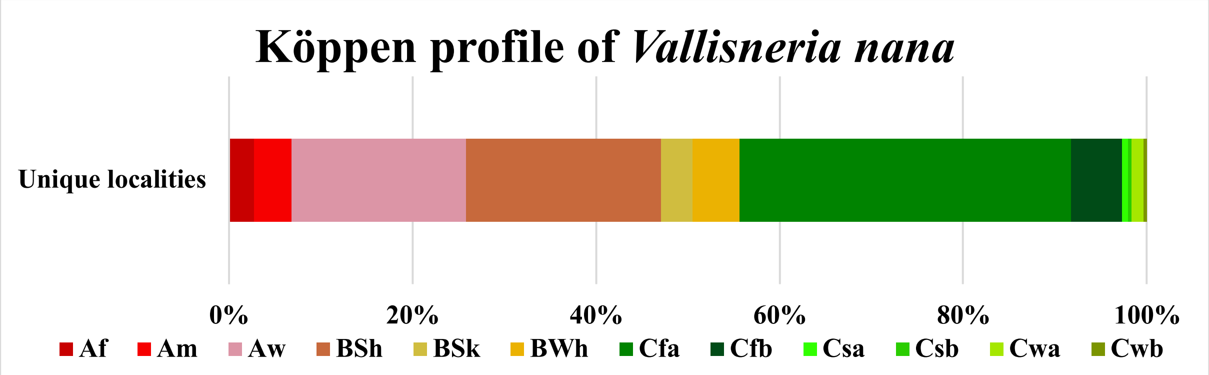

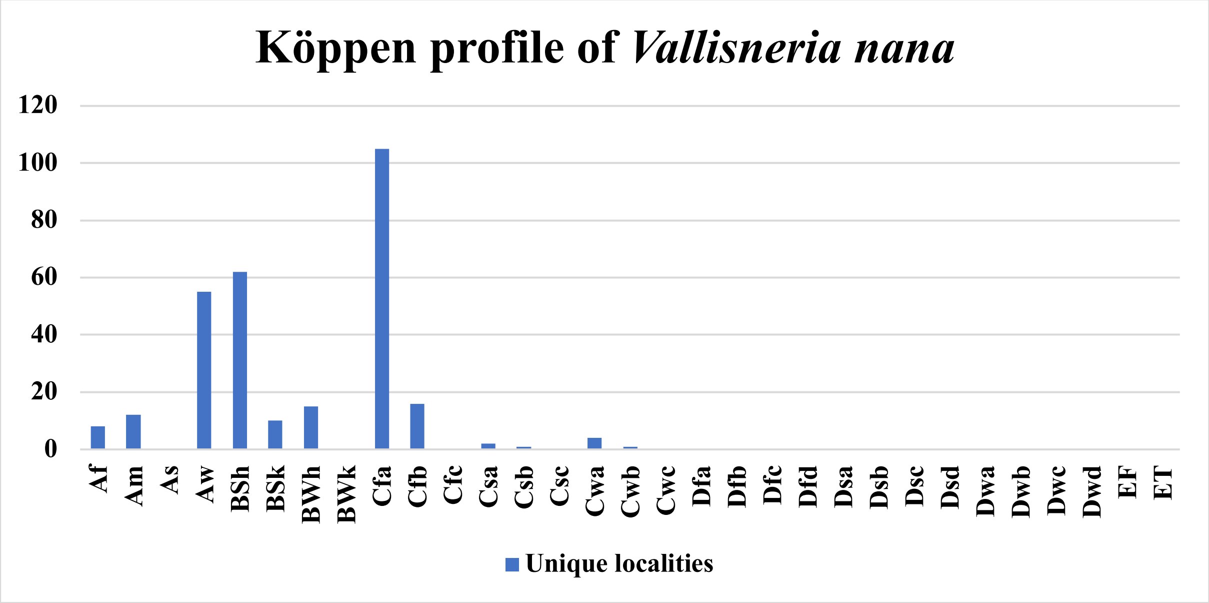

5.3.4.3.7. Köppen profile, distribution and climate map – GBIF occurrences of *Vallisneria natans* (Lour. 1790) H.Hara 1974 herbarium specimens excluding duplicate occurrences (n = 185).

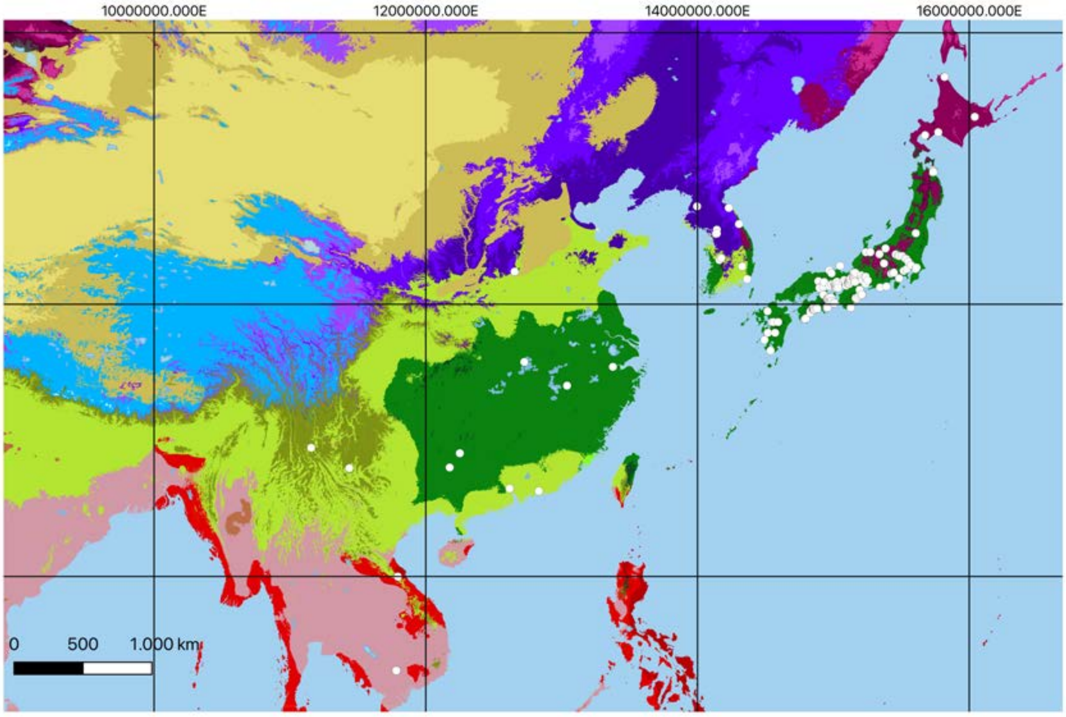

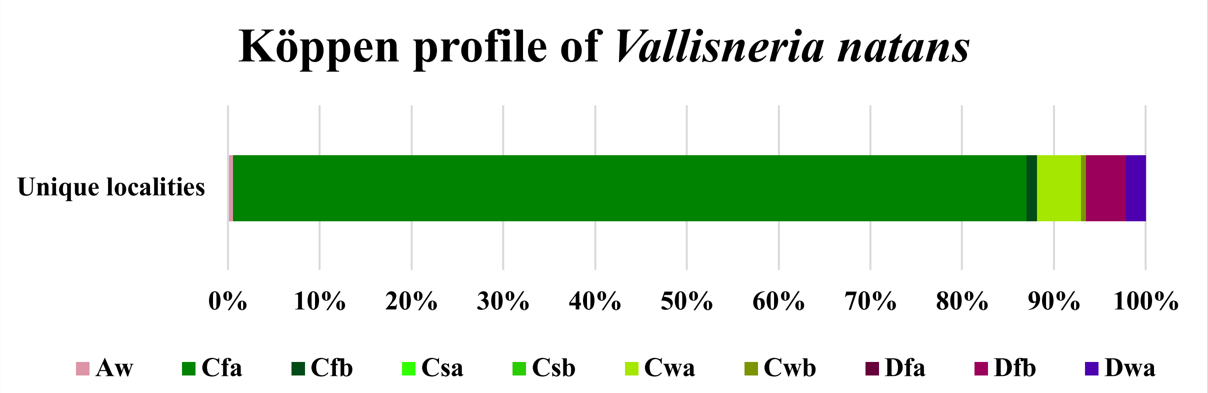

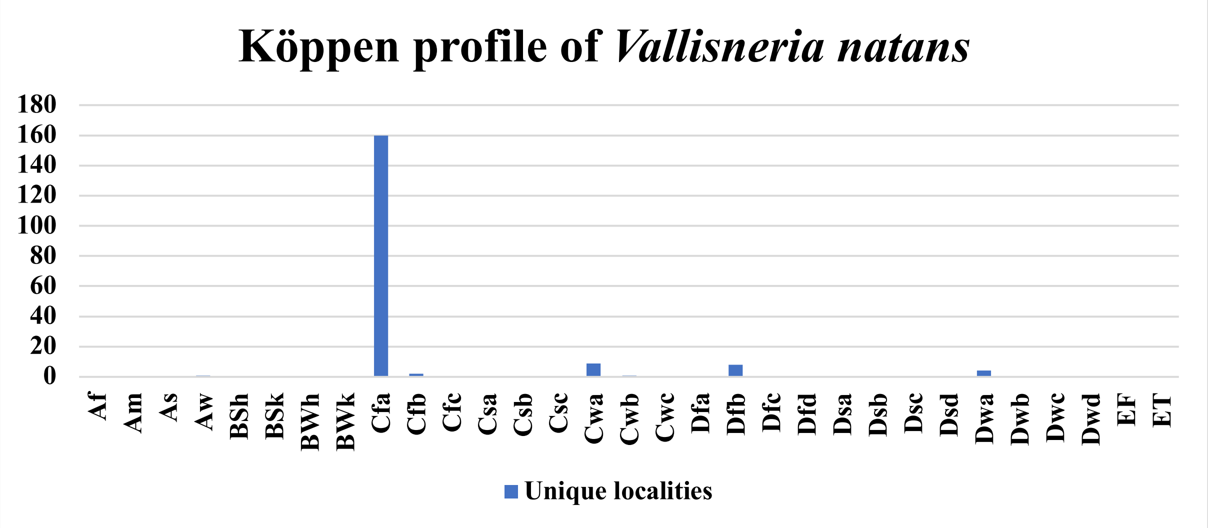

5.3.4.3.8. Köppen profile, distribution and climate map – GBIF occurrences of *Vallisneria rubra* (Rendle 1916) Les & S.W.L.Jacobs 2008; herbarium specimens excluding duplicate occurrences (n = 125).

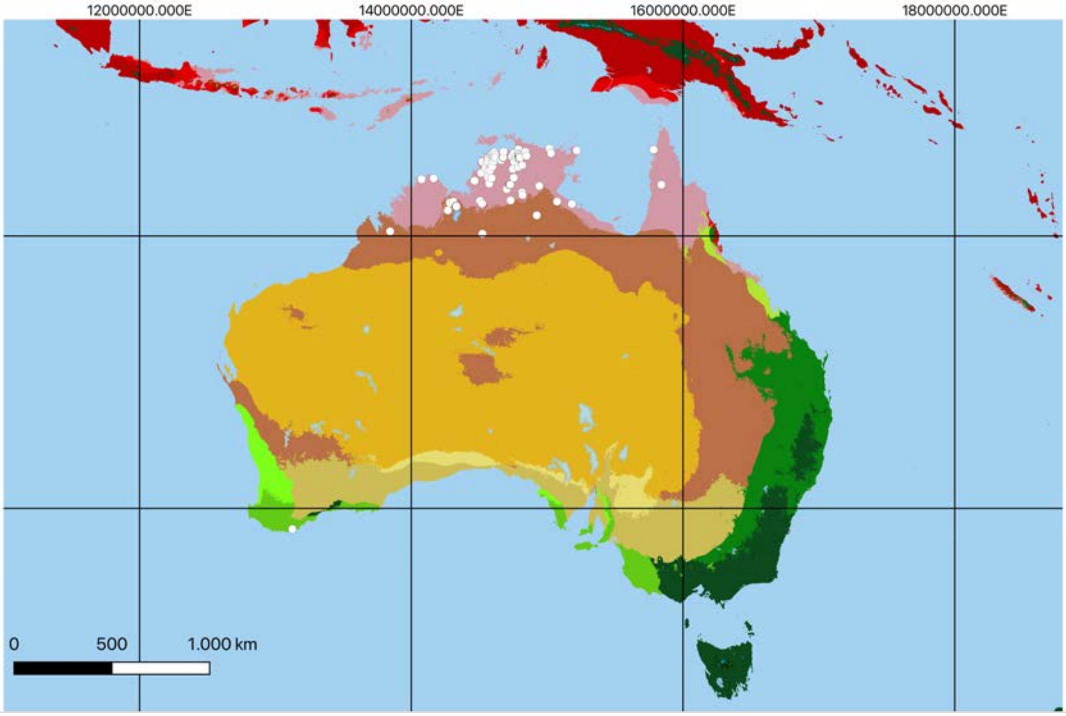

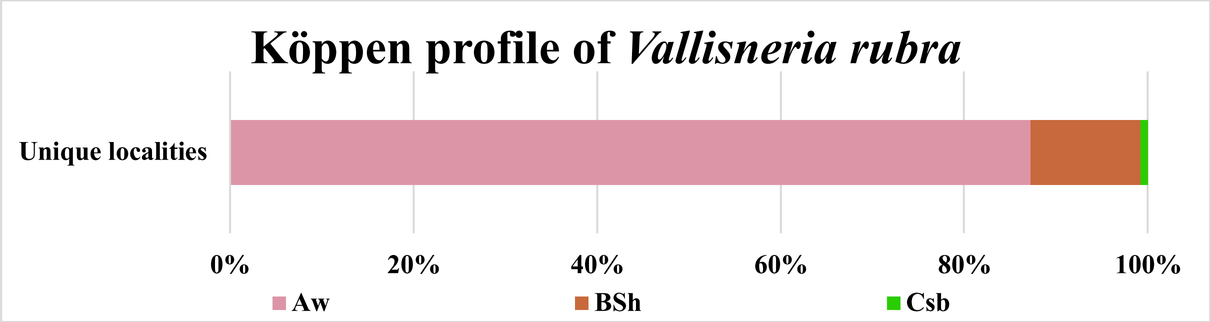

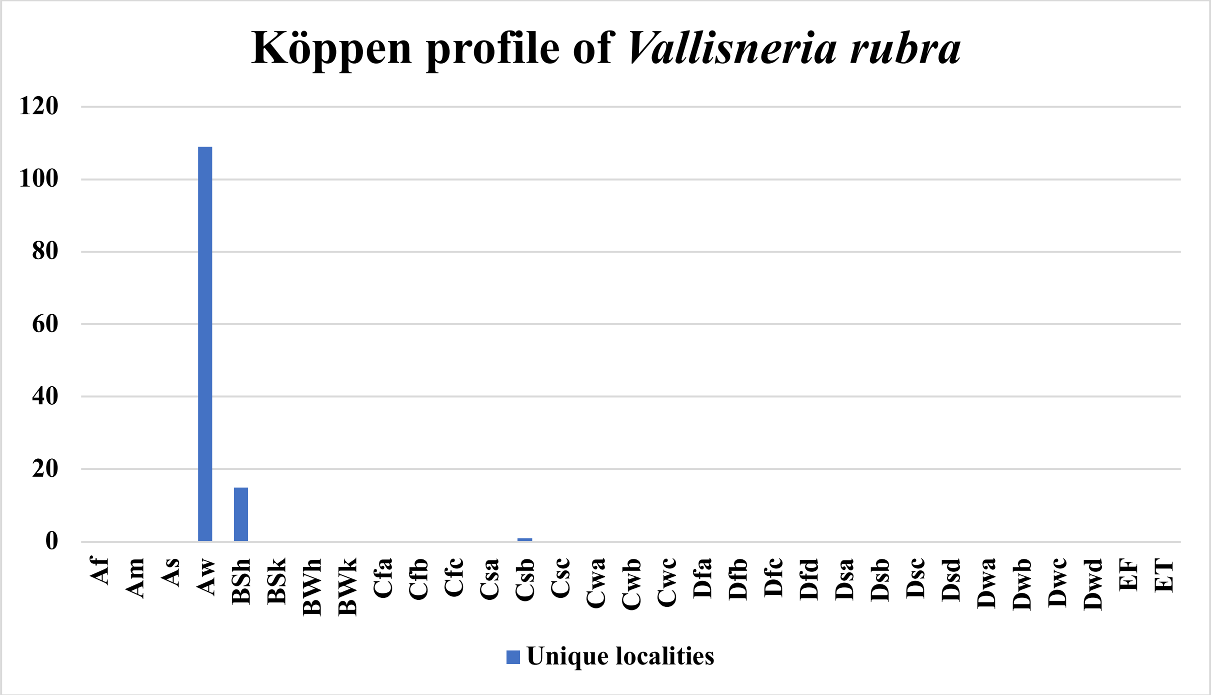

5.3.4.3.9. Köppen profile, distribution and climate map – GBIF occurrences of *Vallisneria spiralis* L. 1753; herbarium specimens excluding duplicate occurrences (n = 137).

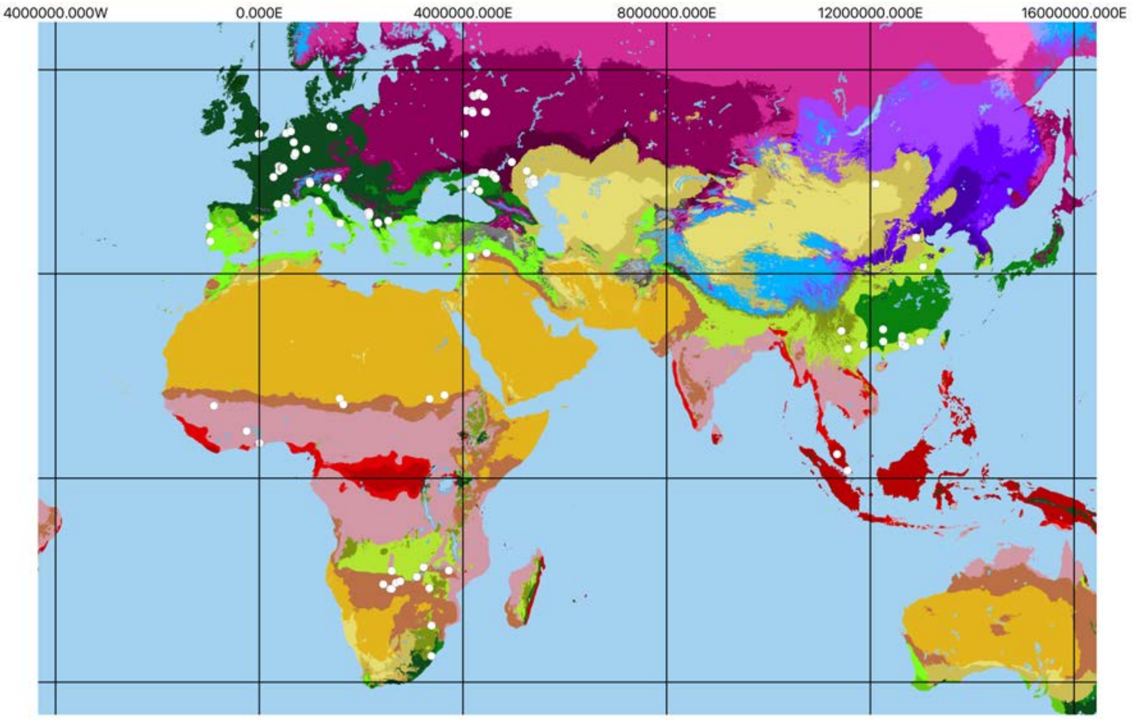

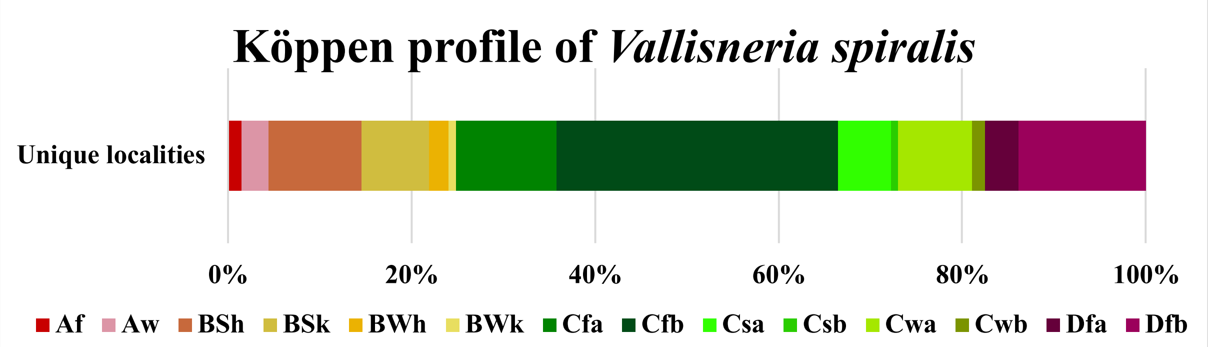

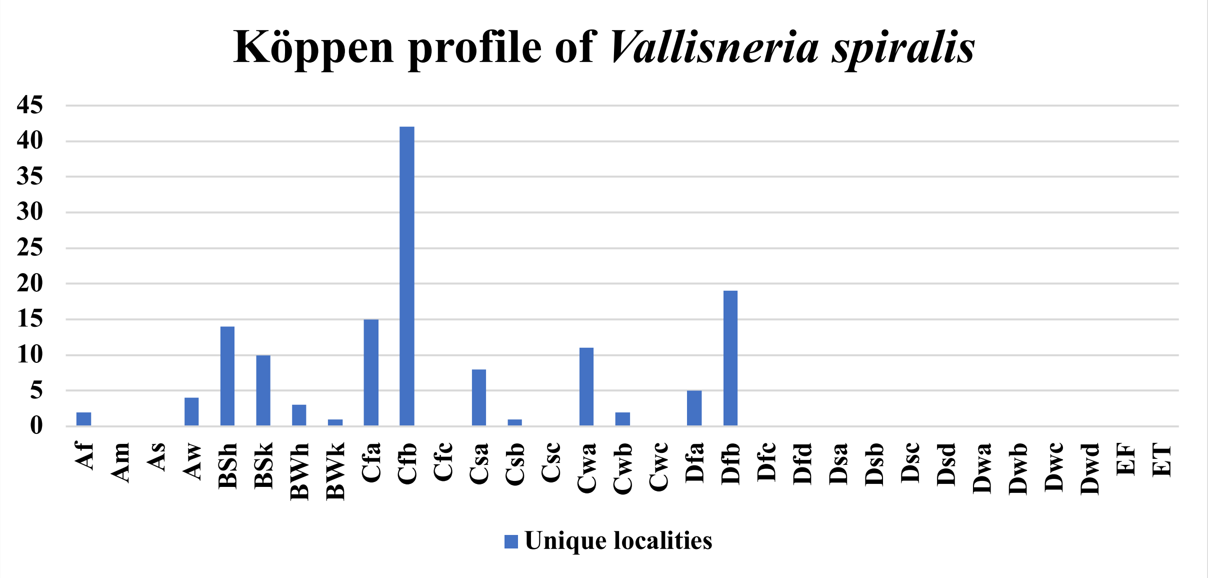

5.3.4.3.10. Köppen profile, distribution and climate map – GBIF occurrences of *Vallisneria spinulosa* S.Z.Yan 1982; herbarium specimens excluding duplicate occurrences (n = 3).

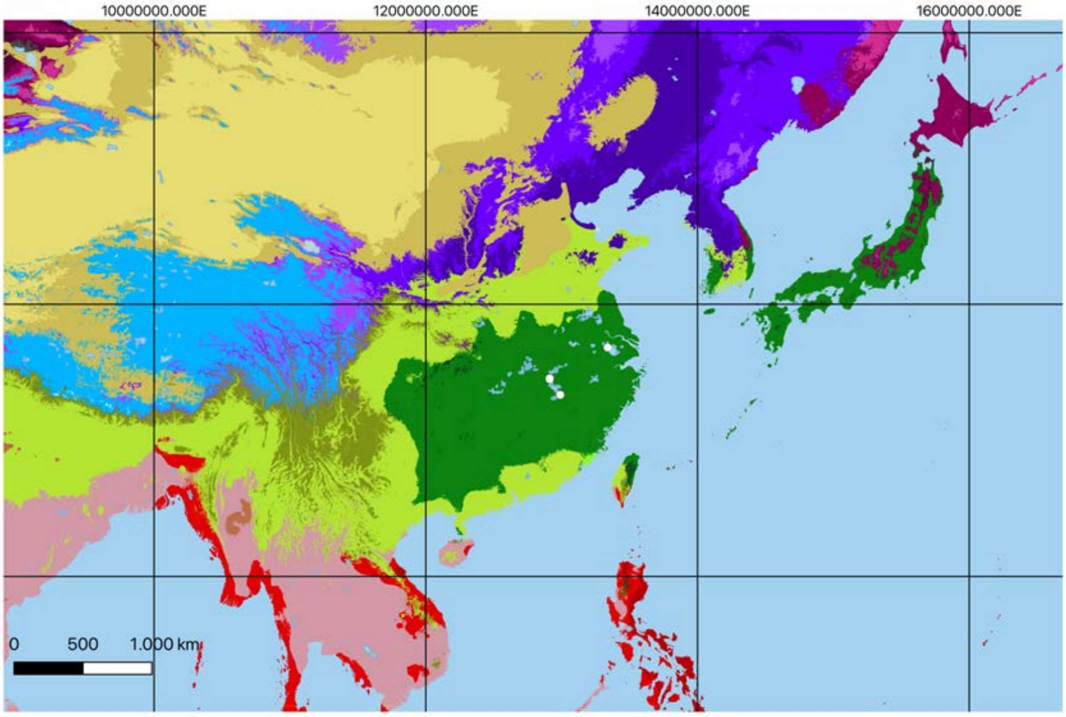

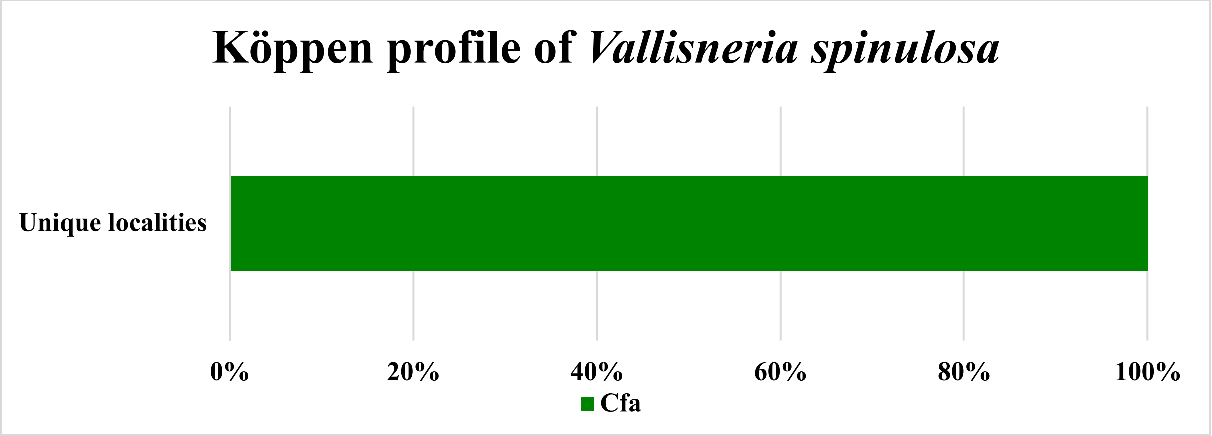

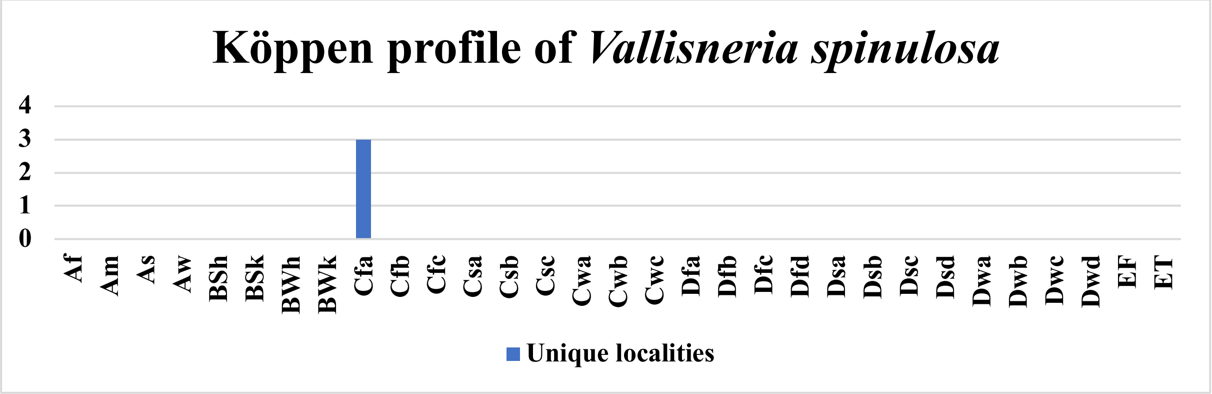

5.3.4.3.11. Köppen profile, distribution and climate map – GBIF occurrences of *Vallisneria triptera* S.W.L.Jacobs & K.A.Frank 1997; herbarium specimens excluding duplicate occurrences (n = 38).

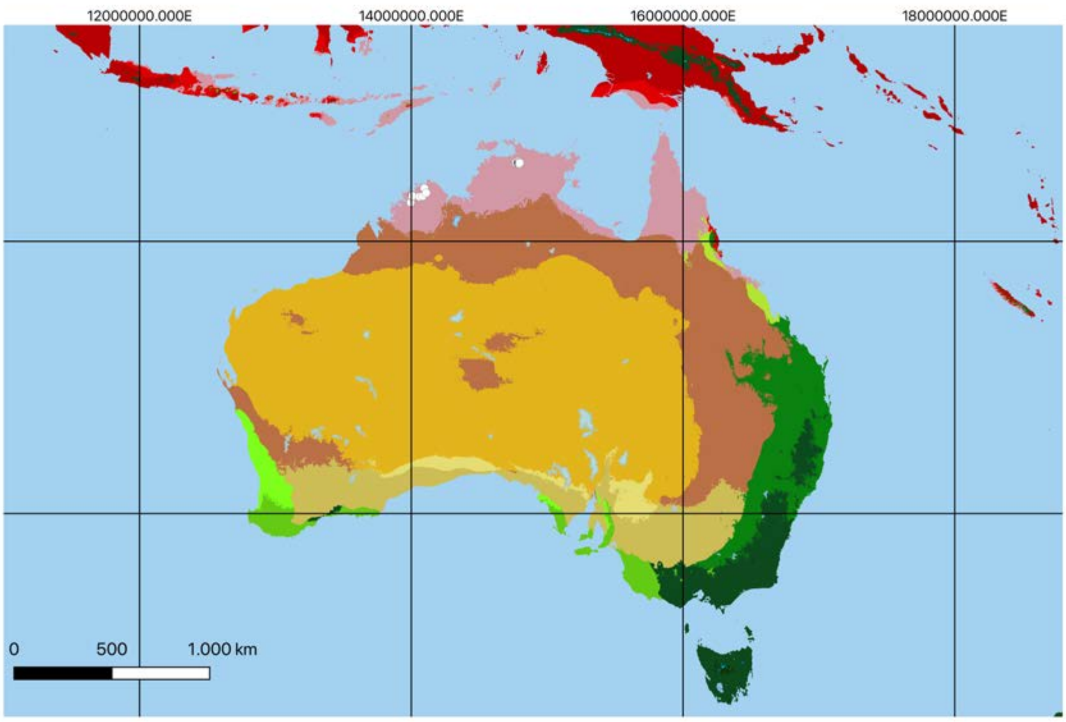

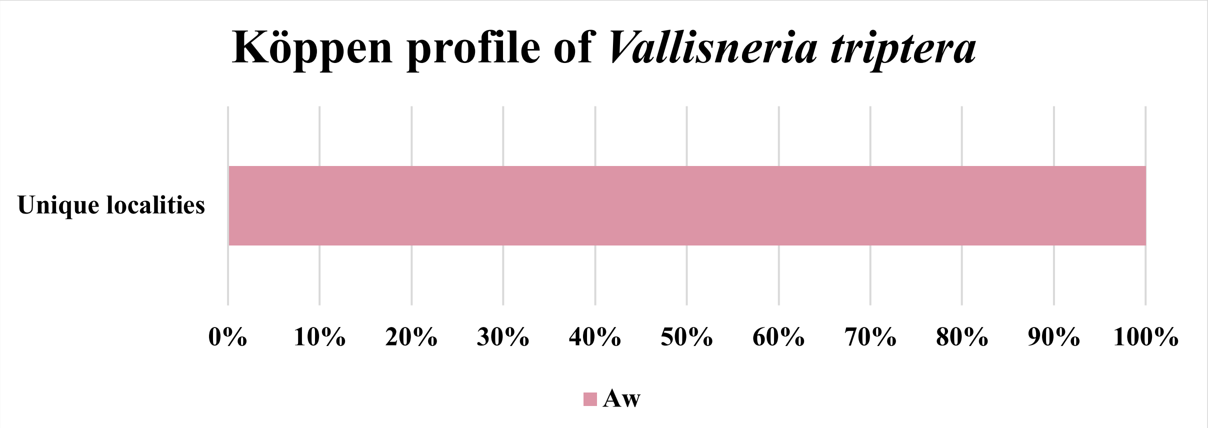

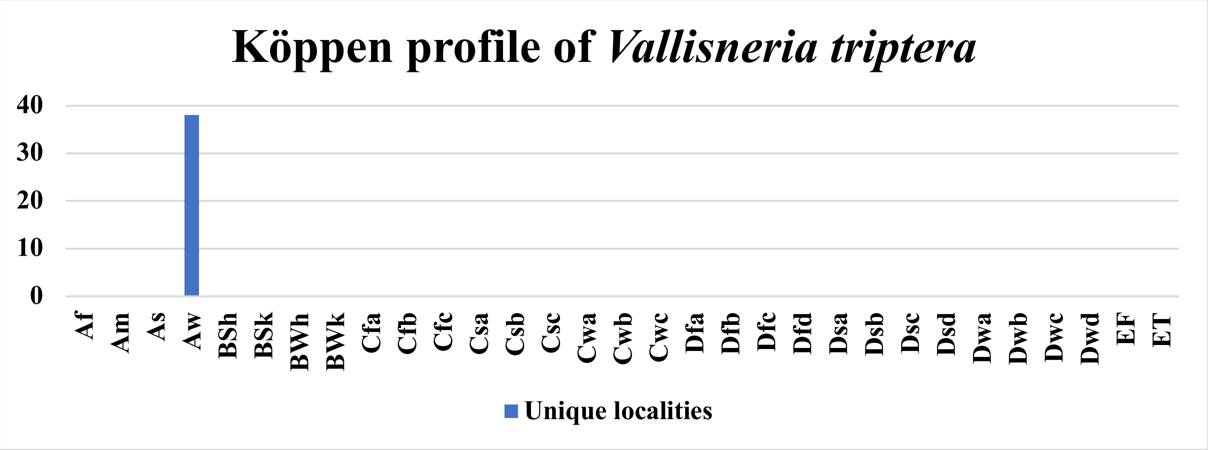

GBIF datasets:

Blyxa:
Blyxa auberti GBIF Data set: https://doi.org/10.15468/dl.3j4svh
Blyxa echinosperma GBIF Data set: https://doi.org/10.15468/dl.tz2q94
Blyxa hexandra GBIF Data set: https://doi.org/10.15468/dl.m74dkr
Blyxa japonica GBIF Data set: https://doi.org/10.15468/dl.qa5fwa
Blyxa octandra GBIF Data set: https://doi.org/10.15468/dl.4cn2yg
Blyxa senegalensis GBIF Data set: https://doi.org/10.15468/dl.tju7pg
Blyxa shimadai GBIF Data set: https://doi.org/10.15468/dl.gt7uv3
Blyxa somai GBIF Data set: https://doi.org/10.15468/dl.7ejqk9

Egeria:
Egeria densa GBIF Data set: https://doi.org/10.15468/dl.ukwfx3
Egeria heterostemon GBIF Data set: https://doi.org/10.15468/dl.hasy2z
Egeria najas GBIF Data set: https://doi.org/10.15468/dl.9h5hst

Elodea:
Elodea bifoliata GBIF Data set: https://doi.org/10.15468/dl.hhnwbb
Elodea callitrichoides GBIF Data set: https://doi.org/10.15468/dl.neghzn
Elodea canadensis GBIF Data set: https://doi.org/10.15468/dl.3nr47e
Elodea granatensis GBIF Data set: https://doi.org/10.15468/dl.7t6hms
Elodea nuttallii GBIF Data set: https://doi.org/10.15468/dl.7t79ef
Elodea potamogeton GBIF Data set: https://doi.org/10.15468/dl.gacw7j

Enhalus:
Enhalus bifoliata GBIF Data set: https://doi.org/10.15468/dl.7m27pd

Halophila:
Halophila australis GBIF Data set: https://doi.org/10.15468/dl.g6maep
Halophila baillonis GBIF Data set: https://doi.org/10.15468/dl.6uswb5
Halophila beccari GBIF Data set: https://doi.org/10.15468/dl.9bk8sz
Halophila capricorni GBIF Data set: https://doi.org/10.15468/dl.4ug7ez
Halophila decipiens GBIF Data set: https://doi.org/10.15468/dl.4z58jg
Halophila engelmannii GBIF Data set: https://doi.org/10.15468/dl.sv6uhx
Halophila gaudichaudii GBIF Data set: https://doi.org/10.15468/dl.9zx4bm
Halophila hawaiiana GBIF Data set: https://doi.org/10.15468/dl.ycjvn6
Halophila major GBIF Data set: https://doi.org/10.15468/dl.jpu9t2
Halophila minor GBIF Data set: https://doi.org/10.15468/dl.rn36mp
Halophila nipponica GBIF Data set: https://doi.org/10.15468/dl.ssxf8d
Halophila ovalis GBIF Data set: https://doi.org/10.15468/dl.9kye3d
Halophila spinulosa GBIF Data set: https://doi.org/10.15468/dl.e8gzfx
Halophila stipulaceae GBIF Data set: https://doi.org/10.15468/dl.ekfzps
Halophila sulawesii GBIF Data set: https://doi.org/10.15468/dl.wzyxq6
Halophila tricostata GBIF Data set: https://doi.org/10.15468/dl.he7ef8

Hydrilla:
Hydrilla verticillata GBIF Data set: https://doi.org/10.15468/dl.fq8rxg

Hydrocharis:
Hydrocharis chevalieri GBIF Data set: https://doi.org/10.15468/dl.rvpz6w
Hydrocharis dubia GBIF Data set: https://doi.org/10.15468/dl.r8nnx4
Hydrocharis morsus-ranae GBIF Data set: https://doi.org/10.15468/dl.naq388
Hydrocharis laevigatum GBIF Data set: https://doi.org/10.15468/dl.59kdvq
Hydrocharis spongia GBIF Data set: https://doi.org/10.15468/dl.rwq9fy

Hydromystria:
Hydromystria stolonifera GBIF Data set: https://doi.org/10.15468/dl.jpuj77

Lagarosiphon:
Lagarosiphon cordofanus GBIF Data set: https://doi.org/10.15468/dl.kvunr9
Lagarosiphon hydrilloides GBIF Data set: https://doi.org/10.15468/dl.6hmd2f
Lagarosiphon ilicifolius GBIF Data set: https://doi.org/10.15468/dl.ff3xzb
Lagarosiphon madagascariensis GBIF Data set: https://doi.org/10.15468/dl.8fmajb
Lagarosiphon major GBIF Data set: https://doi.org/10.15468/dl.2p5pe5
Lagarosiphon muscoides GBIF Data set: https://doi.org/10.15468/dl.yk5qmf
Lagarosiphon rubellus GBIF Data set: https://doi.org/10.15468/dl.j9n25k
Lagarosiphon steudneri GBIF Data set: https://doi.org/10.15468/dl.ry68r9
Lagarosiphon verticillifolius GBIF Data set: https://doi.org/10.15468/dl.hmndzn

Najas:
Najas affinis GBIF data set: https://doi.org/10.15468/dl.t26may
Najas ancistrocarpa GBIF data set: https://doi.org/10.15468/dl.6rxsax
Najas arguta GBIF data set: https://doi.org/10.15468/dl.c85mtz
Najas australis GBIF data set: https://doi.org/10.15468/dl.sd69ng
Najas baldwinia GBIF data set: https://doi.org/10.15468/dl.ejhxz2
Najas browniana GBIF data set: https://doi.org/10.15468/dl.5a374t
Najas chinensis GBIF data set: https://doi.org/10.15468/dl.kg84zf
Najas conferta GBIF data set: https://doi.org/10.15468/dl.ya2xhn
Najas filifolia GBIF data set: https://doi.org/10.15468/dl.a5h9cz
Najas flexilis GBIF data set: https://doi.org/10.15468/dl.wtfxmf
Najas gracillima GBIF data set: https://doi.org/10.15468/dl.kfnjf9
Najas graminea GBIF data set: https://doi.org/10.15468/dl.ckrhqm
Najas guadalupensis GBIF data set: https://doi.org/10.15468/dl.v9ncbf
Najas hagerupi GBIF data set: https://doi.org/10.15468/dl.5449n3
Najas halophila GBIF data set: https://doi.org/10.15468/dl.vbrmdz
Najas horrida GBIF data set: https://doi.org/10.15468/dl.gudekj
Najas indica GBIF data set: https://doi.org/10.15468/dl.5dkpwa
Najas kurziana GBIF data set: https://doi.org/10.15468/dl.6xpwq9
Najas madagascariensis GBIF data set: https://doi.org/10.15468/dl.qukumw
Najas malesiana GBIF data set: https://doi.org/10.15468/dl.a6npgw
Najas marina GBIF data set: https://doi.org/10.15468/dl.56v9xt
Najas oguraensis GBIF data set: https://doi.org/10.15468/dl.3r3wnd
Najas pectinata GBIF data set: https://doi.org/10.15468/dl.xgwnfj
Najas schweinfurthii GBIF data set: https://doi.org/10.15468/dl.eckvda
Najas tenuicaulis GBIF data set: https://doi.org/10.15468/dl.2rsn6q
Najas tenuifolia GBIF data set: https://doi.org/10.15468/dl.j6vqgj
Najas tenuis GBIF data set: https://doi.org/10.15468/dl.wsdw8n
Najas tenuissima GBIF data set: https://doi.org/10.15468/dl.xepmw8
Najas testui GBIF data set: https://doi.org/10.15468/dl.yufchs

Najas welwitschii GBIF data set: https://doi.org/10.15468/dl.pqb8za
Najas wrightiana GBIF data set: https://doi.org/10.15468/dl.388e94

Nechamandra:
Nechamandra alternifolia GBIF data set: https://doi.org/10.15468/dl.f8c75z

Ottelia:
Ottelia acuminata GBIF Data set: https://doi.org/10.15468/dl.nhbs3u
Ottelia alismoides GBIF Data set: https://doi.org/10.15468/dl.9n24x3
Ottelia balansae GBIF Data set: https://doi.org/10.15468/dl.wkv6zr
Ottelia brasiliensis GBIF Data set: https://doi.org/10.15468/dl.grsbhp
Ottelia cordata GBIF Data set: https://doi.org/10.15468/dl.bwkw4n
Ottelia cylindrica GBIF Data set: https://doi.org/10.15468/dl.ncjp28
Ottelia exserta GBIF Data set: https://doi.org/10.15468/dl.v2aebv
Ottelia fischeri GBIF Data set: https://doi.org/10.15468/dl.7bzdda
Ottelia javanica GBIF Data set: https://doi.org/10.15468/dl.9dgnap
Ottelia kunensis GBIF Data set: https://doi.org/10.15468/dl.e47va7
Ottelia mesenterium GBIF Data set: https://doi.org/10.15468/dl.6p8qdx
Ottelia muricata GBIF Data set: https://doi.org/10.15468/dl.p4yhfu
Ottelia ovalifolia GBIF Data set: https://doi.org/10.15468/dl.5pdqds
Ottelia ulvifolia GBIF Data set: https://doi.org/10.15468/dl.x5dm62
Ottelia verdickii GBIF Data set: https://doi.org/10.15468/dl.csnbg2

Stratiotes:
Stratiotes aloides GBIF Data set: https://doi.org/10.15468/dl.stx76u

Thalassia:
Thalassia GBIF Data set: https://doi.org/10.15468/dl.82h3sg

Vallisneria:

Vallisneria americana GBIF data set: https://doi.org/10.15468/dl.wqf33a
Vallisneria annua GBIF data set: https://doi.org/10.15468/dl.cq9ngt
Vallisneria australis GBIF data set: https://doi.org/10.15468/dl.cq9ngt
Vallisneria caulescens GBIF data set: https://doi.org/10.15468/dl.2m7v5v
Vallisneria denseserrulataGBIF data set: https://doi.org/10.15468/dl.ukg4ed
Vallisneria erecta GBIF data set: https://doi.org/10.15468/dl.rdyx2b
Vallisneria natans GBIF data set: https://doi.org/10.15468/dl.wkhqv4
Vallisneria nana GBIF data set: https://doi.org/10.15468/dl.hvka99
Vallisneria rubra GBIF data set: https://doi.org/10.15468/dl.knjwsx
Vallisneria spinulosa GBIF data set: https://doi.org/10.15468/dl.j364pd
Vallisneria spiralis GBIF data set: https://doi.org/10.15468/dl.72599d
Vallisneria triptera GBIF data set: https://doi.org/10.15468/dl.8vs4g9

References:

Chen, L.-Y., Chen, J.-M., Gituru, R. W., Wang, Q.-F. 2012. Gerneric phylogeny, historical biogeography and character evolution of the cosmopolitan aquatic plant family Hydrocharitaceae. BMC Evolutionary Biology, 12, 30.

Cui, D., Liang, S., Wang, D., Liu, Z. 2021. A 1 km global dataset of historical (1979–2013) and future (2020–2100) Köppen–Geiger climate classification and bioclimatic variables. Earth Syst. Sci. Data, 13, 5087–5114. https://doi.org/10.5194/essd-13-5087-2021

Kottek, M., Grieser, J., Beck, C., Rudolf, B., and Rubel, F. 2006. World map of the Köppen-Geiger climate classification updated, Meteorol. Z., 15, 259–263.

Peel, M. C., Finlayson, B. L., and McMahon, T. A. 2007. Updated world map of the Köppen-Geiger climate classification, Hydrol. Earth Syst. Sci., 11, 1633–1644, https://doi.org/10.5194/hess-11-1633-2007.

Rubel, F., Brugger, K., Haslinger, K., and Auer, I. 2017. The climate of the European Alps: Shift of very high resolution Köppen- Geiger climate zones 1800–2100, Meteorol. Z., 26, 115–125, https://doi.org/10.1127/metz/2016/0816.
