# Supplementary material for: Origin and Early Evolution of Hydrocharitaceae and the Ancestral Role of Stratiotes
Source: Plants (Basel). 2024 Mar 31;13(7):1008. doi: 10.3390/plants13071008 (PMC11013807; doi:10.3390/plants13071008)
Supplement: Supplementary file 1 [file plants-13-01008-s001.zip › Supplementary_material_03.pdf]

Supplementary material 03

Authors: Silvia Ulrich, Manuel Vieira, Mario Coiro, Johannes M. Bouchal, Christian Geier, Bonnie F. Jacobs, Ellen D. Currano, Olaf K. Lenz, Volker Wilde, Reinhard Zetter, Friðgeir Grímsson

Article title: Origin and early evolution of Hydrocharitaceae and the ancestral role of *Stratiotes*

Content: Additional LM, SEM, and TEM micrographs of *Stratiotes aloides* pollen

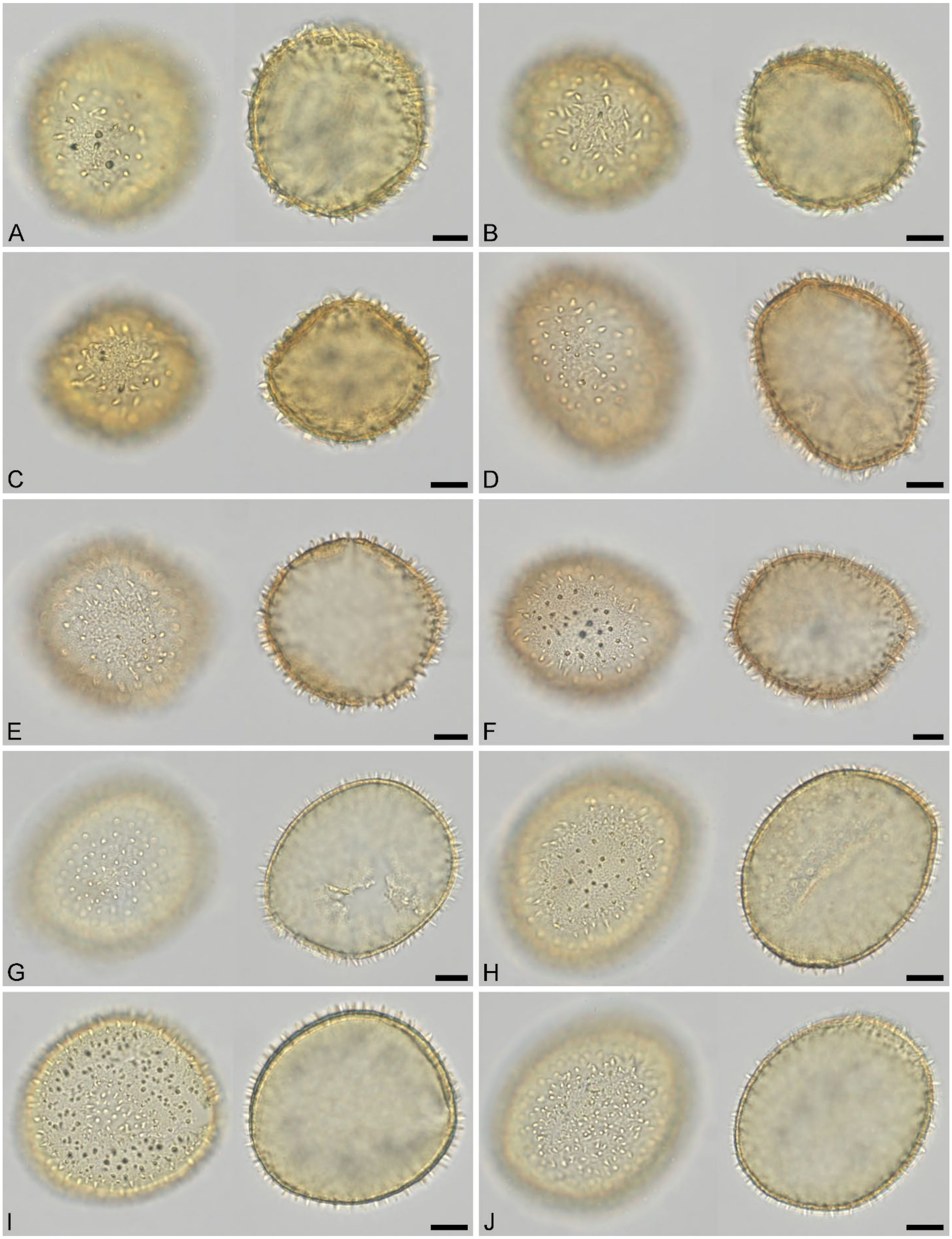

Plate S1. *Stratiotes aloides* pollen from herbaria specimens. LM micrographs. **A–C**. WU0152793. **D–F**. WU0152794. **G–J**. WU0152795. Scale bars – 10 μm (A–J).

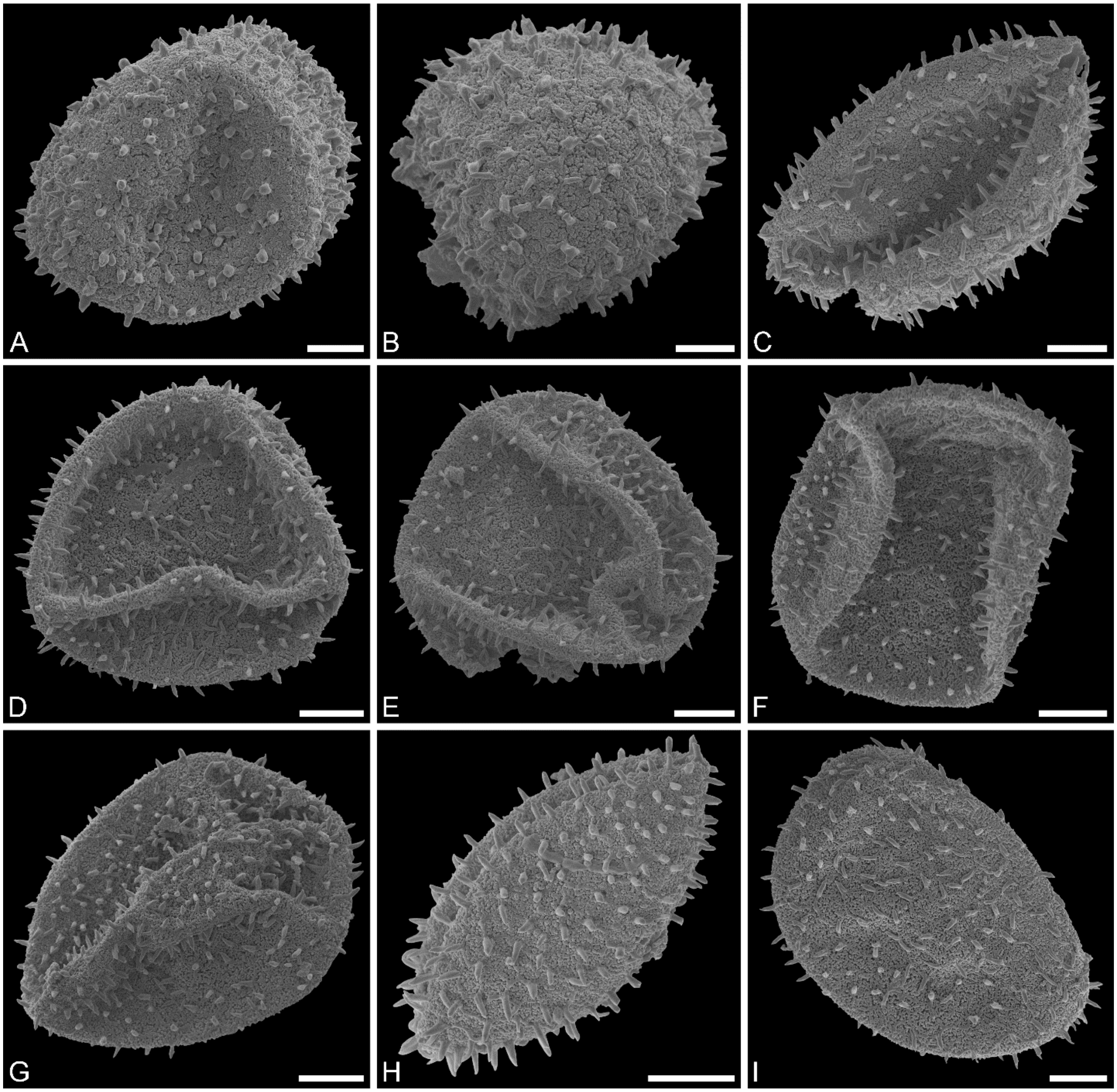

Plate S2. *Stratiotes aloides* L. pollen from herbaria specimens. SEM micrographs. **A, B**. WU0152793. **C**. WU0152794. **D–I**. WU0152795. Scale bars – 10 μm (A–I).

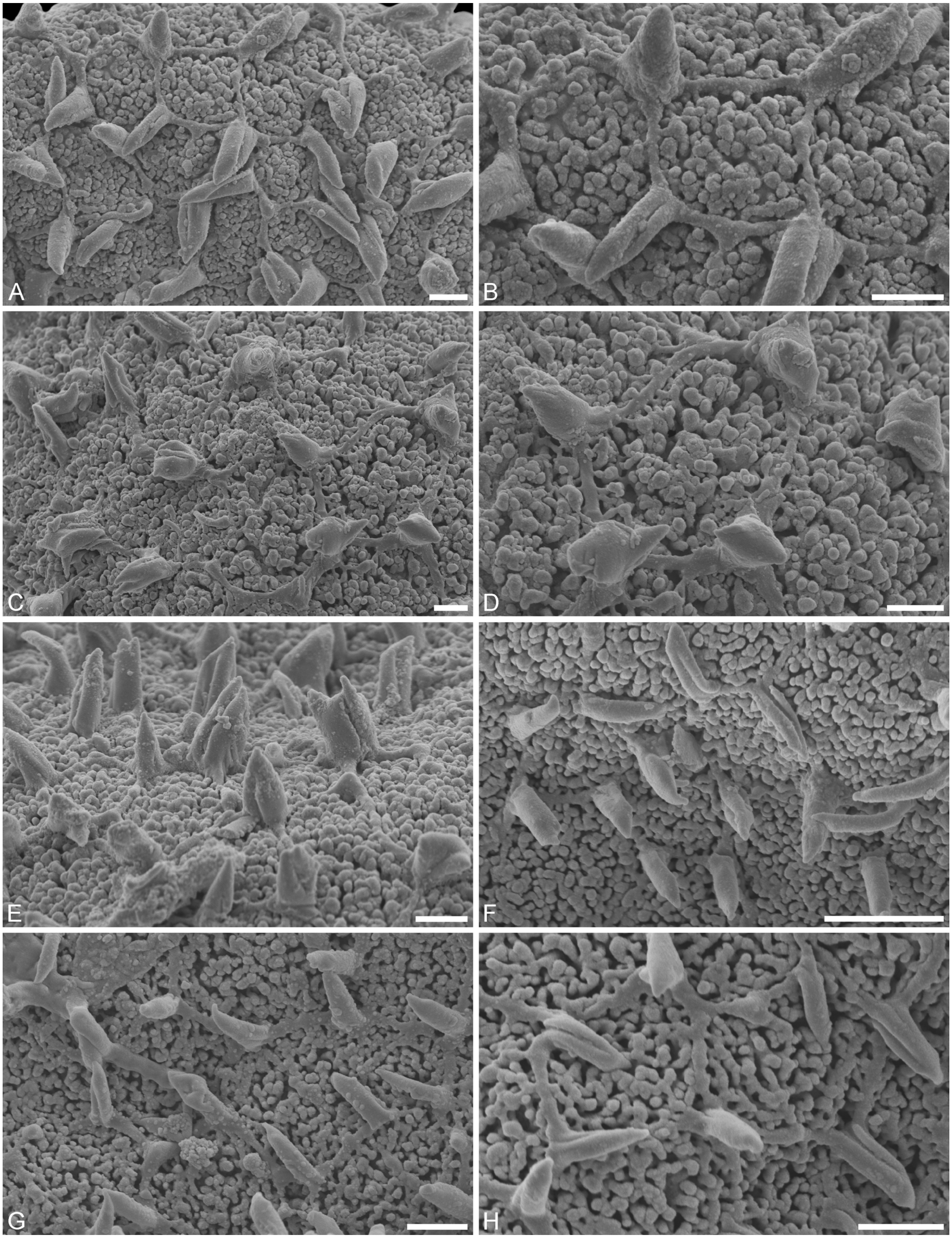

Plate S3. *Stratiotes aloides* L. pollen from herbaria specimens. SEM micrographs of surface sculpture. **A**–**E.** WU0152793. **F.** WU0152794. **G**–**H.** WU0152795. Scale bars – 2 µm (A–H).

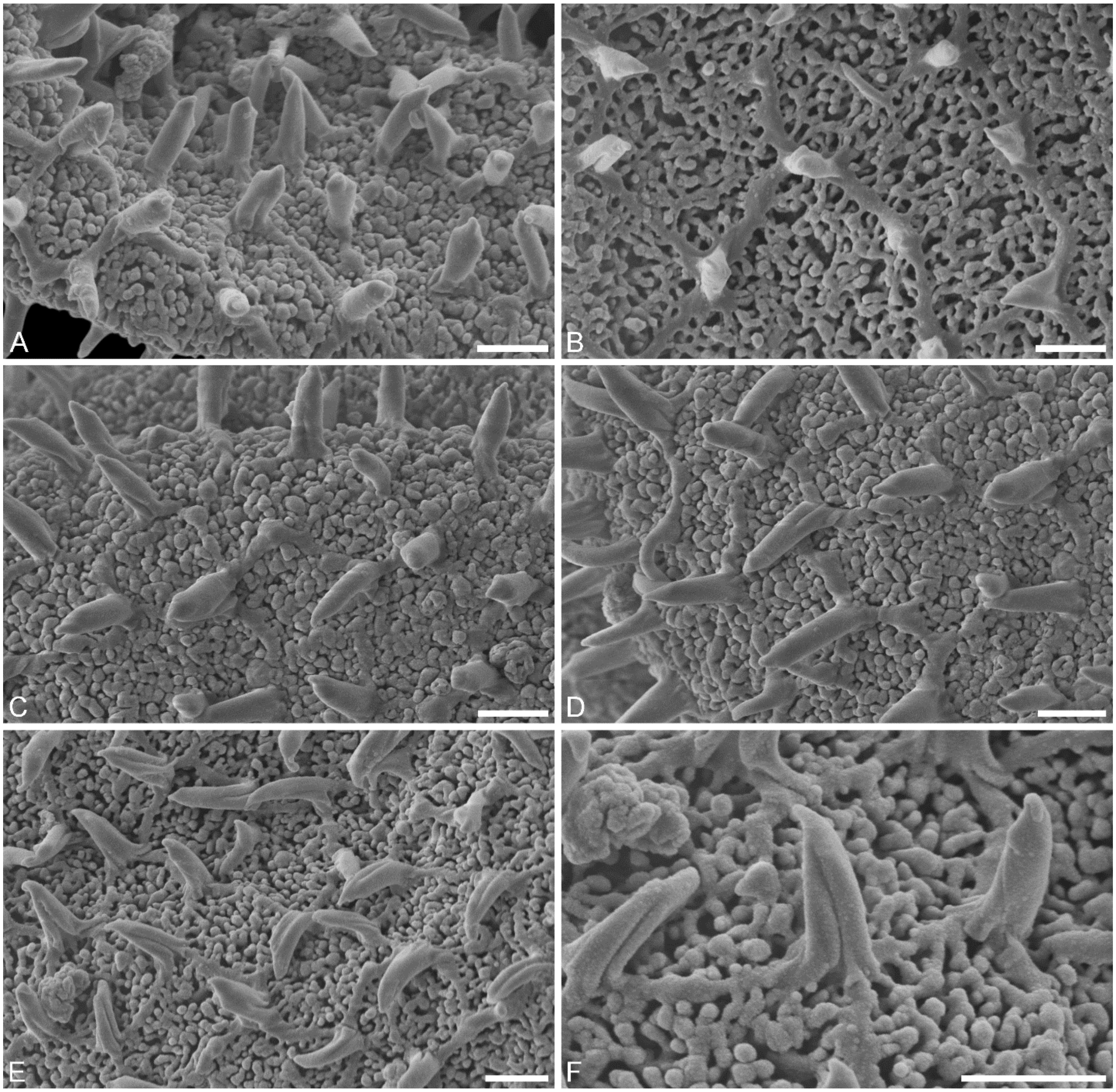

Plate S4. *Stratiotes aloides* L. pollen from herbaria specimens. SEM micrographs of surface sculpture. **A**–**F.** WU0152795. Scale bars – 2 µm (A–F).
