# Supplementary figures and images for: Origin and Early Evolution of Hydrocharitaceae and the Ancestral Role of Stratiotes
Source: Plants (Basel). 2024 Mar 31;13(7):1008. doi: 10.3390/plants13071008 (PMC11013807; doi:10.3390/plants13071008)

# Bayesian

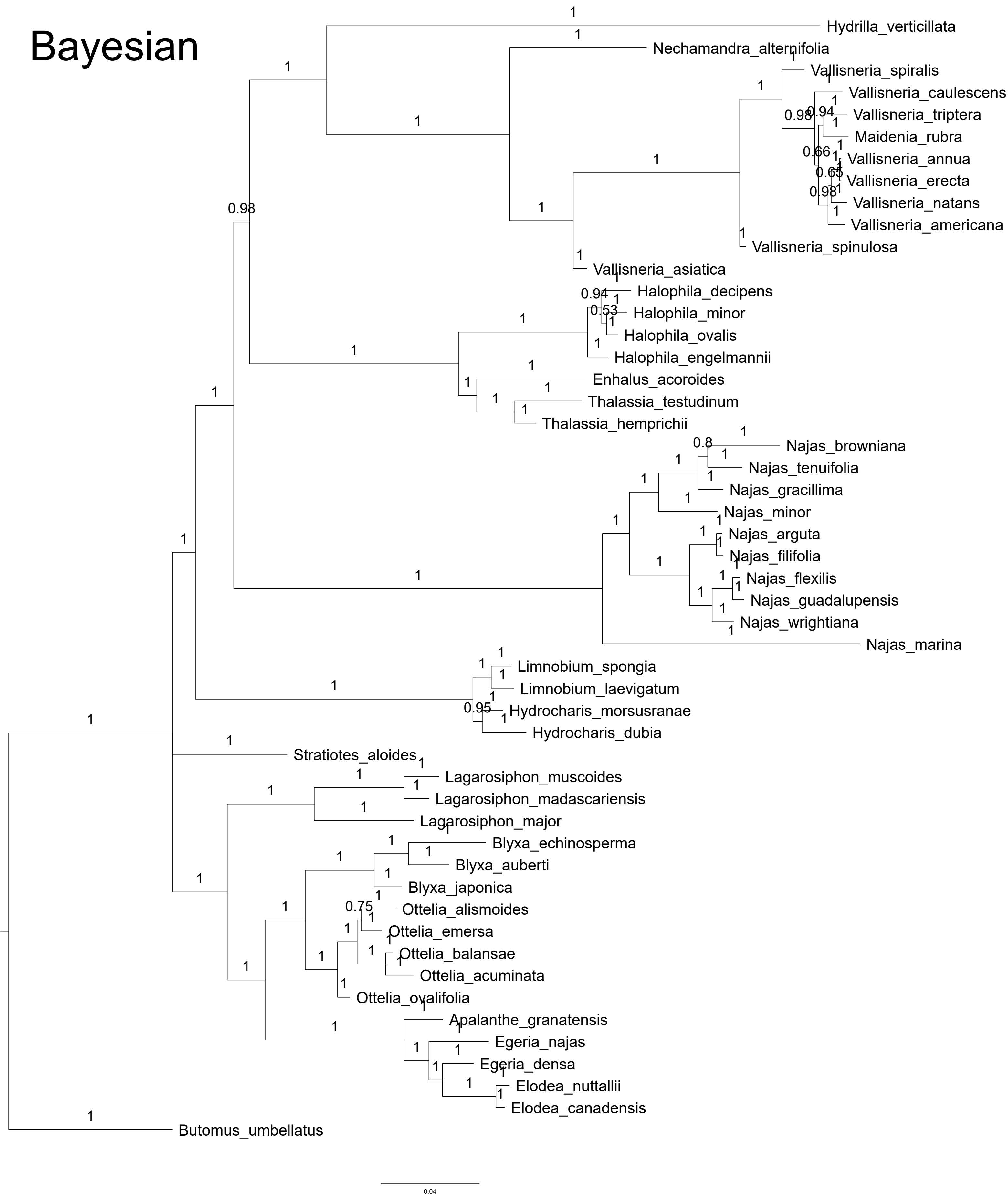

# IQTREE

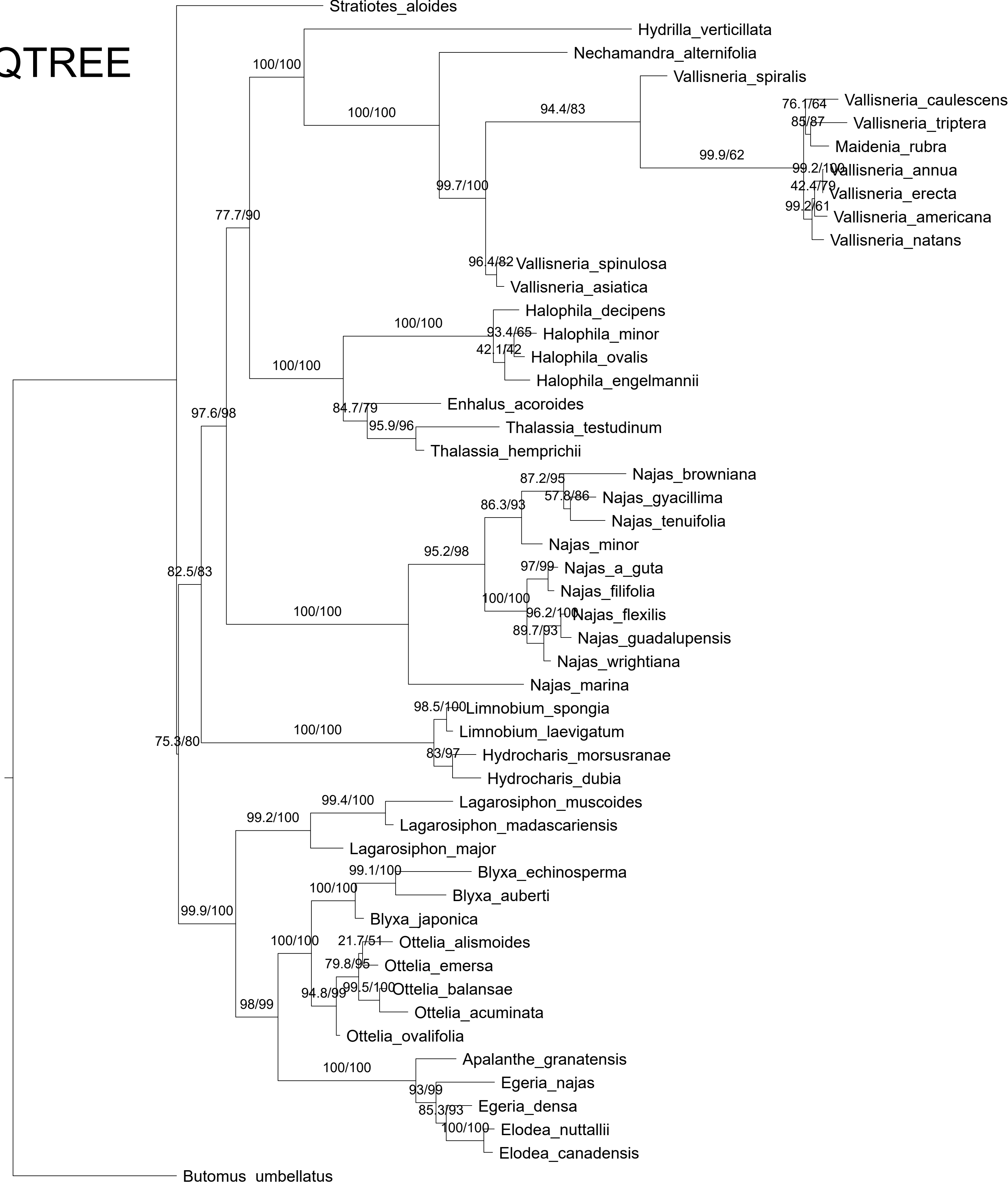

Supplement: Supplementary file 1 [file plants-13-01008-s001.zip › Supplementary_material_04.pdf]
